# Enantioselective Hydration of Non-CoA Enoyl-Thioesters by Enoyl-CoA Hydratase (ECH): Activation of the Active Site Oxyanion Hole with 3′,5′-Adenosine-Diphosphate Enables Competent Catalysis

**DOI:** 10.1021/jacsau.6c00054

**Published:** 2026-03-23

**Authors:** Subhadra Dalwani, Pradip Kumar Mondal, Werner Schmitz, Rik K. Wierenga, Petri M. Pihko

**Affiliations:** † Faculty of Biochemistry and Molecular Medicine, 6370 University of Oulu, P.O. Box 5400, Oulu FI-90014, Finland; ‡ Department of Chemistry and NanoScience Center, 4168 University of Jyväskylä, P.O.Box 35, Jyväskylä FI-40014, Finland; § Institute of Biochemistry and Molecular Biology, 9190 University of Würzburg, Würzburg DE-97070, Germany

**Keywords:** hydratase reactions, pantetheine, thioesters, oxa-Michael addition

## Abstract

Thioester chemistry is exploited in Nature by many CoA-dependent enzymes. However, the covalent nature of CoA attachment largely prevents the use of these enzymes in many applications. Replacing the CoA moiety with simpler, truncated fragments, such as its pantetheine (PAN) moiety, is also hampered by the lack of understanding of the function of the CoA moiety in enzymatic conversions. Herein, we describe the utilization of the enzyme (2*E*)-enoyl-CoA hydratase (ECH) using PAN thioesters and an activator, 3′,5′-ADP (PAP). ECH catalyzes the hydration of the carbon–carbon double bond of (2*E*)-enoyl-CoA substrates in the β-oxidation lipid-degrading pathway. The hydration reaction is very challenging to carry out by traditional chemical synthesis, as no selective catalysts are available. Structural enzymology of ECH and its complexes with (3*S*)-hydroxyacyl-CoA products show that hydrogen bonds between the adenine 6-amino group of the ADP moiety of CoA and loop-2 induce a small structural change in this active site loop, tightening the NN distance between the hydrogen bond donors of the oxyanion hole from 5.2 Å (unliganded) to 4.0 Å and forming a competent oxyanion hole at the catalytic site. A structurally similar and catalytically competent oxyanion hole is observed in the complex with (3*S*)-hydroxyhexanoyl PAN and the activator 3′,5′-ADP, both bound at the active site. The use of 3′,5′-ADP as the activator enables the synthetic use of ECH for the hydration of a wide range of (2*E*)-enoyl-PAN substrates with different steric demands and functionalities. The products, 3-hydroxyacyl-PAN thioesters, were obtained in good isolated yields and excellent stereoselectivities (typically >99:<1 3*S*:3*R*). Even for acyl chains that contain reactive groups such as bromide or methyl ester functionalities at C7, no side products resulting from potentially competing cyclization could be detected in the enzymatic hydration protocol.

## Graphical abstract

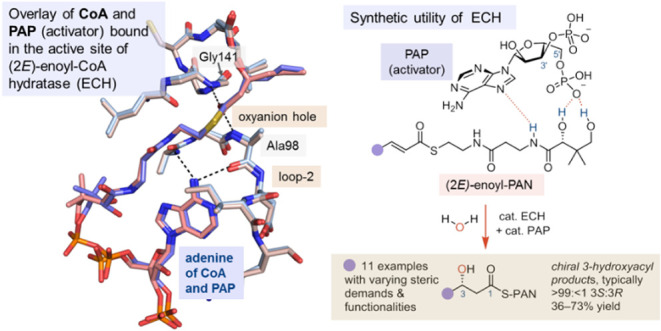

## Introduction

The catalytic, enantioselective addition of water to α,β-unsaturated carbonyl functionalities is a well-established reaction in biocatalysis, but remains elusive with synthetic catalysts. In biocatalysis, the most prominent examples include the hydration of fumarate by fumarase and the hydration of enoyl-CoA thioesters by enoyl-CoA hydratase,^1^ (ECH, Scheme 1a). ECH (E.C. 4.2.1.17) has a wide substrate scope, accepting a range of linear fatty acid tails, and excellent enantioselectivity,^2^ thus rendering it a potentially attractive enzyme for synthetic applications.

**Scheme 1:**
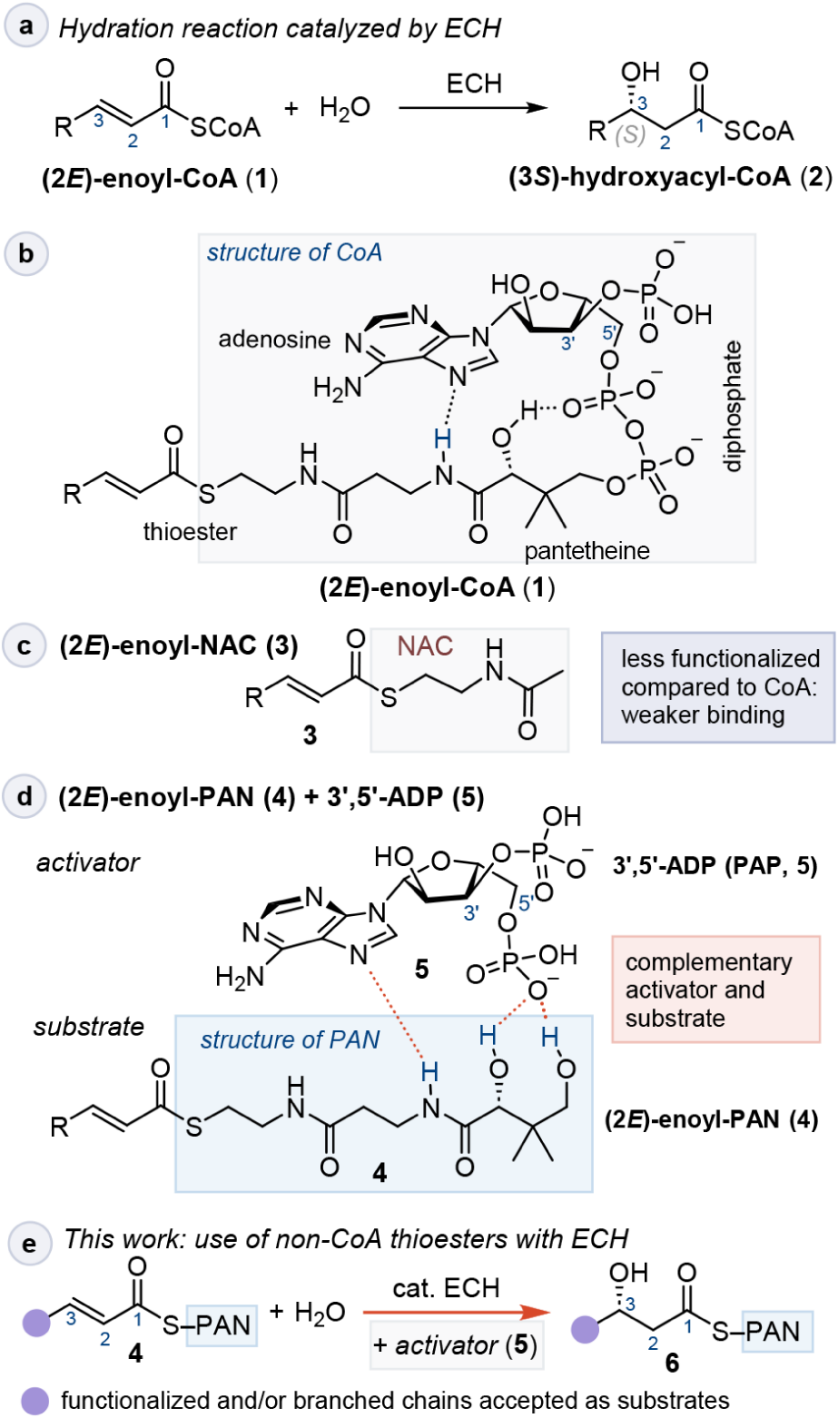
a) The (2*E*)-Enoyl-CoA Hydratase Reaction Catalyzed by ECH; b) Structure of (2*E*)-enoyl-CoA in its Bent Conformation, as Bound to ECH. Dotted Lines Mark Favorable Intramolecular Interactions; c) Structure of (2*E*)-enoyl-NAC; d) Structures of (2*E*)-enoyl-PAN and Activator 3′,5′-ADP (PAP), also Showing the Interactions between PAN and PAP in the Structure of the PAN+PAP Complex, as Shown in this Work; e) Synthetic Utility of the Hydratase Reaction Catalyzed by ECH with the (2*E*)-Enoyl-PAN Substrate in the Presence of Activator **5**

Activating the α,β-unsaturated carbonyl substrate toward nucleophilic addition of water requires polarization of the carbonyl substrate at the active site with an oxyanion hole.^1^ At the same time, the single water molecule must be activated by a general base. To meet both of these requirements, the substrate must be tightly bound to the complementary active site. The challenge is especially acute with enoyl (thio)­esters, since the lipophilic substrate and the thioester group may not be able to contribute significantly to binding. In Nature, this problem is solved by using CoA thioesters, with the CoA serving as an anchor (Scheme 1b). From a synthetic point of view, the stoichiometric use of expensive CoA thioesters is undesirable, given the high polarity and molecular weight of CoA. A more attractive option would be to use truncated variants of CoA as substrates, such as *N*-acetyl-cysteamine (NAC) thioesters (Scheme 1c), but the small size of NAC may prevent sufficient binding and catalytic activity.

It was reported in 1955 by Lynen and coworkers that for the truncated substrates (2*E*)-enoyl-PAN and (2*E*)-enoyl-NAC catalytic activity of ECH was not detectable.^3^ However, in 1972, Waterson, Hass and Hill noted that the activity of bovine ECH toward the truncated CoA derivative (2*E*)-butenoyl-pantetheine ((2*E*)-butenoyl-PAN, Scheme 1d), could be partially restored by additives such as ATP or CoA.^4^ Later studies by Bahnson and Anderson confirmed this observation, and they successfully used 3′,5′-ADP (**5**), also known as PAP (Scheme 1d), as an activator for ECH with the (2*E*)-butenoyl-PAN substrate to enable access to isotopologues of products for mechanistic studies.^5,6^ However, these studies did not address the question *why* these activators restore the activity of ECH for PAN thioester substrates. Furthermore, given the potential utility of the chiral 3-hydroxyacyl units such as **6** (Scheme 1e) in the synthesis of polyketide natural products, it is surprising that the synthetic utility of the enoyl hydratase reaction catalyzed by ECH with alternative thioesters has not been explored.^7^

Herein we report new crystallographic studies of rat mitochondrial ECH in its unliganded state as well as in the presence of (2*E*)-enoyl-CoA or (2*E*)-enoyl-PAN substrates plus the 3′,5′-ADP activator (PAP, **5**), showing that in the unliganded state, the oxyanion hole is not formed. Only in the presence of the adenine moiety, either of the (2*E*)-enoyl-CoA substrate or of the activator, the competent oxyanion hole is observed, restoring the catalytic activity of ECH. Furthermore, we also show that replacing expensive and hydrophilic CoA thioesters (Scheme 1a) with more readily accessible pantetheine (PAN) thioesters enables a synthetic protocol for ECH catalyzed hydration of (2*E*)-enoyl-PAN thioesters in the presence of cocatalyst PAP (Scheme 1e). The hydration reactions proceed with exceptional chemo- and near-perfect enantioselectivity, leaving potentially reactive functionalities in the substrate (such as primary halide, silyl ether or ester groups) completely unaffected.

## Results and Discussion

### Enzymology

In particular, linear short chain (2*E*)-enoyl-CoA thioesters are very efficiently hydrated by ECH.^1,8^ For example, for rat mitochondrial ECH, the *k*_cat_ and *K*_M_ values for the substrate (2*E)*-hexenoyl-CoA are 1101 ± 80 s^–1^ and 23 ± 4 μM, respectively (Table S1, Figure S1). ECH is also known to catalyze the hydration of (2*E*)-enoyl-CoA substrates of which the acyl-tail is branched in various ways,^9,10^ although the efficiency of hydration of these compounds is not well studied. The enoyl hydration reaction involves an equilibrium where the product **2** is the main component (the equilibrium ratio is ca. 3.5:1).^11^ The catalytic efficiency of ECH toward (2*E*)-enoyl-PAN thioesters has only been studied with the (2*E*)-butenoyl-PAN substrate.^4^ To compare the efficiency of different activators, we used (2*E*)-hexenoyl-PAN (**4a**) instead of (2*E*)-hexenoyl-CoA (**1a**) as the substrate. Figure 1 presents the results with different activators, showing the linear part of the Michaelis–Menten curve for **4a** in the presence of several activators as well as without activator. Clearly 3′,5′-ADP (PAP, **5**) is the best activator, better than CoA or ATP, ADP, AMP. Replacing CoA with PAN in the substrate (**1a** vs **4a**) resulted in approximately 10^6^-fold drop in *k*_cat_/*K*_M_ (48 × 10^6^ M^–1^ s^–1^ for **1a** vs 54 M^–1^ s^–1^ for **4a**), corresponding to a ΔΔ*G* ^‡^ difference of the transition state barrier of 8.1 kcal mol^–1^ (Table S2). Including 100 μM of activator **5** restored *k*_cat_/*K*_M_ to 780 M^–1^ s^–1^, reducing the ΔΔ*G* ^‡^ of the barrier by 1.6 kcal mol^–1^ (Table S2).

**Figure 1:**
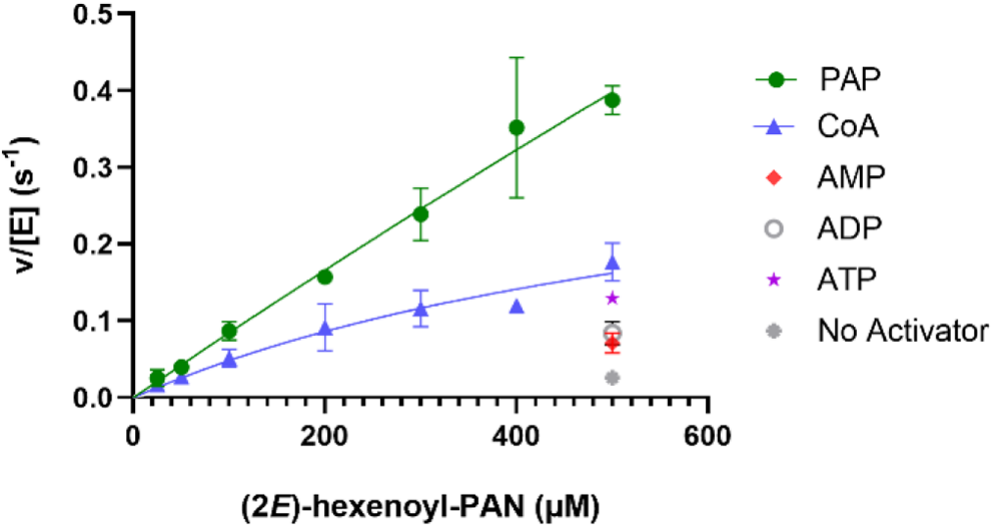
Effect of different activators (100 μM) on the initial rate of hydration, *v*, by ECH for (2*E*)-hexenoyl-PAN as substrate, plotted as *v*/[E] versus substrate concentration. The error bars visualize the range of the plotted rates as obtained from three different data sets.

### Protein Crystallographic Binding Studies

ECH is a homohexameric enzyme (a dimer of trimers) (Figure 2a) of which each subunit adopts the crotonase fold.^12^ Many enzymes of this superfamily are CoA dependent enzymes catalyzing a wide variety of reactions. The CoA is bound in a characteristically bent conformation (Figure 2a), such that the thioester oxygen binds in an oxyanion hole generated by two peptide NH-groups. This oxyanion hole stabilizes the negative charge on the thioester oxygen as it develops in the catalytic cycle (Figure 2b) and various studies have shown the importance of this interaction for efficient catalysis.^1,13^ Previous structural studies of rat mitochondrial ECH have resulted in four crystal structures of this enzyme as complexes with (i) acetoacetyl-CoA (a tight inhibitor, PDB ID 1DUB), (ii) octanoyl-CoA (a nonreactive substrate analogue, PDB ID 2DUB), (iii) (2*E*,4*E*)-hexadienoyl-CoA (a possible substrate, PDB ID 1MJ3) and (iv) 4-(*N*,*N*-dimethylamino)-cinnamoyl-CoA (a possible substrate, PDB ID 1EY3). In the latter two ligands, the (2*E*)-double bond is part of a conjugated system, and as a result the equilibrium of the hydration reaction strongly favors the substrate, not the product.^2,14^ Consistent with this, the latter two structures have indeed captured the enzyme in which the active site is complexed with the substrate, in the presence of the bound catalytic water, as schematically visualized in Figure 2b.

**Figure 2:**
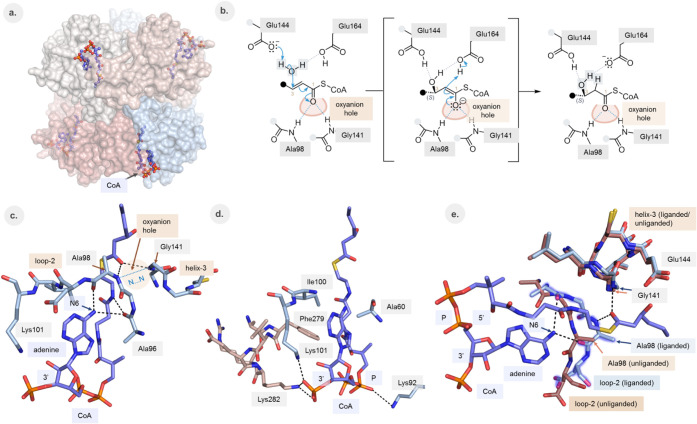
Structures of the liganded (PDB ID 9RGS) and unliganded (PDB ID 9RGQ) active site of ECH. The ligand (3*S*)-hydroxyhexanoyl-CoA (**2a**) is shown in thick, blue sticks. The 6-amino group of the adenine moiety of the ligand is labeled “N6”. Hydrogen bonds are visualized by black dotted lines. (a) ECH is a hexamer, being a dimer of two trimers, with six active sites. The CoA moiety is bound in a bent conformation at the interface of two subunits. (b) The reaction catalyzed by ECH. In the hydration reaction, Glu144 activates the catalytic water and Glu164 donates the proton to the C2 atom.^1^ (c) The 6-amino group of the adenine moiety of CoA is hydrogen bonded to loop-2 by hydrogen bonds with O­(Ala96) and O­(Ala98). N···N identifies the NN distance between N­(Ala98) and N­(Gly141) (blue dotted line). Helix H3 is labeled as helix-3. (d) The mode of binding of the 3′-phosphate-ADP moiety of CoA in its binding pocket. The adenine moiety is wedged between the side chains of Ala60, Ile100 and Phe279 (of helix H10 of the other subunit). Three favorable electrostatic salt bridge interactions between CoA and the protein are highlighted by dotted lines. (e) Superposition of the liganded structure (light blue) and unliganded structure (chocolate). The catalytically competent position of loop-2 of the liganded structure is highlighted by blue glow. Zoomed-out and zoomed-in versions of this superposition are shown in Figure S3 and Figure S4, respectively.

These previously determined structures show that in the active site of ECH, the side chains of the two catalytic glutamates are hydrogen bonded to the catalytic water, being positioned on the *Si*-face of the C3 carbon atom of the (2*E*)-enoyl-CoA substrate, which is bound in the *s-cis* conformation. Glu144 activates the water molecule for nucleophilic attack, by which the C3 carbon is hydroxylated. The negative charge of the resulting planar enolate intermediate is stabilized by the oxyanion hole. Subsequently, a proton is donated to the C2 atom by Glu164 (Figure 2b),^1,8^ completing the *syn* hydration of the (2*E*)-enoyl-CoA substrate,^15^ and providing the (3*S*)-hydroxyacyl-CoA product. The oxyanion hole is formed by the peptide NH groups of Ala98 (located in loop-2) and Gly141 (at the N-terminus of the active site helix, helix H3) of the crotonase fold (Figure 2c). These structures also show the conformational flexibility of the substrate specificity loop, residues 112–121, which covers the acyl-tail binding pocket, as well as the position of the C-terminal helix H10, which covers the active site. In addition, salt bridge interactions between the substrate and the protein (Figure 2d), as well as favorable interactions between the pantetheine and the PAP segment of the bound CoA (Scheme 1b) are also evident from the previous work. However, the complex of ECH with the hydrated product bearing the (3*S*)-hydroxyacyl chain has not been previously characterized. Herein, we report crystallographic binding studies of ECH with substrates (2*E*)-butenoyl-CoA, (2*E*)-hexenoyl-CoA, (2*E*)-decenoyl-CoA and (2*E*)-hexenoyl-PAN (in the presence of the PAP activator), capturing the (3*S*)-hydroxyacyl product. For comparison, the X-ray structure of unliganded ECH was also determined.

High quality crystals of the ECH-CoA complex, obtained by cocrystallization of ECH in the presence of CoA, having one hexamer per asymmetric unit,^16^ were used for the crystallographic binding studies. For the soaking protocol with the three (2*E*)-enoyl-CoA substrates, the CoA of the mother liquor was replaced by the (2*E*)-enoyl-CoA substrate. The structures obtained show that in each case the substrate is hydrated into the respective (3*S*)-hydroxyacyl-CoA product: (3*S*)-hydroxybutanoyl-CoA (**2**), (3*S*)-hydroxyhexanoyl-CoA (**2a**), or (3*S*)-hydroxydecanoyl-CoA (**2b**), capturing the enzyme–product complexes (schematically shown in Figure 2b) at 2.7 Å, 1.7 Å, and 2.0 Å resolution, respectively (Figure S2, Table S3). Additionally, the ECH-CoA crystals, were also used for soaking experiments in which CoA was replaced by (2*E*)-hexenoyl-PAN (**4a**), with and without the 3′,5′-ADP activator (PAP, **5**).

The crystallographic analysis of these structures showed that only in the presence of *both* activator PAP (**5**) as well as (2*E*)-hexenoyl-PAN (**4a**), CoA is replaced by bound PAP (**5**) and (3*S*)-hydroxyhexanoyl-PAN (**6a**) (see Figure S2), indicating synergistic binding of (2*E*)-hexenoyl-PAN and PAP. The structure of this PAN+PAP complex was refined at a resolution of 1.7 Å (Table S3). The structure of the unliganded ECH was obtained from a different crystal form grown in the absence of CoA and this structure was refined at a resolution of 2 Å (Figure S2, Table S3).

### The PAP Segment of CoA Fixes Loop-2 in Its Competent Conformation

Comparison of the structures of the active site of unliganded ECH and of ECH complexed with (3*S*)-hydroxyhexanoyl-CoA (as well as with (3*S*)-hydroxybutanoyl-CoA and (3*S*)-hydroxydecanoyl-CoA) shows a small, but critical, structural rearrangement of loop-2 (Figure 2e, Figure S3). In both liganded and unliganded conformations, the structure of this loop is well-defined by the electron density map. The conformational change associated with binding of the ligand mainly involves a rotation of the Gly97-Ala98 peptide unit (Figure S4), and it directly affects the geometry of the oxyanion hole. The rotation brings the two hydrogen bond donors of the oxyanion hole closer to each other: the distance between the main chain N atoms N (Gly141) and N­(Ala98) (the NN distance, Figure 2c) is reduced from 5.2 Å to 4.0 Å. In the absence of the ligand, this causes electrostatic stress, because of the short distance between the partially positively charged hydrogen bond donors (the NH groups of the main polypeptide chain) of the oxyanion hole. Smaller structural changes of loop-2 are observed for residues before and after Ala98, extending from Ala96 to Lys101. The structure of loop-2 of ECH complexed with CoA is stabilized by two hydrogen bond interactions of the amino group of the adenine moiety of CoA with the main chain oxygen atoms of Ala96 and Ala98, respectively (Figure 2c), showing that the presence of the adenine moiety of CoA is critically important for the competent conformation of loop-2.

### The Mechanism of Enzyme Activation by PAP

The soaking experiment of crystals in the presence of (2*E*)-hexenoyl-PAN and PAP resulted in a structure in which the ECH active site is complexed with the hydration product, (3*S*)-hydroxyhexanoyl-PAN as well as with PAP. The modes of binding of (3*S*)-hydroxyhexanoyl-PAN and PAP are the same as observed for the corresponding moieties of (3*S*)-hydroxyhexanoyl-CoA (Figure 3a). The comparison of these active sites also shows that in the presence of PAP loop-2 adopts its competent conformation, as known from the structures of the CoA complexed active site (Figure 3a).

**Figure 3:**
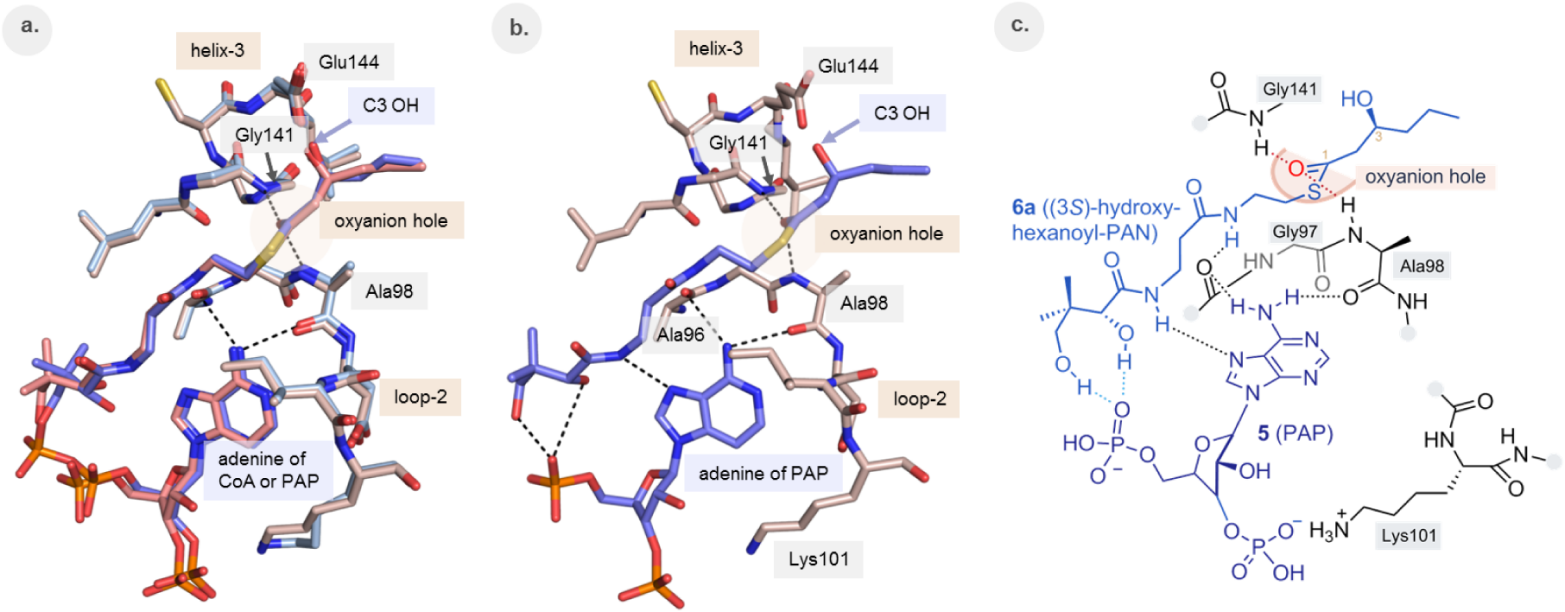
Comparison of the mode of binding of (3*S*)-hydroxyhexanoyl-CoA (**2a**) (PDB ID 9RGS) and (3*S*)-hydroxyhexanoyl-PAN (**6a**) and PAP (**5**) (PDB ID 9RGU). **2a** is shown in light-pink sticks, **6a** and **5** are shown in dark-blue sticks. Hydrogen bond interactions are visualized by dotted lines. (a) Superposition of the active sites complexed with (3*S*)-hydroxyhexanoyl-CoA (**2a**) and complexed with (3*S*)-hydroxyhexanoyl-PAN and PAP (**6a** and **5**), respectively. (b) The structure of the active site complexed with (3*S*)-hydroxyhexanoyl-PAN **6a** and PAP (**5**). (c) Schematic drawing visualizing the hydrogen bond interactions of (3*S*)-hydroxyhexanoyl-PAN (**6a**) with PAP (**5**) as well as with the active site of ECH.

The structure of the PAN+PAP complex explains the critical role of the PAP activator when ECH is used for the hydration of (2*E*)-enoyl-PAN compounds. The crystal structure of the PAN+PAP complex also shows that the interactions between the PAN and ADP moieties, as present in the bent CoA mode of binding are preserved (Scheme 1d, Figure 3b, c), complemented further by an additional hydrogen bond between the terminal hydroxyl group of the PAN moiety of (3*S*)-hydroxyhexanoyl-PAN with the 5′-phosphate of PAP. These favorable interactions explain the synergy of binding of the PAN substrate and the ADP activator, as described previously^4^ and as also observed in the crystallographic binding studies reported here.

The adenine moiety binds in a hydrophobic pocket, shaped by the side chains of Ala60, Ile100, and Phe279 (Figure 2d). The specific interactions of the 6-amino group of adenine with the main chain oxygen atoms of Ala96 and Ala98 of loop-2 explain the observation^4^ that ATP is an activator, whereas the stimulatory effect of ATP is not observed for UTP, CTP, and GTP.

The mode of binding of PAP is also stabilized by two salt bridge interactions between the side chains of Lys101 and Lys282 (of the other subunit) and the 3′-phosphate of PAP (Figure 2d). This is consistent with the observation that PAP is a better activator than AMP, ADP and ATP (Figure 1), none of which include the 3′-phosphate. As shown in Figure 2d, in the corresponding CoA complex there are three salt bridges, with the third salt bridge observed between Lys92 and the additional pantetheine-phosphate of CoA (Scheme 1b), which is not present in PAP (Scheme 1d).

### The Oxyanion Hole of ECH

The induced formation of the competent oxyanion hole on binding of the adenine moiety of CoA, as found here for ECH, is not a common feature of all CoA dependent crotonase fold enzymes. For example, this conformational switch is not found in Δ^3^,Δ^2^‑enoyl-CoA isomerase (ECI) (Table S4), which catalyzes the interconversion of (3*E*)-enoyl-CoA and (2*E*)-enoyl-CoA, and which also belongs to the family of CoA dependent crotonase fold enzymes.^17^

In the ECI active site, there is only one catalytic glutamate, which corresponds to either Glu164 of ECH or to a residue from another loop region near the active site, corresponding to Gly172 of ECH. The ECI catalytic glutamate is proposed to shuttle a proton from the C2-carbon (of the (3*E*)-enoyl-CoA substrate) to the C4-carbon (of the (2*E*)-enoyl-CoA product). In both ECH and ECI-catalyzed reactions, the intermediate is a planar enolate having a partial negative charge on the thioester oxygen atom.

A comparison of the oxyanion hole geometry of ECH and ECI (Table S4) shows that in the competent, ligand bound, ECH active site, the NN distance between the two peptide-NH hydrogen bond donors of the oxyanion hole is shorter than in the ECI active site. The ECH active site geometry must allow for an efficient formation of the (3*S*)-hydroxy enolate intermediate in the first step of the reaction, but must also allow for the efficient protonation of the C2 carbon in the second half of the reaction (Figure 2b). The side chains of both catalytic glutamates are anchored to the main chain by hydrogen bonds and the conformation of these side chains is the same for the unliganded and liganded active site (Figure 4). The details of the mechanism by which the C2-atom is protonated in the second step of the reaction are not completely established.^1,18^ In any case, it is proposed that the side chain oxygen atom of Glu164 donates this proton. This oxygen atom, which is at 3.6 Å distance from the thioester oxygen atom (Figure 4) develops then a negative charge. This negative charge is located close to the negatively charged enolate thioester oxygen atom of the reaction intermediate (Figure 2b), causing additional electrostatic stress in the active site. Electrostatic interactions in active sites are important factors for the catalytic properties of enzymes,^19–21^ and in ECH, fixing the thioester oxygen atom in a rigid oxyanion hole might be a key factor allowing for the hydration reaction to proceed efficiently.^22^

**Figure 4:**
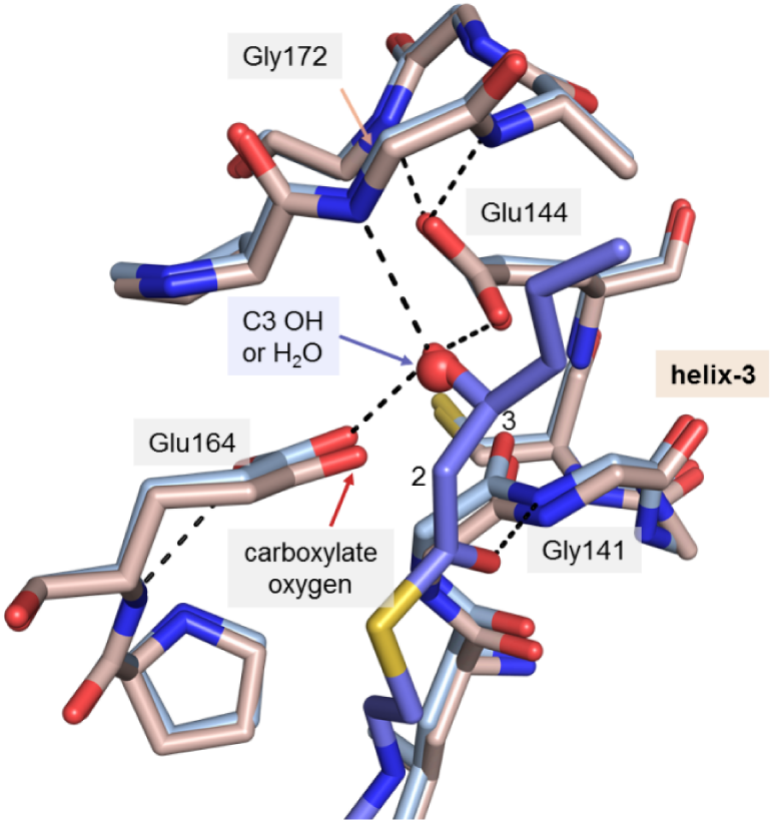
Comparison of the catalytic center of the unliganded active site (light-pink) and the liganded active site (light blue) of the crystal structure of ECH obtained in the presence of (3*S*)-hydroxyhexanoyl-CoA (**2a**) (PDB ID 9RGS). In this crystal form one active site is unliganded due to crystal contacts (Table S4). (3*S*)-hydroxyhexanoyl-CoA **2a** (as bound in the liganded active site) is in thick, blue sticks, with C2 and C3 atoms of the ligand labeled as “2” and “3”, respectively. The catalytic water (as bound in the unliganded active site) is shown as a red sphere. Hydrogen bonds are shown by dotted lines for the liganded active site. The Glu164 side chain oxygen atom (red arrow) is proposed to donate a proton to the C2 carbon in the last step of the hydration reaction (see Figure 2b).

### PAP Preorganizes the Active Site in the Same Way as the PAP Segment of CoA

The binding of the PAP segment of CoA stabilizes the competent conformation of the oxyanion hole of ECH, although the adenosine moiety of CoA is not a reactive part of the substrate. The mechanism of activation by the PAP activator and by the PAP segment of CoA is the same, as the structural studies show that it is bound to the enzyme active site in the same way (Figure 3a). The hydrogen bond interactions of the 6-amino group of the adenine moiety of CoA and of PAP with two peptide oxygen atoms of loop-2 induce the competent conformation of this loop, thereby rigidifying the geometry of the oxyanion hole as is required for catalysis. The preorganization of oxyanion hole geometry is also important for the activity of many other enzymes (and therefore also for enzyme design).^23^ The use of binding energy of nonreactive parts of the substrate for forming an energetically unfavorable active site geometry that stabilizes the high energy transition state of the catalytic cycle, is an important feature of the reaction mechanism of several enzymes, as originally proposed by Jencks.^24,25^ The conformational change of loop-2 of ECH involves a relatively small adjustment in loop-2 (for example the N atom of Ala98 moves by 2 Å), but in other enzymes larger conformational changes have been observed upon substrate binding.^26^ For example, for the enzyme glycerol-3-phosphate dehydrogenase a large (about 10 Å), energetically unfavorable, rearrangement of an active site loop, is shown to be stabilized by the AMP part of NAD^+^.^27^

### Synthetic Manifestation of the ECH-Catalyzed Hydration

Synthetically, several approaches to the enantioselective synthesis of 3-hydroxy (thio)­esters have been disclosed, including enantioselective hydrogenation of β-keto esters,^28^ aldol reactions,^29^ and oxa-Michael additions.^30^ Alternatively, enantioselective addition of water surrogates to Breslow intermediates with *in situ* cleavage of the N–O bond can afford β-hydroxy esters in high enantiopurity.^31^ However, there are no direct catalytic, nonenzymatic methods for oxa-Michael addition of *water* to enoyl (thio)­esters, with or without enantioselectivity. The fact that ECH and other enzymes^32^ are able to simultaneously activate a single water molecule as a nucleophile, and the enoyl substrate as an electrophile is quite remarkable, and presents a challenge for the design of synthetic catalysts. In this study, the catalytic properties of ECH were probed with 11 different (2*E*)-enoyl-PAN substrates.

As established from the Michaelis–Menten analysis (Figure 1), PAP (**5**) is clearly the best activator. While the activity of ECH with (2*E*)-enoyl-PAN (**4a**) and activator (**5)** was not restored to the levels obtained by the native CoA substrate (see the Enzymology section above), the activity was nevertheless fully sufficient for synthetic use. On a preparative scale, we optimized the hydration reaction (Table 1) using **4a** as the substrate and ECH (typically 0.1 mol % enzyme) in pH 7.4 buffer. Pleasingly, using 7.5 mol % PAP as an activator resulted in 72% conversion to **6a** in 1 h (entry 1), and no apparent progress was evident after prolonged reaction times (see the SI for details), suggesting that 2 h incubation time was sufficient to reach maximum conversion. The use of CHCl_3_:IPA as an extraction solvent afforded better recovery of the product from the aqueous medium (entries 1 vs 2), and with this standard protocol, **6a** was obtained 64% isolated yield after purification (entry 1). Control experiments (entries 3 and 4) indicated that both ECH as well as activator **5** were essential for good conversion. Interestingly, ATP (**8**) could also be used as an activator, although only in stoichiometric quantities. Finally, PAN thioester **4a** was superior to NAC thioester **3a** (entry 6), a result which agrees very well with the binding of **6a** and **5** in the enzyme active site, where the pantetheine moiety of **6a** enables several interactions with the activator **5** (see Figure 3 b and c).

**Table 1:**
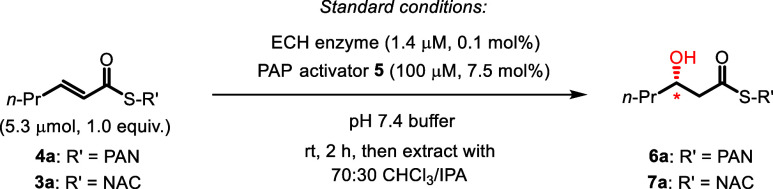
Enantioselective Hydration of (2*E*)-Enoyl-Thioesters with ECH: Standard Protocol with Alternative Activators and Substrates

Entry	Substrate	Deviation from the standard reaction conditions^a^	**4a**:**6a** or **3a**:**7a** ^b^	Conversion to **6a** or **7a** (%)^c^
1	**4a**	none	28:72	72 (64)^d^
2	**4a**	none, but CHCl_3_ as extraction solvent	36:64	64
3	**4a**	no ECH	100:0	0
4^c^	**4a**	no activator	73:27	27
5	**4a**	1.33 mM (100 mol %) ATP (**8**) as activator	25:70	70
6	**3a**	15 mol % activator **5**, 16 h, extraction with CHCl_3_	93:7	7

a Standard reaction conditions: 1.3 mM of **4a** (2.0 mg, 5.3 μmol) or **3a** (1.0 mg, 5.3 μmol), 1.4 μM ECH (0.1 mol %), 100 μM of **5** (7.5 mol %) in 4 mL of 50 mM 2-amino-2-(hydroxymethyl)­propane-1,3-diol (Tris) buffer (pH 7.4)/50 mM KCl, rt, 2 h.

b Ratios of **4a**:**6a** (**3a:7a**) and conversions were determined by ^1^H NMR, using an internal standard (see the SI for details).

c Ratios of **4a**:**6a** (**3a:7a**) and conversions were determined by ^1^H NMR, using an internal standard (see the SI for details).

d Isolated yield (at 53 μmol scale) of **6a**.

The substrate scope of the ECH-catalyzed hydration reaction was then probed by variation of the acyl-tail in **4,** and these experiments are summarized in Scheme 2. The wild-type ECH accepts (2*E*)-enoyl-PAN thioesters with longer chains (**6b**) and substituents/rings in positions 4 and 5 of the tail (**6c**–**6e**), albeit with slightly reduced yields (36–56% compared to 64% yield with **6a**). Pleasingly, ECH accepts PAN thioesters with steric demands further away from the reacting C3 atom (see **6g**, **6i** and **6k**), and even a more bulky *t*-BuMe_2_Si (TBS) group (**6j**), albeit with slightly lower yields. Longer incubation times (4 h or longer) did not improve the yield of **6j.** In addition to accepting substrates with different steric bulk, the functional group tolerance of ECH-catalyzed hydration is excellent, with *N*-Boc (**6e**), primary bromide (**6f**), acetal (**6g**), ester (**6h**), benzyloxy (**6i**) and silyloxy (**6j**) groups, typical handles for functionalization in organic synthesis, are all accepted. While **6f** and **6h** might be expected to suffer from competing cyclization reactions (etherification or lactonization) due to the optimal distance between the C7 functionality and the C3 hydroxy group, no side products resulting from these potential cyclization modes could be identified in the incubation mixtures (see eq 1), although similar side reactions were encountered in the attempted chemical synthesis of the reference (3*RS*)-3-hydroxyacyl products. In all cases, the ECH-catalyzed hydration reactions were very clean, with only product **6** and remaining substrate **4** present in the extracts.
1

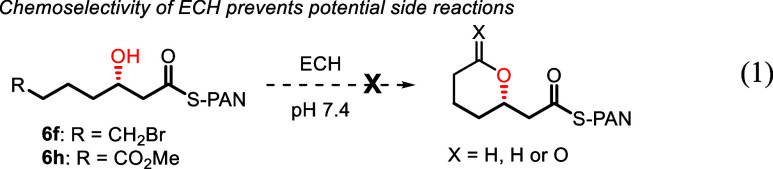




**Scheme 2:**
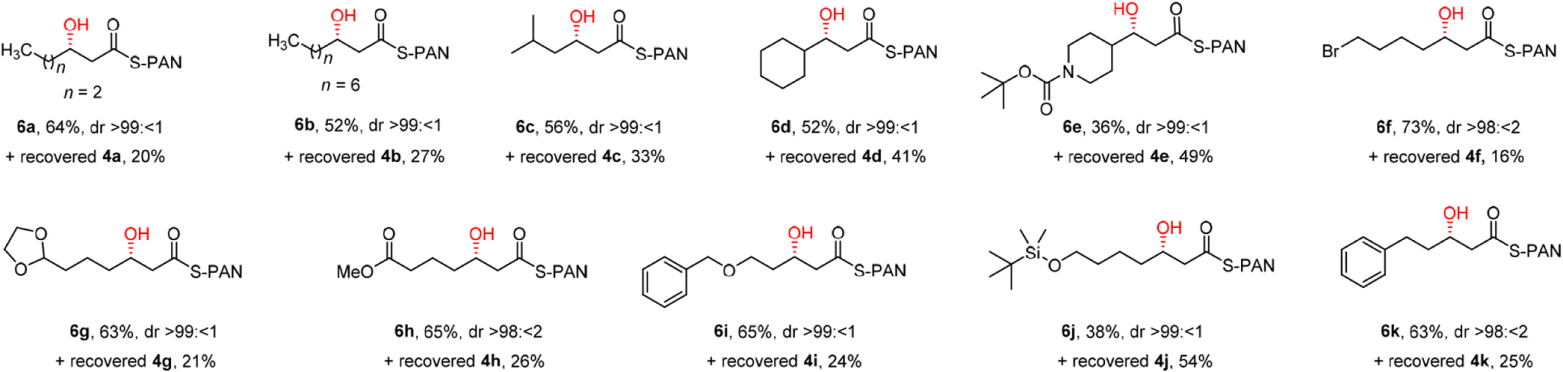
Scope of the enzyme catalyzed hydration reaction^a^

a The reactions were carried out at 0.053 mmol scale using the standard conditions (see Table 1). Reported percentages refer to isolated yields of the product (**6a–6k**) and recovered substrate (**4a–4k**). dr = ratio of (3*S*/3*R*) diastereomers of **6** (assayed by chiral HPLC, see the SI for details)

The stereoselectivities of the hydration reactions were also excellent. The (3*S*/3*R*) diastereomeric ratio (dr) was determined by chiral HPLC, using separately synthesized (3*RS*)-3-hydroxyacyl-PAN thioesters (**6′a**–**6′k**) as reference compounds. In all cases, the ECH hydration products had at least >98:<2 dr, and most products (**6a**–**6e**, **6g**, and **6i**–**6j**) were >99:<1 diastereomerically pure.

It should be noted that the synthesis of functionalized 3-hydroxyacyl reference compounds (**6′a**–**6′k**) was less straightforward than the synthesis of the products **6a**–**6k** using the ECH enzyme, as there are no achiral catalysts available to access the (3*S*/3*R*) mixtures from (2*E*)-enoyl-PAN substrates **4a**–**4k** (see the SI for details of the synthetic routes used for **6′a**–**6′k**). Due to chirality of PAN, the product stereoisomers are diastereomers, although the stereogenic center in PAN is too far away to influence the stereochemical outcome.

## Concluding Remarks

The results of these studies explain the mechanism by which the adenine moiety of the activator allows for the hydration of (2*E*)-enoyl-PAN compounds by ECH. Notably, in the new ECH structures, the enzyme product complexes have been captured, although the crystal soaking experiments were initiated with substrates ((2*E*)-butenoyl-CoA (**1**), (2*E*)-hexenoyl-CoA (**1a**), (2*E*)-decenoyl-CoA (**1b)**, as well as (2*E*)-hexenoyl-PAN (**4a**) together with PAP (**5**)), showing that the catalytic properties of ECH are preserved in the crystalline form. Using the activator enables the biocatalytic properties of ECH to be used for the synthesis of 3-hydroxyacyl-PAN thioesters with high enantiomeric purity using a wide range of (2*E*)-enoyl tails in the substrate. The hydration reaction can also be promoted by low-cost PAP analogues, such as ATP (Figure 1, Table 1). Importantly, ECH, even the wild-type RnECH used in these studies, is highly tolerant of different steric demands and functionalities in the acyl-tail. Ester or bromide substituents did not interfere, even when positioned to allow competing cyclization of the newly formed OH group with the reactant. These results suggest that the substrate acyl-tail remains extended when bound in the acyl-tail-binding tunnel of the active site, away from the general base Glu144. The wide latitude of acyl-tails accepted by wild-type ECH suggests that ECH and related CoA-dependent hydratases can have wider uses in the chemical synthesis of stereoisomerically pure 3-hydroxyacyl building blocks, without the need of using CoA thioesters.

## Experimental Section

### Activity Assay

Enzyme activity was measured with the Jasco V660 spectrophotometer (Jasco, Tokyo, Japan) using a previously reported spectrophotometric direct assay.^33^ All assays were performed at 25 °C using 50 mM Tris, pH 7.5 and 50 mM KCl as reaction buffer with a final volume of 500 μL in quartz cuvettes, with each reaction being monitored for 3 min. The reaction was initiated by mixing enzyme into the reaction buffer. For the assay of (2*E*)-enoyl-CoA, 2 ng of enzyme was added into the cuvette. For the assay with (2*E*)-hexenoyl-PAN (**4a**) 5 μg of enzyme was added into the cuvette. The latter assay was done without activator or in the presence of 100 μM activator, being either CoA, PAP (**5**), ADP, ATP, or AMP. The activity was monitored by measuring the disappearance of the conjugated C=C (C2–C3) of the substrate at λ = 263 nm. Initial reaction rates were determined using the linear part of the progress curve using an absorption coefficient of 6700 M^–1^ cm^–1^. GraphPad Prism version 10 (GraphPad, Boston, USA) was used to plot and analyze the data. The *k*_cat_ and *K*_M_ values for the (2*E*)-enoyl-CoA substrates are the averages of three independent measurements.

### Protein Crystallographic Studies

The two crystal forms of recombinant rat mitochondrial ECH (unliganded and liganded) were obtained at room temperature, using the sitting drop vapor diffusion method and using a 10 mg/mL ECH protein buffer (100 mM potassium phosphate, pH 7.2, 3 mM EDTA). The crystals of unliganded ECH were obtained using a well solution of 0.05 M calcium acetate, 0.1 M sodium cacodylate, pH 6.0, 25% v/v MPD of the commercial screen MD1-38 ProPlex (Molecular Dimensions, California, USA). The ECH crystals used for the crystallographic binding studies were obtained by cocrystallization of ECH in the presence of CoA, using an optimization screen of 2.1 to 2.6 M (NH_4_)_2_SO_4_, 100 mM Tris pH 7.5, 10% octanol, 1 mM EDTA, 1 mM NaN_3_, 1 mM DTT, and using an ECH protein buffer solution, supplemented with 2 mM CoA. All crystallographic binding experiments were done at room temperature using a freshly prepared crystal soaking solution with a final concentration of 2.1 M (NH_4_)_2_SO_4_, 100 mM Tris pH 7.5 supplemented with ligand. Crystals were transferred into a 0.5 μL drop of the soaking solution supplemented with 1.8 mM (2*E*)-butenoyl-CoA­(**1**), 2.4 mM (2*E*)-hexenoyl-CoA (**1a**) and 1.8 mM (2*E*)-decenoyl-CoA (**1b**), respectively, and left to equilibrate for 24 h against the soaking solution before cryo-protection. For the crystallographic binding experiment with (2*E*)-hexenoyl-PAN (**1a**, 2 mM), and PAP (**5**, 10 mM) the crystal was transferred into a fresh drop two times before cryo-treatment. All crystals were cryo-protected by direct transfer into liquid nitrogen. The data collection was done at a temperature of 100 K using the Bruker home source or using synchrotron beamlines at ESRF, MAX IV and Diamond. The statistics of the data processing are listed in Table S3. The program COOT^34^ was used for model building in the electron density map and the structures were refined using REFMAC5 of CCP4 ^35^ and phenix.refine of Phenix.^36^ The unliganded structure was refined at 2.0 Å resolution. In the active sites of the structures obtained from the crystal soaking experiments the mode of binding of the product was captured and these structures were refined at 2.7 Å ((3*S*)-hydroxybutanoyl-CoA), 1.7 Å ((3*S*)-hydroxyhexanoyl-CoA (**2a**), 2.0 Å ((3*S)*-hydroxydecanoyl-CoA (**2b**), and 1.7 Å ((3*S*)-hydroxyhexanoyl-PAN (**6a**) with PAP (**5**)). The refinement statistics are listed in Table S3. The active site ligands were added to the model at the end of the refinement and (Fo-Fc) omit maps representative of the active site of each structure are provided in Figure S2.

### General Procedure for Preparative ECH-Catalyzed Hydration

To a stirred suspension of **4** (selected from **4a–4k**, 0.0534 mmol, ∼20–30 mg) in 40 mL of buffer (50 mM Tris, 50 mM KCl, pH= 7.4) at rt was added successively a stock solution of ECH (108 μL of 14.9 mg/mL solution in 100 mM phosphate buffer, pH 7.2, 3 mM EDTA) and 80 μL of a stock solution of **5** (50 mM, as the disodium salt in 50 mM Tris buffer, pH 7.5). After 2 h, the mixture was extracted with CHCl_3_:IPA (7:3, v/v, 5 × 80 mL). The combined extracts were dried (Na_2_SO_4_) and concentrated, and the residue was purified by CombiFlash (gradient of 3.5 to 6% MeOH/DCM) to obtain recovered **4** and the product **6**.

## Supplementary Material





## Notes

Details of protein production, activity assay, protein crystallographic methods, general synthetic methods, experimental details on the synthesis of starting material and reference samples, characterization of products, and copies of NMR spectra, as well as HPLC chromatograms (PDF)
FAIRdata NMR files (ZIP)

## Abbreviations

dr: ratio of (3*S*/3*R*) diastereomers
ECH: *Rattus norvegicus* (2*E*)-enoyl-CoA hydratase
ECI: Δ^3^,Δ^2^-enoyl-CoA isomerase
IPA: isopropyl alcohol
NAC: *N*-acetylcysteamine
PAN: pantetheine
PAP: 3′,5′-ADP
Tris: tri­(hydroxymethyl)-aminomethane
