# Supplementary material for: Enantioselective Hydration of Non-CoA Enoyl-Thioesters by Enoyl-CoA Hydratase (ECH): Activation of the Active Site Oxyanion Hole with 3′,5′-Adenosine-Diphosphate Enables Competent Catalysis
Source: JACS Au. 2026 Mar 23;6(4):2464–72. doi: 10.1021/jacsau.6c00054 (PMC13126182; doi:10.1021/jacsau.6c00054)
Supplement: Supplementary file 1 [file au6c00054_si_001.pdf]

Supporting Information

Enantioselective Hydration of non-CoA Enoyl-Thioesters by Enoyl-CoA Hydratase (ECH): Activation of the Active Site Oxyanion Hole with 3',5'- Adenosine-diphosphate Enables Competent Catalysis

Subhadra Dalwani[a,‡], Pradip Kumar Mondal[b,‡], Werner Schmitz[c], Rik K. Wierenga[a,*] and Petri M. Pihko[b,*]

‡ *These authors contributed equally.*

[a]Faculty of Biochemistry and Molecular Medicine, University of Oulu, P.O. Box 5400, FI-90014 University of Oulu, Finland.
[b]Department of Chemistry and NanoScience Center, University of Jyväskylä, P.O.Box 35, FI-40014 University of Jyväskylä , Finland.
[c]Institute of Biochemistry and Molecular Biology, University of Würzburg, Würzburg, Germany.

E-mail: rik.wierenga@oulu.fi, petri.pihko@jyu.fi

Contents

1 Enzymology and protein crystallography ... 7
1.1 Abbreviations ... 7
1.2 General experimental procedures and protocols for structural enzymology ... 7
1.2.1 Protein expression and purification ... 7
1.2.2 (2*E*)-enoyl-CoA substrates and activators ... 8
1.3 Activity assays and Michaelis-Menten kinetics ... 8
1.3.1 Spectrophotometric activity assay ... 8
1.4 Protein crystallographic studies ... 12
1.4.1 Crystallization of ECH ... 12
1.4.2 Crystallographic binding studies ... 12
1.4.3 Data collection, data processing and structure refinement ... 13
1.4.4 Ligand restraints ... 16
1.4.5 Structure analysis ... 16
1.4.6 Comparison of the oxyanion hole geometry of four ECH structures and four structures of Δ^3,Δ^2-enoyl-CoA isomerases ... 19
2 General information on the synthetic experiments ... 21
3 Experimental details for the synthetic experiments ... 22
3.1 Optimization of ECH-catalyzed hydration protocol of (2*E*)-enoyl thioesters ... 22
3.1.1 Optimization of the standard reaction conditions with alternative activators and substrates ... 22
3.1.2 Control experiments for the ECH catalyzed hydration reaction ... 23
3.2 General procedures for synthesis of starting materials ... 23
3.2.1 General procedure 1 (GP-1): Synthesis of pantetheine monomer (S1b), OJJ-03 23
3.2.2 General procedure 2 (GP-2): General procedure for the Wittig reaction ... 24
3.2.3 General procedure 3 (GP-3): General procedure for ester hydrolysis ... 24
3.2.4 General procedure 4 (GP-4): General procedure for coupling of (2*E*)-unsaturated carboxylic acids with PAN ... 25
3.3 Syntheses of starting materials for the ECH-catalyzed hydration reaction ... 25

3.3.1 Synthesis of S-(2-acetamidoethyl) (*E*)-hex-2-enethioate (**3**), OJJ-10. 25

3.3.2 Synthesis of (*R*)-*S*-(2-(3-(2,4-dihydroxy-3,3-dimethylbutanamido)propanamido)ethyl) (*E*)-hex-2-enethioate (**4a**), OJJ-04. 26

3.3.3 Synthesis of (*R*)-*S*-(2-(3-(2,4-dihydroxy-3,3-dimethylbutanamido)propanamido)ethyl) (*E*)-dec-2-enethioate (**4b**), OJJ-24. 26

3.3.4 Synthesis of (*R*)-*S*-(2-(3-(2,4-dihydroxy-3,3-dimethylbutanamido)propanamido)ethyl) (*E*)-5-methylhex-2-enethioate (**4c**), OJJ-129. 27

3.3.5 Synthesis of (*R*)-*S*-(2-(3-(2,4-dihydroxy-3,3-dimethylbutanamido)propanamido)ethyl) (*E*)-3-cyclohexylprop-2-enethioate (**4d**), OJJ-93. 29

3.3.6 Synthesis of *tert*-butyl (*R,* *E*)-4-(3-((2-(3-(2,4-dihydroxy-3,3-dimethylbutanamido)propanamido)ethyl)thio)-3-oxoprop-1-en-1-yl)piperidine-1-carboxylate (**4e**), OJJ-48. 31

3.3.7 Synthesis of (*R*)-*S*-(2-(3-(2,4-dihydroxy-3,3-dimethylbutanamido)propanamido)ethyl) (*E*)-7-bromohept-2-enethioate (**4f**), OJJ-112. 33

3.3.8 Synthesis of (*R*)-*S*-(2-(3-(2,4-dihydroxy-3,3-dimethylbutanamido)propanamido)ethyl) (*E*)-6-(1,3-dioxolan-2-yl)hex-2-enethioate (**4g**), OJJ-124. 36

3.3.9 Synthesis of methyl (*R,E*)-7-((2-(3-(2,4-dihydroxy-3,3-dimethylbutanamido)propanamido)ethyl)thio)-7-oxohept-5-enoate (**4h**), OJJ-140. 39

3.3.10 Synthesis of (*R*)-*S*-(2-(3-(2,4-dihydroxy-3,3-dimethylbutanamido)propanamido)ethyl) (*E*)-5-(benzyloxy)pent-2-enethioate (**4i**), OJJ-133. 41

3.3.11 Synthesis of (*R*)-*S*-(2-(3-(2,4-dihydroxy-3,3-dimethylbutanamido)propanamido)ethyl) (*E*)-7-((tert-butyldimethylsilyl)oxy)hept-2-enethioate (**4j**), OJJ-96. 43

3.3.12 Synthesis of (*R*)-*S*-(2-(3-(2,4-dihydroxy-3,3-dimethylbutanamido)propanamido)ethyl) (*E*)-5-phenylpent-2-enethioate (**4k**), OJJ-102. 44

3.4 ECH-catalyzed hydration reactions and characterization of products 47

3.4.1 General procedure for ECH catalyzed hydration reaction (GP-5) 47

3.4.2 Synthesis of *S*-(2-(3-((*R*)-2,4-dihydroxy-3,3-dimethylbutanamido)propanamido)ethyl) (*S*)-3-hydroxyhexanethioate (**6a**), OJJ-142. 47

3.4.3 Synthesis of *S*-(2-(3-((*R*)-2,4-dihydroxy-3,3-dimethylbutanamido)propanamido)ethyl) (*S*)-3-hydroxydecanethioate (**6b**), OJJ-143. 48

3.4.4 Synthesis of *S*-(2-(3-((*R*)-2,4-dihydroxy-3,3-dimethylbutanamido)propanamido)ethyl) (*S*)-3-hydroxy-5-methylhexanethioate (**6c**), OJJ-173. 49

3.4.5 Synthesis of *S*-(2-(3-((*R*)-2,4-dihydroxy-3,3-dimethylbutanamido)propanamido)ethyl) (*R*)-3-cyclohexyl-3-hydroxypropanethioate (**6d**), OJJ-172. 50

3.4.6 Synthesis of *tert*-butyl 4-((*R*)-3-((2-(3-((*R*)-2,4-dihydroxy-3,3-dimethylbutanamido)propanamido)ethyl)thio)-1-hydroxy-3-oxopropyl)piperidine-1-carboxylate (**6e**), OJJ-144 50

3.4.7 Synthesis of *S*-(2-(3-((*R*)-2,4-dihydroxy-3,3-dimethylbutanamido)propanamido)ethyl) (*S*)-7-bromo-3-hydroxyheptanethioate (**6f**), OJJ-146. 51

3.4.8 Synthesis of *S*-(2-(3-((*R*)-2,4-dihydroxy-3,3-dimethylbutanamido)propanamido)ethyl) (*S*)-6-(1,3-dioxolan-2-yl)-3-hydroxyhexanethioate (**6g**), OJJ-147. 52

3.4.9 Synthesis of methyl (*S*)-7-((2-(3-((*R*)-2,4-dihydroxy-3,3-dimethylbutanamido)propanamido)ethyl)thio)-5-hydroxy-7-oxoheptanoate (**6h**), OJJ-148. 53

3.4.10 Synthesis of S-(2-(3-((*R*)-2,4-dihydroxy-3,3-dimethylbutanamido)propanamido)ethyl) (*S*)-5-(benzyloxy)-3-hydroxypentanethioate (**6i**), OJJ-149. 54

3.4.11 Synthesis of S-(2-(3-((*R*)-2,4-dihydroxy-3,3-dimethylbutanamido)propanamido)ethyl) (*S*)-7-((*tert*-butyldimethylsilyl)oxy)-3-hydroxyheptanethioate (**6j**), OJJ-150. 55

3.4.12 Synthesis of S-(2-(3-((*R*)-2,4-dihydroxy-3,3-dimethylbutanamido)propanamido)ethyl) (*S*)-3-hydroxy-5-phenylpentanethioate (**6k**), OJJ-171. 56

3.5 General procedures for the syntheses of (3*RS*)-3-hydroxyacyl-PAN thioesters 57

3.5.1 General procedure 6A (GP-6A): General procedure for the synthesis of (3*RS*)-hydroxy ester (**S14a**). 57

3.5.2 General procedure 6B (GP-6B): General procedure for the synthesis of (3*RS*)-hydroxy acid (**S15a**). 58

3.5.3 General procedure 6C (GP-6C): General procedure for the synthesis of (*3RS*)-hydroxy PAN thioester (**6'**). 58

3.5.4 General procedure 7A (GP-7A): General procedure for the synthesis of (3*RS*)-hydroxy-S-phenyl thioester (**S16a**). 58

3.5.5 General procedure 7B (GP-7B): General procedure for the synthesis of (*3RS*)-hydroxy SPAN thioester (**6'**). 59

3.6 Syntheses of (3*RS*)-3-hydroxyacyl-PAN thioesters (6') 60

3.6.1 Synthesis of *S*-(2-(3-((*R*)-2,4-dihydroxy-3,3-dimethylbutanamido)propanamido)ethyl) 3-hydroxyhexanethioate (**6'a**), OJJ-108. 60

3.6.2 Synthesis of S-(2-(3-((*R*)-2,4-dihydroxy-3,3-dimethylbutanamido)propanamido)ethyl) 3-hydroxydecanethioate (**6'b**), OJJ-157. 61

3.6.3 *S*-(2-(3-((*R*)-2,4-dihydroxy-3,3-dimethylbutanamido)propanamido)ethyl) 3-hydroxy-5-methylhexanethioate (**6'c**), OJJ-194. 63

3.6.4 Synthesis S-(2-(3-((*R*)-2,4-dihydroxy-3,3-dimethylbutanamido)propanamido)ethyl) 3-cyclohexyl-3-hydroxypropanethioate (**6'd**), OJJ-198. 65

3.6.5 Synthesis of *tert*-butyl 4-(3-((2-(3-((*R*)-2,4-dihydroxy-3,3-dimethylbutanamido)propanamido)ethyl)thio)-1-hydroxy-3-oxopropyl)piperidine-1-carboxylate (**6'e**), OJJ-99 67

3.6.6 Synthesis of S-(2-(3-((*R*)-2,4-dihydroxy-3,3-dimethylbutanamido)propanamido)ethyl) 7-bromo-3-hydroxyheptanethioate (**6'f**), OJJ-212. 69

3.6.7 Synthesis of S-(2-(3-((*R*)-2,4-dihydroxy-3,3-dimethylbutanamido)propanamido)ethyl) 6-(1,3-dioxolan-2-yl)-3-hydroxyhexanethioate (**6'g**), OJJ-195 73

3.6.8 Synthesis of methyl 7-((2-(3-((*R*)-2,4-dihydroxy-3,3-dimethylbutanamido)propanamido)ethyl)thio)-5-hydroxy-7-oxoheptanoate (**6'h**), OJJ-208. 79

3.6.9 Synthesis of S-(2-(3-((*R*)-2,4-dihydroxy-3,3-dimethylbutanamido)propanamido)ethyl) 5-(benzyloxy)-3-hydroxypentanethioate (**6'i**), OJJ-197 80

3.6.10 Synthesis of S-(2-(3-((*R*)-2,4-dihydroxy-3,3-dimethylbutanamido)propanamido)ethyl) 7-((*tert*-butyldimethylsilyl)oxy)-3-hydroxyheptanethioate (**6'j**), OJJ-211. 82

3.6.11 Synthesis of S-(2-(3-((*R*)-2,4-dihydroxy-3,3-dimethylbutanamido)propanamido)ethyl) 3-hydroxy-5-phenylpentanethioate (**6'k**), OJJ-158. 84

4 References 87

5 Copies of NMR spectra 90

6 Copies of HPLC chromatograms 229

1 Enzymology and protein crystallography

1.1 Abbreviations

ECH: (2*E*)-enoyl-CoA hydratase
ECI: Δ^3,Δ^2-enoyl-CoA isomerase
BME: β-mercaptoethanol
IPTG: isopropyl β-D-1-thiogalactopyranoside
MES: 2-(N-morpholino)-ethanesulfonic acid
MPD: 2-methyl-2,4-pentanediol
PAP: 3',5'-adenosine-diphosphate
SEC: size exclusion chromatography
Tris: tri(hydroxymethyl)-aminomethane

1.2 General experimental procedures and protocols for structural enzymology

1.2.1 Protein expression and purification

Recombinant rat mitochondrial ECH was produced in *Escherichia coli* BL21 (DE3) pLysS cells, transformed by a previously constructed pET3a expression vector[1]. This construct concerns the mature form of rat mitochondrial ECH (UNIPROT ID P14604), starting with a methionine before Gly30 of the mature form (the 29 amino acids of the mitochondrial targeting sequence were omitted). BL21 (DE3) pLysS cells, containing the expression vector, were first streaked onto a LB agar plate containing ampicillin (100 μg/mL) and chloramphenicol (34 μg/mL) and incubated at 37 °C overnight. A single colony was then harvested and used to start 20 mL of primary culture in LB medium in the 37 °C incubator. 10 mL of primary culture was used to inoculate 1 liter of M9Z medium containing ampicillin and chloramphenicol at 37 °C. The expression was induced with 0.4 mM IPTG when the culture reached an OD_{600} of 0.6, for further growth at 35 ^{o}C for 3 hours. The cells were then harvested by centrifugation at 6000 rpm for 20 minutes, using the Sorvall Lynx 6000 centrifuge (Thermo Fisher Scientific, Waltham, MA, USA) with the F12 6x500 LEX fixed-angle rotor. The harvested cells were resuspended in 50 mL of lysis buffer (30 mM potassium phosphate, pH 7.2, 3 mM EDTA, 0.5 mM β-mercaptoethanol (BME), 200 μg/mL lysozyme, 20 μg/mL DNAse, 20 μg/mL RNAse) and incubated at room temperature for 30 minutes with stirring. The supernatant was then harvested by centrifugation at 16000 rpm for 1 hour (using the Sorvall Lynx 6000 centrifuge and the Fiberlite F20-12 x 50 LEX Fixed Angle Rotor) and loaded onto a pre-

equilibrated 30 mL DEAE Sephacel anion exchange column (GE Healthcare, Chicago, USA) (30 mM potassium phosphate, pH 7.2, 10 mM $MgSO_4$, 0.5 mM BME, 3 mM EDTA). 2 mL fractions were collected. The fractions were checked by SDS-PAGE analysis and the peak fractions were pooled and dialyzed in the cold room, at 5–10 °C, overnight, against 2 liter of dialysis buffer (25 mM Tris, pH 8.5, 0.5 mM BME, 1 mM EDTA). The dialyzed protein solution was then injected onto a Resource Q anion exchange column, pre-equilibrated with 25 mM Tris, pH 8.5, 0.5 mM BME, 1 mM EDTA and eluted between 0.0 M and 0.25 M NaCl in a 25 mL elution volume. Fractions containing ECH were pooled and concentrated to 2 mL using a 10 KDa Centricon concentrator (Merck, Darmstadt, Germany). The concentrated protein solution was then injected onto a S200 SEC column pre-equilibrated with SEC buffer (100 mM potassium phosphate, pH 7.2, 3 mM EDTA). Peak fractions of the protein were collected and concentrated to a final concentration of 15 mg/mL, flash frozen in liquid nitrogen and stored at –70 °C for further use.

1.2.2 (2*E*)-enoyl-CoA substrates and activators

(2*E*)-butenoyl-CoA, 3',5'-adenosine-diphosphate, CoA, ATP, ADP and AMP were purchased from Sigma-Aldrich (Merck, Darmstadt, Germany). (2*E*)-hexenoic acid and (2*E*)-decenoic acid and other chemicals used for the synthesis of (2*E*)-hexenoyl-CoA and (2*E*)-decenoyl-CoA, were also purchased from Merck (Darmstadt, Germany). The coupling of these two carboxylic acids to access their corresponding CoA esters was done by the mixed anhydride method, following our previously published protocol.[2] The identity of the (2*E*)-enoyl-CoA esters was confirmed by high resolution mass spectrometry analysis.

1.3 Activity assays and Michaelis-Menten kinetics

1.3.1 Spectrophotometric activity assay

Enzyme activity was measured with the Jasco V660 spectrophotometer (Jasco, Tokyo, Japan) using a previously reported spectrophotometric direct assay.[3] All assays were performed at 25 °C using 50 mM Tris, pH 7.5 and 50 mM KCl as reaction buffer with a final volume of 500 µL in quartz cuvettes, with each reaction being monitored for 3 minutes. The reaction was initiated by mixing enzyme into the reaction buffer. The concentration of the substrates was verified by the Ellman's test.[4,5] For the assay of (2*E*)-enoyl-CoA, 2 ng of enzyme was added into the cuvette and the substrate concentration varied from 3 µM to 75 µM. For the assays with the (2*E*)-hexenoyl-PAN substrate 5 µg of enzyme was added into the cuvette and the substrate concentration varied from 25 µM to 500 µM (2*E*)-hexenoyl-PAN. The latter assay was done without activator or in the presence of 100 µM activator, being either CoA, PAP, ADP, ATP, or AMP. The activity

was monitored by measuring the disappearance of the C-C double bond of the substrate at 263 nm. The JASCO software Spectra Manager™ was used for carrying out the experiments and for the subsequent rate calculations. Initial reaction rates were determined using the linear part of the progress curve, and using an absorption coefficient of 6700 $M^{-1}cm^{-1}$. GraphPad Prism version 10 (GraphPad, Boston, USA) was used to analyse and plot the data (**Figure S1)**. The k_{cat} and K_M values (**Table S1**) are the averages of three independent measurements.

Table S1. The Michaelis-Menten kinetic constants for the (2*E*)-enoyl-CoA hydratase reaction catalyzed by rat mitochondrial ECH. The listed Michaelis-Menten constants and error bars were calculated as the averages and their standard deviations, respectively, from the corresponding values of three independent data sets.

substrate	(2*E*)-butenoyl-CoA	(2*E*)-hexenoyl-CoA	(2*E*)-decenoyl-CoA
k_{cat} (s^{-1})	2231.0 ± 25.2	1101.3 ±80.0	285.9 ±13.3
K_M (µM)	24.2 ± 1.8	23.3 ± 3.6	2.7 ± 0.2
k_{cat}/K_M ($M^{-1}s^{-1}$)	92.6 ± 6.1 10^6	47.9 ± 6.1 10^6	106.2 ± 3.3 10^6

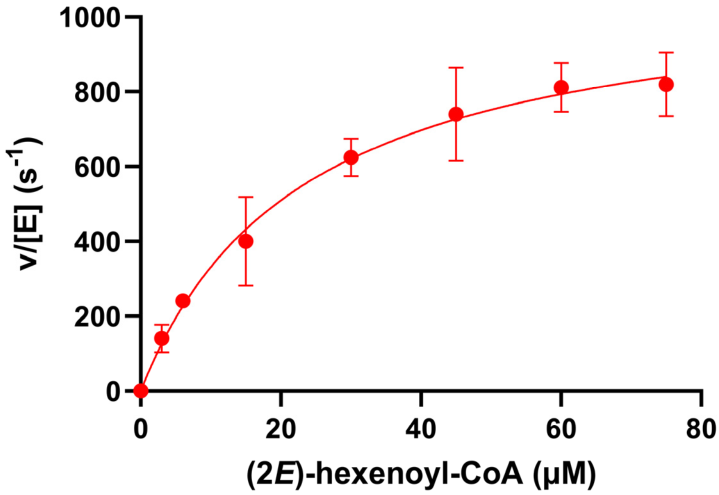

Figure S1. The Michaelis-Menten plot for the hydration by ECH of the substrate (2*E*)-hexenoyl-CoA. On the y-axis are plotted the initial rates, plotted as v/[E]. The error bars visualize the range of the plotted rates as obtained from three different data sets.

Table S2. The k_{cat}/K_M values for three ECH catalyzed reactions. The values are obtained from **Table S1** (for the substrate (2*E*)-hexenoyl-CoA) and from the data used for **Figure 1 (**for the substrate (2*E*)-hexenoyl-PAN (no activator) and for the substrate (2*E*)-hexenoyl-PAN (in the presence of activator, 100 μM PAP)).

**substrate**	$\mathbf{k_{cat}/K_M}$ **($M^{-1}s^{-1}$)**
(2*E*)-hexenoyl-CoA	$48 \quad 10^6$
(2*E*)-hexenoyl-PAN (no activator)	54
(2*E*)-hexenoyl-PAN (in the presence of activator, 100 μM PAP)	780

The free energy difference between the transition state barriers for the ECH catalyzed hydration reaction with (2*E*)-hexenoyl-PAN (no activator) and (2*E*)-hexenoyl-CoA as substrates ($\Delta\Delta G^{\ddagger}_{CoA}$) was calculated from the differences of the respective k_{cat}/K_M values:[6]

$$\Delta\Delta G^{\ddagger}_{CoA} = -RT \ln \left[\frac{\left(\frac{k_{\mathrm{cat}}}{K_{\mathrm{M}}}\right)((2E)\mathrm{-hexenoyl-PAN,\ \ no\ activator})}{\left(\frac{k_{\mathrm{cat}}}{K_{\mathrm{M}}}\right)((2E)\mathrm{-hexenoyl-CoA})} \right] = 8.1\ \mathrm{kcal\ mol^{-1}}$$

The free energy difference of the transition state barriers for the ECH catalyzed hydration reaction (2*E*)-hexenoyl-PAN (no activator) and (2*E*)-hexenoyl-PAN (in the presence of 100 μM PAP activator) ($\Delta\Delta G^{\ddagger}_{PAP}$) is calculated as follows:

$$\Delta\Delta G^{\ddagger}_{PAP} = -RT \ln \left[\frac{\left(\frac{k_{\mathrm{cat}}}{K_{\mathrm{M}}}\right)((2E)\mathrm{-hexenoyl-PAN,\ \ no\ activator})}{\left(\frac{k_{\mathrm{cat}}}{K_{\mathrm{M}}}\right)((2E)\mathrm{-hexenoyl-PAN\ with\ activator})} \right] = 1.6\ \mathrm{kcal\ mol^{-1}}$$

1.4 Protein crystallographic studies

1.4.1 Crystallization of ECH

The crystals of unliganded ECH were obtained at room temperature in an optimization screen designed around the condition H08 (0.05 M calcium acetate, 0.1 M sodium cacodylate, pH 6.0, 25% v/v MPD) of the commercial screen MD1-38 ProPlex (Molecular Dimensions, California, USA) and using a 10 mg/mL ECH stock solution in SEC buffer.

The ECH crystals used for the crystallographic binding studies were obtained by co-crystallization of ECH at room temperature in the presence of CoA, using an optimization screen of 2.1 to 2.6 M $(NH_4)_2SO_4$, 100 mM Tris pH 7.5, 10% octanol, 1 mM EDTA, 1 mM NaN_3, 1 mM DTT, and using a 10 mg/mL ECH stock solution in SEC buffer, supplemented with 2 mM CoA, being allowed to incubate at room temperature for half hour prior to setting up the crystallization experiment.

For both crystal forms, the crystallization drops were set up in 1:1 (400 nL : 400 nL) drop size of protein buffer : well solution in TTP sitting drop plates using a Mosquito nanodispensing robot (TTP Labtech, Melbourn, UK). The plates were imaged using the RI54 Rock imager (Formulatrix, Dubai, UAE) at regular intervals and the formation of the crystals was monitored using the IceBear software[7]. Crystals appeared approximately 3 days after setting up the crystallization drops and were harvested between 2-4 months later.

1.4.2 Crystallographic binding studies

For all crystal soaking experiments, fresh crystal soaking solutions were prepared having a final concentration of 2.1 M $(NH_4)_2SO_4$, 100 mM Tris, pH 7.5 supplemented with ligand. All crystal soaking experiments were done at room temperature, using the crystals obtained by co-crystallization with CoA.

The crystals of the ECH-CoA complex were transferred into a drop of 0.5 µL of the soaking solution and left to equilibrate against the soaking solution before the crystals were harvested. For the soaking experiments with (2*E*)-butenoyl-CoA (final concentration of 1.8 mM), (2*E*)-hexenoyl-CoA (final concentration of 2.4 mM) and (2*E*)-decenoyl-CoA (final concentration of 1.8 mM), crystals were equilibrated in one drop of the respective soaking solution for 24 hours before cryo-protection. For the soaking experiment in the presence of (2*E*)-hexenoyl-PAN and PAP, the crystal was transferred consecutively into 3 separate 0.5 µL drops of soaking solution (each supplemented with 2 mM (2*E*)-hexenoyl-PAN, and 10 mM PAP) and left to equilibrate for 3 hours, 48 hours and 48 hours against the soaking solution in, respectively, the 1st, 2nd and 3rd drop before cryo-protection.

1.4.3 Data collection, data processing and structure refinement

All crystals were cryo-protected by direct transfer into liquid nitrogen and the data collection was done at a temperature of 100 K. For data collection, either the home source Microstar, equipped with the PHOTON II detector (Bruker, Karlsruhe, Germany) was used or beamlines at various European synchrotrons, being DLS, MAX IV and ESRF (**Table S3**) were used. Data processing was either performed using PROTEUM3 (Bruker) or by using data processing pipelines available at the different synchrotron facilities, being xia2-3dii[8] autoPROC[9] and EDNAproc[10] (**Table S3).** STARANISO[11] and AIMLESS[12] were used for merging the data (**Table S3**). Initial phases were calculated after molecular replacement calculations by PHASER[13] or MOLREP[14], using coordinates derived from PDB ID 1DUB (after removing the bound ligands, solutes and waters) as the search model. The positioned coordinates, obtained from the molecular replacement calculations, were used as the initial model to perform iterative rounds of model building and refinement employing, respectively, COOT[15], and phenix.refine of Phenix[16,17] or REFMAC5 of CCP4[18–20]. The active site ligands were only built in their electron density after several rounds of manual model building and refinement and after adding waters. Structure quality was assessed using MolProbity[21], as well as by inspecting the validation report from the PDB validation server[22,23]. The final refinement statistics are listed in **Table S3**. Representative (Fo-Fc) omit maps are shown in **Figure S2**.

Table S3. Data collection setup, data processing statistics and refinement statistics

Complex	unliganded	3*S*-hydroxybutanoyl-CoA	3*S*-hydroxyhexanoyl-CoA	3*S*-hydroxydecanoyl-CoA	3*S*-hydroxyhexanoyl-PAN, 3',5'-adenosine-diphosphate
**Data collection**					
Beam line	DLS, I24	Bruker, Microstar X8	ESRF, ID30B	Bruker, Microstar X8	MAX IV, BioMAX
Detector	Pilatus3 6M	Photon II	EIGER2 X 9M	Photon II	Eiger2 16M
Wavelength (Å)	0.6199	1.5417	0.9686	1.5417	0.7290
Temperature (K)	100	100	100	100	100
**Data processing**					
Space group	H3	$P2_12_12_1$	$P2_12_12_1$	$P2_12_12_1$	$P2_12_12_1$
Unit cell parameters					
a, b, c (Å)	77.40 77.40 217.29	76.11 93.69 247.47	76.08 93.69 248.68	76.79 94.48 248.97	75.83 93.00 247.47
α,β, γ (^{O})	90.0 90.0 120.0	90.0 90.0 90.0	90.0 90.0 90.0	90.0 90.0 90.0	90.0 90.0 90.0
Processing software	xia2-3dii, AIMLESS	PROTEUM3, AIMLESS	autoPROC,STARANISO	PROTEUM3, AIMLESS	EDNAproc, AIMLESS
Resolution range (Å)[a]	38.70-2.00(2.05-2.00)	72.75-2.69(2.78-2.69)	124.70-1.68(1.89-1.68)	73.38-2.0(2.03-2.00)	47.98-1.70(1.73-1.70)
R_{pim} (%) (all I+/I-)	2.1(37.9)	9.6(34.5)	6.3(32.9)	4.0(76.4)	3.7(72.0)
CC1/2	99.0(86.0)	97.9(76.9)	98.9(79.0)	98.7(47.8)	99.9(34.0)
I/s(I)	16.3(2.0)	7.6(2.1)	6.1(1.6)	11(0.8)	14(1.2)
Completeness, ellipsoidal (%)			93.6(71.0)		
Completeness, spherical (%)	100.0(100.0)	99.8(98.4)	55.8(9.3)	97.0(78.7)	100(100)
Multiplicity	10.8(10.8)	8.6(7.1)	4.0(5.2)	9.7(2.5)	13.6(13.6)
Number of measured reflections	355036 (26562)	429104 (31263)	454400 (29407)	1169581 (11761)	2617210 (129035)
Number of unique reflections	32842(2457)	49784(4419)	113963(5698)	120103(4754)	192681(9478)
Wilson B-factor ($Å^2$)[b]	48.1	23.5	23.8	14.9	22.4
Number of subunits in asymmetric unit.	2	6	6	6	6
**Refinement**					
Resolution range (Å)	36.46 - 2.00	72.75 - 2.69	124.70 - 1.68	73.38 - 2.00	47.98 - 1.70
Number of used reflections	32824	49710	113963	119681	192664
R_{work} (%)	19.8	20.1	20.9	23.0	21.7
R_{free} (%)	24.4	25.1	24.2	25.8	24.8
Total number of atoms	4091	11980	12851	12549	12437
Number of waters	111	71	899	574	490
**Average B-factor**					
Protein ($Å^2$)	58.8	27.6	29.4	19.8	35.3
Active site ligands ($Å^2$)[c]		20.1 (3HC)	20.5 (H6C)	14.9 (HSC)	50.0 (HPA)<br>53.1 (A3P)
**Rms deviations**					
Rms bond length (Å)	0.002	0.002	0.002	0.003	0.003
Rms bond angle (^{O})	0.4	0.5	0.8	0.9	0.9
**Ramachandran plot** (%)[d]					
Favored	97.6	97.3	98.6	98.1	98.5
Allowed	1.4	2.7	1.4	1.9	1.5
Outliers	1.0	0.00	0.00	0.00	0.0
**PDB ID**	**9RGQ**	**9RGR**	**9RGS**	**9RGT**	**9RGU**

[a] The numbers in parentheses refer to the highest resolution shell.

[b] As provided by the PDB validation report.

[c] These are the average B-factors of the active site ligands bound to the reference subunit, which is subunit E for the (3*S*)-hydroxyacyl-CoA structures and subunit F for the (3*S*)-hydroxyhexanoyl-PAN+PAP structure. 3HC: (3*S*)-hydroxybutanoyl-CoA; H6C: (3*S*)-hydroxyhexanoyl-CoA, HSC: (3*S*)-hydroxydecanoyl-CoA, HPA: (3*S*)-hydroxyhexanoyl-PAN, A3P: 3'5'-adenosine-diphosphate.

[d] As calculated by MolProbity[21].

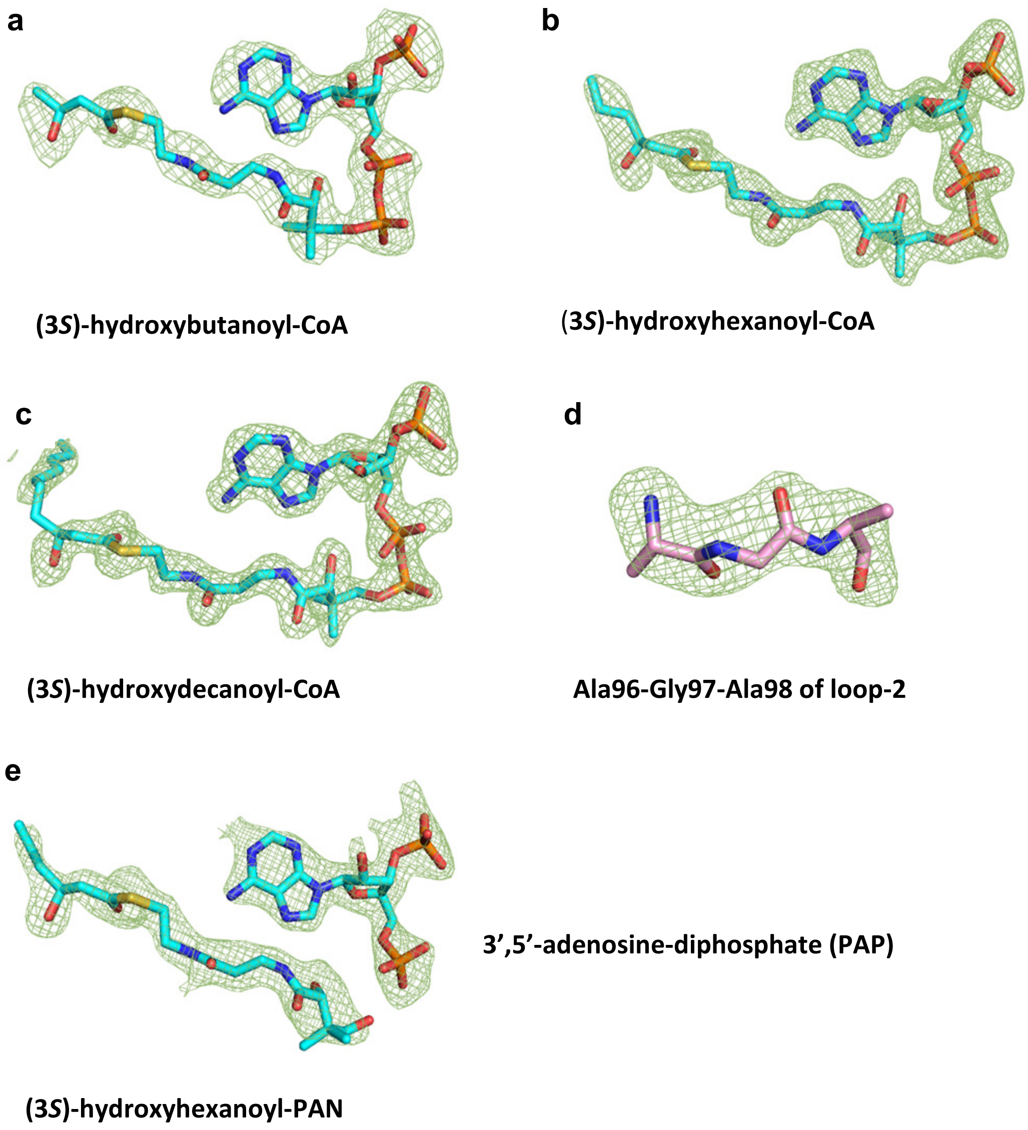

Figure S2. Omit (Fo–Fc) electron density maps, calculated after omit refinement, excluding the shown molecules. (a) The fit to the electron density map for (3*S*)-hydroxybutanoyl-CoA (subunit E, PDB ID 9RGR; the contour level is 2.5 sigma). (b) The fit to the electron density map for (3*S*)-hydroxyhexanoyl-CoA (subunit E, PDB ID 9RGS, the contour level is 3.0 sigma). (c) The fit to the electron density map for (3*S*)-hydroxydecanoyl-CoA (subunit E, PDB ID 9RGT, the contour level is 2.5 sigma). (d) The fit to the electron density map for the residues Ala96-Gly97-Ala98 of loop-2 of the unliganded structure (subunit A, PDB ID 9RGQ, the contour level is 3.0 sigma). (e) The fit to the electron density map for (3*S*)-hydroxyhexanoyl-PAN and 3′,5′-adenosine-diphosphate (subunit F, PDB ID 9RGU, the contour level is 2.5 sigma).

1.4.4 Ligand restraints

Restraints library files were made for four ligands, which are (3*S*)-hydroxybutanoyl-CoA, (3*S*)-hydroxyhexanoyl-CoA, PAP and (3*S*)-hydroxyhexanoyl-PAN. MarvinSketch (Chemaxon, Budapest, Hungary) was used to draw and generate a mol2 representation of each ligand. The resulting mol2 file was then uploaded to the GRADE2 webserver[24] to generate the restraints file for each of these four ligands for the refinement calculations.

1.4.5 Structure analysis

In the unliganded crystal form there are two subunits per asymmetric unit. There are no structural differences between these two subunits and subunit A is used as the reference structure. A phosphate ion is bound in the catalytic site, replacing the catalytic water.

In the crystals obtained by co-crystallization in the presence of CoA there are six subunits in the asymmetric unit. The CoA molecule bound to the active site of subunits A and E is involved in crystal contacts. The active site of subunits C and D is also near crystal contacts. The active site of subunit D is always unliganded in this crystal form[25] (**Table S4**) and a water is bound between the side chains of the two catalytic glutamates. The crystallographic binding studies have been done with (2*E*)-butenoyl-CoA, (2*E*)-hexenoyl-CoA, (2*E*)-decenoyl-CoA and (2*E*)-hexenoyl-PAN (in the presence of PAP). The latter compounds are substrates, being hydrated when bound in the active site. The active site of subunit E is used as the reference structure for describing the mode of binding of the (3*S*)-hydroxybutanoyl-CoA, (3*S*)-hydroxyhexanoyl-CoA and (3*S*)-hydroxydecanoyl-CoA and subunit F is used for describing the mode of binding of (3*S*)-hydroxyhexanoyl-PAN and 3',5'-adenosine-diphosphate. The structures were compared by superimposing the respective subunits, using the SSM protocol[26], as implemented in COOT. Two superpositions of the active site structures of unliganded ECH and of ECH complexed with (2*E*)-hexenoyl-CoA are shown in **Figure S3** and **Figure S4.** The structures are visualized with PyMOL (Schrödinger LLC, New York, NY, USA).

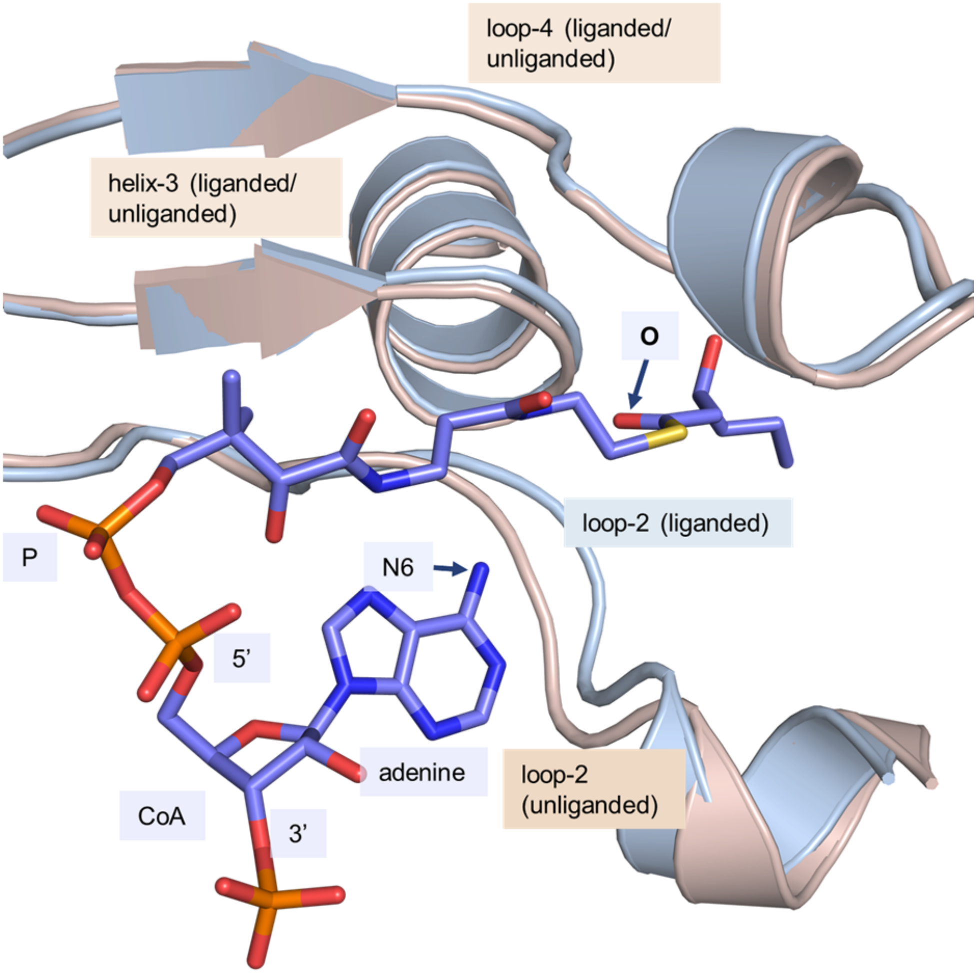

Figure S3. Superposition of the active site region of the unliganded structure (PDB ID 9RGQ, pink) and the liganded structure (PDB ID 9RGS, light-blue), visualizing the conformational change of loop-2 on binding of the PAP part of CoA. Same view as **Figure 2e**, zoomed out. The bound (3*S*)-hydroxyhexanoyl-CoA is shown in dark-blue color. The thioester oxygen atom of the product is indicated by an arrow. N6 identfies the 6-amino group of the adenine moiety. The cartoon representation of the protein part includes loop-2 (with Ala98, hydrogen bond donor of the oxyanion hole), helix-3 (with Gly141, hydrogen bond donor of the oxyanion hole and with Glu144, catalytic glutamate), and loop-4 (with Glu164, catalytic glutamate).

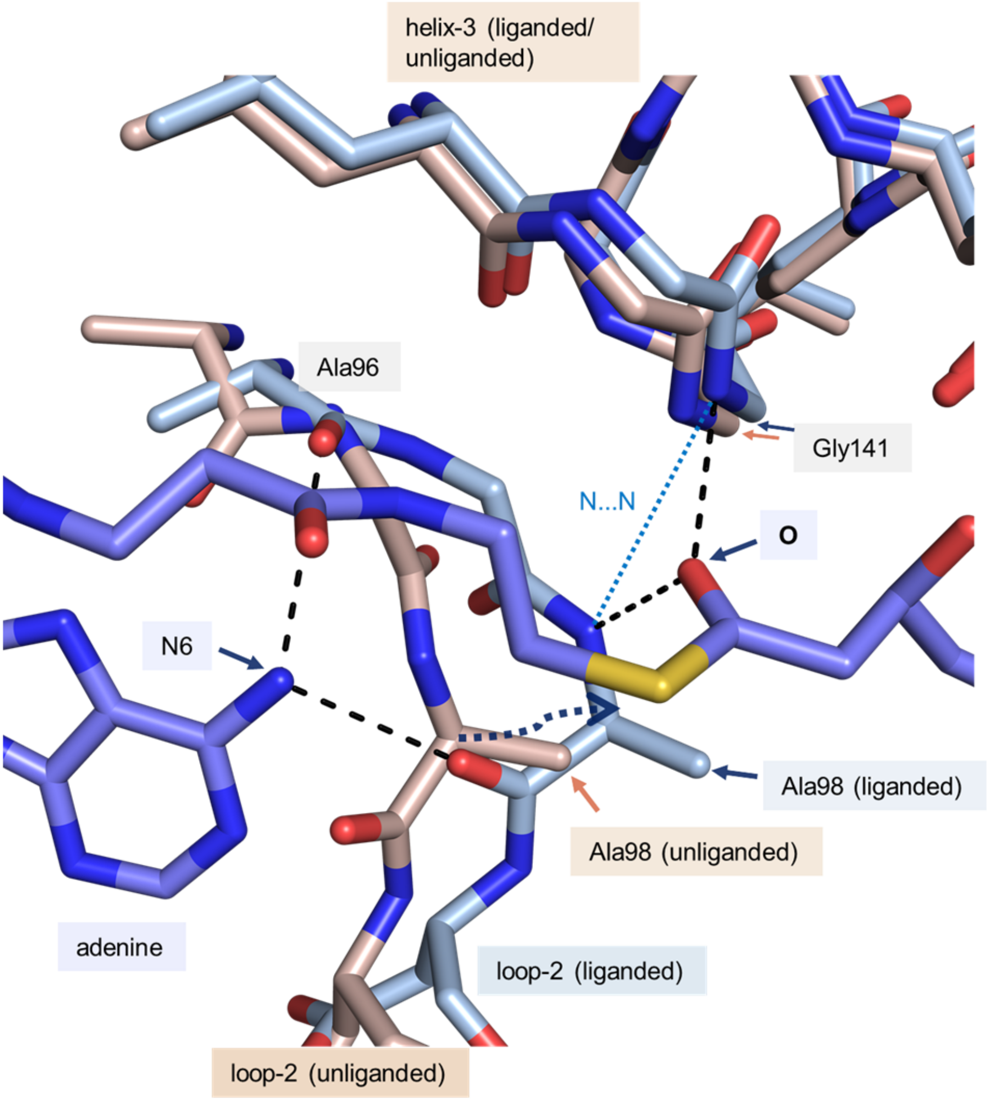

Figure S4. Superposition of the active site region of the unliganded structure (PDB ID 9RGQ, pink) and the liganded structure (PDB ID 9RGS, light-blue), visualising the rotation of the Gly97-Ala98 peptide unit of loop-2 on binding of the PAP part of CoA. Same view as **Figure 2e**, zoomed in**.** The bound (3*S*)-hydroxyhexanoyl-CoA is shown in dark-blue color. The thioester oxygen atom of the bound (3*S*)-hydroxyhexanoyl-CoA is indicated by an arrow. The dotted arrow shows the movement of Ala98 of loop-2 by about 2Å upon ligand binding, tightening the oxyanion hole and shortening the N…N distance. N6 identifies the 6-amino group of the adenine moiety. The black dotted lines show the hydrogen bonds between the amino group of the adenine moiety of CoA and the main chain oxygen atoms of Ala96 and Ala98 of loop-2, as well as between the thioester oxygen atom of the ligand and the peptide NH moieties of Ala98 (loop-2) and Gly141 (helix-3).

1.4.6 Comparison of the oxyanion hole geometry of four ECH structures and four structures of Δ^3,Δ^2-enoyl-CoA isomerases

The incompetent conformation of the oxyanion hole of a (2*E*)-enoyl-CoA hydratase was first described for the hydratase active site of the crystal structure of the trifunctional enzyme of *Mycobacterium tuberculosis* and it was also found in structures of some other (2*E*)-enoyl-CoA hydratases having the crotonase fold[27]. A further comparison of the oxyanion hole geometry of structures of rat mitochondrial ECH is listed in **Table S4**, which includes information on the oxyanion hole geometry of Δ^3,Δ^2-enoyl-CoA isomerases (ECIs). The enzymes of the ECI subfamily are sequence related to the ECH subfamily of enzymes and both adopt the crotonase fold[28]. It can be noted that the conformational switch of loop-2 on binding the adenine moiety of CoA is only seen in the structures of the ECH active sites (**Table S4**). In the ECI active sites loop-2 is always in the competent conformation, but the NN-distance is larger than in the competent ECH active sites.

Table S4. The NN distance (in Å) of four ECH structures and four ECI structures. The NN-distance is the distance between the two peptide NH hydrogen bond donors of the oxyanion hole of the ECH and ECI active sites[a]

enzyme	ECH[b]	ECH[b]	ECH[b]	ECH[b]	ECI[c]	ECI[c]	ECI[d]	ECI[d]
source	rat mito chondrial	rat mito chondrial	rat mito chondrial	rat mito chondrial	human mito chon drial	rat mito chon drial	yeast peroxiso mal	yeast peroxiso mal
resolution	2.1 Å	1.7 Å	1.7 Å	2.0 Å	1.3 Å	2.2 Å	2.1 Å	2.1 Å
PDB ID	1MJ3[25]	9RGS (these studies)	9RGU (these studies)	9RGQ (these studies)	1SG4[29]	1XX4[30]	4ZDC[31]	1PJH[32]
number of subunits in the asymmetric unit	6	6	6	2	3	1	3	3
NN distance (subunit A)	3.9 (in)	4.0 (in)	3.9 (in)	5.1 (out)	4.9	4.5	5.2	5.2
NN distance (subunit B)	3.9 (in)	4.0 (in)	3.9 (in)	5.2 (out)	4.2	NA	5.2	5.2
NN distance (subunit C)	4.0 (in)	4.0 (in)	5.2(out)	NA	4.4	NA	5.2	5.1
NN distance (subunit D)	4.9 (out)	5.2 (out)	5.1(out)	NA	NA	NA	NA	NA
NN distanc (subunit E)	4.0 (in)	4.0 (in)	3.9 (in)	NA	NA	NA	NA	NA
NN distance (subunit F)	4.0 (in)	3.9 (in)	4.0 (in)	NA	NA	NA	NA	NA

[a] The yellow color highlights active sites that are liganded with the ADP part of CoA or PAP. The grey color highlights the subunits of which the active sites are unliganded. The ECH active sites which have the competent oxyanion hole geometry are identified as (in) and those with the incompetent conformation are identified as (out). NA: not applicable.

[b] The crystal packing of the structures 1MJ3 (ECH complexed with (2*E*,4*E*)-hexadienoyl-CoA), 9RGS (ECH complexed with hydrated (2*E*)-hexenoyl-CoA (these studies) and 9RGU (ECH complexed with hydrated (2*E*)-hexenoyl-PAN+PAP (these studies) is the same and crystal packing causes that the active sites of the subunits of the grey colored boxes (in particular subunit D, but also subunit C) are unliganded. In these three structures there is a hexamer in the asymmetric unit. The structure 9RGQ (ECH, unliganded, these studies) is from a different crystal form, in which there are two subunits per asymmetric unit.

[c] In PDB entries 1SG4 (human, mitochondrial ECI) and 1XX4 (rat, mitochondrial ECI) the catalytic glutamate corresponds to Glu164 of rat mitochondrial ECH.

[d] In PDB entries 4ZDC (yeast, peroxisomal ECI) and 1PJH (yeast, peroxisomal ECI) the catalytic glutamate protrudes into the active site from another loop region (also referred to as the flap region) and this catalytic glutamate corresponds to residue Gly172 of rat mitochondrial ECH.

2 General information on the synthetic experiments

All reactions were carried out under inert atmosphere in flame-dried glassware, unless otherwise noted. THF, Et_2O and DCM were obtained by passing deoxygenated solvents through activated alumina columns (MBraun SPS-800 Series solvent purification system). Other solvents and reagents were used as obtained from supplier, unless otherwise noted. The reactions were cooled with ice-water bath (nominally 0 °C, external temperature), or with acetone-liquid nitrogen mixture (external bath temperature –78 °C). The heating reactions were carried out using an oil bath.

Analytical TLC was performed using Merck silica gel F254 (230-400 mesh) plates and analyzed by UV light or by staining upon heating with $KMnO_4$ solution (1.0 g $KMnO_4$, 6.7 g K_2CO_3, 1.7 mL 1 M NaOH, 100 mL H_2O) or PMA solution (20.0 g PMA dissolved in 100 mL absolute ethanol). For silica gel chromatography, the flash chromatography technique with Merck silica gel 60 (230-400 mesh) and CombiFlash Rf 200 with RediSep Gold or Silver columns (20–40 µm spherical silica, 400–632 mesh) were used with p.a. grade solvents unless otherwise noted.

The ^{1}H NMR and $^{13}C\{^{1}H\}$ NMR spectra were recorded in either $CDCl_3$, or D_4-MeOH on Bruker Avance 500 MHz, 400 MHz and 300 MHz NMR spectrometers. The chemical shifts are reported in ppm relative to $CHCl_3$ (δ =7.26), D_4-MeOH (δ =3.31) for ^{1}H NMR. For the $^{13}C\{^{1}H\}$ NMR spectra, the residual $CDCl_3$ (δ=77.16), D_4-MeOH (δ =49.0) were used as the internal standards. IR spectra were recorded on a Bruker FT-IR spectrometer. High resolution mass spectrometric (HRMS) data were measured using Agilent 6530 UHPLC-QTOF mass spectrometer. Optical rotations were measured using Perkin Elmer model 341 polarimeter. The diastereomeric (dr) or enantiomeric ratios (er) of the hydration products were determined using Waters 600 series analytical HPLC using the appropriate chiral columns as specified below, in comparison to the corresponding reference samples (3R/3S mixtures).

3 Experimental details for the synthetic experiments

3.1 Optimization of ECH-catalyzed hydration protocol of (2*E*)-enoyl thioesters

3.1.1 Optimization of the standard reaction conditions with alternative activators and substrates

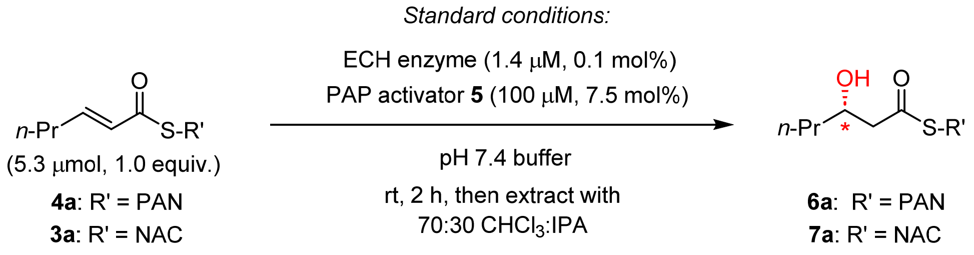

Entry	Substrate	Scale	ECH stock solution	Activator	Buffer quantity	Time	Solvent for extraction	**4a**:**6a** or **3a**:**7a**[b]	Conversion to **6a** or **7a** (%)[c]
1.	**4a**	2.0 mg (1.3 mM)	10.8 µL (1.4 µM)	**5** (200 µM)	4 mL	1h	$CHCl_3$	36: 64	64.0
2.	**4a**	2.0 mg (1.3 mM)	10.8 µL (1.4 µM)	**5** (200 µM)	4 mL	2h	$CHCl_3$	31: 64	64.0
3.	**4a**	2.6 mg (2.6 mM)	10.8 µL (2.8 µM)	**5** (400 µM)	2 mL	2h	$CHCl_3$	28: 70	70.0
4.	**4a**	2.0 mg (1.3 mM)	10.8 µL (1.4 µM)	**5** (100 µM)	4 mL	2h	$CHCl_3$:IPA (7:3)	28: 72	72.0 (64)[d]
5.	**4a**	2.0 mg (2.6 mM)	10.8 µL (2.8 µM)	**5** (50 µM)	2 mL	2h	$CHCl_3$:IPA (7:3)	25: 65	65.0
6.	**4a**	2.0 mg (1.3 mM)	0.0 µL	**5** (100 µM)	4 mL	2h	$CHCl_3$:IPA (7:3)	~ 100:0	0.0
7.	**4a**	2.0 mg (1.3 mM)	10.8 µL (1.4 µM)	0.0 µL	4 mL	2h	$CHCl_3$:IPA (7:3)	~ 73: 27	27.0
8.	**4a**	2.0 mg (1.3 mM)	10.8 µL (1.4 µM)	**8** (1.33 mM)	4 mL	2h	$CHCl_3$:IPA (7:3)	~ 25: 70	70.0
9.	**3a**	1.0 mg (1.3 mM)	9.4 µL (1.4 µM)	**5** (200 µM)	3.5 mL	4h	DCM	~ 100: ~0	0.0
10.	**3a**	1.0 mg (1.3 mM)	9.4 µL (1.4 µM)	**5** (200 µM)	3.5 mL	4h	$CHCl_3$	> 90: < 10	10.0
11.	**3a**	1.0 mg (1.3 mM)	9.4 µL (1.4 µM)	**5** (200 µM)	3.5 mL	16h	$CHCl_3$	> 93: < 7	7.0

[a]Standard reaction conditions: 1.3 mM of **4a** (2.0 mg, 5.3 µmol) or **3a** (1.0 mg, 5.3 µmol), 1.4 µM ECH (0.1 mol%), 100 µM of **5** (7.5 mol%) in 4 mL of 50 mM 2-amino-2-(hydroxymethyl)propane-1,3-diol (tris) buffer (pH 7.4) / 50 mM KCl, rt, 2 h. [b,c] Ratios of

4a:**6a** (**3a**:**7a**) and conversions were determined by ^{1}H NMR, using 1,3,5-trimethoxybenzene as an internal standard. [d]Isolated yield (at 53 µmol scale) of **6a**.

3.1.2 Control experiments for the ECH catalyzed hydration reaction

3.1.2.1 Control experiment without enzyme (ECH):

This experiment was carried out according to GP-5 using 5.3 µmol of **4a** and without using ECH enzyme (see optimization table, entry 6). ^{1}H NMR analysis of the crude reaction mixture shows no formation of desired product **6a** leaving only the unreacted **4a** (~ 100%).

3.1.2.2 Control experiment without 3′,5′-ADP activator (PAP, **5**):

This experiment was carried out according to GP-5 using 5.3 µmol of **4a**, without using 3′,5′-ADP activator (PAP, **5**: see optimization table, entry 7). ^{1}H NMR analysis of the crude reaction mixture shows formation of desired product **6a** (27%) and remaining **4a** (73%). No other products were detectable in the ^{1}H NMR spectrum.

3.2 General procedures for synthesis of starting materials

3.2.1 General procedure 1 (GP-1): Synthesis of pantetheine monomer (S1b), OJJ-03

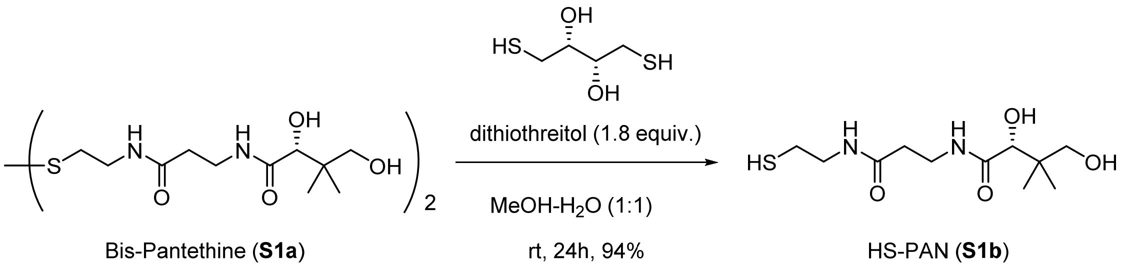

Scheme S1. Synthesis of pantetheine monomer (**S1b**) from bis-pantethine (**S1a**)

To a degassed suspension of bis-pantethine **S1a** (0.56 g, 1.0 mmol, 1.0 equiv.) in 20 mL of MeOH-H_2O (1:1, v/v) at rt was added dithiothreitol (0.28 g, 1.8 mmol, 1.8 equiv.) under nitrogen. After 24 h (monitored by TLC), the reaction mixture was concentrated and the crude product was purified using CombiFlash using 5% MeOH/DCM eluent, providing the corresponding PAN monomer **S1b** (0.52 g, 94%) as a colorless sticky oil. The spectral data matches with the data reported in the literature.[33]

R_f = 0.5 in 10% MeOH/DCM.

^{1}H NMR (300 MHz, MeOD) δ 3.89 (s, 1H), 3.59 – 3.42 (m, 2H), 3.41 (m, 2H), 3.40 – 3.29 (m, 2H), 2.65 – 2.54 (m, 2H), 2.44 (t, *J* = 6.7 Hz, 2H), 0.92 (s, 6H). **^{13}C{^{1}H} NMR** (75 MHz, MeOD) δ 176.0, 173.9, 77.4, 70.4, 43.9, 40.4, 36.4, 36.4, 24.5, 21.3, 20.9.

3.2.2 General procedure 2 (GP-2): General procedure for the Wittig reaction

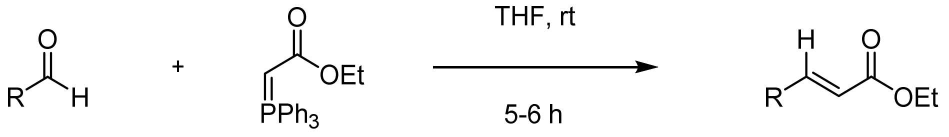

S2a (1.0 equiv.) **S2b** (1.2 equiv.) **S2c**

Scheme S2. General procedure for the syntheses of Wittig-products (**S2c**)

To a stirred suspension of aldehyde **S2a** (3 mmol, 1.0 equiv.) in 30 mL of dry THF at rt was added Wittig salt **S2b** (3.6 mmol, 1.2 equiv.) under nitrogen. After 5-6 h (monitored by TLC), the reaction mixture was concentrated and directly subjected to column chromatography using CombiFlash to obtain corresponding *trans*-Wittig product (**S2c**).

3.2.3 General procedure 3 (GP-3): General procedure for ester hydrolysis

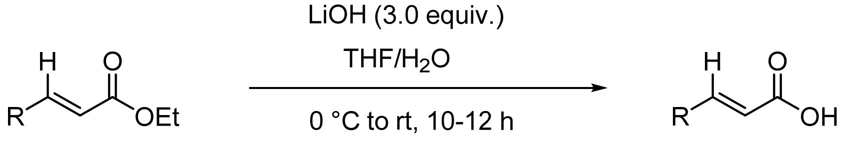

S2c (1.0 equiv.) **S3a**

Scheme S3. General procedure for the syntheses of α,β-unsaturated acids (**S3a**) from the corresponding ethyl esters

To a stirred suspension of the Wittig product **S2c** (1.0 mmol, 1.0 equiv.) in 30 mL THF/H_2O (1:1, v/v) or MeOH/H_2O (1:1, v/v) at 0 °C was added LiOH (3.0 mmol, 3.0 equiv.). The reaction mixture was allowed to acclimate at rt slowly. After 10-12 h, when almost all the starting material was consumed (monitored by TLC), the reaction mixture was concentrated. To the residue was added Et_2O (25 mL) and aqueous saturated $NaHCO_3$ solution (5 mL) with stirring. The organic layer was separated, and the aqueous layer was further extracted with 25 mL of Et_2O. The combined organic layer was discarded and the aqueous layer was acidified with dropwise addition of 3 M HCl at 0 °C (pH ~ 3 to 4). **Note**: for the synthesis of **S7c** and **S9c**, the pH of the solution was adjusted to 5-6. The aqueous layer was further extracted with DCM (30 mL x 3) and the combined organic layer was dried (Na_2SO_4), concentrated. No further purification was required.

3.2.4 General procedure 4 (GP-4): General procedure for coupling of (2*E*)-unsaturated carboxylic acids with PAN

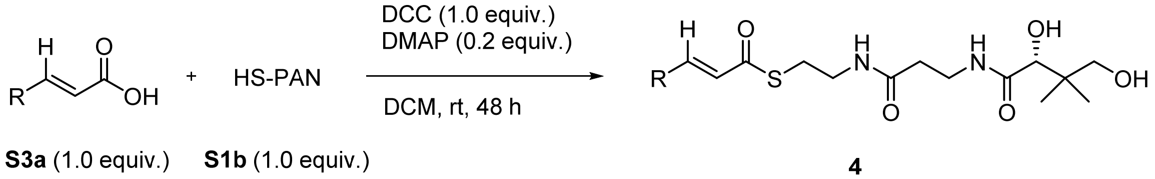

Scheme S4. General procedure for the acid/PAN coupling reaction

To a stirred suspension of α,β-unsaturated acid **S3a** (1.0 mmol, 1.0 equiv.) in 20 mL of dry DCM at 0 °C was added successively DCC (1.1 mmol, 1.1 equiv.), **S1b** (1.0 mmol, 1.0 equiv.) and DMAP (0.2 equiv.) under nitrogen. The reaction mixture was allowed to acclimate to rt slowly and after 48 h, solvent was evaporated, and the crude reaction mixture was directly purified using CombiFlash by passing 3.5-4.0% MeOH in DCM providing the desired coupling product **4**.

3.3 Syntheses of starting materials for the ECH-catalyzed hydration reaction

3.3.1 Synthesis of S-(2-acetamidoethyl) (*E*)-hex-2-enethioate (**3**), OJJ-10.

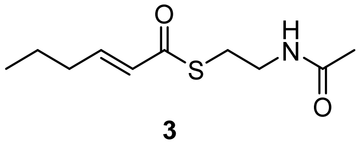

3

The title compound **3** was prepared via coupling reaction using commercially available *trans*-2-hexenoic acid (0.3 g, 2.6 mmol) and HS-NAC (1.0 mmol, 1.0 equiv.) following GP-4, providing the α,β-unsaturated thioester **3** (0.40 g, 60%) as a white semi-solid.

R_f = 0.3 in 100% EtOAc.

^{1}H NMR (500 MHz, MeOD) δ 6.92 (dt, *J* = 15.5, 7.0 Hz, 1H), 6.17 (dt, *J* = 15.5, 1.6 Hz, 1H), 3.38 – 3.30 (m, 2H), 3.06 (t, *J* = 6.7 Hz, 2H), 2.20 (qd, *J* = 7.1, 1.6 Hz, 2H), 1.92 (s, 3H), 1.51 (q, *J* = 7.4 Hz, 2H), 0.95 (t, *J* = 7.4 Hz, 3H). **^{13}C{^{1}H} NMR** (126 MHz, MeOD) δ 191.0, 173.4, 147.2, 129.7, 40.3, 40.2, 35.1, 28.9, 22.5, 22.4, 14.0.

IR (film, cm^{-1}): 3300, 2955, 2927, 2869, 1684, 1639, 1544, 1464, 1433, 1404, 1371, 1356, 1290, 1250, 1238, 1196, 1127, 1107, 1072, 1048, 1025, 1003, 771, 603, 514.

HRMS (ESI$^+$) m/z: [M+Na]$^+$ calculated for [$C_{10}H_{17}NNaO_2S$]$^+$= 238.0872, found 238.0874, Δ = 0.29 ppm.

3.3.2 Synthesis of (*R*)-*S*-(2-(3-(2,4-dihydroxy-3,3-dimethylbutanamido)propanamido)ethyl) (*E*)-hex-2-enethioate (**4a**), OJJ-04.

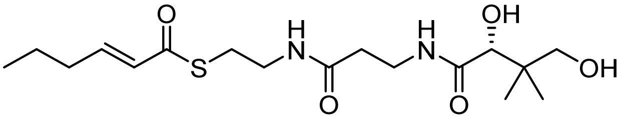

4a

The title compound **4a** was prepared via coupling reaction using commercially available *trans*-2-hexenoic acid (0.3 g, 2.6 mmol) following GP-4, providing **4a** (0.51 g, 52%) as a colorless sticky oil.

R_f = 0.6 in 10% MeOH/DCM.

^{1}H NMR (500 MHz, MeOD) δ 6.93 (dt, *J* = 15.5, 7.0 Hz, 1H), 6.17 (dt, *J* = 15.5, 1.5 Hz, 1H), 3.89 (s, 1H), 3.53 – 3.44 (m, 3H), 3.43 – 3.35 (m, 2H), 3.34 (m, 1H), 3.06 (t, *J* = 6.7 Hz, 2H), 2.41 (t, *J* = 6.7 Hz, 2H), 2.20 (qd, *J* = 7.1, 1.5 Hz, 2H), 1.51 (sextet, *J* = 7.4 Hz, 2H), 0.95 (t, *J* = 7.4 Hz, 3H), 0.92 (s, 6H); **^{13}C{^{1}H} NMR** (126 MHz, MeOD) δ 191.1, 176.0, 173.9, 147.3, 129.7, 77.3, 70.3, 40.4, 40.2, 36.4, 36.3, 35.1, 28.9, 22.4, 21.3, 20.9, 14.0.

IR (film, cm^{-1}): 3294, 2959, 2931, 2873, 1631, 1526, 1462, 1433, 1363, 1336, 1287, 1244, 1200, 1165, 1064, 1031, 970, 804, 760, 639.

HRMS (ESI$^+$) m/z: [M+Na]$^+$ calculated for [$C_{17}H_{30}N_2NaO_5S$]$^+$= 397.1768, found 397.1785, Δ = 3.95 ppm.

$[\alpha]_D^{20}$ = +12.0 (*c*= 0.44 g/mL, MeOH)

3.3.3 Synthesis of (*R*)-*S*-(2-(3-(2,4-dihydroxy-3,3-dimethylbutanamido)propanamido)ethyl) (*E*)-dec-2-enethioate (**4b**), OJJ-24.

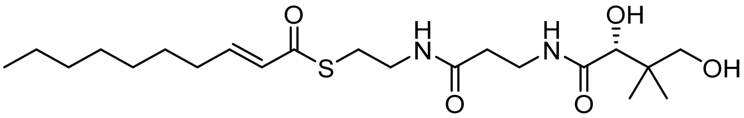

4b

The title compound **4b** was prepared via coupling reaction using commercially available *trans*-2-decenoic acid (0.21 g, 1.2 mmol) following GP-4, providing **4b** (0.25 g, 47%) as a colorless sticky liquid.

$\boldsymbol{R}_\mathrm{f}$ = 0.6 in 10% MeOH/DCM.

^{1}H NMR (500 MHz, MeOD) δ 6.93 (dt, *J* = 15.6, 7.0 Hz, 1H), 6.17 (dt, *J* = 15.5, 1.6 Hz, 1H), 3.89 (s, 1H), 3.54 – 3.32 (m, 6H), 3.06 (t, *J* = 6.7 Hz, 2H), 2.41 (t, *J* = 6.7 Hz, 2H), 2.22 (qd, *J* = 7.1, 1.6 Hz, 2H), 1.53 – 1.43 (m, 2H), 1.33 (td, *J* = 8.9, 5.4 Hz, 8H), 0.91 (s, 6H), 0.90 (t, *J* = 7.4 Hz, 3H); **^{13}C{^{1}H} NMR** (126 MHz, MeOD) δ 191.0, 176.0, 173.9, 147.6, 129.5, 77.2, 70.4, 40.4, 40.2, 36.4, 36.3, 33.1, 32.9, 30.24, 30.16, 29.1, 28.9, 23.7, 21.3, 20.9, 14.4.

IR (film, cm^{-1}): 3336, 3276, 3083, 2924, 2854, 1642, 1543, 1446, 1408, 1391, 1367, 1291, 1245, 1194, 1155, 1089, 1077, 1032, 967, 910, 886, 690, 667, 627.

HRMS (ESI$^+$) m/z: [M+Na]$^+$ calculated for [$C_{21}H_{38}N_2NaO_5S$]$^+$= 453.2394, found 453.2412, Δ = 3.5 ppm.

$[\alpha]_D^{20}$ = +11.2 (c= 0.36 g/mL, MeOH)

3.3.4 Synthesis of (*R*)-*S*-(2-(3-(2,4-dihydroxy-3,3-dimethylbutanamido)propanamido)ethyl) (*E*)-5-methylhex-2-enethioate (**4c**), OJJ-129.

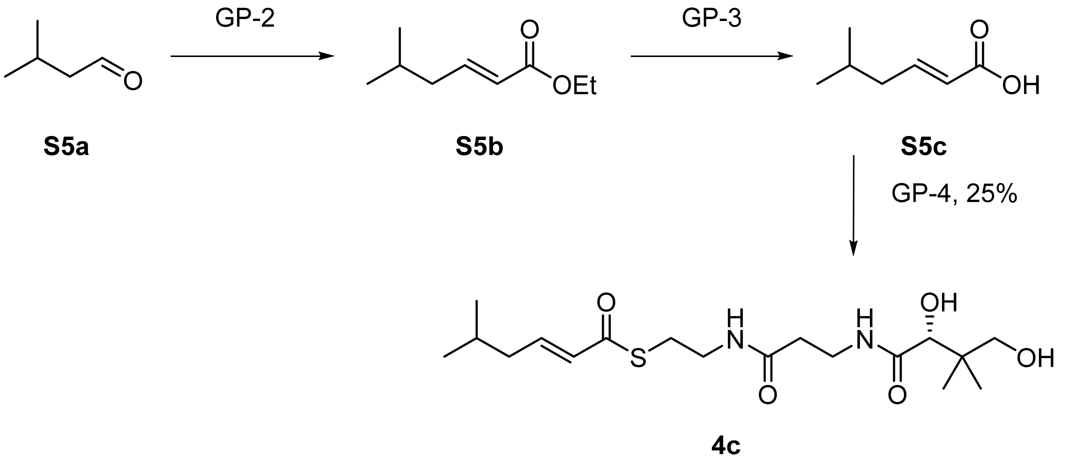

Scheme S5. Synthesis of **4c** from isovaleraldehyde **S5a**

3.3.4.1 Ethyl (*E*)-5-methylhex-2-enoate (**S5b**), OJJ-121.

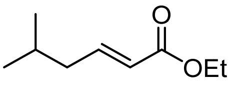

S5b

The corresponding Wittig product (**S5b**) was volatile in nature and was prepared with care following GP-2 using commercially available isovaleraldehyde (**S5a**) and this intermediate was used in the next step without further purification.

3.3.4.2 (*E*)-5-methylhex-2-enoic acid (**S5c**), OJJ-125.

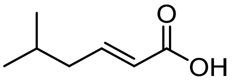

S5c

The α,β-unsaturated acid (**S5c**) was prepared from **S5b** following GP-3 and this intermediate was used in the next step without further purification.

3.3.4.3 (*R*)-*S*-(2-(3-(2,4-dihydroxy-3,3-dimethylbutanamido)propanamido)ethyl) (*E*)-5-methylhex-2-enethioate (**4c**), OJJ-129.

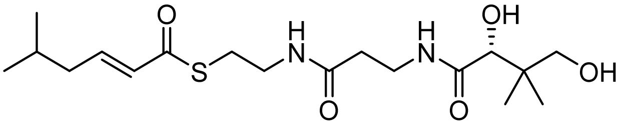

4c

The α,β-unsaturated thioester **4c** was prepared following GP-4 via acid-thiol coupling reaction using corresponding α,β-unsaturated acid **S5c** (0.1g, 0.78 mmol), providing **4c** (73.0 mg, 25%) as a colorless sticky liquid.

$\boldsymbol{R_f}$ = 0.6 in 10% MeOH/DCM.

^{1}H NMR (500 MHz, MeOD) δ 6.91 (ddd, *J* = 15.0, 7.9, 7.2 Hz, 1H), 6.17 (dd, *J* = 15.5, 1.3 Hz, 1H), 3.89 (s, 1H), 3.53 – 3.41 (m, 3H), 3.41 – 3.33 (m, 3H), 3.07 (t, *J* = 6.8 Hz, 2H), 2.41 (t, *J* = 6.6 Hz, 2H), 2.12 (ddd, *J* = 7.5, 6.7, 1.5 Hz, 2H), 1.78 (dp, *J* = 13.4, 6.7 Hz, 1H), 0.94 (dd, *J* = 6.7, 0.8 Hz, 6H), 0.92 (s, 6H); **^{13}C{^{1}H} NMR** (126 MHz, MeOD) δ 191.0, 176.1, 173.9, 146.3, 130.6, 77.3, 70.4, 42.3, 40.4, 40.2, 36.4, 36.3, 29.1, 28.9, 22.7, 21.3, 20.9.

IR (film, cm^{-1}): 3355, 3276, 3086, 2953, 2927, 2870, 1663, 1645, 1629, 1533, 1462, 1444, 1411, 1345, 1313, 1275, 1237, 1189, 1141, 1087, 890, 736. 675, 591.

HRMS (ESI+) m/z: [M+Na]+ calculated for $[C_{18}H_{32}N_2NaO_5S]^+$= 411.1924, found 411.1924, Δ = 0 ppm.

[α]$_D^{20}$ = +22.6 (c= 0.42 g/mL, MeOH)

3.3.5 Synthesis of (*R*)-*S*-(2-(3-(2,4-dihydroxy-3,3-dimethylbutanamido)propanamido)ethyl) (*E*)-3-cyclohexylprop-2-enethioate (**4d**), OJJ-93.

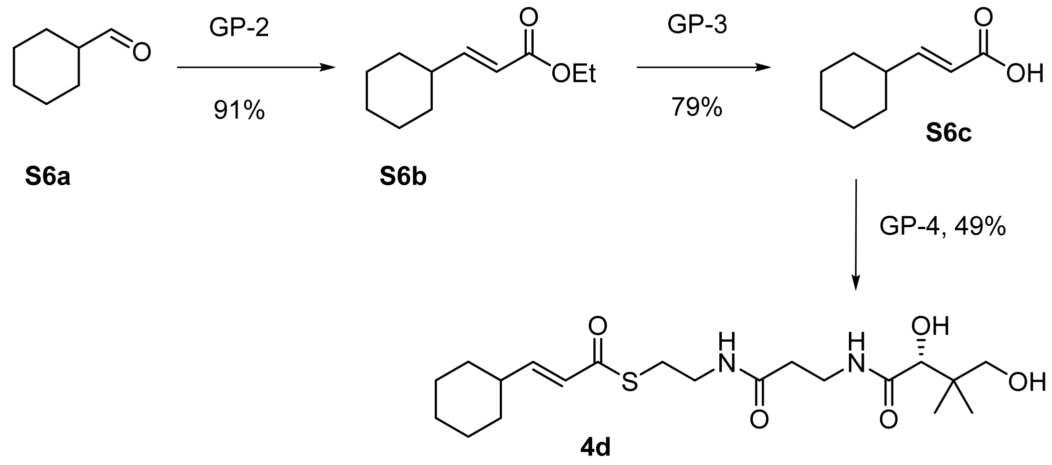

Scheme S6. Synthesis of **4d** from cyclohexanecarboxaldehyde **S6a**

3.3.5.1 Ethyl (*E*)-3-cyclohexylacrylate (**S6b**), OJJ-71.

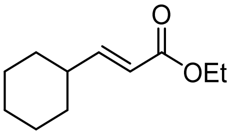

S6b

The Wittig product **S6b** was prepared from commercially available cyclohexanecarboxaldehyde **S6a** (0.2 g, 1.78 mmol) following GP-2, providing **S6b** (0.3 g, 91%) as a colorless oil.

***R*f** = 0.7 in 10% EtOAc/Hex.

^{1}H NMR (300 MHz, $CDCl_3$) δ 6.97 – 6.83 (m, 1H), 5.75 (dt, *J* = 15.8, 1.2 Hz, 1H), 4.25 – 4.11 (m, 2H), 2.19 – 2.03 (m, 1H), 1.82 – 1.61 (m, 5H), 1.38 – 1.22 (m, 5H), 1.21 – 1.04 (m, 3H); **^{13}C{^{1}H} NMR** (75 MHz, $CDCl_3$) δ 167.3, 154.4, 119.1, 60.2, 40.5, 31.9, 26.1, 25.9, 14.4.

IR (film, cm^{-1}): 2980, 2924, 2852,1717, 1650, 1447, 1367, 1297, 1262, 1225, 1190, 1166, 1136, 1099, 1063, 1034, 982, 966, 850, 708.

HRMS (ESI^{+}) m/z: [M+H]$^{+}$ calculated for [$C_{11}H_{19}O_2$]$^{+}$= 183.1380, found 183.1379, Δ = −0.17 ppm.

3.3.5.2 (*E*)-3-cyclohexylacrylic acid (**S6c**), OJJ-72.

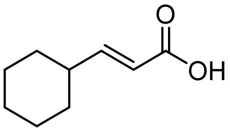

S6c

The α,β-unsaturated acid (**S6c**) was prepared following GP-3 using corresponding ester **S6b** and this intermediate was used in the next step without further purification.

3.3.5.3 (*R*)-*S*-(2-(3-(2,4-dihydroxy-3,3-dimethylbutanamido)propanamido)ethyl) (*E*)-3-cyclohexylprop-2-enethioate (**4d**), OJJ-93**.**

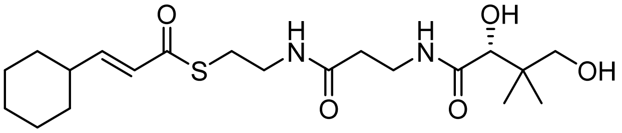

4d

The α,β-unsaturated thioester **4d** was prepared via coupling reaction from corresponding α,β-unsaturated acid **S6c** (0.2 g, 1.3 mmol) following GP-4 providing **4d** (0.26 g, 49%) as a colorless sticky liquid.

$\boldsymbol{R_f}$ = 0.5 in 10% MeOH/DCM.

^{1}H NMR (500 MHz, MeOD) δ 6.89 – 6.83 (m, 1H), 6.12 (dq, *J* = 15.6, 1.4 Hz, 1H), 3.89 (s, 1H), 3.53 – 3.44 (m, 3H), 3.43 – 3.35 (m, 2H), 3.34 (d, *J* = 6.8 Hz, 1H), 3.10–3.03 (m, 2H), 2.44 – 2.37 (m, 2H), 2.21 – 2.13 (m, 1H), 1.81 – 1.73 (m, 4H), 1.73 – 1.65 (m, 1H), 1.41 – 1.29 (m, 2H), 1.28 – 1.11 (m, 3H), 0.92 (s, 6H); **^{13}C{^{1}H} NMR** (126 MHz, MeOD) δ 191.4, 176.0, 173.9, 152.1, 127.2, 77.2, 70.3, 41.7, 40.4, 40.2, 36.4, 36.3, 32.8, 28.9, 27.0, 26.7, 21.3, 20.9.

IR (film, cm^{-1}): 3293, 3084, 2924, 2851, 1645, 1626, 1526, 1446, 1362, 1290, 1273, 1073, 1018, 975, 964. 914, 888, 849, 823, 773, 643, 589, 553.

HRMS (ESI^+) m/z: $[M+Na]^+$ calculated for $[C_{20}H_{34}N_2NaO_5S]^+$= 437.2081, found 437.2095, Δ = 2.94 ppm.

$[\alpha]_D^{20}$ = +19.7 (c= 0.33 g/mL, MeOH)

3.3.6 Synthesis of *tert*-butyl (*R*, *E*)-4-(3-((2-(3-(2,4-dihydroxy-3,3-dimethylbutanamido)propanamido)ethyl)thio)-3-oxoprop-1-en-1-yl)piperidine-1-carboxylate (**4e**), OJJ-48.

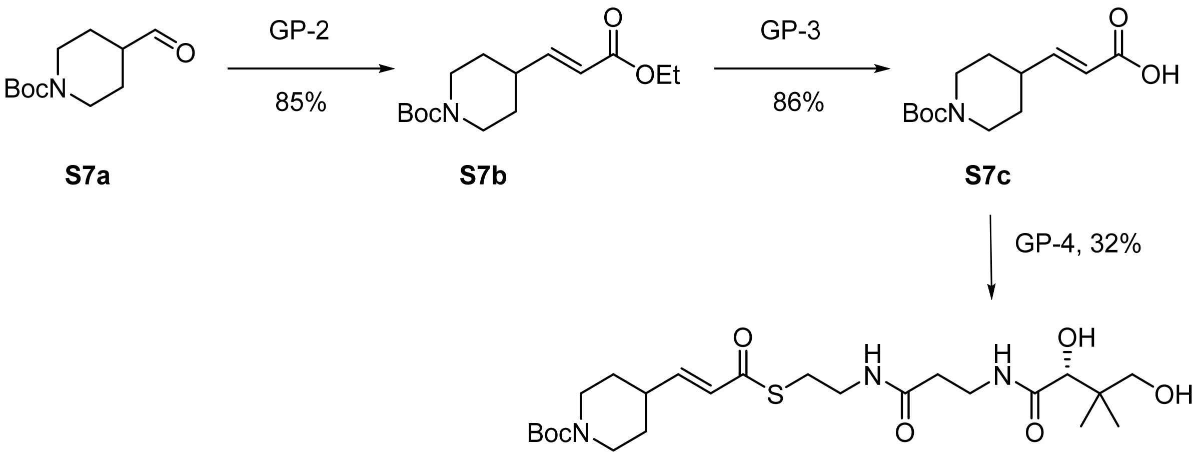

4e

Scheme S7. Synthesis of **4e** from aldehyde **S7a**

3.3.6.1 *tert*-Butyl (*E*)-4-(3-ethoxy-3-oxoprop-1-en-1-yl)piperidine-1-carboxylate (**S7b**), OJJ-39.

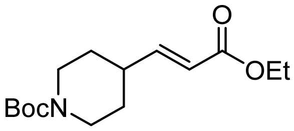

S7b

The Wittig product **S7b** was prepared from commercially available aldehyde **S7a** (0.5 g, 2.3 mmol), following GP-2, providing **S7b** (0.57 g, 85%) as a colorless liquid.

***R*$_f$** = 0.65 in 20% EtOAc/Hex.

^{1}H NMR (500 MHz, $CDCl_3$) δ 6.89 (dd, *J* = 15.8, 6.6 Hz, 1H), 5.80 (dd, *J* = 15.8, 1.5 Hz, 1H), 4.19 (q, *J* = 7.1 Hz, 2H), 4.13 (dd, *J* = 19.7, 6.5 Hz, 2H), 2.76 (t, *J* = 12.6 Hz, 2H), 2.34 – 2.23 (m, 1H), 1.78 – 1.67 (m, 2H), 1.46 (s, 9H), 1.40 – 1.30 (m, 2H), 1.29 (t, *J* = 7.1 Hz,

3H).[13]**C{^{1}H} NMR** (126 MHz, $CDCl_3$) δ 166.9, 154.9, 151.8, 120.2, 79.7, 60.5, 43.7, 38.8, 30.8, 28.6, 14.4.

IR (film, cm^{-1}): 2979, 2937, 2861, 1716, 1686, 1655, 1468, 1446, 1418, 1388, 1374, 1362, 1303, 1262, 1183, 1160, 1140, 1089, 1035, 1006, 994, 969, 940, 873, 864, 813, 767, 718.

HRMS (ESI^+) m/z: $[M+Na]^+$ calculated for $[C_{15}H_{25}NNaO_4]^+$= 306.1676, found 306.1664, Δ = −4.52 ppm.

3.3.6.2 (*E*)-3-(1-(*tert*-butoxycarbonyl)piperidin-4-yl)acrylic acid (**S7c**), OJJ-46.

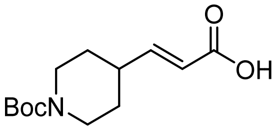

S7c

The α,β-unsaturated acid (**S7c**) was prepared from corresponding ester **S7b** following GP-3 and this intermediate was used in the next step without further purification.

3.3.6.3 *tert*-butyl (*R,E*)-4-(3-((2-(3-(2,4-dihydroxy-3,3-dimethylbutanamido)propanamido)ethyl)thio)-3-oxoprop-1-en-1-yl)piperidine-1-carboxylate (**4e**), OJJ-48.

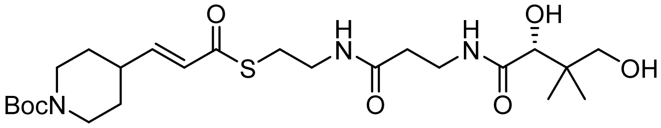

4e

The α,β-unsaturated thioester **4e** was prepared via coupling reaction from corresponding α,β-unsaturated acid **S7c** (0.32 g, 1.27 mmol) following GP-4 providing **4e** (0.21 g, 32%) as a colorless sticky liquid.

$\boldsymbol{R}_\mathbf{f}$ = 0.6 in 10% MeOH/DCM.

[1]**H NMR** (500 MHz, MeOD) δ 6.86 (dd, *J* = 15.7, 6.7 Hz, 1H), 6.18 (dd, *J* = 15.7, 1.4 Hz, 1H), 4.13 – 4.05 (m, 2H), 3.89 (s, 1H), 3.53 – 3.43 (m, 3H), 3.42 – 3.36 (m, 2H), 3.35 (m, 1H), 3.07 (t, *J* = 6.7 Hz, 2H), 2.82 (bs, 2H), 2.41 (t, *J* = 6.7 Hz, 2H), 2.39 – 2.34 (m, 1H), 1.75 (dd, *J* = 13.8, 3.5 Hz, 2H), 1.45 (s, 9H), 1.35 – 1.27 (m, 2H), 0.92 (s, 6H); [13]**C{^{1}H} NMR** (126 MHz, MeOD) δ 191.1, 176.0, 173.8, 156.3, 149.7, 128.1, 81.0, 77.2, 70.2, 44.8, 40.4, 40.1, 39.6, 36.4, 36.3, 31.6, 29.0, 28.7, 21.3, 20.9.

IR (film, cm^{-1}): 3323, 2932, 2873, 2845, 2474, 1650, 1629, 1532, 1448,1417, 1365, 1299, 1265, 1248, 1146, 1106, 1078, 1044, 1031, 1019, 1008, 975, 961, 939, 875, 845, 791, 773, 759, 640, 522.

HRMS (ESI^+) m/z: $[M+Na]^+$ calculated for $[C_{24}H_{41}N_3NaO_7S]^+$= 538.2557, found 538.2567, Δ = 1.52 ppm.

$[\alpha]_D^{20}$ = +14.5 (c= 0.42 g/mL, MeOH)

3.3.7 Synthesis of (*R*)-*S*-(2-(3-(2,4-dihydroxy-3,3-dimethylbutanamido)propanamido)ethyl) (*E*)-7-bromohept-2-enethioate (**4f**), OJJ-112.

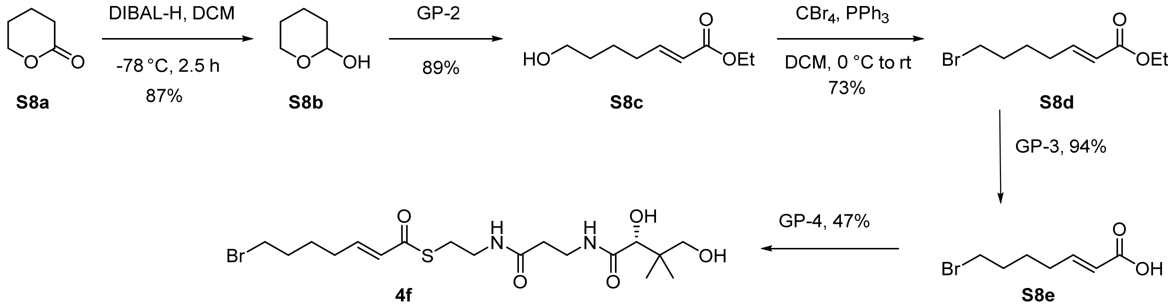

Scheme S8. Synthesis of **4f** from δ-valerolactone **S8a**

3.3.7.1 Tetrahydro-2H-pyran-2-ol (**S8b**), OJJ-82.

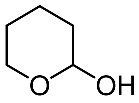

S8b

The lactol **S8b** was prepared from δ-valerolactone **S8a** (2.0 g, 19.97 mmol) adapting the literature reported procedure[34], providing **S8b** (1.77 g, 87%) as a colorless liquid. The spectral data matches with the reported literature data[35].

***R*$_f$** = 0.5 in 50% EtOAc/Hex.

^{1}H NMR (500 MHz, $CDCl_3$) δ 4.90 – 4.83 (m, 1H), 4.01 – 3.97 (m, 1H), δ 3.95 (dd, *J* = 5.1, 2.0 Hz, 1H), 3.51 (ddd, *J* = 11.4, 6.3, 3.8 Hz, 1H), 1.90 – 1.73 (m, 2H), 1.62 – 1.42 (m, 4H); **$^{13}C\{^1H\}$ NMR** (126 MHz, $CDCl_3$) δ 94.6, 64.0, 32.0, 25.3, 20.4.

3.3.7.2 Ethyl (*E*)-7-hydroxyhept-2-enoate (**S8c**), OJJ-82-wittig.

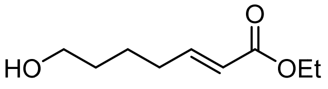

S8c

The Wittig product **S8c** was prepared from lactol **S8b** (2.04 g, 20.0 mmol) following the literature procedure[35], providing the ester **S8c** (3.1 g, 89%). The spectral data matches with the reported literature data[35].

***R*$_f$** = 0.5 in 50% EtOAc/Hex.

^{1}H NMR (500 MHz, $CDCl_3$) δ 6.91 (dt, *J* = 15.7, 6.9 Hz, 1H), 5.77 (dt, *J* = 15.6, 1.6 Hz, 1H), 4.13 (q, *J* = 7.2 Hz, 2H), 3.58 (t, *J* = 6.3 Hz, 2H), 2.26 (bs, 1H), 2.19 (qd, *J* = 7.0, 1.6 Hz, 2H), 1.59 – 1.44 (m, 4H), 1.23 (t, *J* = 7.2 Hz, 3H); **^{13}C{^{1}H} NMR** (126 MHz, $CDCl_3$) δ 166.9, 149.1, 121.5, 62.3, 60.3, 32.1, 31.9, 24.3, 14.3.

3.3.7.3 Ethyl (*E*)-7-bromohept-2-enoate (**S8d**), OJJ-109.

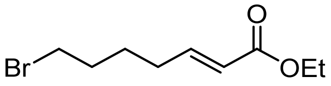

S8d

To a stirred suspension of alcohol **S8c** (0.2 g, 1.16 mmol, 1equiv.) in 10 mL of dry DCM at 0 °C was added successively tetrabromomethane (0.42 g, 1.28 mmol, 1.1 equiv.) and triphenylphosphine (0.34 g, 1.28 mmol, 1.1 equiv.). After 15 min, the reaction mixture was acclimated to rt slowly. After 2 h, when almost all the starting was consumed (monitored by TLC), the reaction mixture was concentrated and directly subjected to purification using CombiFlash (12 g Silica, 5 % EtOAc/hexane) providing desired bromo compound **S8d** (0.2 g, 73%) as a colorless oil.

***R*$_f$** = 0.45 in 10% EtOAc/Hex.

^{1}H NMR (300 MHz, $CDCl_3$) δ 6.93 (dt, *J* = 15.6, 6.9 Hz, 1H), 5.83 (dt, *J* = 15.6, 1.6 Hz, 1H), 4.18 (q, *J* = 7.1 Hz, 2H), 3.40 (t, *J* = 6.7 Hz, 2H), 2.23 (qd, *J* = 7.3, 1.6 Hz, 2H), 1.96 – 1.81 (m, 2H), 1.70 – 1.54 (m, 2H), 1.28 (t, *J* = 7.1 Hz, 3H); **^{13}C{^{1}H} NMR** (75 MHz, $CDCl_3$) δ 166.6, 148.2, 122.1, 60.3, 33.3, 32.2, 31.3, 26.7, 14.4.

IR (film, cm^{-1}): 2937, 2866, 1714, 1652, 1444, 1390, 1366, 1265, 1251, 1185, 1133, 1094, 1038, 977, 929, 874, 707, 642, 560.

HRMS (ESI+) m/z: $[M+H]^+$ calculated for $[C_9H_{16}BrO_2]^+$= 235.0328, found 235.0323, Δ = −2.13 ppm.

3.3.7.4 (E)-7-bromohept-2-enoic acid (**S8e**), OJJ-110.

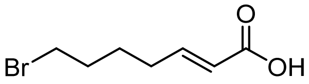

S8e

The α,β-unsaturated acid (**S8e**) was prepared from corresponding ester **S8d** following GP-3 and this intermediate was used in the next step without further purification.

3.3.7.5 (*R*)-*S*-(2-(3-(2,4-dihydroxy-3,3-dimethylbutanamido)propanamido)ethyl) (*E*)-7-bromohept-2-enethioate, **4f**, OJJ-112.

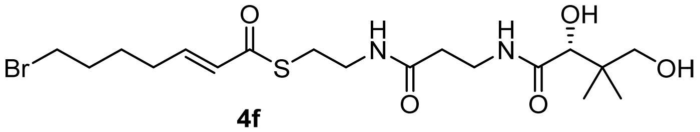

4f

The α,β-unsaturated thioester **4f** was prepared via coupling reaction from corresponding α,β-unsaturated acid **S8e** (0.15 g, 0.72 mmol) following GP-4 (0.16 g, 47%) as a colorless sticky liquid.

$\boldsymbol{R}_\mathbf{f}$ = 0.6 in 10% MeOH/DCM.

^{1}**H NMR** (500 MHz, MeOD) δ 6.92 (dt, *J* = 15.5, 7.0 Hz, 1H), 6.20 (dt, *J* = 15.5, 1.6 Hz, 1H), 3.89 (s, 1H), 3.50 – 3.44 (m, 4H), 3.43 – 3.36 (m, 2H), 3.35 – 3.34 (m, 2H), 3.07 (t, *J* = 6.7 Hz, 2H), 2.41 (t, *J* = 6.6 Hz, 2H), 2.27 (qd, *J* = 7.2, 1.6 Hz, 2H), 1.87 (dt, *J* = 14.9, 6.7 Hz, 2H), 1.63 (p, *J* = 7.5 Hz, 2H), 0.92 (s, 6H); ^{13}**C{^{1}H} NMR** (126 MHz, MeOD) δ 191.0, 176.0, 174.0, 146.7, 129.9, 77.3, 70.4, 40.4, 40.1, 36.4, 36.3, 33.9, 33.3, 32.1, 29.0, 27.6, 21.3, 21.0.

IR (film, cm^{-1}): 3294, 3084, 2932, 2872, 1632, 1525, 1433, 1361, 1286, 1246, 1197, 1075, 1040, 969, 884, 810, 738, 638, 556.

HRMS (ESI+) m/z: $[M+H]^+$ calculated for $[C_{18}H_{32}BrN_2O_5S]^+$= 467.1210, found 467.1222, Δ = 2.18 ppm.

$[\alpha]_D^{20}$ = +18.2 (c= 0.4 g/mL, MeOH)

3.3.8 Synthesis of (*R*)-*S*-(2-(3-(2,4-dihydroxy-3,3-dimethylbutanamido)propanamido)ethyl) (*E*)-6-(1,3-dioxolan-2-yl)hex-2-enethioate (**4g**), OJJ-124.

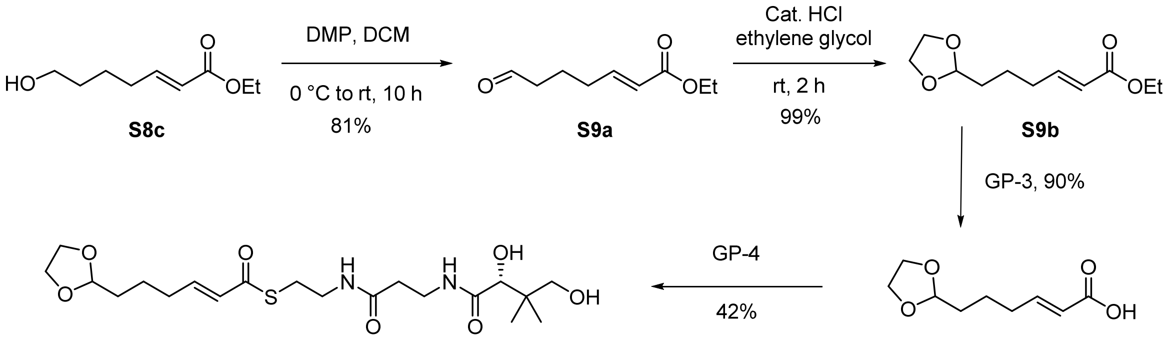

Scheme S9. Synthesis of **4g** from **S8c**

3.3.8.1 Ethyl (*E*)-7-oxohept-2-enoate (**S9a**), OJJ-111.

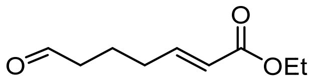

S9a

To a stirred suspension of alcohol **S8c** (0.5 g, 2.9 mmol, 1 equiv.) in 40 mL dry DCM at 0 °C was added Dess–Martin periodinane, DMP, (0.92 g, 2.2 mmol, 1.1 equiv.). The reaction mixture was allowed to acclimate to rt slowly. After 2 h, it was quenched with saturated aqueous $NaHCO_3$ solution (30 mL). Subsequently, the reaction mixture was extracted with DCM (3x80 mL) and the combined organic layer was dried (Na_2SO_4), concentrated and the crude residue was purified using CombiFlash by passing 10% EtOAc/hexane providing the desired aldehyde **S9a** (0.4 g, 81%) as a colorless oil.

$\boldsymbol{R_f}$ = 0.35 in 20% EtOAc/Hex.

^{1}H NMR (500 MHz, $CDCl_3$) δ 9.75 (t, *J* = 1.5 Hz, 1H), 6.88 (dt, *J* = 15.7, 6.9 Hz, 1H), 5.81 (dt, *J* = 15.7, 1.6 Hz, 1H), 4.15 (q, *J* = 7.1 Hz, 2H), 2.46 (td, *J* = 7.3, 1.5 Hz, 2H), 2.26 – 2.18 (m, 2H), 1.78 (p, *J* = 7.3 Hz, 2H), 1.25 (t, *J* = 7.2 Hz, 3H). **^{13}C{^{1}H} NMR** (126 MHz, $CDCl_3$) δ 201.7, 166.5, 147.6, 122.3, 60.34, 43.0, 31.3, 20.4, 14.3.

IR (film, cm^{-1}): 2981, 2938, 1713, 1653, 1446, 1391, 1367, 1310, 1265, 1191, 1155, 1095, 1040, 981, 851, 711, 668.

HRMS (ESI^+) m/z: $[M+H]^+$ calculated for $[C_9H_{15}O_3]^+$= 171.1016, found 171.1011, Δ = −1.80 ppm.

3.3.8.2 Ethyl (*E*)-6-(1,3-dioxolan-2-yl)hex-2-enoate (**S9b**), OJJ-118.

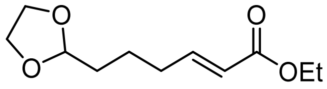

S9b

To a stirred suspension of aldehyde **S9a** (0.16 g, 0.94 mmol) in 2.0 mL ethylene glycol at rt was added catalytic amount of conc. HCl (8.0 µL). After 2 h, the reaction mixture was quenched with 1.0 mL of saturated aqueous $NaHCO_3$ solution, diluted with 5.0 mL DCM and 4.0 mL H_2O. Organic layer was separated, and the aqueous layer was further extracted with DCM (2x5 mL). The combined organic layer was evaporated to dryness producing desired acetal **S9b** (0.19 g) in 95% crude yield, characterized by crude ^{1}H NMR and this intermediate was used in the next step without further purification.

$\boldsymbol{R_f}$ = 0.4 in 20% EtOAc/Hex.

^{1}H NMR (500 MHz, $CDCl_3$) δ 6.93 (dt, *J* = 15.7, 6.9 Hz, 1H), 5.85 – 5.77 (m, 1H), 4.84 (t, *J* = 4.6 Hz, 1H), 4.16 (q, *J* = 7.1 Hz, 2H), 3.99 – 3.89 (m, 2H), 3.88 – 3.78 (m, 2H), 2.24 (qd, *J* = 7.1, 1.6 Hz, 2H), 1.71 – 1.64 (m, 2H), 1.62 – 1.54 (m, 2H), 1.27 (td, *J* = 7.1, 0.6 Hz, 3H).

3.3.8.3 (*E*)-6-(1,3-dioxolan-2-yl)hex-2-enoic acid (**S9c**), OJJ-119.

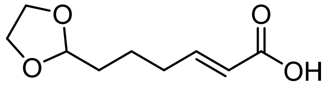

S9c

The α,β-unsaturated acid **S9c** was prepared from corresponding ester **S9b** (0.31 g, 1.43 mmol) following GP-3 providing **S9c** (0.24 g, 90%) as a colorless liquid. The compound was characterized by ^{1}H NMR and this intermediate was used in the next step without further purification.

^{1}H NMR (500 MHz, $CDCl_3$) δ 7.12 – 7.01 (m, 1H), 5.84 (ddd, *J* = 15.6, 4.0, 2.1 Hz, 1H), 4.87 (dq, *J* = 5.2, 2.1 Hz, 1H), 3.95 (m, 2H), 3.88 – 3.82 (m, 2H), 2.28 (qd, *J* = 7.5, 3.4 Hz, 2H), 1.74 – 1.65 (m, 2H), 1.65 – 1.56 (m, 2H).

3.3.8.4 (*R*)-*S*-(2-(3-(2,4-dihydroxy-3,3-dimethylbutanamido)propanamido)ethyl) (*E*)-6-(1,3-dioxolan-2-yl)hex-2-enethioate (**4g**), OJJ-124.

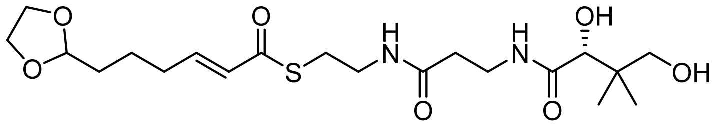

4g

The α,β-unsaturated thioester **4g** was prepared via coupling reaction from corresponding α,β-unsaturated acid **S9c** (0.12 g, 0.65 mmol) following GP-4 in providing **4g** (0.12 g, 42%) as a colorless sticky liquid.

R_f = 0.55 in 10% MeOH/DCM.

^{1}H NMR (500 MHz, MeOD) δ 6.92 (dt, *J* = 15.6, 7.0 Hz, 1H), 6.19 (dt, *J* = 15.5, 1.5 Hz, 1H), 4.83 (t, *J* = 4.4 Hz, 1H), 3.95 – 3.92 (m, 2H), 3.89 (s, 1H), 3.84 – 3.80 (m, 2H), 3.53 – 3.42 (m, 3H), 3.42 – 3.34 (m, 3H), 3.07 (t, *J* = 6.7 Hz, 2H), 2.41 (t, *J* = 6.7 Hz, 2H), 2.27 (qd, *J* = 7.1, 1.6 Hz, 2H), 1.70–1.54 (m, 4H), 0.92 (s, 6H); **^{13}C{^{1}H} NMR** (126 MHz, MeOD) δ 191.0, 176.1, 173.9, 147.1, 129.8, 105.3, 77.3, 70.4, 65.9, 40.4, 40.2, 36.4, 36.3, 34.2, 32.8, 28.9, 23.4, 21.3, 20.9.

IR (film, cm^{-1}): 3305, 3084, 2934, 2873, 1645, 1525, 1435, 1409, 1361, 1286, 1237, 1182, 1139, 1032, 973, 946, 885, 805.

HRMS (ESI$^+$) m/z: [M+H]$^+$ calculated for $[C_{20}H_{35}N_2O_7S]^+$= 447.2159, found 447.2191, Δ = 2.54 ppm.

$[\alpha]_D^{20}$ = +18.2 (c= 0.33 g/mL, MeOH)

3.3.9 Synthesis of methyl (*R,E*)-7-((2-(3-(2,4-dihydroxy-3,3-dimethylbutanamido)propanamido)ethyl)thio)-7-oxohept-5-enoate (**4h**), OJJ-140.

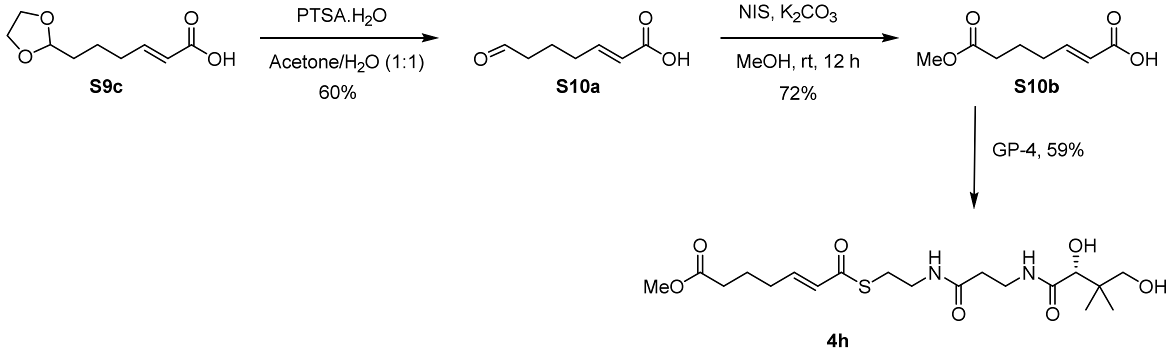

Scheme S10. Synthesis of **4h** from **S9c**

3.3.9.1 (*E*)-7-oxohept-2-enoic acid, (**S10a**), OJJ-126.

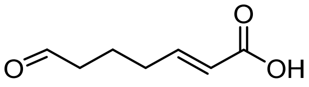

S10a

To a stirred suspension of acetal **S9c** (0.12 g, 0.65 mmol, 1.0 equiv.) in acetone/H_2O (4.0 mL, 4:1, v/v) at rt was added *p*-toluenesulfonic acid monohydrate (0.012 g, 0.065 mmol, 0.1 equiv.). After 12 h, when almost all the starting material was consumed (monitored by TLC), the reaction was quenched with the addition of 2.0 mL of saturated aqueous $NaHCO_3$ solution. Solvent was removed by evaporation using rotary evaporator and the reaction mixture was extracted with DCM (3x5 mL). Combined organic layer was evaporated to dryness producing aldehyde **S10a** (0.55 g, 60% crude yiled) and this intermediate was used in the next step without further purification.

3.3.9.2 (*E*)-7-methoxy-7-oxohept-2-enoic acid, (**S10b**), OJJ-127.

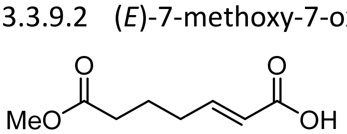

S10b

To a stirred suspension of aldehyde **S10a** (0.08 g, 0.62 mmol, 1 equiv.) in 5.0 mL of dry MeOH at rt was added successively K_2CO_3 (0.26 g, 1.9 mmol, 3.0 equiv.) and *N*-iodosuccinimide (0.31 g, 1.4 mmol, 2.2 equiv.). After 3 h, when almost all the starting material was consumed (monitored by TLC), the reaction was quenched with aqueous saturated $Na_2S_2O_3.5H_2O$ solution (10 mL). Solvent was reduced to 1/3 of total volume using rotary evaporator. The reaction mixture was diluted and extracted with EtOAc (3x 50 mL), combined organic layer was dried (Na_2SO_4) and concentrated producing

corresponding crude ester **S10b** (0.07 g, 72 %) as a colorless liquid. The crude product was characterized by NMR and this intermediate was used in the next step without further purification.

^{1}H NMR (500 MHz, $CDCl_3$) δ 7.04 (dt, *J* = 15.7, 6.9 Hz, 1H), 5.85 (dt, *J* = 15.6, 1.6 Hz, 1H), 3.68 (s, 3H), 2.35 (t, *J* = 7.4 Hz, 2H), 2.31 – 2.24 (m, 2H), 1.82 (p, *J* = 7.5 Hz, 2H). **^{13}C{^{1}H} NMR** (126 MHz, $CDCl_3$) δ 173.6, 171.9, 150.8, 121.6, 51.8, 33.3, 31.6, 23.2.

3.3.9.3 Methyl (*R,E*)-7-((2-(3-(2,4-dihydroxy-3,3-dimethylbutanamido)propanamido)ethyl)thio)-7-oxohept-5-enoate (**4h**), OJJ-140.

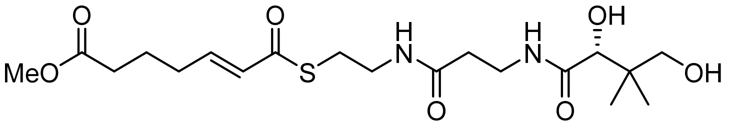

4h

The α,β-unsaturated thioester **4h** was prepared via coupling reaction from corresponding α,β-unsaturated acid **S10b** (0.07 g, 0.41 mmol) following GP-4 providing **4h** (0.11 g, 59%) as a colorless sticky liquid.

***R*$_f$** = 0.55 in 10% MeOH/DCM.

^{1}H NMR (500 MHz, MeOD) δ 6.96 – 6.84 (m, 1H), 6.19 (ddq, *J* = 15.5, 2.5, 1.4 Hz, 1H), 3.89 (s, 1H), 3.66 (s, 3H), 3.52 – 3.42 (m, 3H), 3.41 – 3.33 (m, 3H), 3.11 – 3.02 (m, 2H), 2.44 – 2.31 (m, 4H), 2.26 (qd, *J* = 6.8, 3.2 Hz, 2H), 1.84–1.71 (m, 2H), 0.92 (s, 6H); **^{13}C{^{1}H} NMR** (126 MHz, MeOD) δ 191.0, 176.0, 175.3, 173.9, 146.2, 130.1, 77.3, 70.4, 52.07, 40.4, 40.1, 36.4, 36.3, 34.0, 32.3, 29.0, 24.3, 21.3, 20.9.

IR (film, cm^{-1}): 3305, 3083, 2935, 2873, 1733, 1646, 1525, 1436, 1362, 1198, 1172, 1033, 972, 885, 805, 634.

HRMS (ESI^+) m/z: $[M+Na]^+$ calculated for $[C_{19}H_{32}N_2NaO_7S]^+$= 455.1822, found 455.1837, Δ = 2.93 ppm.

$[\alpha]_D^{20}$ = +21.1 (c= 0.29 g/mL, MeOH)

3.3.10 Synthesis of (*R*)-*S*-(2-(3-(2,4-dihydroxy-3,3-dimethylbutanamido)propanamido)ethyl) (*E*)-5-(benzyloxy)pent-2-enethioate (**4i**), OJJ-133.

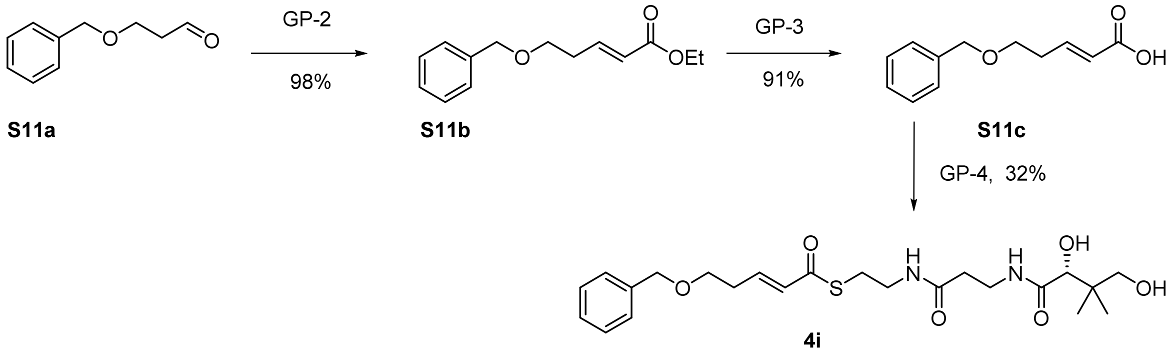

Scheme S11. Synthesis of **4i** from **S11a**

3.3.10.1 Ethyl (*E*)-5-(benzyloxy)pent-2-enoate (**S11b**), OJJ-128.

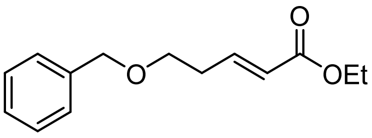

S11b

The Wittig product **S11b** was prepared from commercially available aldehyde **S11a** (0.35 g, 2.13 mmol) following GP-2 providing **S11b** (0.49 g, 98%) as a colorless oil.

R_f = 0.5 in 10% EtOAc/Hex.

^{1}H NMR (300 MHz, $CDCl_3$) δ 7.34 – 7.15 (m, 5H), 6.91 (dt, *J* = 15.7, 6.9 Hz, 1H), 5.83 (dt, *J* = 15.7, 1.6 Hz, 1H), 4.45 (s, 2H), 4.12 (q, *J* = 7.1 Hz, 2H), 3.51 (t, *J* = 6.5 Hz, 2H), 2.44 (qd, *J* = 6.6, 1.6 Hz, 2H), 1.22 (t, *J* = 7.1 Hz, 3H); **^{13}C{^{1}H} NMR** (75 MHz, $CDCl_3$) δ 166.5, 145.6, 138.2, 128.5, 127.8, 123.1, 73.2, 68.4, 60.3, 32.7, 14.4.

IR (film, cm^{-1}): 3030, 2980, 2936, 2858, 1714, 1654, 1495, 1453, 1390, 1365, 1310, 1264, 1213, 1173, 1094, 1041, 977, 857, 807, 736, 697, 610.

HRMS (ESI^+) m/z: $[M+Na]^+$ calculated for $[C_{14}H_{18}NaO_3]^+$= 257.1148, found 257.1144, Δ = −2.15 ppm.

3.3.10.2 (*E*)-5-(benzyloxy)pent-2-enoic acid (**S11c**), OJJ-131.

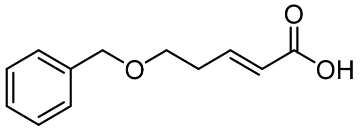

S11c

The α,β-unsaturated acid (**S11c**) was prepared from corresponding ester **S11b** following GP-3 and this intermediate was used in the next step without further purification.

3.3.10.3 (*R*)-*S*-(2-(3-(2,4-dihydroxy-3,3-dimethylbutanamido)propanamido)ethyl) (*E*)-5-(benzyloxy)pent-2-enethioate (**4i**), OJJ-133.

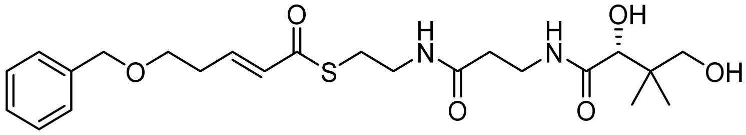

4i

The α,β-unsaturated thioester **4i** was prepared via coupling reaction from corresponding α,β-unsaturated acid **S11c** (0.15 g, 0.73 mmol) following GP-4 providing **4i** (0.11 g, 32%) as a colorless sticky oil.

$\boldsymbol{R}_\mathrm{f}$ = 0.6 in 10% MeOH/DCM.

^{1}H NMR (500 MHz, MeOD) δ 7.35 – 7.24 (m, 5H), 6.94 (dt, *J* = 15.6, 6.9 Hz, 1H), 6.24 (dt, *J* = 15.5, 1.6 Hz, 1H), 4.51 (s, 2H), 3.88 (s, 1H), 3.61 (t, *J* = 6.2 Hz, 2H), 3.51 – 3.43 (m, 3H), 3.41 – 3.36 (m, 2H), 3.34 (m, 1H), 3.07 (t, *J* = 6.7 Hz, 2H), 2.50 (qd, *J* = 6.2, 1.6 Hz, 2H), 2.40 (t, *J* = 6.7 Hz, 2H), 0.91 (s, 6H); **^{13}C{^{1}H} NMR** (126 MHz, MeOD) δ 191.0, 176.1, 173.9, 144.3, 139.6, 130.9, 129.4, 129.0,128.9, 128.7, 77.3, 73.9, 70.3, 69.3, 40.4, 40.2, 36.4, 36.3, 33.5, 28.9, 21.3, 20.9.

IR (film, cm^{-1}): 3305, 3086, 2932, 2870, 1644, 1525, 1452, 1408, 1361, 1288, 1201, 1153, 1075, 1026, 910, 886, 802, 737, 697, 609.

HRMS (ESI^{+}) m/z: [M+Na]$^{+}$ calculated for [$C_{23}H_{34}N_2NaO_6S$]$^{+}$= 489.2030, found 489.2042, Δ = 2.13 ppm.

$[\alpha]_D^{20}$ = +18.3 (c= 0.4 g/mL, MeOH)

3.3.11 Synthesis of (*R*)-*S*-(2-(3-(2,4-dihydroxy-3,3-dimethylbutanamido)propanamido)ethyl) (*E*)-7-((tert-butyldimethylsilyl)oxy)hept-2-enethioate (**4j**), OJJ-96.

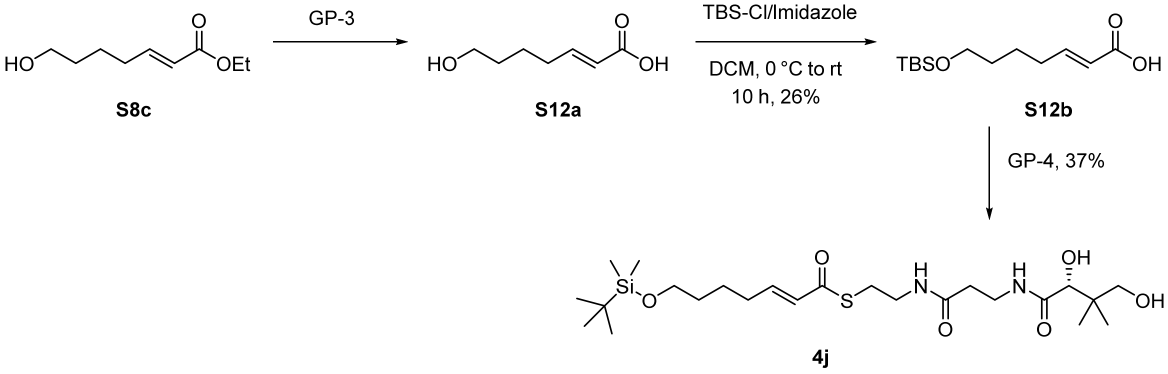

Scheme S12. Synthesis of **4j** from **S8c**

3.3.11.1 (*E*)-7-hydroxyhept-2-enoic acid (**S12a**), OJJ-85.

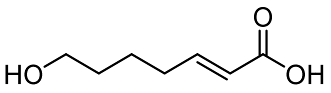

S12a

The hydroxy α,β-unsaturated acid **S12a** was synthesized from corresponding ester **S8c** (0.3 g, 1.74 mmol) following GP-3 and this intermediate was used in the next step without further purification.

3.3.11.2 (*E*)-7-((tert-butyldimethylsilyl)oxy)hept-2-enoic acid (**S12b**), OJJ-86.

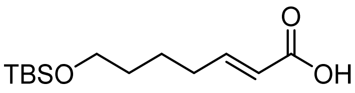

S12b

To the crude rection mixture of **S12a** (0.26 g) in 10 mL dry DCM at 0 °C was added successively TBSCl (0.57 g, 2.1 equiv.) and imidazole (0.26 g, 2.1 equiv.) under nitrogen atmosphere and the reaction mixture was allowed to acclimate at rt slowly. After 10 h, the solvent was evaporated, and the crude mixture was purified using combiFlash by passing 15-20% EtOAc/hex providing the desired **S12b** (0.12 g, 26 %) as a colorless oil.

^{1}H NMR (300 MHz, $CDCl_3$) δ 7.08 (dt, *J* = 15.6, 6.9 Hz, 1H), 5.90 – 5.77 (m, 1H), 3.67 – 3.55 (m, 2H), 2.32 – 2.18 (m, 2H), 1.54 (p, *J* = 3.4 Hz, 4H), 0.89 (s, 9H), 0.05 (s, 6H). **^{13}C NMR** (75 MHz, CDCl3) δ 171.4, 152.3, 120.8, 62.9, 32.7, 32.2, 26.1, 24.5, 18.5, -5.2.
IR (film, cm^{-1}): 2928, 2886, 2856, 1695, 1649, 1771, 1462, 1418, 1388, 1360, 1282, 1253, 1168, 1096, 1004, 982, 937, 833, 773, 686, 661.

3.3.11.3 (*R*)-*S*-(2-(3-(2,4-dihydroxy-3,3-dimethylbutanamido)propanamido)ethyl) (*E*)-7-((tert-butyldimethylsilyl)oxy)hept-2-enethioate (**4j**), OJJ-96.

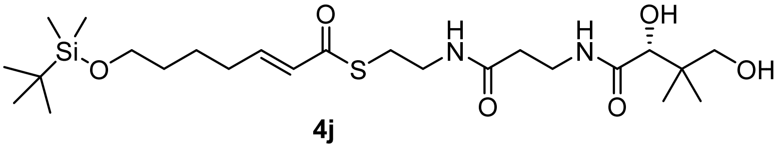

4j

The α,β-unsaturated thioester **4j** was prepared via coupling reaction from corresponding α,β-unsaturated acid **S12b** (0.09 g, 0.36 mmol) following GP-4 providing **4j** (0.07 g, 37%) as a colorless sticky oil.

***R*f** = 0.65 in 10% MeOH/DCM.

^{1}H NMR (500 MHz, MeOD) δ 6.98 – 6.88 (dt, *J* = 15.6, 6.9 Hz, 1H), 6.18 (dt, *J* = 15.6, 1.5 Hz, 1H), 3.89 (s, 1H), 3.66 (ddd, *J* = 5.8, 4.4, 2.0 Hz, 2H), 3.53 – 3.44 (m, 3H), 3.43 – 3.34 (m, 3H), 3.07 (t, *J* = 6.7 Hz, 2H), 2.41 (t, *J* = 6.7 Hz, 2H), 2.29 – 2.21 (m, 2H), 1.55 (p, *J* = 3.3 Hz, 4H), 0.92 (d, *J* = 1.7 Hz, 6H), 0.91 (s, 9H), 0.06 (s, 6H); **^{13}C{^{1}H} NMR** (126 MHz, MeOD) δ 191.0, 176.0, 173.9, 147.3, 129.6, 77.3, 70.4, 63.8, 40.4, 40.2, 36.4, 36.33, 33.2, 32.8, 28.9, 26.4, 25.5, 21.3, 21.0, 19.1, -5.2.

IR (film, cm^{-1}): 3303, 3084, 2929, 2857, 1634, 1528, 1471, 1461, 1435, 1386, 1360, 1293, 1252, 1186, 1147, 1095, 1040, 977, 939, 885, 833, 812, 714, 709, 659.

HRMS (ESI^+) m/z: $[M+Na]^+$ calculated for $[C_{24}H_{46}N_2NaO_6SSi]^+$= 541.2738, found 541.2755, Δ=2.79 ppm.

$[\alpha]_D^{20}$ = +17.6 (c= 0.33 g/mL, MeOH)

3.3.12 Synthesis of (*R*)-*S*-(2-(3-(2,4-dihydroxy-3,3-dimethylbutanamido)propanamido)ethyl) (*E*)-5-phenylpent-2-enethioate (**4k**), OJJ-102.

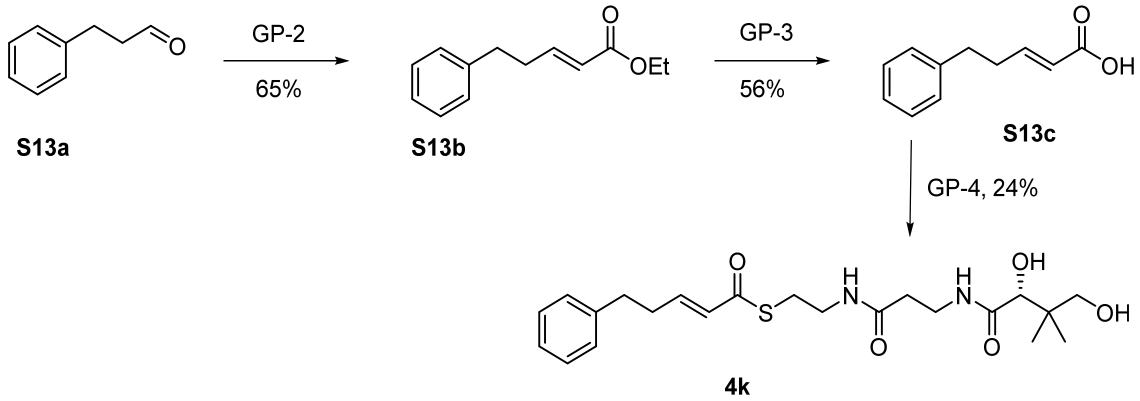

Scheme S13. Synthesis of **4k** from **S13a**

3.3.12.1 Ethyl (*E*)-5-phenylpent-2-enoate (**S13b**), OJJ-98.

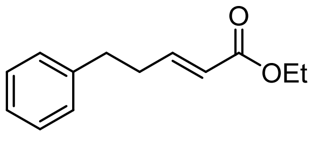

S13b

The Wittig product **S13b** was prepared from commercially available aldehyde **S13a** (0.4 g, 2.98 mmol) following GP-2 providing **S13b** (0.4 g, 65%) as a colorless liquid.

R_f = 0.6 in 10% EtOAc/Hex.

^{1}H NMR (300 MHz, $CDCl_3$) δ 7.34 – 7.25 (m, 2H), 7.25 – 7.16 (m, 3H), 7.02 (dt, *J* = 15.7, 6.8 Hz, 1H), 5.86 (dt, *J* = 15.6, 1.6 Hz, 1H), 4.20 (q, *J* = 7.1 Hz, 2H), 2.79 (dd, *J* = 8.9, 6.6 Hz, 2H), 2.59 – 2.46 (m, 2H), 1.29 (t, *J* = 7.1 Hz, 3H); **^{13}C NMR** (75 MHz, $CDCl_3$) δ 166.6, 148.1, 140.9, 128.6, 128.4, 126.3, 122.0, 60.3, 34.5, 34.0, 14.4.

IR (film, cm^{-1}): 3026, 2980, 2933, 2858, 1715, 1652, 1603, 1496, 1453, 1390, 1366, 1313, 1264, 1193, 1177, 1147, 1087, 1034, 973, 906, 852, 810, 747, 686, 589, 567, 495.

HRMS (ESI$^+$) m/z: $[M+H]^+$ calculated for $[C_{13}H_{17}O_2]^+$= 205.1223, found 205.1224, Δ=0.03 ppm.

3.3.12.2 (*E*)-5-phenylpent-2-enoic acid (**S13c**), OJJ-100.

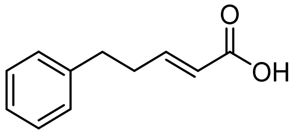

S13c

The α,β-unsaturated acid **S13c** was prepared from corresponding ester **S13b** (0.3 g, 1.47 mmol) according to GP-3 providing **S13c** (0.15 g) in 56% crude yield as a colorless liquid and this intermediate was used in the next step without further purification.

3.3.12.3 (*R*)-*S*-(2-(3-(2,4-dihydroxy-3,3-dimethylbutanamido)propanamido)ethyl) (*E*)-5-phenylpent-2-enethioate (**4k**), OJJ-102.

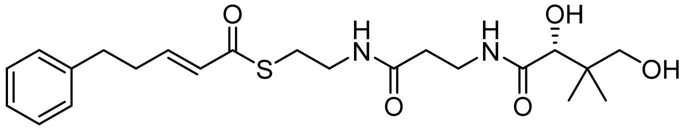

4k

The α,β-unsaturated thioester **4k** was prepared via coupling reaction from corresponding α,β-unsaturated acid **S13c** (0.15 g, 0.85 mmol) following GP-4 providing **4k** (0.09 g, 24%) as a colorless sticky oil.

$\boldsymbol{R}_\mathbf{f}$ = 0.6 in 10% MeOH/DCM.

^{1}H NMR (500 MHz, MeOD) δ 7.26 (dd, *J* = 8.2, 6.9 Hz, 2H), 7.21 – 7.13 (m, 3H), 6.93 (dt, *J* = 15.6, 6.9 Hz, 1H), 6.15 (dt, *J* = 15.6, 1.6 Hz, 1H), 3.90 (s, 1H), 3.53 – 3.43 (m, 3H), 3.42 – 3.33 (m, 3H), 3.05 (t, *J* = 6.7 Hz, 2H), 2.76 (t, *J* = 7.6 Hz, 2H), 2.51 (dtd, *J* = 8.8, 7.0, 1.6 Hz, 2H), 2.40 (t, *J* = 6.7 Hz, 2H), 0.93 (s, 6H); **^{13}C{^{1}H} NMR** (126 MHz, MeOD) δ 191.0, 175.9, 173.8, 146.4, 142.0, 129.9, 129.4, 129.4, 127.1, 77.3, 70.4, 40.3, 40.1, 36.4, 36.3, 35.2, 34.9, 28.9, 21.3, 21.0.

IR (film, cm^{-1}): 3304, 3084, 3026, 2930, 2873, 1644, 1632, 1525, 1497, 1452, 1434, 1362, 1290, 1242, 1196, 1135, 1072, 1031, 980, 907, 886, 801, 747, 698, 639, 583, 494.

HRMS (ESI$^+$) m/z: [M+Na]$^+$ calculated for [$C_{22}H_{32}N_2NaO_5S$]$^+$= 459.1924, found 459.1938, Δ=2.73 ppm.

$[\alpha]_D^{20}$ = +11.3 (c= 0.35 g/mL, MeOH)

3.4 ECH-catalyzed hydration reactions and characterization of products

3.4.1 General procedure for ECH catalyzed hydration reaction (GP-5)

To a stirred suspension of **4** (0.0534 mmol, ~20-30 mg) in 40 mL buffer (50 mM Tris, 50 mM KCl, pH = 7.4) at rt was added successively a stock solution of ECH (108 µL of 14.9 mg/mL solution in 100 mM potassium phosphate buffer, pH 7.2, 3 mM EDTA) and 80 µL of a solution of **5** (as the disodium salt in 50 mM Tris buffer, pH 7.5). After 2 h, the mixture was extracted with $CHCl_3$:i-PrOH (7: 3, v/v, 5 × 80 mL). The combined extracts were dried (Na_2SO_4) and concentrated, and the residue was purified by CombiFlash (gradient of 3.5 to 6 % MeOH/DCM) to obtain recovered **4** and the desired product **6**.

3.4.2 Synthesis of *S*-(2-(3-((*R*)-2,4-dihydroxy-3,3-dimethylbutanamido)propanamido)ethyl) (*S*)-3-hydroxyhexanethioate (**6a**), OJJ-142.

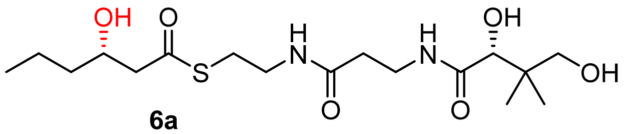

6a

The title compound (**6a**) was synthesized via enzyme catalyzed hydration reaction according to the GP-5 from the corresponding α,β-unsaturated pantetheine thioester **4a** (0.0534 mmol, 20 mg) providing **6a** (13.6 mg, 64%) as a colorless sticky oil along with recovery of **4a** (4.0 mg, 20%).

***R*$_f$** = 0.5 in 10% MeOH/DCM.

^{1}H NMR (500 MHz, MeOD) δ 4.08 – 3.99 (m, 1H), 3.89 (s, 1H), 3.53 – 3.42 (m, 3H), 3.42 – 3.32 (m, 3H), 3.02 (td, *J* = 6.6, 1.2 Hz, 2H), 2.75 – 2.64 (m, 2H), 2.41 (t, *J* = 6.7 Hz, 2H), 1.53 – 1.43 (m, 3H), 1.41 – 1.33 (m, 1H), 0.98 – 0.92 (t, *J* = 6.7 Hz ,3H), 0.92 (s, 6H); **^{13}C{^{1}H} NMR** (126 MHz, MeOD) δ 198.9, 176.1, 173.9, 77.3, 70.4, 69.3, 52.6, 40.4, 40.3, 40.0, 36.4, 36.3, 29.3, 21.3, 20.9, 19.7, 14.3.

IR (film, cm^{-1}): 3294, 2958, 2932, 2873, 1639, 1526, 1435, 1363, 1291, 1243, 1199, 1075, 1039, 596.

HRMS (ESI^+) m/z: $[M+H]^+$ calculated for $[C_{17}H_{33}N_2O_6S]^+$= 393.2054, found 393.2060, Δ=1.53 ppm.

$[\alpha]_D^{20}$ = +23.6 (c= 0.67 g/mL, MeOH).

The stereoisomeric ratio was determined by HPLC (Chiralpak IB, 1.0 mL/min, 93:7 hexanes:IPA, λ = 230 nm, t_r = 31.8 min (minor, (3*R*)-isomer), t_r =37.0 min (major, (3*S*)-isomer), >99:<1 dr.

3.4.3 Synthesis of *S*-(2-(3-((*R*)-2,4-dihydroxy-3,3-dimethylbutanamido)propanamido)ethyl) (*S*)-3-hydroxydecanethioate (**6b**), OJJ-143.

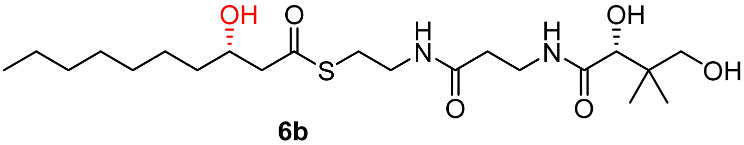

6b

The title compound (**6b**) was synthesized via enzyme catalyzed hydration reaction according to the GP-5 from the corresponding α,β-unsaturated pantetheine thioester **4b** (0.0534 mmol, 23 mg) providing **6b** (12.5 mg, 52%) as a colorless sticky oil along with recovered **4b** (6.2 mg, 27%).

***R*$_f$** = 0.55 in 10% MeOH/DCM.

^{1}H NMR (500 MHz, MeOD) δ 4.06 – 3.99 (m, 1H), 3.89 (s, 1H), 3.54 – 3.43 (m, 3H), 3.41 – 3.33 (m, 3H), 3.06 – 2.99 (m, 2H), 2.77 – 2.65 (m, 2H), 2.41 (t, *J* = 6.7 Hz, 2H), 1.46 (q, *J* = 5.4 Hz, 2H), 1.34–1.28 (m, 10H), 0.92 (s, 6H), 0.92–0.88 (t, 10.0 Hz, 3H); **$^{13}C\{^1H\}$ NMR** (126 MHz, MeOD) δ 198.9, 176.1, 173.9, 77.3, 70.4, 69.6, 52.6, 40.4, 40.0, 38.1, 36.4, 36.3, 33.0, 30.6, 30.4, 29.3, 26.6, 23.7, 21.3, 21.0, 14.4.

IR (film, cm^{-1}): 3306, 2925, 2856, 1639, 1531, 1461, 1361, 1294, 1033.

HRMS (ESI^+) m/z: $[M+H]^+$ calculated for $[C_{21}H_{41}N_2O_6S]^+$= 449.2680, found 449.2704, Δ=0.1 ppm.

$[\alpha]_D^{20}$ = +24.2 (c= 0.6 g/mL, MeOH)

The stereoisomeric ratio was determined by HPLC (Chiralpak IB, 1.0 mL/min, 93:7 hexanes:IPA, λ = 235 nm, t_r = 59.9 min (minor, (3*R*)-isomer), t_r =70.7 min (major, (3*S*)-isomer), >99:<1 dr.

3.4.4 Synthesis of *S*-(2-(3-((*R*)-2,4-dihydroxy-3,3-dimethylbutanamido)propanamido)ethyl) (*S*)-3-hydroxy-5-methylhexanethioate (**6c**), OJJ-173.

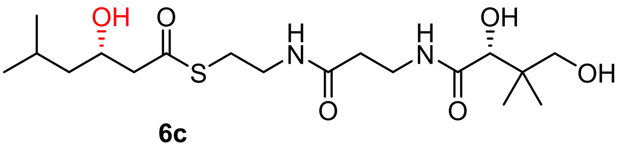

6c

The title compound (**6c**) was synthesized via enzyme catalyzed hydration reaction according to the GP-5 from the corresponding α,β-unsaturated pantetheine thioester **4c** (0.0534 mmol, 20.75 mg) providing **6c** (12.3 mg, 56%) as a colorless sticky oil along with recovery of **4c** (7.0 mg, 33%).

R_f = 0.5 in 10% MeOH/DCM.

^{1}H NMR (300 MHz, MeOD) δ 4.17 – 4.06 (m, 1H), 3.89 (s, 1H), 3.57 – 3.42 (m, 3H), 3.42 – 3.34 (m, 2H), 334-3.32 (m, 1H), 3.02 (t, *J* = 6.5 Hz, 2H), 2.68 (d, *J* = 6.3 Hz, 2H), 2.41 (t, *J* = 6.7 Hz, 2H), 1.87 – 1.70 (m, 1H), 1.42 (ddd, *J* = 14.2, 9.1, 5.3 Hz, 1H), 1.24 (ddd, *J* = 13.5, 8.7, 4.3 Hz, 1H), 0.94 (dd, *J* = 6.7, 0.8 Hz, 6H), 0.92 (s, 6H); **^{13}C{^{1}H} NMR** (75 MHz, MeOD) δ 198.9, 176.0, 173.9, 77.4, 70.4, 67.8, 53.1, 47.3, 40.4, 40.0, 36.4, 36.3, 29.3, 25.6, 23.7, 22.2, 21.3, 21.0.

IR (film, cm^{-1}): 3304, 2954, 2871, 1642, 1528, 1466, 1437, 1385, 1365, 1292, 1253, 1199, 1076, 1033, 978.

HRMS (ESI$^+$) m/z: [M+H]$^+$ calculated for $[C_{18}H_{35}N_2O_6S]^+$= 407.2210, found 407.2213, Δ=0.46 ppm.

$[\alpha]_D^{20}$ = +25.1 (c= 0.55 g/mL, MeOH).

The stereoisomeric ratio was determined by HPLC (Chiralpak IB, 1.0 mL/min, 92:8 hexanes:IPA, λ = 235 nm, t_r = 41.4 min (minor, (3*R*)-isomer), t_r =46.2 min (major, (3*S*)-isomer), >99:<1 dr.

3.4.5 Synthesis of *S*-(2-(3-((*R*)-2,4-dihydroxy-3,3-dimethylbutanamido)propanamido)ethyl) (*R*)-3-cyclohexyl-3-hydroxypropanethioate (**6d**), OJJ-172.

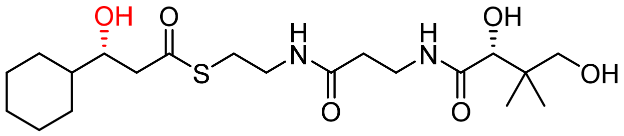

6d

The title compound (**6d**) was synthesized via enzyme catalyzed hydration reaction according to the GP-5 from the corresponding α,β-unsaturated pantetheine thioester **4d** (0.0534 mmol, 22.14 mg) providing **6d** (12.0 mg, 52%) as a colorless sticky oil along with recovery of **4d** (9.2 mg, 41%).

$\boldsymbol{R_f}$ = 0.45 in 10% MeOH/DCM.

^{1}H NMR (300 MHz, MeOD) δ 3.89 (s, 1H), 3.86-3.77 (m, 1H), 3.53 – 3.43 (m, 3H), 3.42-3.35 (m, 2H), 3.35-3.32 (m, 1H), 3.02 (t, *J* = 6.4 Hz, 2H), 2.81 – 2.60 (m, 2H), 2.41 (t, *J* = 6.7 Hz, 2H), 1.90–1.60 (m, 5H), 1.42–1.17 (m, 4H), 1.16–0.99 (m, 2H), 0.92 (s, 6H); **^{13}C{^{1}H} NMR** (75 MHz, MeOD) δ 199.5, 176.0, 173.9, 77.4, 73.8, 70.4, 45.0, 40.4, 40.0, 36.4, 36.3, 30.2, 29.3, 29.1, 27.6, 27.3, 27.2, 21.3, 21.0.

IR (film, cm^{-1}): 3294, 2923, 2853, 1644, 1527, 1446, 1409, 1362, 1291, 1032, 618, 581.

HRMS (ESI^{+}) m/z: [M+H]$^{+}$ calculated for [$C_{20}H_{37}N_2O_6S$]$^{+}$= 433.2367, found 433.2370, Δ=0.66 ppm.

$[\alpha]_D^{20}$ = +39.8 (c= 0.53 g/mL, MeOH)

The stereoisomeric ratio was determined by HPLC (Chiralpak IB, 1.0 mL/min, 88:12 hexanes:IPA, λ = 235 nm, t_r = 26.6 min (minor, (3*S*)-isomer), t_r =30.0 min (major, (3*R*)-isomer), >99:<1 dr.

3.4.6 Synthesis of *tert*-butyl 4-((*R*)-3-((2-(3-((*R*)-2,4-dihydroxy-3,3-dimethylbutanamido)propanamido)ethyl)thio)-1-hydroxy-3-oxopropyl)piperidine-1-carboxylate (**6e**), OJJ-144.

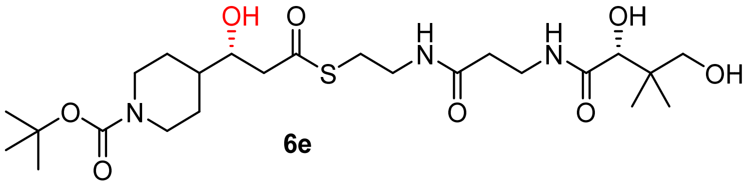

6e

The title compound (**6e**) was synthesized via enzyme catalyzed hydration reaction according to the GP-5 from the corresponding α,β-unsaturated pantetheine thioester **4e** (0.0534 mmol, 27.54 mg) providing **6e** (10.2 mg, 36%) as a colorless sticky oil along with recovery of **4e** (13.4 mg, 49%).

$\boldsymbol{R_f}$ = 0.5 in 10% MeOH/DCM.

^{1}**H NMR** (500 MHz, MeOD) δ 4.16 – 4.06 (m, 2H), 3.89 (s, 1H), 3.89 – 3.82 (m, 1H), 3.53 – 3.43 (m, 3H), 3.42 – 3.36 (m, 2H), 3.36-3.33 (m, 1H), 3.03 (td, *J* = 6.6, 1.7 Hz, 2H), 2.80-2.67 (m, 4H), 2.41 (t, *J* = 6.7 Hz, 2H), 1.79 (dt, *J* = 13.4, 2.8 Hz, 1H), 1.60 (dt, *J* = 12.8, 2.9 Hz, 1H), 1.53 (ddq, *J* = 12.1, 6.6, 2.9 Hz, 1H), 1.45 (s, 9H), 1.26–1.15 (m, 2H), 0.92 (s, 6H); ^{13}**C{^{1}H} NMR** (126 MHz, MeOD) δ 199.1, 176.1, 173.9, 156.5, 80.9, 77.3, 72.8, 70.4, 44.9, 43.1, 40.4, 40.0, 36.4, 36.3, 29.5, 29.4, 28.7, 28.2, 21.3, 21.0.

IR (film, cm^{-1}): 3293, 2935, 2872, 1638, 1526, 1472, 1431, 1365, 1280, 1239, 1196, 1163, 1044, 1033, 1018.

HRMS (ESI^{+}) m/z: [M+H]$^{+}$ calculated for $[C_{24}H_{44}N_3O_8S]^{+}$= 534.2844, found 534.2864, Δ=1.91 ppm.

$[\alpha]_D^{20}$ = +27.0 (c= 0.47 g/mL, MeOH).

The stereoisomeric ratio was determined by HPLC (Chiralpak IB, 1.0 mL/min, 89:11 hexanes:IPA, λ = 235 nm, t_r = 61.8 min (minor, (3*S*)-isomer), t_r =71.9 min (major, (3*R*)-isomer), >99:<1 dr.

3.4.7 Synthesis of *S*-(2-(3-((*R*)-2,4-dihydroxy-3,3-dimethylbutanamido)propanamido)ethyl) (*S*)-7-bromo-3-hydroxyheptanethioate (**6f**), OJJ-146.

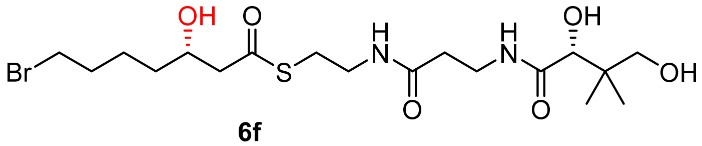

6f

The title compound (**6f**) was synthesized via enzyme catalyzed hydration reaction according to the GP-5 from the corresponding α,β-unsaturated pantetheine thioester **4f** (0.0534 mmol, 25.0 mg) providing **6f** (19.0 mg, 73%) as a colorless sticky oil along with recovery of **4f** (4.0 mg, 16%).

R_f = 0.5 in 10% MeOH/DCM.

^{1}H NMR (500 MHz, MeOD) δ 8.19 (br m, 1H), δ 7.96 (br m, 1H), δ 4.06-4.01 (m, 1H), 3.89 (s, 1H), 3.53 – 3.43 (m, 5H), 3.42 – 3.35 (m, 2H), 3.35 – 3.32 (m, 1H), 3.02 (td, *J* = 6.7, 1.4 Hz, 2H), 2.74 – 2.66 (m, 2H), 2.41 (t, *J* = 6.7 Hz, 2H), 1.93–1.80 (m, 2H), 1.65-1.57 (m, 1H), 1.55–1.43 (m, 3H), 0.92 (s, 6H); **^{13}C{^{1}H} NMR** (126 MHz, MeOD) δ 198.8, 176.1, 173.9, 77.3, 70.3, 69.3, 52.6, 40.4, 40.0, 37.1, 36.4, 36.3, 34.2, 33.8, 29.3, 25.3, 21.3, 20.9.

IR (film, cm^{-1}): 3295, 2933, 2872, 1641, 1526, 1436, 1362, 1290, 1244, 1199, 1075, 1026, 737, 587.

HRMS (ESI^+) m/z: $[M+H]^+$ calculated for $[C_{18}H_{34}BrN_2O_6S]^+$= 485.1315, found 485,1322, Δ=0.55 ppm.

$[\alpha]_D^{20}$ = +24.5 (c= 1.0 g/mL, MeOH).

The stereoisomeric ratio was determined by HPLC (Chiralpak IC, 1.0 mL/min, 75:25 hexanes:IPA, λ = 230 nm, t_r = 39.2 min (minor, (3*R*)-isomer), t_r =47.3 min (major, (3*S*)-isomer), >98:<2 dr.

3.4.8 Synthesis of *S*-(2-(3-((*R*)-2,4-dihydroxy-3,3-dimethylbutanamido)propanamido)ethyl) (*S*)-6-(1,3-dioxolan-2-yl)-3-hydroxyhexanethioate (**6g**), OJJ-147.

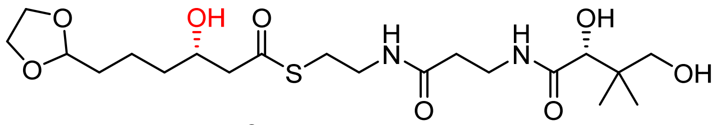

6g

The title compound (**6g**) was synthesized via enzyme catalyzed hydration reaction according to the GP-5 from the corresponding α,β-unsaturated pantetheine thioester **4g** (0.0534 mmol, 23.85 mg) providing **6g** (15.6 mg, 63%) as a colorless sticky oil along with recovery of **4g** (5.0 mg, 21%).

R_f = 0.5 in 10% MeOH/DCM.

^{1}H NMR (500 MHz, MeOD) δ 4.82 (t, *J* = 4.6 Hz, 1H), 4.07-3.99 (m, 1H), 3.97 – 3.91 (m, 2H), 3.89 (s, 1H), 3.85 – 3.80 (m, 2H), 3.53-3.44 (m, 3H), 3.43-3.35 (m, 2H), 3.34-3.32 (m, 1H), δ 3.02 (td, *J* = 6.6, 2.0 Hz, 2H), 2.70 (dd, *J* = 6.2, 1.7 Hz, 2H), 2.41 (t, *J* = 6.7 Hz, 2H), 1.70–1.55 (m, 3H), 1.54–1.42 (m, 3H), 0.92 (s, 6H); **^{13}C{^{1}H} NMR** (126 MHz, MeOD) δ 198.8, 176.1, 173.9, 105.5, 77.3, 70.3, 69.4, 65.8, 52.6, 40.4, 40.0, 37.9, 36.4, 36.3, 34.7, 29.3, 21.3, 21.1, 20.9.

IR (film, cm^{-1}): 3306, 2937, 2872, 1639, 1528, 1460, 1438, 1362, 1292, 1241, 1197, 1141, 1048, 1032.

HRMS (ESI$^+$) m/z: [M+H]$^+$ calculated for $[C_{20}H_{37}N_2O_8S]^+$= 465.2265, found 465.2266, Δ=0.17 ppm.

$[\alpha]_D^{20}$ = +24.2 (c= 0.93 g/mL, MeOH).

The stereoisomeric ratio was determined by HPLC (Chiralpak IC, 1.0 mL/min, 70:30 hexanes:IPA, λ = 230 nm, t_r = 81.6 min (minor, (3*R*)-isomer), t_r =90.6 min (major, (3*S*)-isomer), >99:<1 dr.

3.4.9 Synthesis of methyl (*S*)-7-((2-(3-((*R*)-2,4-dihydroxy-3,3-dimethylbutanamido)propanamido)ethyl)thio)-5-hydroxy-7-oxoheptanoate (**6h**), OJJ-148.

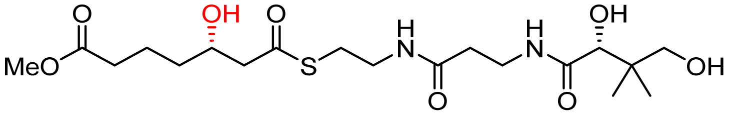

6h

The title compound (**6h**) was synthesized via enzyme catalyzed hydration reaction according to the GP-5 from the corresponding α,β-unsaturated pantetheine thioester **4h**

(0.0534 mmol, 23.1 mg) providing **6h** (15.5 mg, 65%) as a colorless sticky oil along with recovery of **4h** (6.0 mg, 26%).

R_f = 0.5 in 10% MeOH/DCM.

^{1}H NMR (500 MHz, MeOD) δ 4.07 – 4.00 (m, 1H), 3.89 (s, 1H), 3.66 (s, 3H), 3.54 – 3.43 (m, 3H), 3.41 – 3.35 (m, 2H), 3.34-3.32 (m, 1H), 3.05 – 2.99 (m, 2H), 2.73 – 2.68 (m, 2H), 2.43 – 2.39 (m, 2H), 2.38-2.32 (m, 2H), 1.83 – 1.71 (m, 1H), 1.70 – 1.60 (m, 1H), 1.55 – 1.43 (m, 2H), 0.92 (s, 6H); **^{13}C{^{1}H} NMR** (126 MHz, MeOD) δ 198.7, 176.0, 175.7, 173.9, 77.3, 70.4, 69.2, 52.5, 52.0, 40.4, 40.0, 37.3, 36.42 36.33 34.5, 29.3, 22.1, 21.3, 20.9.

IR (film, cm^{-1}): 3306, 2949, 2874, 1730, 1643, 1527, 1436, 1362, 1240, 1198, 1075, 1040, 1016, 746, 587.

HRMS (ESI$^+$) m/z: [M+H]$^+$ calculated for $[C_{19}H_{35}N_2O_8S]^+$= 451.2109, found 451.2113, Δ=0.64 ppm.

$[\alpha]_D^{20}$ = +21.6 (c= 1.07 g/mL, MeOH).

The stereoisomeric ratio was determined by HPLC (Chiralpak IC, 1.0 mL/min, 60:40 hexanes:IPA, λ = 230 nm, t_r = 47.0 min (minor, (3*R*)-isomer), t_r =73.9 min (major, (3*S*)-isomer), >98:<2 dr.

3.4.10 Synthesis of S-(2-(3-((*R*)-2,4-dihydroxy-3,3-dimethylbutanamido)propanamido)ethyl) (*S*)-5-(benzyloxy)-3-hydroxypentanethioate (6i), OJJ-149.

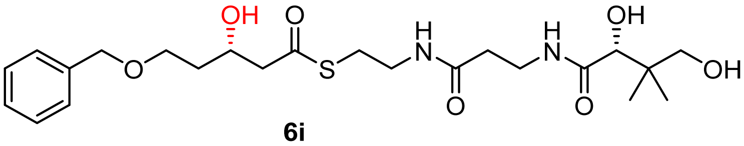

6i

The title compound (**6i**) was synthesized via enzyme catalyzed hydration reaction according to the GP-5 from the corresponding α,β-unsaturated pantetheine thioester **4i** (0.0534 mmol, 24.9 mg) providing **6i** (17.0 mg, 65%) as a colorless sticky oil along with recovery of **4i** (6.0 mg, 24%).

R_f = 0.55 in 10% MeOH/DCM.

^{1}H NMR (500 MHz, MeOD) δ 7.36 – 7.31 (m, 4H), 7.30 – 7.24 (m, 1H), 4.50 (s, 2H), 4.26-4.19 (m, 1H), 3.89 (s, 1H), 3.67 – 3.55 (m, 2H), 3.53 – 3.42 (m, 3H), 3.42 – 3.35 (m, 2H), 3.34-3.33 (m, 1H), 3.02 (td, *J* = 6.7, 1.8 Hz, 2H), 2.79 – 2.67 (m, 2H), 2.40 (t, *J* = 6.7 Hz, 2H), 1.85–1.77 (m, 1H), 1.76-1.68 (m, 1H), 0.92 (s, 6H). **^{13}C{^{1}H} NMR** (126 MHz, MeOD) δ 198.7, 176.0, 173.9, 139.7, 129.4, 128.9, 128.6, 77.3, 74.0, 70.3, 67.9, 67.1, 52.6, 40.4, 40.0, 37.9, 36.4, 36.3, 29.3, 21.3, 20.9.

IR (film, cm^{-1}): 3306, 2934, 2872, 1645, 1528, 1453, 1410, 1363, 1289, 1252, 1201, 1075, 1024, 738, 697, 607.

HRMS (ESI^{+}) m/z: [M+H]$^{+}$ calculated for $[C_{23}H_{37}N_2O_7S]^{+}$= 485.2316, found 485.2325, Δ=1.48 ppm.

$[\alpha]_D^{20}$ = +17.9 (c= 1.07 g/mL, MeOH).

The stereoisomeric ratio was determined by HPLC (Chiralpak IC, 1.0 mL/min, 77:23 hexanes:IPA, λ = 230 nm, t_r = 92.5 min (major, (3*S*)-isomer), t_r =110.3 min (minor, (3*R*)-isomer), >99:<1 dr.

3.4.11 Synthesis of S-(2-(3-((*R*)-2,4-dihydroxy-3,3-dimethylbutanamido)propanamido)ethyl) (*S*)-7-((*tert*-butyldimethylsilyl)oxy)-3-hydroxyheptanethioate (**6j**), OJJ-150.

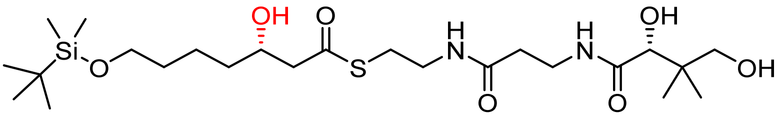

6j

The title compound (**6j**) was synthesized via enzyme catalyzed hydration reaction according to the GP-5 from the corresponding α,β-unsaturated pantetheine thioester **4j** (0.0534 mmol, 27.7 mg) providing **6j** (11.0 mg, 38%) as a colorless sticky oil along with recovery of **4j** (15.0 mg, 54%).

***R*$_f$** = 0.55 in 10% MeOH/DCM.

^{1}H NMR (500 MHz, MeOD) δ 4.07 – 3.99 (m, 1H), 3.89 (s, 1H), 3.65 (t, *J* = 6.0 Hz, 2H), 3.53 – 3.44 (m, 3H), 3.43 – 3.35 (m, 2H), 3.34 (m, 1H), 3.02 (t, *J* = 6.6 Hz, 2H), 2.70 (dd, *J*

= 6.4, 2.3 Hz, 2H), 2.41 (t, *J* = 6.7 Hz, 2H), 1.59–1.43 (m, 5H), 1.44 – 1.36 (m, 1H), 0.92 (s, 6H), 0.91 (s, 9H), 0.06 (s, 6H); **$^{13}C\{^{1}H\}$ NMR** (126 MHz, MeOD) δ 198.7, 176.1, 173.9, 77.3, 70.4, 69.5, 64.1, 52.6, 40.4, 40.0, 37.9, 36.4, 36.3, 33.7, 29.3, 26.4, 23.0, 21.3, 20.9, 19.2, -5.2.
IR (film, cm^{-1}): 3305, 2927, 2856, 1646, 1529, 1461, 1437, 1360, 1252, 1092, 1038, 834, 774, 660.

HRMS (ESI^+) m/z: $[M+H]^+$ calculated for $[C_{24}H_{49}N_2O_7SSi]^+$= 537.3024, found 537.3033, Δ=1.24 ppm.

$[\alpha]_D^{20}$ = +21.3 (c= 0.47 g/mL, MeOH).

The stereoisomeric ratio was determined by HPLC (Chiralpak IB, 1.0 mL/min, 93:7 hexanes:IPA, λ = 235 nm, t_r = 50.2 min (minor, (3*R*)-isomer), t_r = 60.4 min (major, (3*S*)-isomer), >99:<1 dr.

3.4.12 Synthesis of S-(2-(3-((*R*)-2,4-dihydroxy-3,3-dimethylbutanamido)propanamido)ethyl) (*S*)-3-hydroxy-5-phenylpentanethioate (**6k**), OJJ-171.

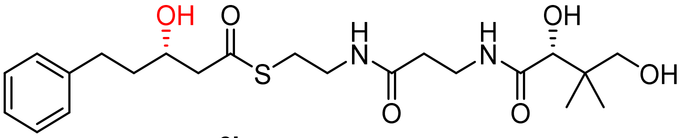

6k

The title compound (**6k**) was synthesized via enzyme catalyzed hydration reaction according to the GP-5 from the corresponding α,β-unsaturated pantetheine thioester **4k** (0.0534 mmol, 23.31 mg) providing **6k** (15.3 mg, 63%) as a colorless sticky oil along with recovery of **4k** (6.0 mg, 25%).

***R*f** = 0.55 in 10% MeOH/DCM.

^{1}H NMR (300 MHz, MeOD) δ 7.31 – 7.09 (m, 5H), 4.09-3.99 (m, 1H), 3.89 (s, 1H), 3.52 – 3.41 (m, 3H), 3.41 – 3.35 (m, 2H), 3.35-3.33 (m, 1H), 3.02 (t, *J* = 6.6 Hz, 2H), 2.85 – 2.57 (m, 4H), 2.40 (t, *J* = 6.6 Hz, 2H), 1.83-1.69 (m, 2H), 0.92 (s, 6H); **$^{13}C\{^{1}H\}$ NMR** (75 MHz, MeOD) δ 198.7, 176.0, 173.9, 143.2, 129.4, 129.4, 126.8, 77.4, 70.4, 69.0, 52.6, 40.4, 40.0, 40.0, 36.4, 36.3, 32.8, 29.3, 21.3, 21.0.

IR (film, cm^{-1}): 3295, 2935, 2872, 1641, 1526, 1453, 1437, 1362, 1291, 1240, 1199, 1047, 1033, 1017, 746, 699, 592.

HRMS (ESI^+) m/z: $[M+Na]^+$ calculated for $[C_{22}H_{34}N_2NaO_6S]^+$= 477.2030, found 477.2029, Δ= −0.44 ppm.

$[\alpha]_D^{20}$ = +14.7 (c= 0.7 g/mL, MeOH).

The stereoisomeric ratio was determined by HPLC (Chiralpak IC, 1.0 mL/min, 75:25 hexanes:IPA, λ = 230 nm, t_r = 44.1 min (minor, (3*R*)-isomer), t_r =54.3 min (major, (3*S*)-isomer), >98:<2 dr.

3.5 General procedures for the syntheses of (3*RS*)-3-hydroxyacyl-PAN thioesters

3.5.1 General procedure 6A (GP-6A): General procedure for the synthesis of (3*RS*)-hydroxy ester (**S14a**).

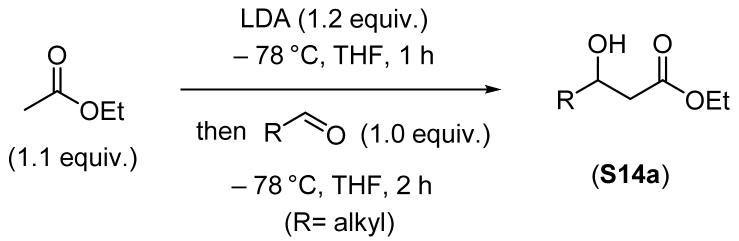

Scheme S14. Synthesis of (3*RS*)-hydroxy ester (**S14a**).

(3*RS*)-hydroxy esters (**S14a**) were synthesized following a literature report with slight modification of the reaction condition[36]. To a stirred solution of diisopropylamine (0.34 mL, 2.4 mmol) in 7 mL of dry THF under argon atmosphere at 0 °C was added dropwise solution of 2.5 M n-BuLi in hexane (1.0 mL, 2.4 mmol) over 2 min. The resulting pale-yellow solution was continued stirring for an additional 5 min at 0 °C before cooling to –78 °C. Freshly distilled ethyl acetate (0.2 mL, 2.2 mmol, 1.1 equiv.) dissolved in dry THF (2 mL) was added slowly via syringe over 2 min. After 1 h at –78 °C, a solution of aldehyde (2 mmol, 1.0 equiv.) in 10.0 mL dry THF was slowly introduced in the reaction mixture. After 2 h at −78 °C, the reaction mixture was allowed to acclimate at 0 °C and stirred for an additional 10 min before it was quenched with 20 mL of saturated aqueous NH_4Cl solution. The aqueous layer was extracted with EtOAc (25 mLx3), the

combined organic layer was dried (Na_2SO_4) and concentrated providing **S14a**. This intermediate **S14a** was used in the next step without further purification.

3.5.2 General procedure 6B (GP-6B): General procedure for the synthesis of (3*RS*)-hydroxy acid (**S15a**).

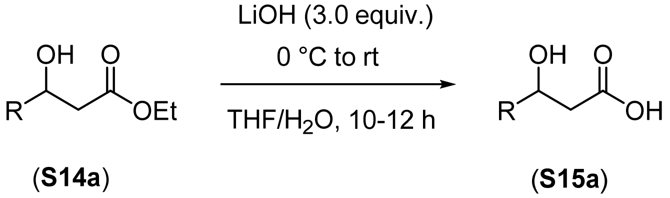

Scheme S15. Synthesis of (3*RS*)-hydroxy acid (**S15a**).

All (3*RS*)-hydroxy acids (**S15a**) were synthesized following GP-3.

3.5.3 General procedure 6C (GP-6C): General procedure for the synthesis of (*3RS*)-hydroxy PAN thioester (**6’**).

All (*3RS*)-hydroxy SPAN thioesters (**6’**) were synthesized following GP-4 with a modified reaction time (3 days) and purified using CombiFlash by a eluting with a gradient of 4.5 to 6% MeOH/DCM.

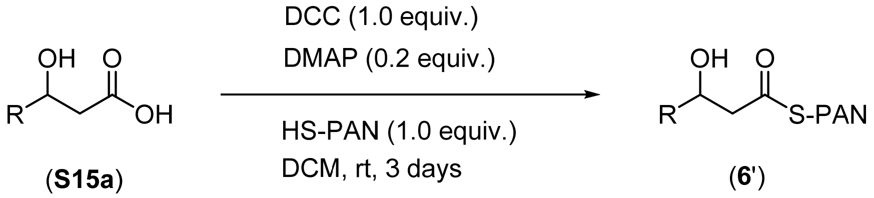

Scheme S16. Synthesis of (*3RS*)-hydroxy PAN thioester (**6’**).

3.5.4 General procedure 7A (GP-7A): General procedure for the synthesis of (3*RS*)-hydroxy-S-phenyl thioester (**S16a**).

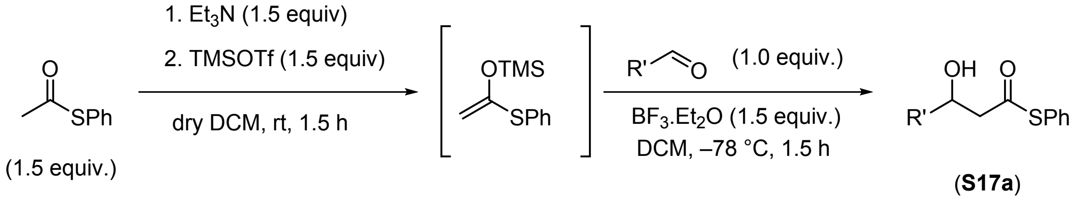

Scheme S17. Synthesis of (3*RS*)-hydroxy-S-phenyl thioester (**S17a**)

(3*RS*)-hydroxy-*S*-phenyl thioesters (**S17a**) were synthesized according to the literature reported procedure[37] with slight modification in reaction condition. To a stirred suspension of *S*-phenylthioacetate (1.5 mmol, 1.5 equiv.) in 7.0 mL of dry CH_2Cl_2 at rt was added successively triethylamine (1.5 mmol, 1.5 equiv.) and TMSOTf (1.5 mmol, 1.5 equiv.) under argon atmosphere. After 1.5 h, a solution of aldehyde (1.0 mmol, 1.0 equiv.) in dry CH_2Cl_2 (5.0 mL) and $BF_3.Et_2O$ (1.5 mmol, 1.5 equiv.) were introduced successively to the reaction mixture at −78 °C and stirring was continued for another 1-1.5 h depending upon the progress of the reaction (monitored by TLC). When almost all the aldehydes were consumed, the reaction was quenched with addition of aqueous saturated $NaHCO_3$ solution (10 mL) and the organic layer was extracted by DCM (3x15 mL). Combined organic layer was dried (Na_2SO_4), concentrated and purified using CombiFlash by passing 10-20% EtOAc/hexane providing the corresponding (3*RS*)-hydroxy-S-phenyl thioesters (**S17a**).

3.5.5 General procedure 7B (GP-7B): General procedure for the synthesis of (*3RS*)-hydroxy SPAN thioester (**6'**).

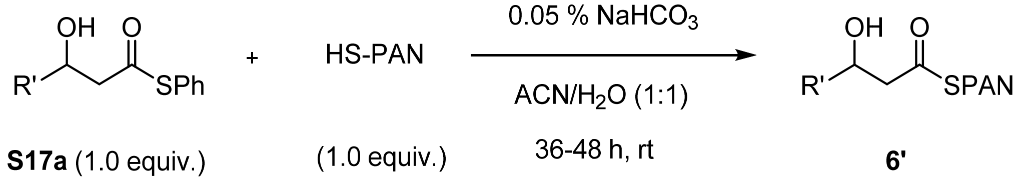

Scheme S18. Synthesis of (*3RS*)-hydroxy PAN thioester (**6'**)

To a degassed solution of hydroxy-thioester **S17a** (1.0 mmol, 1equiv.) and PAN monomer (1.0 mmol, 1equiv.) in 12.0 mL of ACN-H_2O (1:1, v/v) at rt was added solid $NaHCO_3$ (5 mol%, 0.05 equiv.) under argon atmosphere. After 36-48 h, when almost all the hydroxy-thioester was consumed (monitored by TLC), the reaction mixture was extracted with chloroform (3x15 mL). The combined organic layer was evaporated to dryness and purified using CombiFlash by passing 4.0-6% MeOH/DCM providing the corresponding (3*RS*)-hydroxy-SPAN thioesters (**6'**).

3.6 Syntheses of (3*RS*)-3-hydroxyacyl-PAN thioesters (6')

In general, the reference compounds, (3*RS*)-3-hydroxyacyl-PAN thioesters **6',** are spectroscopically indistinguishable from the stereoisomerically pure products **6** as they are mixtures of pseudoenantiomers. The identities of **6'a–6'k** were confirmed by comparison of the NMR spectra to those of pure **6a**–**6k**.

3.6.1 Synthesis of *S*-(2-(3-((*R*)-2,4-dihydroxy-3,3-dimethylbutanamido)propanamido)ethyl) 3-hydroxyhexanethioate (**6'a**), OJJ-108.

The title compound **6'a** was prepared following the reaction sequences of GP-6 as shown in Scheme S19.

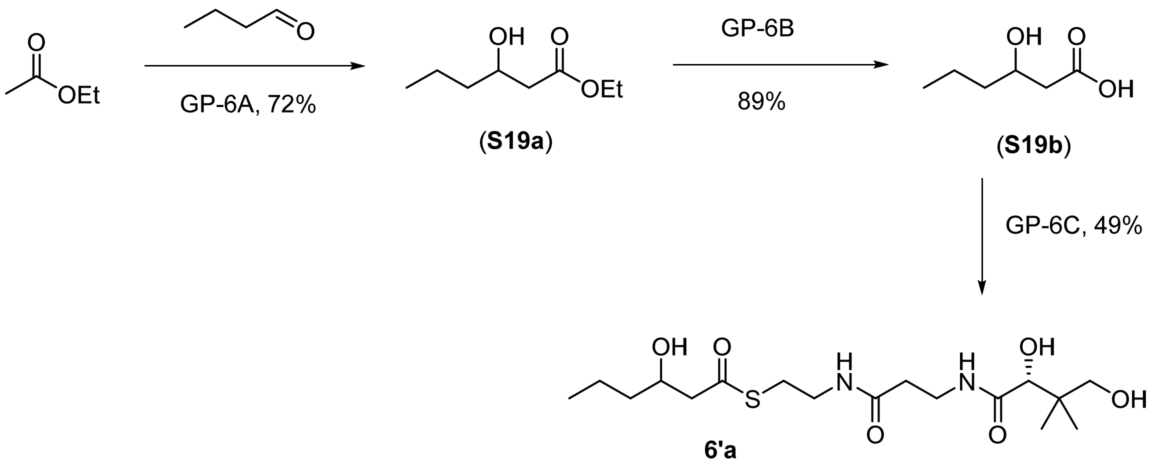

Scheme S19. Synthesis of **6'a** from butyraldehyde

3.6.1.1 Ethyl 3-hydroxyhexanoate (**S19a**), OJJ-60.

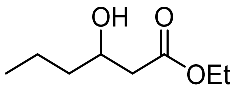

S19a

3-Hydroxy ester (**S19a**) was prepared from butyraldehyde (0.19 g, 2.6 mmol) following GP-6A providing **S19a** (0.3 g, 72%) and this intermediate was used in the next step without further purification.

3.6.1.2 3-Hydroxyhexanoic acid (**S19b**), OJJ-67.

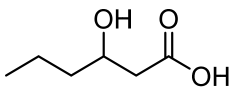

S19b

3-hydroxy acid (**S19b**) was prepared from corresponding ester **S19a** (0.3 g, 1.9 mmol) following GP-6B providing **S19b** (0.22 g, 89%), characterized by crude NMR and this intermediate was used in the next step without further purification. The spectral data matches with the literature reported data[38].

^{1}**H NMR** (500 MHz, $CDCl_3$) δ 4.05 (dddd, *J* = 8.9, 7.7, 4.5, 3.1 Hz, 1H), 2.56 (dd, *J* = 16.6, 3.1 Hz, 1H), 2.47 (dd, *J* = 16.5, 9.0 Hz, 1H), 1.59 – 1.51 (m, 1H), 1.46 (dddd, *J* = 15.4, 9.0, 4.6, 2.2 Hz, 2H), 1.39 (dddd, *J* = 17.1, 10.0, 4.5, 2.5 Hz, 1H), 0.94 (t, *J* = 7.0 Hz, 3H). ^{13}**C{^{1}H} NMR** (126 MHz, $CDCl_3$) δ 176.9, 68.1, 41.3, 38.6, 18.7, 13.9.

3.6.1.3 *S*-(2-(3-((*R*)-2,4-Dihydroxy-3,3-dimethylbutanamido)propanamido)ethyl)-3-hydroxyhexanethioate (**6'a**), OJJ-108.

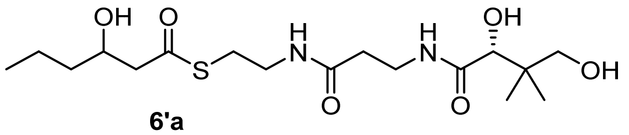

6'a

The (3*RS*)-hydroxy thioester **6'a** was prepared from corresponding 3-hydroxy acid **S19b** (0.14 g, 1.0 mmol) via coupling reaction following GP-6C providing **6'a** (0.2 g, 49%) as a colorless sticky liquid.

R_f = 0.5 in 10% MeOH/DCM.

^{1}**H NMR** (500 MHz, MeOD) δ 4.04 (p, *J* = 5.9 Hz, 1H), 3.89 (s, 1H), 3.56 – 3.31 (m, 6H), 3.02 (t, *J* = 6.6 Hz, 2H), 2.75 – 2.64 (m, 2H), 2.41 (t, *J* = 6.7 Hz, 2H), 1.53–1.42 (m, 3H), 1.41–1.32 (m, 1H), 0.95-0.92 (t, 3H), 0.92 (s, 6H); ^{13}**C{^{1}H} NMR** (126 MHz, MeOD) δ 198.8, 176.0, 173.9, 77.3, 70.3, 69.3, 52.6, 40.4, 40.3, 40.0, 36.4, 36.3, 29.3, 21.3, 20.9, 19.7, 14.2. The NMR data match with those obtained for **6a** (see Section 3.4.2).

3.6.2 Synthesis of S-(2-(3-((*R*)-2,4-dihydroxy-3,3-dimethylbutanamido)propanamido)ethyl) 3-hydroxydecanethioate (**6'b**), OJJ-157.

The title compound **6'b** was prepared following the reaction sequences of GP-6 as shown in Scheme S20.

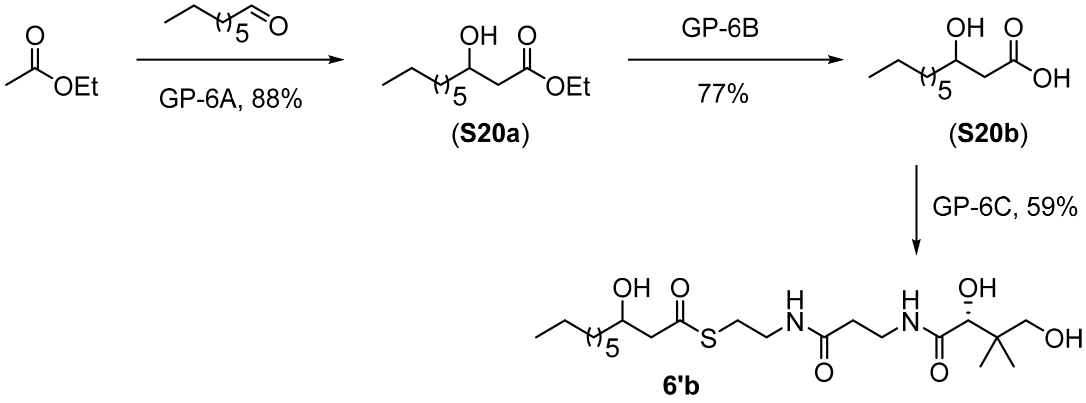

Scheme S20. Synthesis of **6'b** from octanal

3.6.2.1 Ethyl 3-hydroxydecanoate (**S20a**), OJJ-152.

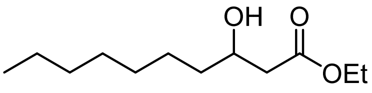

S20a

3-hydroxy ester (**S20a**) was prepared from octanal (0.4 g, 3.1 mmol) following GP-6A providing **S20a** (0.6 g, 88%) and this intermediate was used in the next step without further purification.

3.6.2.2 3-Hydroxydecanoic acid (**S20b**), OJJ-154.

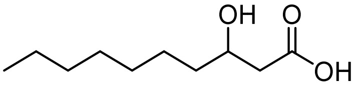

S20b

3-hydroxy acid (**S20b**) was prepared from corresponding ester **S20a** (0.4 g, 1.8 mmol) following GP-6B providing **S20b** (0.27 g, 77%), characterized by NMR and this intermediate was used in the next step without further purification. The spectral data matches with the literature reported data[38].

^{1}H NMR (500 MHz, $CDCl_3$) δ 4.03 (dddd, *J* = 9.0, 7.9, 4.7, 3.1 Hz, 1H), 2.58 (ddt, *J* = 16.6, 2.8, 1.3 Hz, 1H), 2.48 (ddt, *J* = 16.6, 9.0, 1.2 Hz, 1H), 1.59 – 1.51 (m, 1H), 1.46 (qd, *J* = 9.7, 3.9 Hz, 2H), 1.36–1.24 (m, 9H), 0.90–0.85 (m, 3H). **^{13}C{^{1}H} NMR** (126 MHz, $CDCl_3$) δ 178.0, 68.3, 41.2, 36.6, 31.9, 29.6, 29.3, 25.6, 22.8, 14.2.

3.6.2.3 S-(2-(3-((*R*)-2,4-dihydroxy-3,3-dimethylbutanamido)propanamido)ethyl) 3-hydroxydecanethioate (**6’b**), OJJ-157.

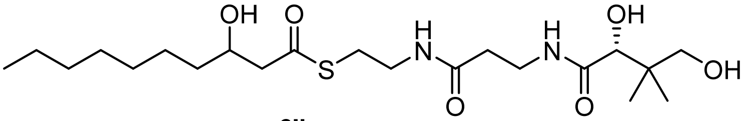

6'b

The (3*RS*)-hydroxy thioester **6’b** was prepared from corresponding 3-hydroxy acid **S20b** (0.113 g, 0.6 mmol) via coupling reaction following GP-6C providing **6’b** (0.16 g, 59%) as a colorless sticky liquid.

R_f = 0.55 in 10% MeOH/DCM.

^{1}H NMR (300 MHz, MeOD) δ 4.03 (q, *J* = 6.4 Hz, 1H), 3.89 (s, 1H), 3.56 – 3.32 (m, 6H), 3.02 (t, *J* = 6.6 Hz, 2H), 2.74 – 2.65 (m, 2H), 2.41 (t, *J* = 6.7 Hz, 2H), 1.46 (s, 3H), 1.31 (s, 9H), 0.92 (s, 6H), 0.92–0.88 (t, 6.0 Hz, 3H); **^{13}C{^{1}H} NMR** (75 MHz, MeOD) δ 198.9, 176.1, 173.9, 77.4, 70.4, 69.6, 52.6, 40.4, 40.0, 38.1, 36.4, 36.3, 33.0, 30.6, 30.4, 29.3, 26.6, 23.7, 21.3, 21.0, 14.4. The NMR data match with those obtained for **6b** (see Section 3.4.3).

3.6.3 *S*-(2-(3-((*R*)-2,4-dihydroxy-3,3-dimethylbutanamido)propanamido)ethyl) 3-hydroxy-5-methylhexanethioate (**6’c**), OJJ-194.

The title compound **6’c** was prepared following the reaction sequences of GP-6 as shown in Scheme S21.

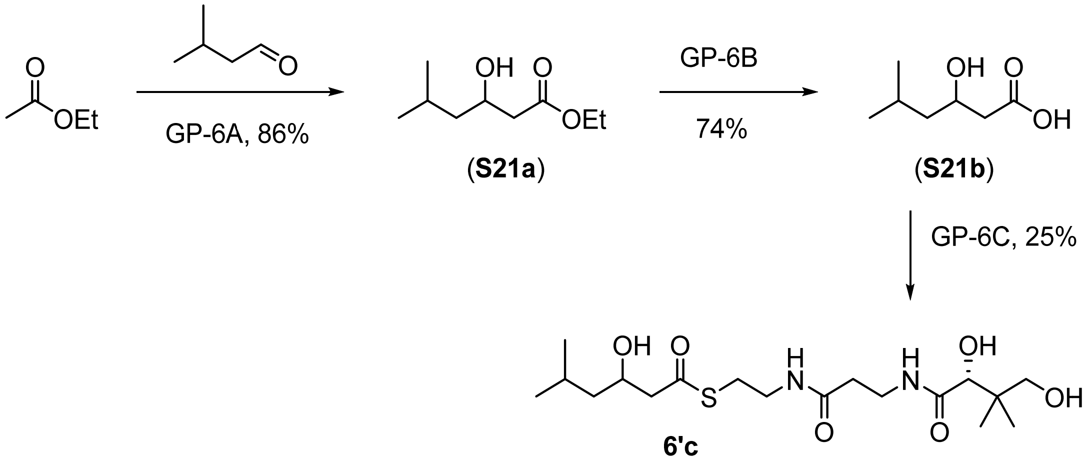

Scheme S21. Synthesis of **6’c** from isovaleraldehyde

3.6.3.1 ethyl 3-hydroxy-5-methylhexanoate (**S21a**), OJJ-175.

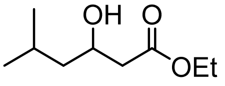

S21a

3-hydroxy ester (**S21a**) was prepared from isovaleraldehyde (0.26 g, 3.0 mmol) following GP-6A providing **S21a** (0.45 g, 86%) and this intermediate was used in the next step without further purification.

3.6.3.2 3-hydroxy-5-methylhexanoic acid (**S21b**), OJJ-191.

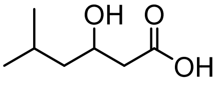

S21b

3-hydroxy acid (**S21b**) was prepared from corresponding ester **S21a** (0.45 g, 2.6 mmol) following GP-6B providing **S21b** (0.28 g, 74%) and this intermediate was used in the next step without further purification.

^{1}H NMR (300 MHz, $CDCl_3$) δ 4.12 (tt, *J* = 8.5, 4.2 Hz, 1H), 2.67 – 2.37 (m, 2H), 1.90 – 1.69 (m, 1H), 1.51 (ddd, *J* = 14.3, 8.9, 5.6 Hz, 1H), 1.23 (ddd, *J* = 13.3, 8.4, 4.4 Hz, 1H), 0.93 (d, *J* = 6.6 Hz, 6H). **^{13}C{^{1}H} NMR** (75 MHz, $CDCl_3$) δ 178.0, 66.4, 45.7, 41.7, 24.6, 23.3, 22.1.

IR (film, cm^{-1}): 2956, 2930, 2871, 1704, 1467, 1407, 1387, 1368, 1270, 1172, 1138, 1072, 1035, 987, 909, 880, 841, 733.

HRMS (ESI^+) m/z: $[M+Na]^+$ calculated for $[C_7H_{14}O_3Na]^+$= 169.0835, found 169.0823, Δ=−7.34 ppm.

3.6.3.3 S-(2-(3-((*R*)-2,4-dihydroxy-3,3-dimethylbutanamido)propanamido)ethyl) 3-hydroxy-5-methylhexanethioate (**6'c**), OJJ-194.

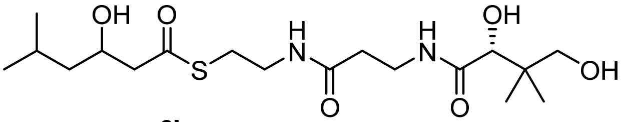

6'c

The (3*RS*)-hydroxy thioester **6'c** was prepared from corresponding 3-hydroxy acid **S21b** (0.113 g, 0.6 mmol) via coupling reaction following GP-6C providing **6'c** (0.113 g, 25%) as a colorless sticky liquid.

***R*$_f$** = 0.5 in 10% MeOH/DCM.

^{1}H NMR (500 MHz, MeOD) δ 4.12 (dtd, *J* = 10.3, 6.2, 3.0 Hz, 1H), 3.89 (s, 1H), 3.52 – 3.44 (m, 3H), 3.39 (q, *J* = 9.6 Hz, 2H), 3.34 (d, *J* = 6.8 Hz, 1H), 3.02 (t, *J* = 6.7 Hz, 2H), 2.68 (d, *J* = 6.3 Hz, 2H), 2.42 (dt, *J* = 13.4, 6.7 Hz, 2H), 1.78 (app nonet, *J* = 6.6 Hz, 1H), 1.42 (ddd, *J* = 14.2, 9.2, 5.2 Hz, 1H), 1.24 (ddd, *J* = 15.2, 8.8, 4.1 Hz, 1H), 0.92 (q, *J* = 3.5 Hz, 12H). **^{13}C{^{1}H} NMR** (126 MHz, MeOD) δ 198.8, 176.0, 173.9, 77.2, 70.3, 67.7, 53.1, 47.3, 40.4, 40.0, 36.4, 36.3, 29.3, 25.6, 23.7, 22.2, 21.3, 20.9. The NMR data match with those obtained for **6c** (see Section 3.4.4).

3.6.4 Synthesis S-(2-(3-((*R*)-2,4-dihydroxy-3,3-dimethylbutanamido)propanamido)ethyl) 3-cyclohexyl-3-hydroxypropanethioate (**6'd**), OJJ-198.

The title compound **6'd** was prepared following the reaction sequences of GP-6 as shown in Scheme S22.

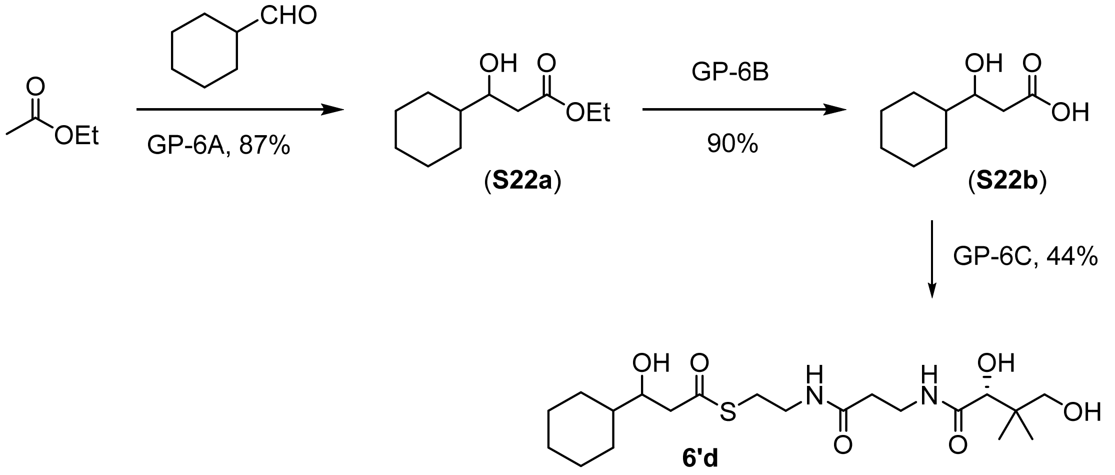

Scheme S22. Synthesis of **6'd** from cyclohexanecarboxaldehyde

3.6.4.1 Synthesis of ethyl 3-cyclohexyl-3-hydroxypropanoate (**S22a**), OJJ-176.

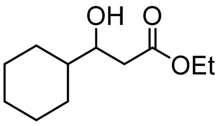

(**S22a**)

3-hydroxy ester (**S22a**) was prepared from cyclohexanecaboxaldehyde (0.34 g, 3.0 mmol) following GP-6A providing **S22a** (0.52 g, 87%) and this intermediate was used in the next step without further purification.

3.6.4.2 Synthesis of 3-cyclohexyl-3-hydroxypropanoic acid (**S22b**), OJJ-179.

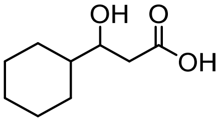

(**S22b**)

3-hydroxy acid (**S22b**) was prepared from corresponding ester **S22a** (0.6 g, 3.0 mmol) following GP-6B providing **S22b** (0.46 g, 90%), characterized by NMR and this intermediate was used in the next step without further purification.

^{1}H NMR (500 MHz, MeOD) δ 3.77 (dddd, *J* = 6.9, 5.0, 3.8, 1.1 Hz, 1H), 2.50 (ddd, *J* = 15.2, 3.8, 1.0 Hz, 1H), 2.33 (ddd, *J* = 15.2, 9.3, 1.2 Hz, 1H), 1.90 – 1.82 (m, 1H), 1.82 – 1.72 (m, 2H), 1.71 – 1.61 (m, 2H), 1.35 (dddd, *J* = 12.2, 9.3, 7.4, 3.2 Hz, 1H), 1.30 – 1.14 (m, 3H), 1.13 – 0.97 (m, 2H). **^{13}C{^{1}H} NMR** (126 MHz, MeOD) δ 176.2, 73.56, 44.9, 40.3, 30.2, 29.2, 27.6, 27.4, 27.3.

IR (film, cm^{-1}): 3336, 2923, 2850, 2544, 1681, 1445, 1435, 1398, 1371, 1305, 1279, 1245, 1223, 1189, 1151, 1103, 1086, 1038, 989, 975, 903, 590.

HRMS (ESI^{+}) m/z: [M+Na]$^{+}$ calculated for [$C_9H_{16}NaO_3$]$^{+}$= 195.0992, found 195.0981, Δ=−5.86 ppm.

3.6.4.3 S-(2-(3-((*R*)-2,4-dihydroxy-3,3-dimethylbutanamido)propanamido)ethyl) 3-cyclohexyl-3-hydroxypropanethioate (**6’d**), OJJ-198.

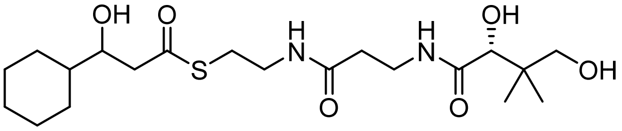

6'd

The (3*RS*)-hydroxy thioester **6’d** was prepared from corresponding 3-hydroxy acid **S22b** (0.142 g, 0.82 mmol) via coupling reaction following GP-6C providing **6’d** (0.157 g, 44%) as a colorless sticky liquid.

***R*$_{f}$** = 0.45 in 10% MeOH/DCM.

^{1}H NMR (300 MHz, MeOD) δ 3.89 (s, 1H), 3.82 (ddd, *J* = 9.3, 5.7, 3.8 Hz, 1H), 3.47 (dd, *J* = 11.6, 5.7 Hz, 3H), 3.39 (d, *J* = 11.3 Hz, 2H), 3.34 (d, *J* = 6.7 Hz, 1H), 3.02 (t, *J* = 6.6 Hz, 2H), 2.82 – 2.56 (m, 2H), 2.41 (t, *J* = 6.6 Hz, 2H), 1.90 – 1.57 (m, 5H), 1.41 – 0.98 (m, 6H), 0.93 – 0.91 (m, 6H). **^{13}C{^{1}H} NMR** (75 MHz, MeOD) δ 199.4, 175.9, 173.9, 77.3, 73.7, 70.4,

45.0, 40.3, 40.0, 36.4, 36.3, 30.2, 29.3, 29.1, 27.5, 27.3, 27.2, 21.3, 21.0. The NMR data match with those obtained for **6d** (see Section 3.4.5).

3.6.5 Synthesis of *tert*-butyl 4-(3-((2-(3-((*R*)-2,4-dihydroxy-3,3-dimethylbutanamido)propanamido)ethyl)thio)-1-hydroxy-3-oxopropyl)piperidine-1-carboxylate (**6'e**), OJJ-99.

The title compound **6'e** was prepared following the reaction sequences of GP-6 as shown in Scheme S23.

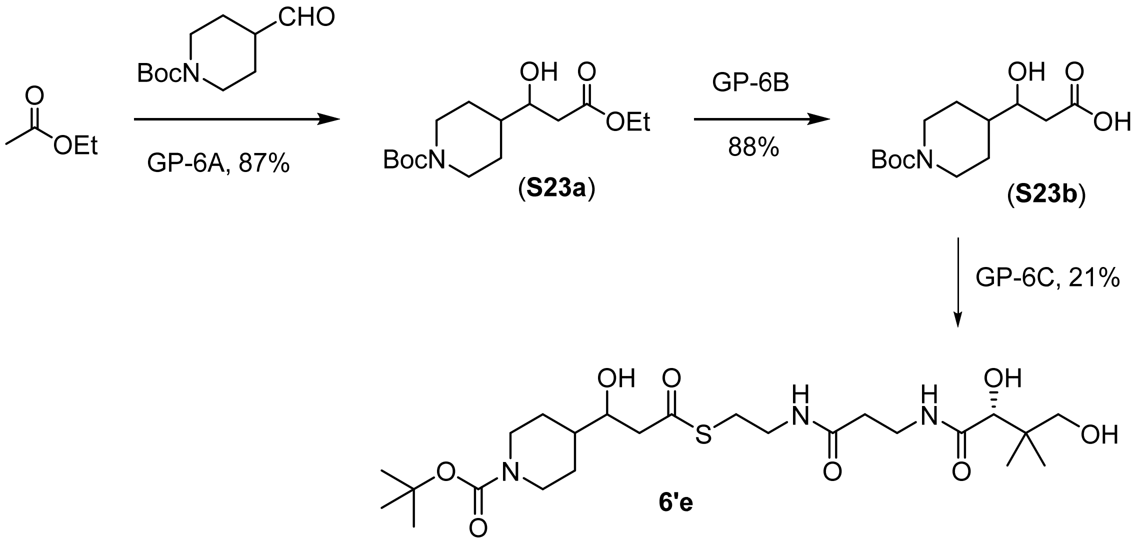

Scheme S23. Synthesis of **6'e** from Cyclohexanecarboxaldehyde

3.6.5.1 *tert*-butyl 4-(3-ethoxy-1-hydroxy-3-oxopropyl)piperidine-1-carboxylate (**S23a**), OJJ-54.

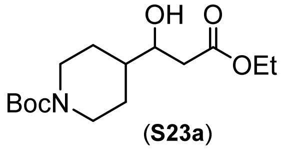

3-hydroxy ester (**S23a**) was prepared from 1-Boc-piperidine-4-carboxaldehyde (0.5 g, 2.3 mmol) following GP-6A providing **S23a** (0.6 g, 87%). This intermediate was used in the next step without further purification.

3.6.5.2 3-(1-(*tert*-butoxycarbonyl)piperidin-4-yl)-3-hydroxypropanoic acid (**S23b**), OJJ-55.

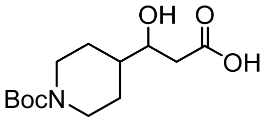

(**S23b**)

3-hydroxy acid (**S23b**) was prepared from corresponding ester **S23a** (0.6 g, 2.0 mmol) following GP-6B providing **S23b** (0.48 g, 88% crude yield). No further purification was necessary.

^{1}H NMR (500 MHz, MeOD) δ 4.11 (dt, *J* = 12.3, 5.2 Hz, 2H), 3.85 – 3.77 (m, 1H), 2.71 (m, 2H), 2.53 (dt, *J* = 15.4, 3.0 Hz, 1H), 2.36 (ddd, *J* = 15.3, 9.2, 2.0 Hz, 1H), 1.80 (d, *J* = 13.3 Hz, 1H), 1.67–1.51 (m, 2H), 1.45 (s, 9H), 1.34–1.12 (m, 2H). **^{13}C{^{1}H} NMR** (126 MHz, MeOD) δ 175.7, 156.4, 80.9, 72.6, 45.3, 44.5, 43.0, 40.3, 29.5, 28.7, 28.2.

IR (film, cm^{-1}): 3433, 2977, 2952, 2928, 2886, 2862, 2545, 1697, 1654, 1467, 1438, 1393, 13667, 1298, 1236, 1159, 1139, 1114, 1021, 1008, 993, 942, 903, 775, 548.

HRMS (ESI^{+}) m/z: [M+Na]$^{+}$ calculated for $[C_{13}H_{23}NNaO_5]^{+}$= 296.1468, found 296.1479, Δ= −2.87 ppm.

3.6.5.3 *tert*-butyl 4-(3-((2-(3-((*R*)-2,4-dihydroxy-3,3-dimethylbutanamido)propanamido)ethyl)thio)-1-hydroxy-3-oxopropyl)piperidine-1-carboxylate (**6’e**), OJJ-99.

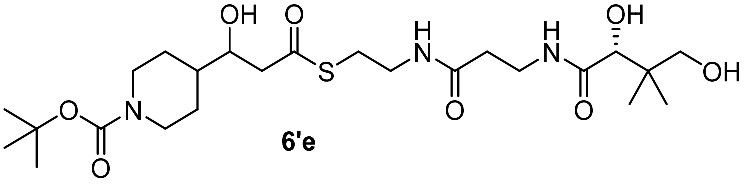

6'e

The (3*RS*)-hydroxy thioester **6’e** was prepared from corresponding 3-hydroxy acid **S23b** (0.2 g, 0.73 mmol) via coupling reaction following GP-6C providing **6’e** (0.08 g, 21%) as a colorless sticky liquid.

***R*$_{f}$** = 0.5 in 10% MeOH/DCM.

^{1}H NMR (500 MHz, MeOD) δ 4.16 – 4.06 (m, 2H), 3.89 (s, 1H), 3.86 (ddd, *J* = 9.3, 5.8, 3.7 Hz, 1H), 3.53 – 3.42 (m, 3H), 3.39 (d, *J* = 10.9 Hz, 1H), 3.35 (t, *J* = 6.6 Hz, 2H), 3.06 – 2.99 (m, 2H), 2.81 – 2.63 (m, 4H), 2.41 (t, *J* = 6.7 Hz, 2H), 1.79 (dt, *J* = 13.2, 2.8 Hz, 1H), 1.64 –

1.50 (m, 2H), 1.45 (s, 9H), 1.31 – 1.13 (m, 2H), 0.92 (s, 6H). **^{13}C{^{1}H} NMR** (126 MHz, MeOD) δ 199.1, 176.0, 173.9, 156.5, 80.9, 77.3, 72.8, 70.3, 45.3, 43.1, 40.4, 40.0, 36.4, 36.3, 29.5, 29.4, 28.7, 28.2, 21.3, 20.9. The NMR data match with those obtained for **6e** (see Section 3.4.6).

3.6.6 Synthesis of S-(2-(3-((*R*)-2,4-dihydroxy-3,3-dimethylbutanamido)propanamido)ethyl) 7-bromo-3-hydroxyheptanethioate (**6'f**), OJJ-212.

The title compound **6'f** was prepared from aldehyde **S24a** following the reaction sequences as shown in Scheme S24.

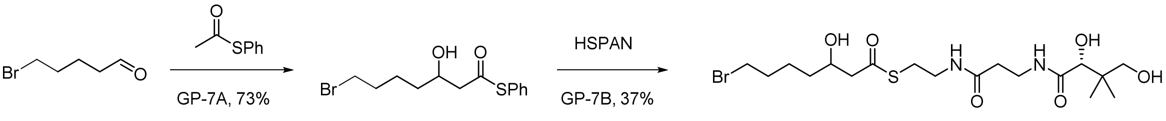

Scheme S24. Synthesis of **6'f** from 5-bromopentanal (**S24a**)

3.6.6.1 Synthesis of 5-bromopentanal (**S24a**), OJJ-186/OJJ-168.

The aldehyde **S24a** was prepared from 1, 5-pentanediol (**S25a**) following the reaction sequences as shown in Scheme S25. Note: Compound **S24a** was volatile in nature and special care was taken during its synthesis.

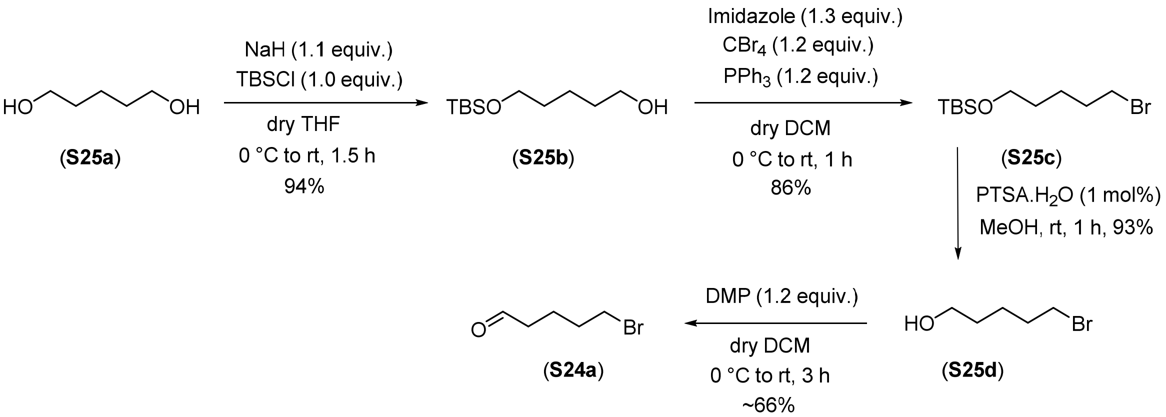

Scheme S25. Synthesis of **S24a** from 1, 5-pentanediol (**S25a**)

3.6.6.1.1 Synthesis of 5-((*tert*-butyldimethylsilyl)oxy)pentan-1-ol (**S25b**), OJJ-161.

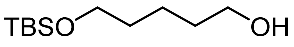

(**S25b**)

NaH (60% dispersion in oil, 1.27 g, 31.68 mmol, 1.1 equiv.) was washed thoroughly with dry hexane (5.0 mL) and the solvent was decanted under argon atmosphere and the process was repeated for 3 times. 115 mL of dry THF was added to it, and the reaction mixture was cooled down to 0 °C. A solution of 1,5-pentanediol (3.0 g, 28.8 mmol, 1.0 equiv.) in 25 mL dry THF was added dropwise using a liquid addition funnel over a period of 15-20 min at 0 °C. After stirring it for 1 h at rt, the mixture was again cooled down to 0 °C, and a solution of tert-butyldimethylchlorosilane (4.34 g, 28.8 mmol, 1.0 equiv.) in 50 mL dry THF was added dropwise over a period of 30 min. The reaction mixture was brought to rt and after 1 h it was quenched with addition of 5% aqueous K_2CO_3 (100 mL) solution and subsequently extracted with diethyl ether (3x100 mL). The combined organic layer was dried (Na_2SO_4), concentrated and the crude residue was purified using CombiFlash (24 g Silica, 15% EtOAc/hexane) providing mono-TBS protected alcohol **S25b** (5.9 g, 94%) as a colorless oil. The spectral data matches with the previously reported literature data[39].

R$_f$ = 0.3 in 20 % EtOAc/Hex.

^{1}H NMR (300 MHz, $CDCl_3$) δ 3.58 (t, *J* = 6.4 Hz, 4H), 2.32 (bs, 1H), 1.61 – 1.44 (m, 4H), 1.43 – 1.29 (m, 2H), 0.85 (s, 9H), 0.01 (s, 6H). **^{13}C{^{1}H} NMR** (75 MHz, $CDCl_3$) δ 63.2, 62.7, 32.6, 32.5, 26.0, 22.1, 18.4, -5.2.

3.6.6.1.2 Synthesis of ((5-bromopentyl)oxy)(tert-butyl)dimethylsilane (**S25c**), OJJ-166

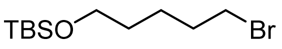

(**S25c**)

To a stirred suspension of TBS-protected alcohol **S25b** (0.7 g, 3.21 mmol, 1 equiv.) in dry DCM at 0 °C was added successively imidazole (0.28 g, 4.2 mmol, 1.3 equiv.) and tetrabromomethane (1.28 g, 3.9 g, 1.2 equiv.). Triphenylphosphine (1.0 g, 3.9 mmol, 1.2 equiv.) was introduced portion wise to this solution and the reaction mixture was stirred for another 15 min at 0 °C. The reaction mixture was allowed to acclimate at rt. After 2 h, when almost all the starting was consumed (monitored by TLC), solvent was evaporated and the reaction mixture was subjected directly to purification using CombiFlash (12 g Silica, 0-5 % EtOAc/hexane) providing desired bromo compound **S25c** (0.77 g, 85%) as a colorless oil. The spectral data matches with the reported literature data[40].

R$_f$ = 0.8 in 10 % EtOAc/Hex.

^{1}H NMR (500 MHz, $CDCl_3$) δ 3.61 (t, *J* = 6.2 Hz, 2H), 3.40 (t, *J* = 6.9 Hz, 2H), 1.87 (p, *J* = 7.0 Hz, 2H), 1.58 – 1.43 (m, 4H), 0.89 (s, 9H), 0.04 (s, 6H). **$^{13}C\{^1H\}$ NMR** (126 MHz, $CDCl_3$) δ 63.0, 33.9, 32.8, 32.0, 26.1, 24.7, 18.5, -5.2.

3.6.6.1.3 Synthesis of 5-bromopentan-1-ol (**S25d**), OJJ-167/OJJ-184

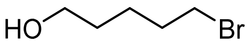

(**S25d**)

To a stirred suspension of bromo TBS-protected alcohol **S25c** (1.35 g, 4.82 mmol, 1 equiv.) in 20 mL of MeOH at rt was added *p*-Toluenesulfonic acid monohydrate (9.2 mg, 0.05 mmol, 1.0 mol%). After 1 h (monitored by TLC), the reaction was quenched with 2.0 mL of saturated aqueous $NaHCO_3$ solution. MeOH was evaporated using rotary evaporator and the reaction mixture was diluted with the addition of DCM (20.0 mL) and water (10.0 mL). The organic layer was separated, and extraction was repeated with DCM (2x20 mL). Combined organic layer was dried (Na_2SO_4), concentrated and the crude residue was purified using CombiFlash (24 g Silica, 25% EtOAc/hexane) providing bromo alcohol **S25d** (0.6 g, 74%) as an colorless oil. The spectral data matches with the reported literature data[40].

R$_f$ = 0.4 in 40 % EtOAc/Hex.

^{1}H NMR (500 MHz, $CDCl_3$) δ 3.71 – 3.63 (m, 2H), 3.43 (t, *J* = 6.8 Hz, 2H), 1.90 (dt, *J* = 14.8, 6.9 Hz, 2H), 1.61 (dtd, *J* = 11.9, 6.3, 3.0 Hz, 2H), 1.57 – 1.49 (m, 2H). **$^{13}C\{^1H\}$ NMR** (75 MHz, $CDCl_3$) δ 62.5, 33.8, 32.6, 31.8, 24.5.

3.6.6.1.4 Synthesis of 5-bromopentanal, OJJ-168/OJJ-186

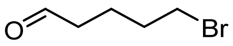

(**S24a**)

Note: This compound **S24a** was volatile in nature and extra care was taken during evaporation of solvent (after carrying out the reaction as well as after purification of the reaction mixture).

To a stirred suspension of bromo alcohol **S25d** (0.57 g, 3.41 mmol, 1.0 equiv.) in 25 mL of dry DCM at 0 °C was added successively solid $NaHCO_3$ (0.63 g, 7.5 mmol, 2.2 equiv.) and Dess–Martin periodinane, DMP, (1.74 g, 4.1 mmol, 1.2 equiv.) under nitrogen. The reaction mixture was allowed to acclimate to rt slowly and continued stirring for additional 2 h (monitored by TLC) before it was quenched with saturated aqueous $NaHCO_3$ solution (25 mL). Subsequently, the reaction mixture was extracted with DCM (3x60 mL) and combined organic layer was dried (Na_2SO_4), concentrated carefully under

reduced pressure using rotary evaporator keeping the bath temperature ~37 °C and pressure at ~750 mbar. The residue was purified using CombiFlash (24 g Silica, 10-15% Et_2O in pentane) and same measure was taken in evaporating the solvent after purification. The desired product **S24a** was isolated in ~66% yield (0.37 g, Note: finding the exact yield of the reaction was difficult because the desired compound was volatile in nature). The spectral data matches with the reported literature data[41].

R$_f$ = 0.5 in 20 % EtOAc/Hex.

^{1}H NMR (500 MHz, $CDCl_3$) δ 9.78 (t, *J* = 1.5 Hz, 1H), 3.42 (td, *J* = 6.6, 1.9 Hz, 2H), 2.49 (td, *J* = 7.1, 1.5 Hz, 2H), 1.97 – 1.84 (m, 2H), 1.83 – 1.76 (m, 2H). **^{13}C{^{1}H} NMR** (126 MHz, $CDCl_3$) δ 201.9, 43.0, 33.2, 32.0, 20.8.

3.6.6.2 Synthesis of S-phenyl-7-bromo-3-hydroxyheptanethioate (**S24b**), OJJ-210.

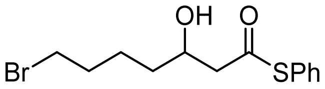

(**S24b**)

The corresponding (3*RS*)-hydroxy-S-phenyl thioester (**S24b**) was prepared from 5-bromopentanal **S24a** (0.165 g, 1.0 mmol) following GP-7A providing **S24b** (0.23 g, 73%) as a colorless oil.

R$_f$ = 0.25 in 20 % EtOAc/Hex.

^{1}H NMR (300 MHz, $CDCl_3$) δ 7.42 (m, 5H), 4.10 (dq, *J* = 11.3, 5.7 Hz, 1H), 3.41 (t, *J* = 6.7 Hz, 2H), 2.93 – 2.72 (m, 2H), 2.69 (h, 1H), 1.90 (ddt, *J* = 9.2, 6.7, 3.2 Hz, 2H), 1.72 – 1.44 (m, 4H). **^{13}C{^{1}H} NMR** (75 MHz, $CDCl_3$) δ 197.9, 134.6, 129.8, 129.4, 127.2, 68.4, 50.3, 35.6, 33.6, 32.6, 24.2.

IR (film, cm^{-1}): 3411, 2936, 2862, 1693, 1477, 1457, 1439, 1403, 1272, 1248, 1201, 1065, 997, 744, 705, 688, 644, 558.

HRMS (ESI^+) m/z: $[M+Na]^+$ calculated for $[C_{13}H_{17}BrO_2NaS]^+$ = 339.0025, found 339.0048, Δ=3.33 ppm.

3.6.6.3 S-(2-(3-((*R*)-2,4-dihydroxy-3,3-dimethylbutanamido)propanamido)ethyl) 7-bromo-3-hydroxyheptanethioate (**6’f**), OJJ-212.

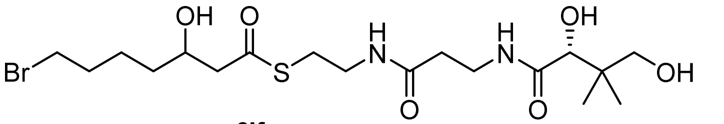

6'f

The (3*RS*)-hydroxy thioester **6’f** was prepared from corresponding (3*RS*)-hydroxy-S-phenyl thioester **S22b** (0.114 g, 0.36 mmol) via thiol-exchange reaction with HSPAN following GP-7B providing **6’f** (0.065 g, 37%) as a colorless sticky liquid.

R_f = 0.5 in 10% MeOH/DCM.

^{1}H NMR (400 MHz, MeOD) δ 8.15 (br m, 1H), δ 7.93 (br m, 1H), δ 4.04 (q, *J* = 6.0 Hz, 1H), 3.89 (s, 1H), 3.54 – 3.42 (m, 5H), 3.41 – 3.35 (m, 2H), 3.34 (d, *J* = 6.4 Hz, 1H), 3.03 (t, *J* = 6.7 Hz, 2H), 2.74 – 2.64 (m, 2H), 2.41 (t, *J* = 6.7 Hz, 2H), 1.86 (dt, *J* = 12.5, 6.9 Hz, 2H), 1.62 (ddd, *J* = 9.7, 7.8, 5.0 Hz, 1H), 1.55 – 1.43 (m, 3H), 0.92 (s, 6H). **^{13}C{^{1}H} NMR** (101 MHz, MeOD) δ 198.8, 176.0, 173.9, 77.4, 70.4, 69.3, 52.6, 40.4, 40.0, 37.1, 36.4, 36.3, 34.2, 33.8, 29.4, 25.3, 21.3, 21.0. The NMR data match with those obtained for **6f** (see Section 3.4.7).

3.6.7 Synthesis of S-(2-(3-((*R*)-2,4-dihydroxy-3,3-dimethylbutanamido)propanamido)ethyl) 6-(1,3-dioxolan-2-yl)-3-hydroxyhexanethioate (**6’g**), OJJ-195

The title compound **6’g** was prepared from aldehyde **S26a** following the reaction sequences as shown in Scheme S26.

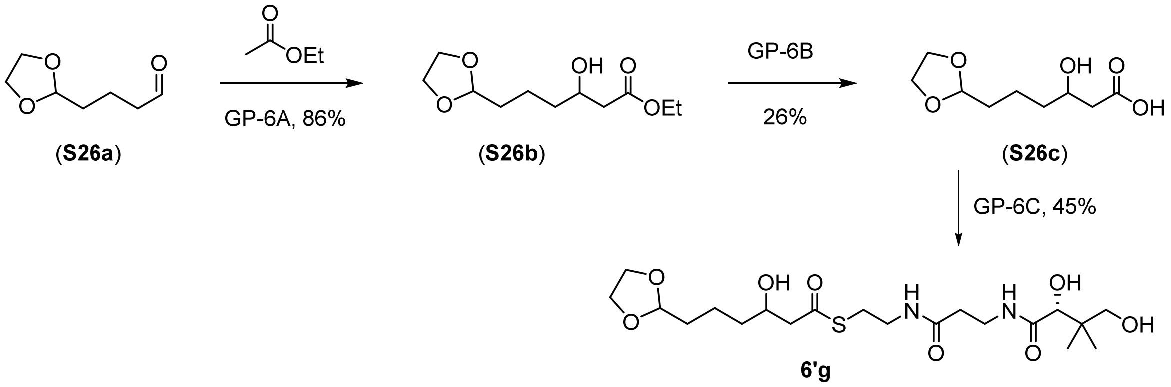

Scheme S26. Synthesis of **6’g** from aldehyde **S26a**

3.6.7.1 Synthesis of 4-(1,3-dioxolan-2-yl)butanal (**S26a**), OJJ-187.

The aldehyde **S26a** was prepared from δ-Valerolactone (**S27a**) following the reaction sequences as shown in Scheme S27.

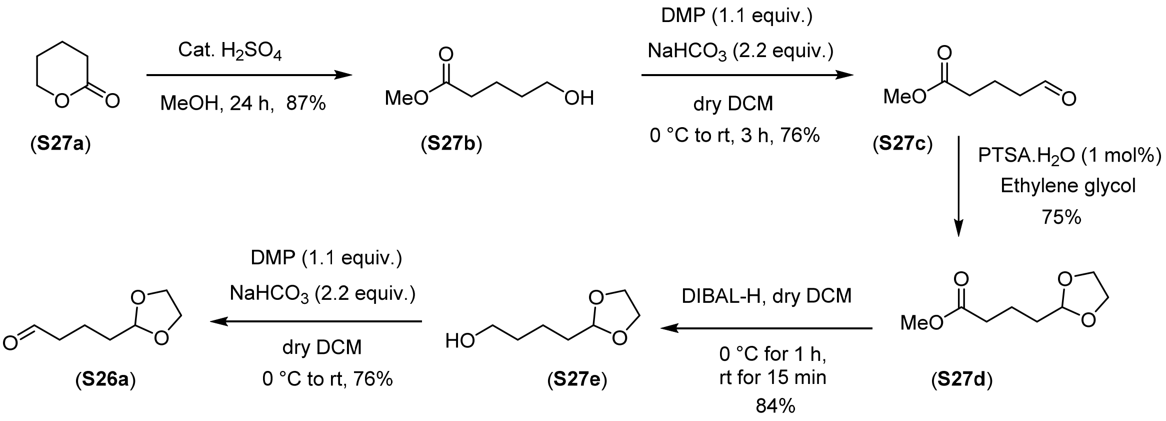

Scheme S27. Synthesis of **S26a** from δ-Valerolactone (**S27a**)

3.6.7.1.1 Synthesis of methyl 5-hydroxypentanoate (**S27b**), Ojj-165-B.

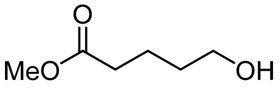

(**S27b**)

The hydroxy ester **S27b** was prepared adapting the literature reported procedure providing **S27b** (1.1g, 87%) as a colorless oil. The spectral data matches with the literature reported data[42].

R_f = 0.4 in 70% EtOAc/Hex.

^{1}H NMR (400 MHz, $CDCl_3$) δ 3.65 (s, 3H), 3.63 (t, *J* = 6.3 Hz, 2H), 2.34 (t, *J* = 7.3 Hz, 2H), 1.89 (s, 1H), 1.76 – 1.63 (m, 2H), 1.62 – 1.53 (m, 2H). **^{13}C{^{1}H} NMR** (75 MHz, $CDCl_3$) δ 174.2, 62.0, 51.5, 33.7, 32.0, 21.1.

IR (film, cm^{-1}): 3400, 2949, 2871, 1731, 1436, 1362, 1317, 1197, 1157, 1056, 1007, 939, 856, 589.

HRMS (ESI$^+$) m/z: $[M+H]^+$ calculated for $[C_6H_{13}O_3]^+$= 133.0859, found 133.0856, Δ= −2.38 ppm.

3.6.7.1.2 Synthesis of methyl 5-oxopentanoate (**S27c**), OJJ-181.

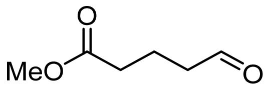

(**S27c**)

To a stirred suspension of alcohol **S27b** (2.0 g, 15.1 mmol, 1 equiv.) in 60 mL of dry DCM at 0 ° was added successively solid $NaHCO_3$ (2.8 g, 33.2 mmol, 2.2 equiv.) and Dess–Martin periodinane (7.7 g, 18.2 mmol, 1.2 equiv.). The reaction mixture was allowed to acclimate to rt slowly and after 2 h (monitored by TLC) it was quenched with saturated aqueous $NaHCO_3$ solution (50 mL). Subsequently, the reaction mixture was extracted with DCM (3x100 mL) and combined organic layer was dried (Na_2SO_4), concentrated and the crude residue was purified using CombiFlash (24 g Silica, 10-15% EtOAc/hexane) providing aldehyde **S27c** (1.5 g, 76%) as a colorless oil.

R_f = 0.4 in 30 % EtOAc/Hex.

^{1}H NMR (300 MHz, $CDCl_3$) δ 9.73 (q, *J* = 1.4 Hz, 1H), 3.69 – 3.56 (m, 3H), 2.54 – 2.40 (m, 2H), 2.39 – 2.25 (m, 2H), 2.02 – 1.83 (m, 2H). **^{13}C{^{1}H} NMR** (75 MHz, $CDCl_3$) δ 201.5, 173.4, 51.6, 43.0, 33.0, 17.4.

IR (film, cm^{-1}): 2954, 1705, 1437, 1416, 1369, 1199, 1151, 1059, 1032, 1014, 899, 862, 786.
HRMS (ESI^+) m/z: $[M+Na]^+$ calculated for $[C_6H_{10}NaO_3]^+$= 153.0522, found 153.0518, Δ=−2.87 ppm.

3.6.7.1.3 Synthesis of methyl 4-(1,3-dioxolan-2-yl)-butanoate (**S27d**), OJJ-182.

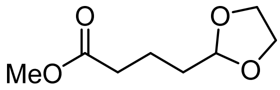

(**S27d**)

To a stirred suspension of aldehyde **S27c** (1.2 g, 9.2 mmol, 1.0 equiv.) in ethylene glycol (1.0 mL, 2.0 equiv.) at rt was added catalytic amount of *p*-Toluene sulfonic acid monohydrate (17 .0 mg, 0.09 mmol, 0.01 equiv.) under nitrogen. Afetr 24 h, the reaction mixture was quenched with saturated aqueous $NaHCO_3$ solution (1.0 mL) and diluted with DCM (20 mL) and H_2O (10 mL). Organic layer was separated, and the aqueous layer was further extracted with DCM (2x20 mL). The combined organic layer was evaporated to dryness, purified by combiFlash providing desired acetal **S27d** (1.2 g, 75%) as a colorless liquid**,** characterized by NMR and proceeded for the next step without further purification.

R_f = 0.4 in 30% EtOAc/Hex.

^{1}H NMR (300 MHz, $CDCl_3$) δ 4.82 (ddd, *J* = 5.4, 3.1, 1.2 Hz, 1H), 3.91 (tdd, *J* = 4.7, 2.4, 1.1 Hz, 2H), 3.83 – 3.75 (m, 2H), 3.62 (dq, *J* = 2.5, 1.2 Hz, 3H), 2.33 (td, *J* = 7.3, 1.5 Hz, 2H), 1.81 – 1.57 (m, 4H). **^{13}C{^{1}H} NMR** (75 MHz, $CDCl_3$) δ 173.8, 104.2, 64.9, 51.5, 33.8, 33.1, 19.4.

IR (film, cm^{-1}): 2953, 2883, 1732, 1436, 1416, 1364, 1335, 1316, 1249, 1197, 1165, 1135, 1031, 989, 941, 841.

3.6.7.1.4 Synthesis of 4-(1,3-dioxolan-2-yl)butan-1-ol (**S27e**), OJJ-185.

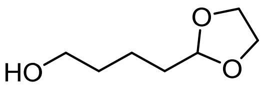

(**S27e**)

A stirred solution of acetal protected ester **S27d** (0.61 g, 3.5 mmol, 1.0 equiv.) in 20 mL of dry DCM at –78 °C was treated with di-isobutylaluminum hydride (1M in hexane, 7.4 mL, 7.4 mmol, 2.1 equiv.). After 1 h, the reaction mixture was allowed to acclimate at rt and stirred for another 0.5 h before it was quenched with 20 mL of aqueous saturated solution of potassium sodium tartrate tetrahydrate (Rochelle Salt). The solution was diluted with another 20 mL of DCM and continued to stir until the organic and aqueous layers got nicely separated. Organic layer was separated, and the aqueous layer was further extracted with DCM (2x40 mL). The combined organic layer was evaporated to dryness, purified by CombiFlash by passing 50% EtOAc/hexane providing desired acetal **S27e** (0.43 g, 84%) as a colorless liquid. This intermediate was used in the next step without further purification.

R_f = 0.3 in 50% EtOAc/Hex.

^{1}H NMR (300 MHz, $CDCl_3$) δ 4.84 (t, *J* = 4.7 Hz, 1H), 4.00 – 3.91 (m, 2H), 3.86 – 3.78 (m, 2H), 3.61 (t, *J* = 6.4 Hz, 2H), 1.70 – 1.63 (m, 2H), 1.63 – 1.54 (m, 2H), 1.53 – 1.44 (m, 2H). **^{13}C{^{1}H} NMR** (75 MHz, $CDCl_3$) δ 104.6, 65.0, 62.7, 33.6, 32.6, 20.3.

IR (film, cm^{-1}): 2952, 2882, 1719, 1409, 1138, 1084, 1049, 1024, 943, 898, 857.

HRMS (ESI^+) m/z: $[M+Na]^+$ calculated for $[C_7H_{14}NaO_3]^+$= 169.0835, found 169.0826, Δ=−6.12 ppm.

3.6.7.1.5 Synthesis of 4-(1,3-dioxolan-2-yl)butanal (**S26a**), OJJ-187.

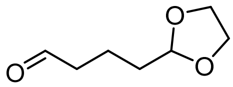

(**S26a**)

To a stirred suspension of alcohol **S27e** (0.4 g, 2.74 mmol, 1.0 equiv.) in 40 mL of dry DCM at 0 °C were added $NaHCO_3$ (0.51 g, 6.0 mmol, 2.2 equiv.) and Dess–Martin periodinane (1.4 g, 3.3 mmol, 1.2 equiv.) successively. Then the reaction mixture was allowed to acclimate to rt and continued stirring for additional 2 h before it was quenched with saturated aqueous $NaHCO_3$ solution (30 mL). Subsequently, organic layer was separated, and the aqueous layer was further extracted with DCM (2x 80 mL). The combined organic layer was dried (Na_2SO_4), concentrated and the crude residue was purified using CombiFlash by passing 25-30% EtOAc/hexane providing the desired aldehyde **S26a** (0.3 g, 76%) as a colorless oil.

R_f = 0.3 in 30% EtOAc/Hex.

^{1}H NMR (300 MHz, $CDCl_3$) δ 9.74 (t, *J* = 1.6 Hz, 1H), 4.84 (t, *J* = 4.3 Hz, 1H), 3.99 – 3.89 (m, 2H), 3.85 – 3.76 (m, 2H), 2.47 (td, *J* = 7.1, 1.6 Hz, 2H), 1.83 – 1.58 (m, 4H). **^{13}C{^{1}H} NMR** (75 MHz, CDCl3) δ 202.3, 104.2, 65.0, 43.6, 33.0, 16.6.

IR (film, cm^{-1}): 2953, 2882, 2725, 1719, 1454, 1436, 1409, 1226, 1138, 1084, 1049, 1024, 943, 857, 708, 659, 515.
HRMS (ESI^+) m/z: $[M+H]^+$ calculated for $[C_7H_{13}O_3]^+$= 145.0859, found 145.0850, Δ=−5.88 ppm.

3.6.7.2 Synthesis of ethyl 6-(1,3-dioxolan-2-yl)-3-hydroxyhexanoate (**S26b**), OJJ-188.

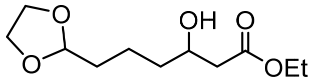

(**S26b**)

3-hydroxy ester (**S26b**) was prepared from aldehyde **S26a** (0.43 g, 3 .0mmol) following GP-6A providing **S26b** (0.6 g, 86%) as a colorless oil. This intermediate was used in the next step without further purification.

3.6.7.3 Synthesis of 6-(1,3-dioxolan-2-yl)-3-hydroxyhexanoic acid (**S26c**), OJJ-192.

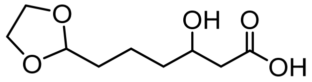

(**S26c**)

3-hydroxy acid (**S26c**) was prepared from corresponding ester **S26b** (0.6 g, 2.6 mmol) following GP-6B providing **S26c** (0.14 g, 26%) as a colorless liquid and was characterized by NMR. This intermediate was used in the next step without further purification.

^{1}H NMR (300 MHz, $CDCl_3$) δ 4.86 (t, *J* = 4.5 Hz, 1H), 4.04 (m, 1H), 3.98 – 3.93 (m, 2H), 3.85 (dt, *J* = 3.7, 2.1 Hz, 2H), 2.63 – 2.42 (m, 2H), 1.69 (dd, *J* = 8.8, 3.6 Hz, 2H), 1.63 – 1.46 (m, 4H). **^{13}C{^{1}H} NMR** (75 MHz, $CDCl_3$) δ 177.1, 104.5, 68.0, 65.0, 41.2, 36.4, 33.5, 20.0.

IR (film, cm^{-1}): 3400, 2939, 1708, 1401, 1287, 1261, 1201, 1126, 1082, 1055, 1028, 984, 942, 882, 620.

HRMS (ESI^+) m/z: $[M+Na]^+$ calculated for $[C_9H_{16}NaO_5]^+$= 227.0890, found 227.0880, Δ=−5.06 ppm.

3.6.7.4 Synthesis of S-(2-(3-((*R*)-2,4-dihydroxy-3,3-dimethylbutanamido)propanamido)ethyl) 6-(1,3-dioxolan-2-yl)-3-hydroxyhexanethioate (**6’g**), OJJ-195.

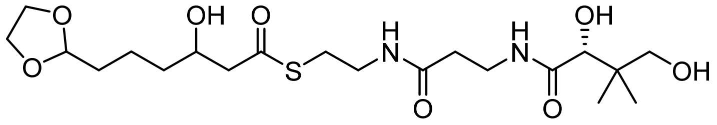

6'g

The (3*RS*)-hydroxy thioester **6’g** was prepared from corresponding 3-hydroxy acid **S26c** (0.11 g, 0.54 mmol) via coupling reaction according following GP-6C providing **6’g** (0.113 g, 45%) as a colorless sticky liquid.

***R*$_f$** = 0.5 in 10% MeOH/DCM.

^{1}H NMR (300 MHz, MeOD) δ 8.14 (br m, 1H), δ 7.92 (br m, 1H), δ 4.82 (t, *J* = 4.6 Hz, 1H), 4.08 – 3.98 (m, 1H), 3.97 – 3.90 (m, 2H), 3.89 (s, 1H), 3.86 – 3.77 (m, 2H), 3.51 – 3.43 (m, 3H), 3.42 – 3.36 (m, 2H), 3.35 – 3.32 (m, 1H), 3.08 – 2.98 (m, 2H), 2.74 – 2.67 (m, 2H), 2.41 (t, *J* = 6.6 Hz, 2H), 1.61 (m, 3H), 1.51 (m, 3H), 0.92 (s, 6H). **^{13}C{^{1}H} NMR** (75 MHz, MeOD) δ 198.8, 176.0, 174.0, 105.6, 77.4, 70.4, 69.4, 65.8, 52.6, 40.4, 40.0, 37.9, 36.5, 36.3, 34.7, 29.3, 21.3, 21.1, 21.0. The NMR data match with those obtained for **6g** (see Section 3.4.8).

3.6.8 Synthesis of methyl 7-((2-(3-((*R*)-2,4-dihydroxy-3,3-dimethylbutanamido)propanamido)ethyl)thio)-5-hydroxy-7-oxoheptanoate (**6'h**), OJJ-208.

The title compound **6'h** was prepared from aldehyde **S27c** following the reaction sequences as shown in Scheme S28.

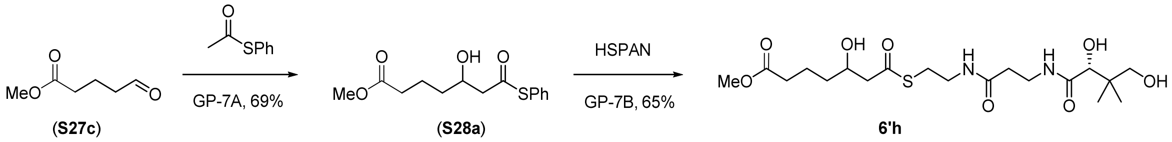

Scheme S28. Synthesis of **6'h** from aldehyde **S27c**

3.6.8.1 Synthesis of methyl 5-oxopentanoate (**S27c**), OJJ-181.

The aldehyde **S27c** was synthesized from corresponding alcohol **S27b** following the reaction sequences as shown in Scheme S27.

3.6.8.2 Synthesis of methyl 5-hydroxy-7-oxo-7-(phenylthio)heptanoate (**S28a**), OJJ-207-B

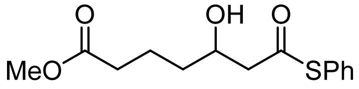

(**S28a**)

The corresponding (3*RS*)-hydroxy-S-phenyl thioester (**S28a**) was prepared from aldehyde **S27c** (0.13 g, 1.0 mmol) following GP-7A providing **S28a** (0.2 g, 69%) as a colorless liquid.

R$_f$ = 0.3 in 30% EtOAc/Hex.
^{1}H NMR (500 MHz, $CDCl_3$) δ 7.41 (m, 5H), 4.09 (tq, *J* = 8.3, 4.1 Hz, 1H), 3.66 (s, 3H), 2.86 (d, *J* = 4.0 Hz, 1H), 2.84 – 2.75 (m, 2H), 2.35 (t, *J* = 7.4 Hz, 2H), 1.86 – 1.75 (m, 1H), 1.71 (dtd, *J* = 13.5, 7.8, 4.5 Hz, 1H), 1.59 – 1.45 (m, 2H). **^{13}C{^{1}H} NMR** (126 MHz, $CDCl_3$) δ 197.8, 174.1, 134.6, 129.7, 129.4, 127.2, 68.1, 51.7, 50.3, 35.8, 33.7, 20.9.

IR (film, cm^{-1}): 3447, 2949, 1731, 1700, 1477, 1438, 1415, 1362, 1330, 1241, 1197, 1154, 1067, 985, 915, 860, 746, 705, 689, 588, 541.

HRMS (ESI^+) m/z: $[M+Na]^+$ calculated for $[C_{14}H_{18}NaO_4S]^+$= 305.0818, found 305.0835, Δ=−2.22 ppm.

3.6.8.3 methyl 7-((2-(3-((*R*)-2,4-dihydroxy-3,3-dimethylbutanamido)propanamido)ethyl)thio)-5-hydroxy-7-oxoheptanoate (**6'h**), OJJ-208.

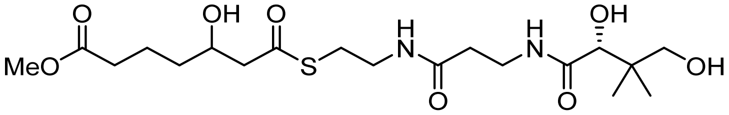

6'h

The (3*RS*)-hydroxy thioester **6'h** was prepared from corresponding (3*RS*)-hydroxy-S-phenyl thioester **S28a** (0.16 g, 0.57 mmol) via thiol-exchange reaction with HSPAN following GP-7B providing **6'h** (0.165 g, 65%) as a colorless sticky liquid.

$\boldsymbol{R_f}$ = 0.5 in 10% MeOH/DCM.

^{1}H NMR (300 MHz, MeOD) δ 8.12 (br m, 1H), δ 7.91 (br m, 1H), δ 4.02 (p, *J* = 6.4 Hz, 1H), 3.88 (s, 1H), 3.64 (s, 3H), 3.55 – 3.42 (m, 3H), 3.42 – 3.34 (m, 2H), 3.33 (d, *J* = 2.0 Hz, 1H), 3.01 (t, *J* = 6.6 Hz, 2H), 2.70 (d, *J* = 6.4 Hz, 2H), 2.40 (t, *J* = 6.7 Hz, 2H), 2.34 (t, *J* = 7.1 Hz, 2H), 1.84 – 1.70 (m, 1H), 1.70 – 1.57 (m, 1H), 1.48 (dtd, *J* = 9.6, 6.6, 5.1 Hz, 2H), 0.91 (s, 6H). **^{13}C{^{1}H} NMR** (75 MHz, MeOD) δ 198.7, 176.0, 175.7, 173.9, 77.3, 70.4, 69.1, 52.5, 52.0, 40.4, 40.0, 37.3, 36.4, 36.3, 34.5, 29.3, 22.1, 21.3, 21.0. The NMR data match with those obtained for **6h** (see Section 3.4.9).

3.6.9 Synthesis of S-(2-(3-((*R*)-2,4-dihydroxy-3,3-dimethylbutanamido)propanamido)ethyl) 5-(benzyloxy)-3-hydroxypentanethioate (**6'i**), OJJ-197.

The title compound **6'i** was prepared following the reaction sequences as shown in Scheme S29.

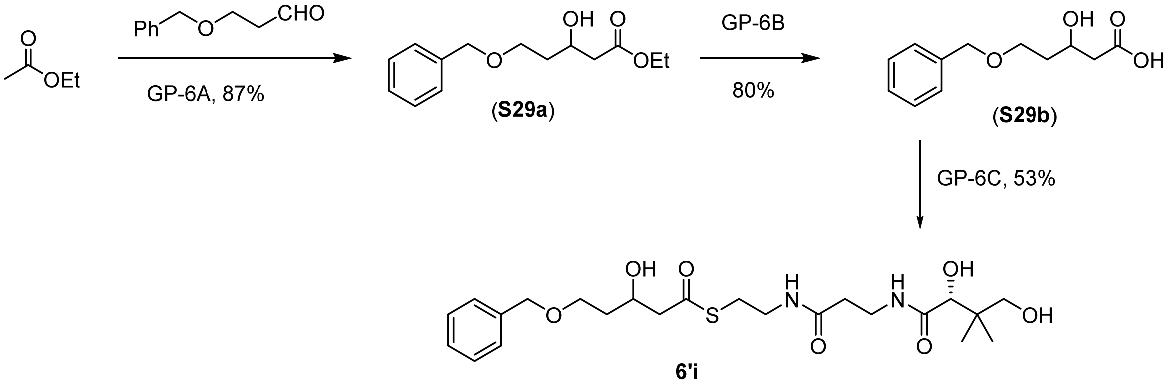

Scheme S29. Synthesis of **6'i** from 3-(benzyloxy)propanal

3.6.9.1 Synthesis of ethyl 5-(benzyloxy)-3-hydroxypentanoate (**S29a**), OJJ-174.

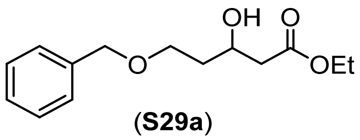

(**S29a**)

3-hydroxy ester (**S29a**) was prepared from 3-(benzyloxy)propanal (0.3 g, 3.0 mmol) following GP-6A providing **S29a** (0.41 g, 87%) as a colorless liquid and this intermediate was used in the next step without further purification.

5-(benzyloxy)-3-hydroxypentanoic acid (**S29b**), OJJ-178.

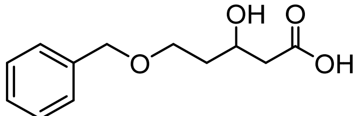

(**S29b**)

3-hydroxy acid (**S29b**) was prepared from corresponding ester **S29a** (0.7 g, 2.7 mmol) following GP-6B providing **S29b** (0.5 g, 80%) as a colorless liquid, characterizedby NMR and this intermediate was used in the next step without further purification.

^{1}H NMR (500 MHz, MeOD) δ 7.35 – 7.30 (m, 4H), 7.26 (ddt, *J* = 8.6, 5.6, 2.8 Hz, 1H), 4.50 (d, *J* = 1.5 Hz, 2H), 4.18 (tt, *J* = 8.5, 4.5 Hz, 1H), 3.68 – 3.55 (m, 2H), 2.49 (dd, *J* = 15.4, 4.7 Hz, 1H), 2.41 (dd, *J* = 15.4, 8.2 Hz, 1H), 1.83 (dddd, *J* = 13.9, 7.7, 6.2, 4.4 Hz, 1H), 1.73 (ddt, *J* = 14.1, 8.4, 5.7 Hz, 1H). **^{13}C{^{1}H} NMR** (126 MHz, MeOD) δ 175.4, 139.7, 129.4, 128.8, 128.6, 74.0, 68.1, 66.9, 43.2, 37.8.

IR (film, cm^{-1}): 3030, 2922, 2864, 1707, 1453, 1406, 1362, 1273, 1205, 1171, 1073, 1025, 909, 872, 737, 697, 608.

HRMS (ESI$^+$) m/z: [M+H]$^+$ calculated for [$C_{12}H_{17}O_4$]$^+$= 225.1121, found 225.1104, Δ= −8.02 ppm.

3.6.9.2 S-(2-(3-((*R*)-2,4-dihydroxy-3,3-dimethylbutanamido)propanamido)ethyl) 5-(benzyloxy)-3-hydroxypentanethioate (**6'i**), OJJ-197.

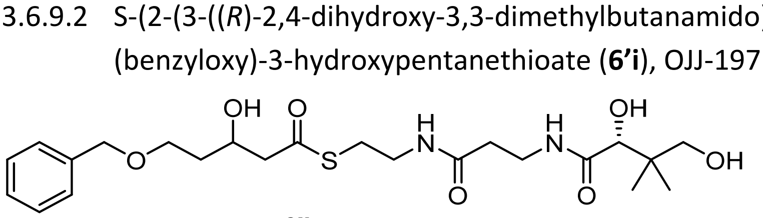

6'i

The (3*RS*)-hydroxy thioester **6'i** was prepared from corresponding 3-hydroxy acid **S29b** (0.2 g, 0.8 mmol) via coupling reaction following GP-6C providing **6'i** (0.21 g, 53%) as a colorless sticky liquid.

***R*$_f$** = 0.55 in 10% MeOH/DCM.

^{1}H NMR (300 MHz, MeOD) δ 7.32 (dt, *J* = 4.3, 0.6 Hz, 4H), 7.29 – 7.21 (m, 1H), 4.49 (s, 2H), 4.22 (tt, *J* = 7.7, 5.0 Hz, 1H), 3.88 (s, 1H), 3.65 – 3.47 (m, 3H), 3.46 – 3.38 (m, 2H), 3.35 (m, 1H), 3.01 (t, *J* = 6.6 Hz, 2H), 2.73 (dd, *J* = 6.4, 1.7 Hz, 2H), 2.39 (t, *J* = 6.6 Hz, 2H), 1.87 – 1.62 (m, 2H), 0.91 (s, 6H). **^{13}C{^{1}H} NMR** (75 MHz, MeOD) δ 198.7, 176.0, 173.9, 139.7, 129.4, 128.8, 128.6, 77.4, 74.0, 70.4, 68.0, 67.2, 52.6, 40.4, 40.0, 37.9, 36.4, 36.3, 29.3, 21.3, 21.0. The NMR data match with those obtained for **6h** (see Section 3.4.10).

3.6.10 Synthesis of S-(2-(3-((*R*)-2,4-dihydroxy-3,3-dimethylbutanamido)propanamido)ethyl) 7-((*tert*-butyldimethylsilyl)oxy)-3-hydroxyheptanethioate (**6'j**), OJJ-211.

The title compound **6'j** was prepared from aldehyde **S30a** following the reaction sequences as shown in Scheme S30.

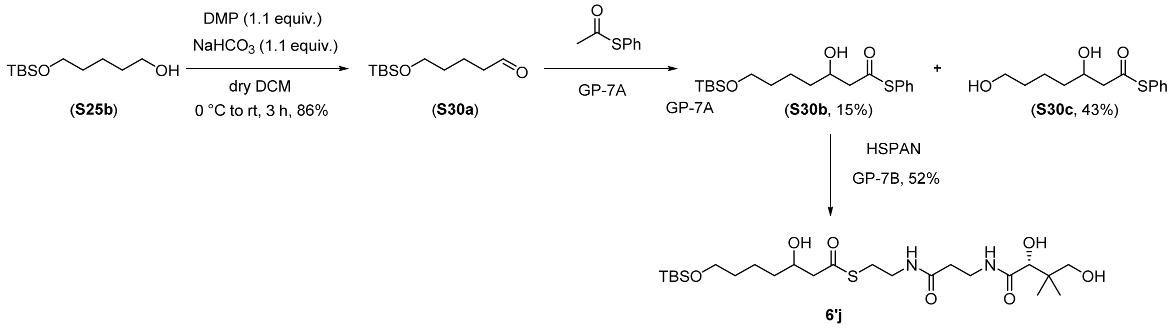

Scheme S30. Synthesis of **6'j** from 5-((*tert*-butyldimethylsilyl)oxy)pentanal (**S30a**)

3.6.10.1 Synthesis of 5-((*tert*-butyldimethylsilyl)oxy)pentanal (**S30a**), OJJ-159/162.

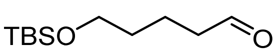

(**S30a**)

To a stirred suspension of TBS-protected alcohol **S25b** (2.0 g, 9.16 mmol, 1 equiv.) in 90 mL of dry DCM at 0 °C were added solid $NaHCO_3$ (1.7 g, 20.2 mmol, 1.1 equiv.) and Dess–Martin periodinane (4.27 g, 10.1 mmol, 1.1 equiv.) successively. The reaction mixture was allowed to acclimate to rt slowly and continued stirring for additional 2 h before it was quenched with saturated aqueous $NaHCO_3$ solution (80 mL). Subsequently, the reaction mixture was extracted with DCM (3x100 mL) and combined organic layer was dried (Na_2SO_4), concentrated and the crude residue was purified using CombiFlash (24 g Silica, 10% EtOAc/hexane) providing

S30a (1.7 g, 86% yield) as a colorless oil. Spectral data matches with the previously reported literature data[39].

***R*$_f$** = 0.65 in 20 % EtOAc in Hex.

^{1}H NMR (500 MHz, $CDCl_3$) δ 9.76 (s, 1H), 3.62 (t, *J* = 6.2, 2H), 2.45 (t, *J* = 7.3, 2H), 1.74 – 1.65 (m, 2H), 1.60 – 1.50 (m, 2H), 0.88 (s, 9H), 0.04 (s, 6H). **^{13}C{^{1}H} NMR** (126 MHz, $CDCl_3$) δ 202.9, 62.7, 43.8, 32.2, 26.1, 18.8, 18.5, -5.2.

3.6.10.2 Synthesis of S-phenyl 7-((*tert*-butyldimethylsilyl)oxy)-3-hydroxyheptanethioate (**S30b**), OJJ-209.

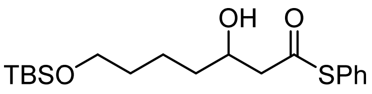

(**S30b**)

The corresponding (3*RS*)-hydroxy-S-phenyl thioester (**S30b**) was prepared from aldehyde **S30a** (0.24 g, 1.0 mmol) following GP-7A providing **S30b** (0.04 g, 15%) as a colorless liquid.

R$_f$ = 0.5 in 20 % EtOAc/Hex.

^{1}H NMR (300 MHz, $CDCl_3$) δ 7.42 (m, 5H), 4.10 (m, 1H), 3.62 (t, *J* = 5.4 Hz, 2H), 2.92 – 2.73 (m, 2H), 2.68 (h, 1H), 1.62 – 1.37 (m, 6H), 0.90 (s, 9H), 0.05 (s, 6H). **^{13}C{^{1}H} NMR** (75 MHz, $CDCl_3$) δ 197.9, 134.6, 129.7, 129.4, 127.4, 68.7, 63.1, 50.4, 36.4, 32.7, 26.1, 21.9, 18.5, -5.1.
IR (film, cm^{-1}): 3287, 2935, 2858, 1700, 1439, 1403, 1251, 1098, 1046, 1031, 1007, 979, 960, 862, 833, 772, 750, 704, 688, 662, 446, 419.
HRMS (ESI$^+$) m/z: $[M+K]^+$ calculated for $[C_{19}H_{32}KO_3SSi]^+$= 407.1473, found 407.1468, Δ=−0.26 ppm.

3.6.10.3 Synthesis of S-phenyl 3,7-dihydroxyheptanethioate (**S30c**), OJJ-209.

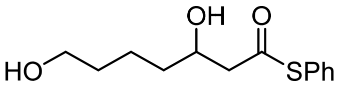

(**S30c**)

During the synthesis of (3*RS*)-hydroxy-S-phenyl thioester (**S30b**), this di-hydroxy thioester **S30c** was also found to be formed providing **S30c** (0.11 g, 43%) as a colorless liquid. This compound was isolated by passing 70-80% EtOAc/Hex in CombiFlash.

R$_f$ = 0.3 in 70 % EtOAc/Hex.

^{1}H NMR (300 MHz, $CDCl_3$) δ 7.41 (m, 5H), 4.09 (m, 1H), 3.61 (t, *J* = 6.0 Hz, 2H), 3.11 (h, 1H, OH), 2.90 – 2.71 (m, 2H), 2.19 (h, 1H, OH), 1.64 – 1.36 (m, 6H). **^{13}C{^{1}H} NMR** (75 MHz, $CDCl_3$) δ 197.8, 134.6, 129.7, 129.4, 127.3, 68.5, 62.5, 50.5, 36.2, 32.3, 21.7.

IR (film, cm^{-1}): 3285, 2935, 2913, 2860, 1700, 1477, 1439, 1403, 1346, 1318, 1273, 1221, 1099, 1047, 1031, 1008, 999, 978, 959, 863, 750, 704, 688, 603, 557.

HRMS (ESI$^+$) m/z: $[M+H]^+$ calculated for $[C_{13}H_{19}O_3S]^+$= 255.1049, found 255.1038, Δ= −7.54 ppm.

3.6.10.4 S-(2-(3-((*R*)-2,4-dihydroxy-3,3-dimethylbutanamido)propanamido)ethyl) 7-((*tert*-butyldimethylsilyl)oxy)-3-hydroxyheptanethioate (**6’j**), OJJ-211.

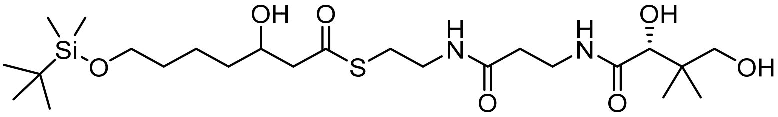

6'j

The (3*RS*)-hydroxy thioester **6’j** was prepared from corresponding (3*RS*)-hydroxy-S-phenyl thioester **S30c** (0.04 g, 0.11 mmol) via thiol-exchange reaction with HSPAN following GP-7B providing **6’j** (0.032 g, 52%) as a colorless sticky liquid.

***R*$_f$** = 0.55 in 10% MeOH/DCM.

^{1}H NMR (300 MHz, MeOD) δ 8.15 (br m, 1H), δ 7.93 (br m, 1H), δ 4.04 (d, *J* = 6.4 Hz, 1H), 3.89 (s, 1H), 3.64 (t, *J* = 5.6 Hz, 2H), 3.53 – 3.43 (m, 3H), 3.42 – 3.35 (m, 2H), 3.34 (d, *J* = 2.0 Hz, 1H), 3.02 (t, *J* = 6.6 Hz, 2H), 2.75 – 2.67 (m, 2H), 2.41 (t, *J* = 6.6 Hz, 2H), 1.61 – 1.37 (m, 6H), 0.92 (s, 6H), 0.90 (s, 9H), 0.06 (s, 6H). **^{13}C{^{1}H} NMR** (75 MHz, MeOD) δ 198.8, 176.0, 173.9, 77.4, 70.4, 69.5, 64.2, 52.6, 40.4, 40.0, 37.9, 36.4, 36.3, 33.7, 29.3, 26.4, 23.0, 21.3, 21.0, 19.2, -5.2. The NMR data match with those obtained for the **6j** The NMR data match with those obtained for the pseudoenantiomeric **6k** (see section 3.4.11 above).

3.6.11 Synthesis of S-(2-(3-((*R*)-2,4-dihydroxy-3,3-dimethylbutanamido)propanamido)ethyl) 3-hydroxy-5-phenylpentanethioate (**6’k**), OJJ-158.

The title compound **6’k** was prepared following the reaction sequences as shown in Scheme S31.

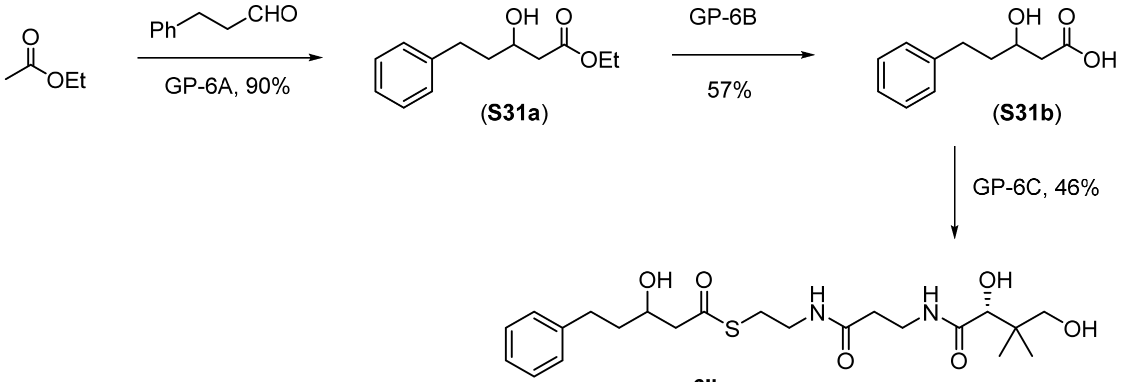

Scheme S31. Synthesis of **6'k** from 3-phenylpropanal.

3.6.11.1 Synthesis of ethyl 3-hydroxy-5-phenylpentanoate (**S31a**), OJJ-153.

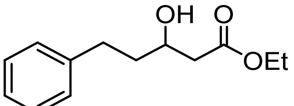

(**S31a**)

3-hydroxy ester (**S31a**) was prepared from 3-phenylpropanal (0.34 g, 2.5 mmol) following GP-6A providing **S31a** (0.5 g, 90%) as a colorless liquid and this intermediate was used in the next step without further purification.

Synthesis of 3-hydroxy-5-phenylpentanoic acid (**S31b**), OJJ-155.

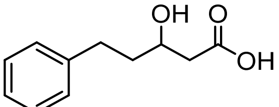

(**S31b**)

3-hydroxy acid (**S31b**) was prepared from corresponding ester **S31a** (0.5 g, 2.3 mmol) following GP-6B providing **S31b** (0.25 g, 57%) as a colorless liquid, characterized by NMR and this intermediate was used in the next step without further purification. The spectral data matches with the literature reported data.[43]

^{1}H NMR (500 MHz, MeOD) δ 7.28 – 7.23 (m, 2H), 7.23 – 7.18 (m, 2H), 7.17 – 7.12 (m, 1H), 4.00 (tt, *J* = 8.1, 4.7 Hz, 1H), 2.79 (ddd, *J* = 13.6, 9.8, 5.6 Hz, 1H), 2.66 (ddd, *J* = 13.6, 9.8, 6.8 Hz, 1H), 2.52 – 2.37 (m, 2H), 1.90 – 1.69 (m, 2H). **^{13}C{^{1}H} NMR** (126 MHz, MeOD) δ 175.5, 143.3, 129.4, 129.4, 126.8, 68.7, 43.2, 40.0, 32.9.

3.6.11.2 *S*-(2-(3-((*R*)-2,4-dihydroxy-3,3-dimethylbutanamido)propanamido)ethyl) 3-hydroxy-5-phenylpentanethioate (**6’k**), OJJ-158.

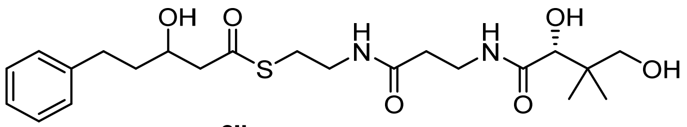

6'k

The (3*RS*)-hydroxy thioester **6’k** was prepared from corresponding 3-hydroxy acid **S31b** (0.12 g, 0.6 mmol) *via* coupling reaction following GP-6C providing **6’k** (0.13 g, 46%) as a colorless sticky liquid.

$\boldsymbol{R}_{\mathbf{f}}$ = 0.55 in 10% MeOH/DCM.

^{1}H NMR (300 MHz, MeOD) δ 8.13 (br m, 1H), 7.92 (br m, 1H), 7.30 – 7.09 (m, 5H), 4.85 (s, 1H, O**H**), 4.04 (p, *J* = 6.3 Hz, 1H), 3.90 (s, 1H), 3.52 – 3.41 (m, 3H), 3.41 – 3.35 (m, 2H), 3.35 – 3.29 (m, 1H), 3.02 (t, *J* = 6.6 Hz, 2H), 2.85 – 2.57 (m, 4H), 2.40 (t, *J* = 6.6 Hz, 2H), 1.77 (dddd, *J* = 9.3, 7.3, 5.4, 3.5 Hz, 2H), 0.92 (s, 6H). **^{13}C{^{1}H} NMR** (75 MHz, MeOD) δ 198.8, 176.0, 173.9, 143.2, 129.4, 129.4, 126.8, 77.4, 70.4, 68.9, 52.6, 40.4, 39.98, 39.96, 36.4, 36.3, 32.8, 29.3, 21.3, 20.95. The NMR data match with those obtained for the **6k** (see section 3.4.12 above).

3.6.11.3 Synthesis of *S*-(2-acetamidoethyl) 3-hydroxyhexanethioate (**7’a**), OJJ-201.

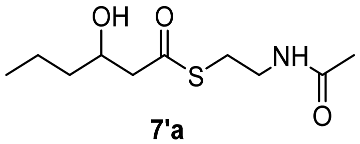

7'a

The (3*RS*)-hydroxy thioester **7’a** was prepared from corresponding 3-hydroxy acid **S19b** (0.08 g, 0.6 mmol) using HS-NAC (0.6 mmol, 0.07 g) via coupling reaction following GP-6C providing **7’a** (0.11 g, 78%) as a white sticky semisolid compound.

$\boldsymbol{R}_{\mathbf{f}}$ = 0.7 in 10% MeOH/DCM.

^{1}H NMR (300 MHz, MeOD) δ 8.10 (br m, 1H), δ 4.04 (q, *J* = 6.1 Hz, 1H), 3.40 – 3.30 (m, 2H), 3.02 (t, *J* = 6.4 Hz, 2H), 2.76 – 2.60 (m, 2H), 1.92 (s, 3H), 1.56 – 1.33 (m, 4H), 0.94 (q, *J* = 4.5 Hz, 3H). **^{13}C{^{1}H} NMR** (75 MHz, MeOD) δ 198.8, 173.5, 173.4, 69.3, 52.6, 40.3, 40.1, 29.3, 22.5, 19.7, 14.2.

IR (film, cm^{-1}): 3300, 2955, 2927, 2869, 1684, 1639, 1544, 1464, 1433, 1404, 1371, 1356, 1290, 1250,1196, 1127, 1072, 1048, 1025, 1003, 958, 851, 771, 717, 603.

HRMS (ESI$^+$) m/z: $[M+H]^+$ calculated for $[C_{10}H_{20}NO_3S]^+$= 234.1158, found 234.1153, Δ=−2.57 ppm.

4 References

[1] Kiema, T.-R.; Engel, C. K.; Schmitz, W.; Filppula, S. A.; Wierenga, R. K.; Hiltunen, J. K. *Biochemistry* **1999**, *38* (10), 2991–2999.

[2] Schmitz, W.; Fingerhut, R.; Conzelmann, E. *European Journal of Biochemistry* **1994**, *222* (2), 313–323.

[3] Waterson, R. M.; Hill, R. L. *J Biol Chem* **1972**, *247* (16), 5258–5265.

[4] Riddles, P. W.; Blakeley, R. L.; Zerner, B. *Methods Enzymol.* **1983**, *91* (Journal Article), 49–60.

[5] Riddles, P. W.; Blakeley, R. L.; Zerner, B. *Analytical Biochemistry* **1979**, *94* (1), 75–81.

[6] Richard, J. P.; Amyes, T. L.; Reyes, A. C. *Acc Chem Res* **2018**, *51* (4), 960–969.

[7] Daniel, E.; Wierenga, R. K.; Lehtiö, L. *Acta Crystallogr D Struct Biol* **2024**, *80* (Pt 8), 580–587.

[8] Winter, G. *Journal of Applied Crystallogr* **2010**, *43* (1), 186–190.

[9] Vonrhein, C.; Flensburg, C.; Keller, P.; Sharff, A.; Smart, O.; Paciorek, W.; Womack, T.; Bricogne, G. *Acta Crystallogr D Biol Crystallogr* **2011**, *67* (4), 293–302.

[10] Ursby, T.; Åhnberg, K.; Appio, R.; Aurelius, O.; Barczyk, A.; Bartalesi, A.; Bjelčić, M.; Bolmsten, F.; Cerenius, Y.; Doak, R. B.; Eguiraun, M.; Eriksson, T.; Friel, R. J.; Gorgisyan, I.; Gross, A.; Haghighat, V.; Hennies, F.; Jagudin, E.; Norsk Jensen, B.; Jeppsson, T.; Kloos, M.; Lidon-Simon, J.; De Lima, G. M. A.; Lizatovic, R.; Lundin, M.; Milan-Otero, A.; Milas, M.; Nan, J.; Nardella, A.; Rosborg, A.; Shilova, A.; Shoeman, R. L.; Siewert, F.; Sondhauss, P.; Talibov, V. O.; Tarawneh, H.; Thånell, J.; Thunnissen, M.; Unge, J.; Ward, C.; Gonzalez, A.; Mueller, U. *J Synchrotron Rad* **2020**, *27* (5), 1415–1429.

[11] Tickle, I.J.; Flensburg, C.; Keller, P.; Paciorek, W.; Sharff, A.,; Vonrhein, C.,; Bricogne, G. Cambridge, United Kingdom: Global Phasing Ltd. **2018**.

[12] Evans, P. R.; Murshudov, G. N. *Acta Crystallogr D Struct Biol* **2013**, *69* (Pt 7), 1204–1214.

[13] McCoy, A. J.; Grosse-Kunstleve, R. W.; Adams, P. D.; Winn, M. D.; Storoni, L. C.; Read, R. J. *Journal of Applied Crystallography* **2007**, *40* (Pt 4), 658–674.

[14] Vagin, A.; Teplyakov, A. *Acta Crystallogr D Biol Crystallogr* **2010**, *66* (Pt 1), 22–25.

[15] Emsley, P.; Lohkamp, B.; Scott, W. G.; Cowtan, K. *Acta Crystallogr D Biol Crystallogr* **2010**, *66* (Pt 4), 486–501.

[16] Liebschner, D.; Afonine, P. V.; Baker, M. L.; Bunkoczi, G.; Chen, V. B.; Croll, T. I.; Hintze, B.; Hung, L. W.; Jain, S.; McCoy, A. J.; Moriarty, N. W.; Oeffner, R. D.; Poon, B. K.; Prisant, M. G.; Read, R. J.; Richardson, J. S.; Richardson, D. C.; Sammito, M. D.; Sobolev, O. V.; Stockwell, D. H.; Terwilliger, T. C.; Urzhumtsev, A. G.; Videau, L. L.; Williams, C. J.; Adams, P. D. *Acta Crystallogr D Struct Biol* **2019**, *75* (Pt 10), 861–877.

[17] Afonine, P. V.; Grosse-Kunstleve, R. W.; Echols, N.; Headd, J. J.; Moriarty, N. W.; Mustyakimov, M.; Terwilliger, T. C.; Urzhumtsev, A.; Zwart, P. H.; Adams, P. D. *Acta*

Crystallogr D Biol Crystallogr **2012**, *68* (Pt 4), 352–367.
[18] Murshudov, G. N.; Skubák, P.; Lebedev, A. A.; Pannu, N. S.; Steiner, R. A.; Nicholls, R. A.; Winn, M. D.; Long, F.; Vagin, A. A. *Acta Crystallogr D Biol Crystallogr* **2011**, *67* (4), 355–367.
[19] Potterton, L.; Agirre, J.; Ballard, C.; Cowtan, K.; Dodson, E.; Evans, P. R.; Jenkins, H. T.; Keegan, R.; Krissinel, E.; Stevenson, K.; Lebedev, A.; McNicholas, S. J.; Nicholls, R. A.; Noble, M.; Pannu, N. S.; Roth, C.; Sheldrick, G.; Skubak, P.; Turkenburg, J.; Uski, V.; Delft, F. von; Waterman, D.; Wilson, K.; Winn, M.; Wojdyr, M. *Acta Crystallogr D Struct Biol* **2018**, *74* (2), 68–84.
[20] Winn, M. D.; Ballard, C. C.; Cowtan, K. D.; Dodson, E. J.; Emsley, P.; Evans, P. R.; Keegan, R. M.; Krissinel, E. B.; Leslie, A. G.; McCoy, A.; McNicholas, S. J.; Murshudov, G. N.; Pannu, N. S.; Potterton, E. A.; Powell, H. R.; Read, R. J.; Vagin, A.; Wilson, K. S. *Acta Crystallogr D Biol Crystallogr* **2011**, *67* (Pt 4), 235–242.
[21] Williams, C. J.; Headd, J. J.; Moriarty, N. W.; Prisant, M. G.; Videau, L. L.; Deis, L. N.; Verma, V.; Keedy, D. A.; Hintze, B. J.; Chen, V. B.; Jain, S.; Lewis, S. M.; Arendall, W. B.; Snoeyink, J.; Adams, P. D.; Lovell, S. C.; Richardson, J. S.; Richardson, D. C. *Protein Sci* **2018**, *27* (1), 293–315.
[22] Feng, Z.; Westbrook, J. D.; Sala, R.; Smart, O. S.; Bricogne, G.; Matsubara, M.; Yamada, I.; Tsuchiya, S.; Aoki-Kinoshita, K. F.; Hoch, J. C.; Kurisu, G.; Velankar, S.; Burley, S. K.; Young, J. Y. *Structure* **2021**, *29* (4), 393-400.e1.
[23] Smart, O. S.; Horský, V.; Gore, S.; Svobodová Vařeková, R.; Bendová, V.; Kleywegt, G. J.; Velankar, S. *Acta Crystallogr D Struct Biol* **2018**, *74* (3), 237–244.
[24] Smart, O. S.; Womack, T. O.; Sharff, A.; Flensburg, C.; Keller, P.; Paciorek, W.; Vonrhein, C.; Bricogne, G. Grade. https://www.globalphasing.com.
[25] Bell, A. F.; Feng, Y.; Hofstein, H. A.; Parikh, S.; Wu, J.; Rudolph, M. J.; Kisker, C.; Whitty, A.; Tonge, P. J. *Chemistry & Biology* **2002**, *9* (11), 1247–1255.
[26] Krissinel, E.; Henrick, K. *Acta Crystallogr D Biol Crystallogr* **2004**, *60* (12), 2256–2268.
[27] Dalwani, S.; Lampela, O.; Leprovost, P.; Schmitz, W.; Juffer, A,H.; Wierenga, R.K.; Venkatesan R. *J Struct Biol* **2021**, 213(3):107776.
[28] Srivastava, S.; Chaudhary, S.; Thukral, L.; Shi, C.; Gupta, R. D.; Gupta, R.; Priyadarshan, K.; Vats, A.; Haque, A. S.; Sankaranarayanan, R.; Natarajan, V. T.; Sharma, R.; Aldrich, C. C.; Gokhale, R. S. *Chemistry & biology* **2015**, *22* (12), 1577–1587.
[29] Partanen, S. T.; Novikov, D. K.; Popov, A. N.; Mursula, A. M.; Hiltunen, J. K.; Wierenga, R. K. *J Mol Biol* **2004**, *342* (4), 1197–1208.
[30] Hubbard, P. A.; Yu, W.; Schulz, H.; Kim, J.-J. P. *Protein Sci* **2005**, *14* (6), 1545–1555.
[31] Onwukwe, G. U.; Koski, M. K.; Pihko, P., Schmitz, W.; Wierenga, R. K. *Acta Crystallogr D Struct Biol* **2015**, *71* (Pt 11), 2178–2191.
[32] Mursula, A. M.; Hiltunen, J. K.; Wierenga, R. K. *FEBS Lett* **2004**, *557* (1–3), 81–87.
[33] Heine, D.; Sundaram, S.; Beudert, M.; Martin, K.; Hertweck, C. *Chem. Sci.* **2016**, *7* (8), 4848–4855.
[34] Chen, S.-H.; Hong, B.-C.; Su, C.-F.; Sarshar, S. *Tetrahedron Letters* **2005**, *46* (51), 8899–8903.
[35] Marshall, J. A.; Piettre, A.; Paige, M. A.; Valeriote, F. *J. Org. Chem.* **2003**, *68* (5), 1771–1779.

[36] Dunetz, J. R.; Roush, W. R. *Org. Lett.* **2008**, *10* (10), 2059–2062.
[37] Itoh, T.; Miyazaki, M.; Nagata, K.; Ohsawa, A. *Tetrahedron* **2000**, *56* (26), 4383–4395.
[38] Sailer, M.; Dubicki, K.; Sorensen, J. *Synthesis* **2015**, *47* (01), 79–82.
[39] Frankowski, K. J.; Golden, J. E.; Zeng, Y.; Lei, Y.; Aubé, J. *J. Am. Chem. Soc.* **2008**, *130* (18), 6018–6024.
[40] Kaiser, F.; Schwink, L.; Velder, J.; Schmalz, H.-G. *J. Org. Chem.* **2002**, *67,* 26, 9248–9256.
[41] Dreizler, J. K.; Meyners, C.; Hausch, F. *ACS Med. Chem. Lett.* **2024**, *15* (11), 2012–2018.
[42] Reid, B. T.; Mailyan, A. K.; Zakarian, A. *J. Org. Chem.* **2018**, *83* (16), 9492–9496.
[43] Shiina, I.; Umezaki, Y.; Kuroda, N.; Iizumi, T.; Nagai, S.; Katoh, T. *J. Org. Chem.* **2012**, *77* (11), 4885–4901.

5 Copies of NMR spectra

5.1 ^{1}H NMR spectrum of S1b

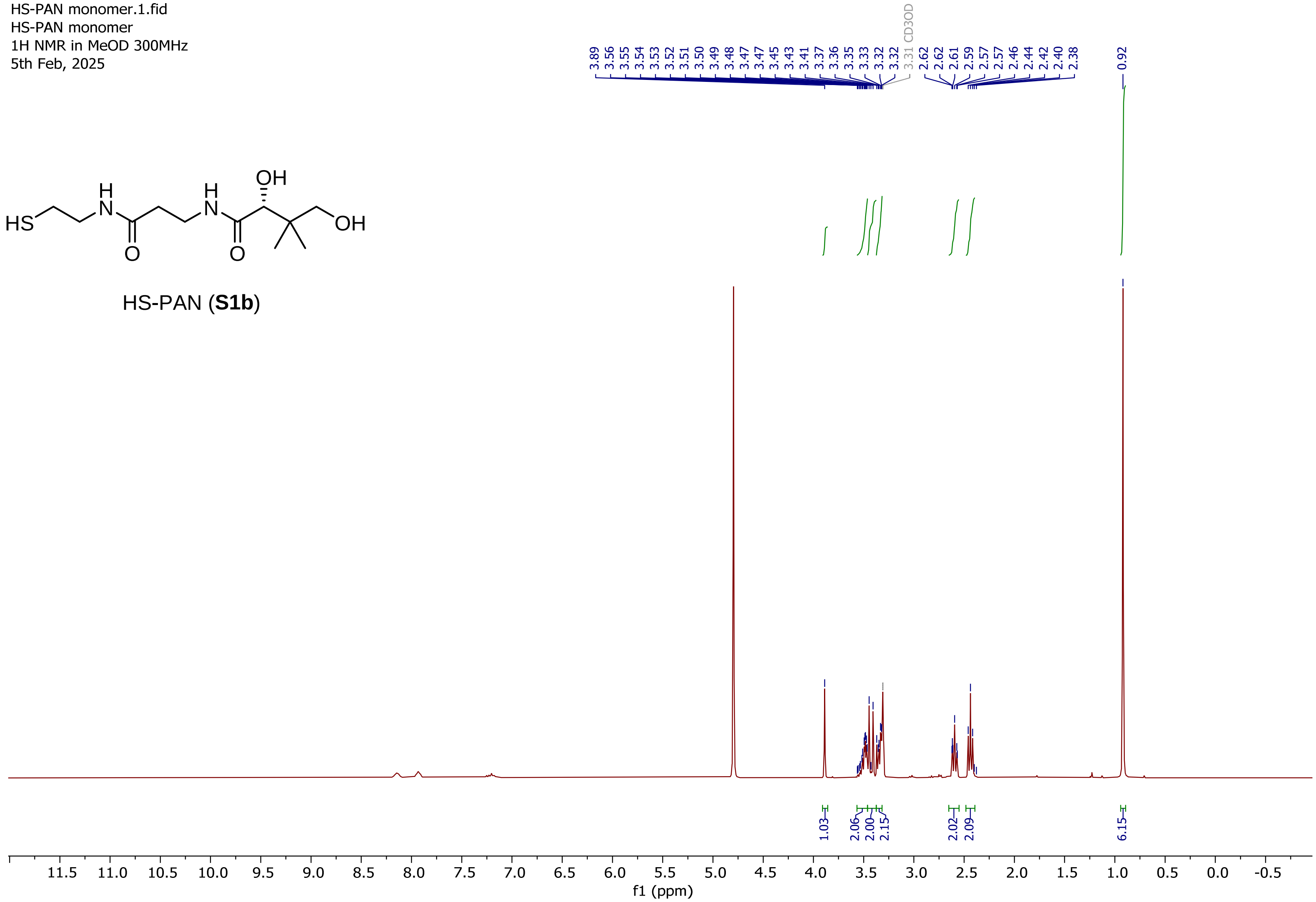

5.2 $^{13}C\{^{1}H\}$ NMR spectrum of S1b

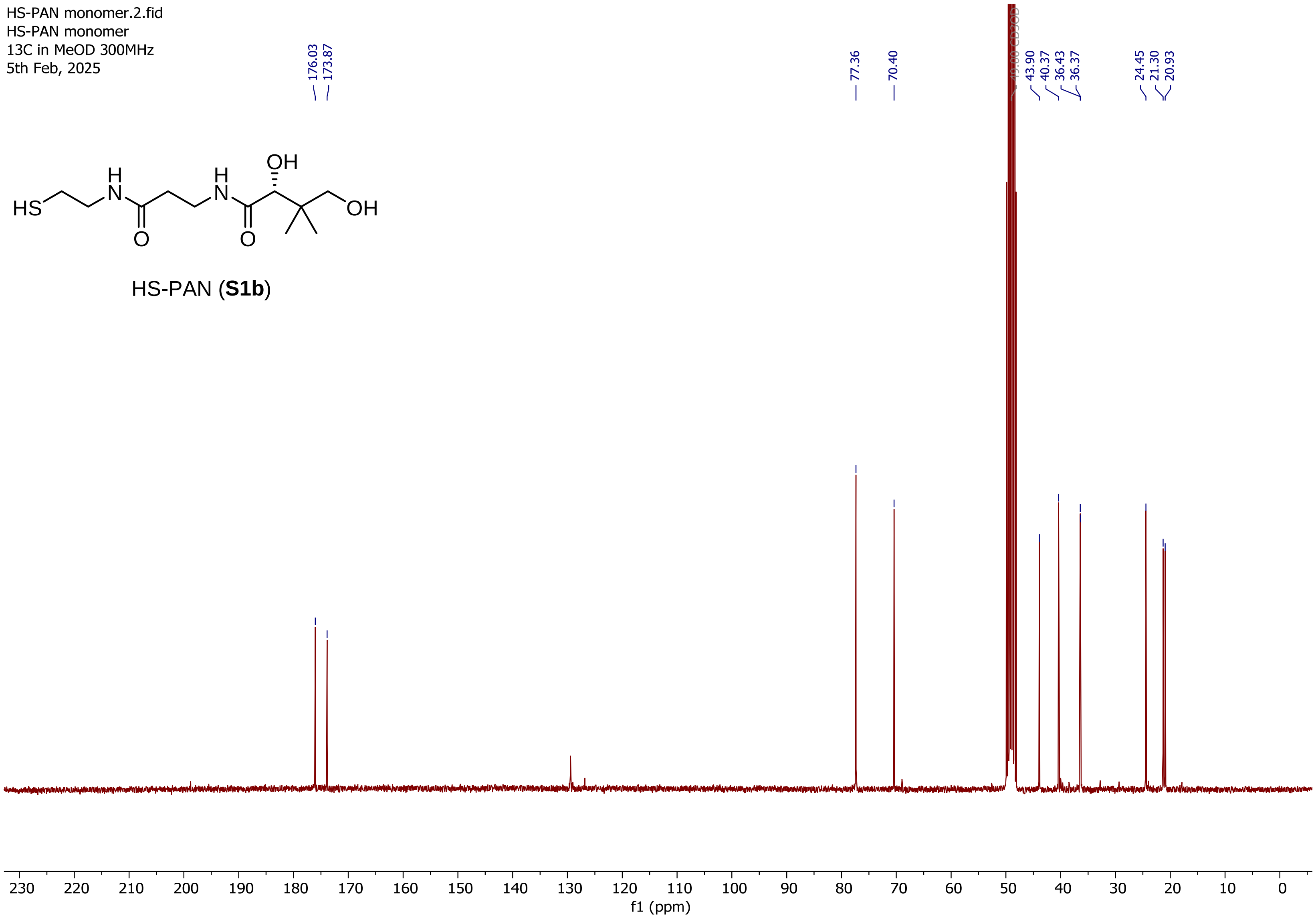

5.3 ¹H NMR spectrum of 3

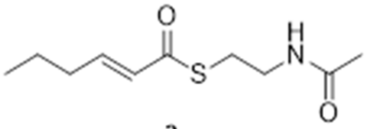

3

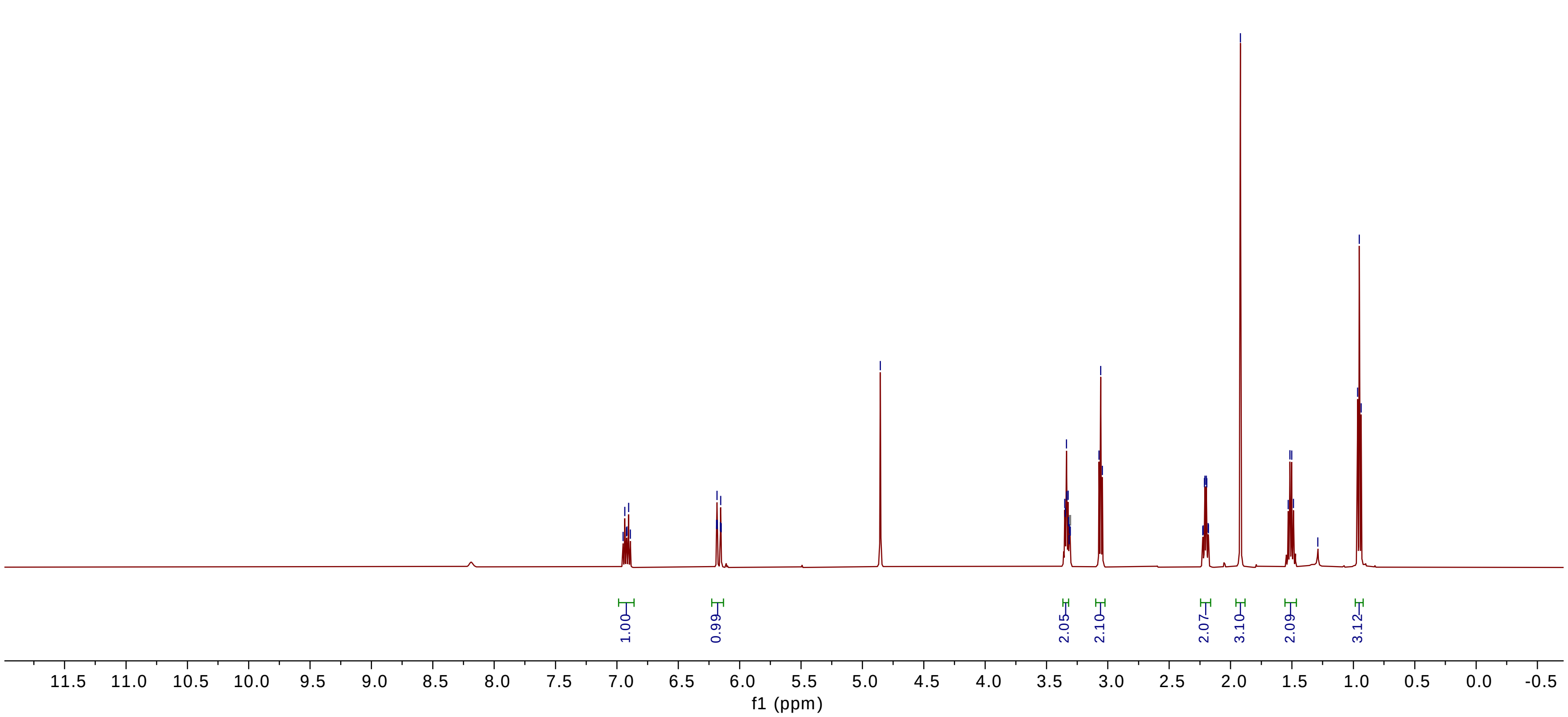

5.4 $^{13}C\{^{1}H\}$ NMR spectrum of 3

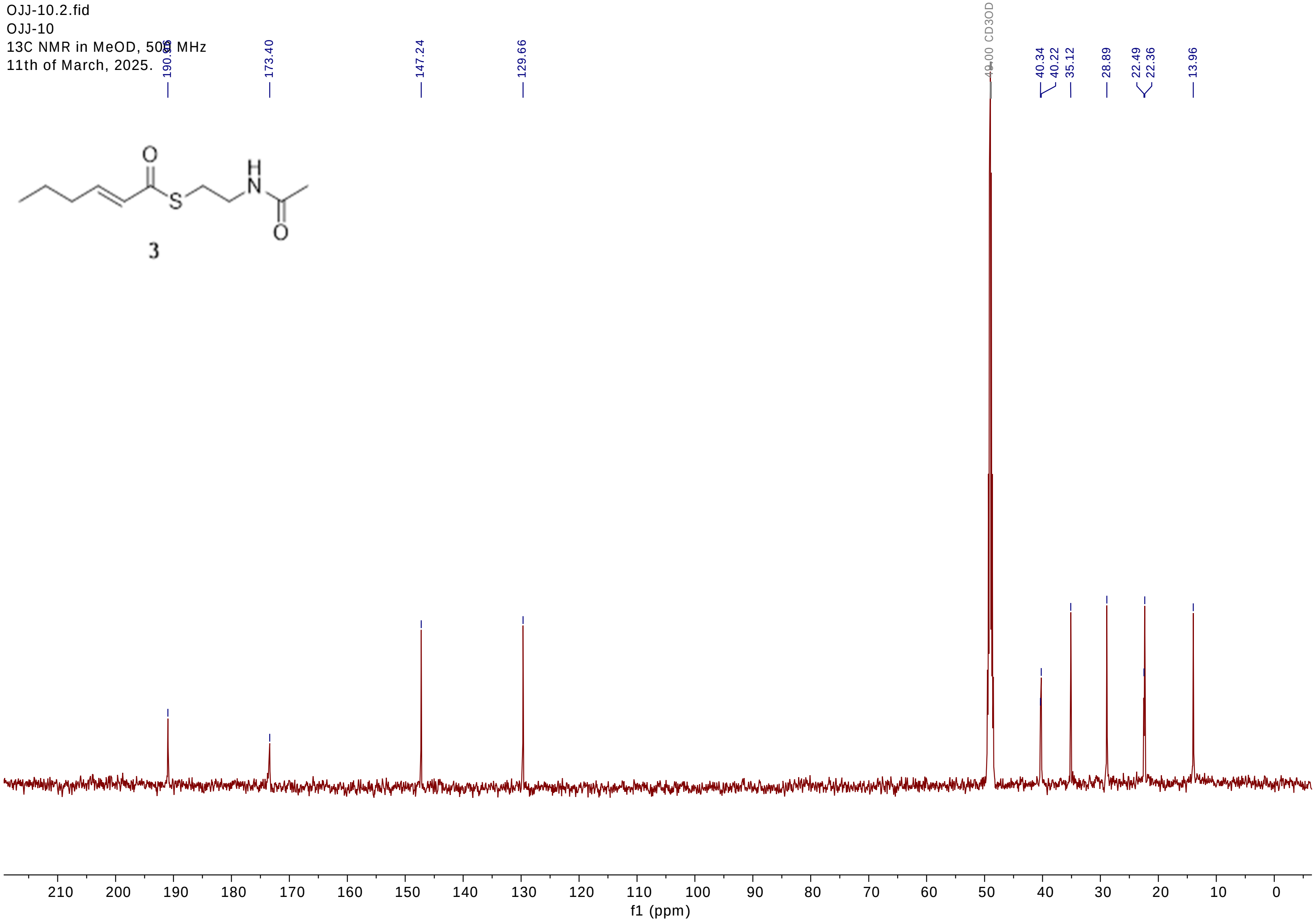

5.5 ^{1}H NMR spectrum of 4a

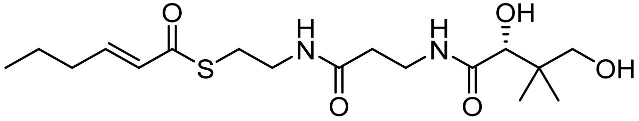

4a

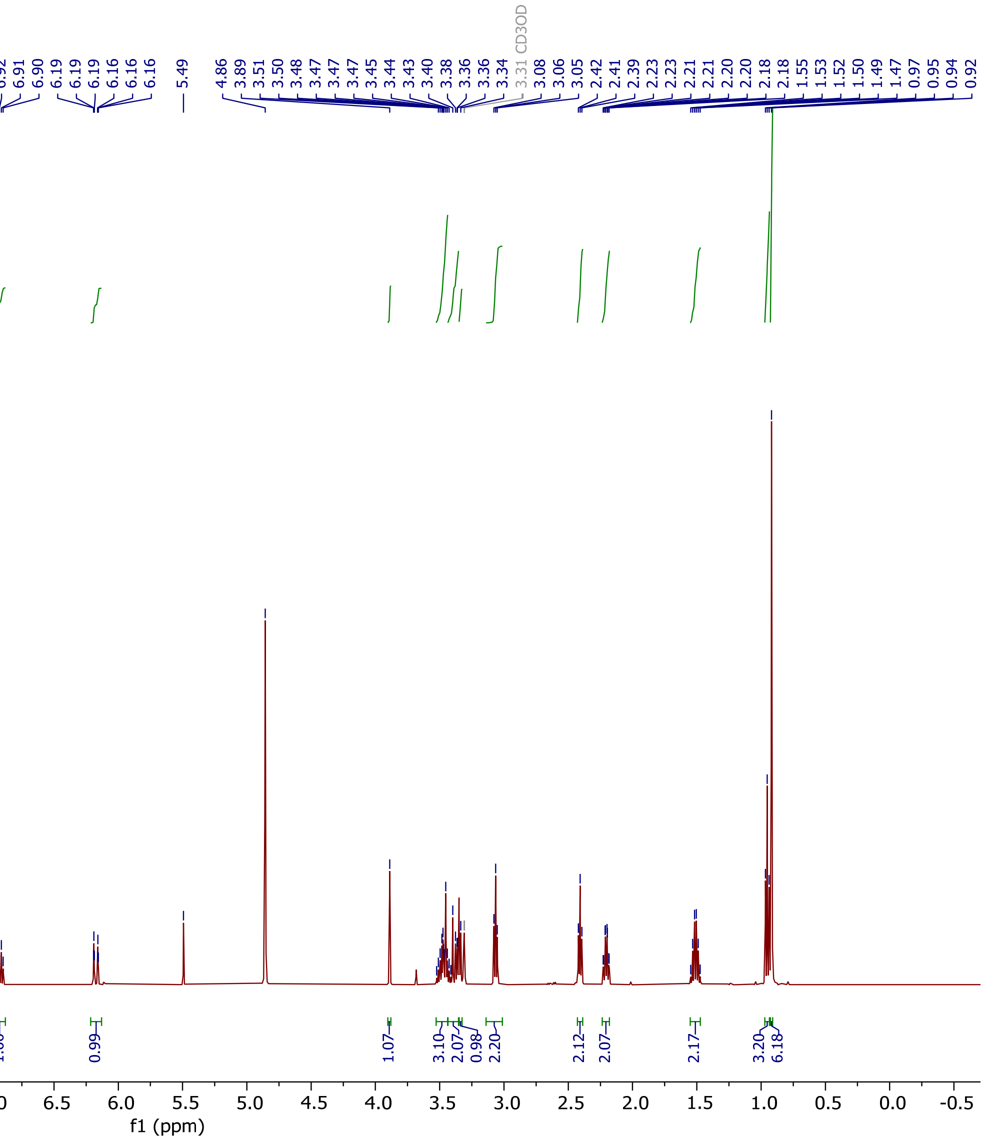

5.6 $^{13}C\{^{1}H\}$ NMR spectrum of 4a

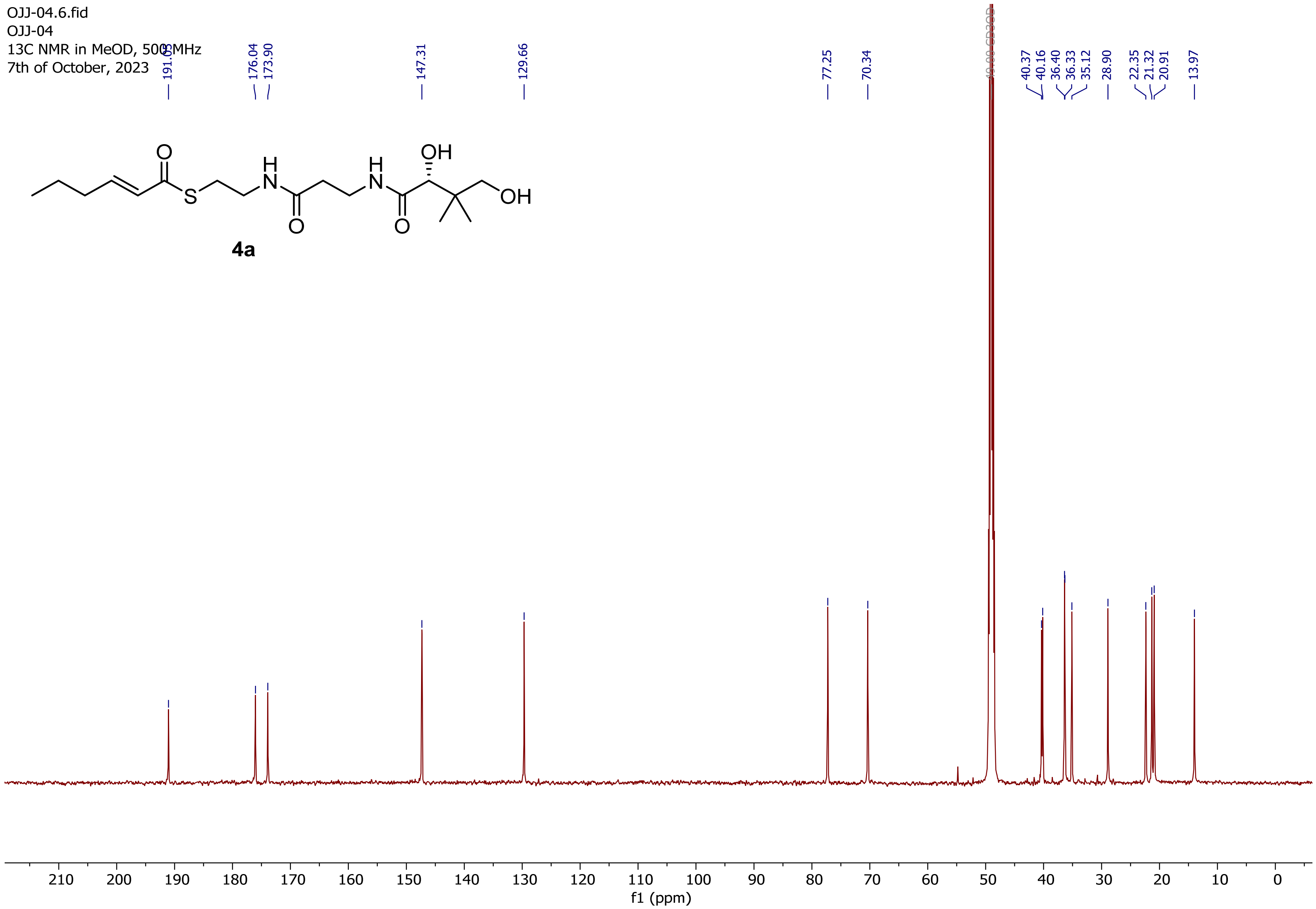

5.7 ^{1}H NMR spectrum of 4b

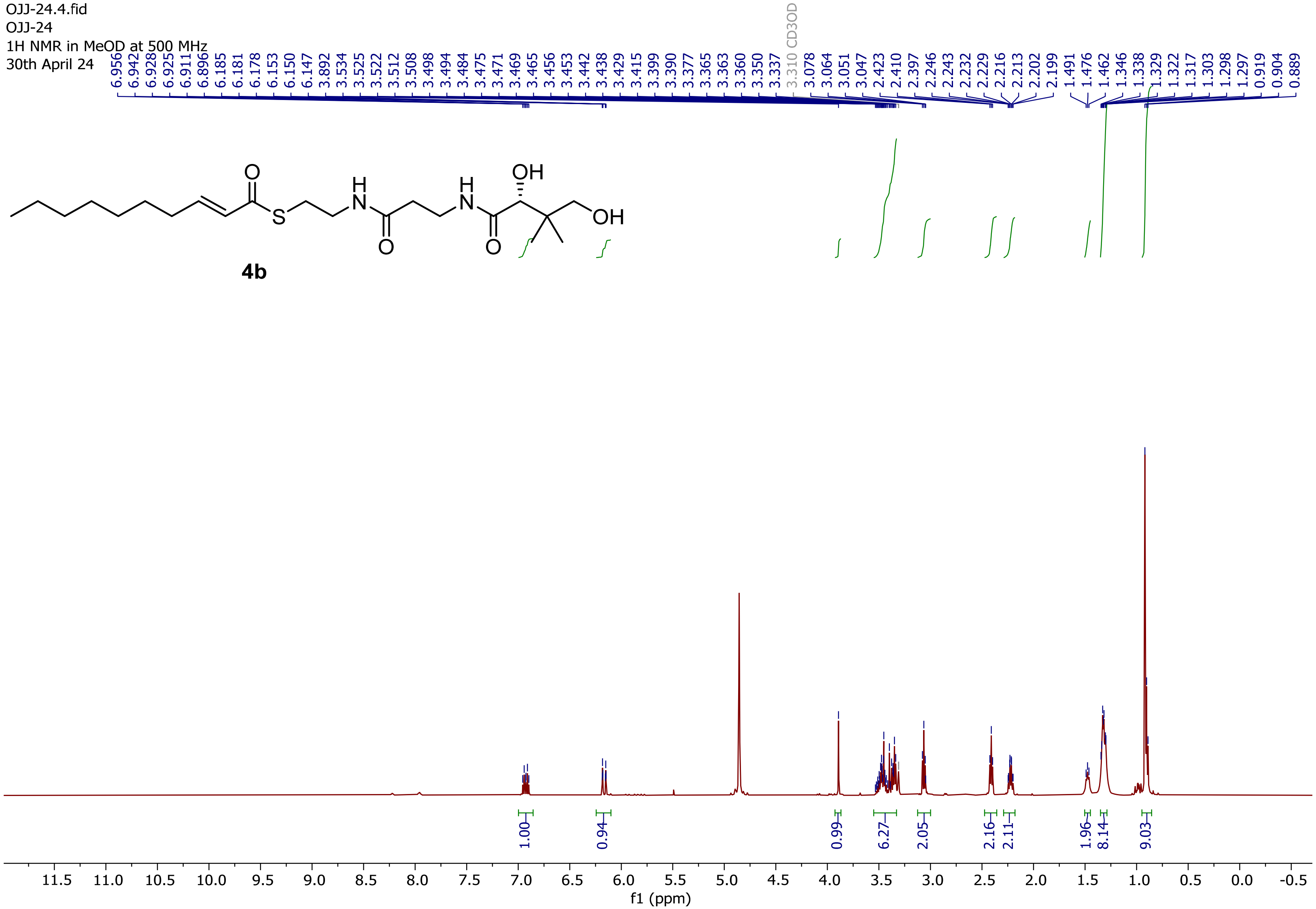

5.8 $^{13}C\{^{1}H\}$ NMR spectrum of 4b

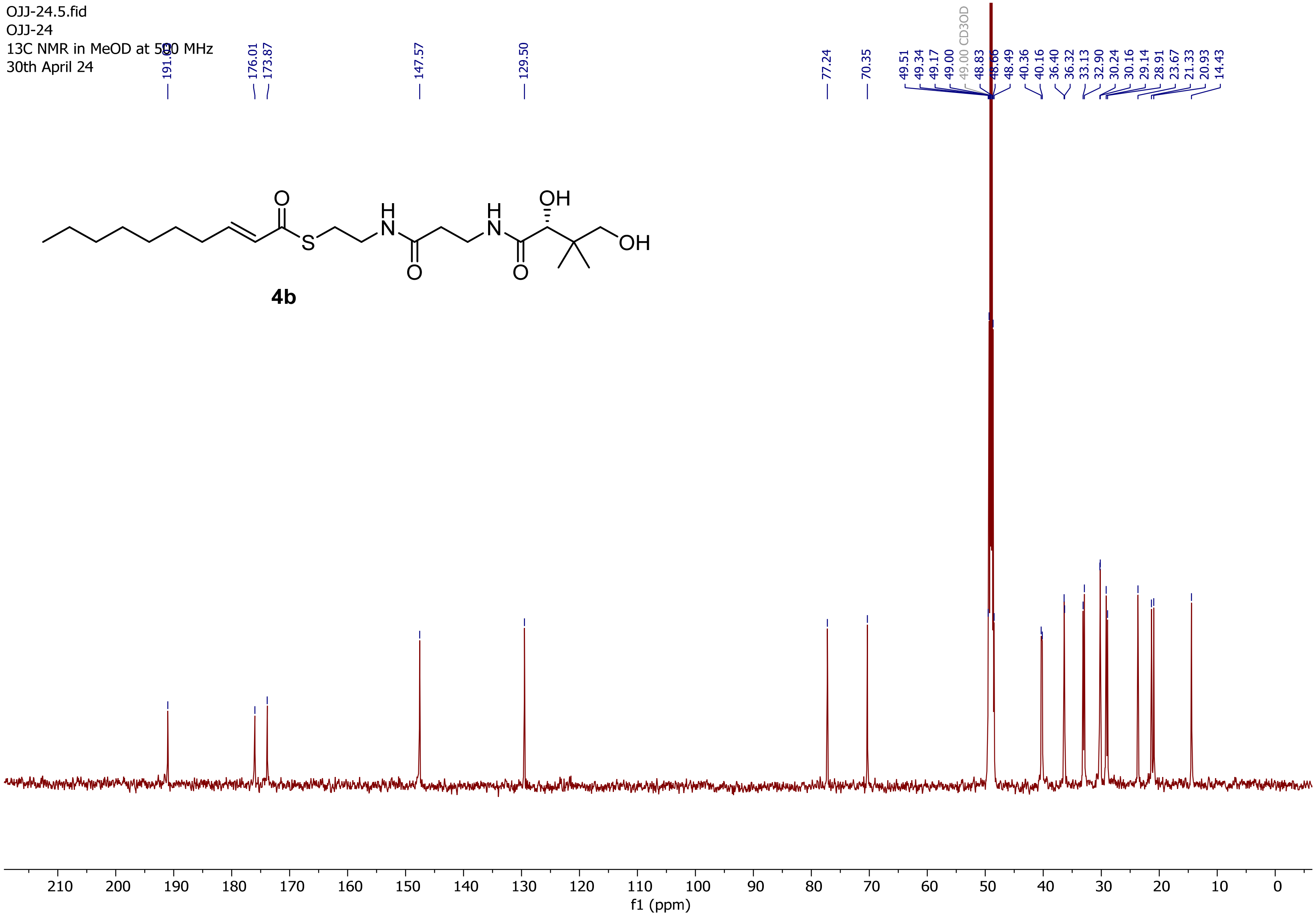

4b

5.9 ^{1}H NMR spectrum of 4c

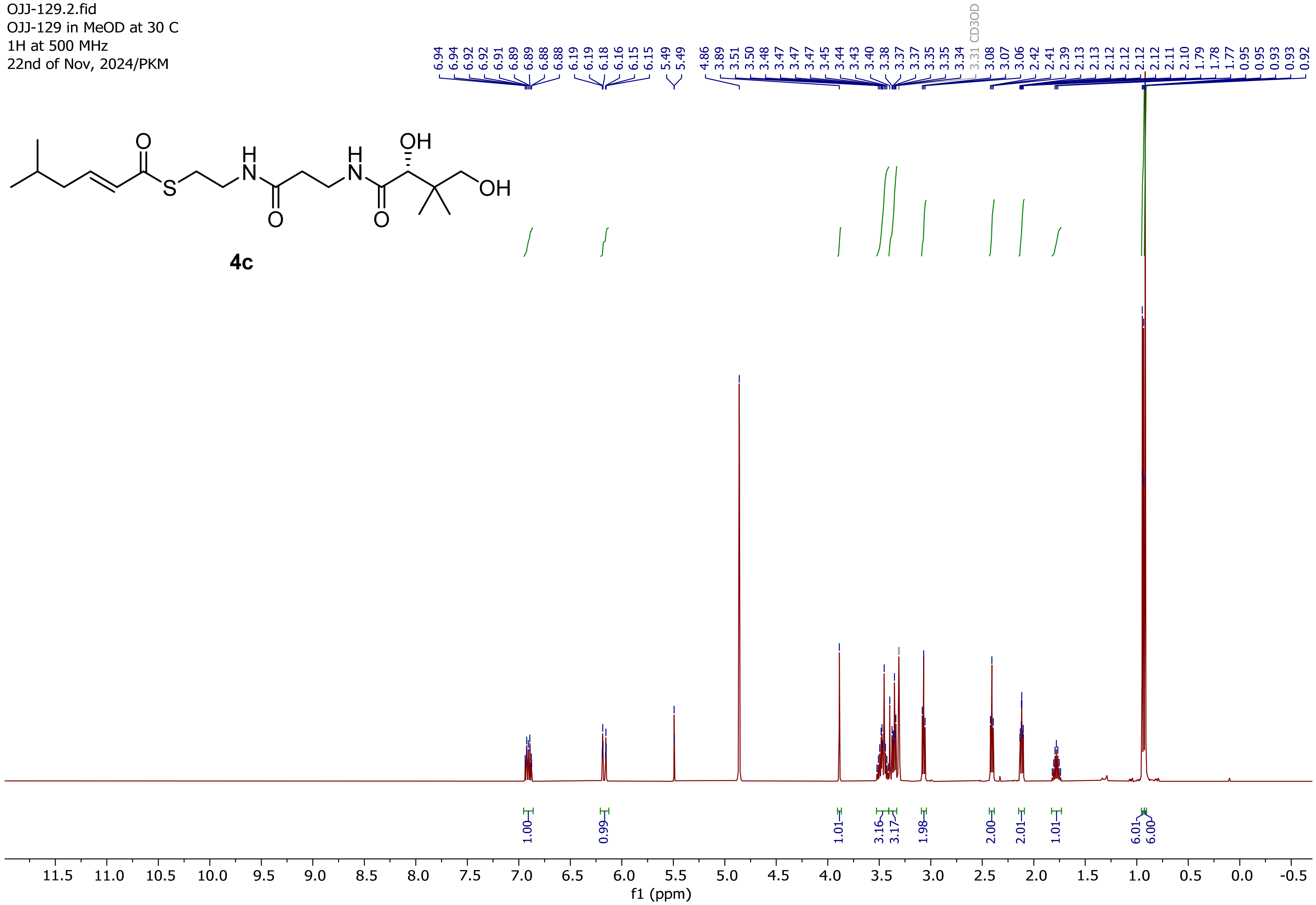

5.10 $^{13}C\{^{1}H\}$ NMR spectrum of 4c

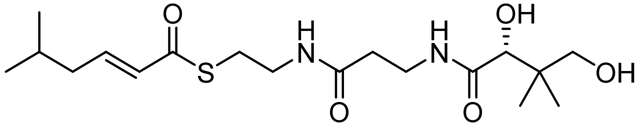

4c

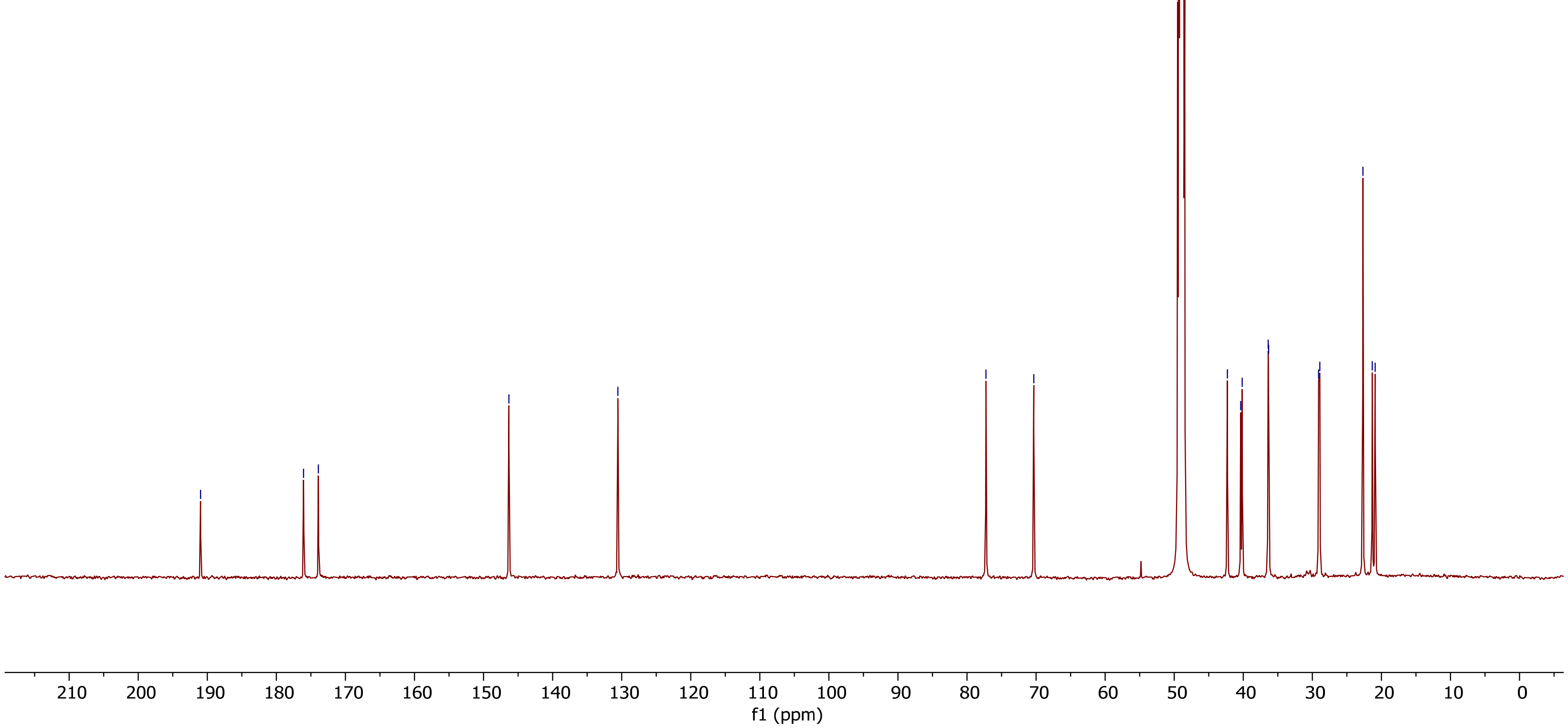

5.11 ^{1}H NMR spectrum of S6b

OJJ-71.2.fid
OJJ-71
in CDCl3, 300MHz
24th March, 2025

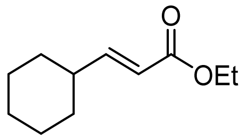

S6b

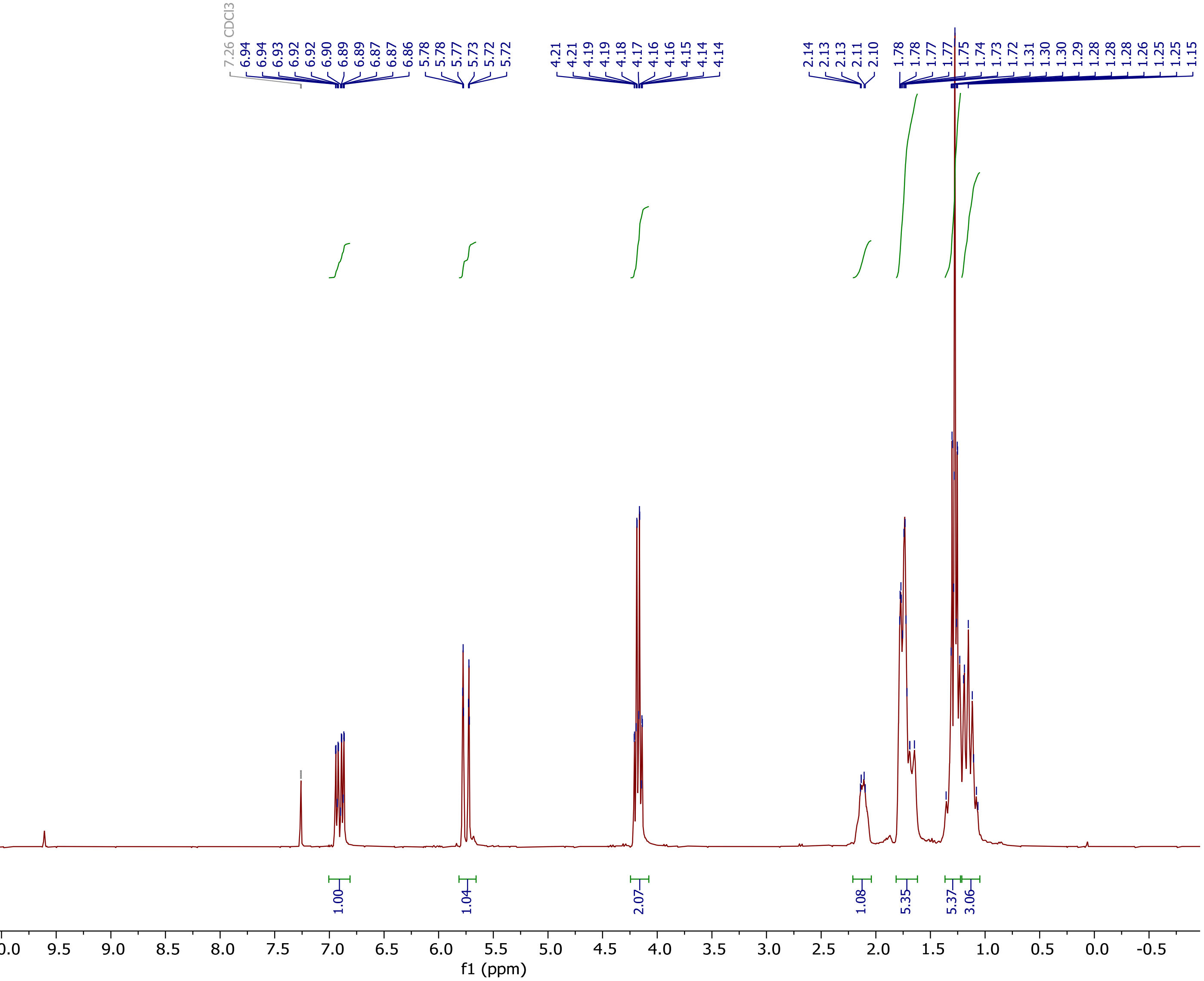

5.12 $^{13}C\{^{1}H\}$ NMR spectrum of S6b

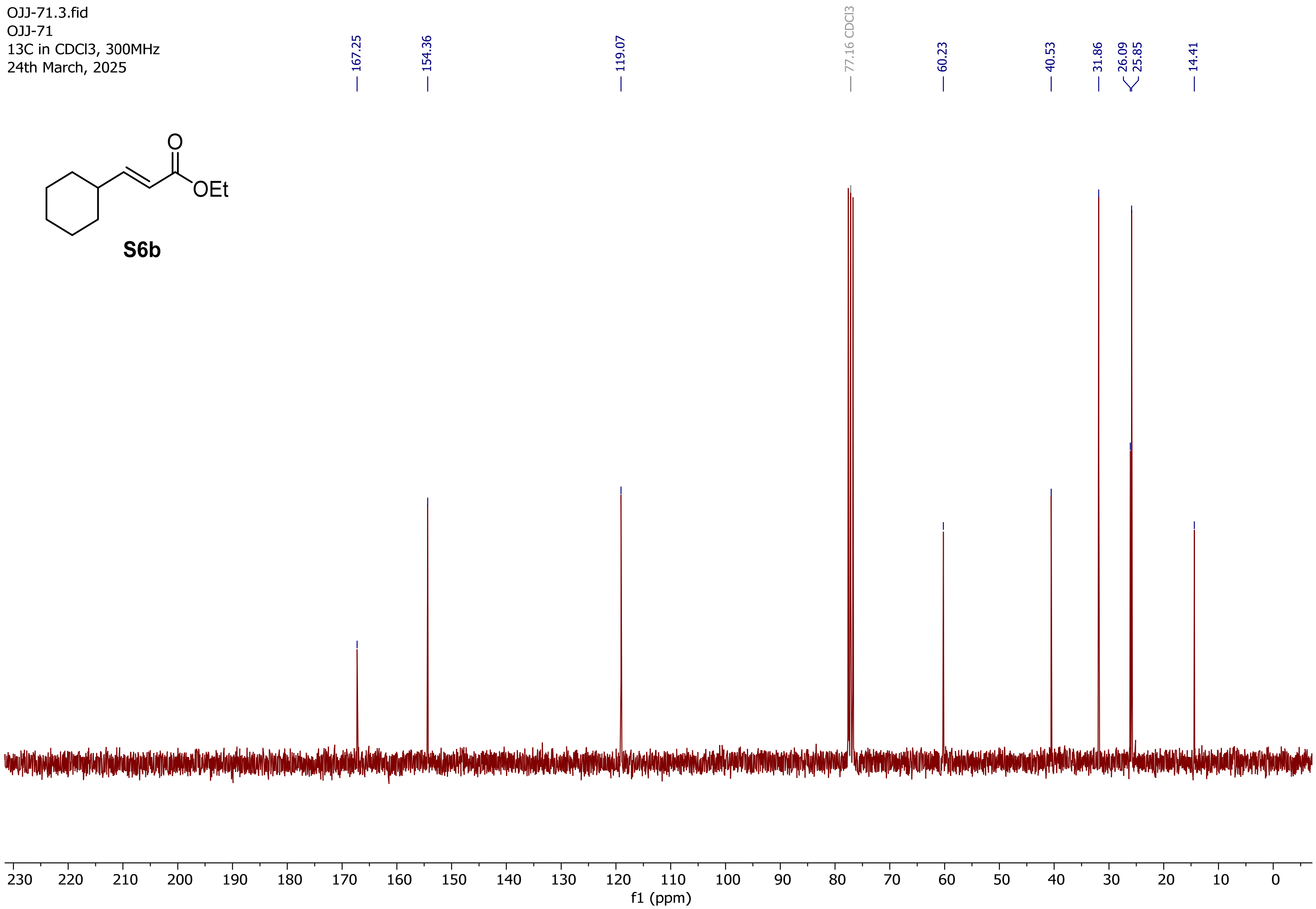

5.13 ^{1}H NMR spectrum of 4d

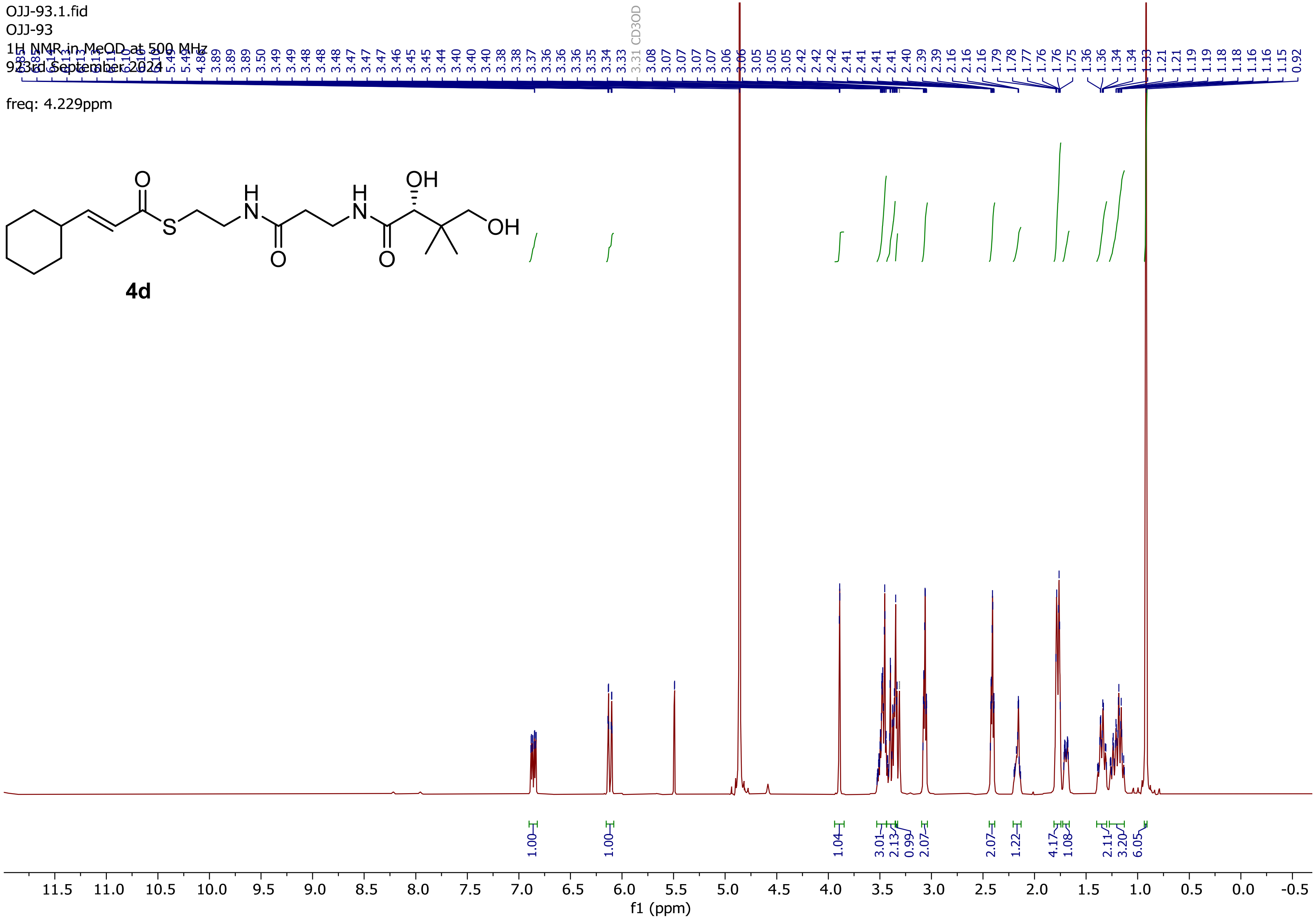

5.14 $^{13}C\{^{1}H\}$ NMR spectrum of 4d

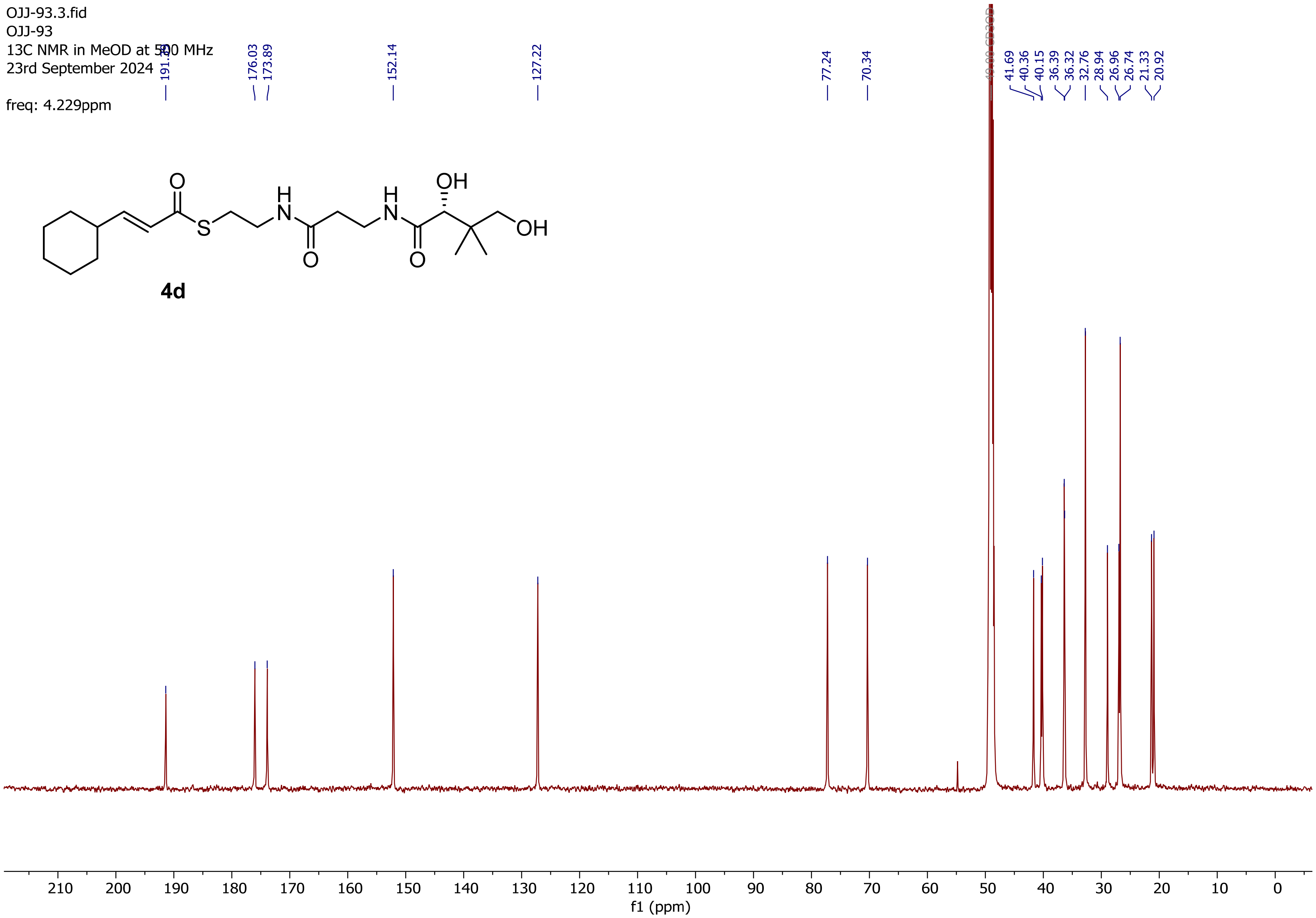

5.15 ^{1}H NMR spectrum of S7b

OJJ-39-Fr-30.1.fid
OJJ-39-Fr-30
1H NMR in CDCl3 at 500 MHz
6th May 2024

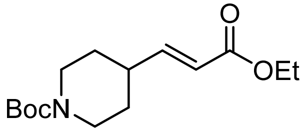

S7b

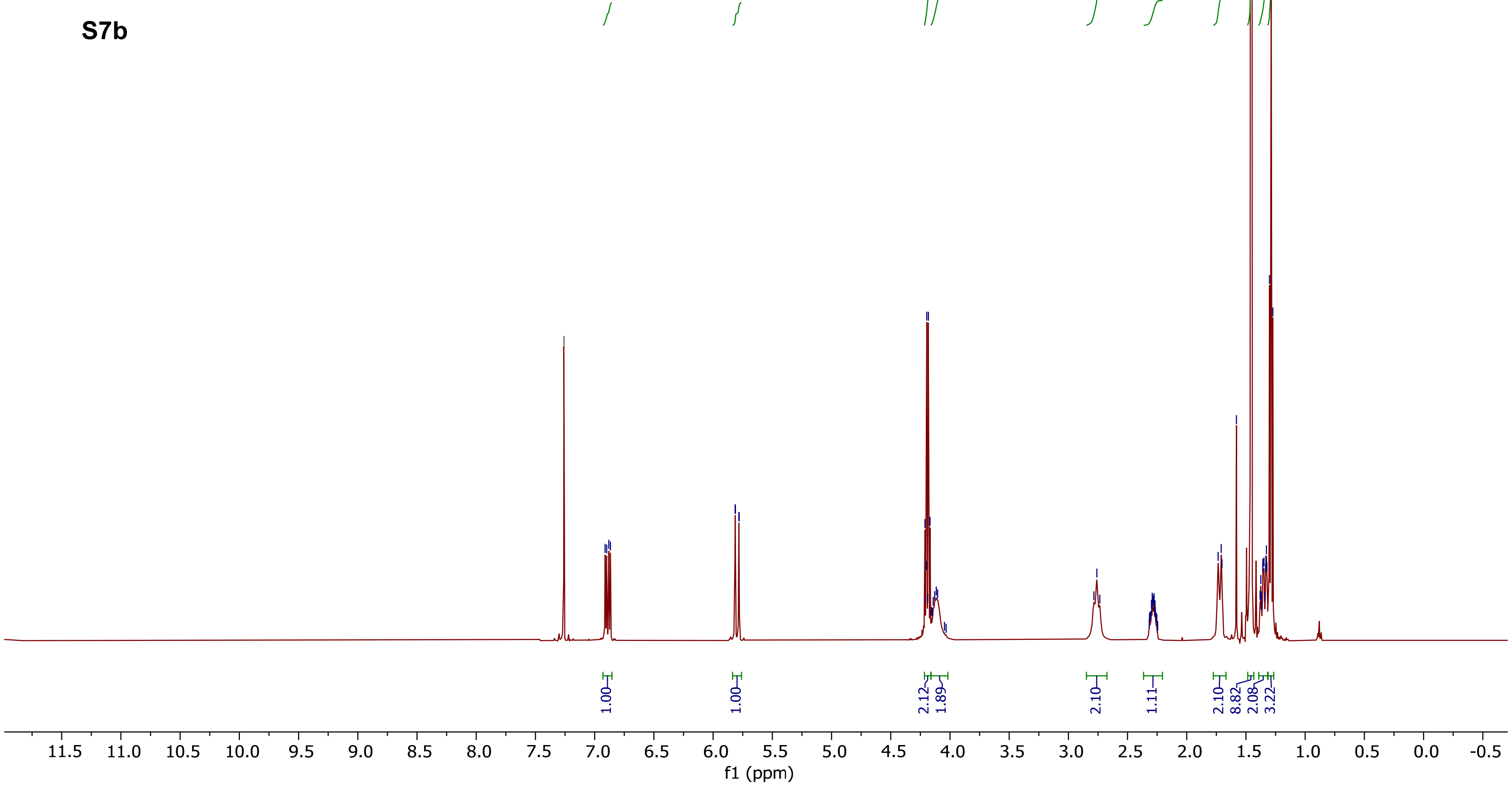

5.16 $^{13}C\{^1H\}$ NMR spectrum of S7b

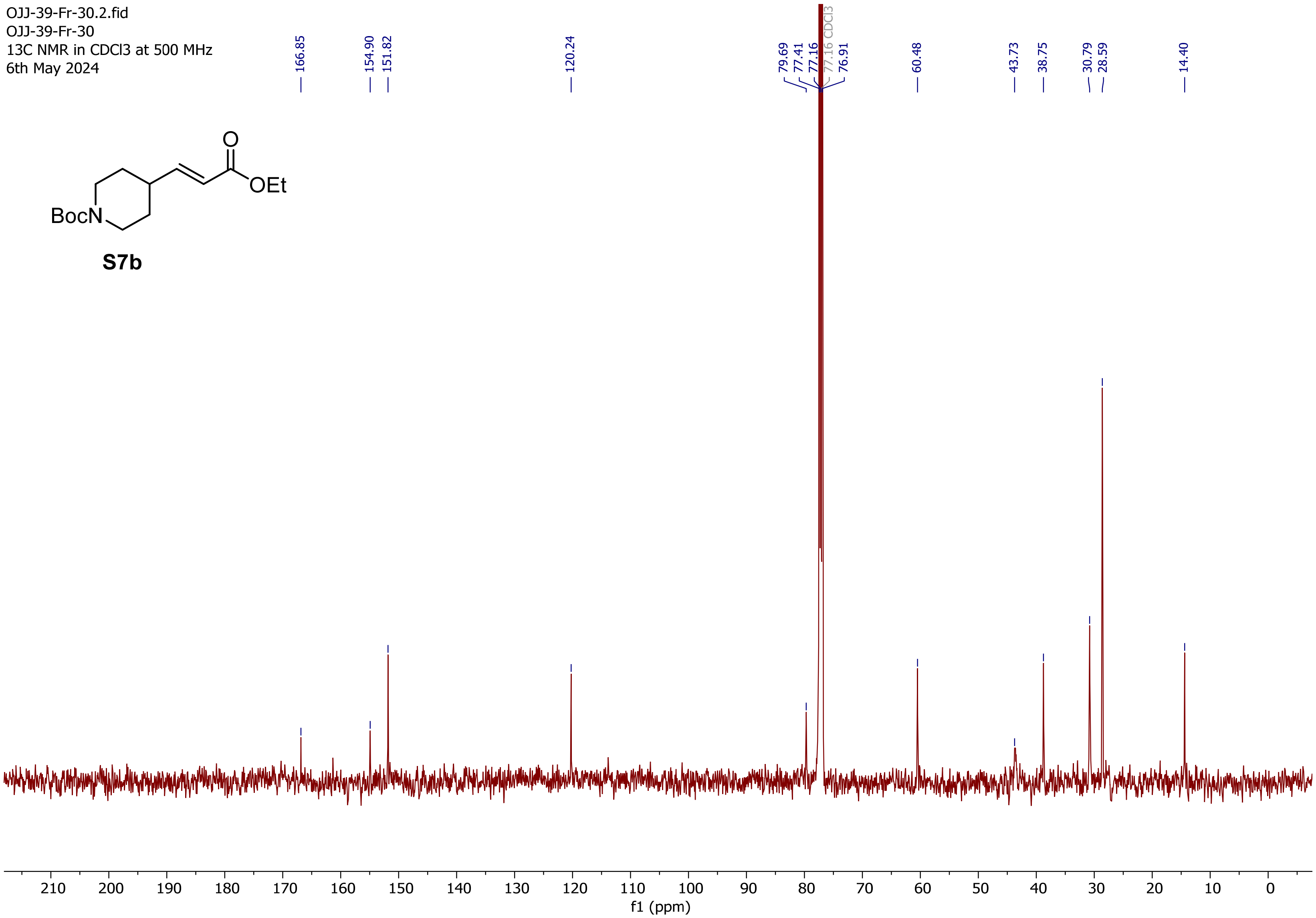

5.17 ^{1}H NMR spectrum of 4e

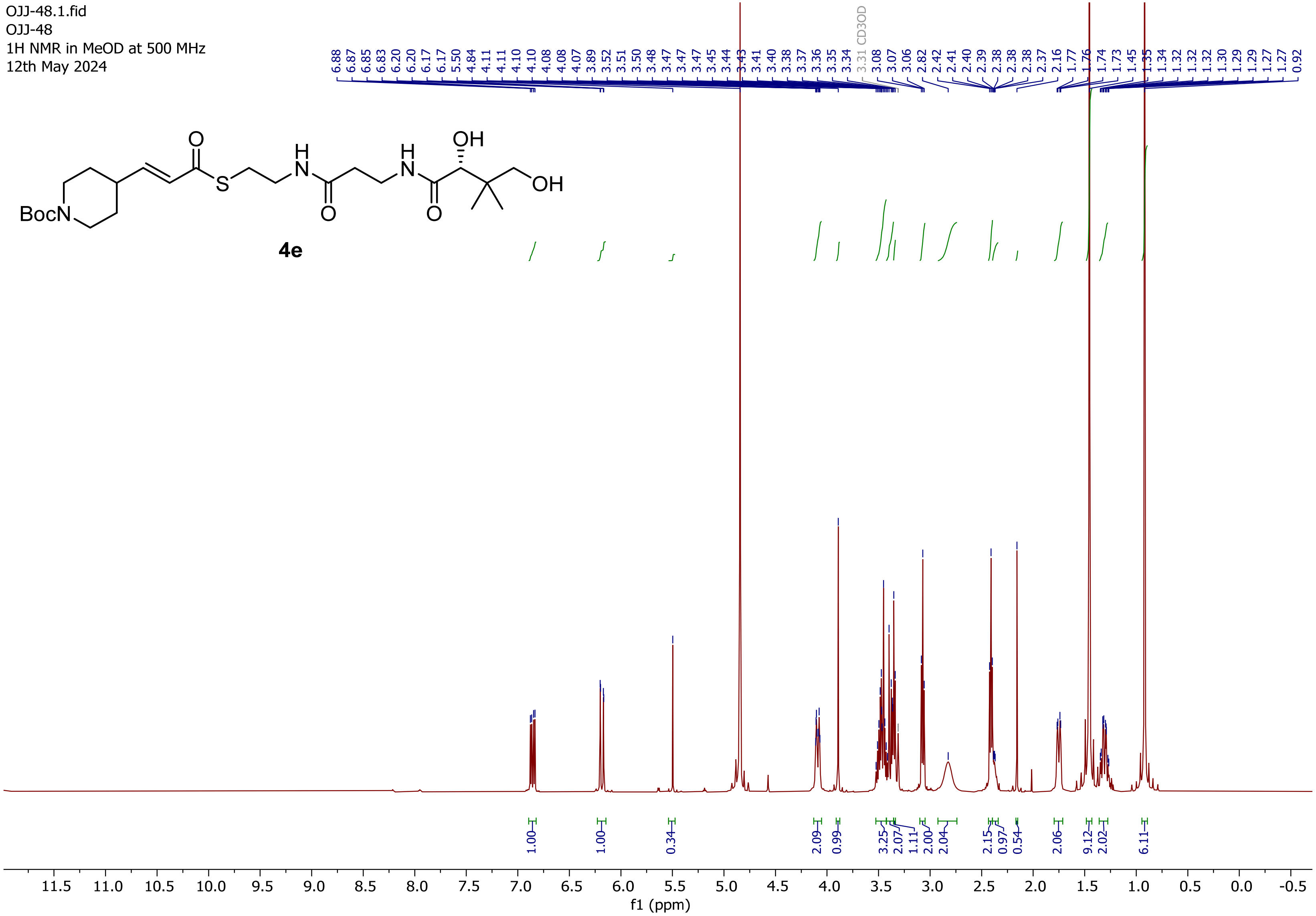

5.18 $^{13}C\{^{1}H\}$ NMR spectrum of 4e

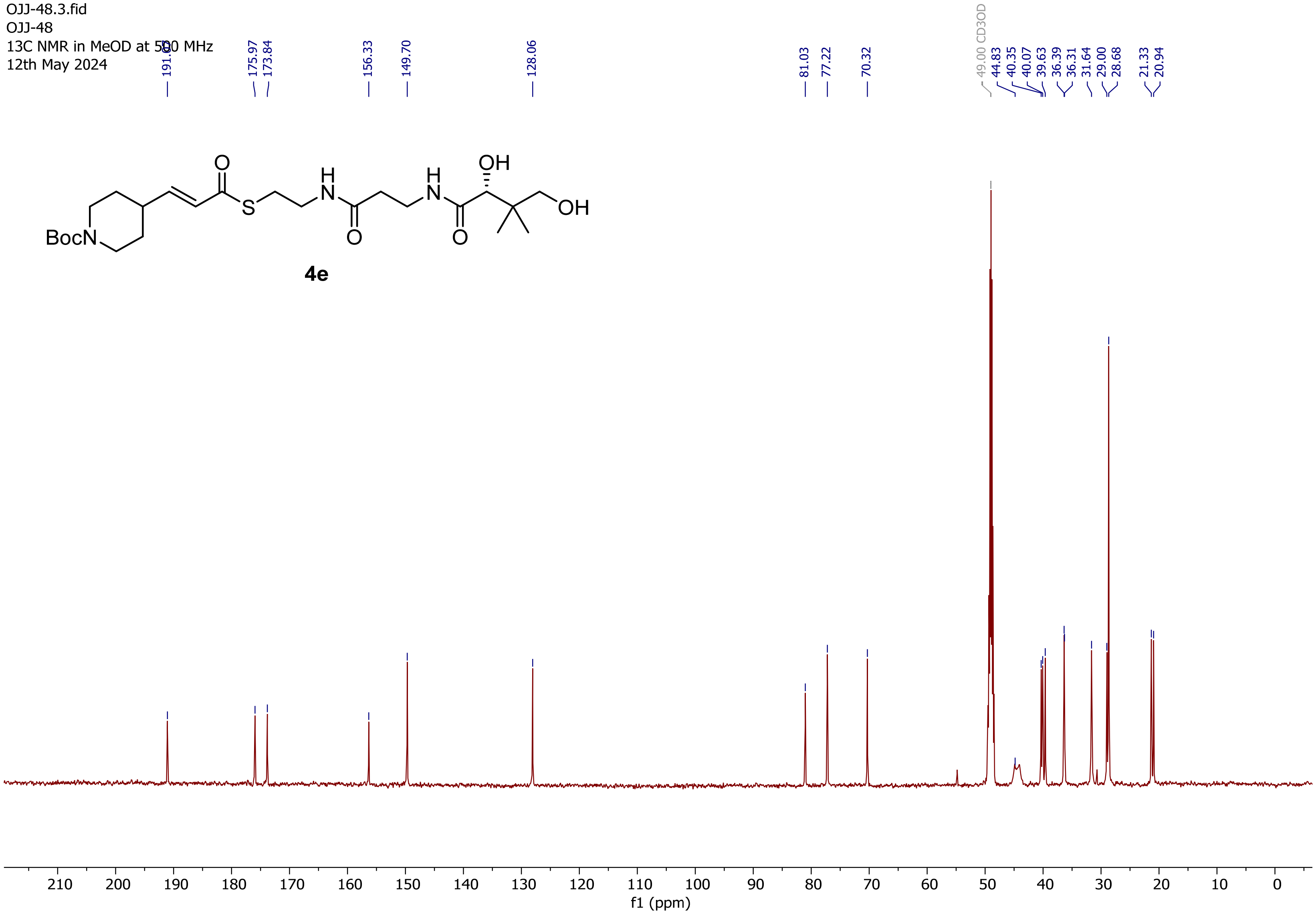

5.19 ¹H NMR spectrum of S8b

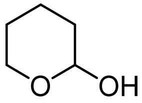

S8b

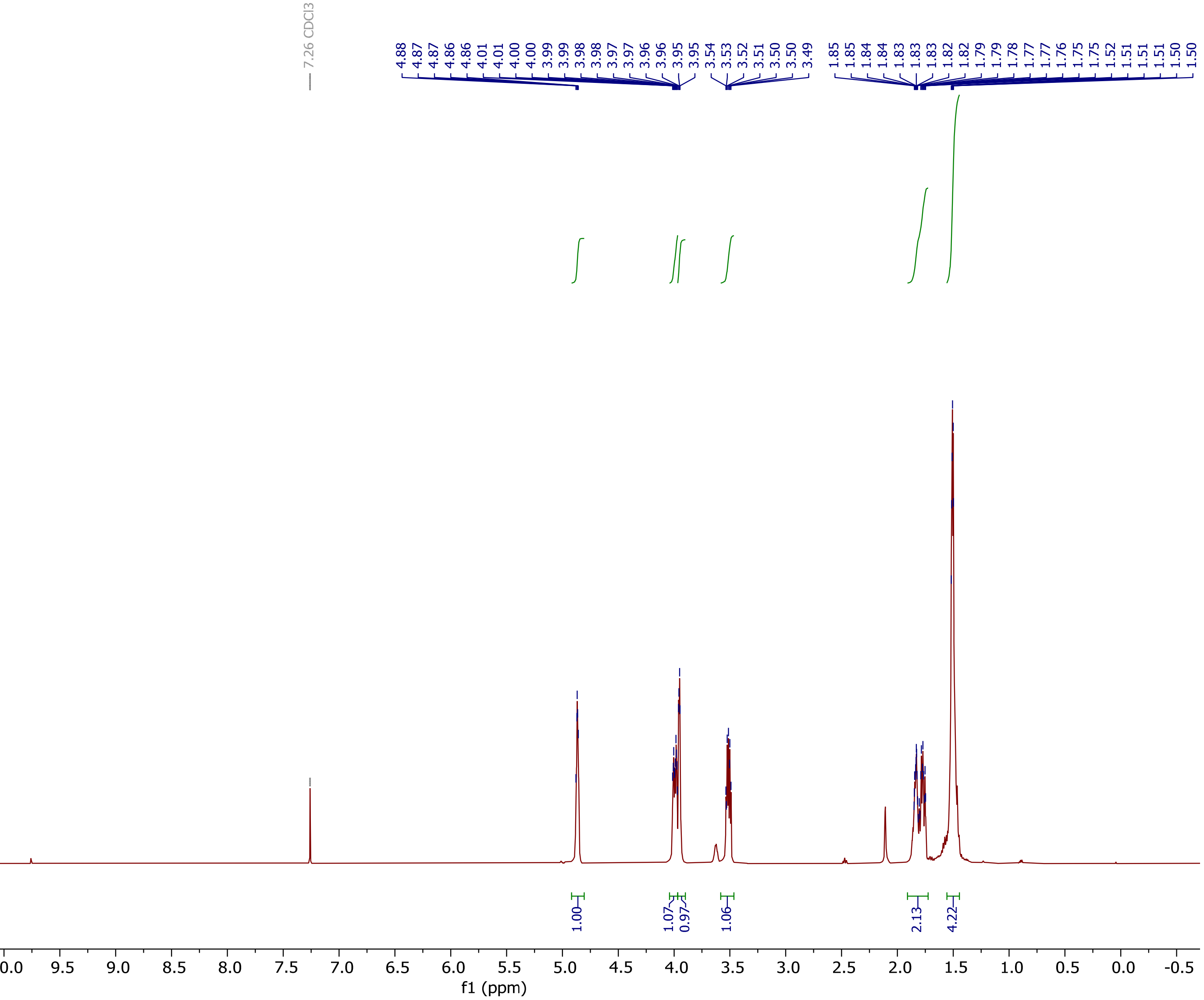

8.20 $^{13}C\{^{1}H\}$ NMR spectrum of S8b

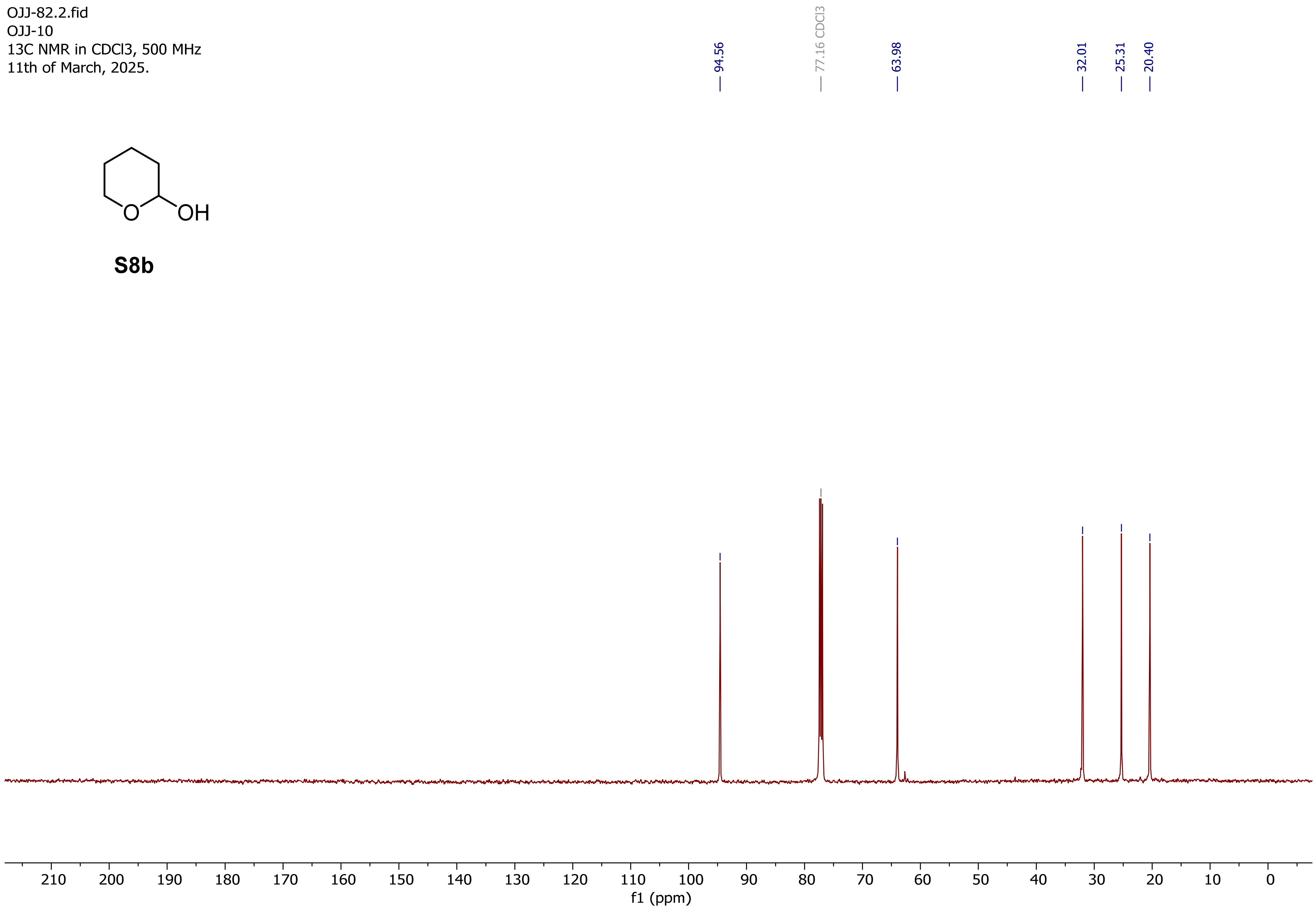

8.21 ^{1}H NMR spectrum of S8c

OJJ-82-wittig.2.fid
OJJ-82-wittig
1H NMR in CDCl3, 500 MHz
11th of March, 2025.

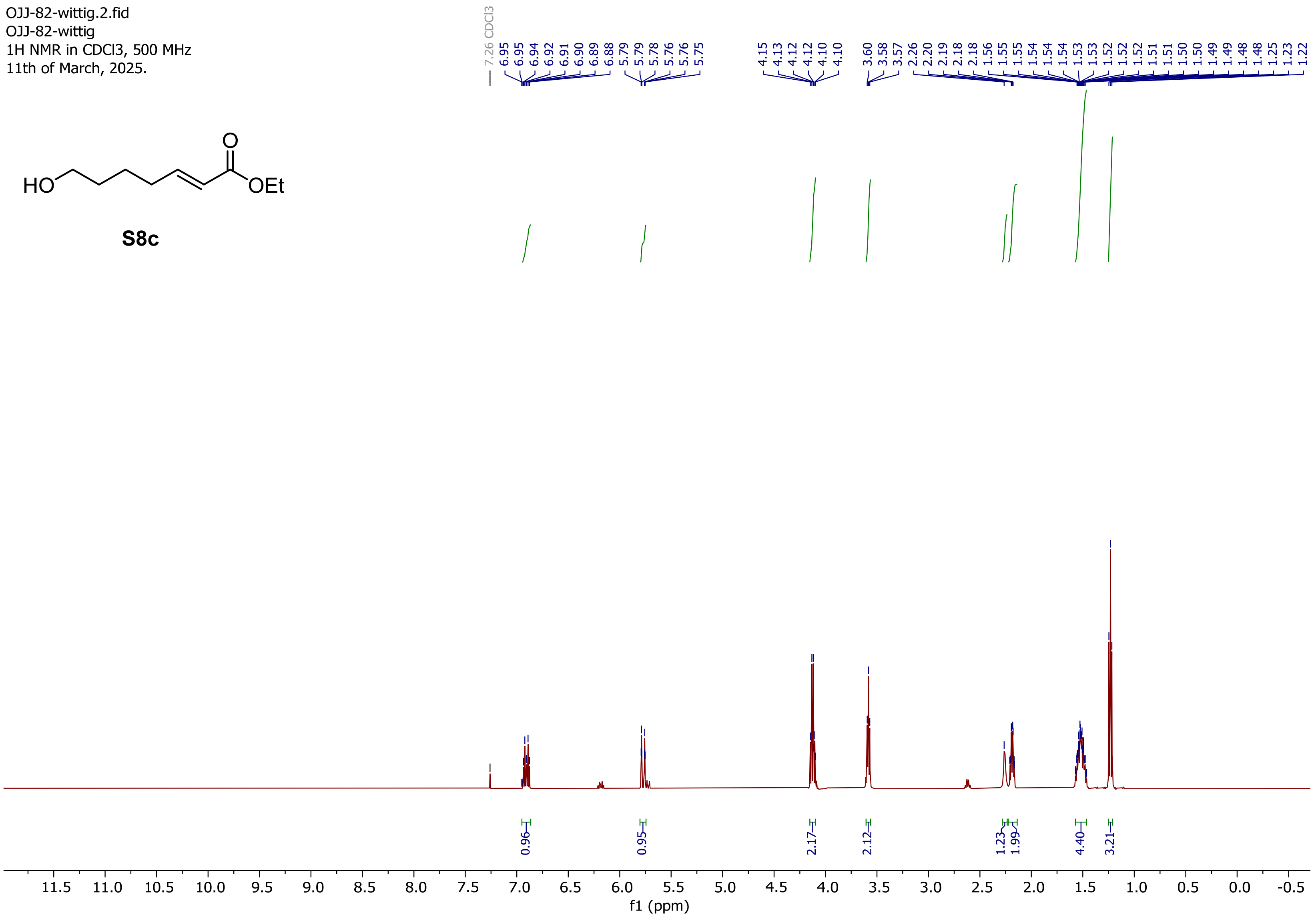

8.22 $^{13}C\{^{1}H\}$ NMR spectrum of S8c

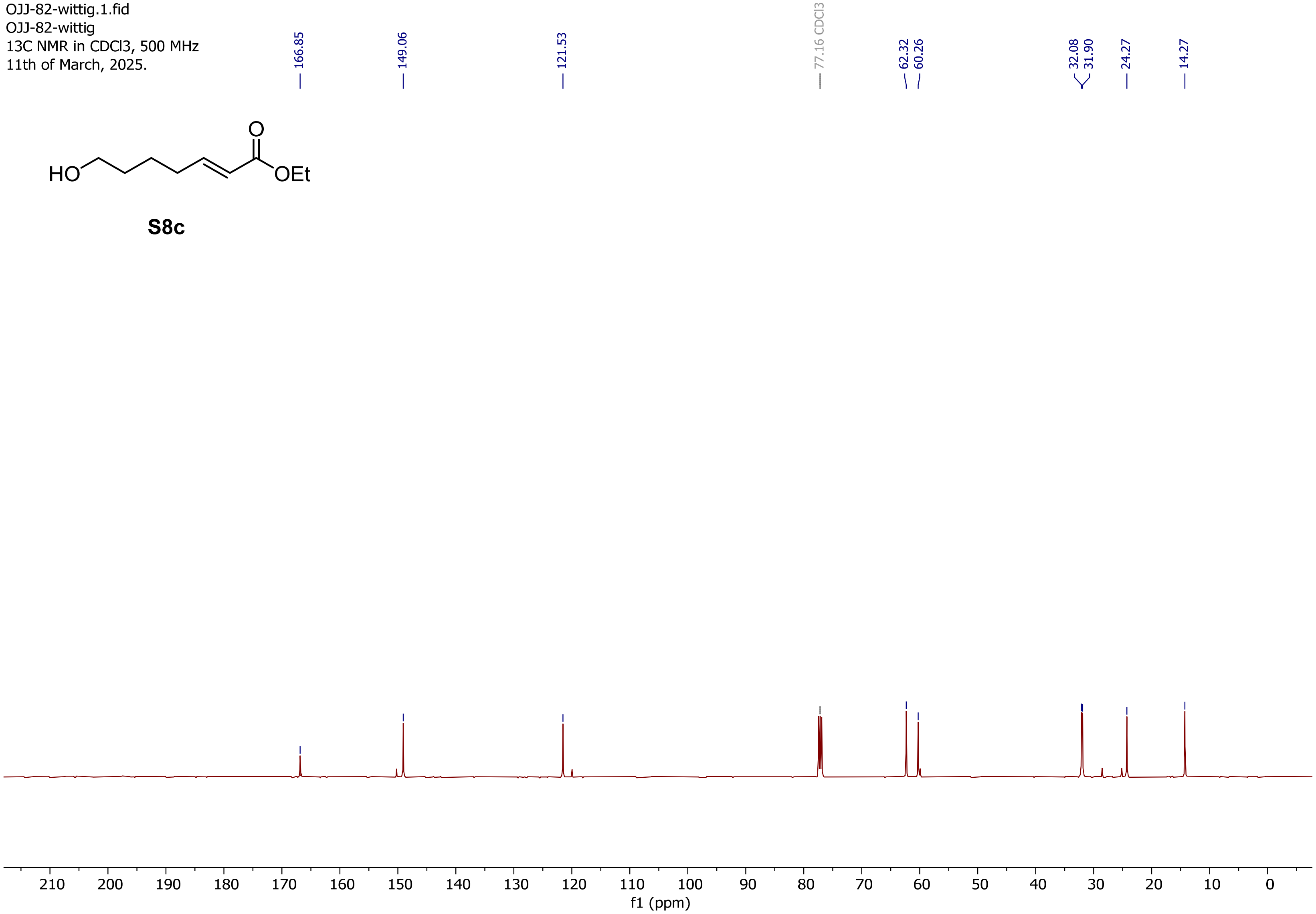

8.23 ^{1}H NMR spectrum of S8d

OJJ-109.1.fid
OJJ-109
1H in CDCl3, 300MHz
11th March, 2025

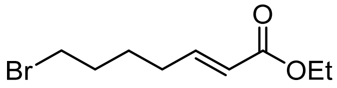

S8d

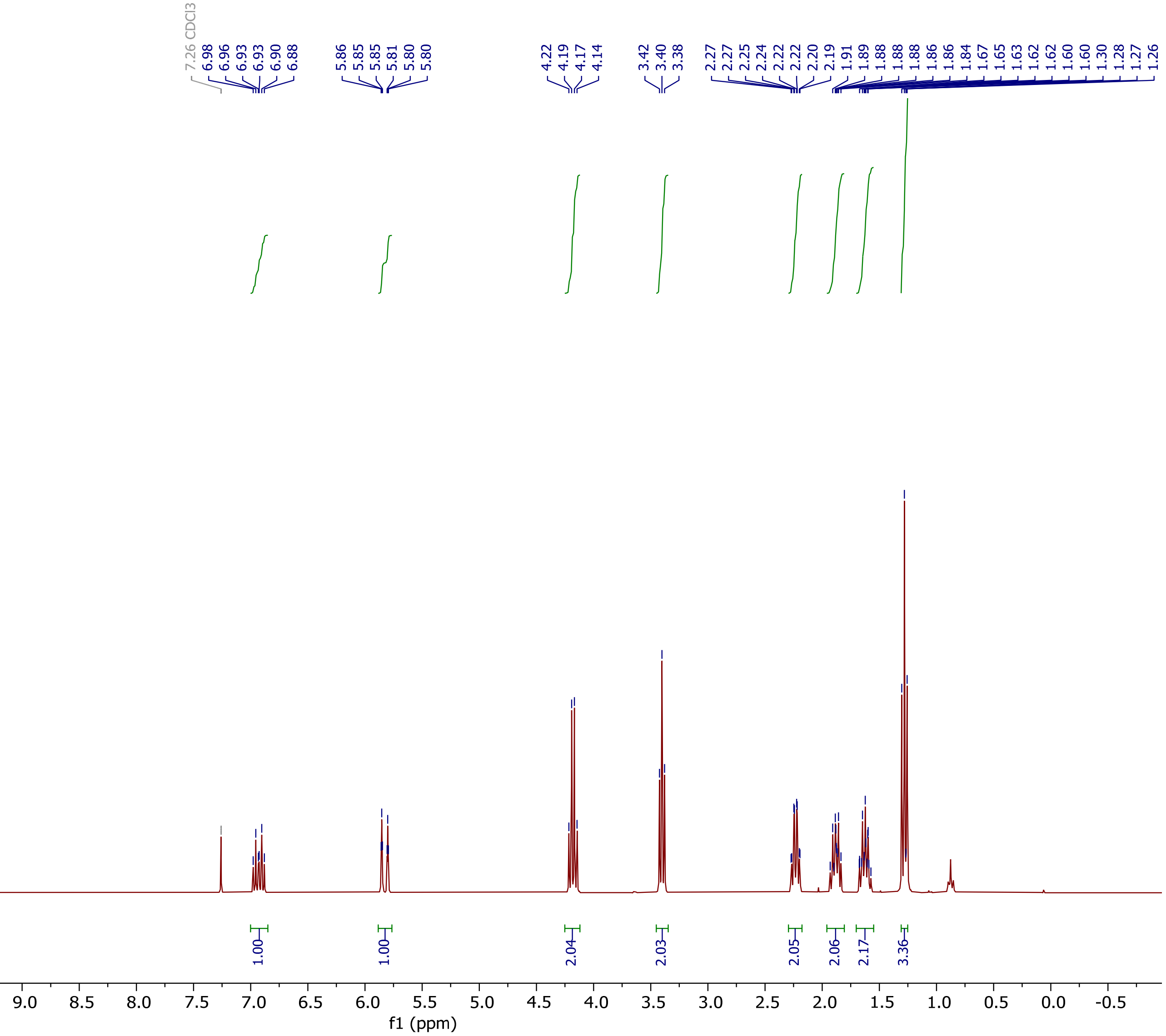

8.24 $^{13}C\{^1H\}$ NMR spectrum of S8d

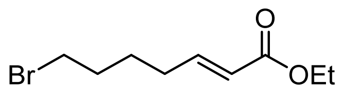

S8d

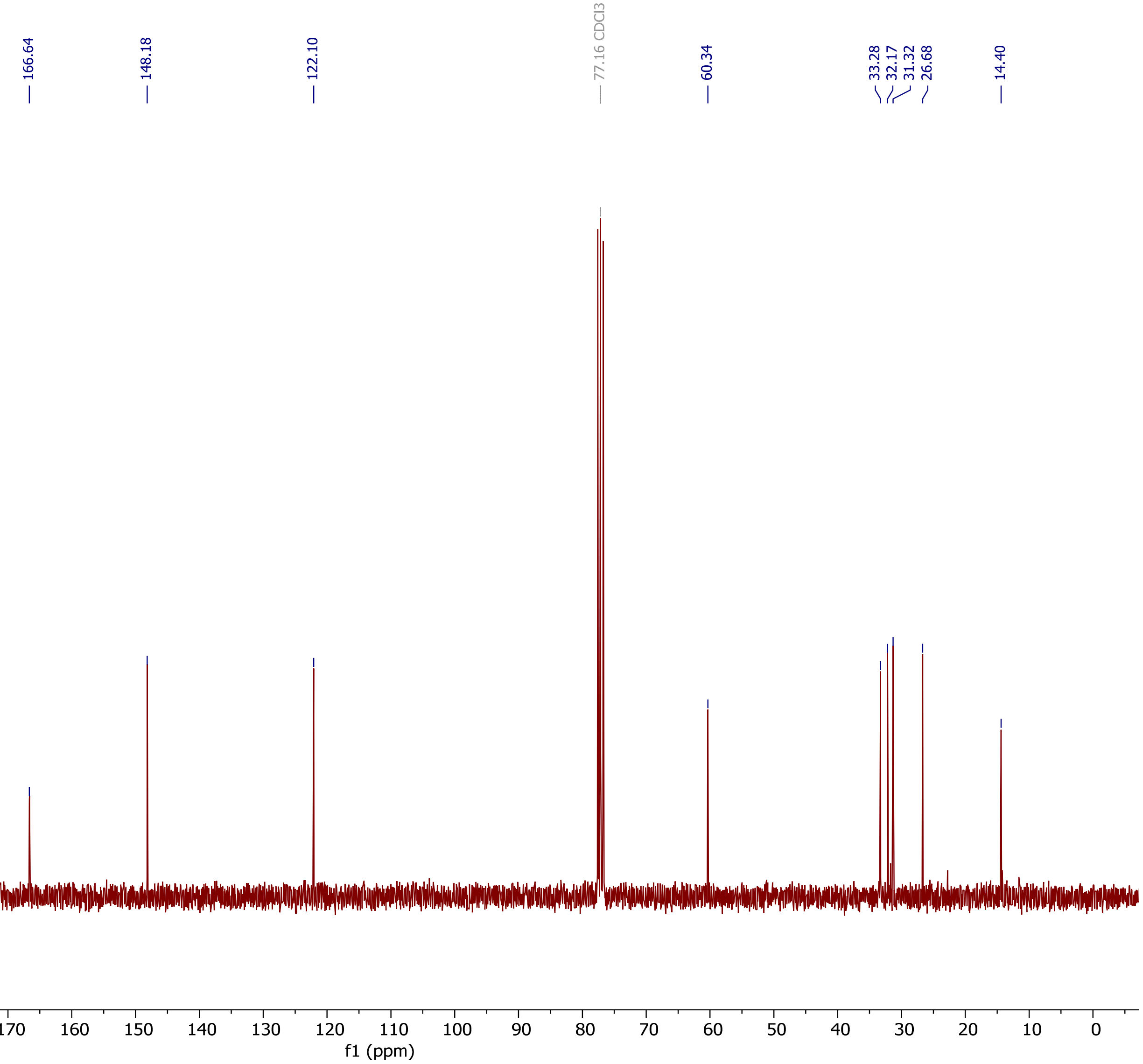

8.25 ^{1}H NMR spectrum of 4f

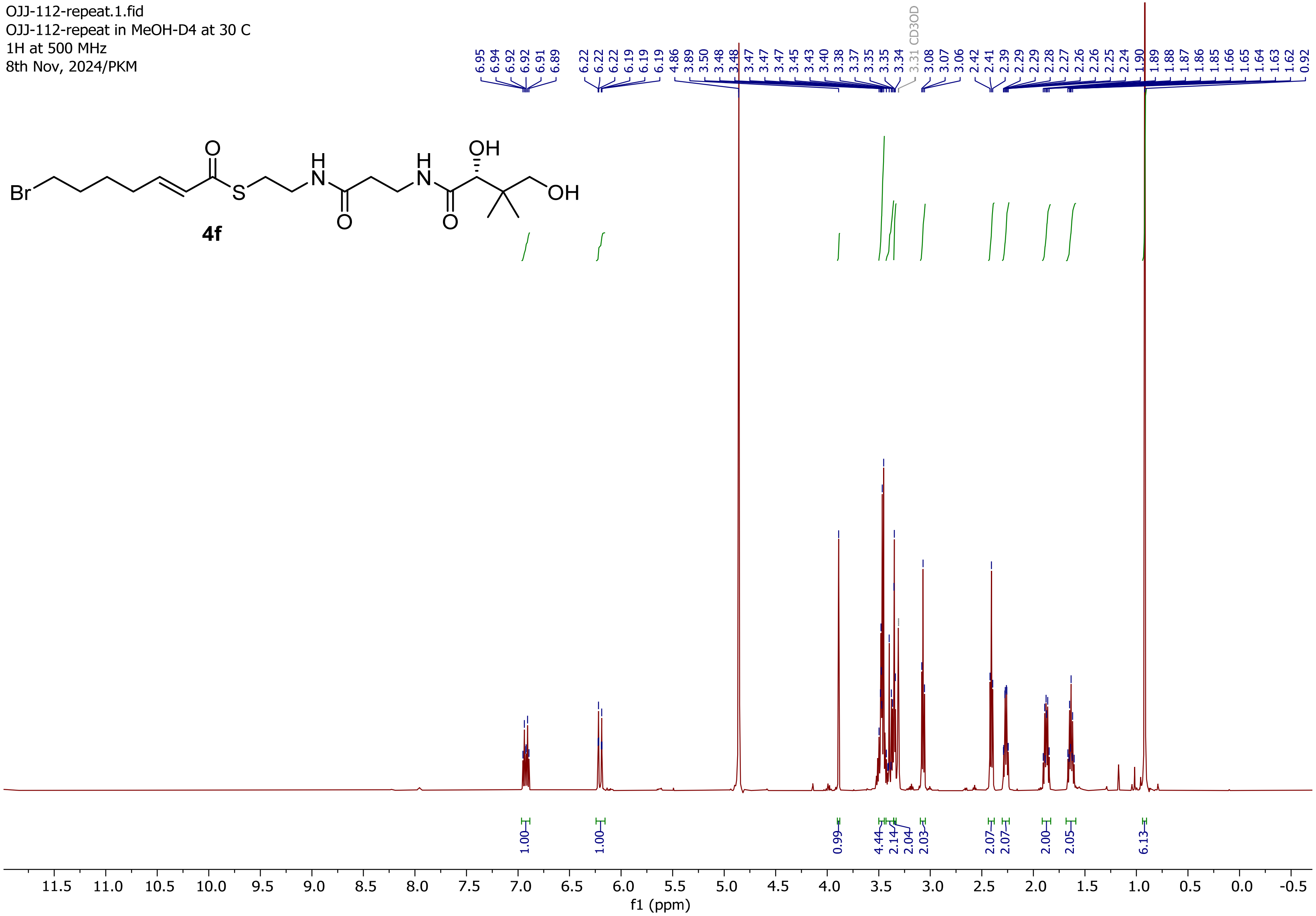

8.26 $^{13}C\{^{1}H\}$ NMR spectrum of 4f

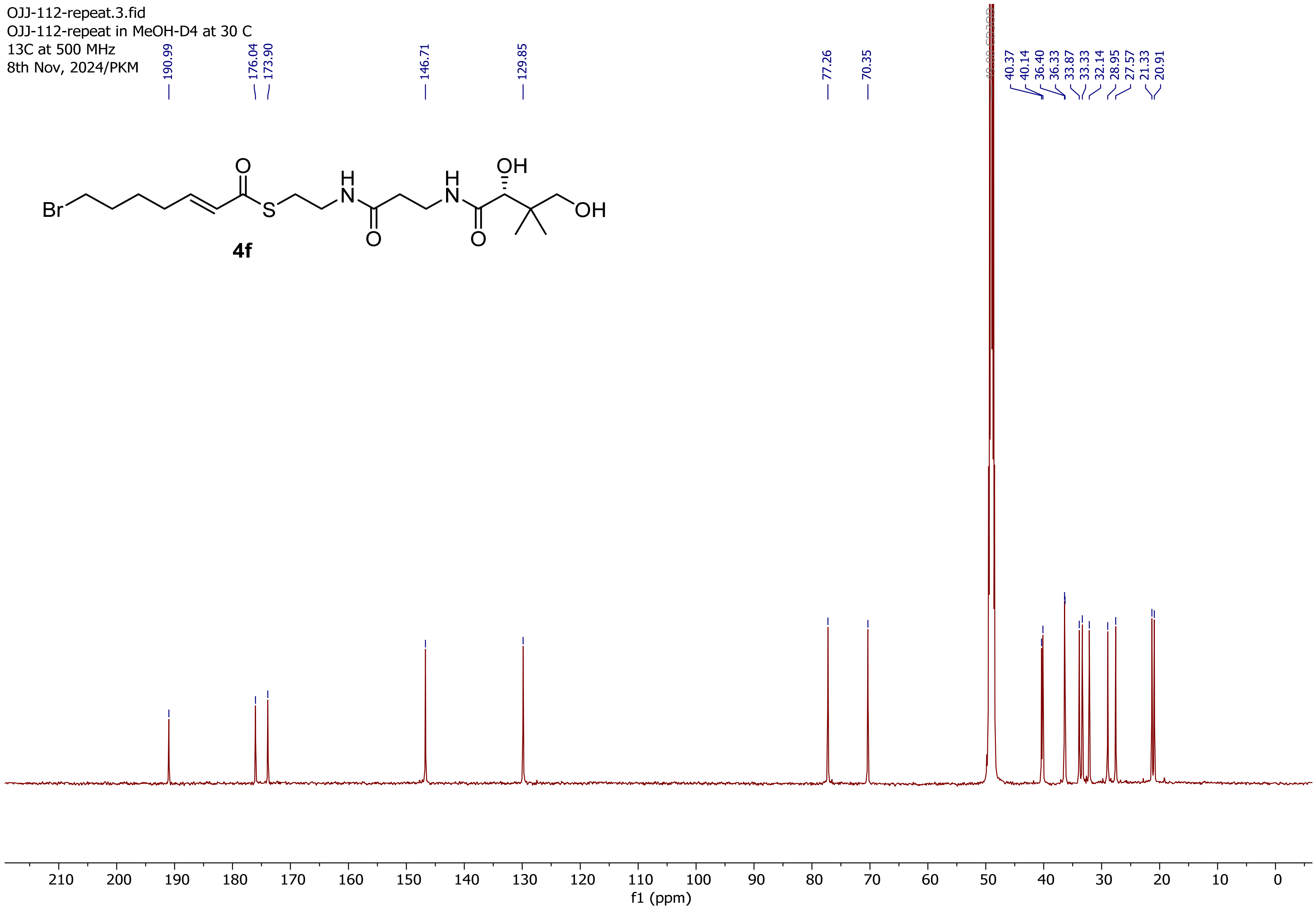

8.27 ¹H NMR spectrum of S9a

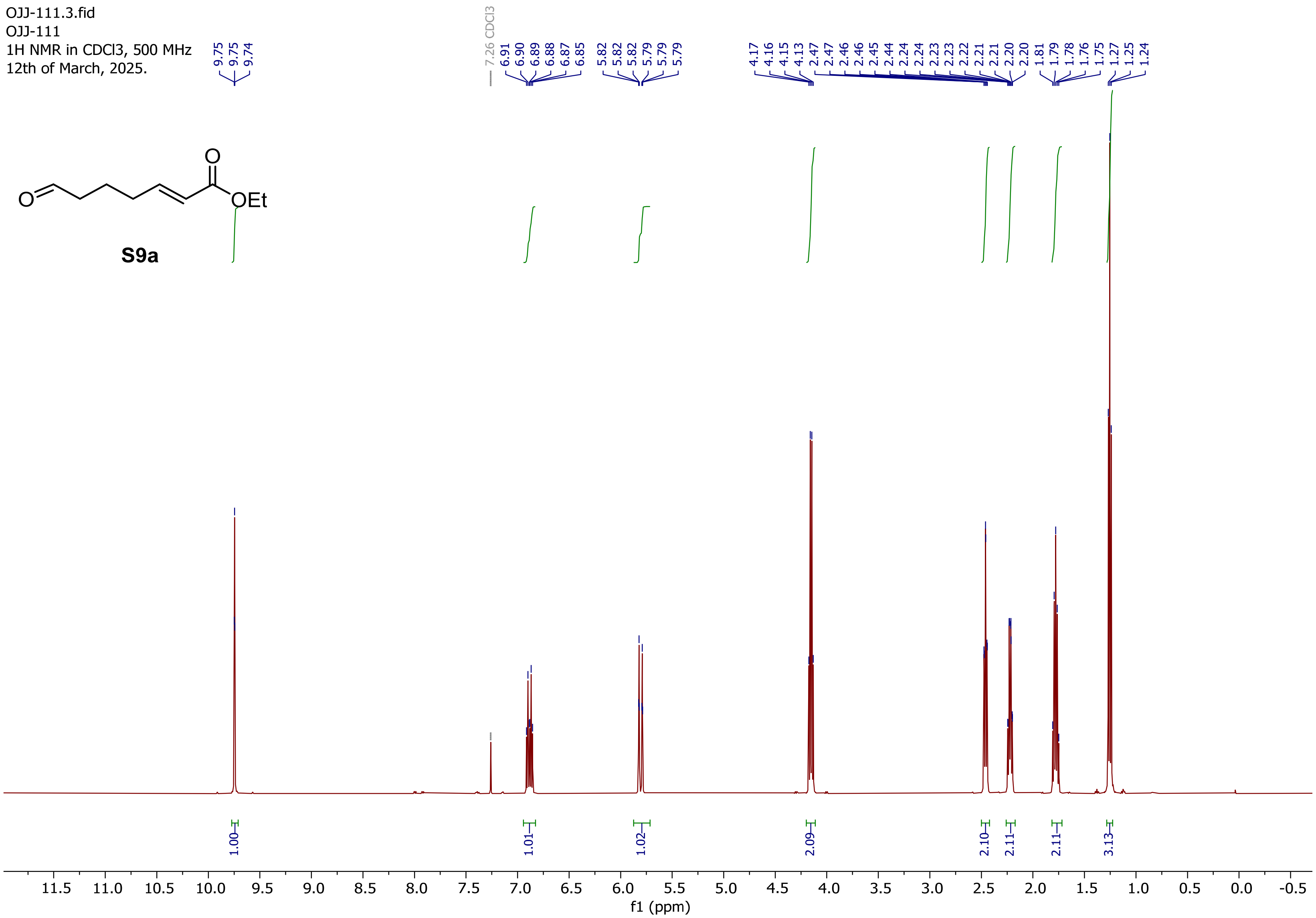

8.28 $^{13}C\{^{1}H\}$ NMR spectrum of S9a

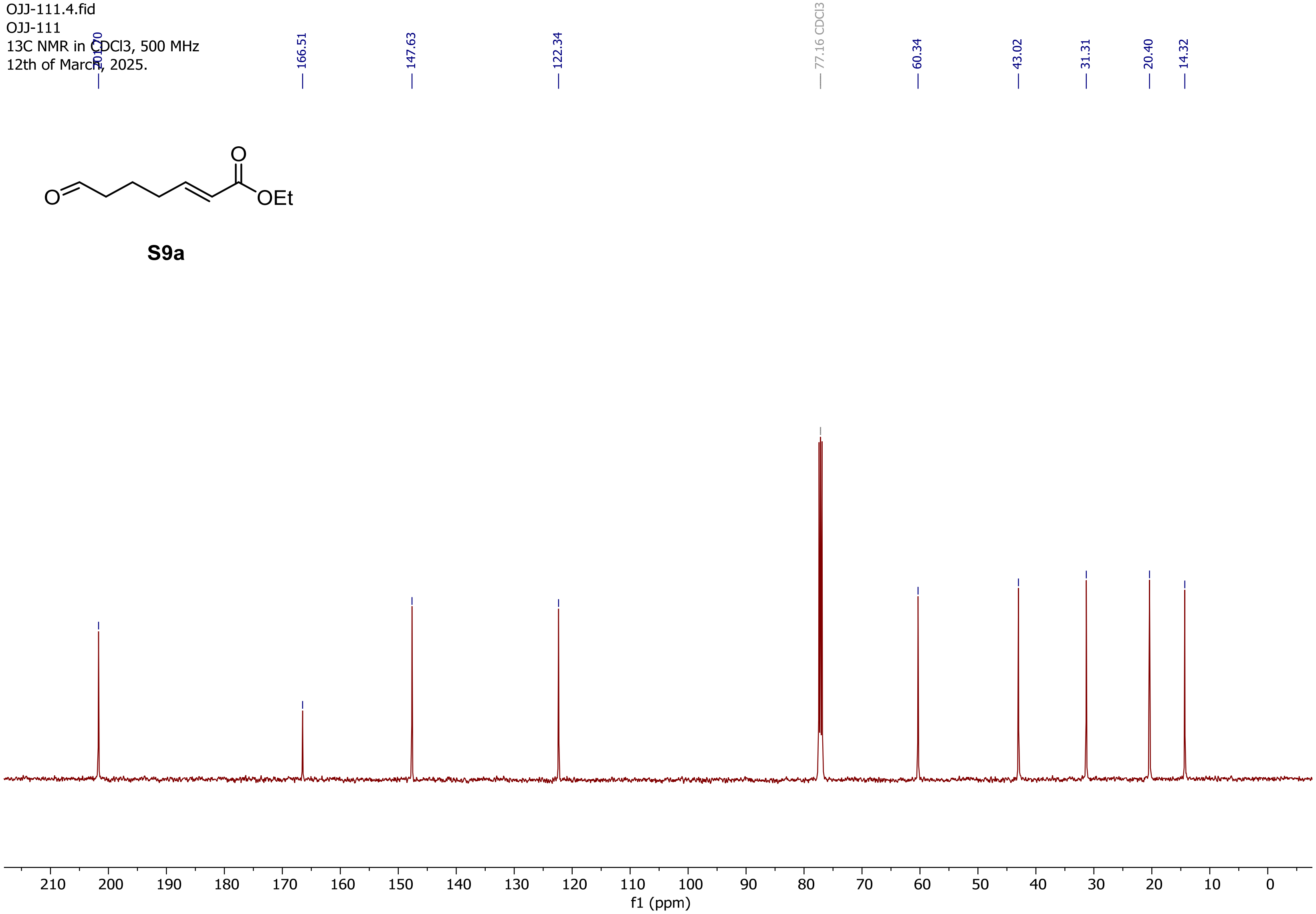

8.29 ^{1}H NMR spectrum of S9b

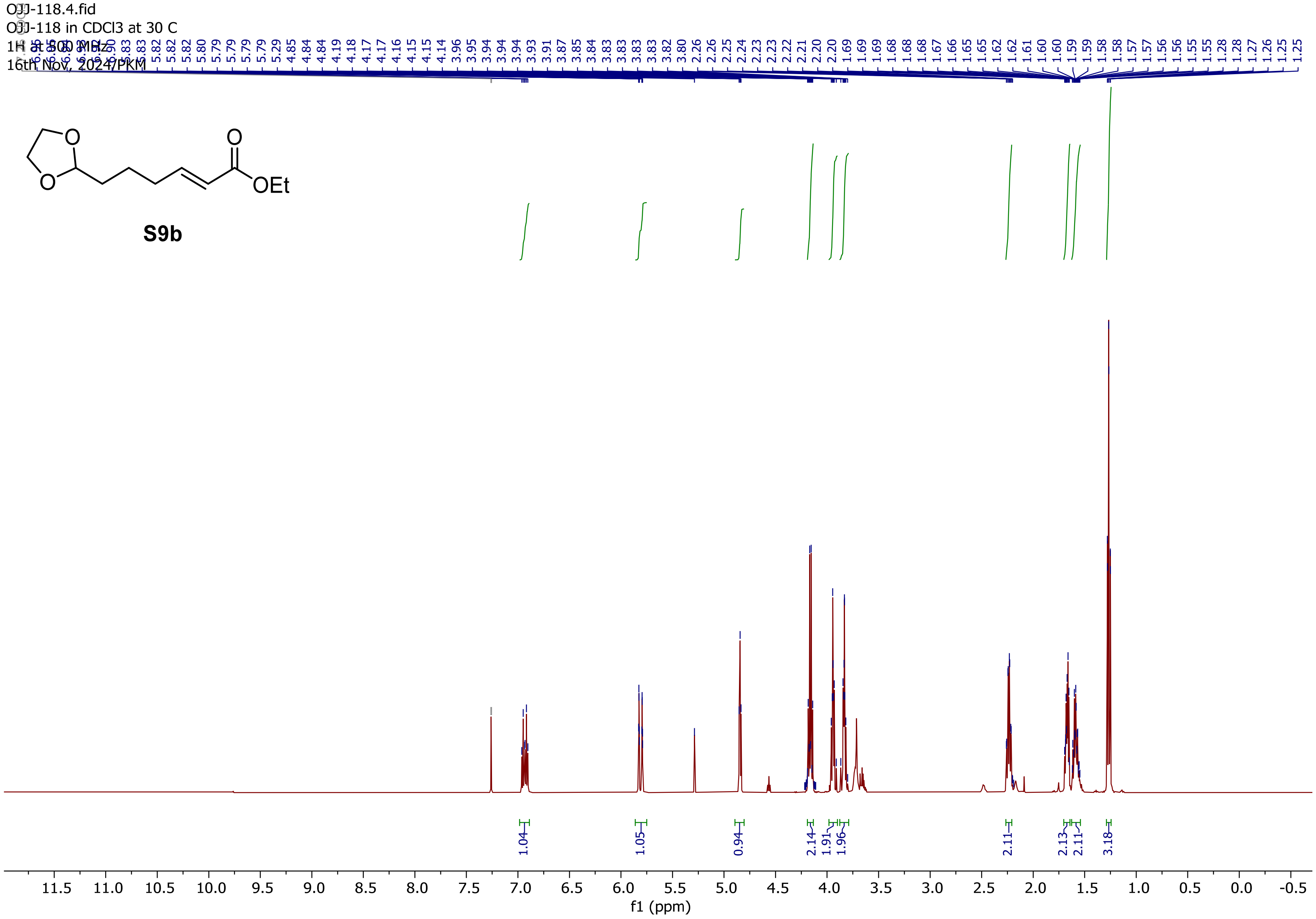

8.30 ^{1}H NMR spectrum of S9c

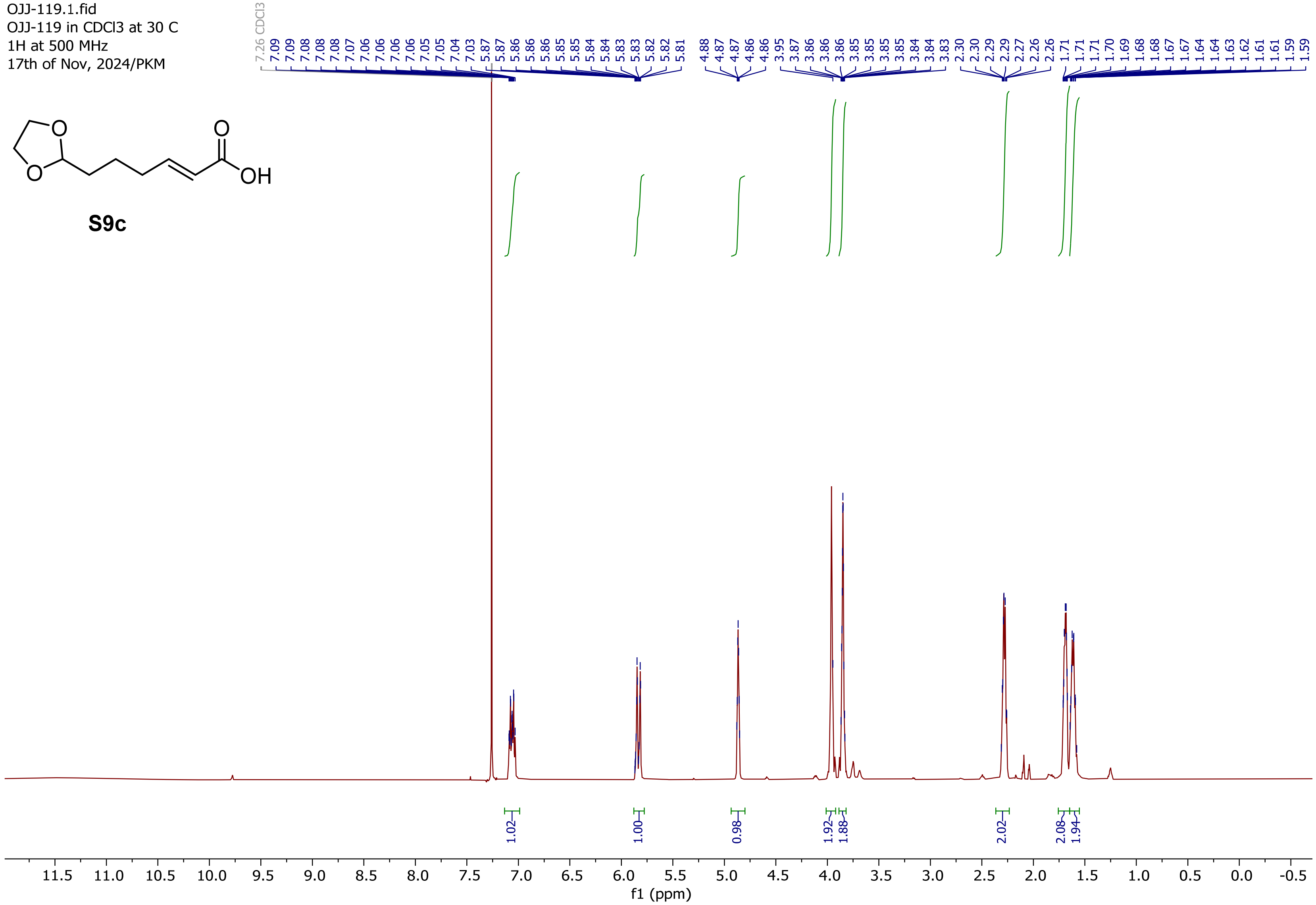

8.31 ^{1}H NMR spectrum of 4g

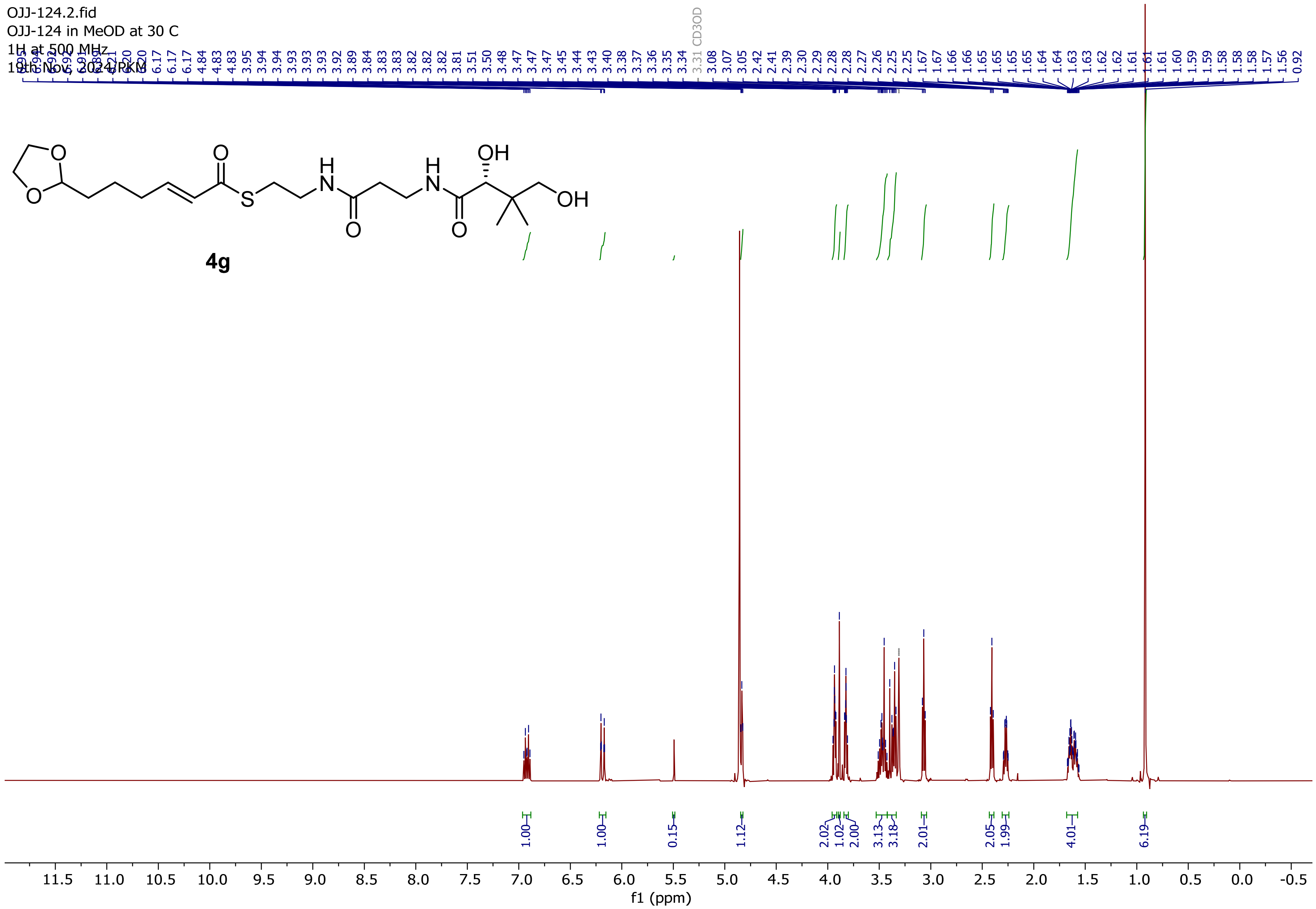

8.32 $^{13}C\{^1H\}$ NMR spectrum of 4g

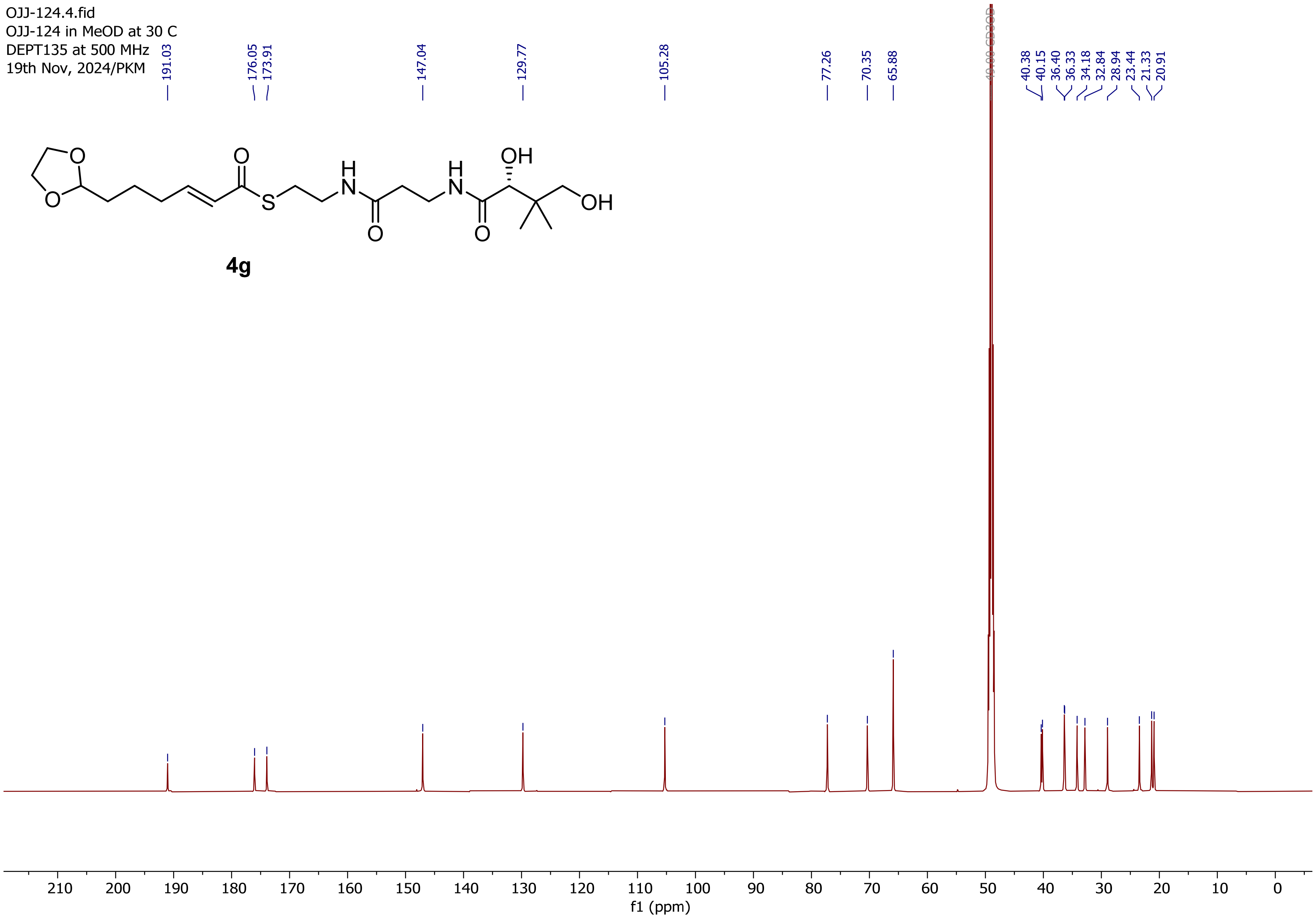

8.33 ^{1}H NMR spectrum of S10b

OJJ-127.7.fid
OJJ-127 in CDCl3 at 30 C
1H at 500 MHz
13th of Dec, 2024/PKM

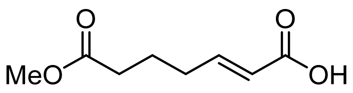

S10b

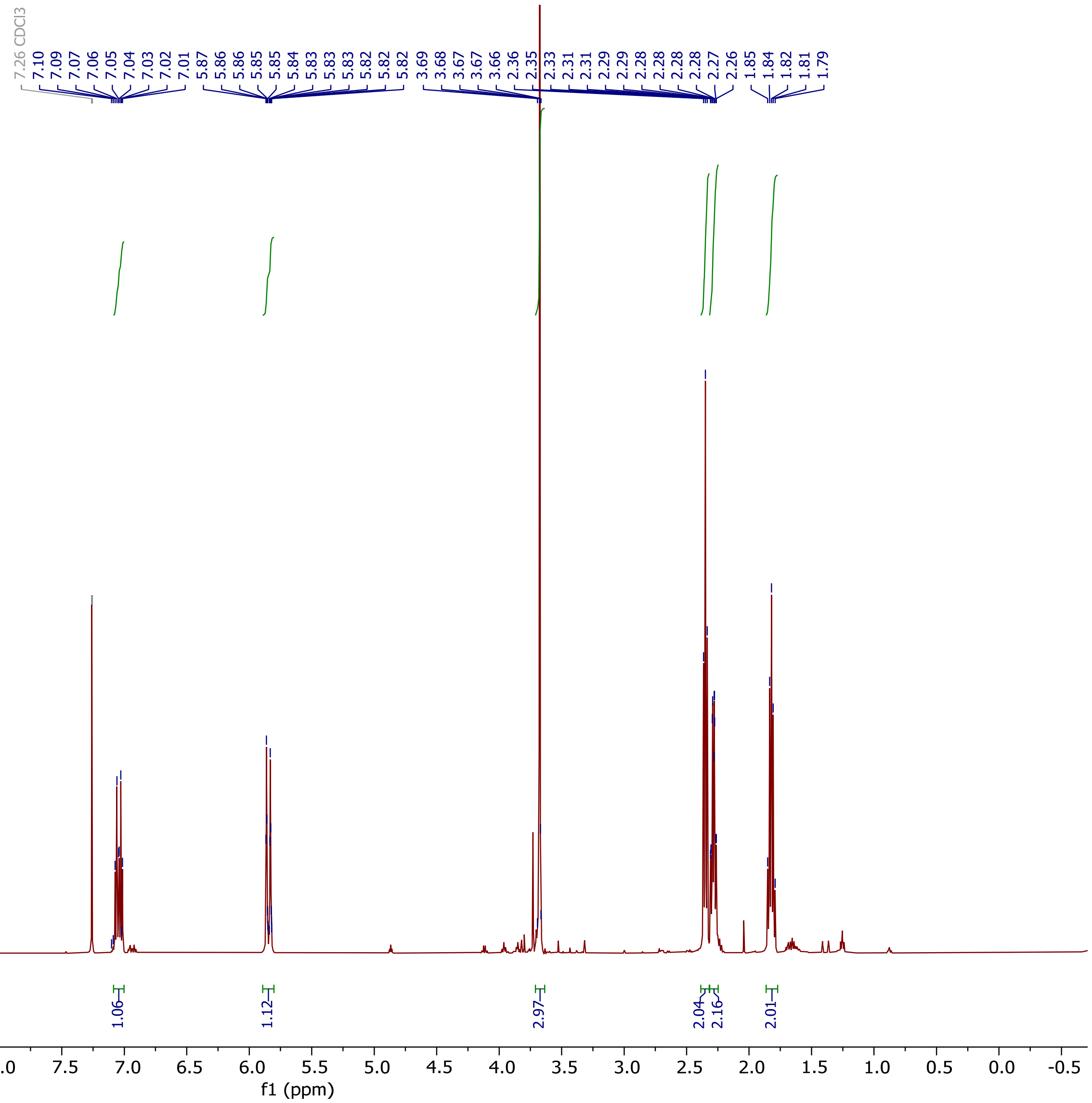

8.34 $^{13}C\{^{1}H\}$ NMR spectrum of S10b

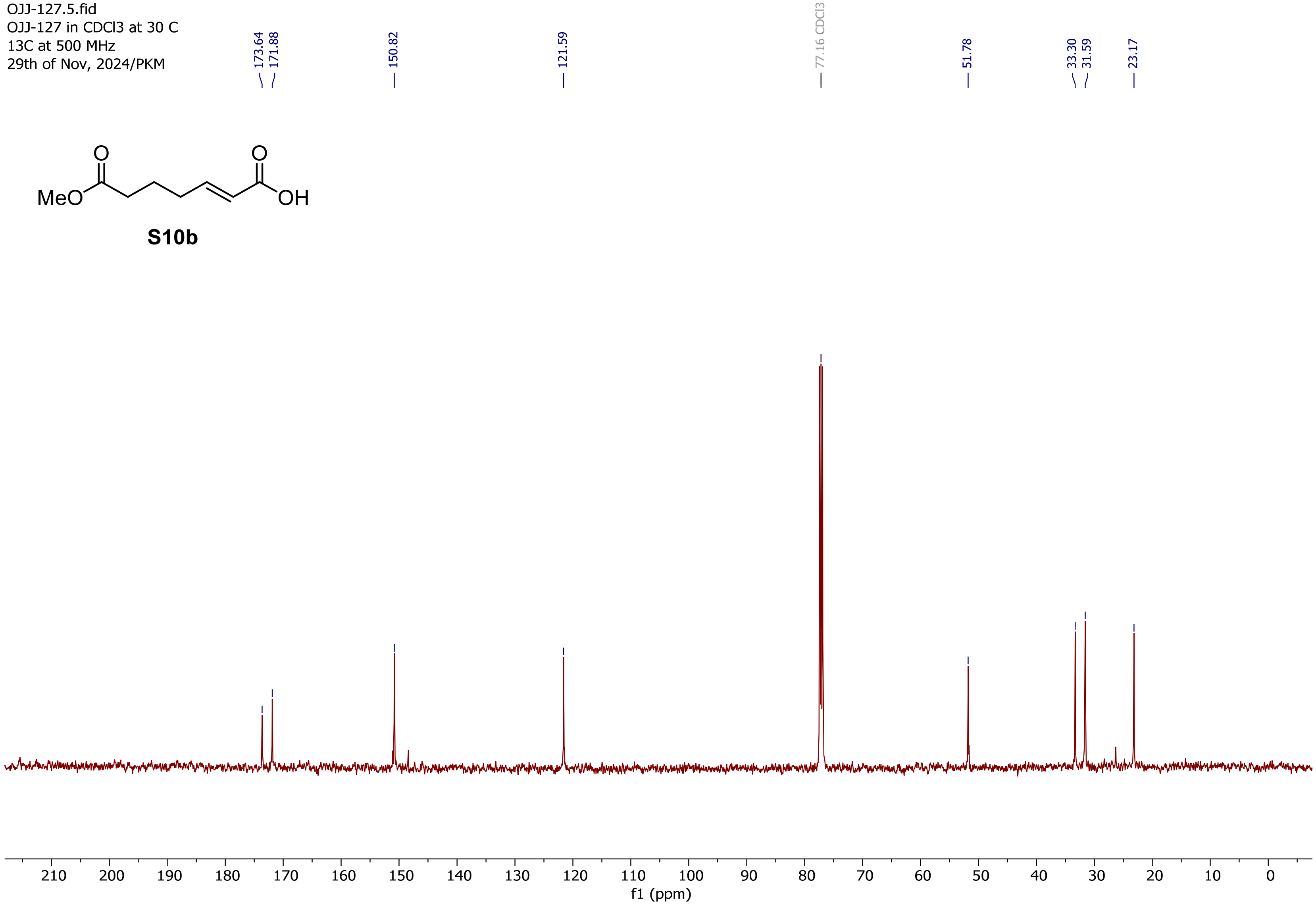

8.35 ^{1}H NMR spectrum of 4h

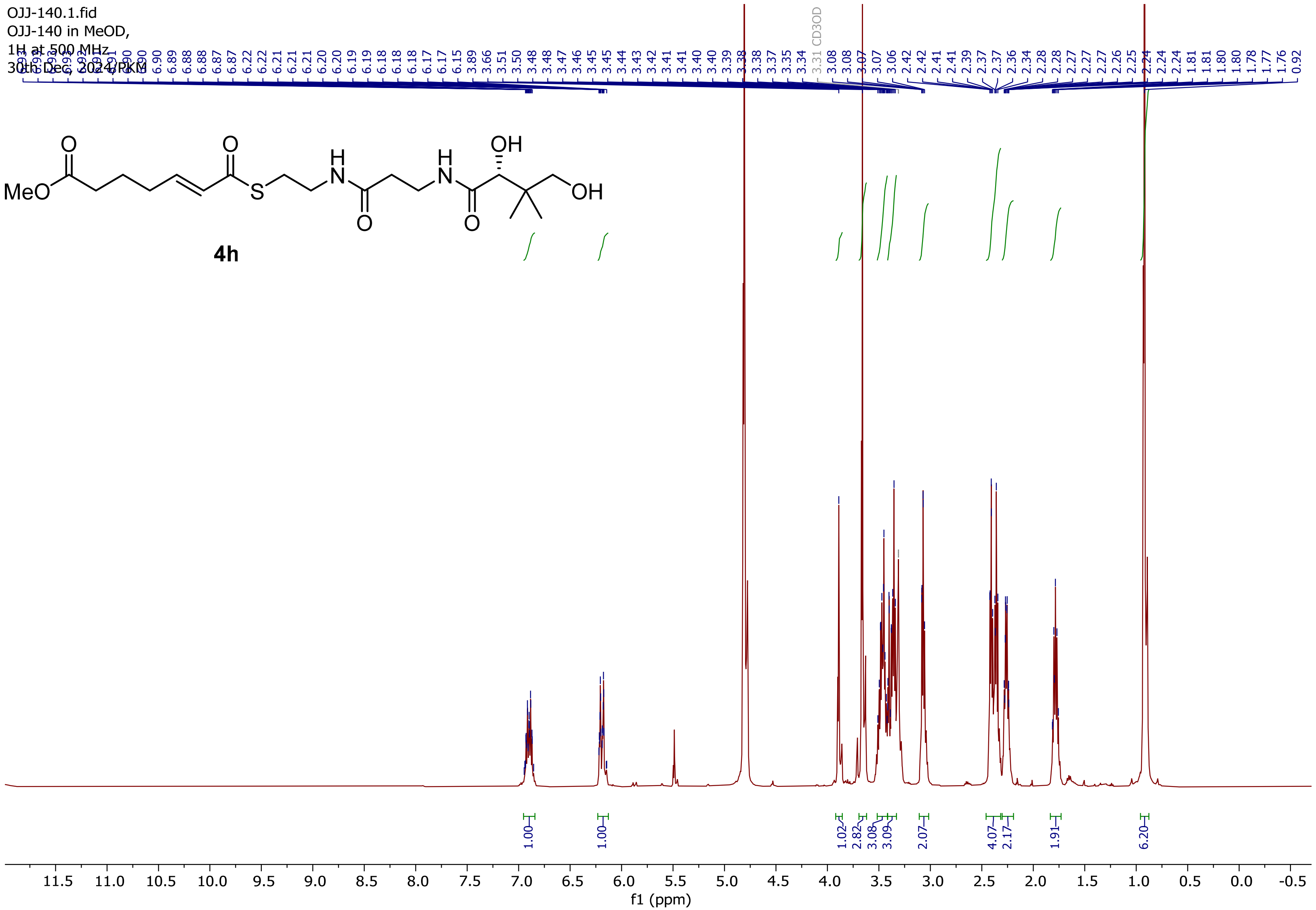

8.36 $^{13}C\{^1H\}$ NMR spectrum of 4h

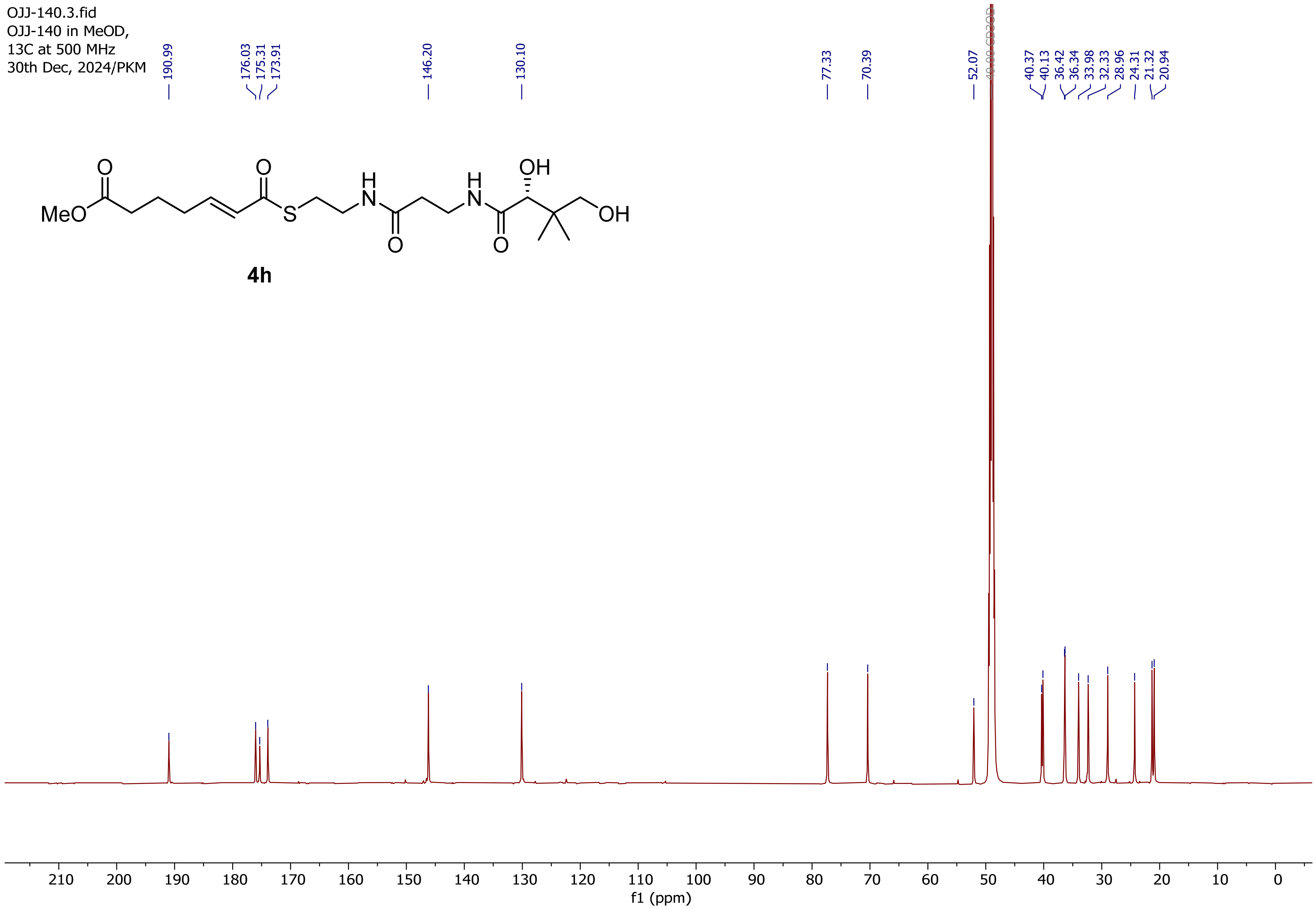

8.37 ¹H NMR spectrum of S11b

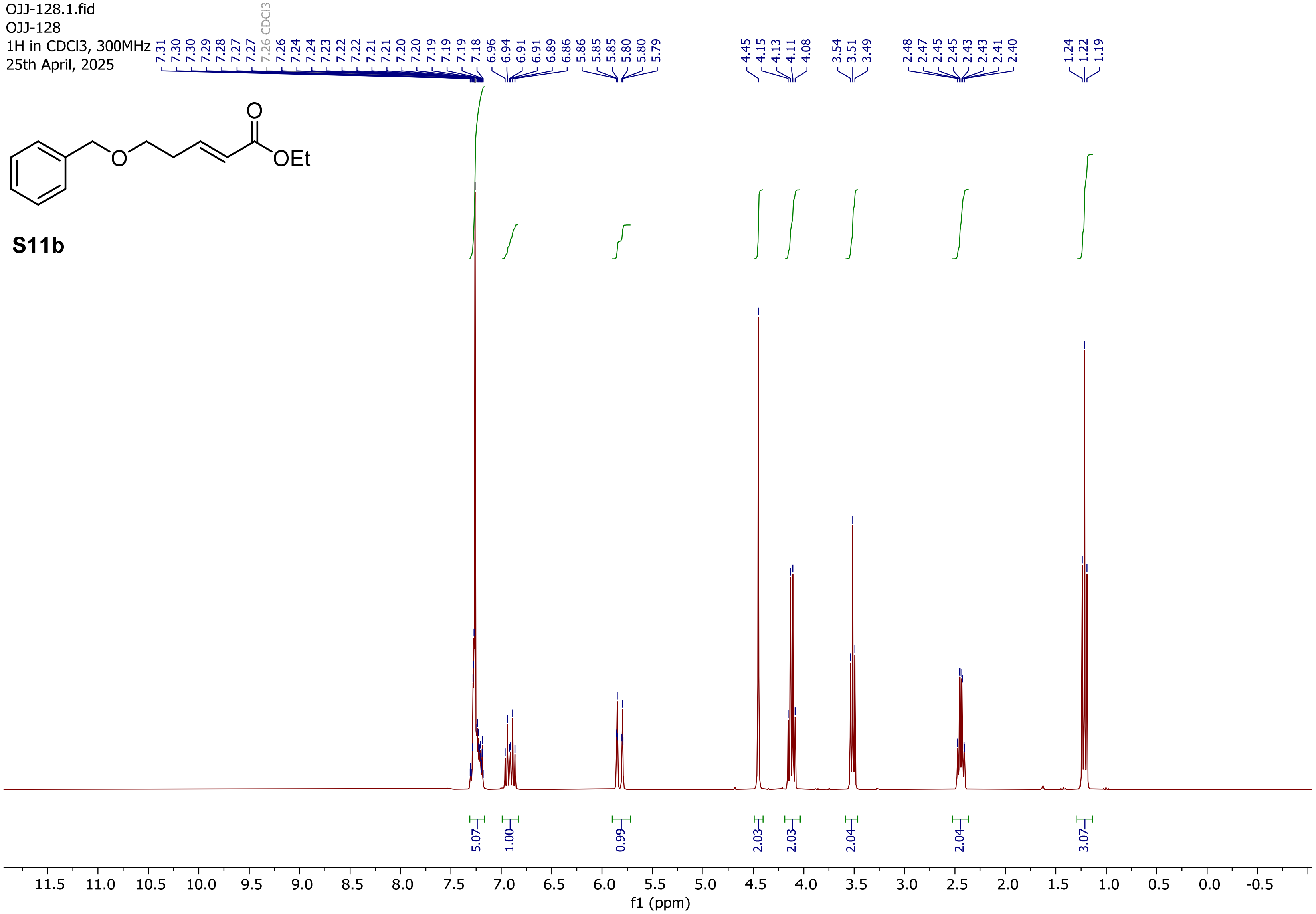

8.38 $^{13}C\{^1H\}$ NMR spectrum of S11b

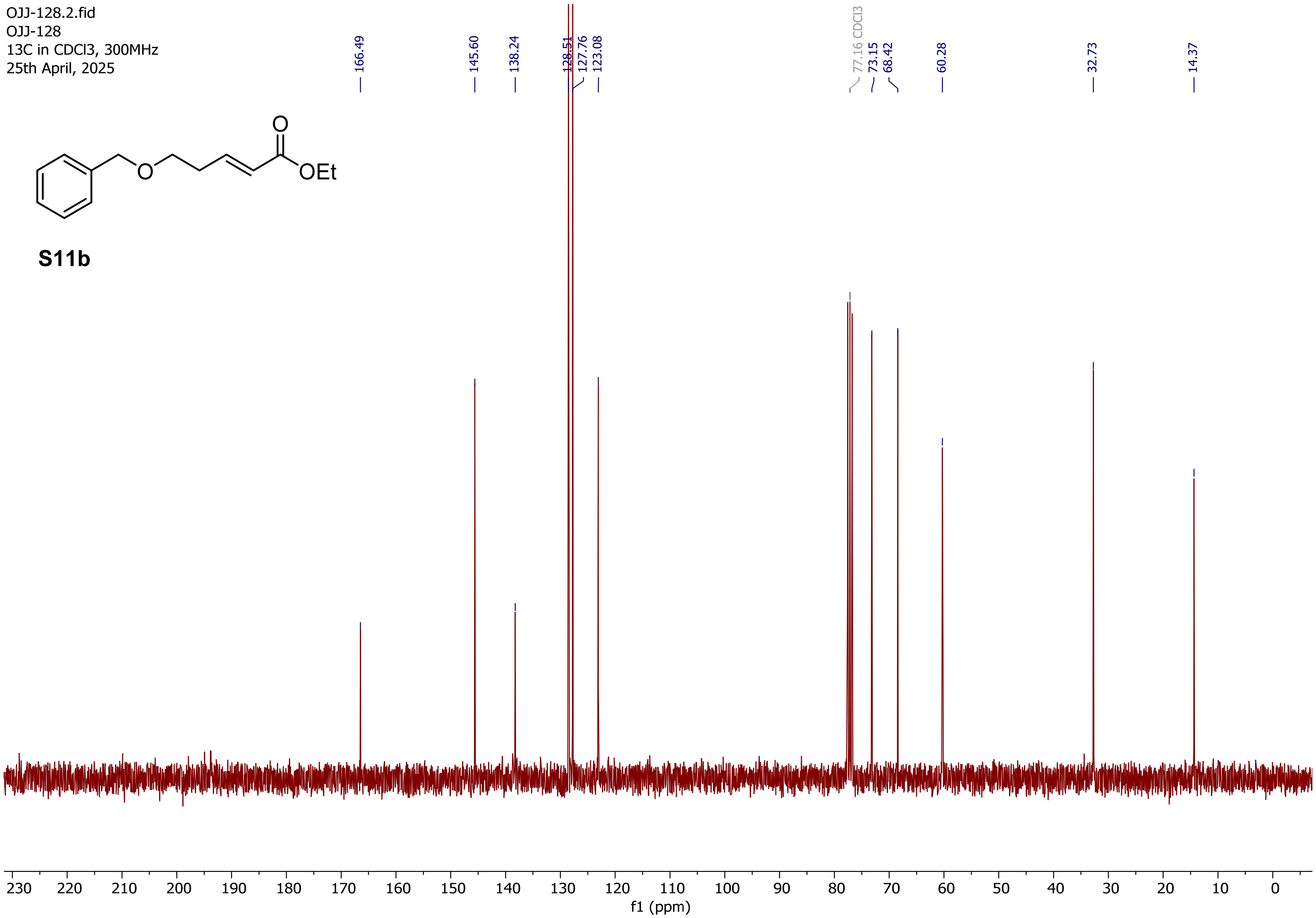

S11b

8.39 ^{1}H NMR spectrum of 4i

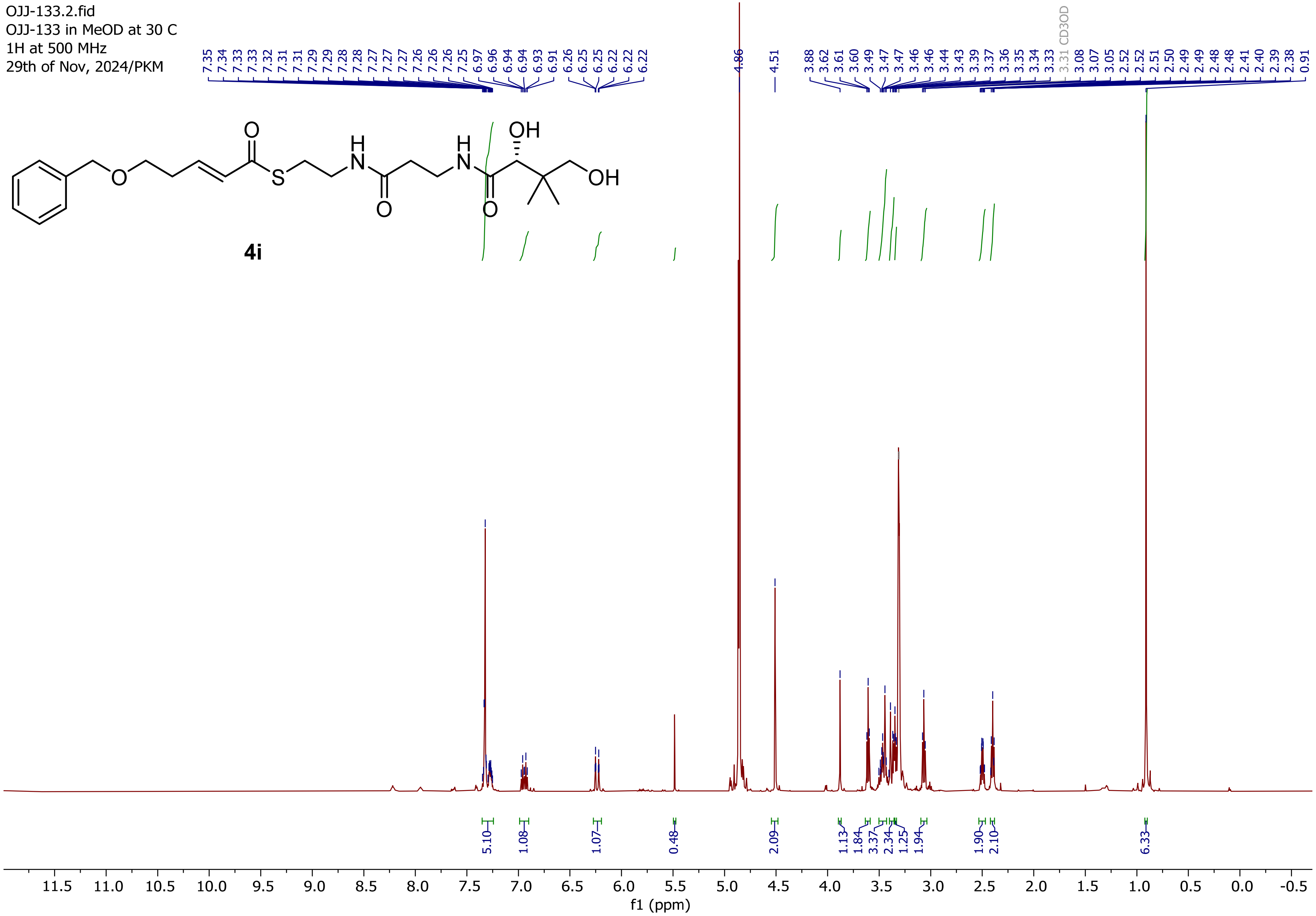

8.40 $^{13}C\{^1H\}$ NMR spectrum of 4i

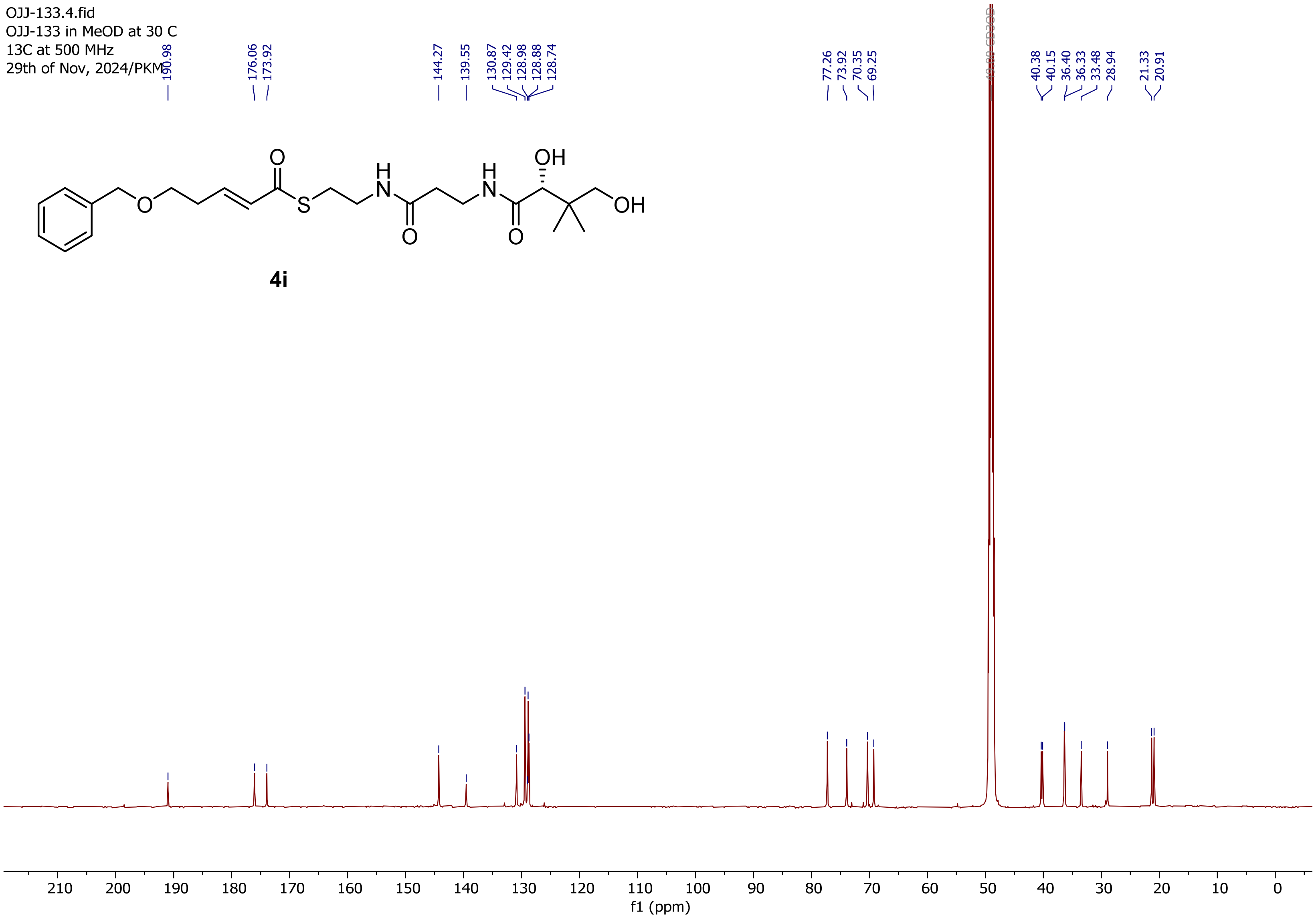

8.41 ^{1}H NMR spectrum of S12b

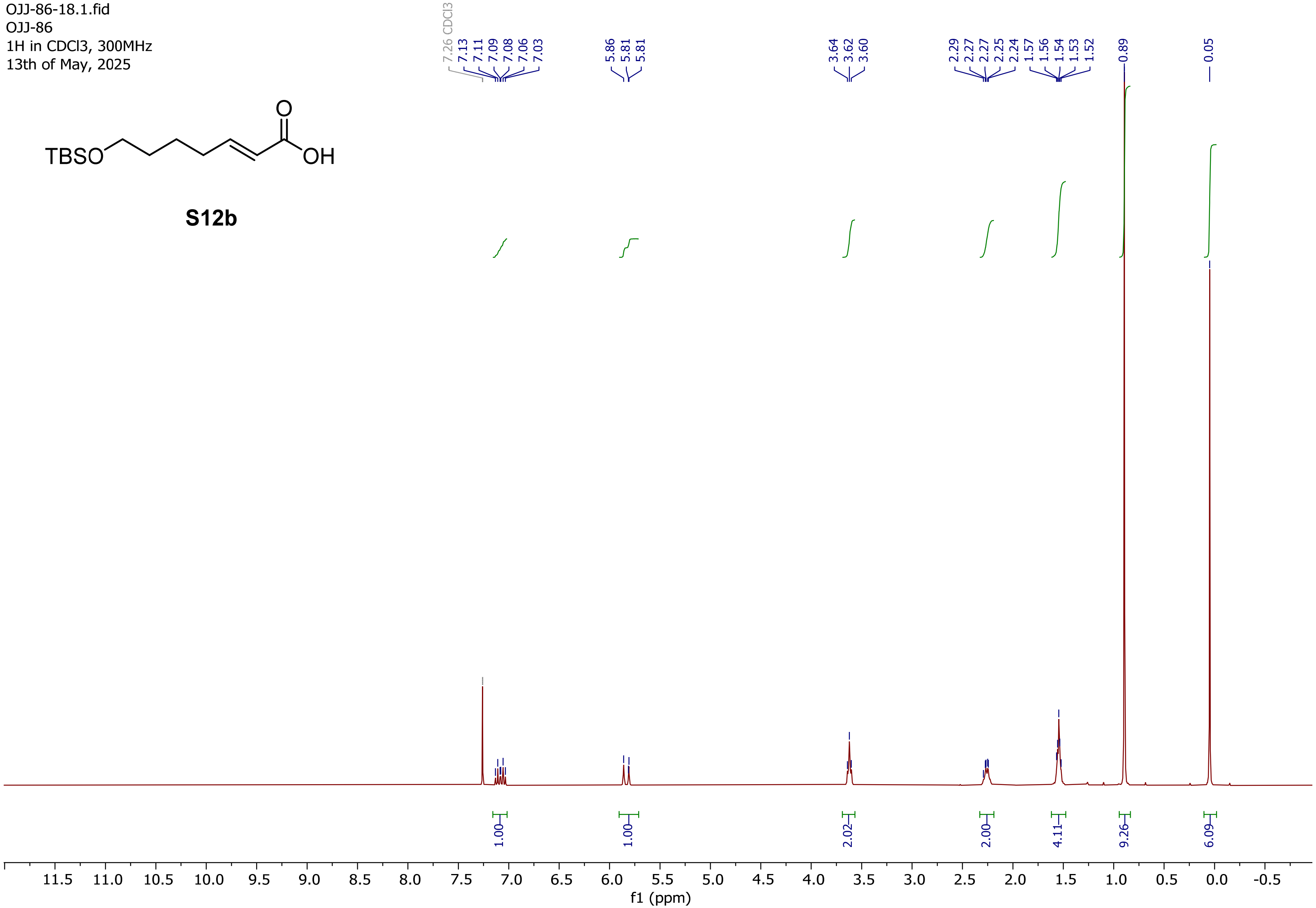

8.42 $^{13}C\{^{1}H\}$ NMR spectrum of S12b

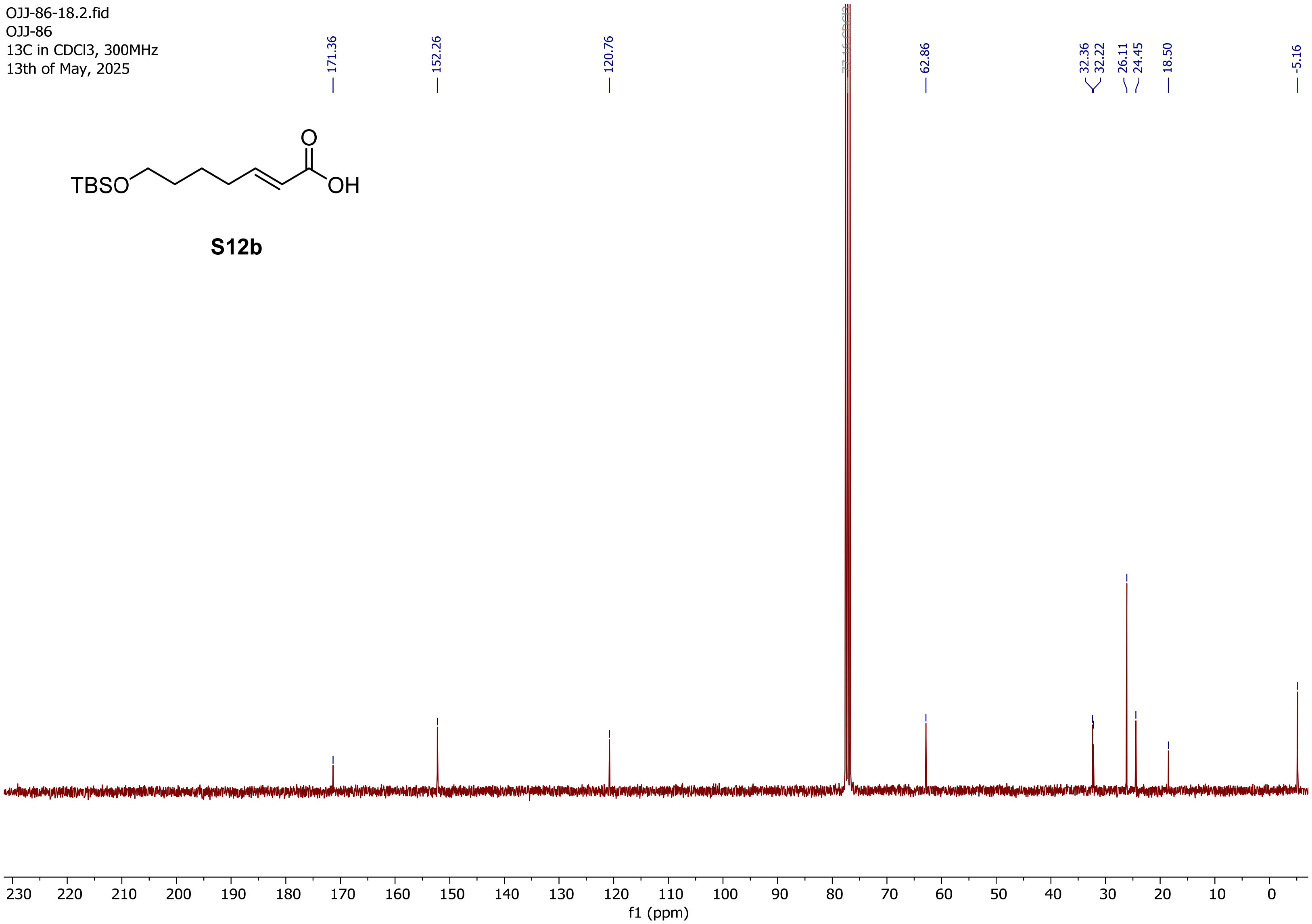

8.43 ^{1}H NMR spectrum of 4j

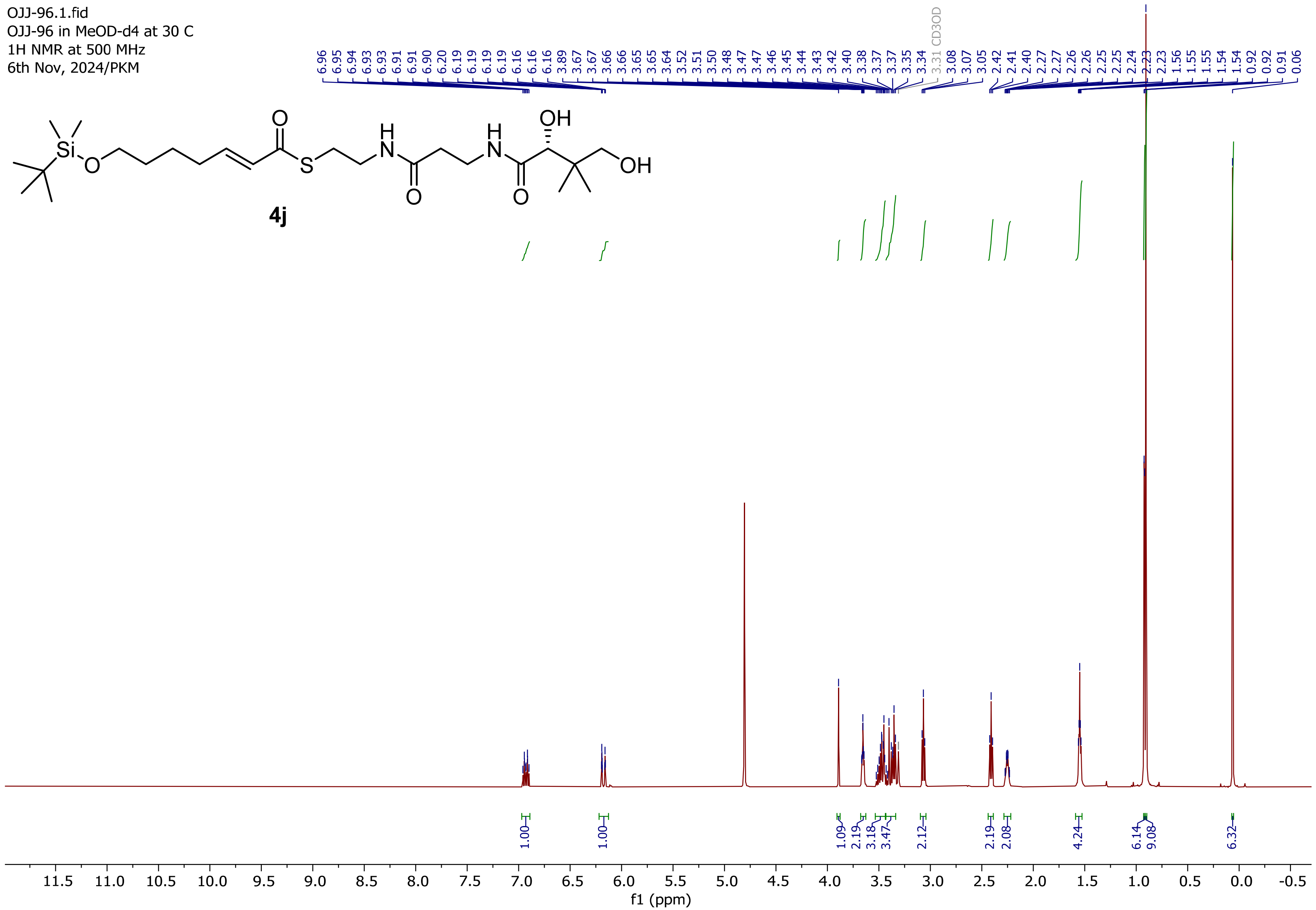

8.44 $^{13}C\{^{1}H\}$ NMR spectrum of 4j

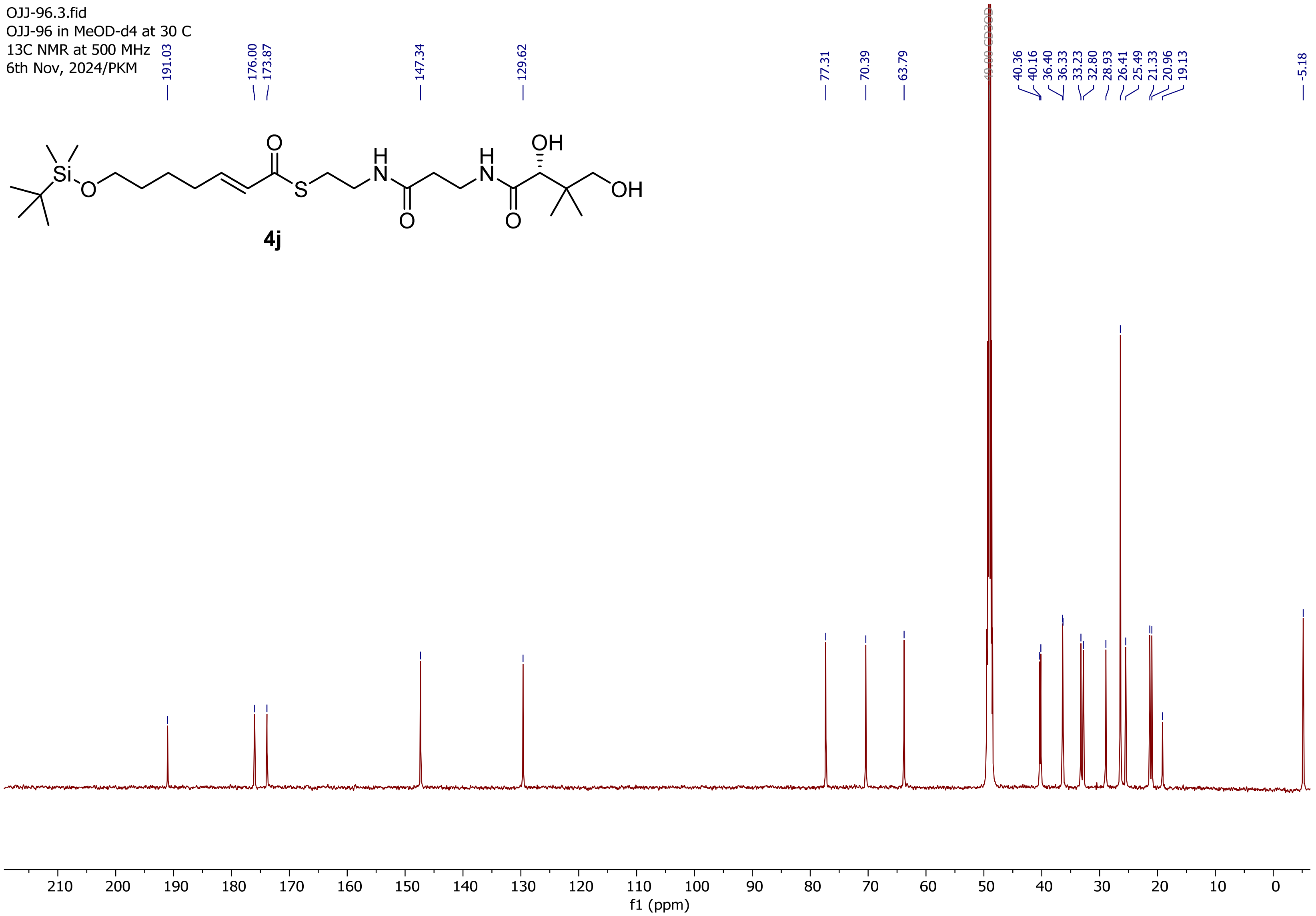

8.45 ^{1}H NMR spectrum of S13b

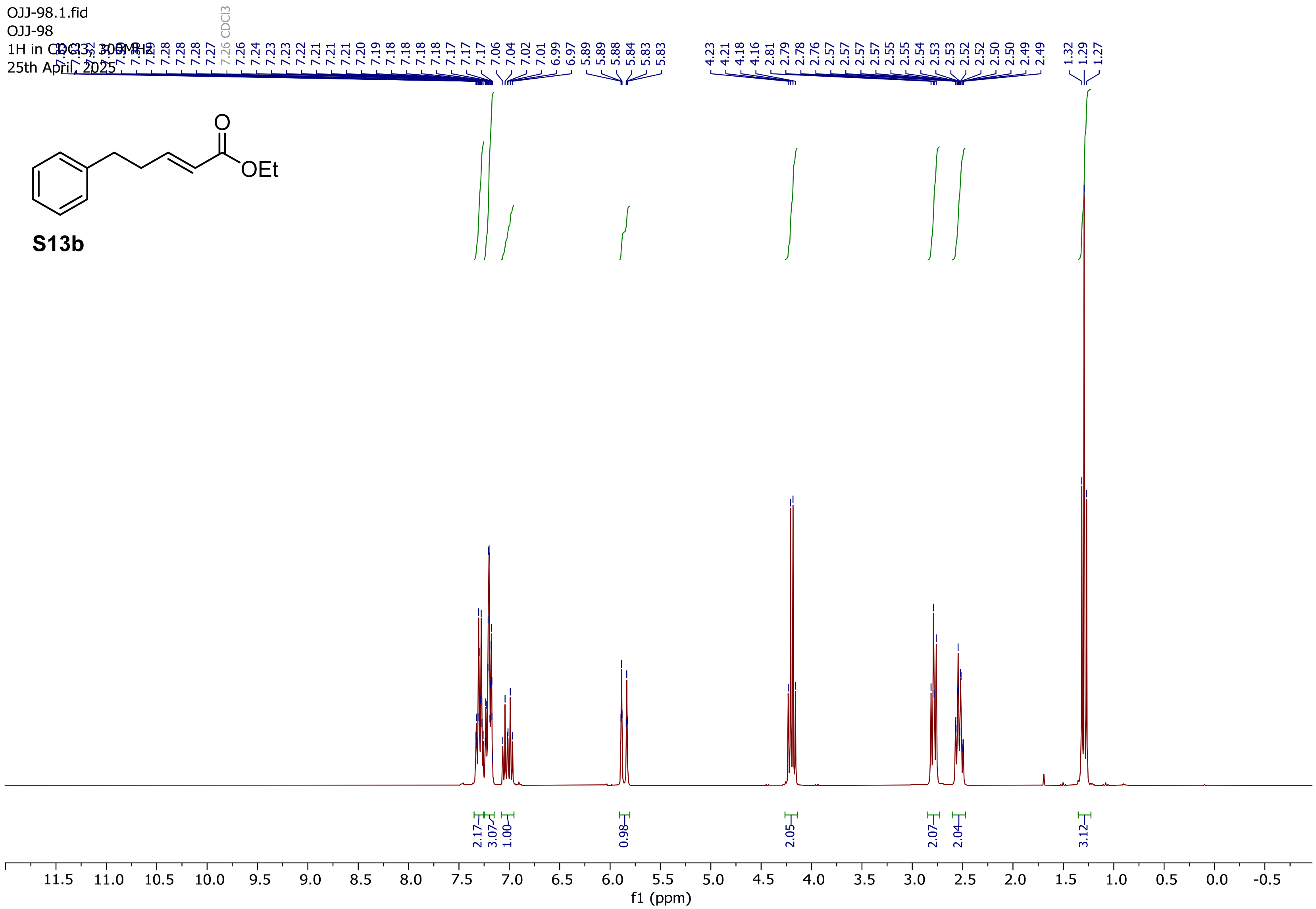

8.46 $^{13}C\{^{1}H\}$ NMR spectrum of S13b

OJJ-98.2.fid
OJJ-98
13C in CDCl3, 300MHz
25th April, 2025

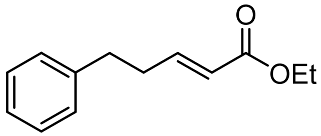

S13b

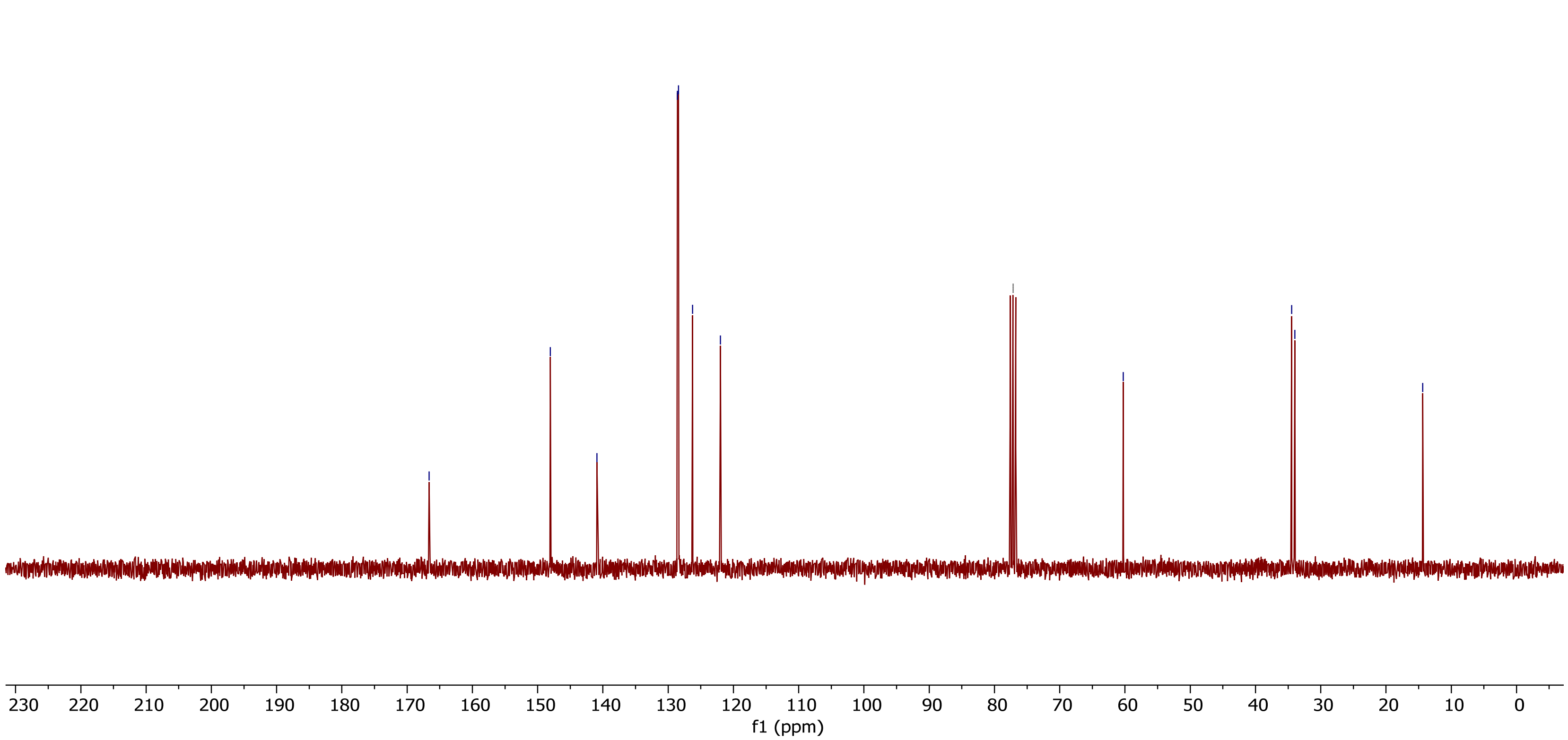

8.47 ^{1}H NMR spectrum of 4k

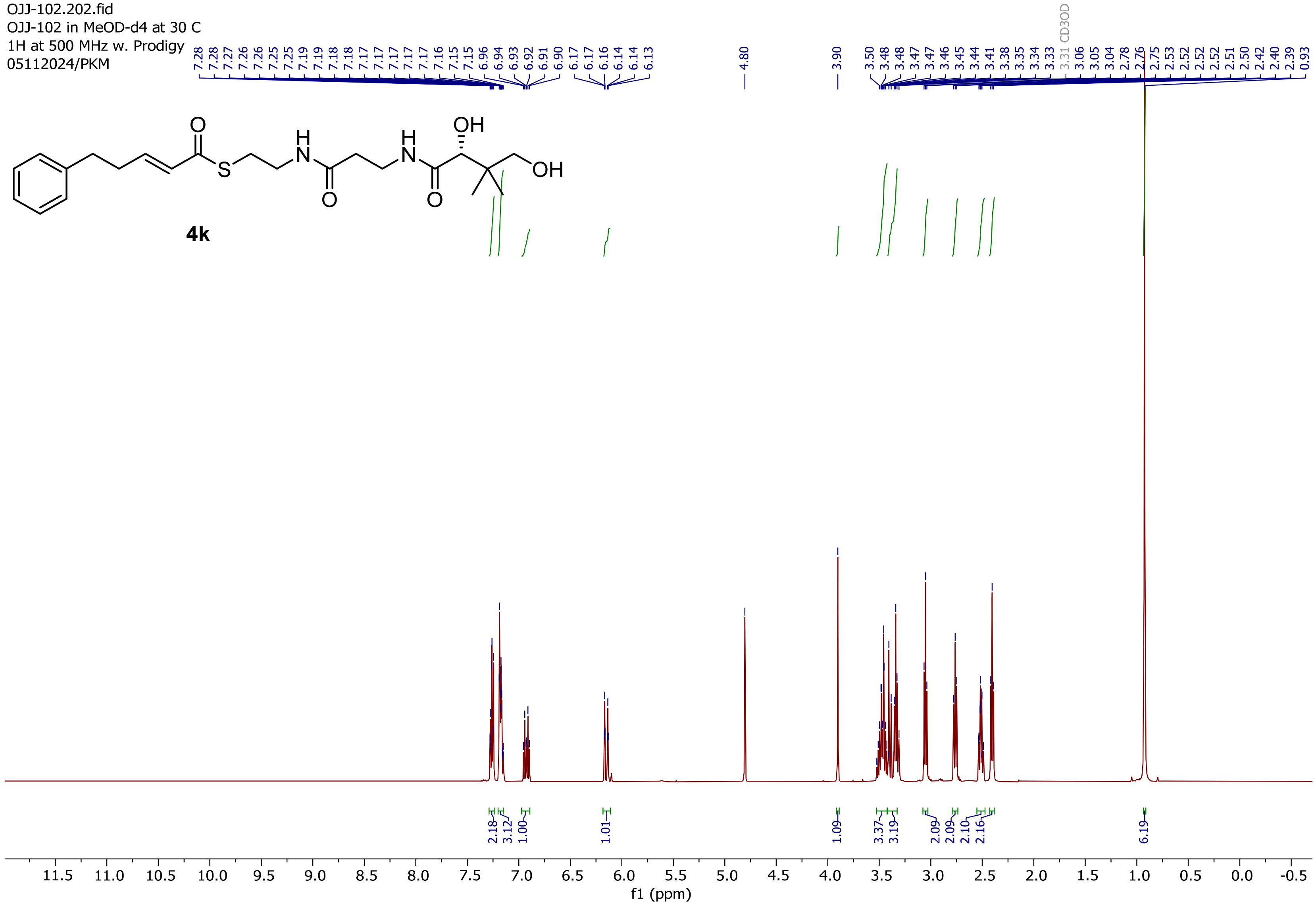

8.48 $^{13}C\{^1H\}$ NMR spectrum of 4k

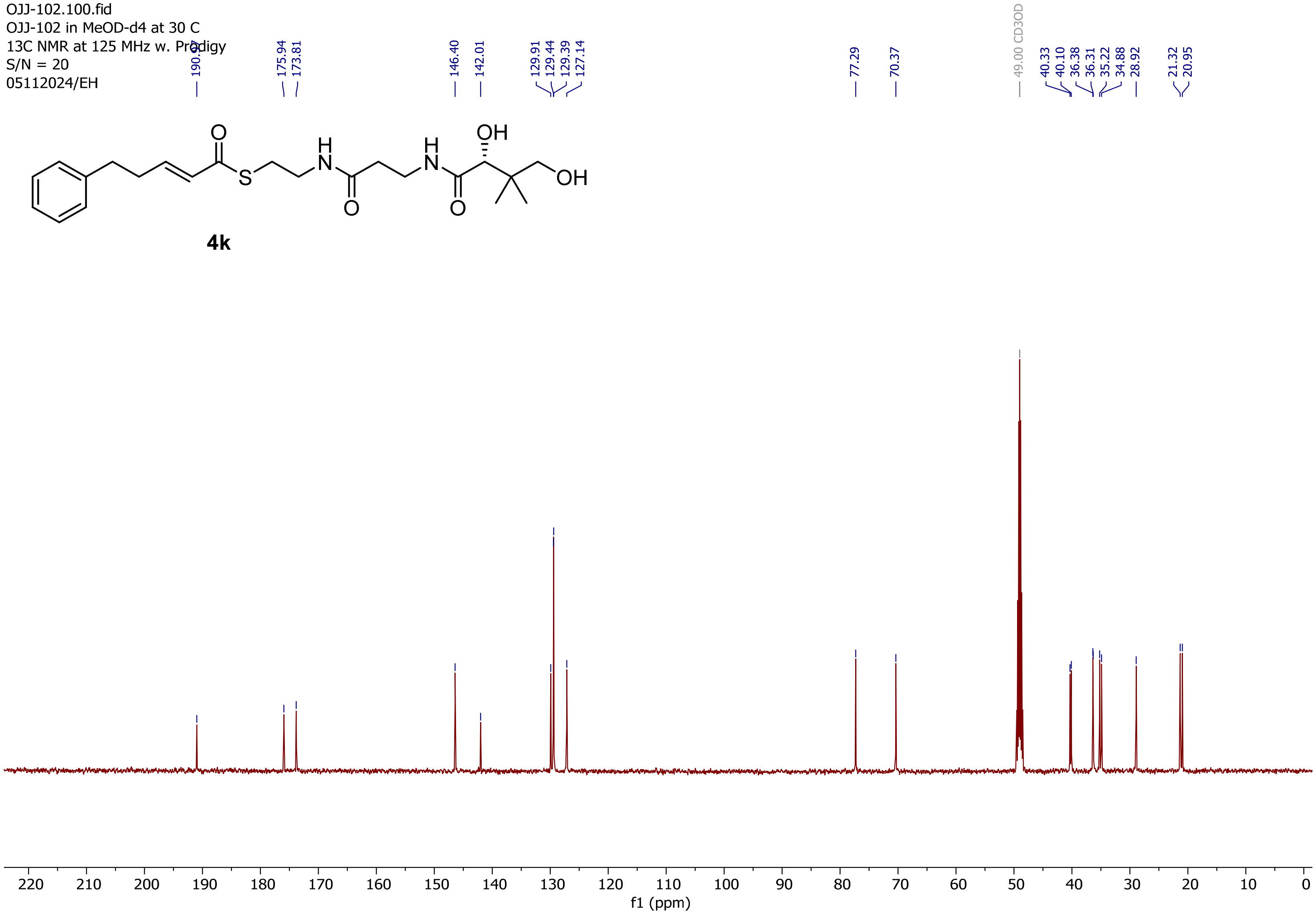

8.49 ¹H NMR spectrum of 6a

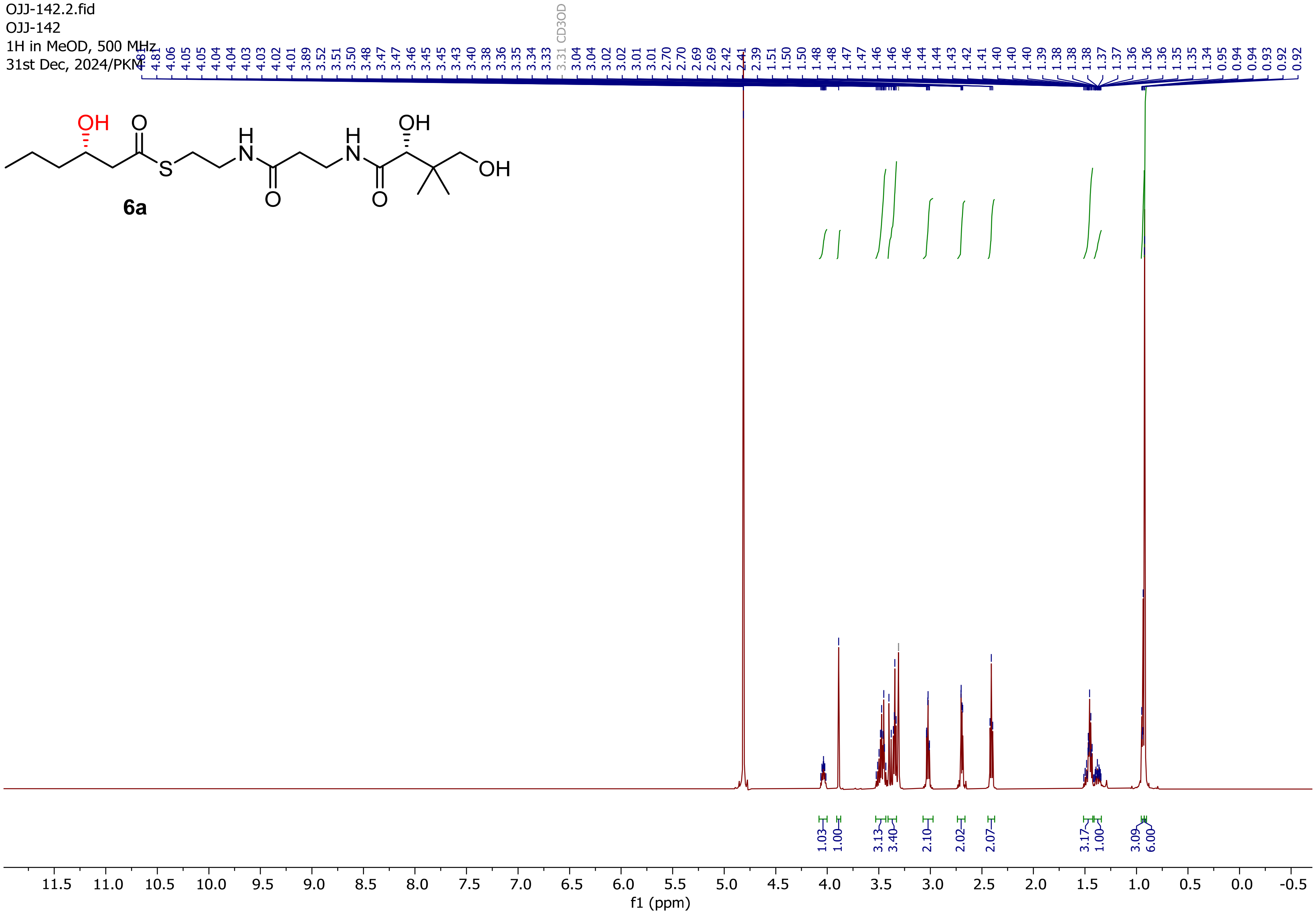

8.50 $^{13}C\{^1H\}$ NMR spectrum of 6a

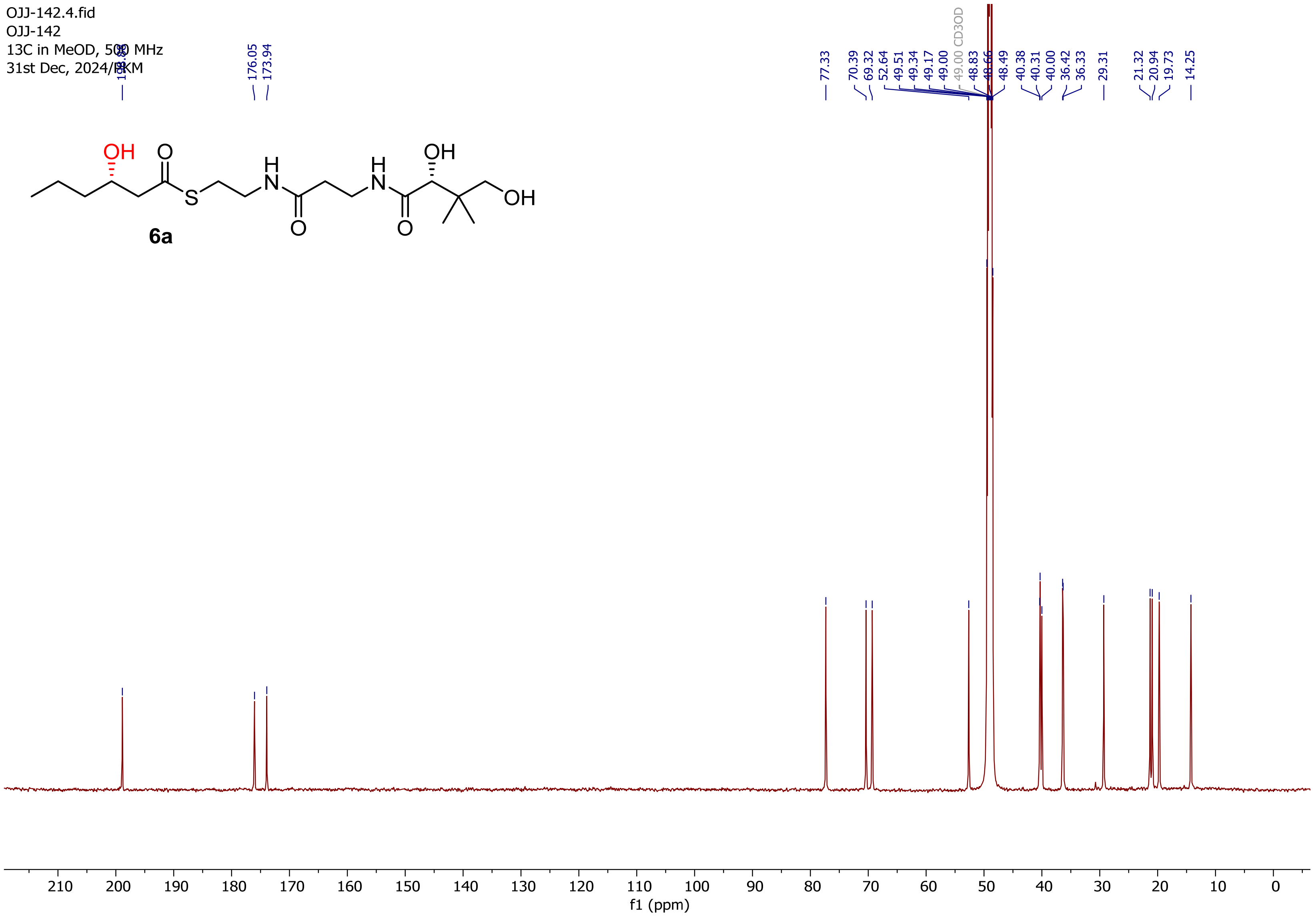

8.51 ^{1}H NMR spectrum of 6b

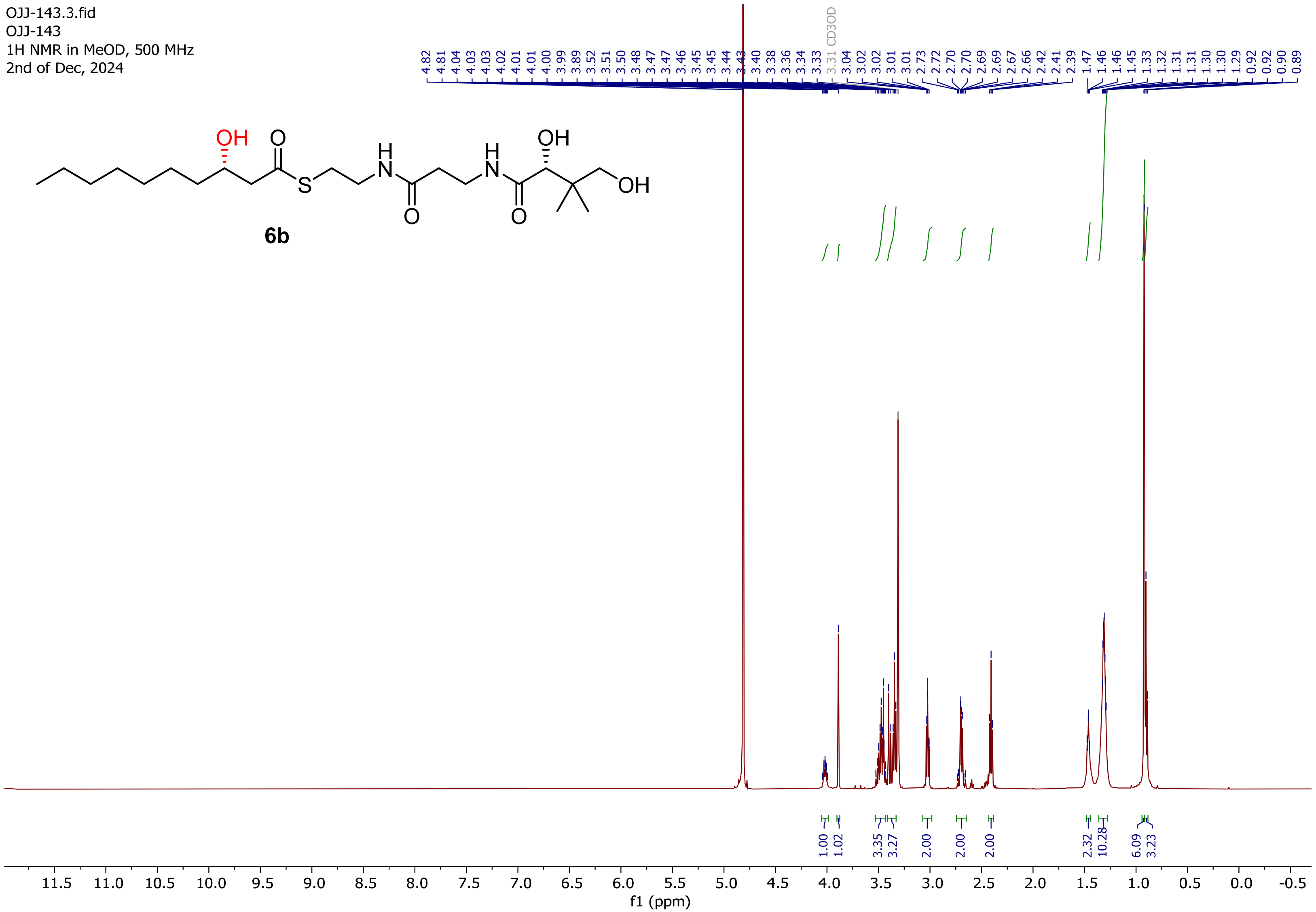

8.52 $^{13}C\{^{1}H\}$ NMR spectrum of 6b

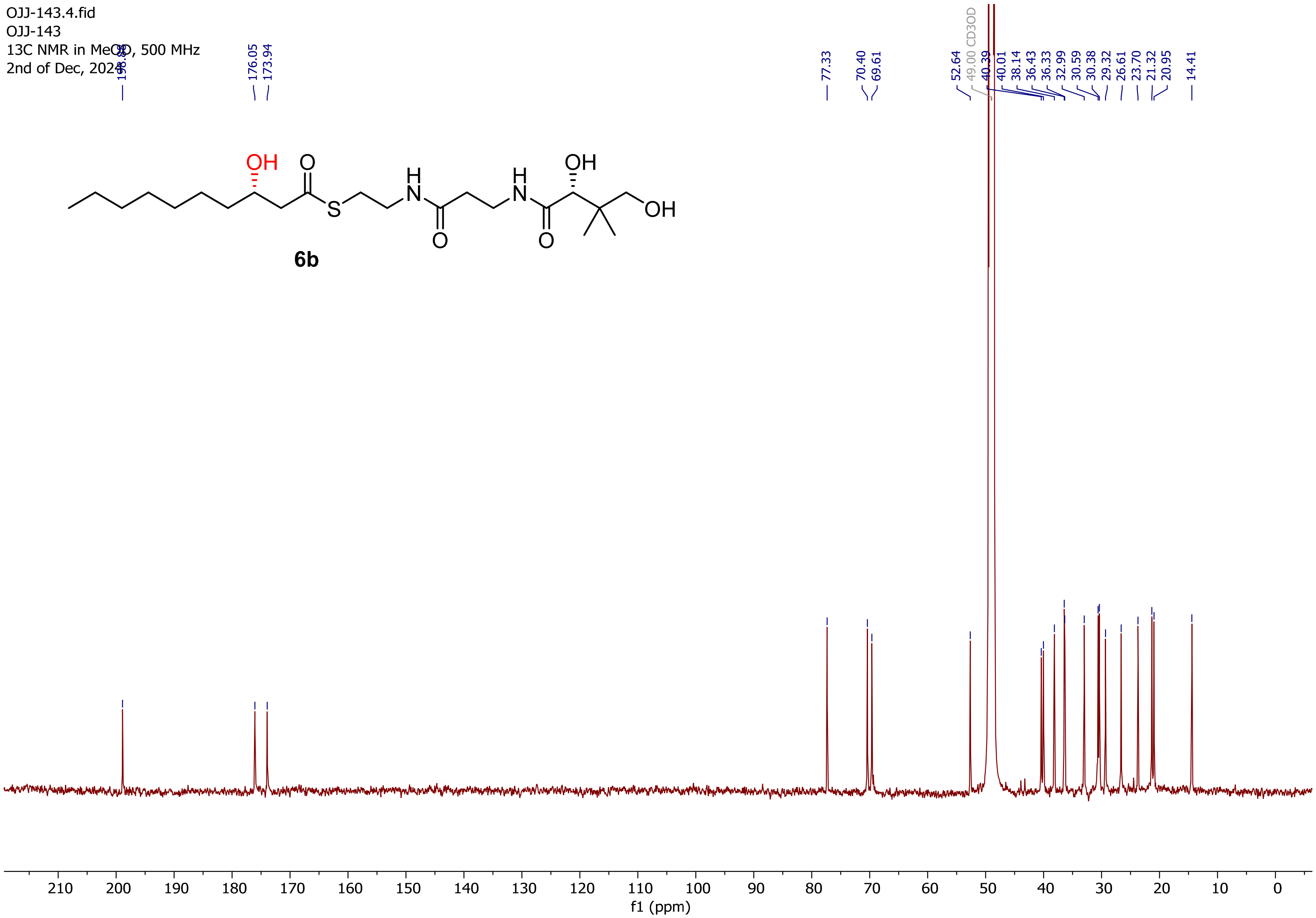

8.53 ^{1}H NMR spectrum of 6c

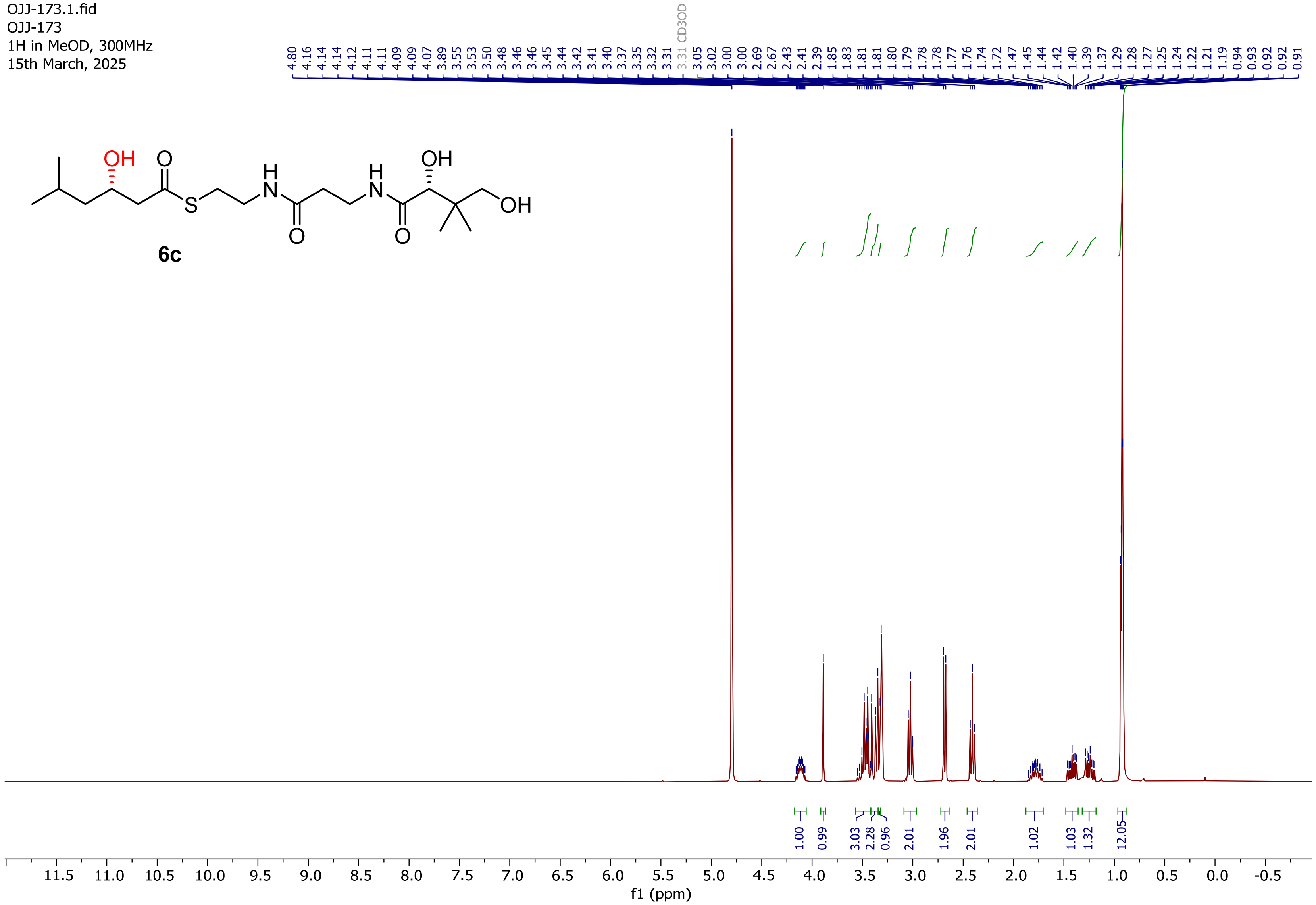

8.54 $^{13}C\{^{1}H\}$ NMR spectrum of 6c

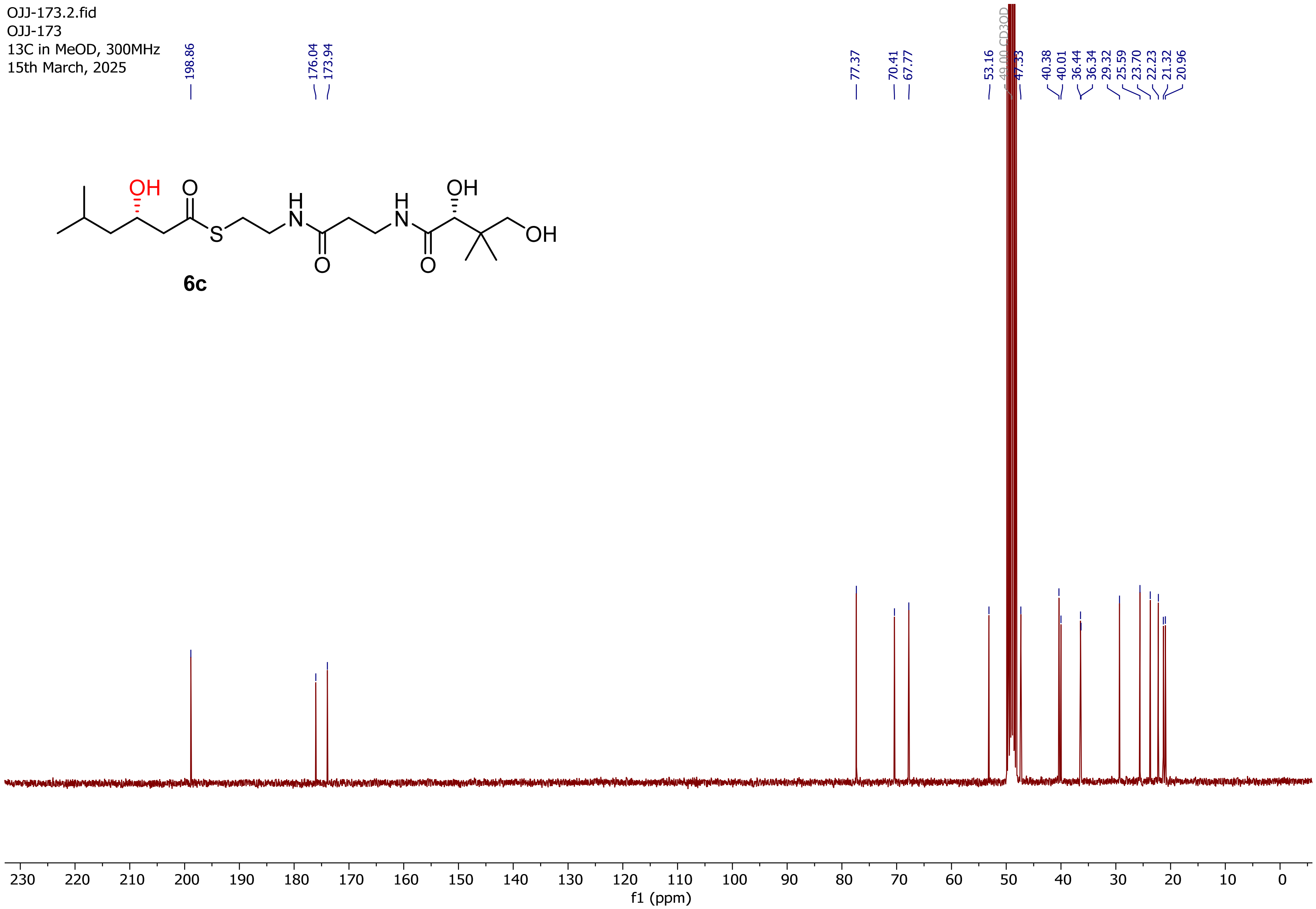

8.55 ^{1}H NMR spectrum of 6d

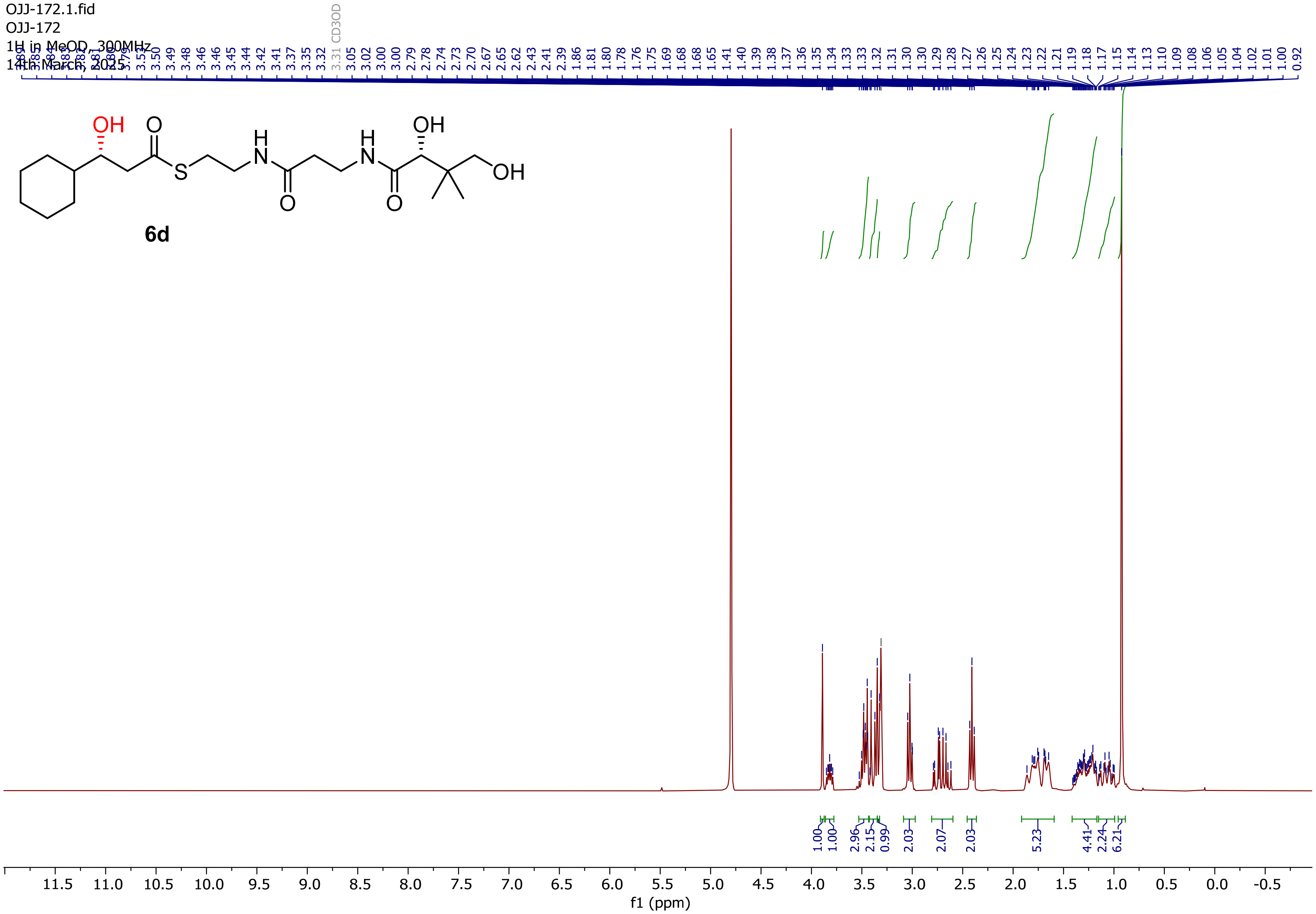

8.56 $^{13}C\{^{1}H\}$ NMR spectrum of 6d

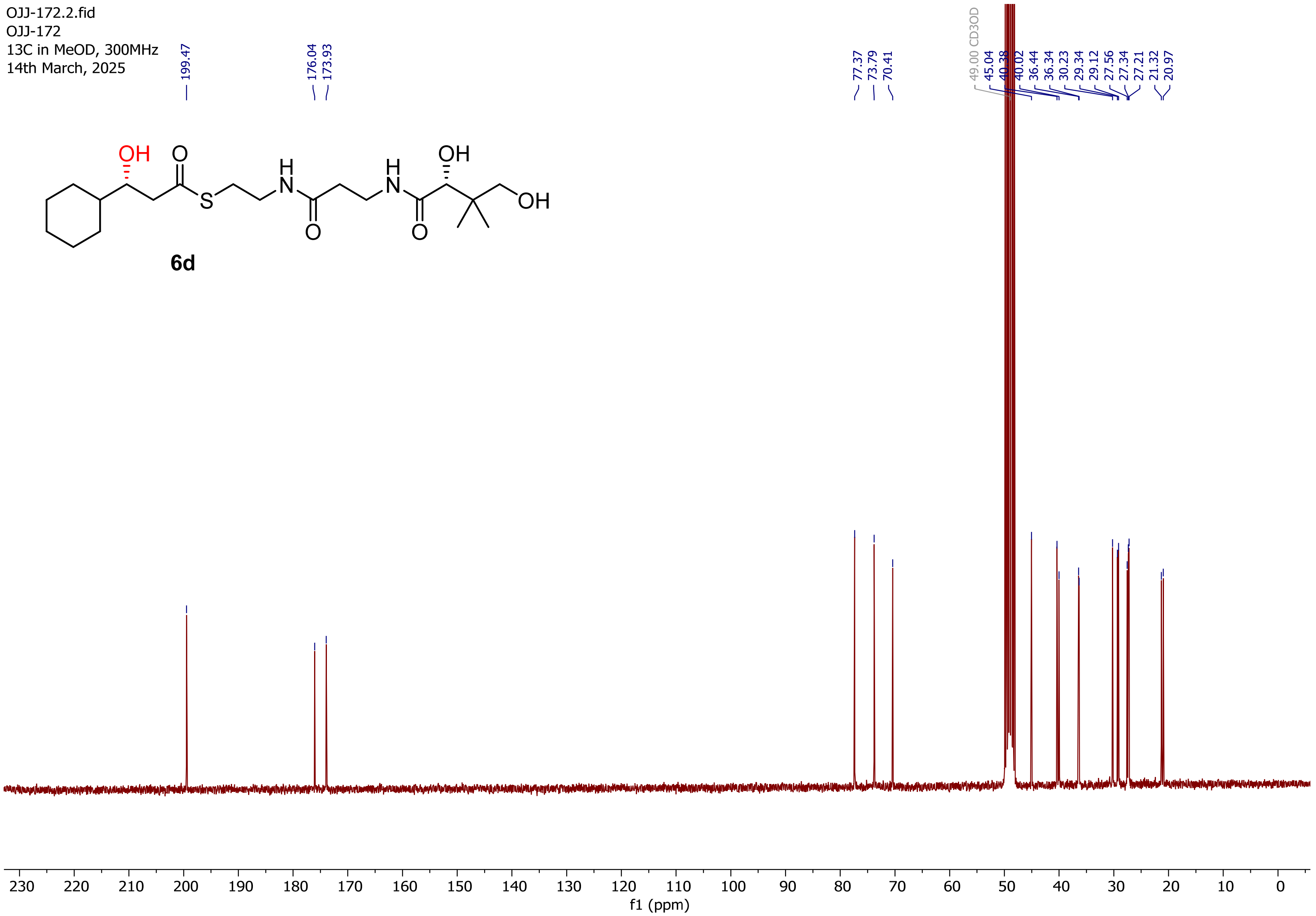

8.57 ^{1}H NMR spectrum of 6e

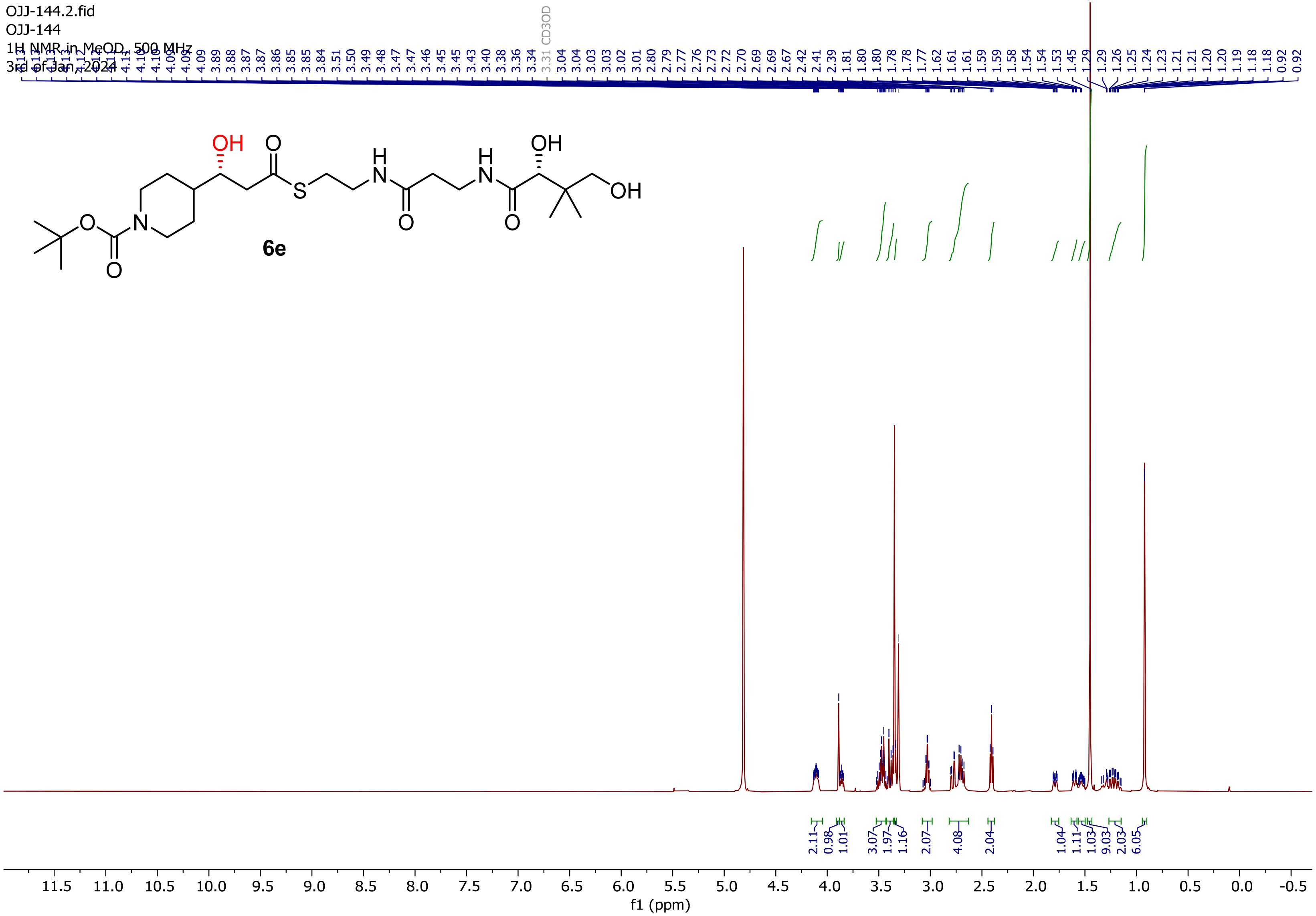

8.58 $^{13}C\{^1H\}$ NMR spectrum of 6e

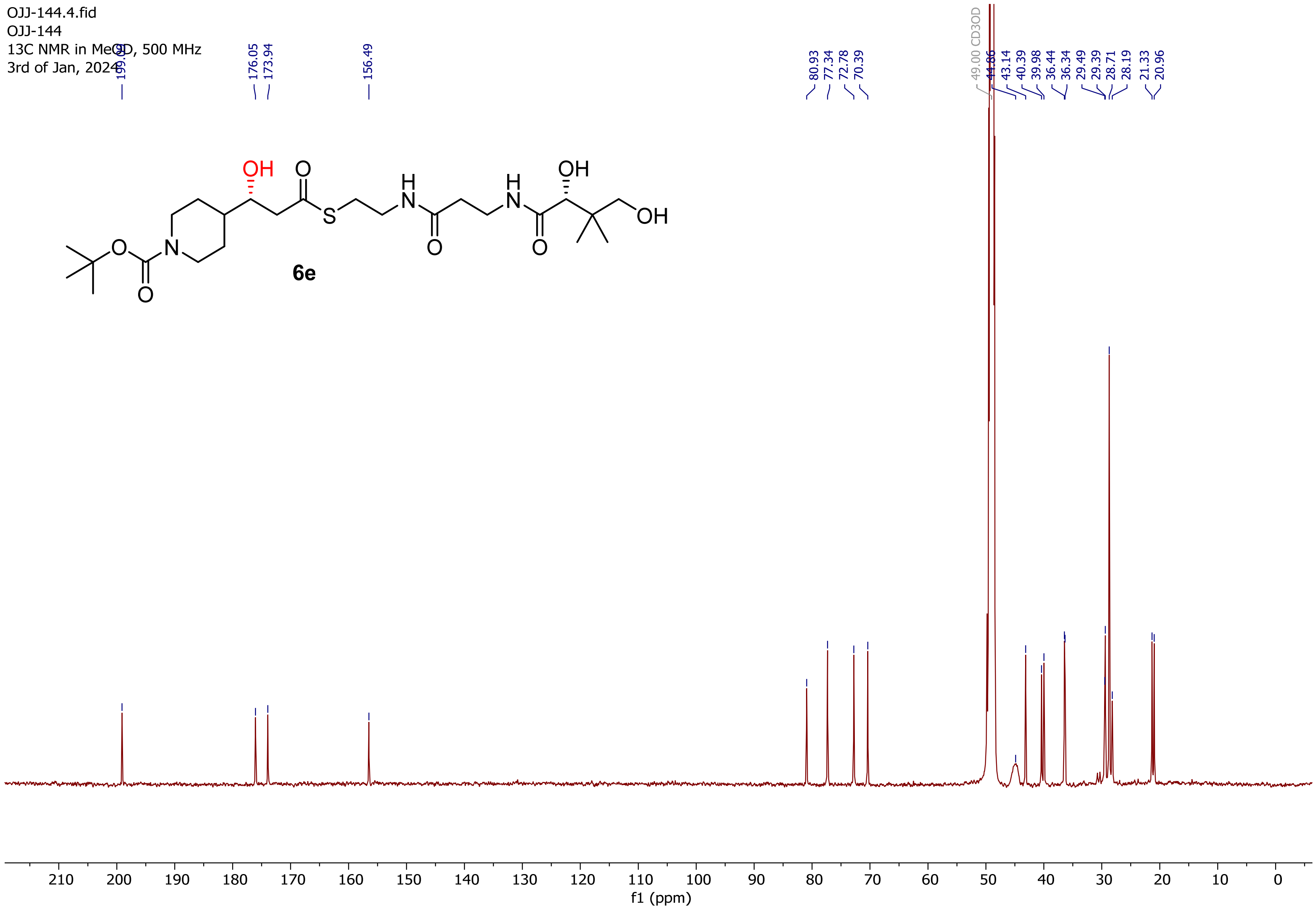

8.59 ^{1}H NMR spectrum of 6f

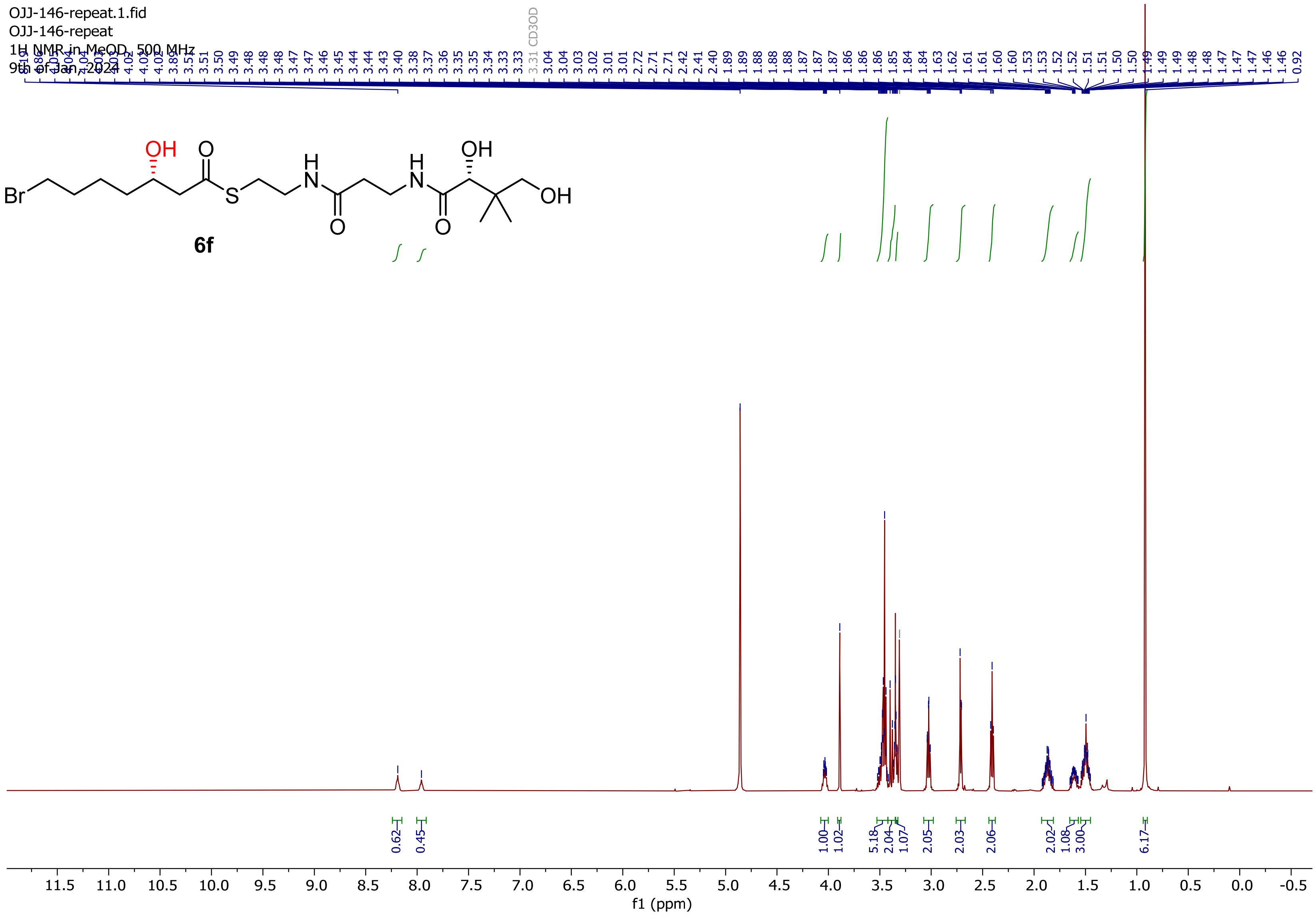

8.60 $^{13}C\{^{1}H\}$ NMR spectrum of 6f

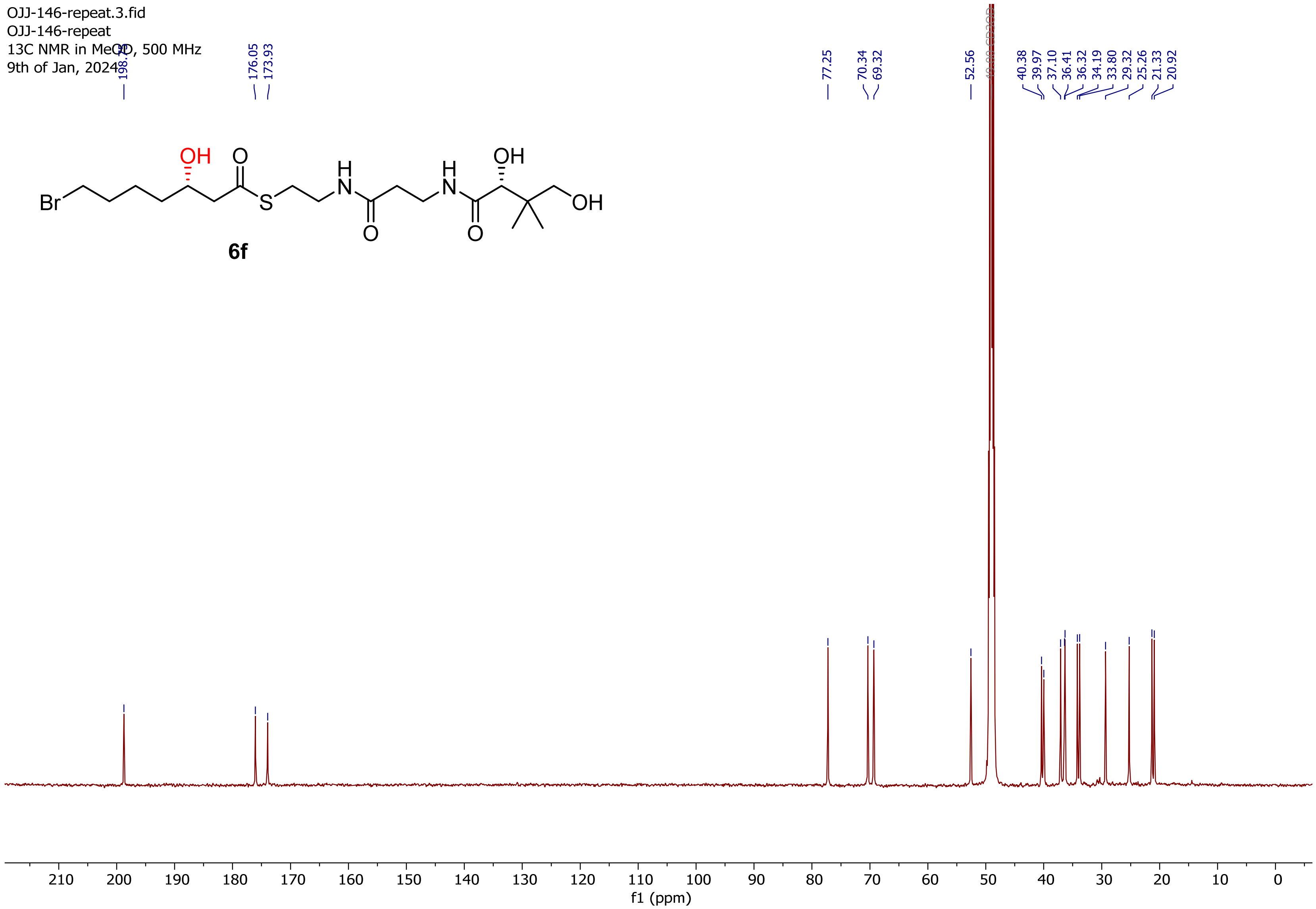

8.61 ¹H NMR spectrum of 6g

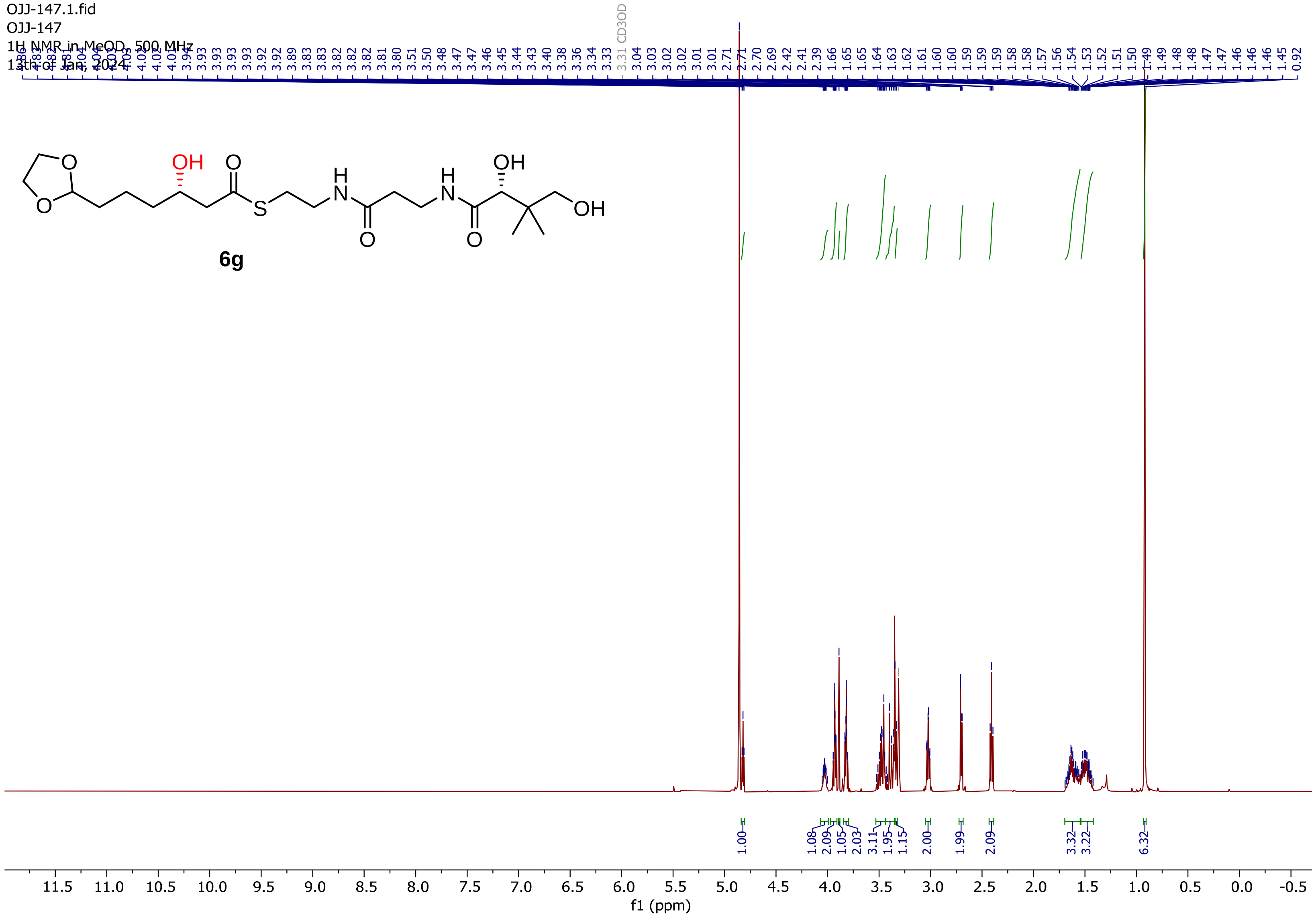

8.62 $^{13}C\{^{1}H\}$ NMR spectrum of 6g

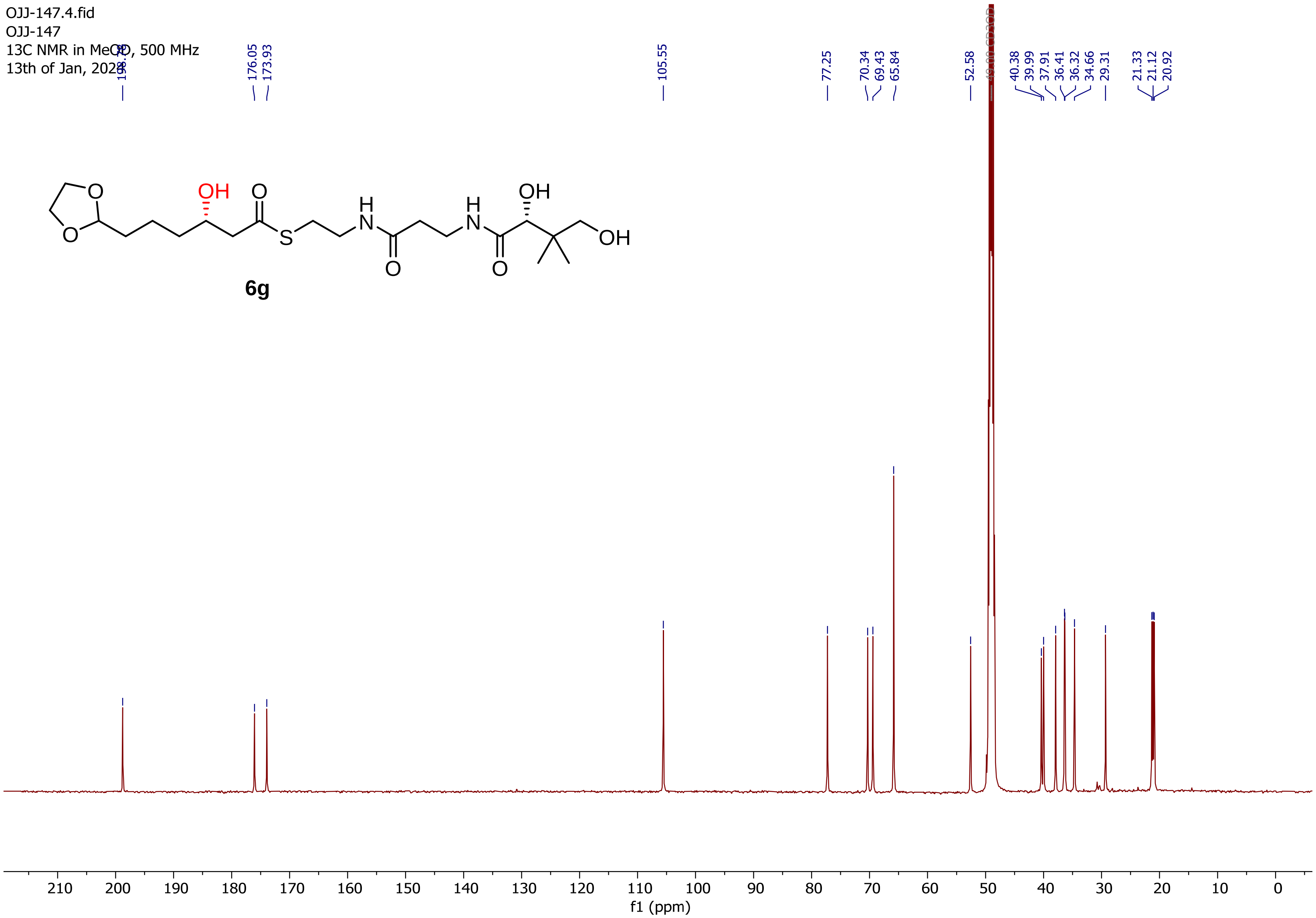

8.63 ^{1}H NMR spectrum of 6h

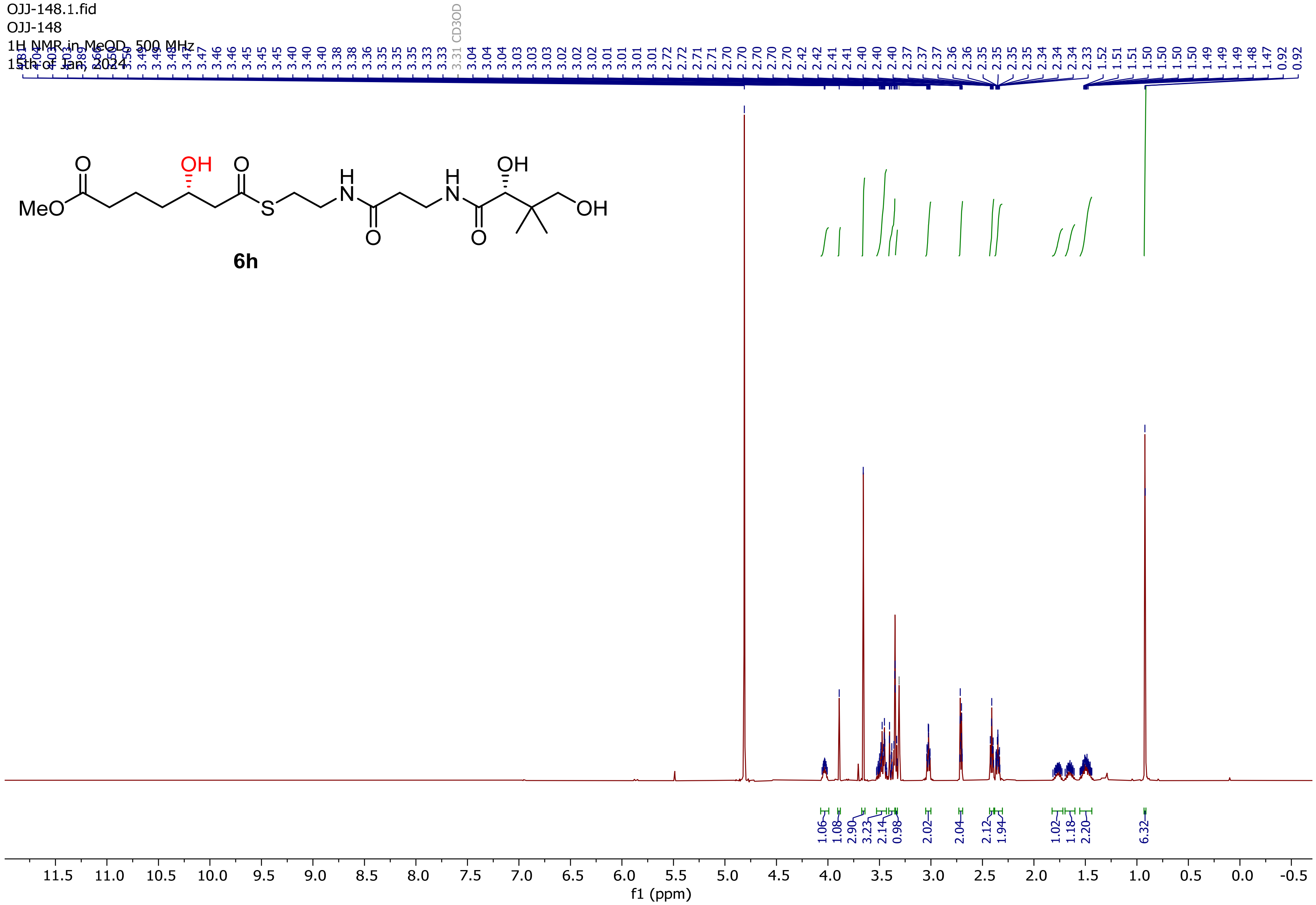

8.64 $^{13}C\{^1H\}$ NMR spectrum of 6h

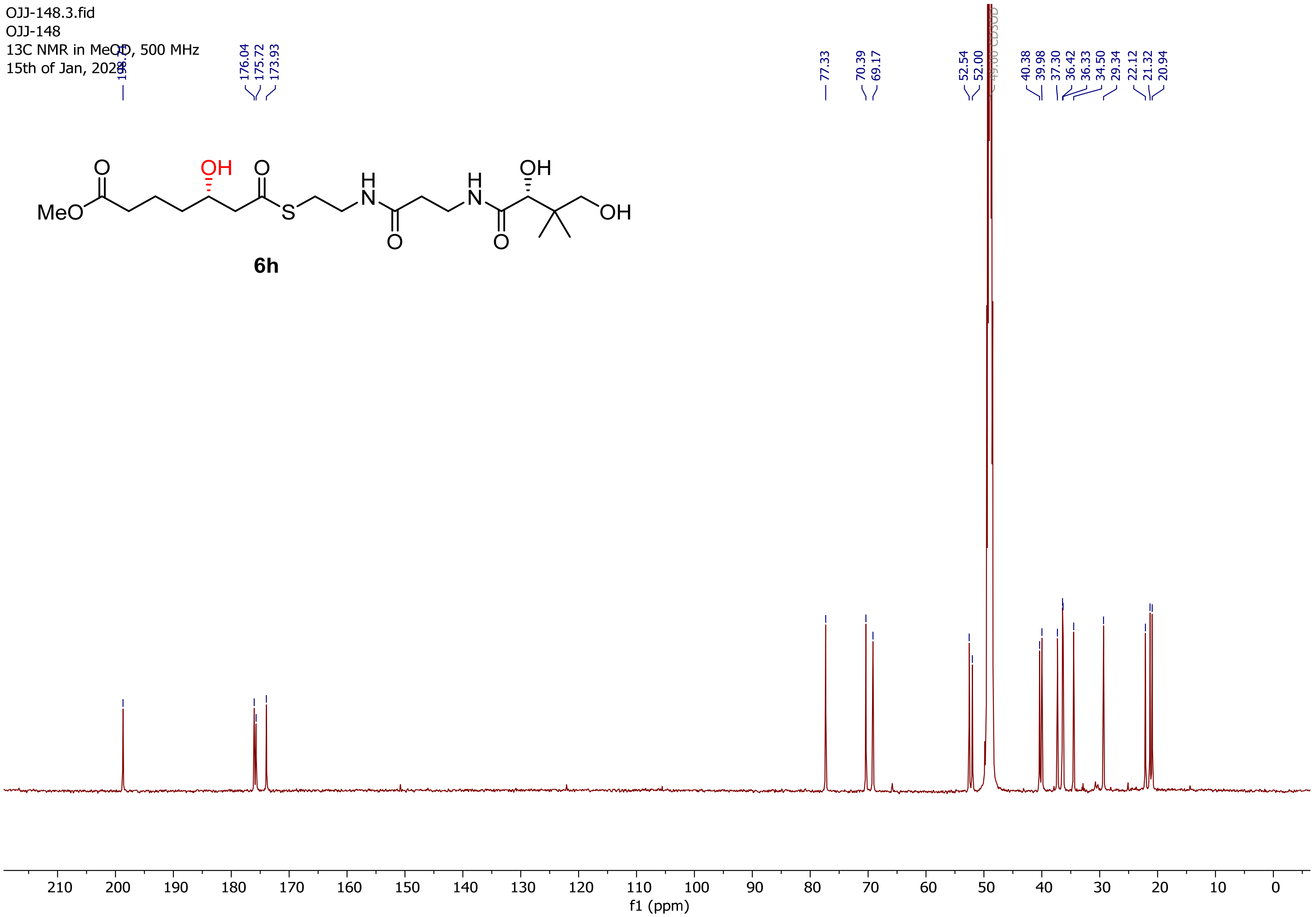

8.65 ^{1}H NMR spectrum of 6i

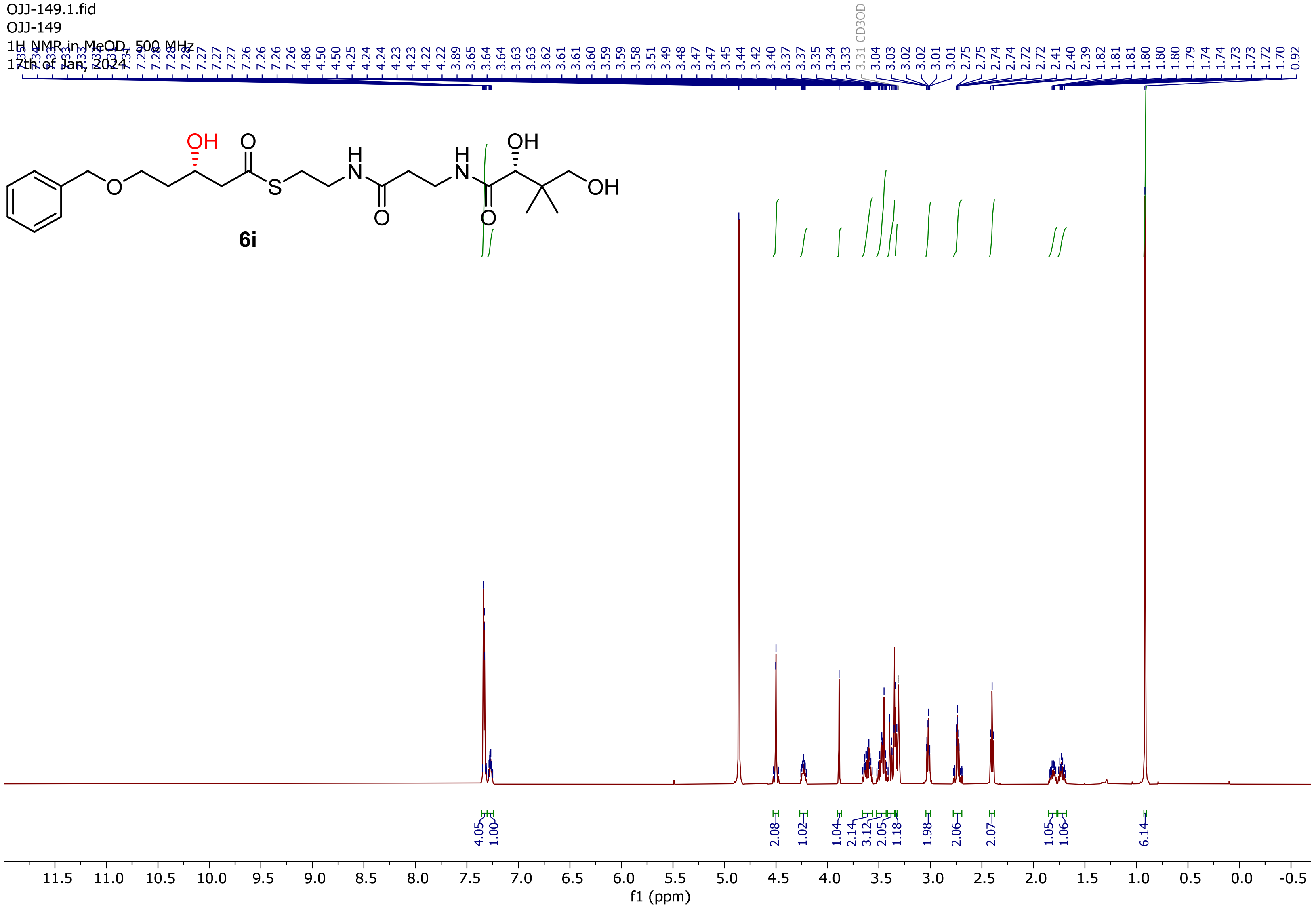

8.66 $^{13}C\{^{1}H\}$ NMR spectrum of 6i

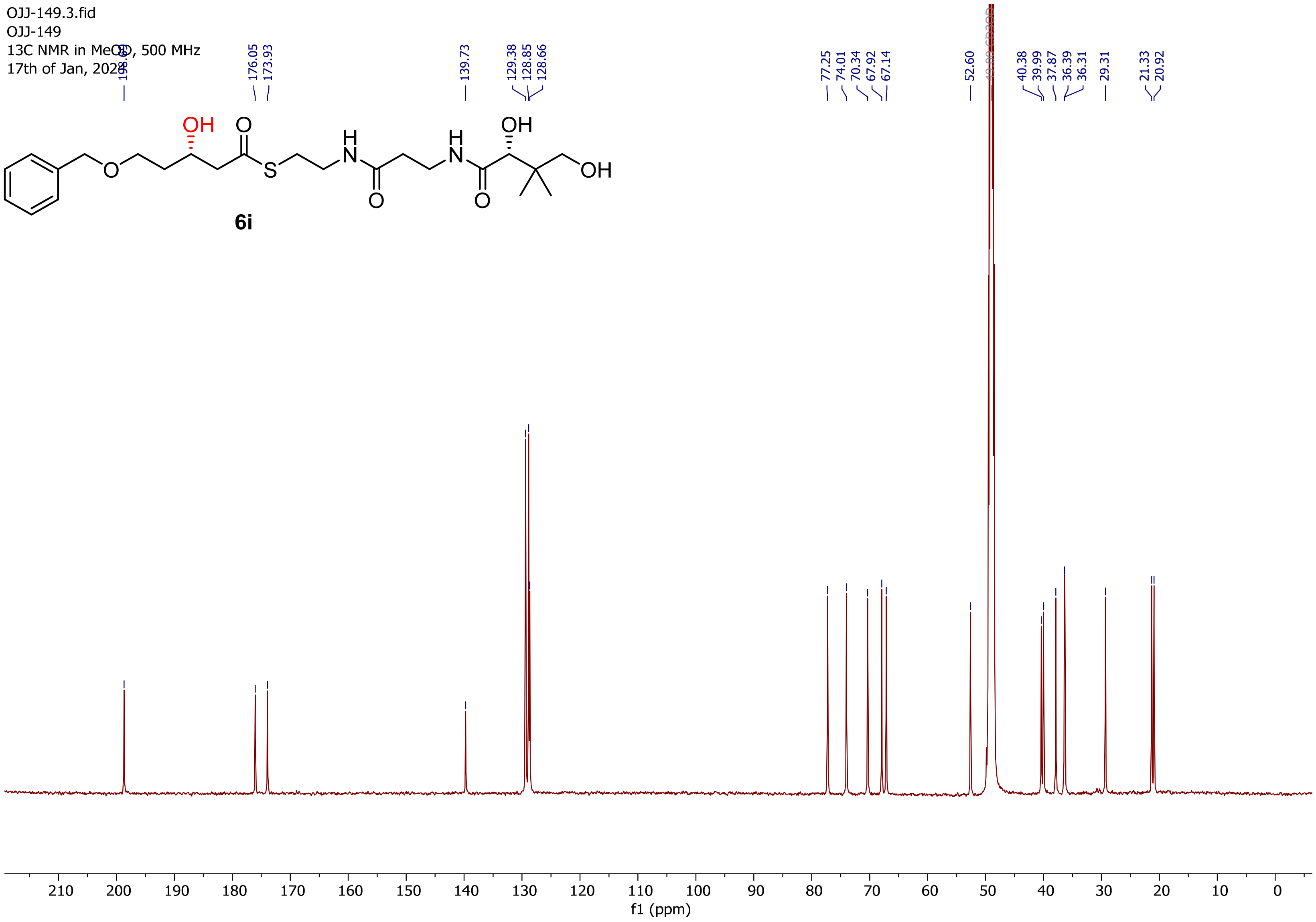

8.67 ^{1}H NMR spectrum of 6j

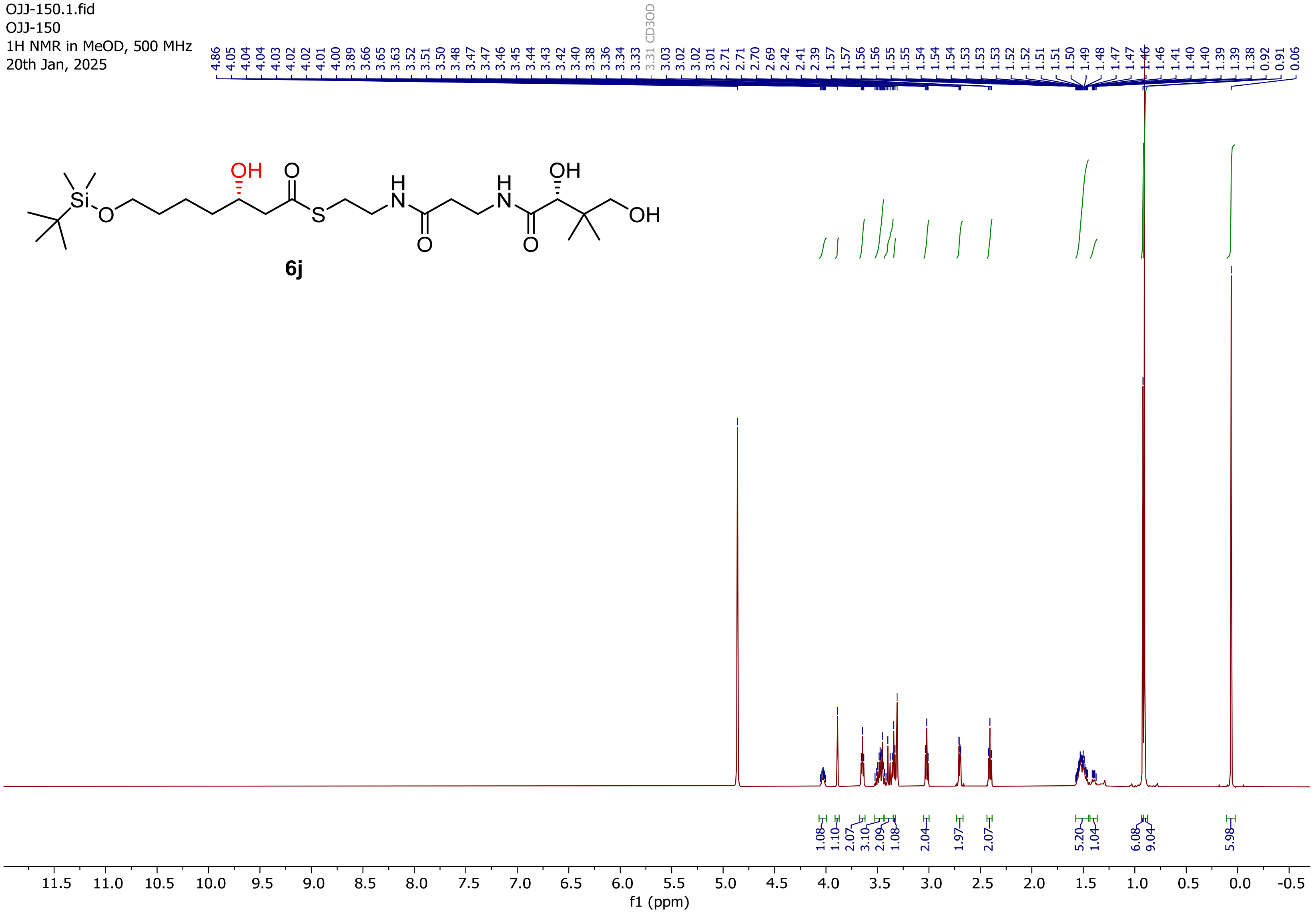

8.68 $^{13}C\{^1H\}$ NMR spectrum of 6j

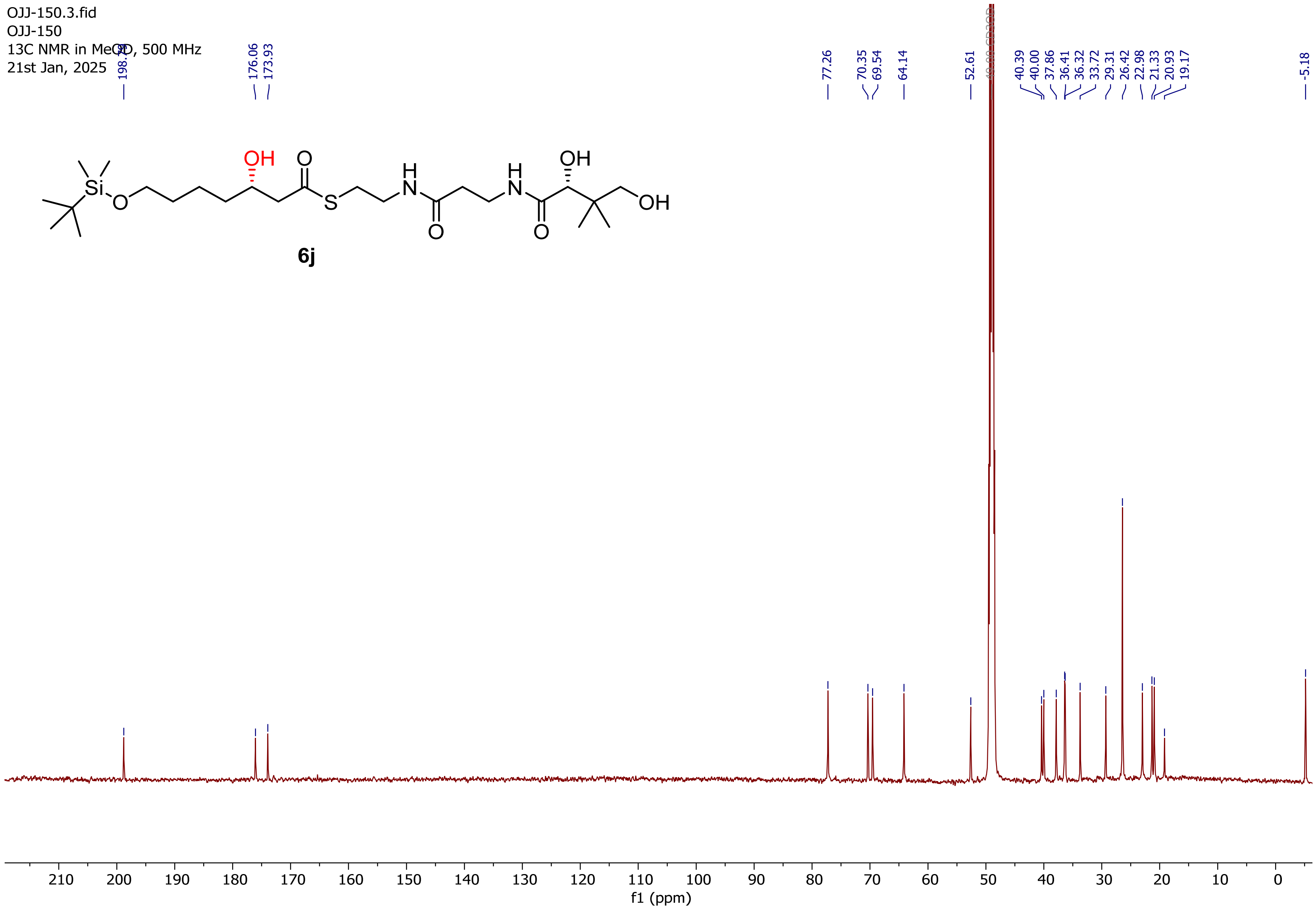

8.69 ^{1}H NMR spectrum of 6k

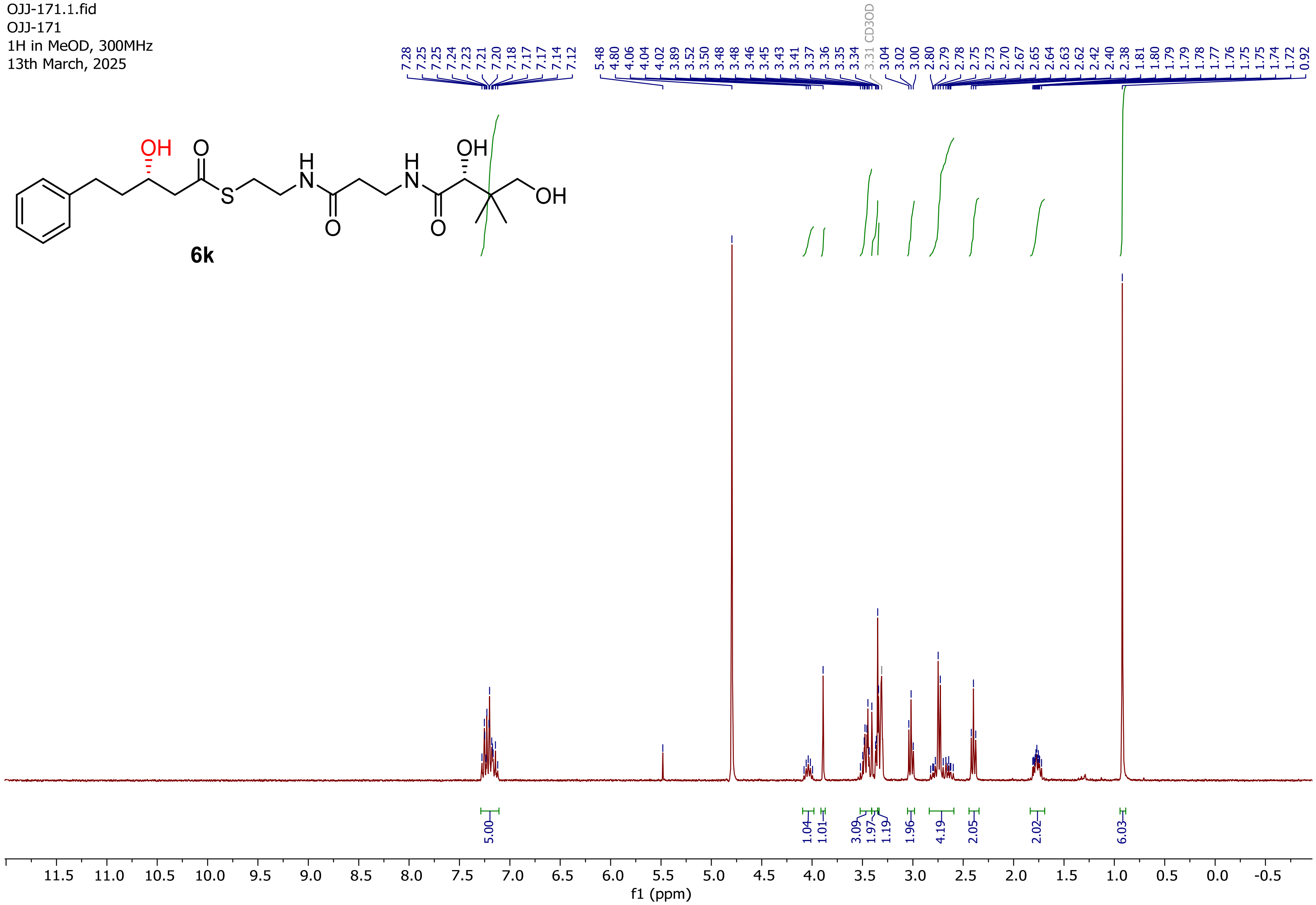

8.70 $^{13}C\{^{1}H\}$ NMR spectrum of 6k

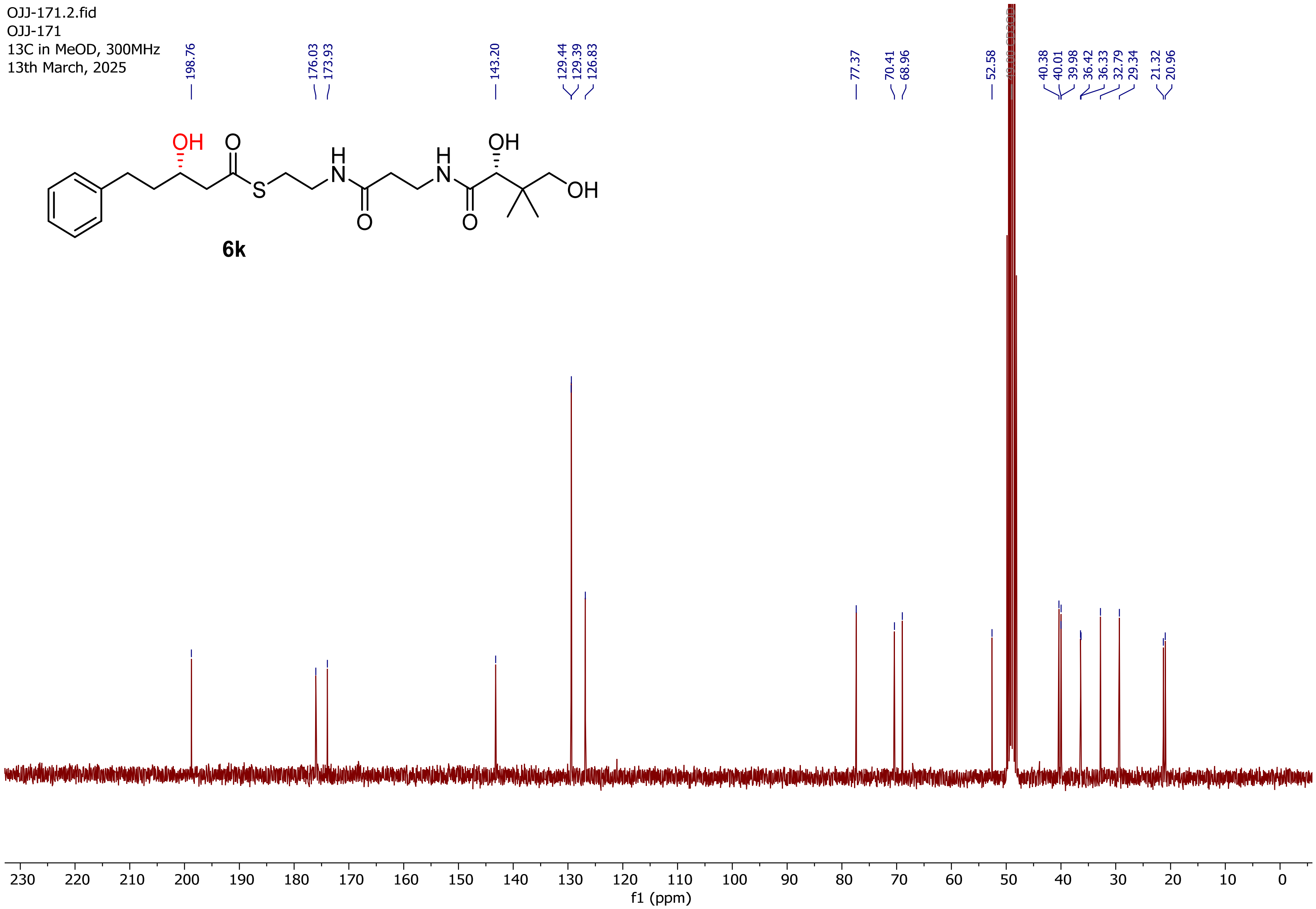

6k

8.71 ^{1}H NMR spectrum of S19b

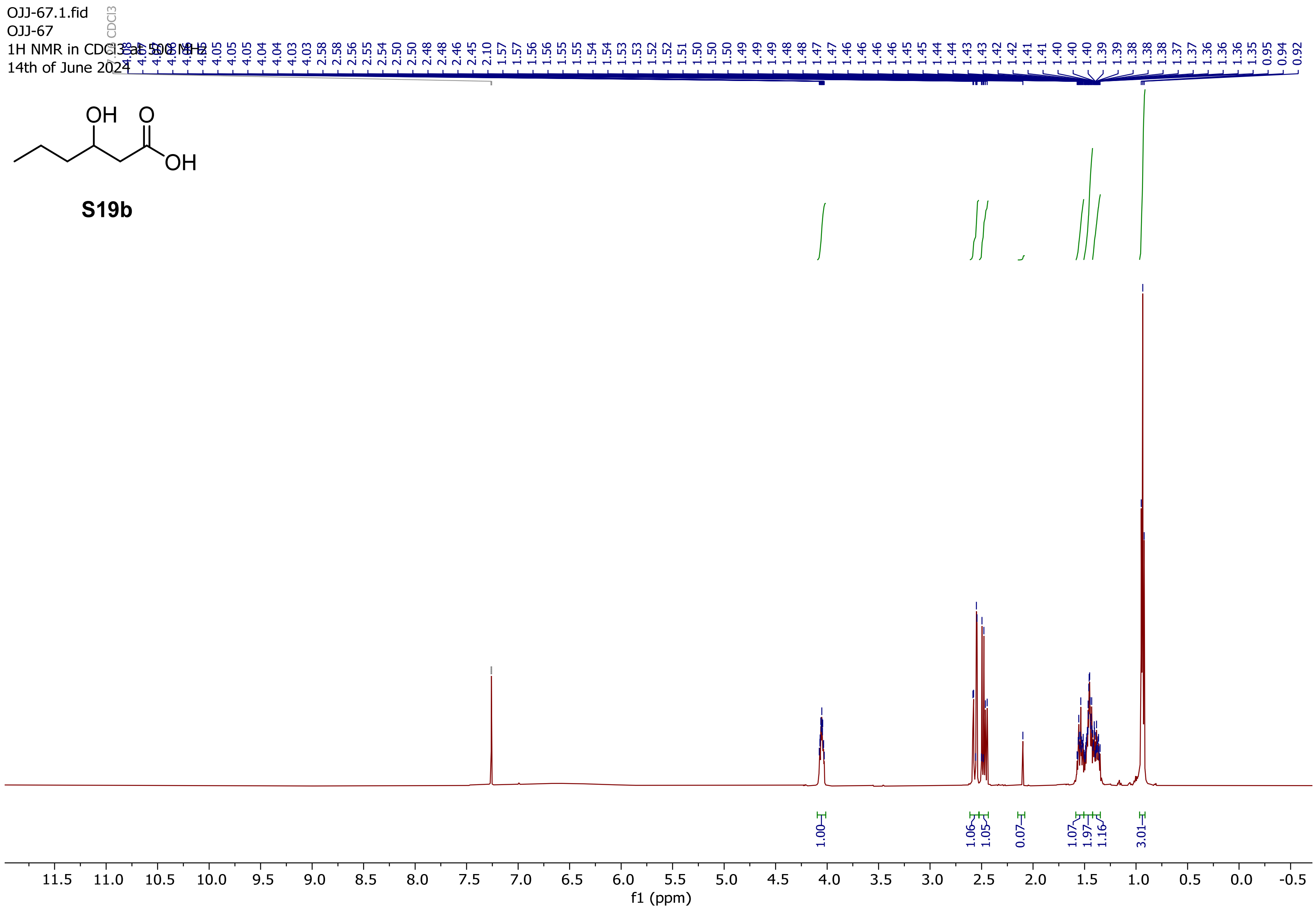

8.72 $^{13}C\{^{1}H\}$ NMR spectrum of S19b

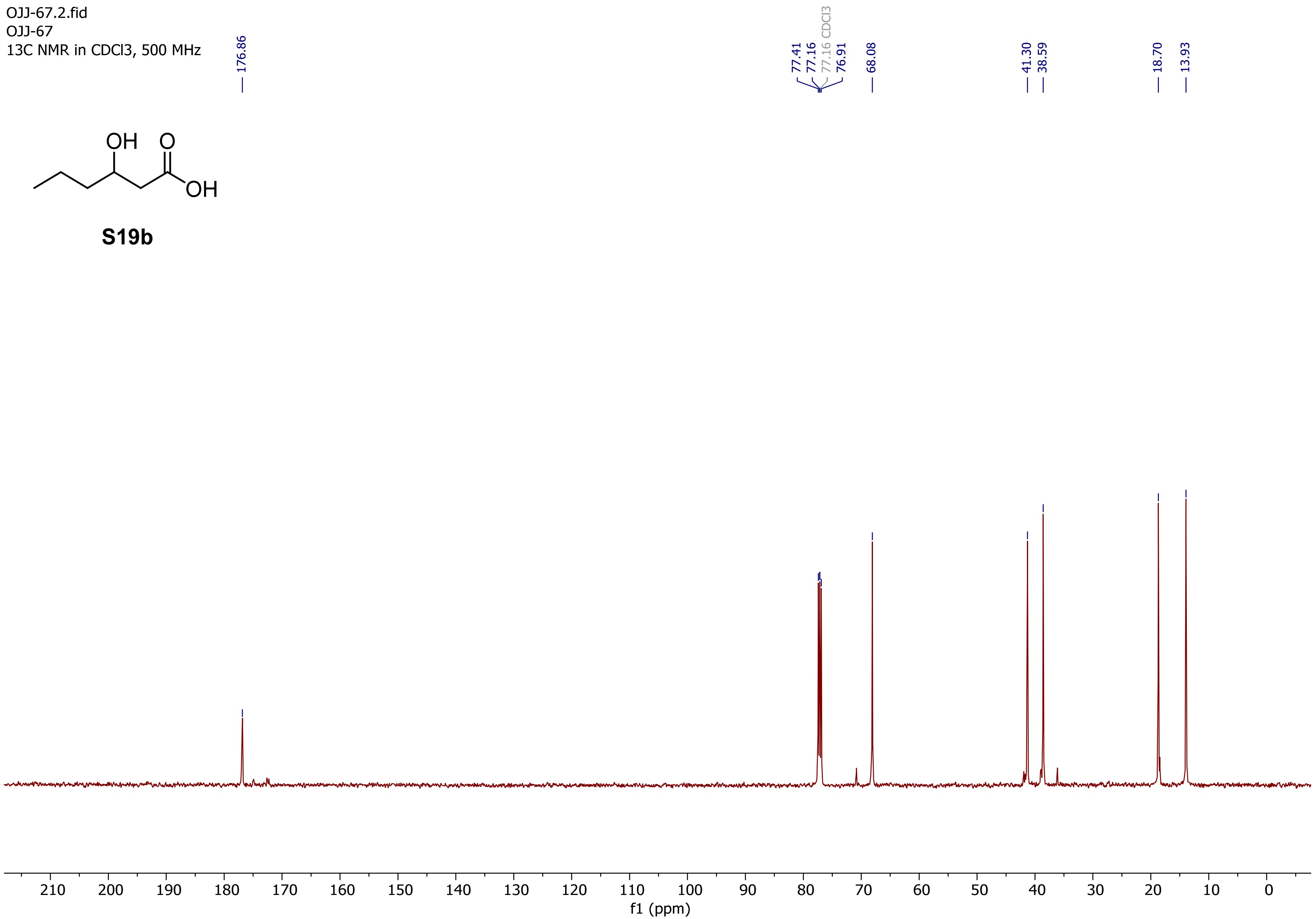

S19b

8.73 ^{1}H NMR spectrum of 6’a

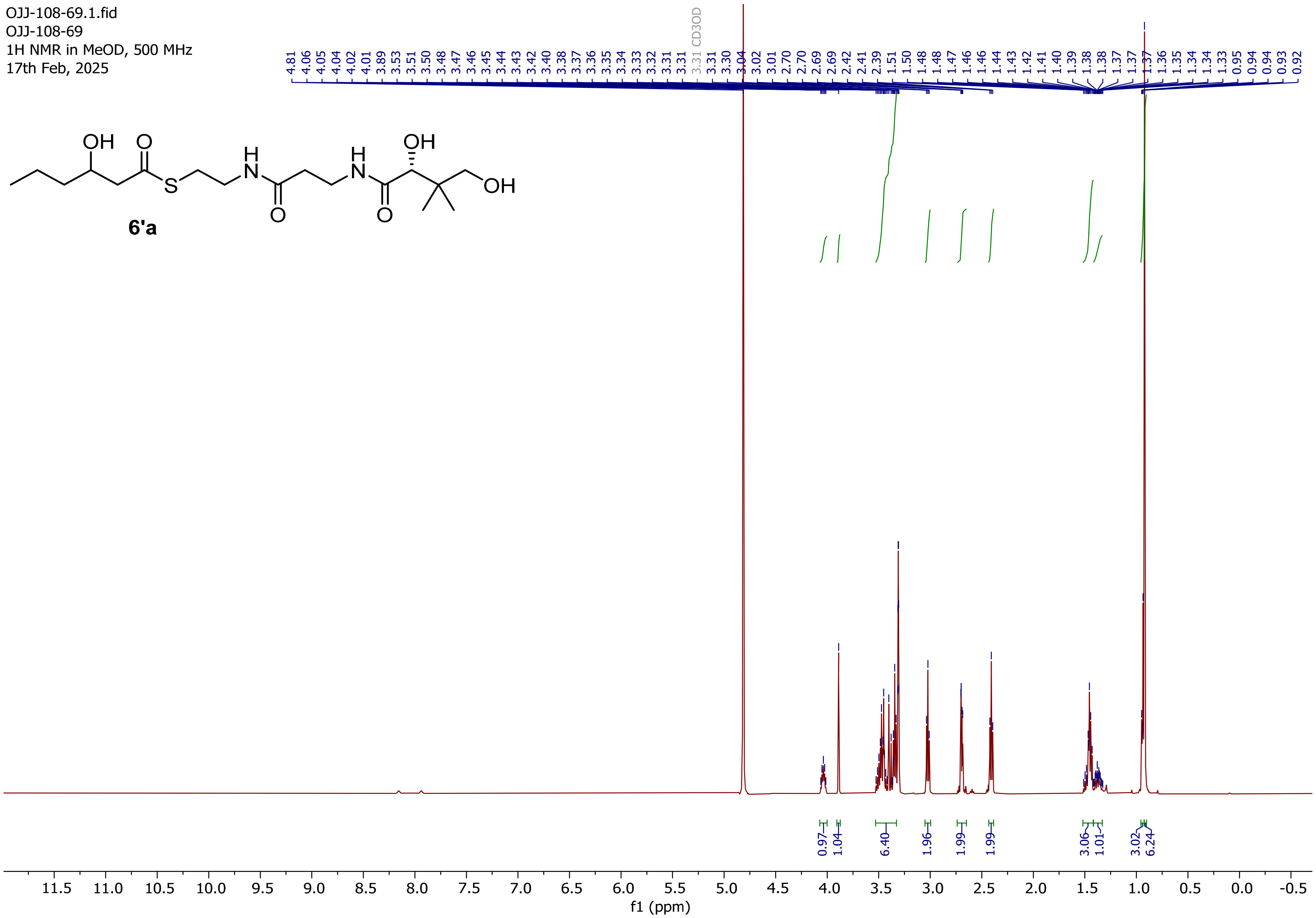

8.74 $^{13}C\{^{1}H\}$ NMR spectrum of 6'a

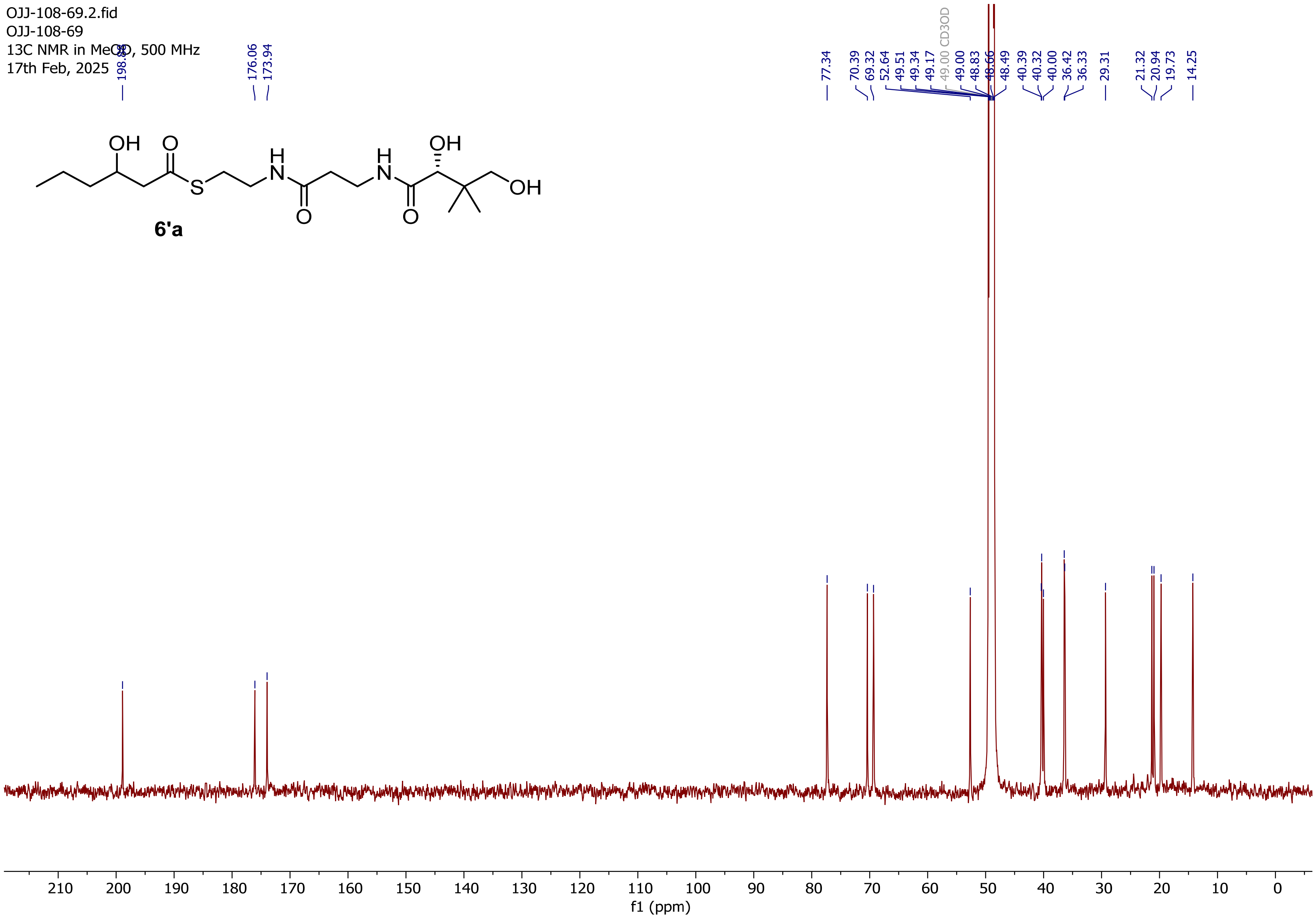

8.75 ^{1}H NMR spectrum of *S*20b

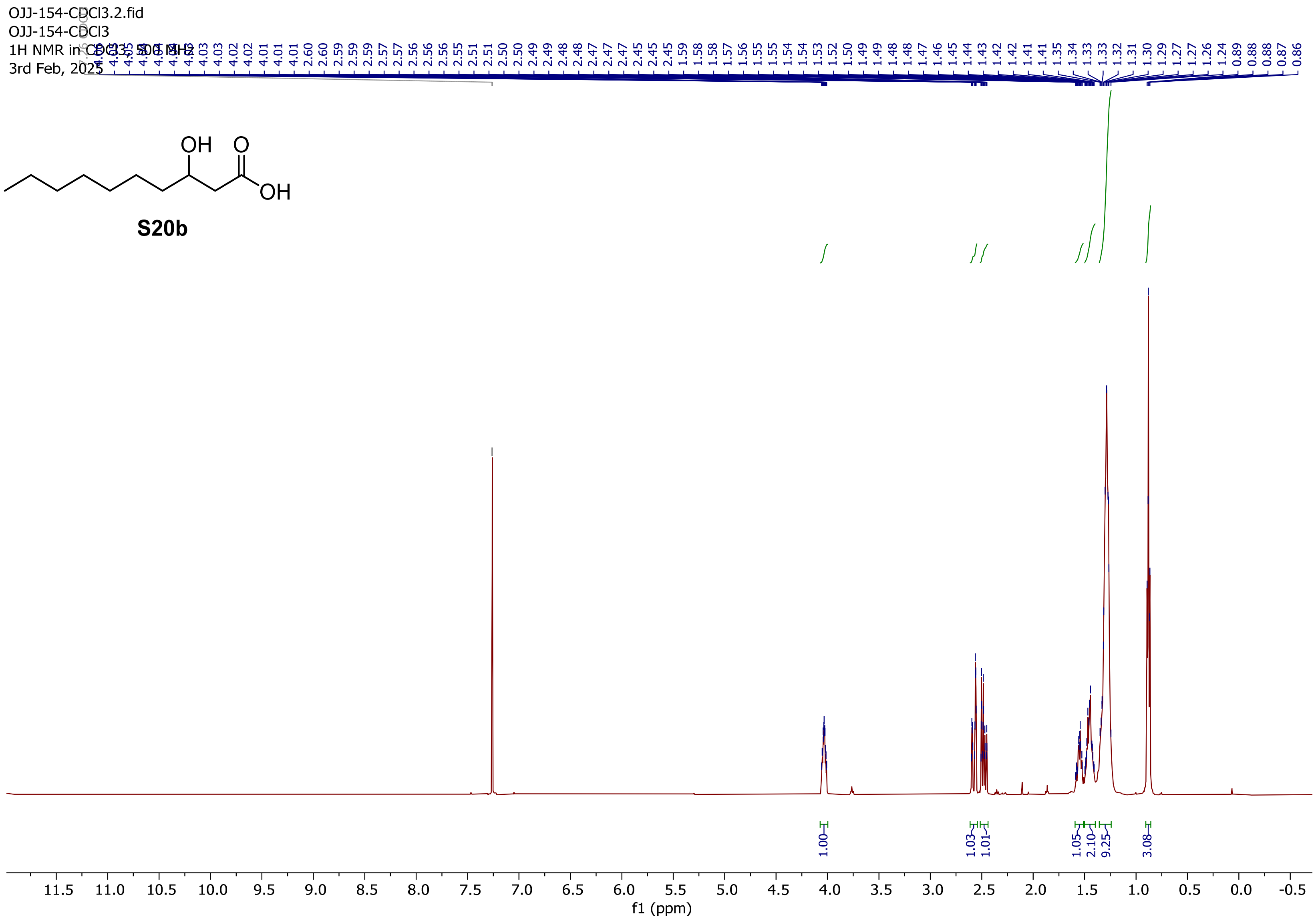

8.76 $^{13}C\{^1H\}$ NMR spectrum of S20b

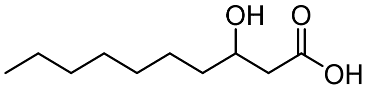

S20b

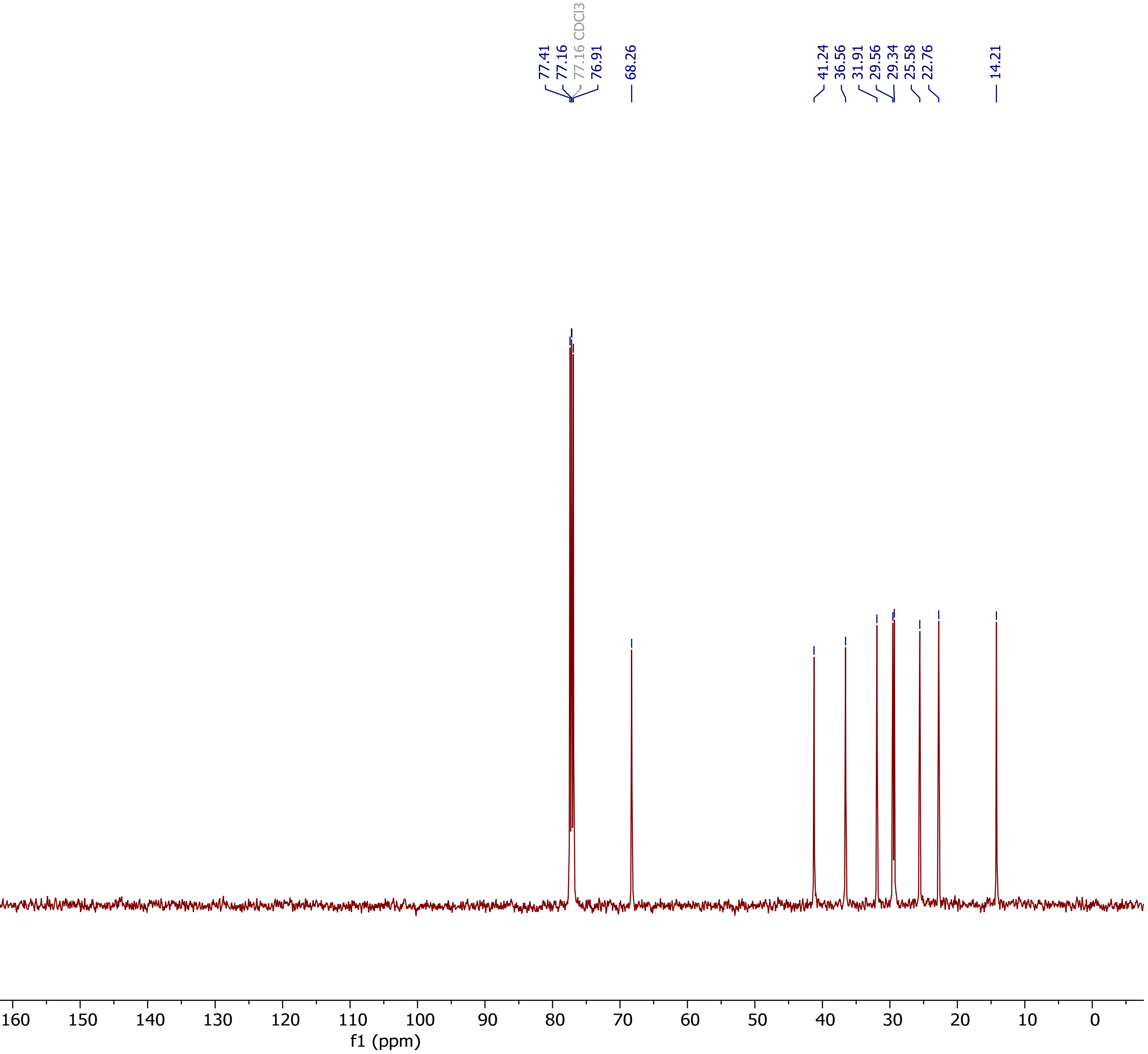

8.77 ^{1}H NMR spectrum of 6'b

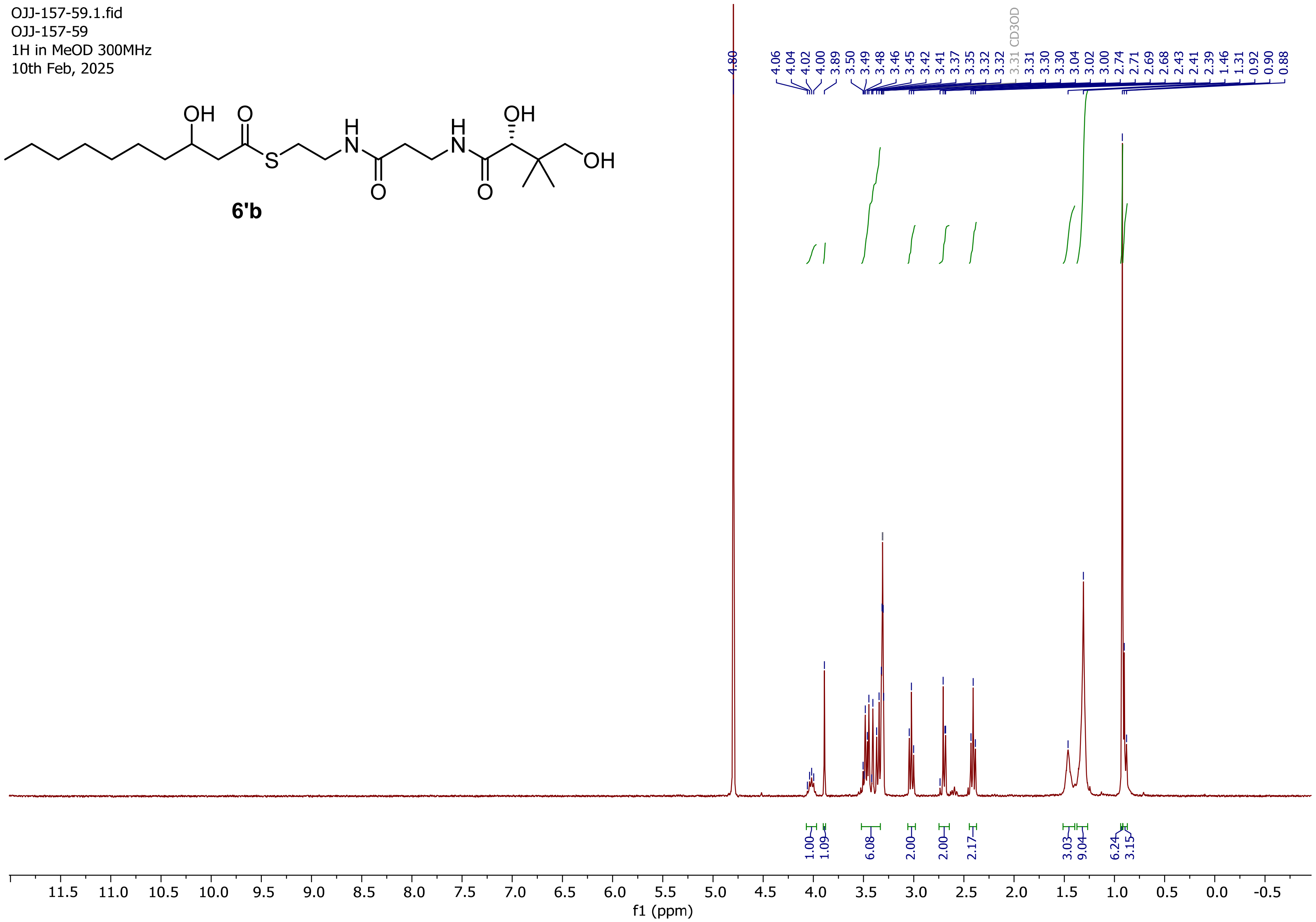

8.78 $^{13}C\{^1H\}$ NMR spectrum of 6’b

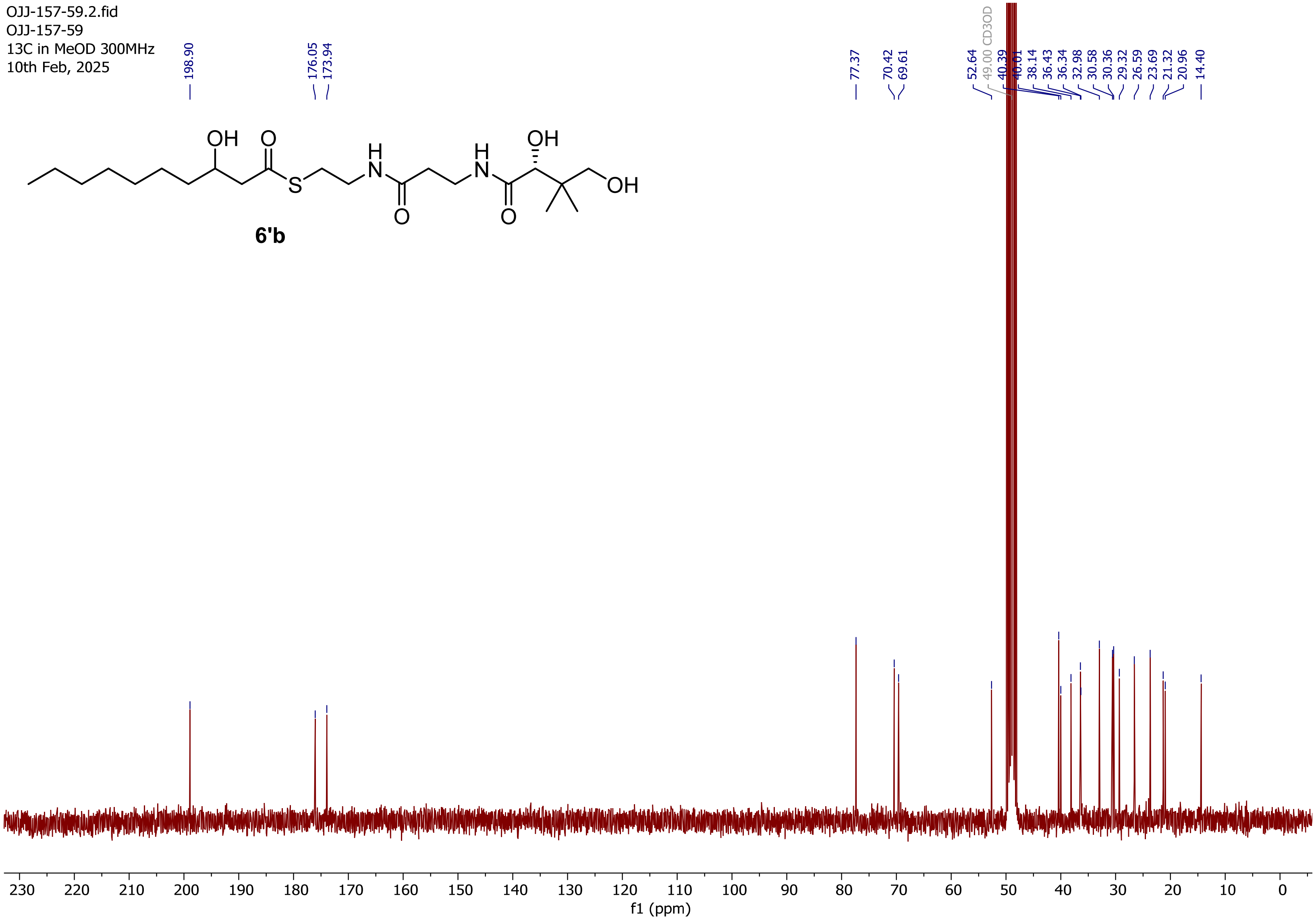

8.79 ^{1}H NMR spectrum of S21b

OJJ-191.1.fid
OJJ-191
1H in CDCl3, 300MHz
19th of May, 2025

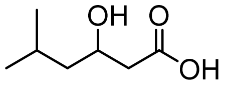

S21b

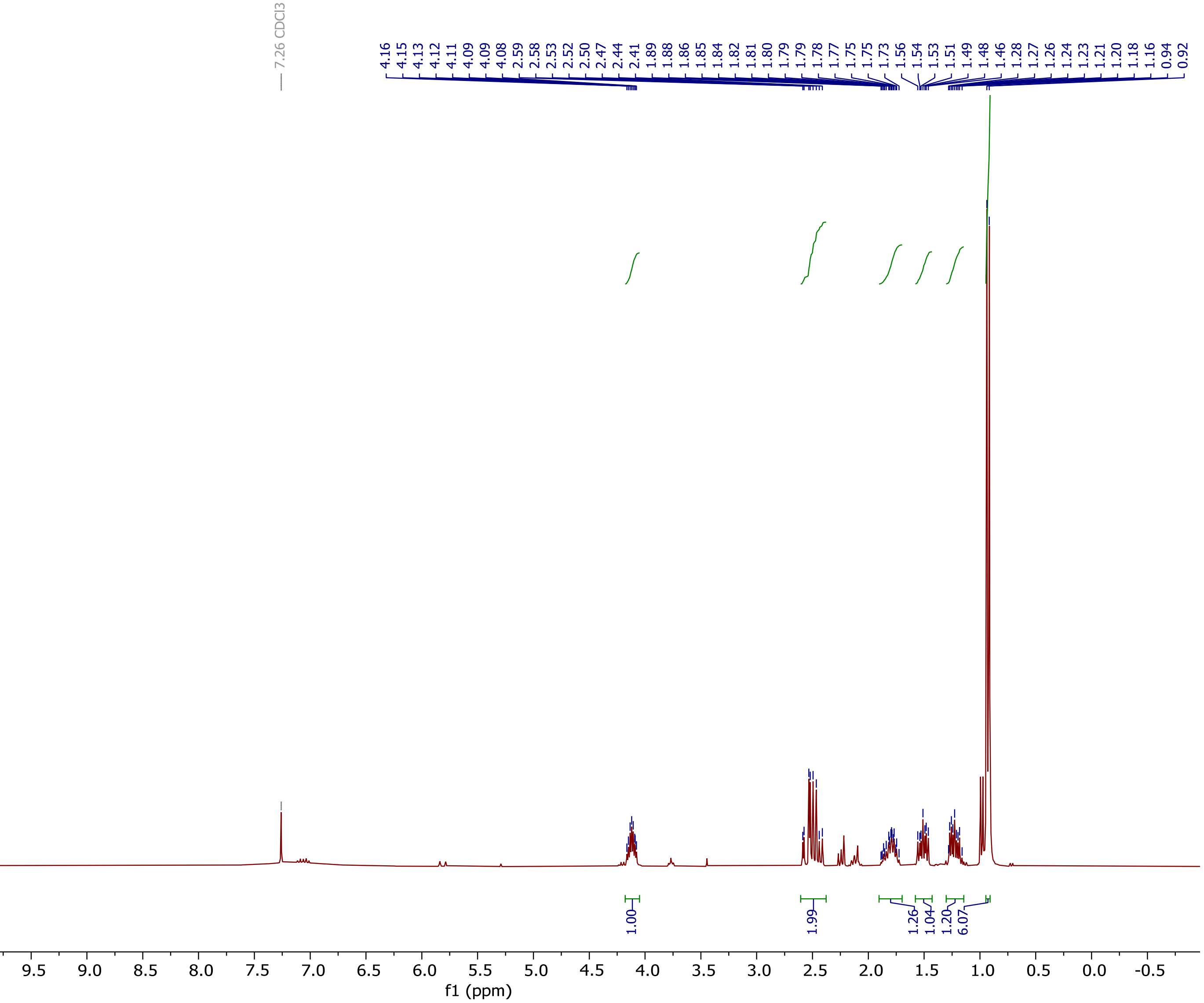

8.80 $^{13}C\{^1H\}$ NMR spectrum of S21b

OJJ-191.2.fid
OJJ-191
13C in CDCl3, 300MHz
19th of May, 2025

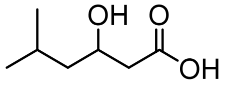

S21b

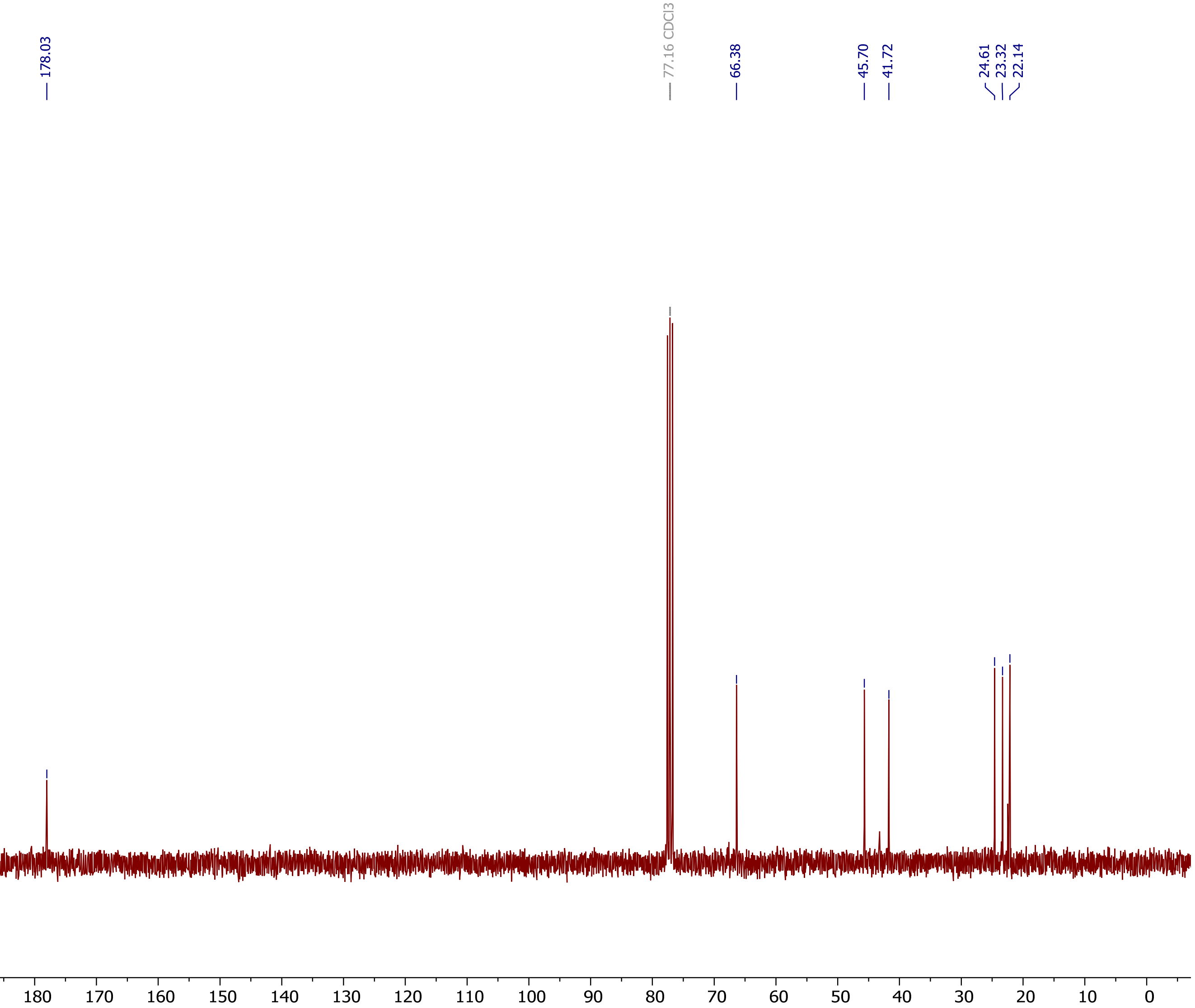

8.81 ^{1}H NMR spectrum of 6'c

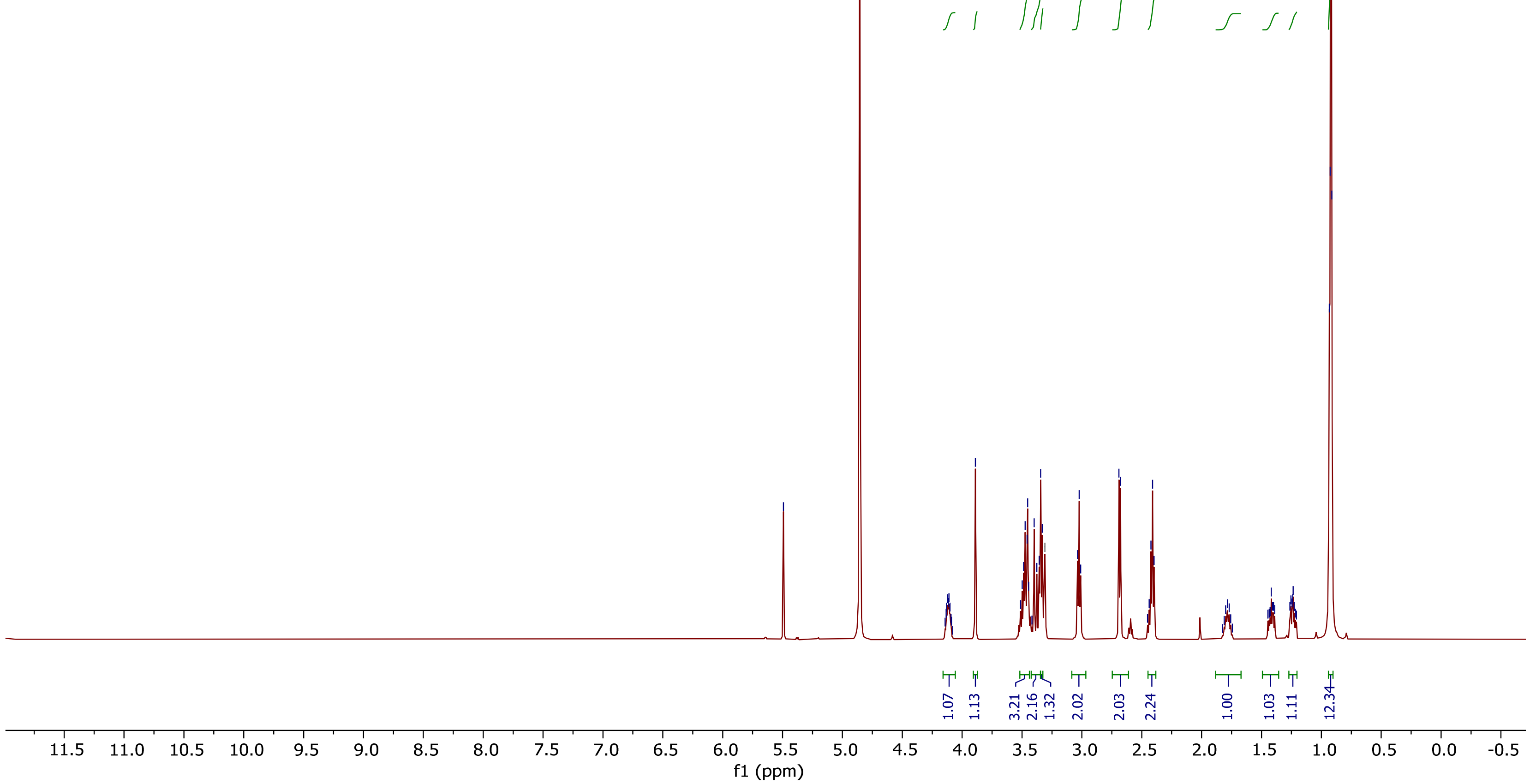

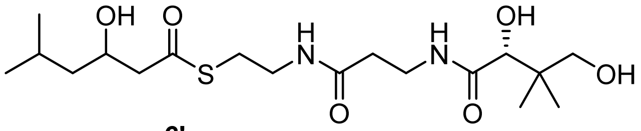

6'c

8.82 $^{13}C\{^{1}H\}$ NMR spectrum of 6'c

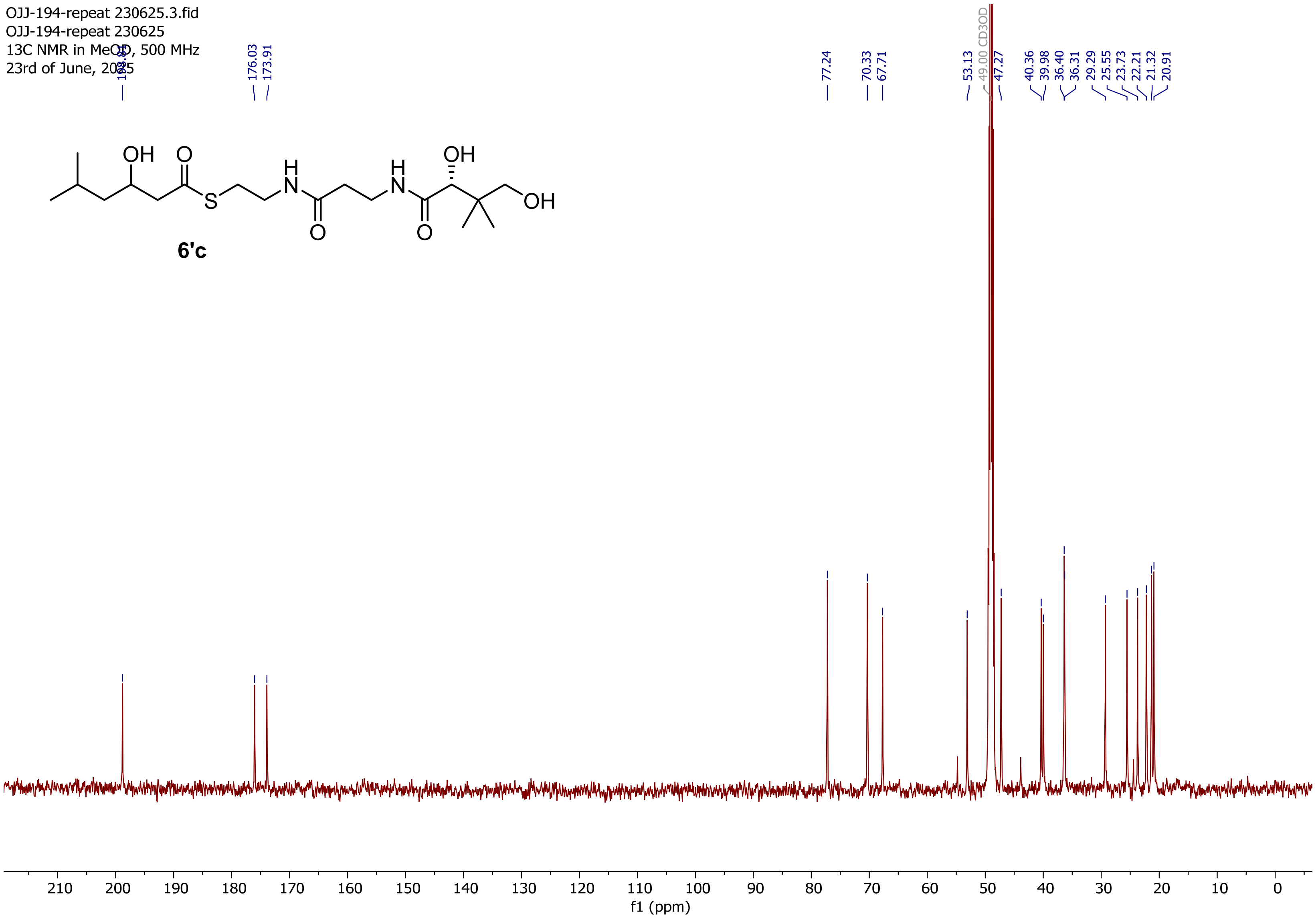

8.83 ^{1}H NMR spectrum of S22b

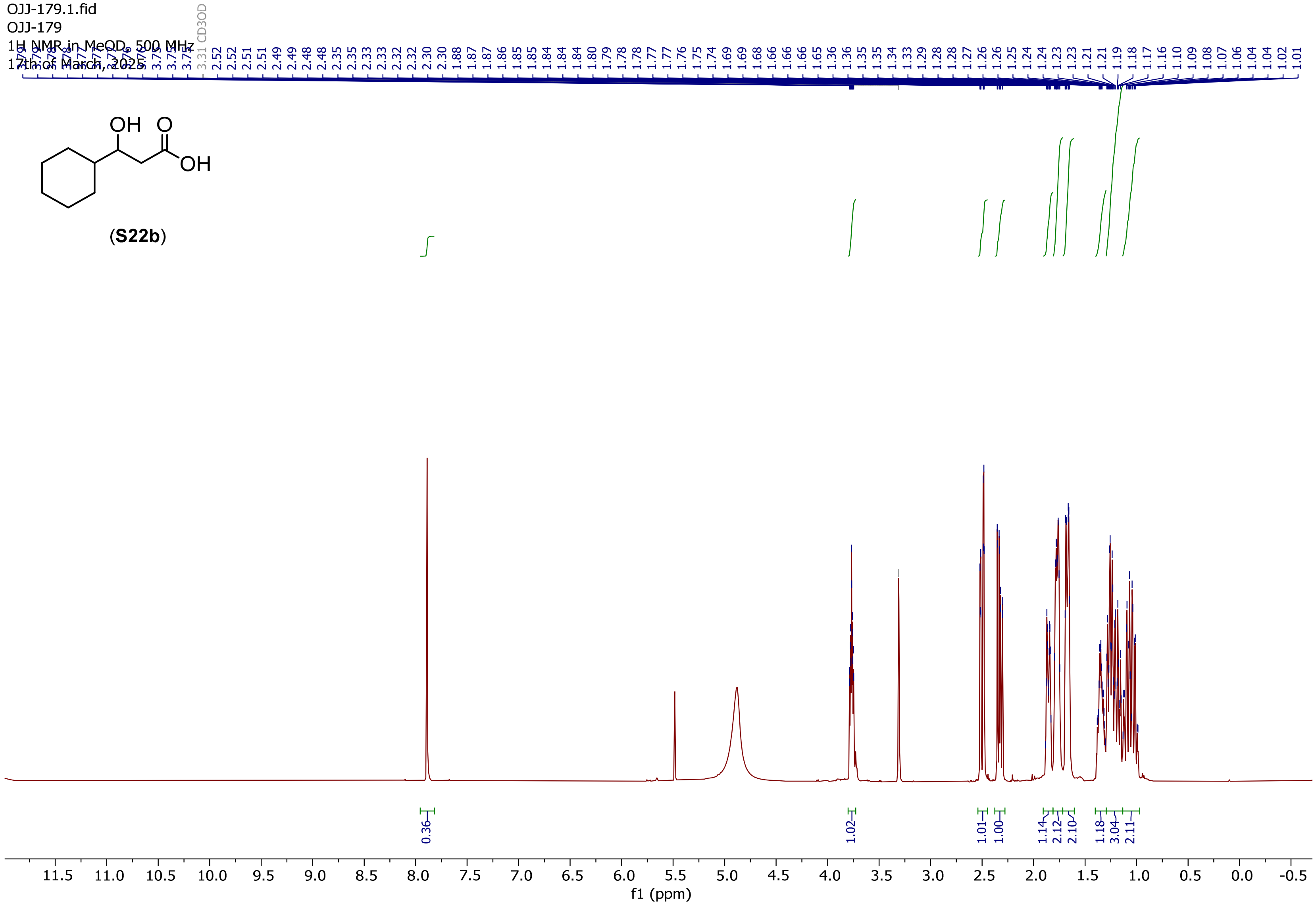

8.84 $^{13}C\{^{1}H\}$ NMR spectrum of S22b

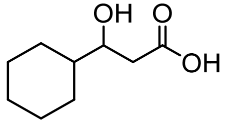

(**S22b**)

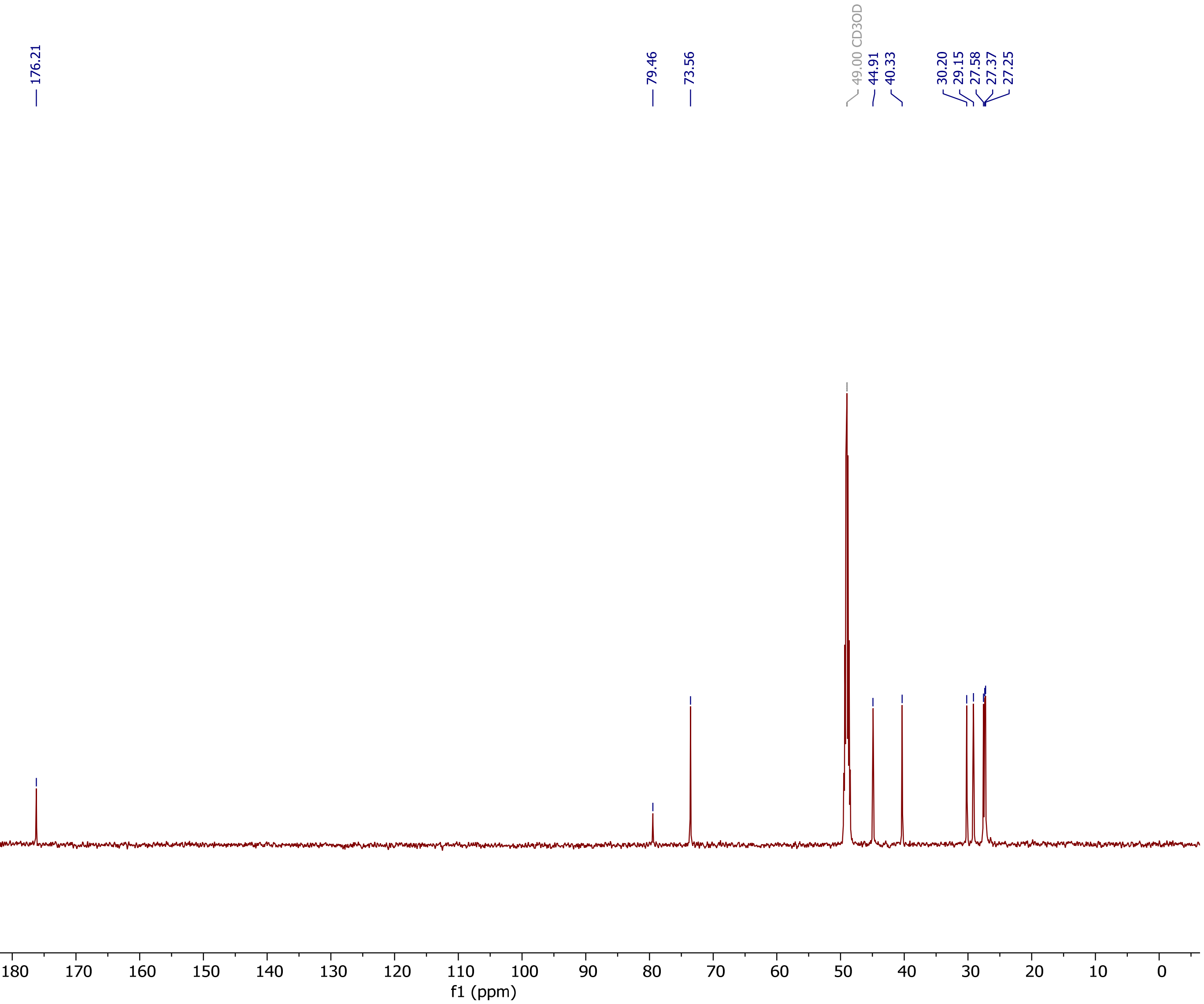

8.85 ^{1}H NMR spectrum of 6’d

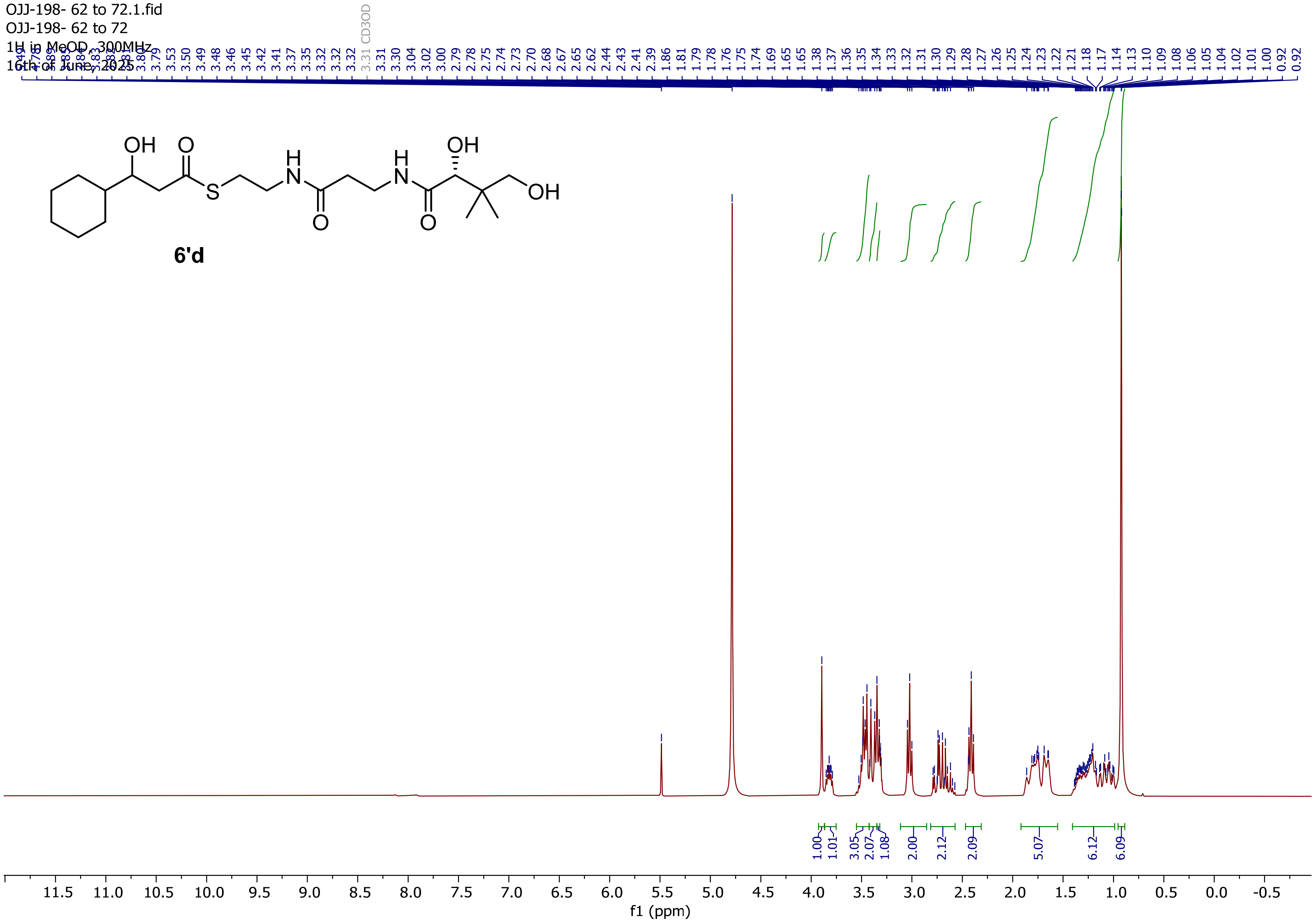

8.86 $^{13}C\{^{1}H\}$ NMR spectrum of 6'd

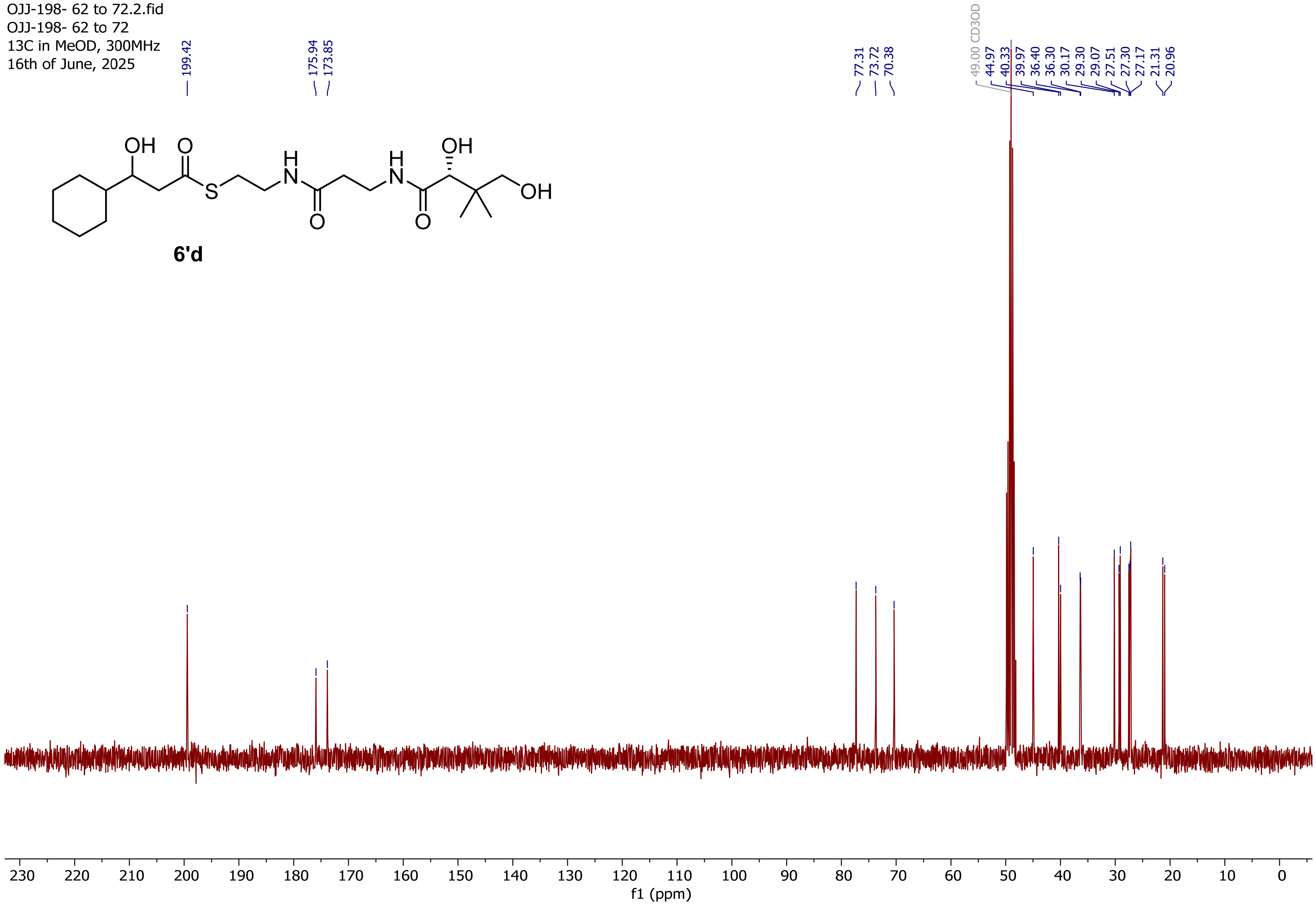

8.87 ¹H NMR spectrum of S23b

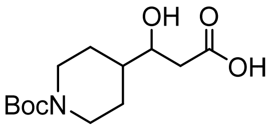

(**S23b**)

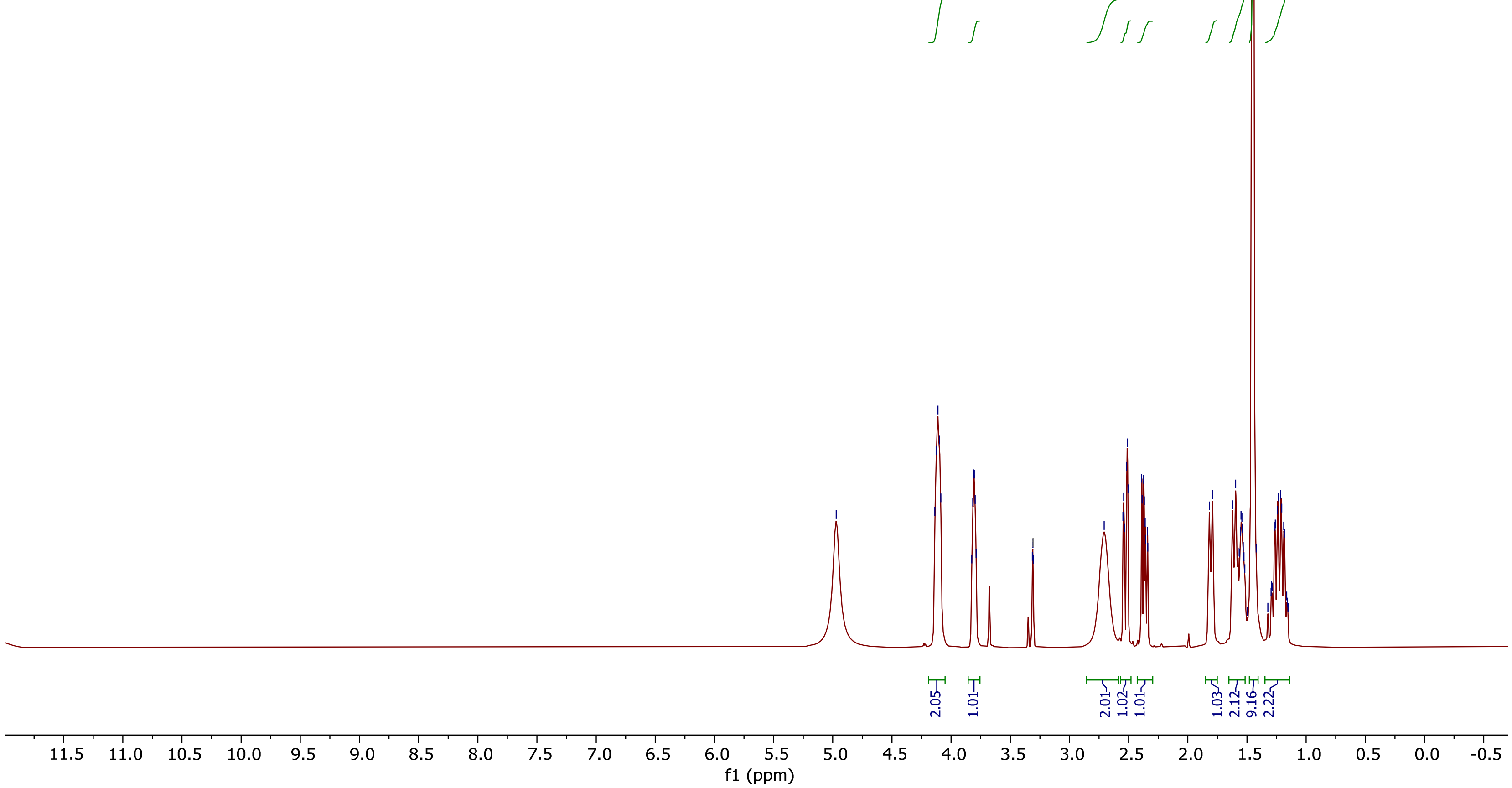

8.88 $^{13}C\{^{1}H\}$ NMR spectrum of S23b

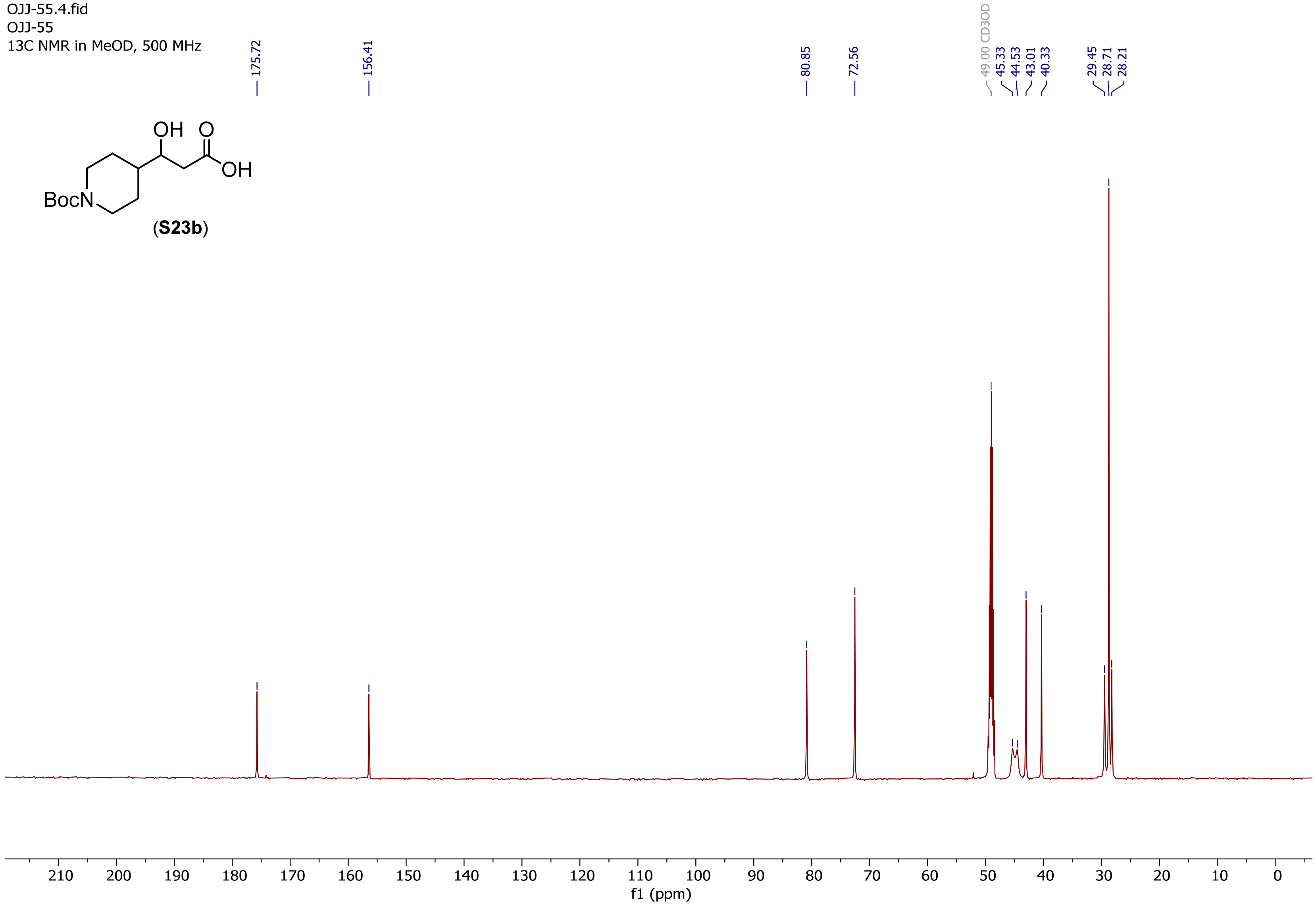

8.89 ^{1}H NMR spectrum of 6'e

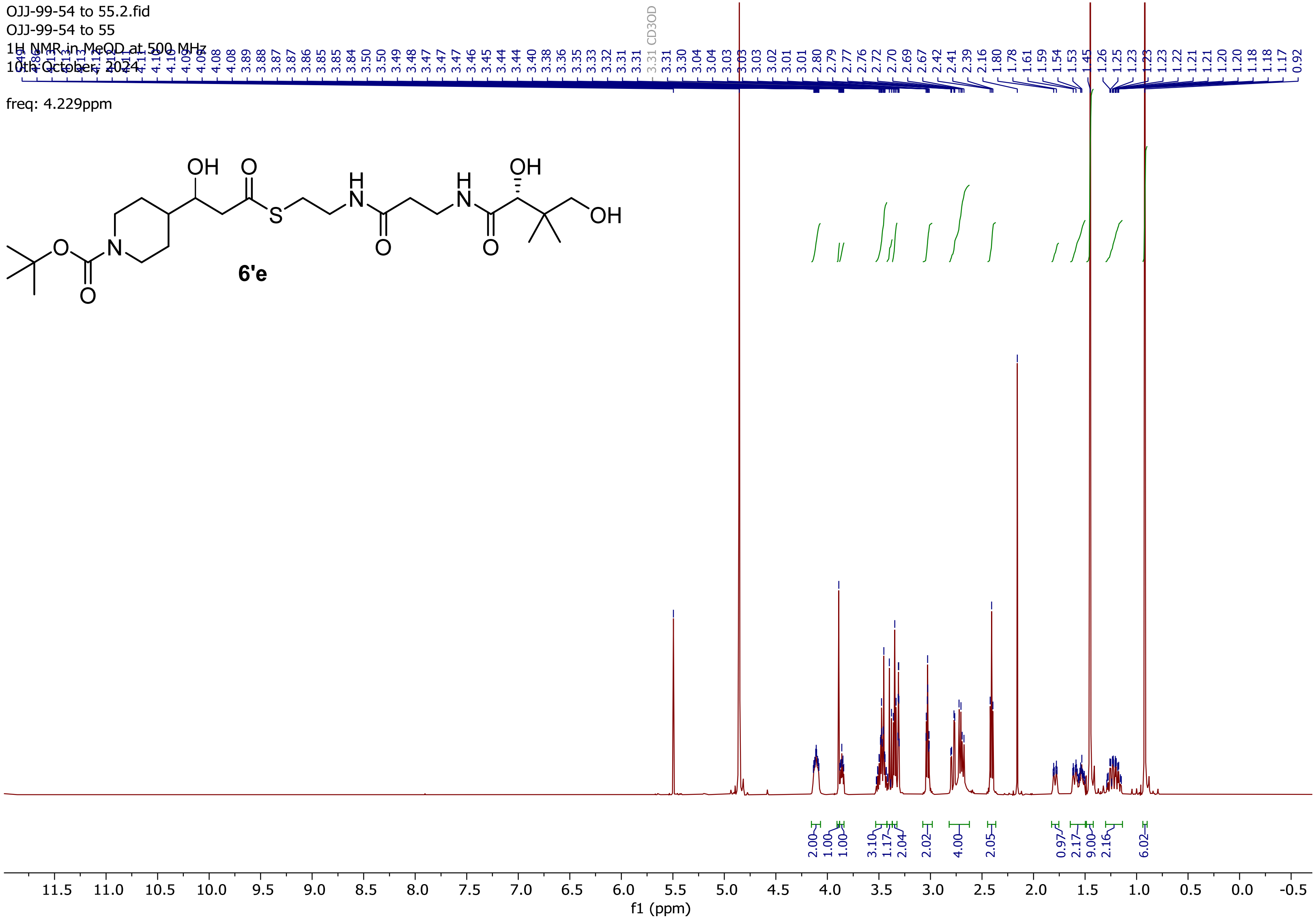

8.90 $^{13}C\{^{1}H\}$ NMR spectrum of 6’e

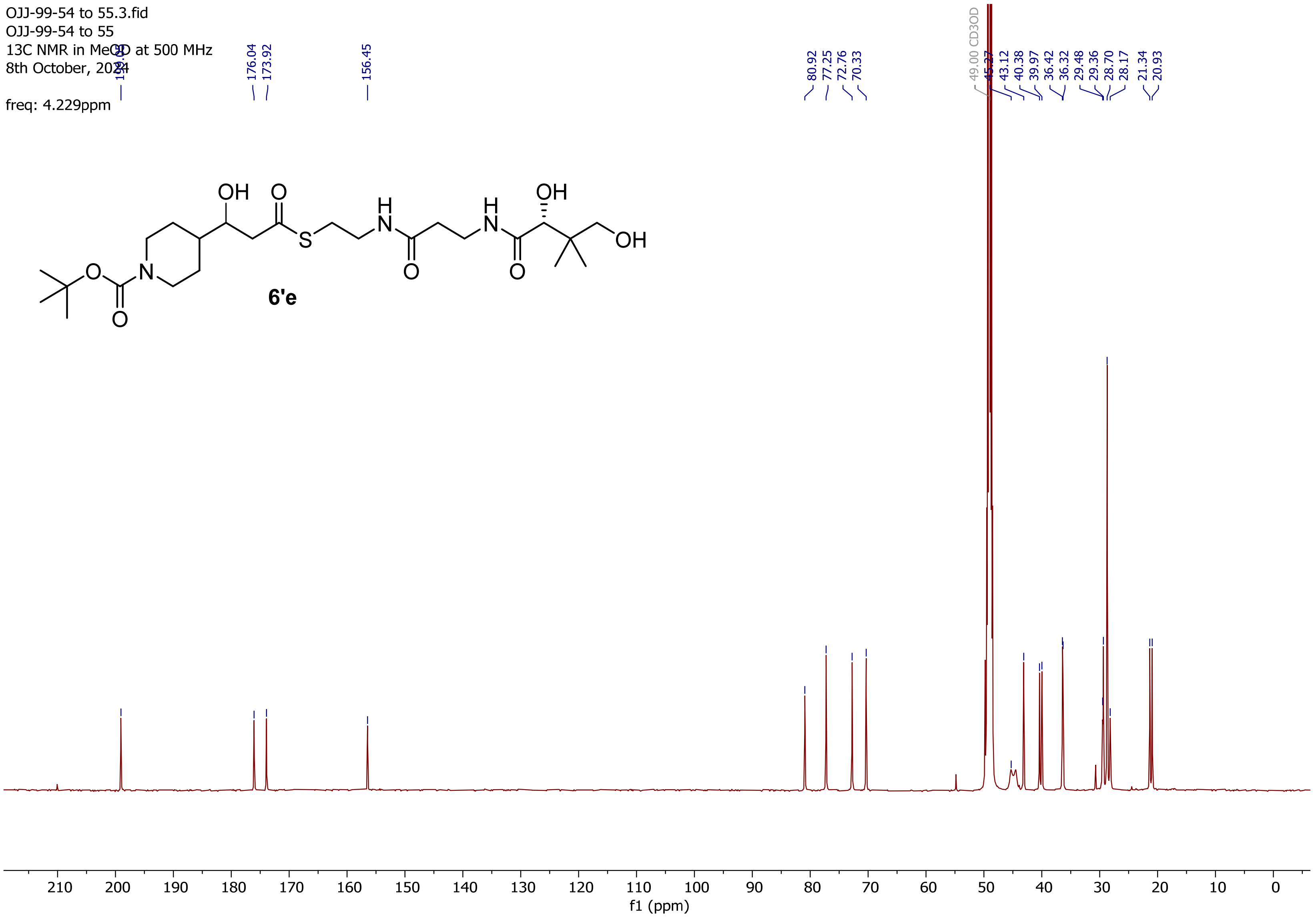

8.91 ^{1}H NMR spectrum of S25b

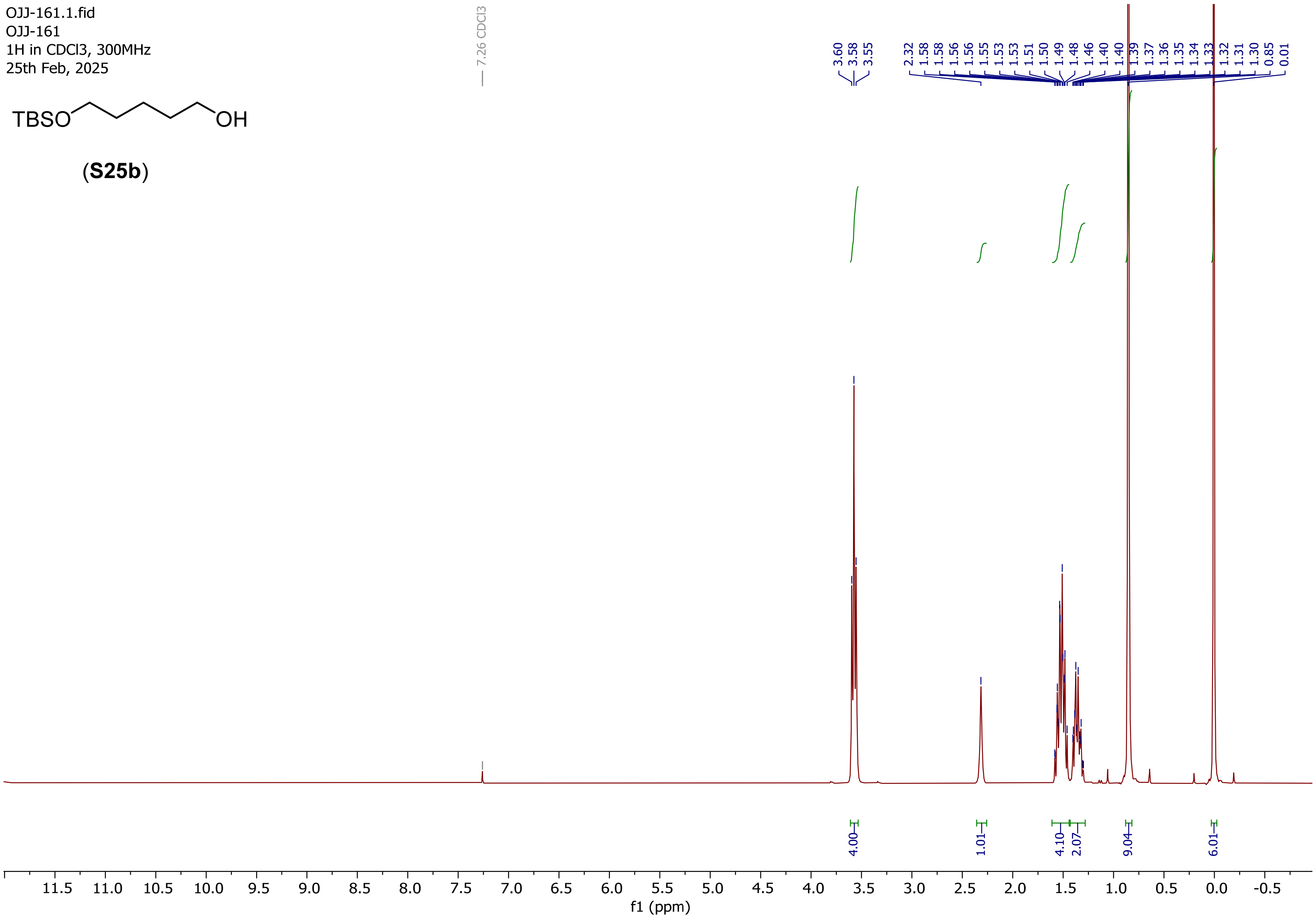

8.92 $^{13}C\{^1H\}$ NMR spectrum of S25b

OJJ-161.2.fid
OJJ-161
13C in CDCl3, 300MHz
25th Feb, 2025

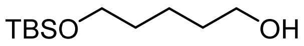

(**S25b**)

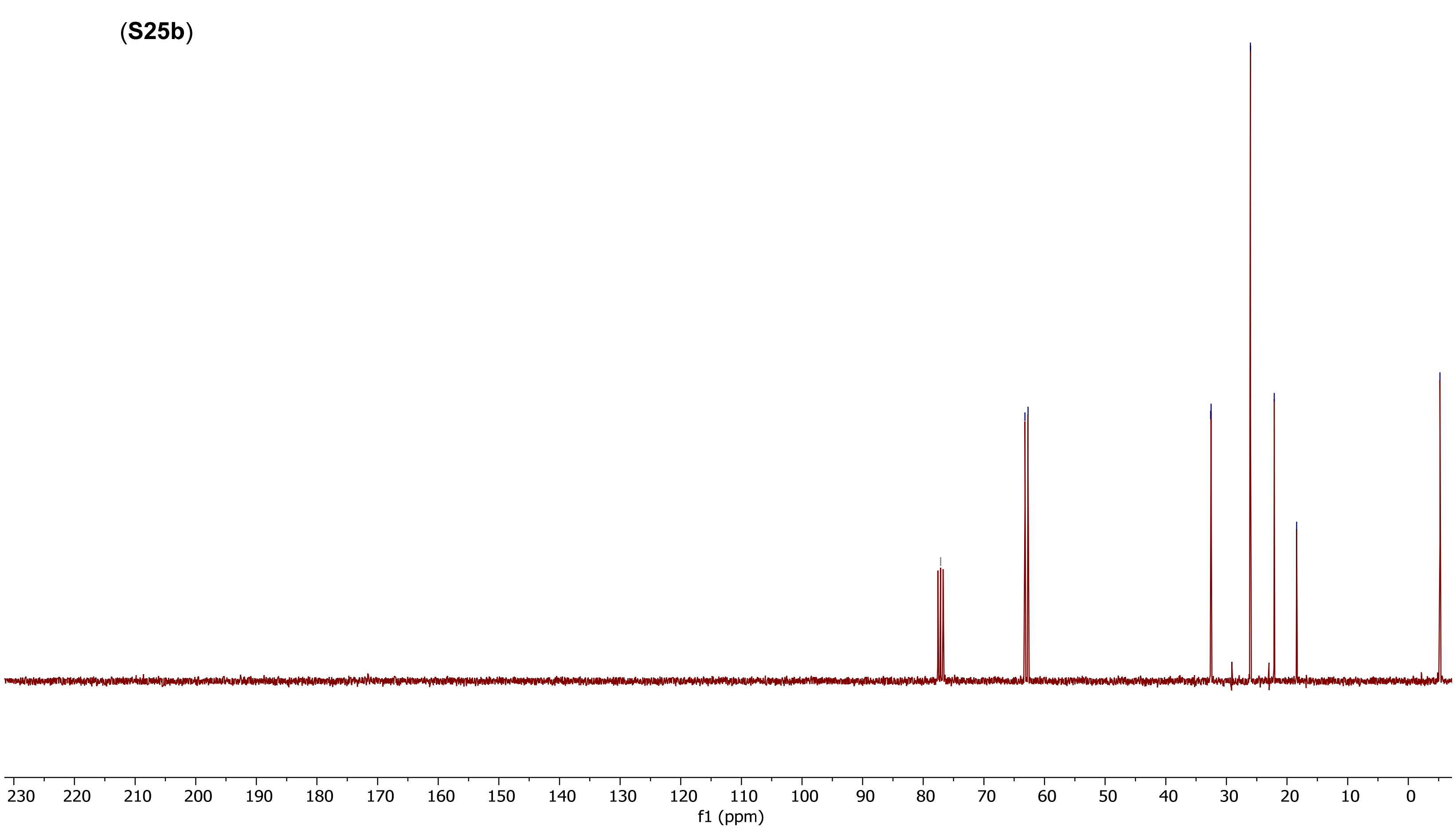

8.93 ^{1}H NMR spectrum of S25c

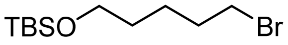

(**S25c**)

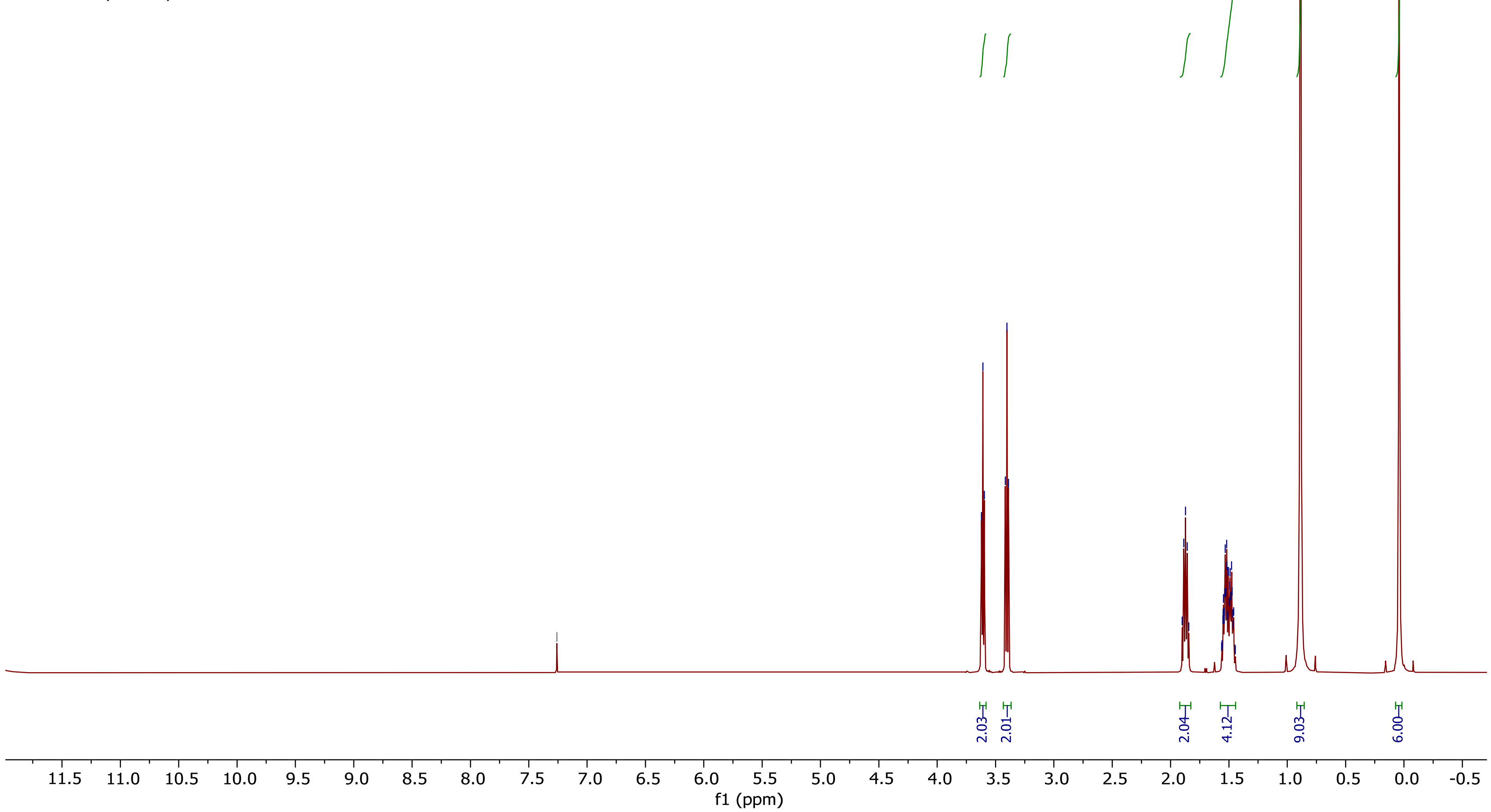

8.94 $^{13}C\{^{1}H\}$ NMR spectrum of S25c

OJJ-166.2.fid
OJJ-166
13C NMR in CDCl3, 500 MHz
4th March, 2025

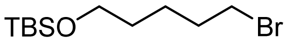

(**S25c**)

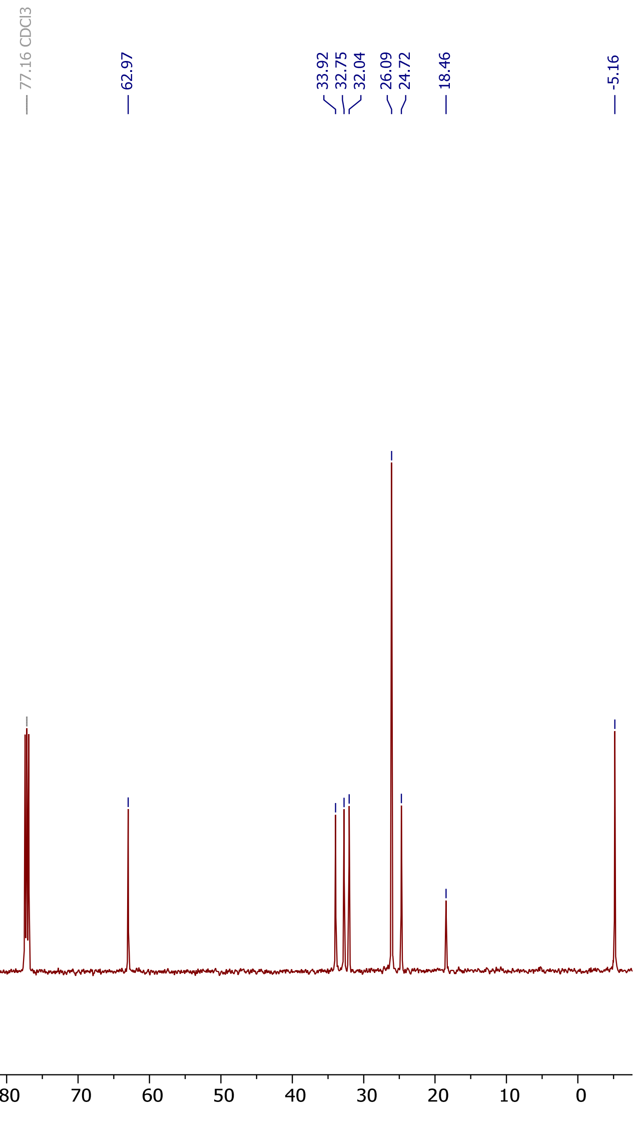

8.95 ^{1}H NMR spectrum of S25d

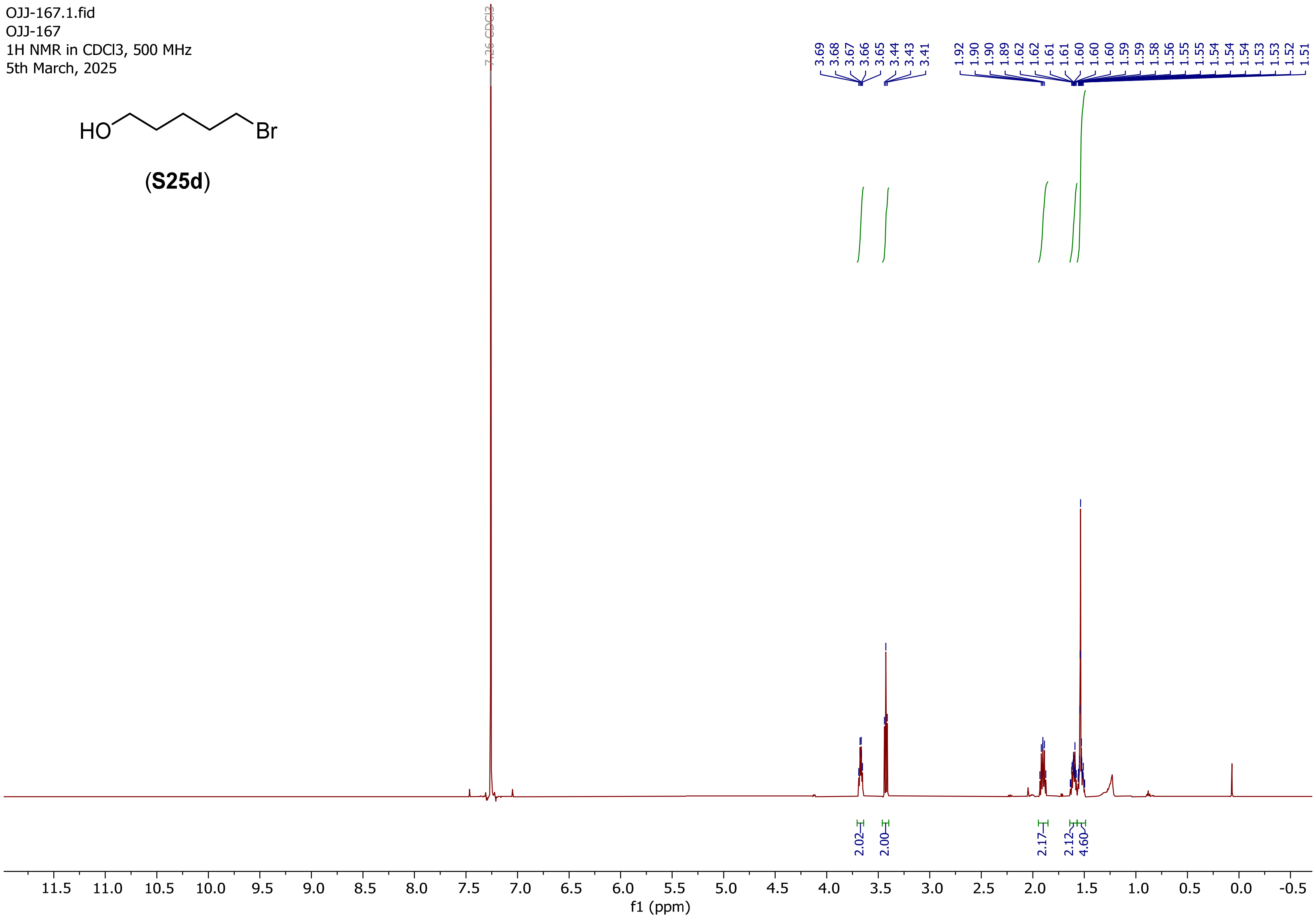

8.96 $^{13}C\{^{1}H\}$ NMR spectrum of S25d

OJJ-184.2.fid
OJJ-184
13C in CDCl3, 300MHz
5th of May, 2025

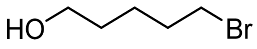

(**S25d**)

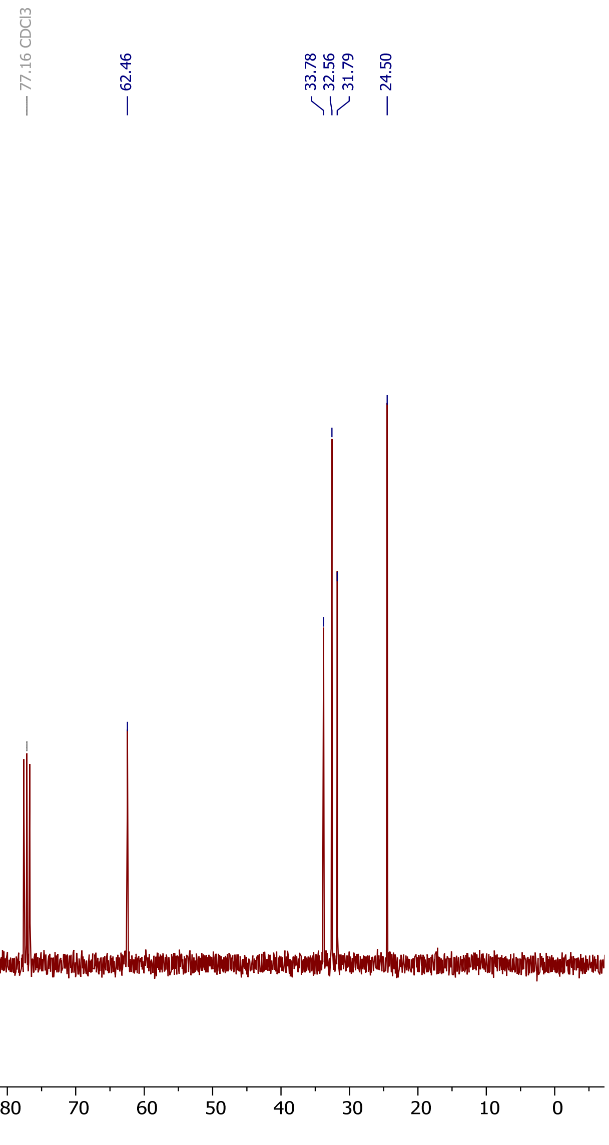

8.97 ^{1}H NMR spectrum of S24a

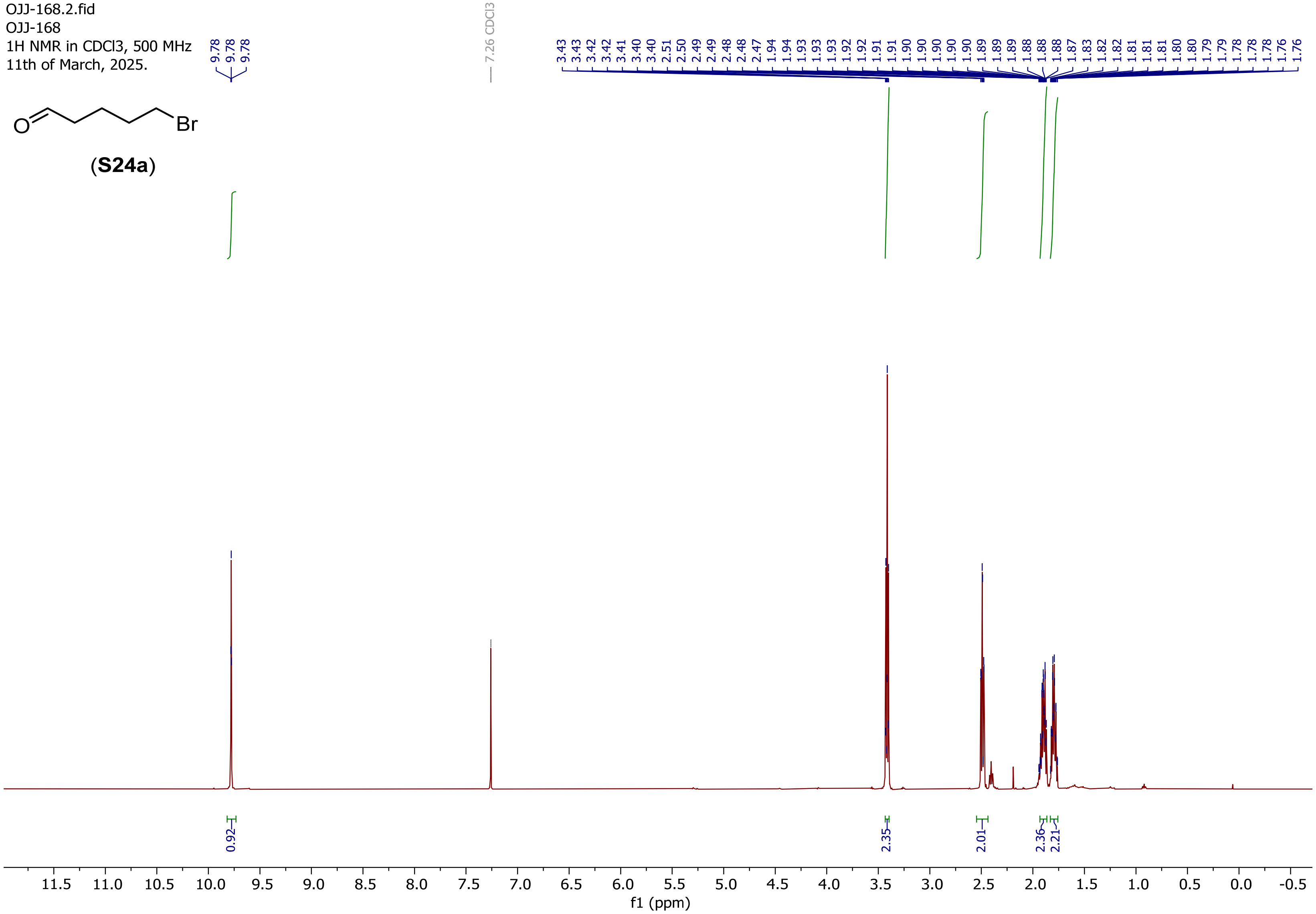

8.98 $^{13}C\{^{1}H\}$ NMR spectrum of S24a

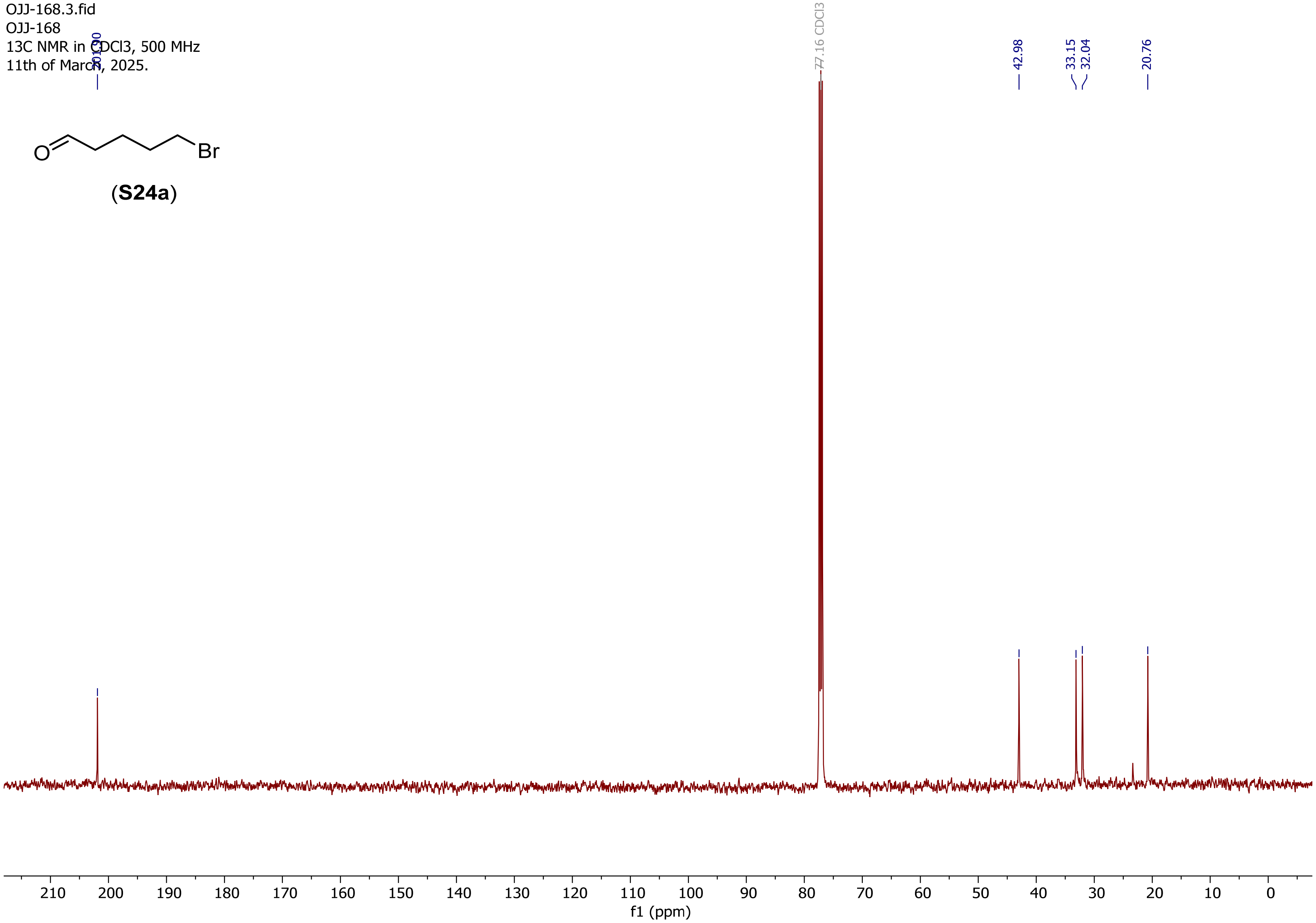

8.99 ^{1}H NMR spectrum of S24b

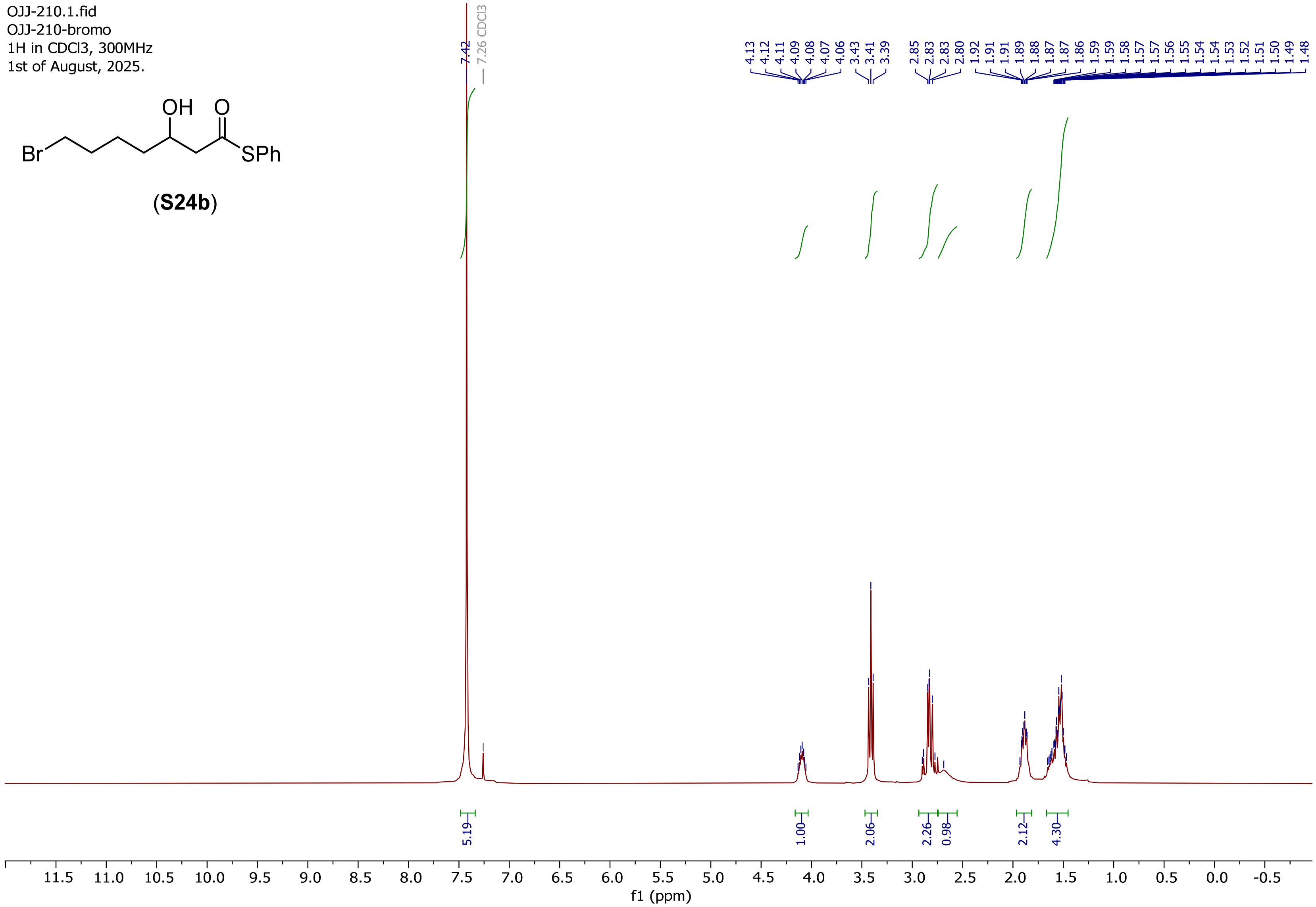

8.100 $^{13}C\{^{1}H\}$ NMR spectrum of S24b

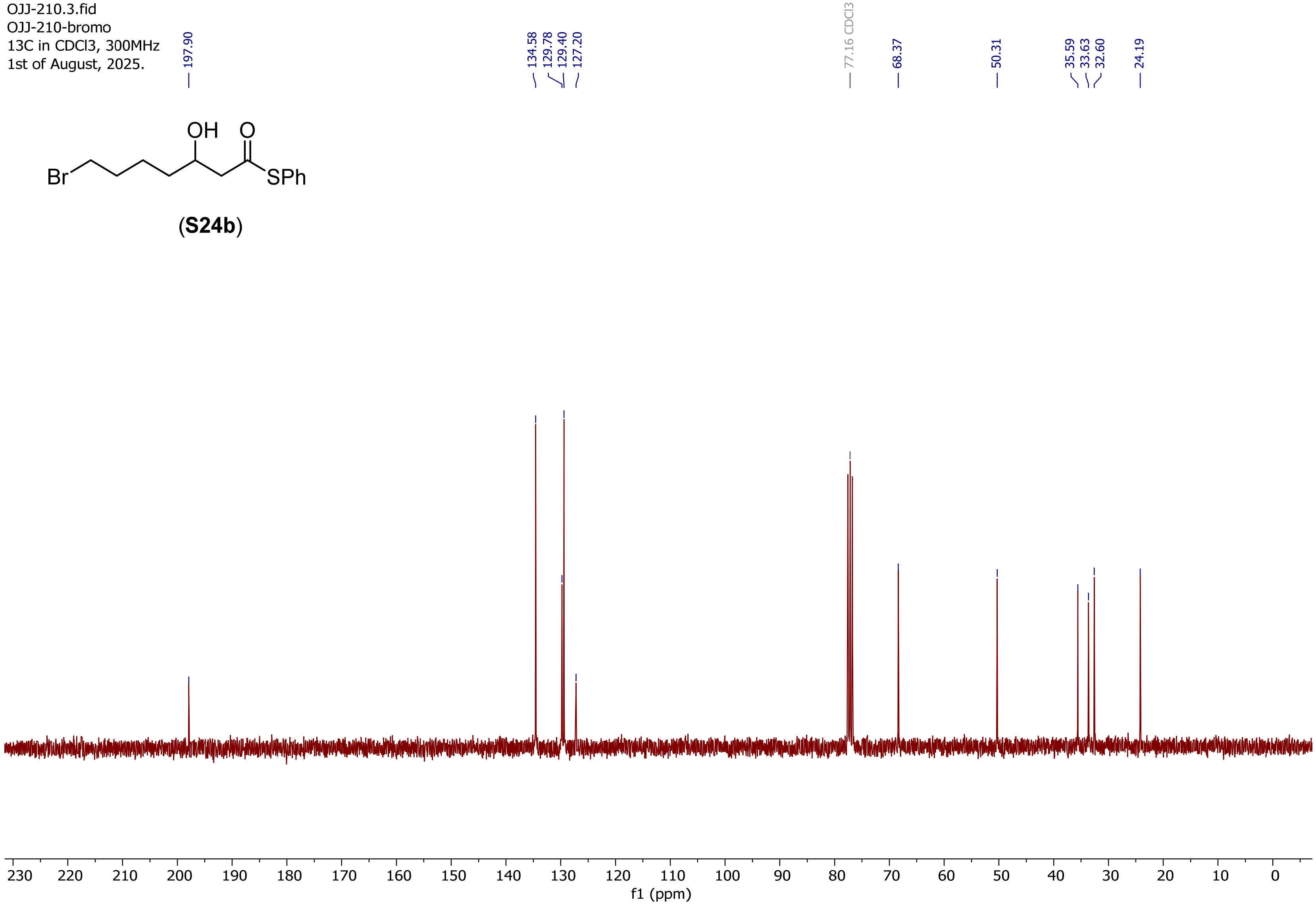

8.101 ^{1}H NMR spectrum of 6’f

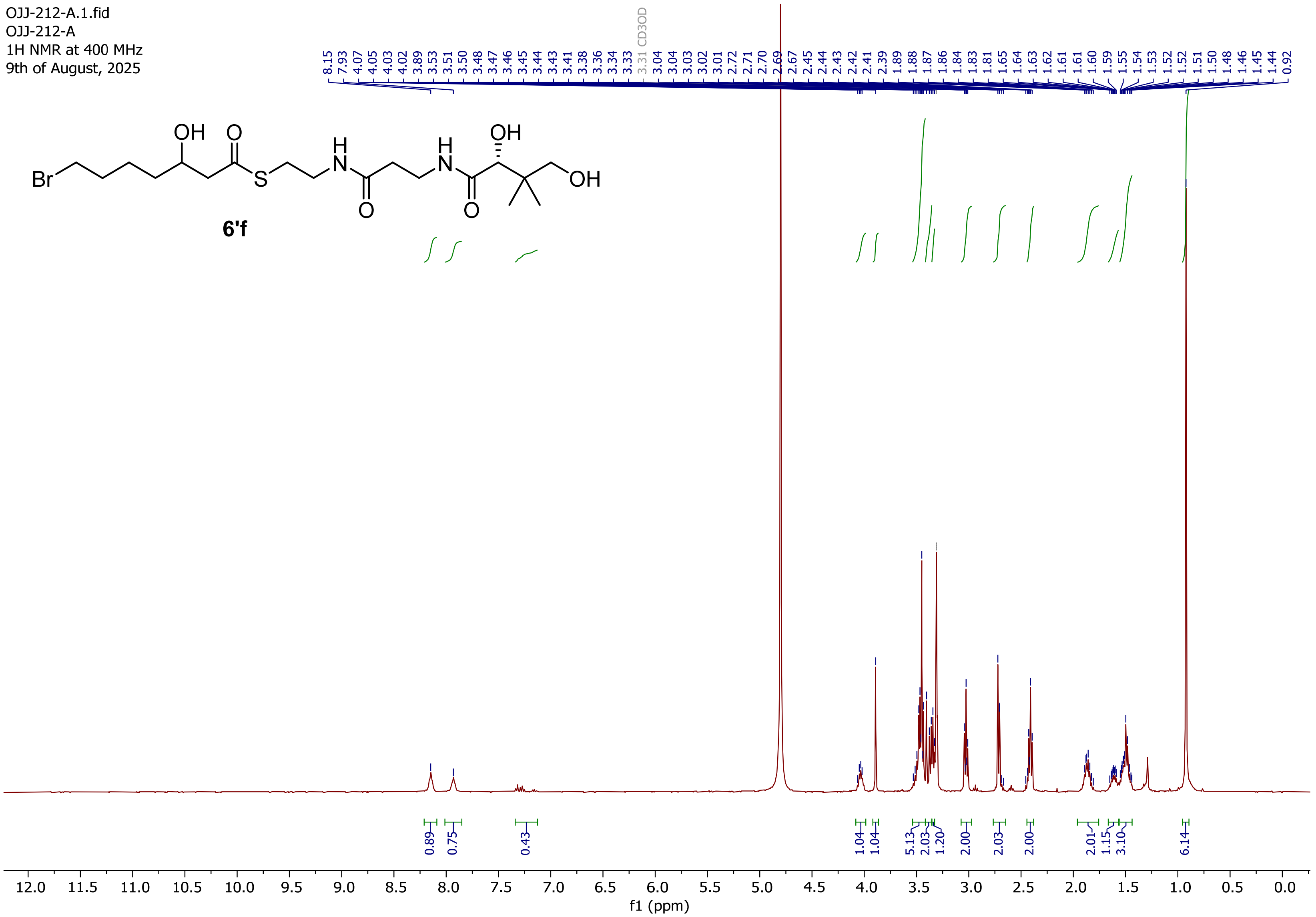

8.102 $^{13}C\{^{1}H\}$ NMR spectrum of 6’f

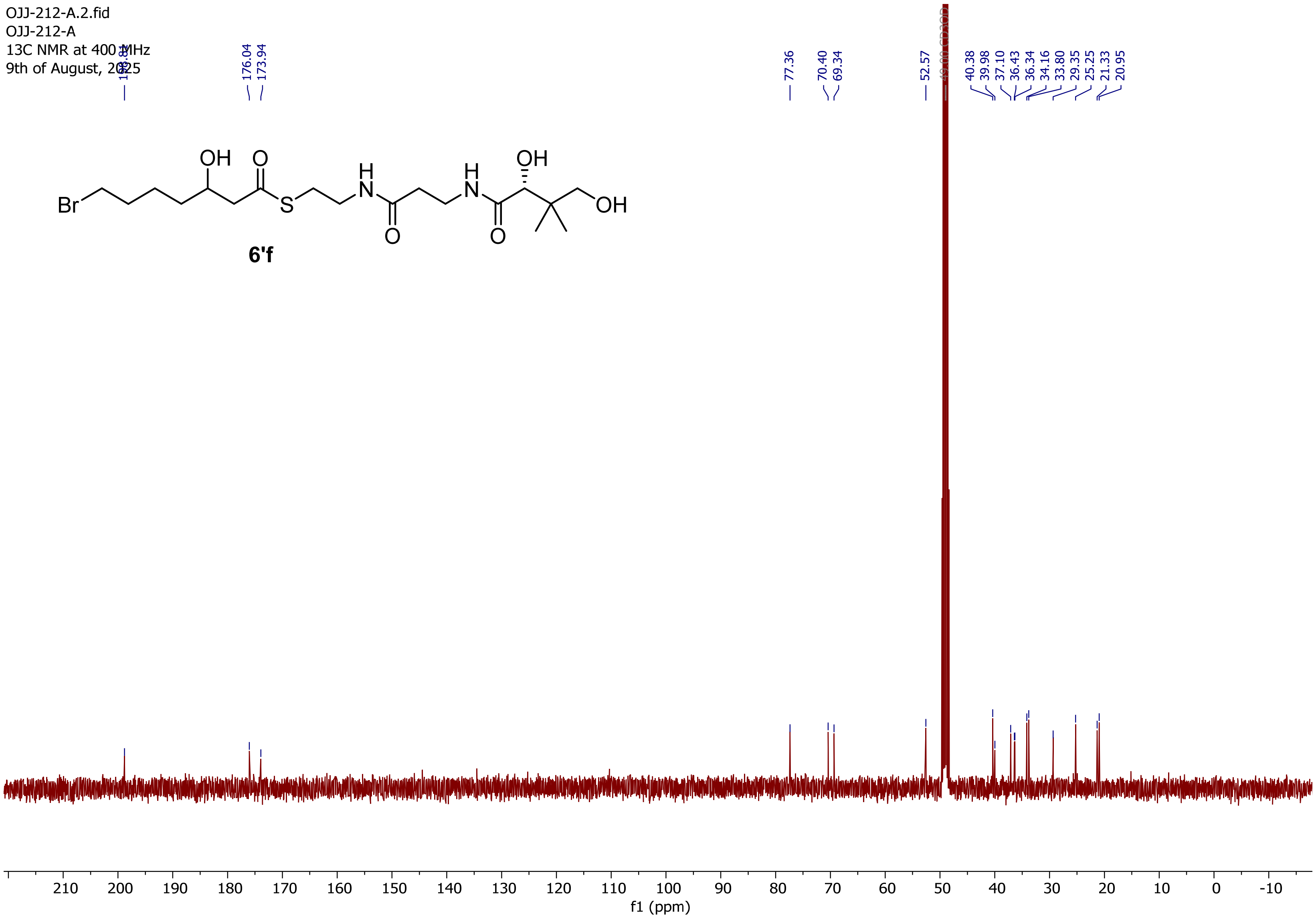

8.103 ^{1}H NMR spectrum of S27b

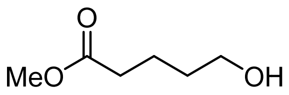

(**S27b**)

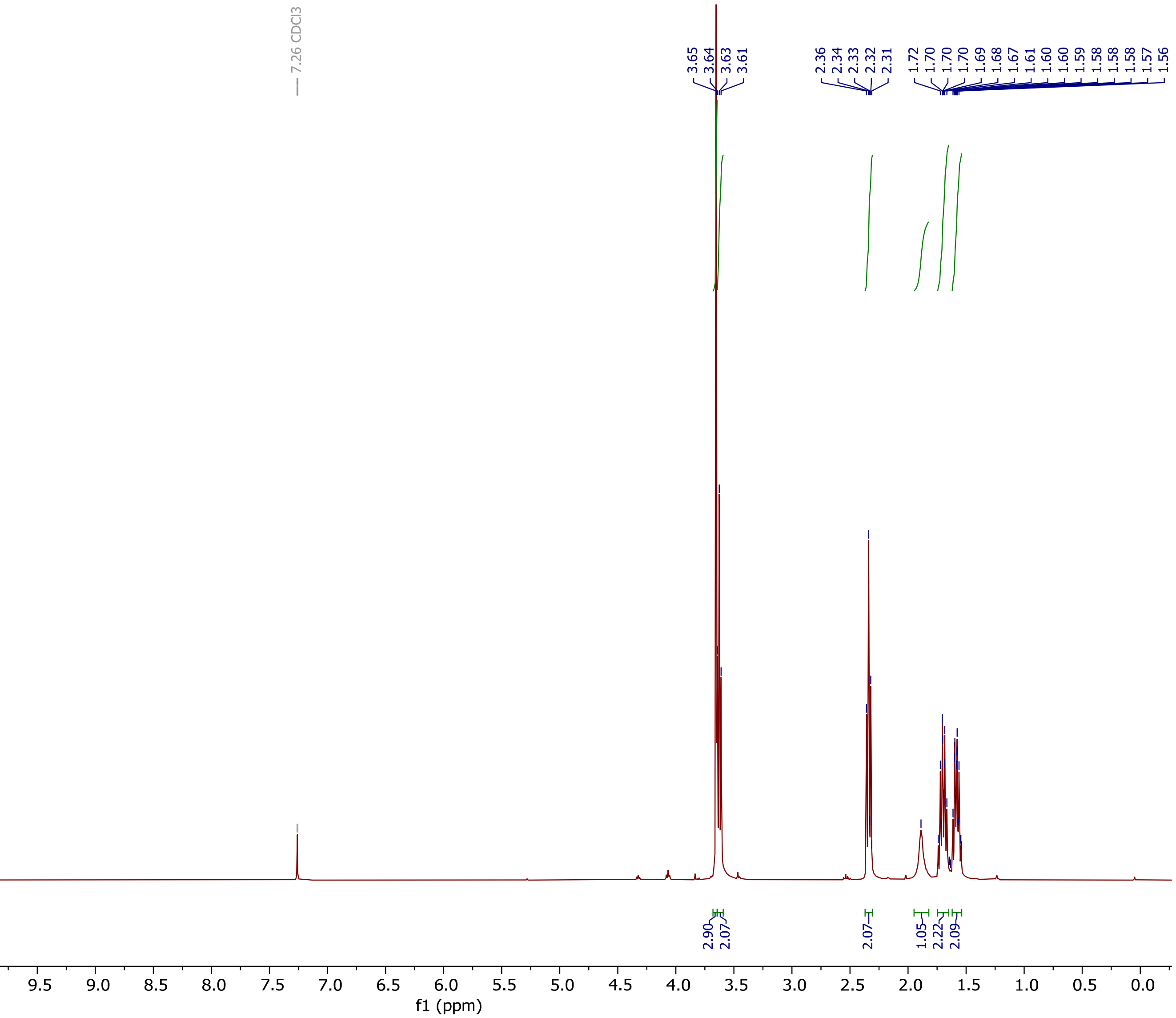

8.104 $^{13}C\{^1H\}$ NMR spectrum of S27b

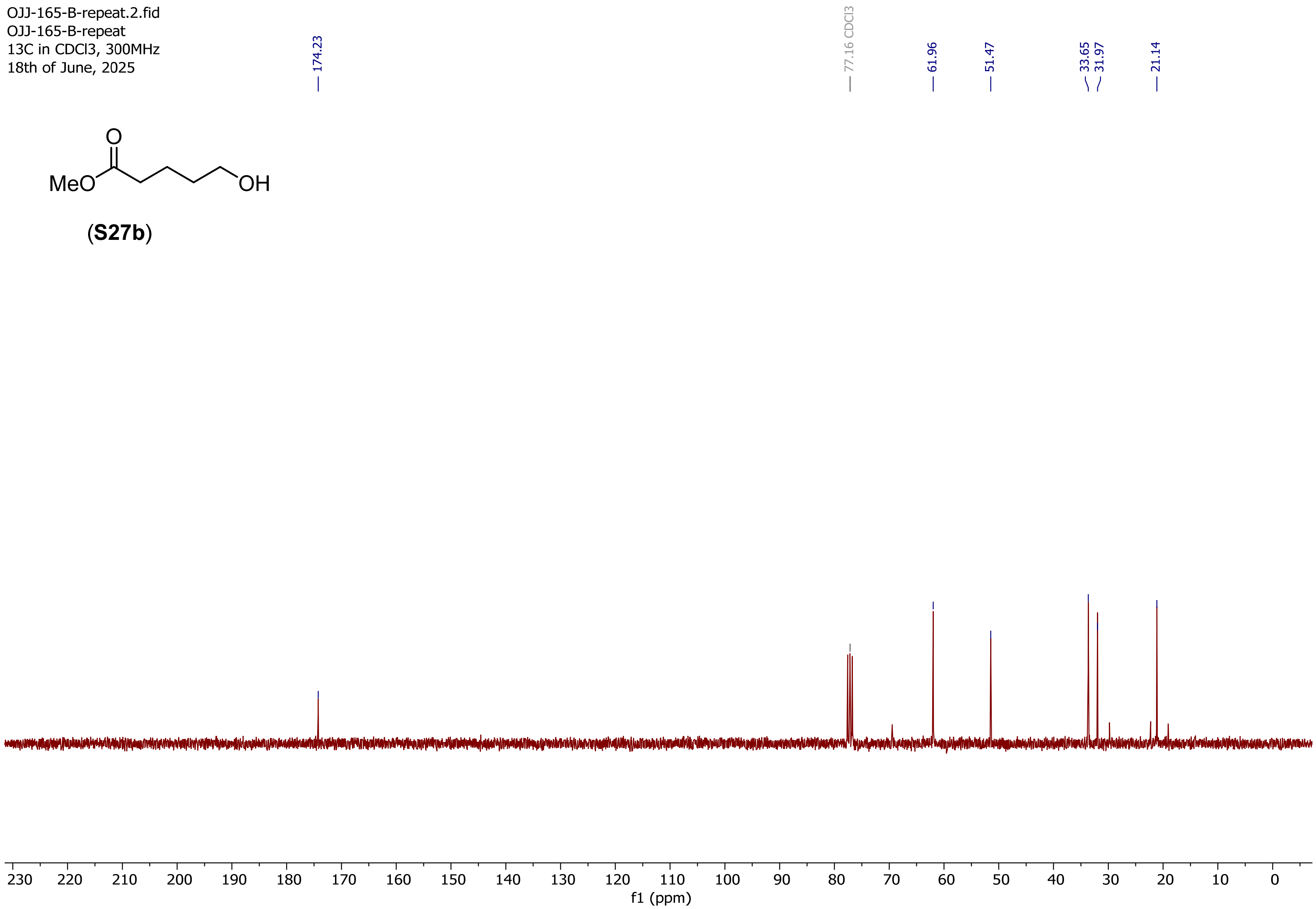

8.105 ^{1}H NMR spectrum of S27c

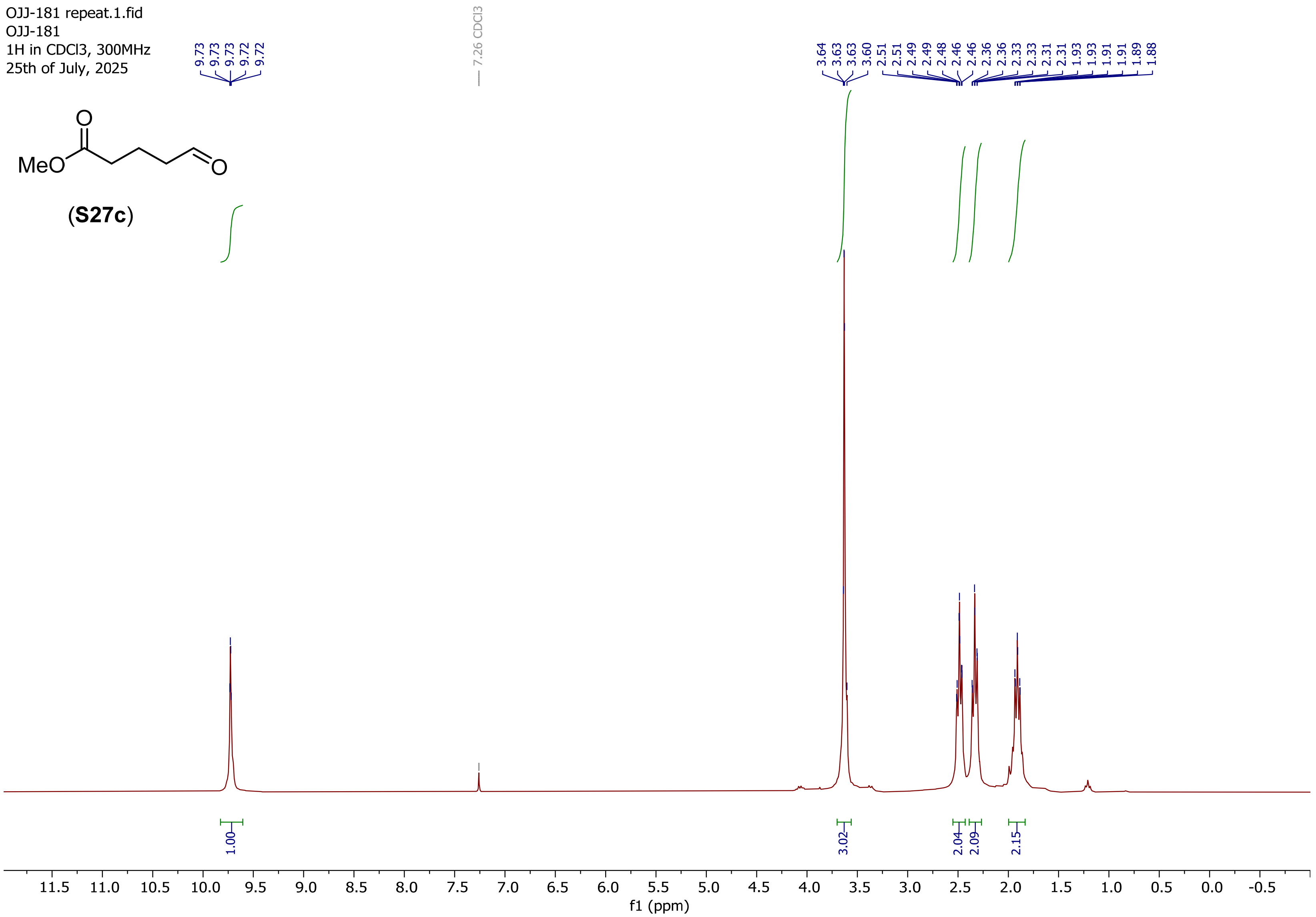

8.106 $^{13}C\{^{1}H\}$ NMR spectrum of S27c

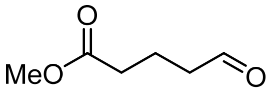

(**S27c**)

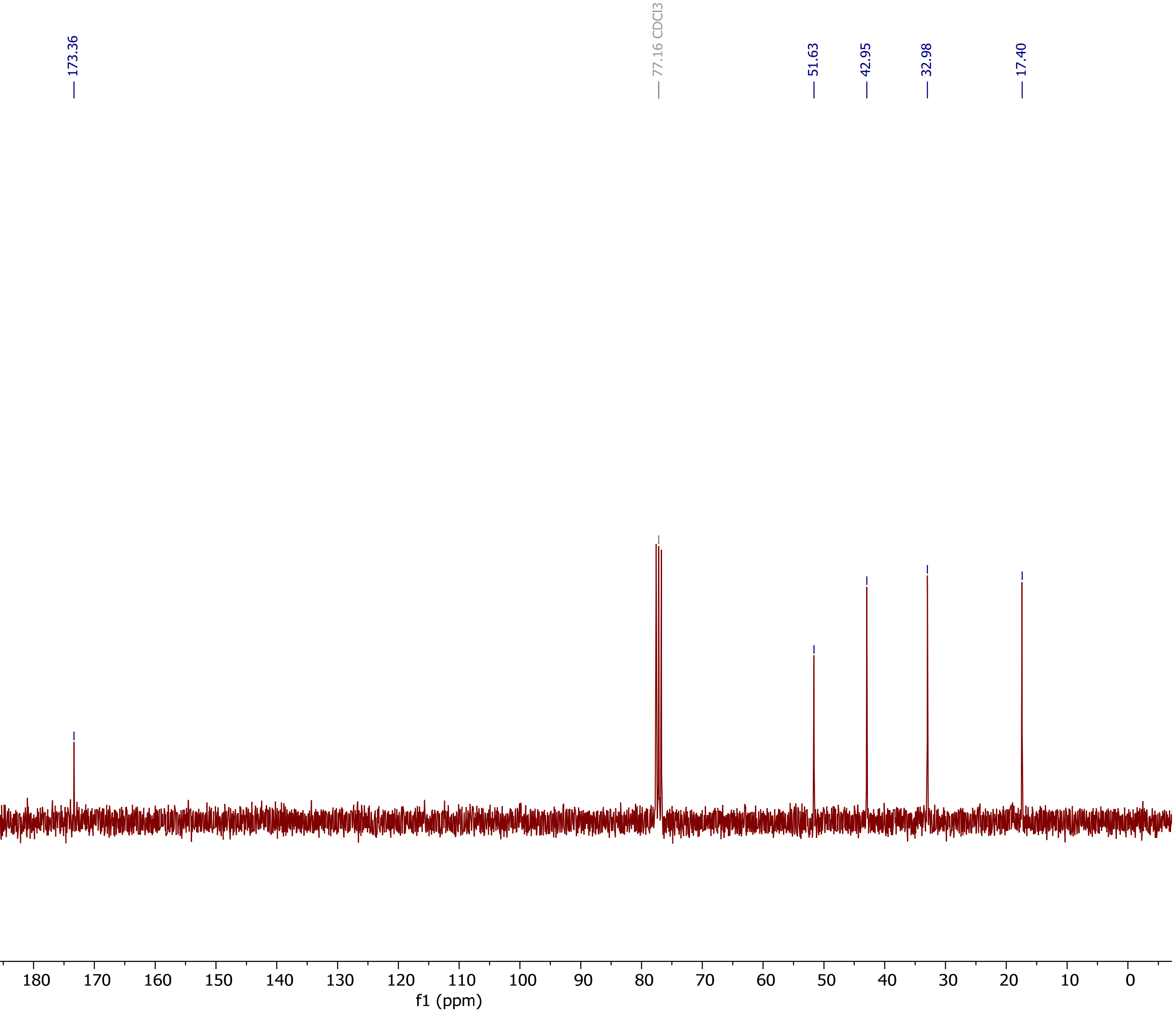

8.107 ^{1}H NMR spectrum of S27d

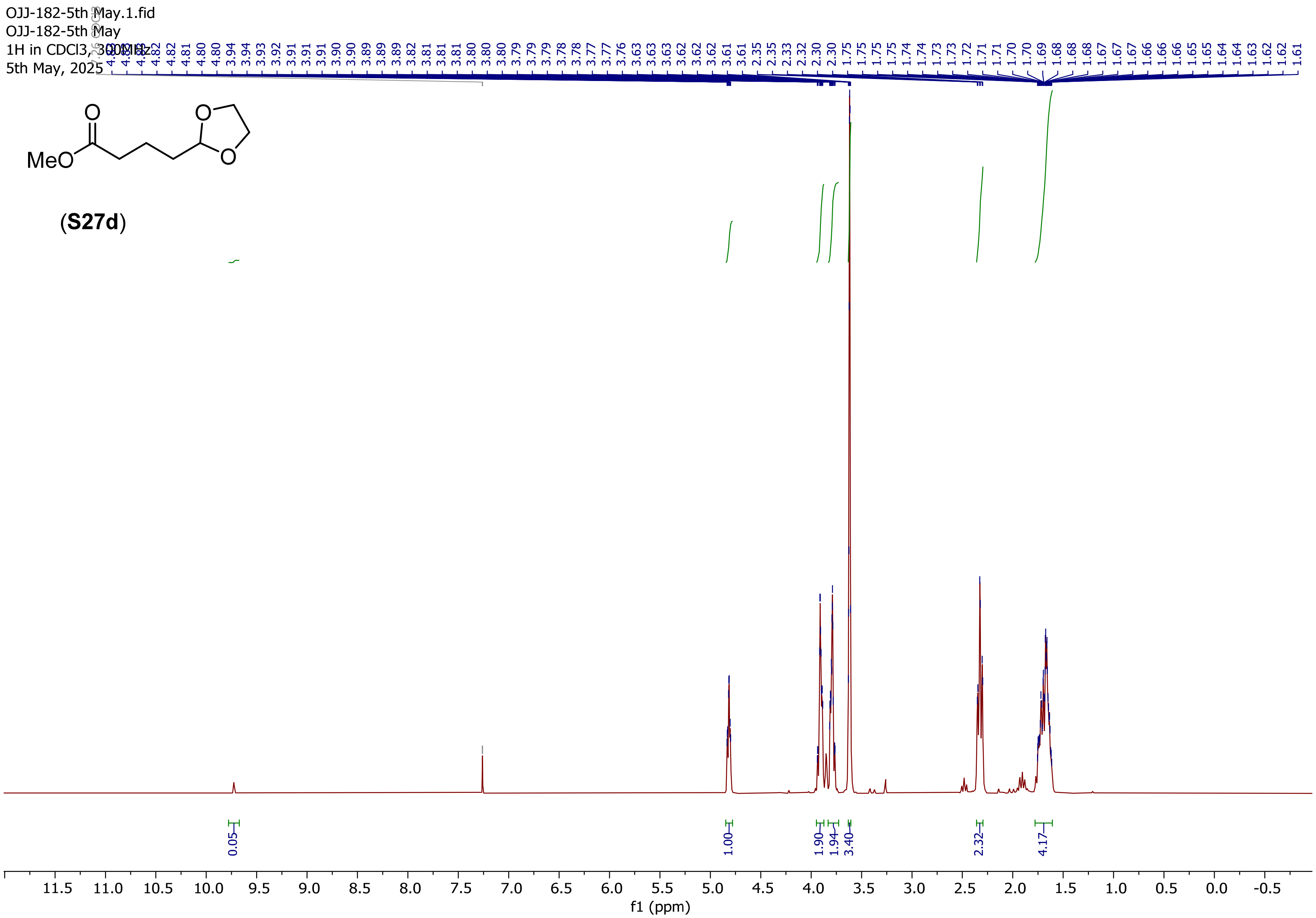

8.108 $^{13}C\{^{1}H\}$ NMR spectrum of S27d

OJJ-182-5th May.2.fid
OJJ-182-5th May
13C in CDCl3, 300MHz
5th May, 2025

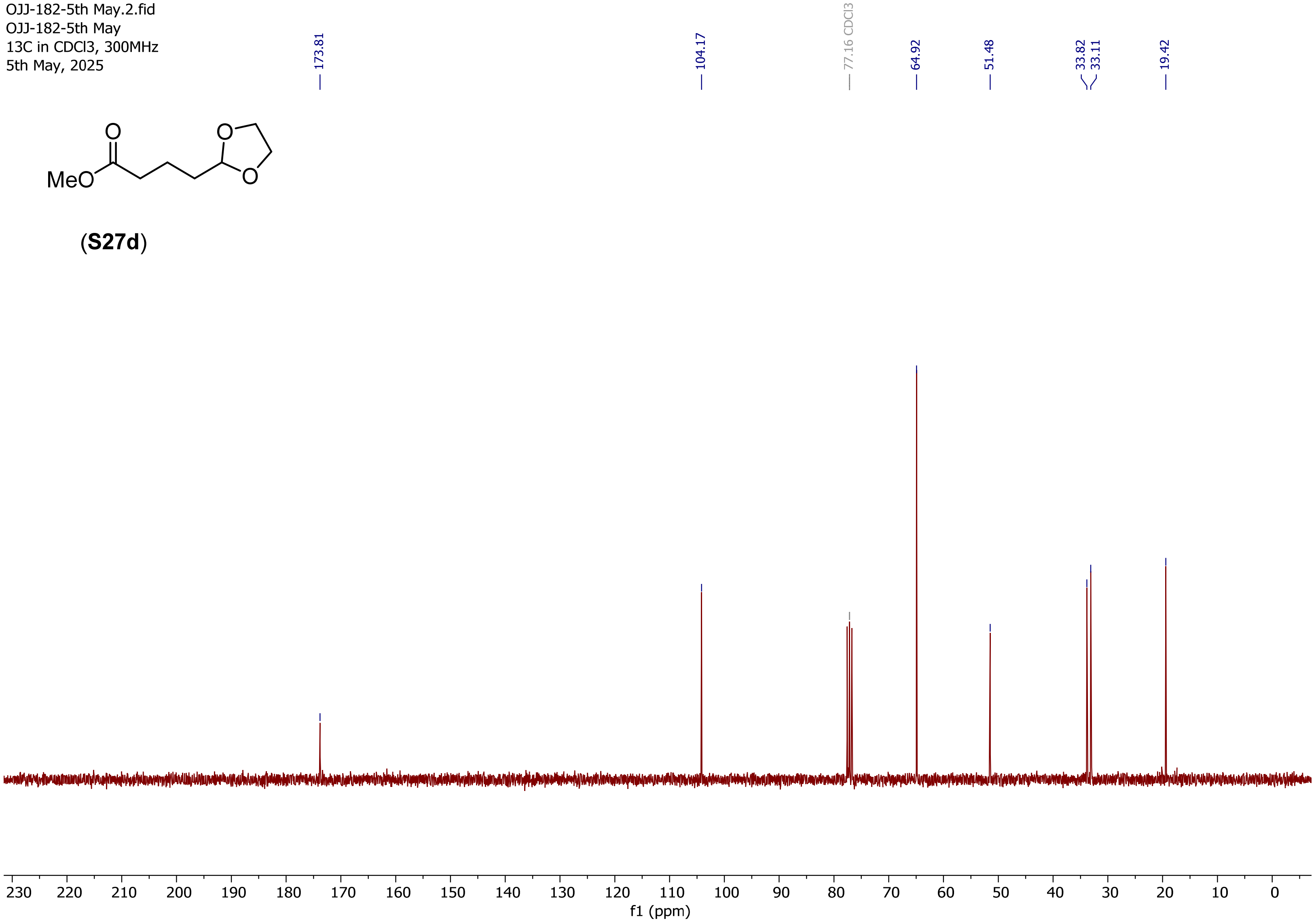

8.109 ¹H NMR spectrum of S27e

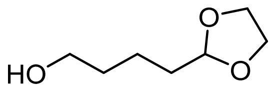

(**S27e**)

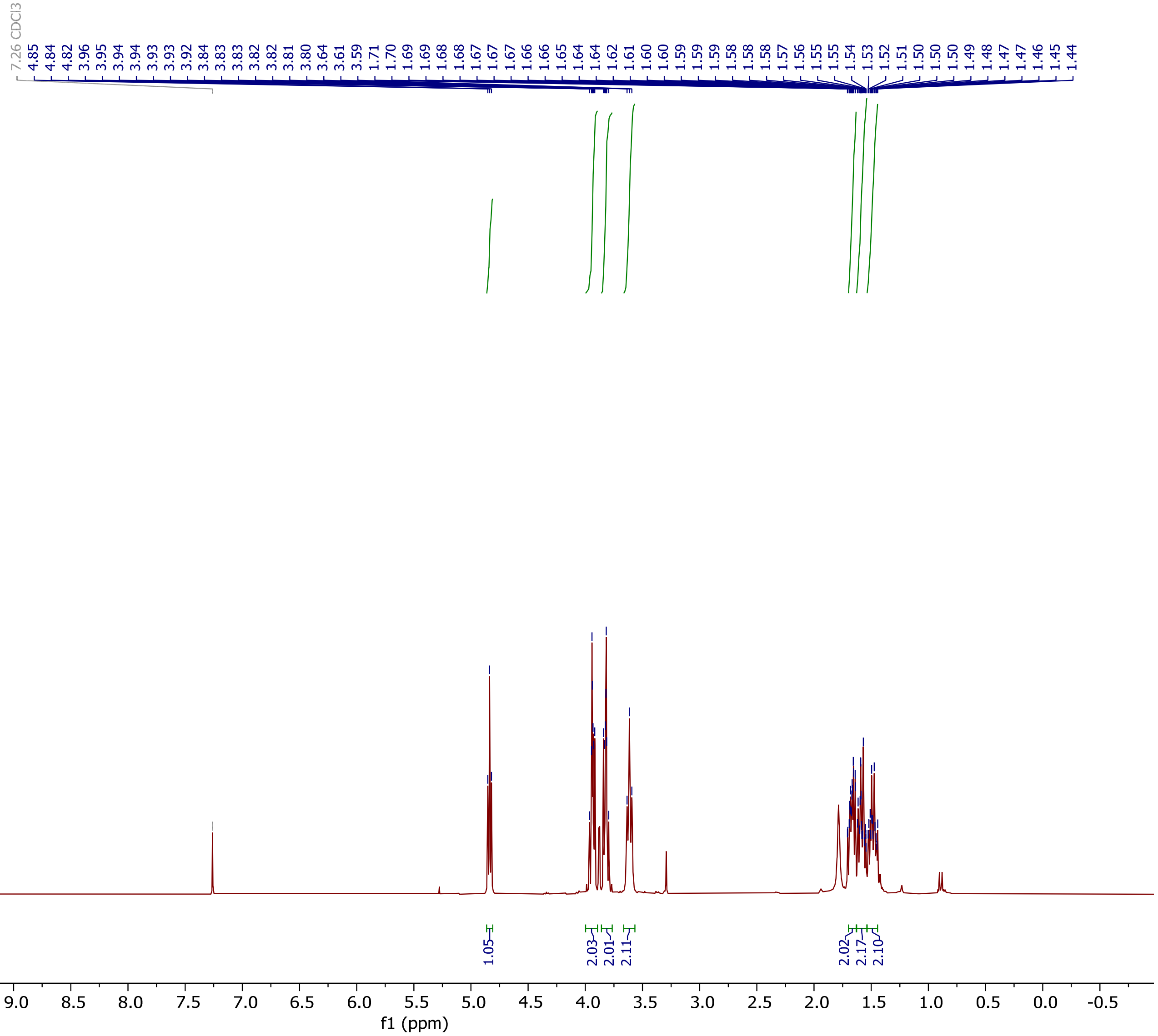

8.110 $^{13}C\{^{1}H\}$ NMR spectrum of S27e

OJJ-185.2.fid
OJJ-185
13C in CDCl3, 300MHz
5th of May, 2025

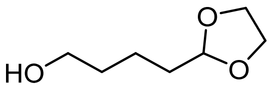

(**S27e**)

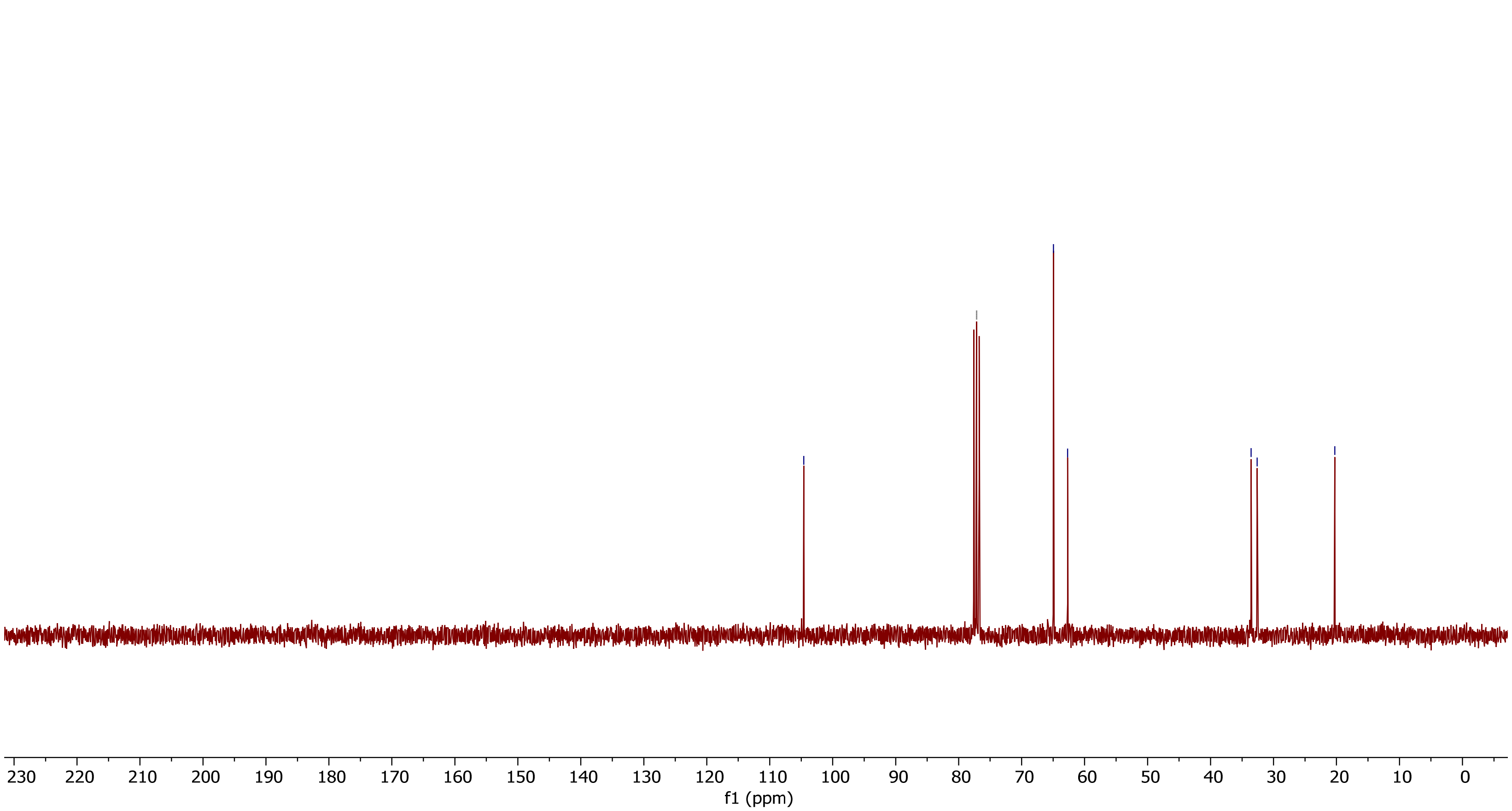

8.111 ^{1}H NMR spectrum of S26a

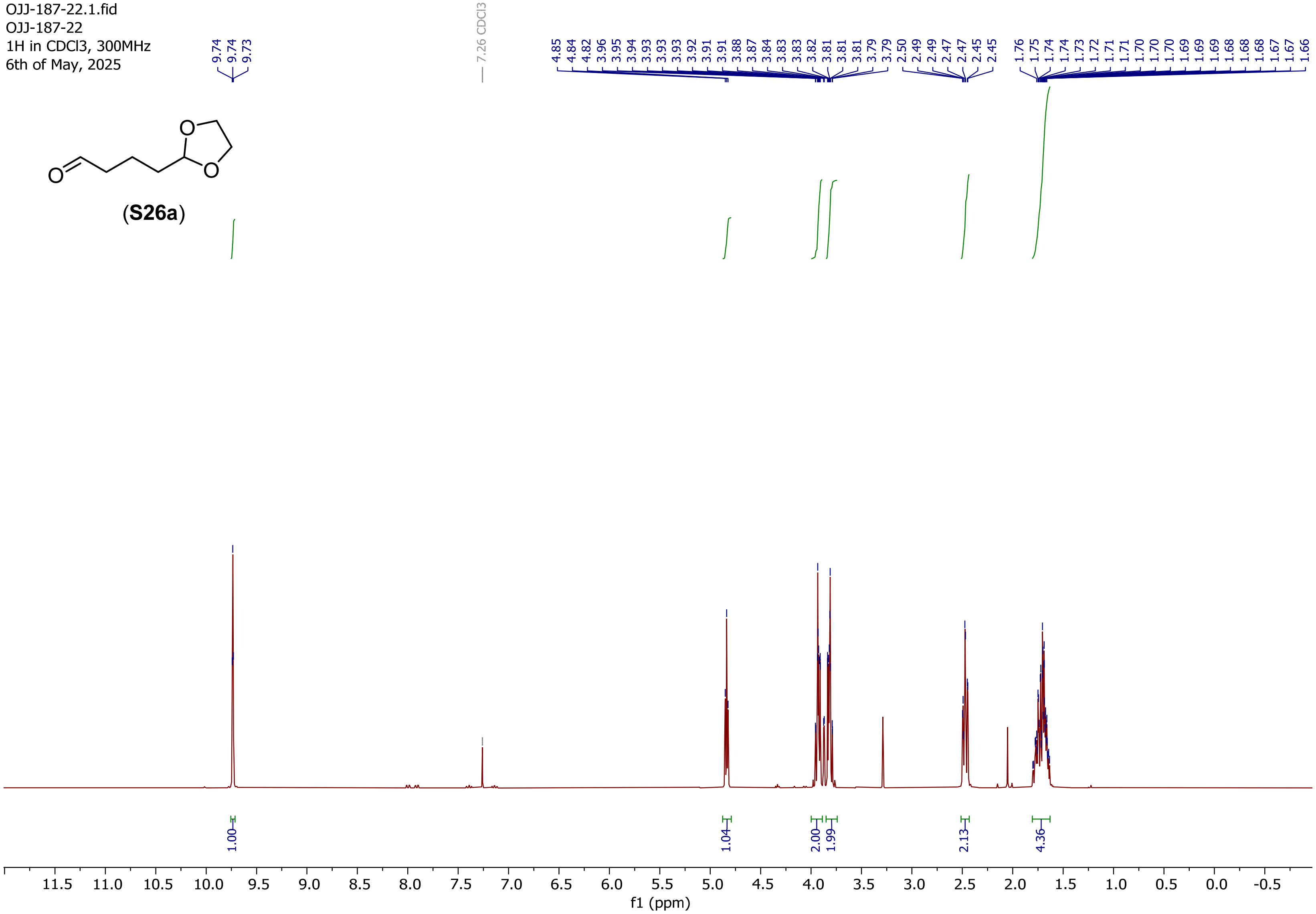

8.112 $^{13}C\{^{1}H\}$ NMR spectrum of S26a

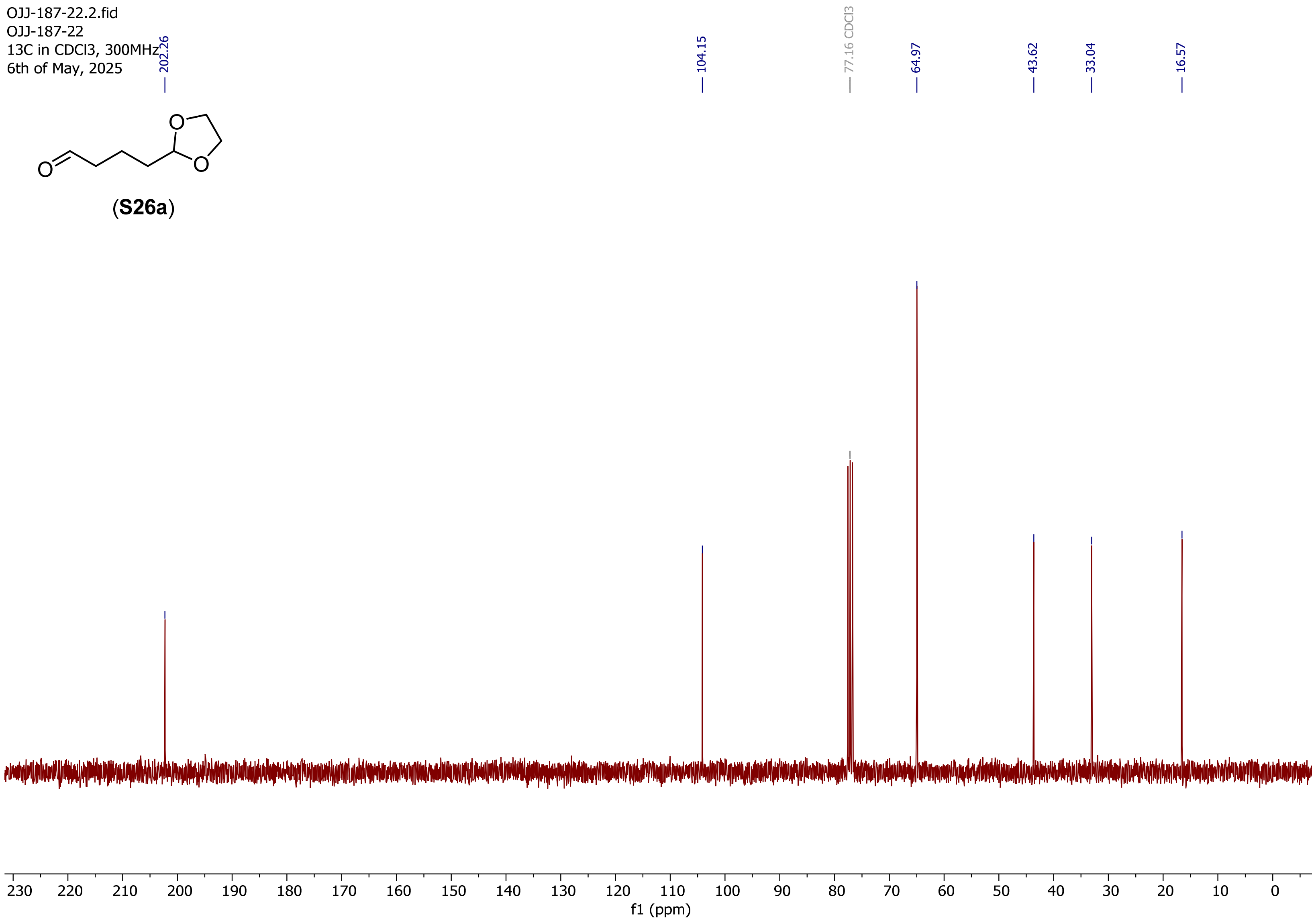

8.113 ^{1}H NMR spectrum of S26c

OJJ-192.1.fid
OJJ-192
1H in CDCl3, 300MHz
19th of May, 2025

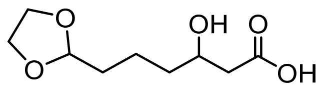

(**S26c**)

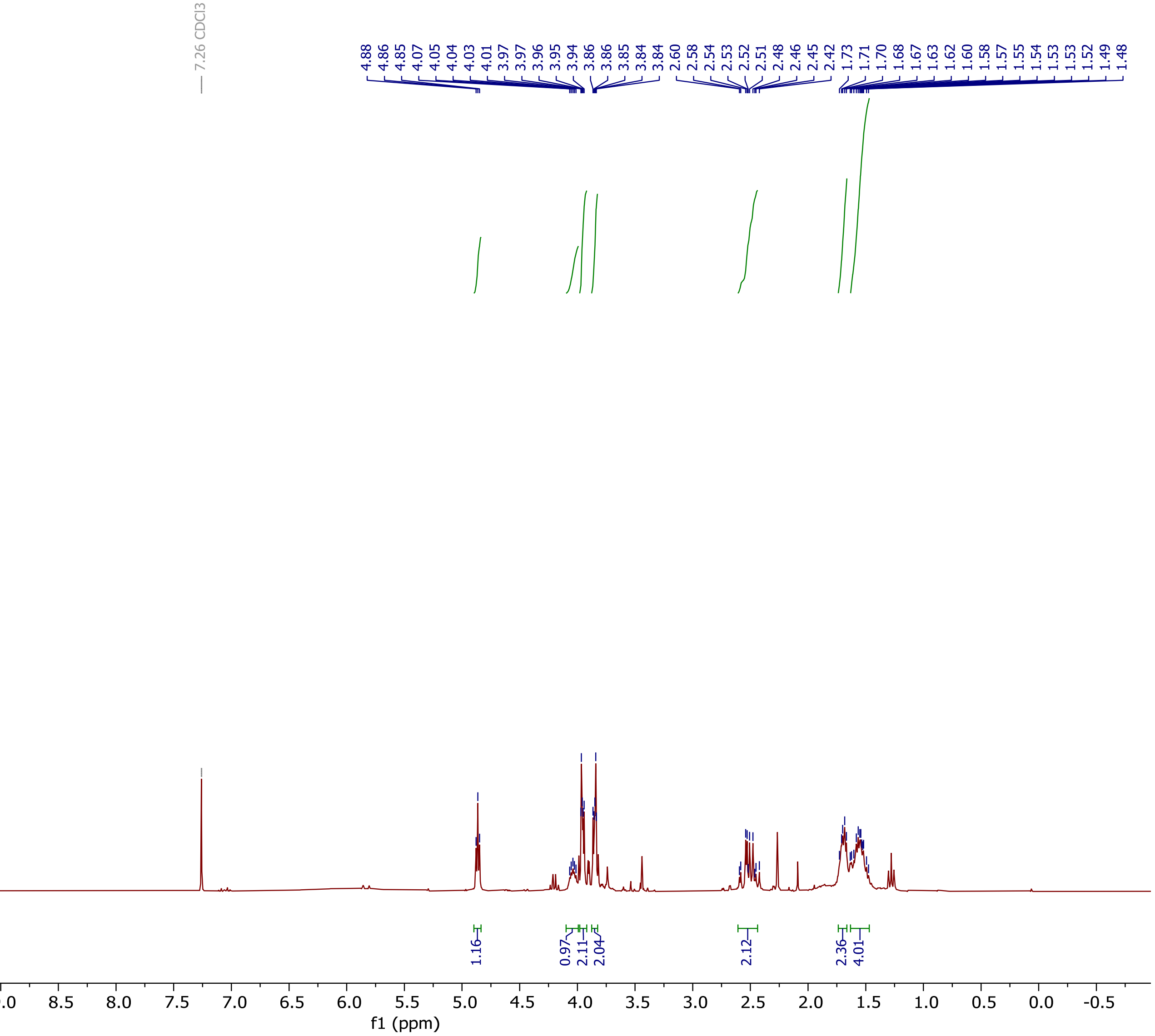

8.114 $^{13}C\{^{1}H\}$ NMR spectrum of S26c

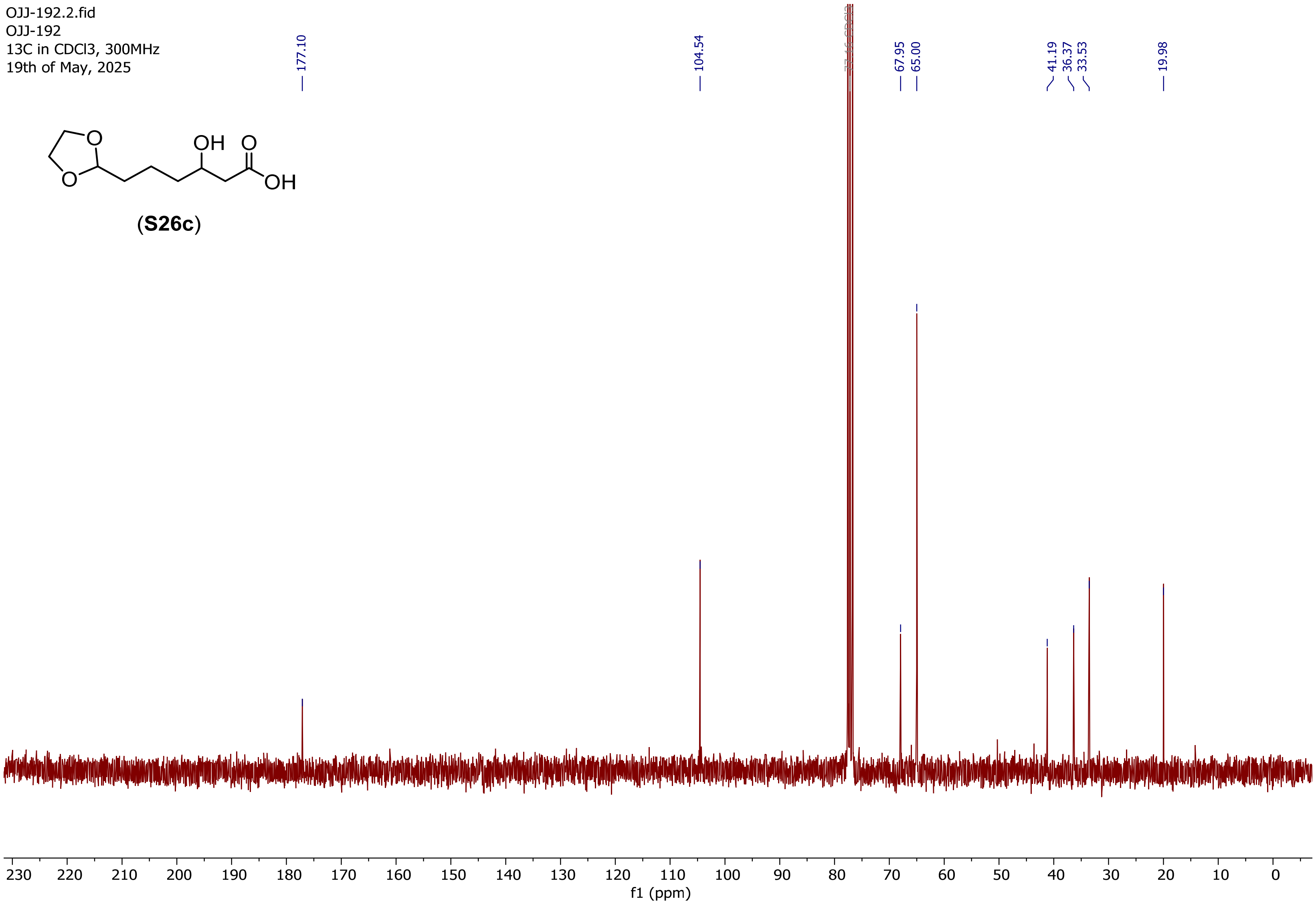

8.115 ¹H NMR spectrum of 6’g

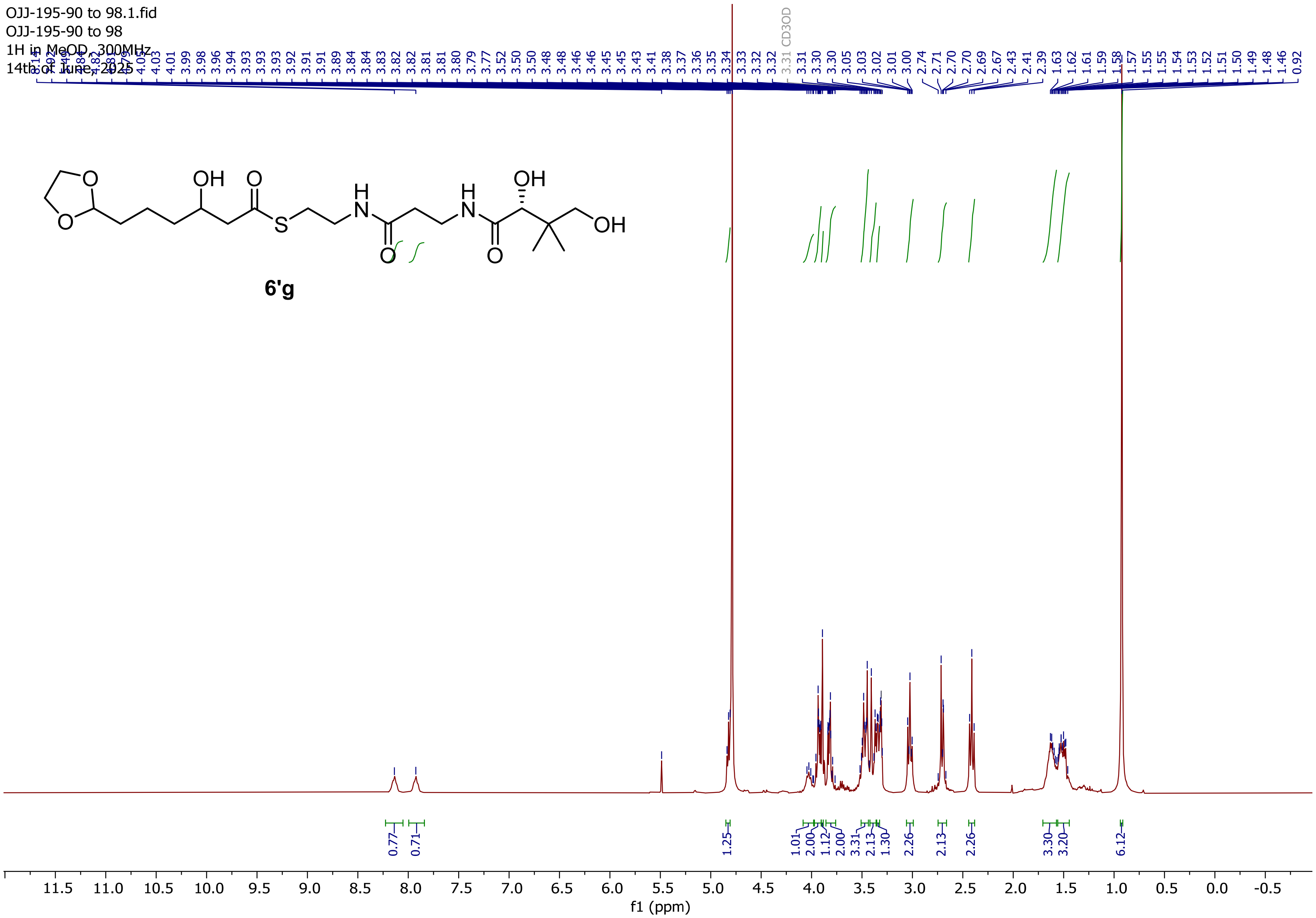

8.116 $^{13}C\{^1H\}$ NMR spectrum of 6'g

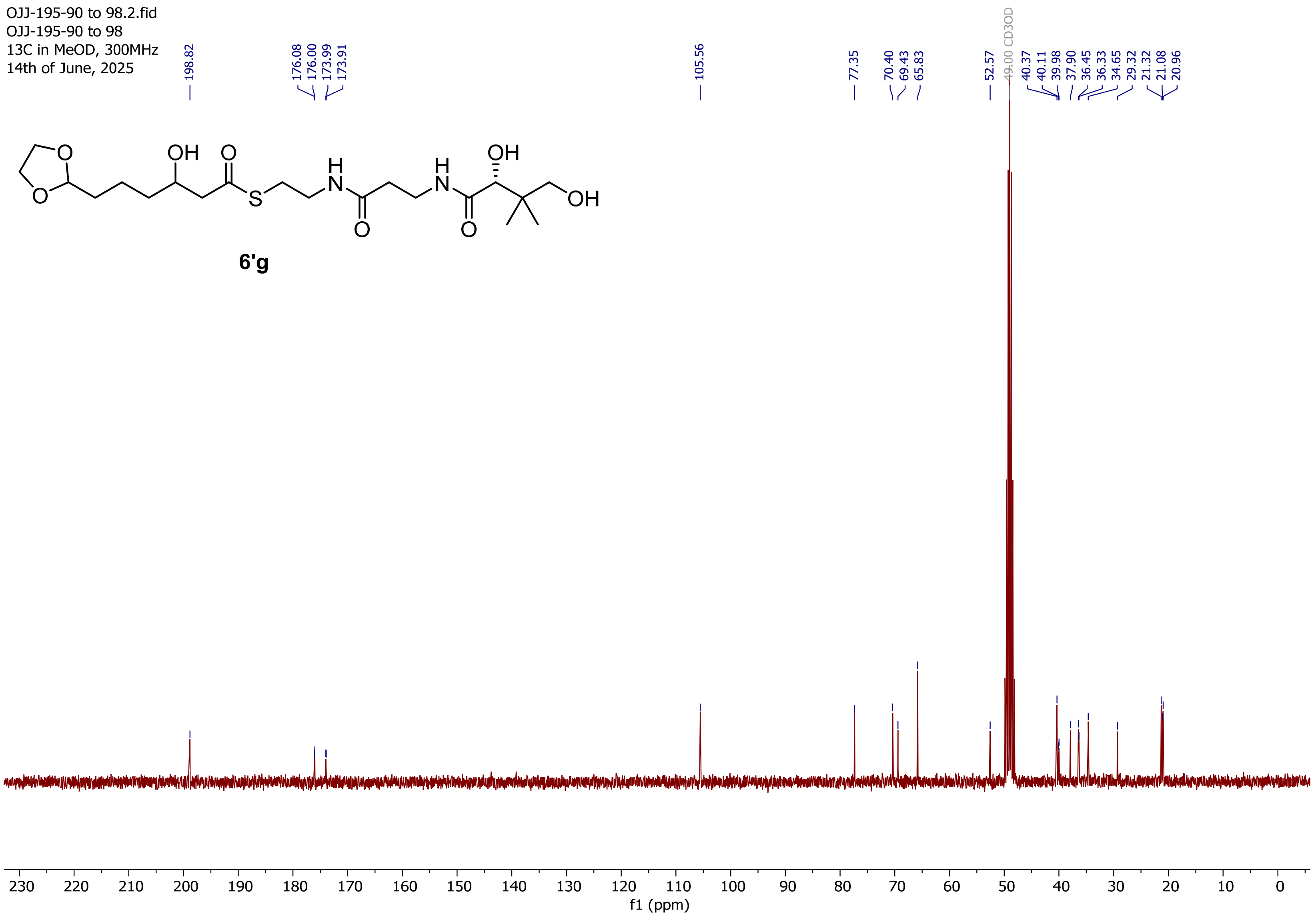

8.117 ^{1}H NMR spectrum of S28a

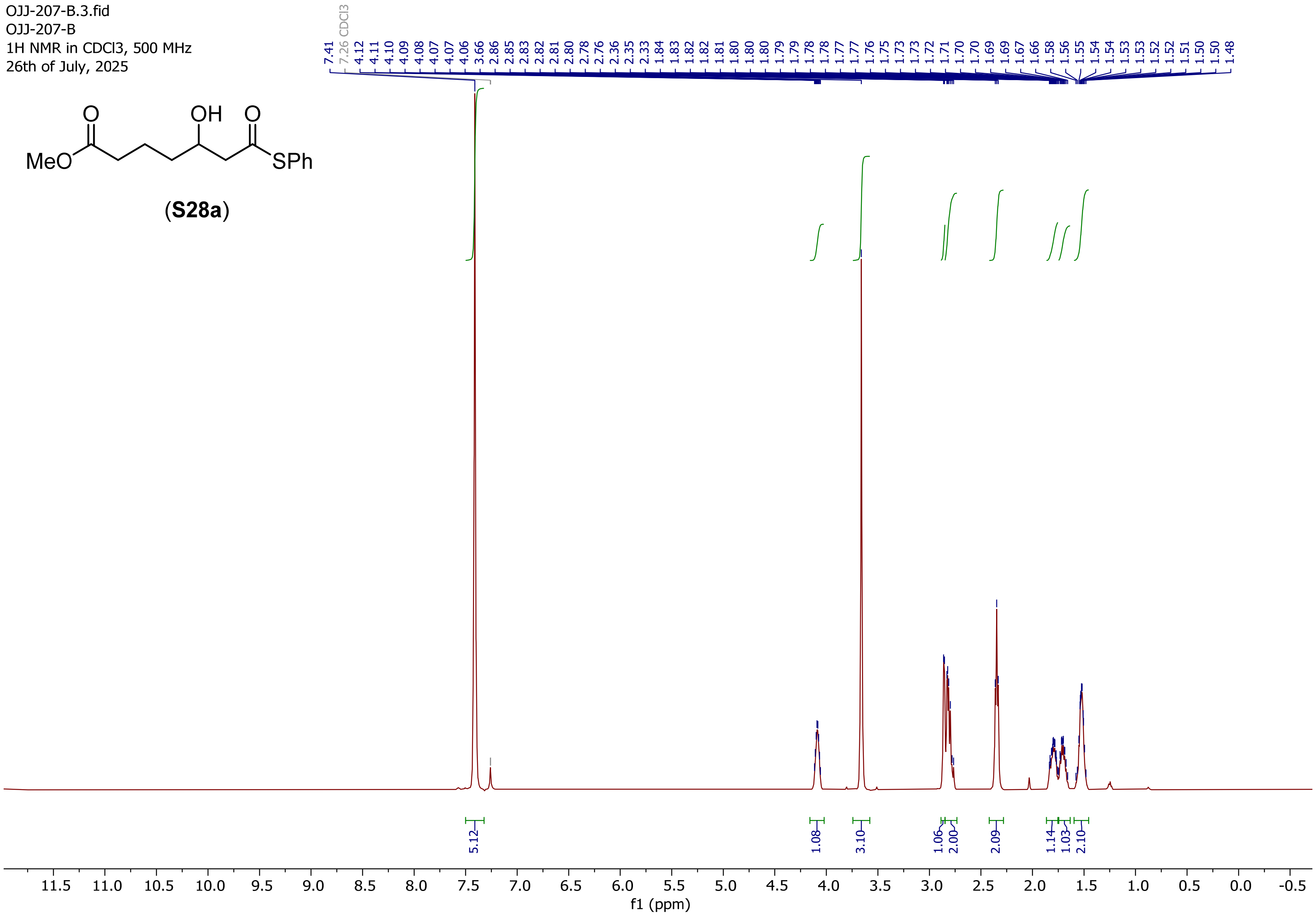

8.118 $^{13}C\{^1H\}$ NMR spectrum of S28a

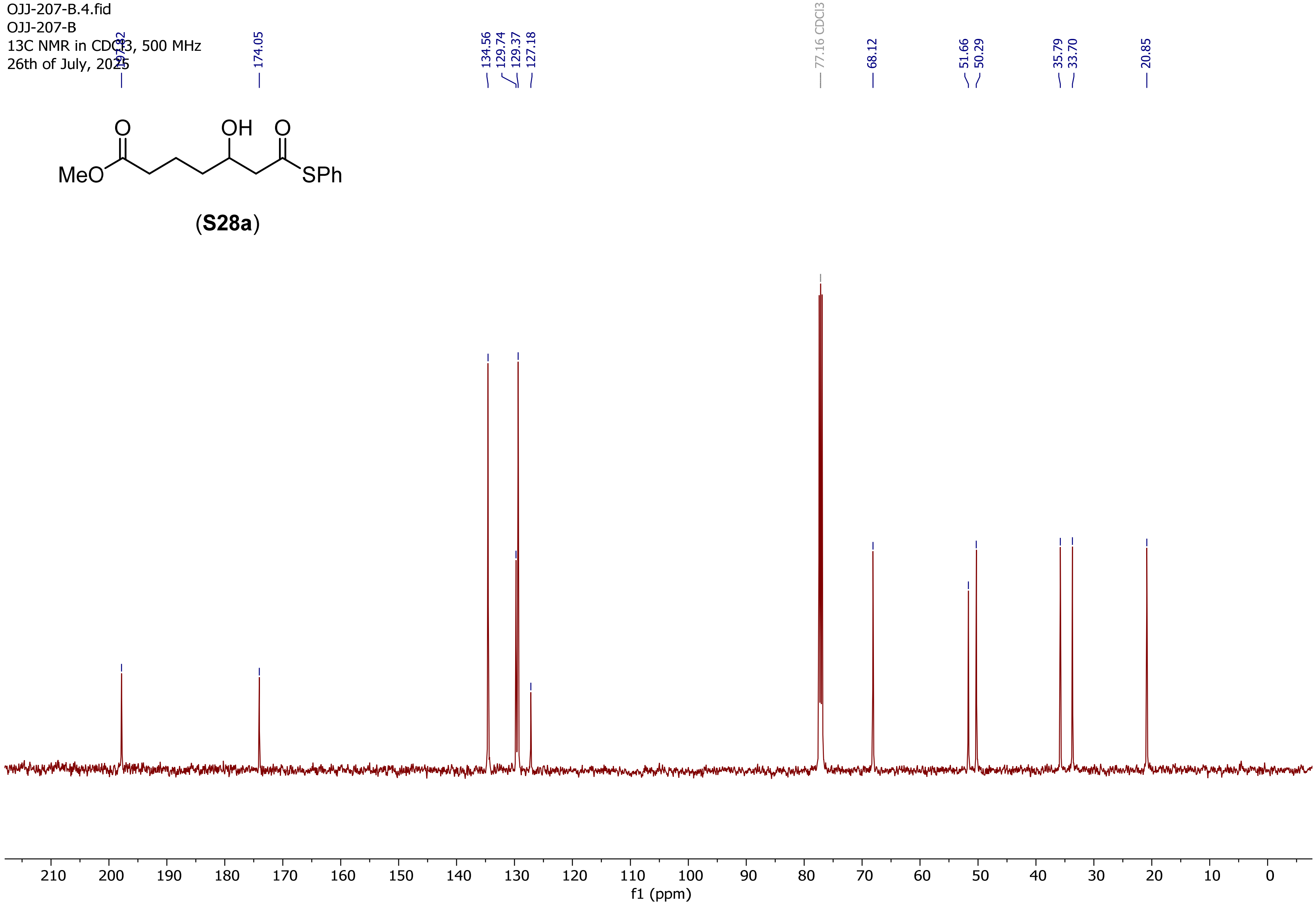

8.119 ^{1}H NMR spectrum of 6'h

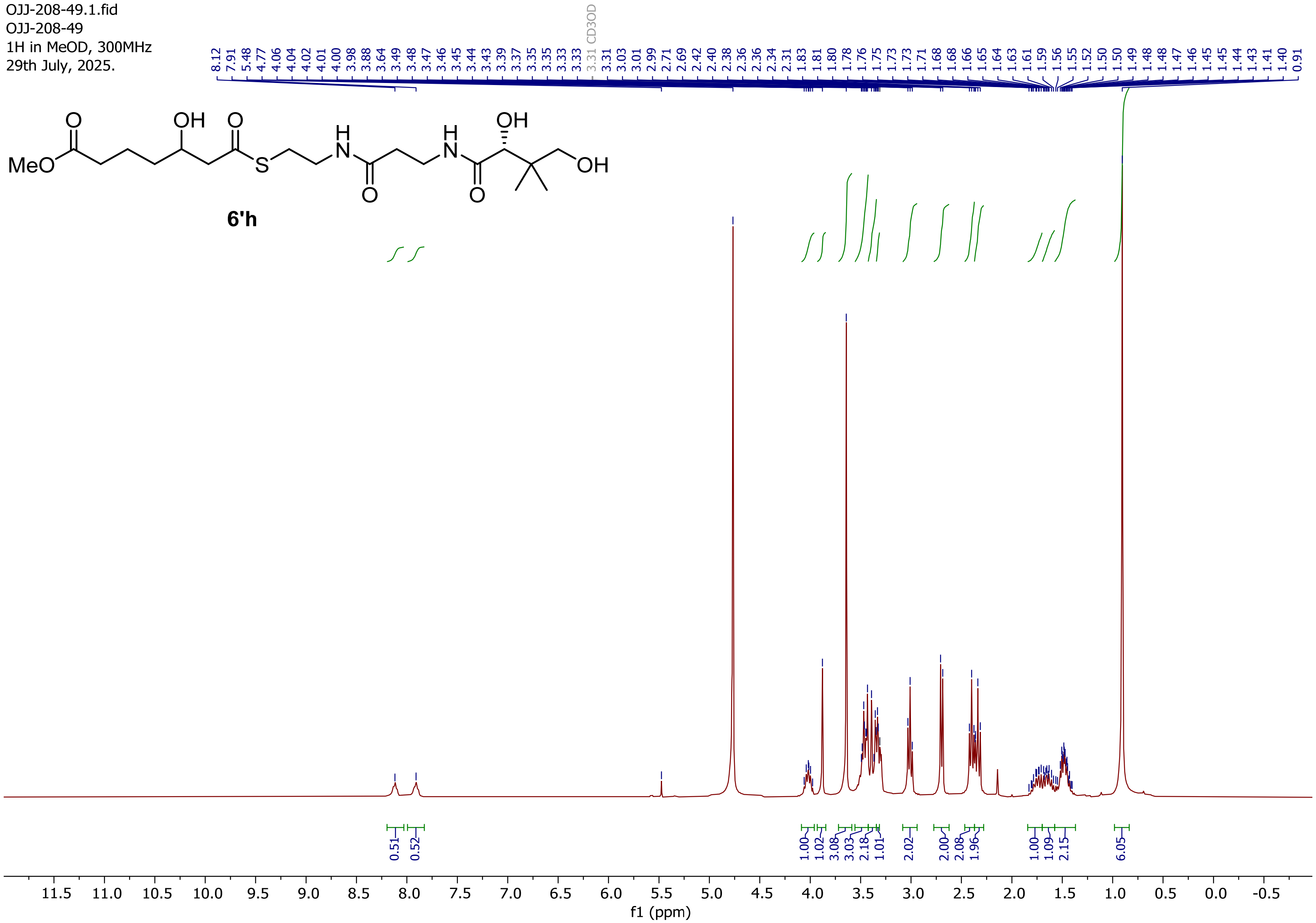

8.120 $^{13}C\{^{1}H\}$ NMR spectrum of 6'h

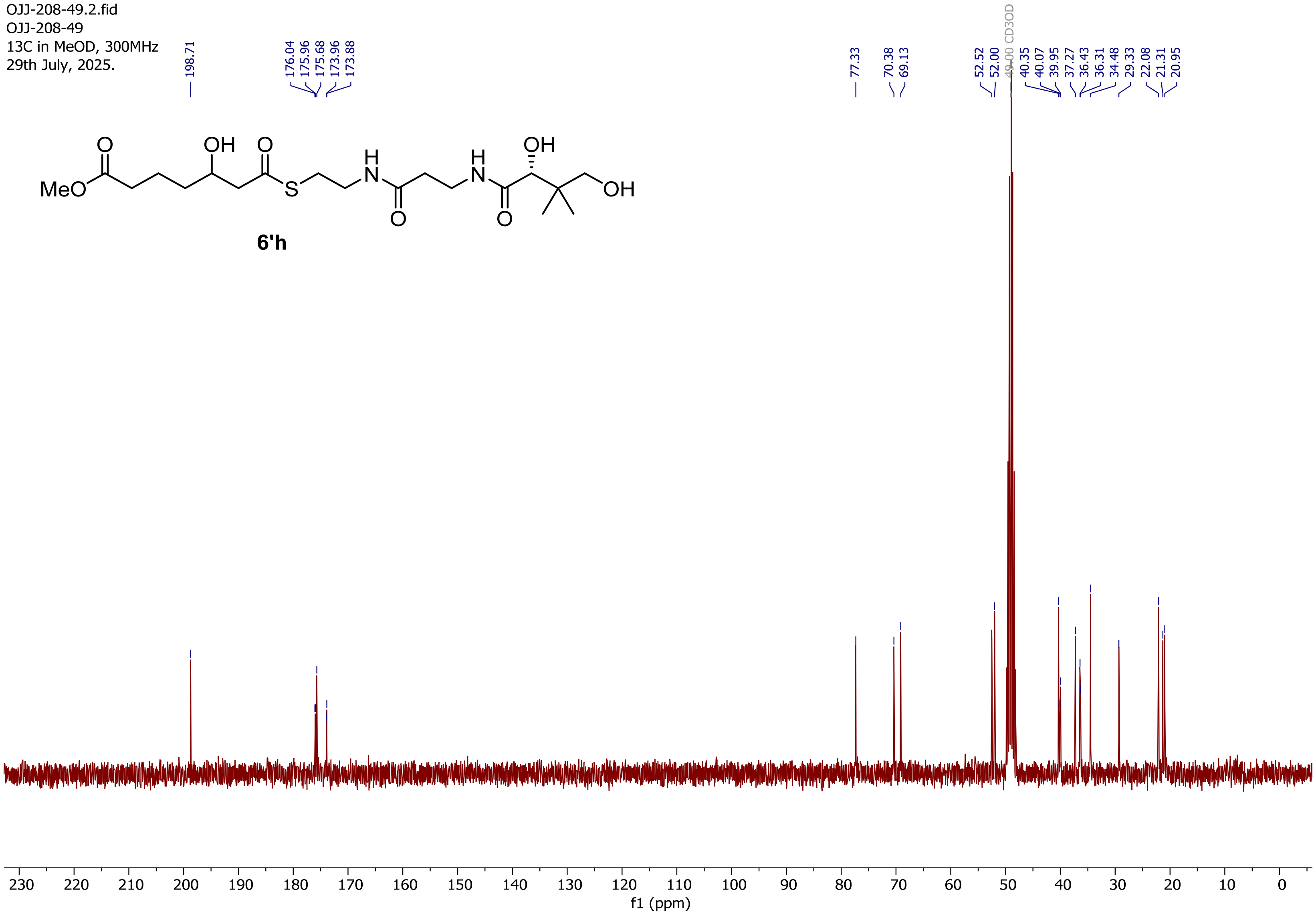

8.121 ¹H NMR spectrum of S29b

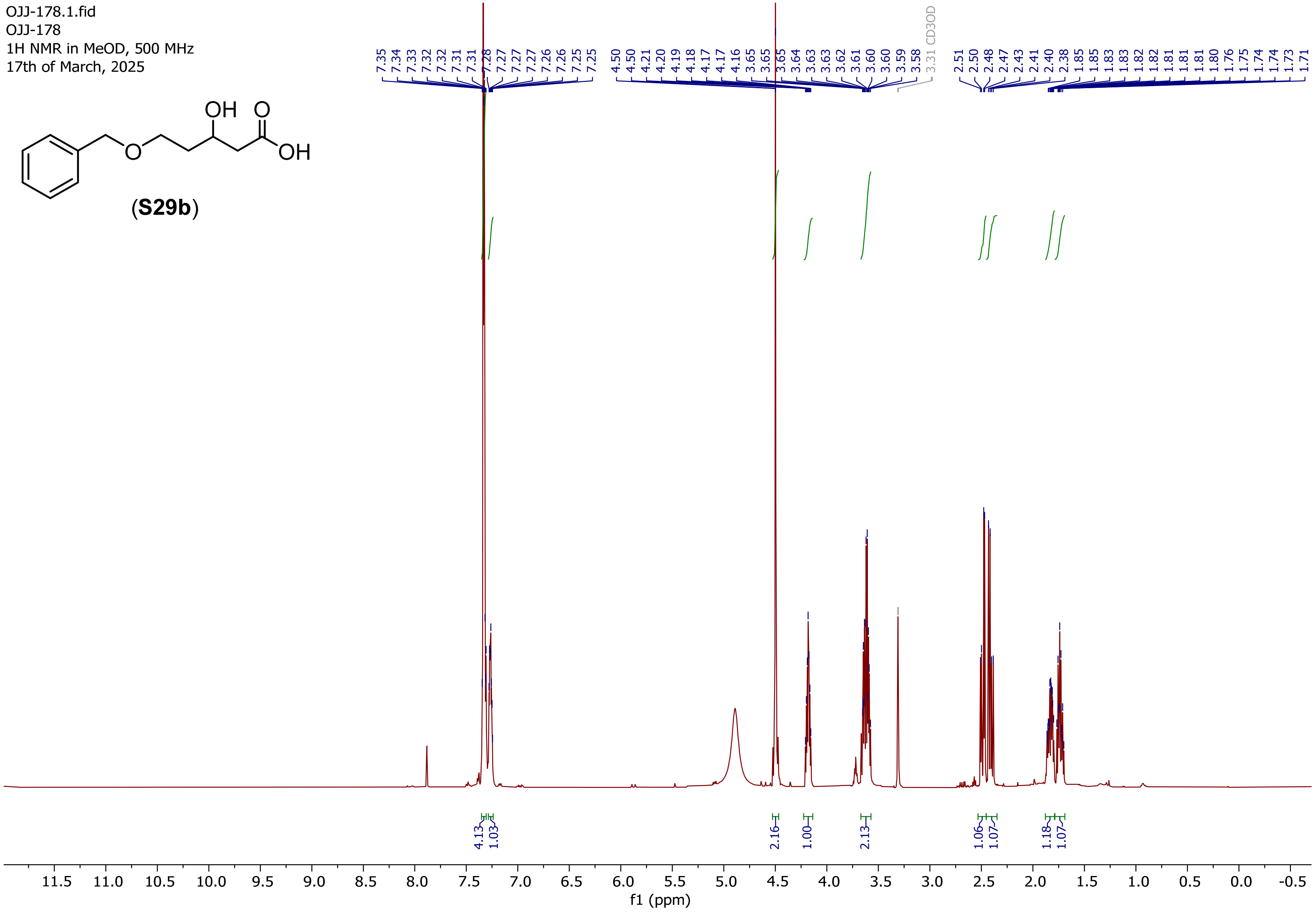

8.122 $^{13}C\{^{1}H\}$ NMR spectrum of S29b

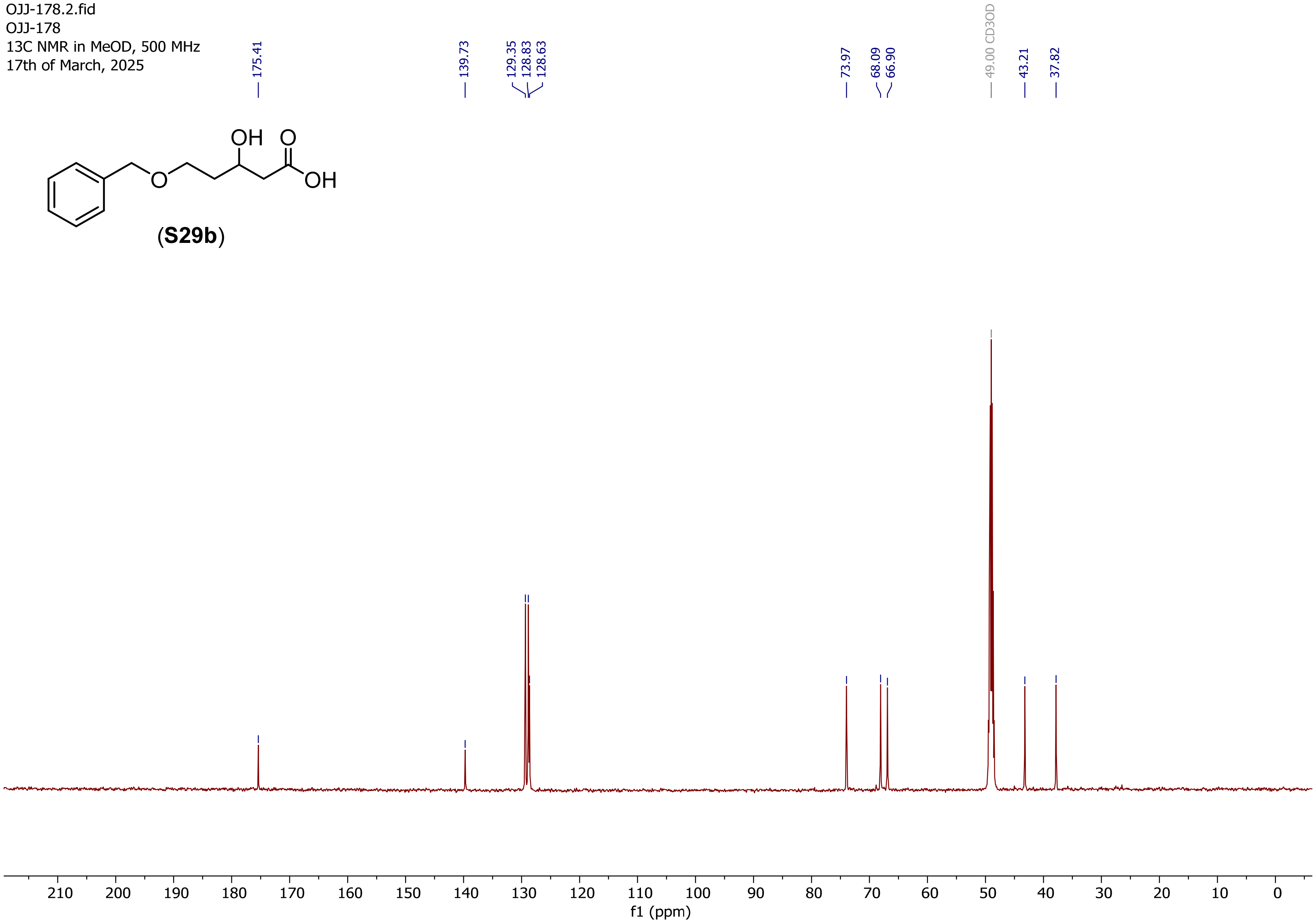

8.123 ^{1}H NMR spectrum of 6'i

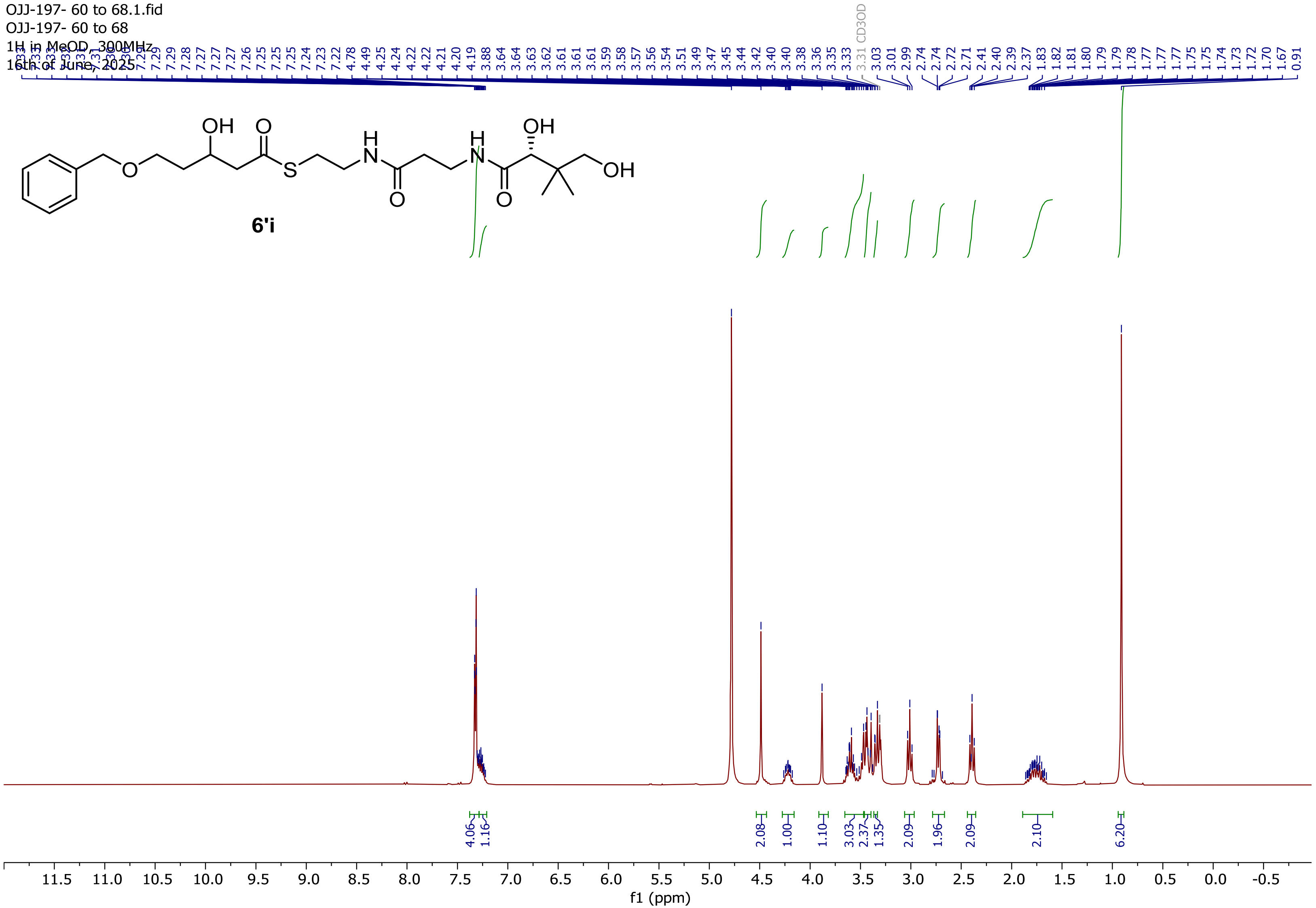

8.124 $^{13}C\{^{1}H\}$ NMR spectrum of 6'i

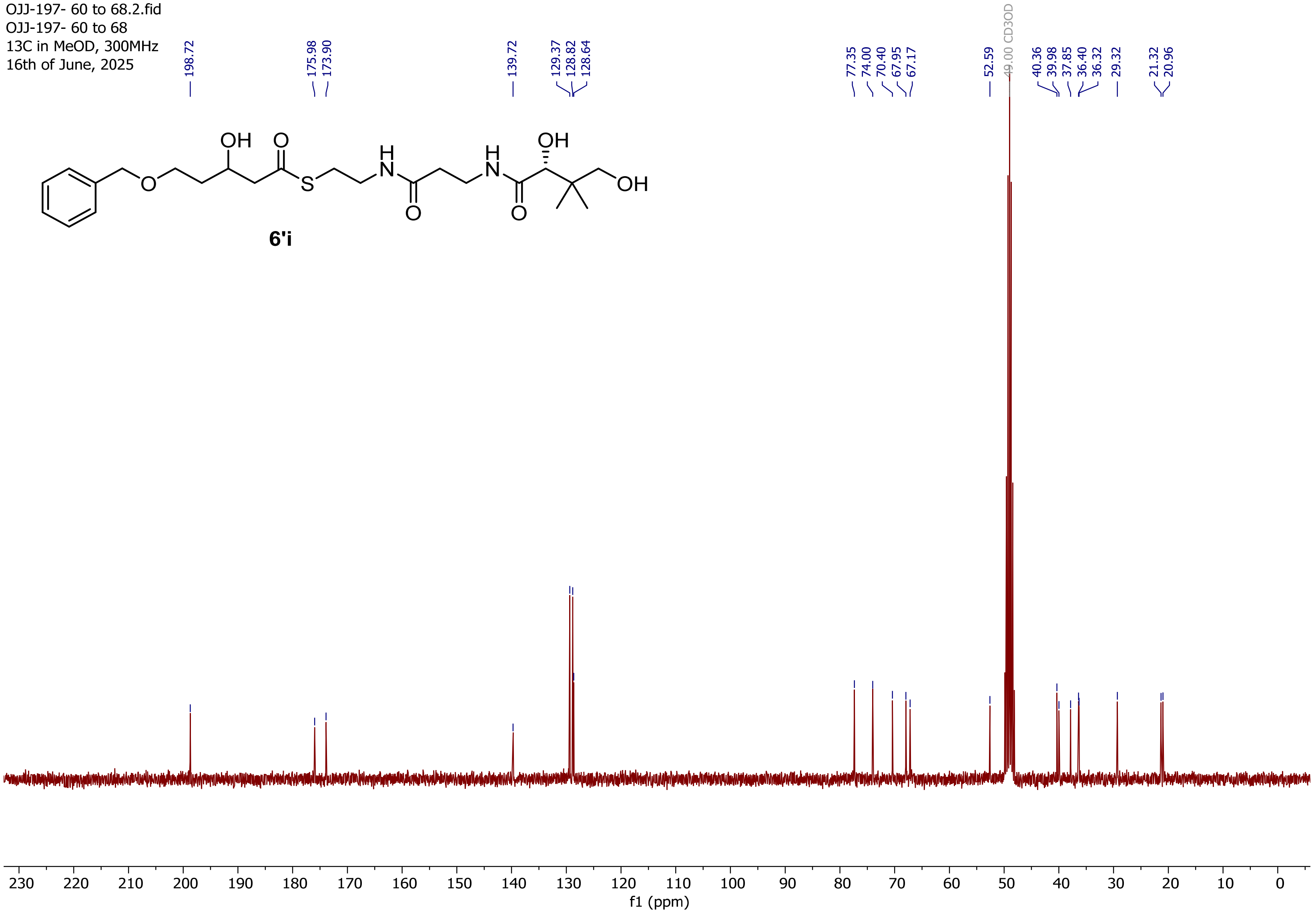

8.125 ^{1}H NMR spectrum of S30a

OJJ-159.1.fid
OJJ-159
1H NMR in CDCl3, 500 MHz
21st Feb, 2025

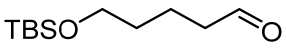

(**S30a**)

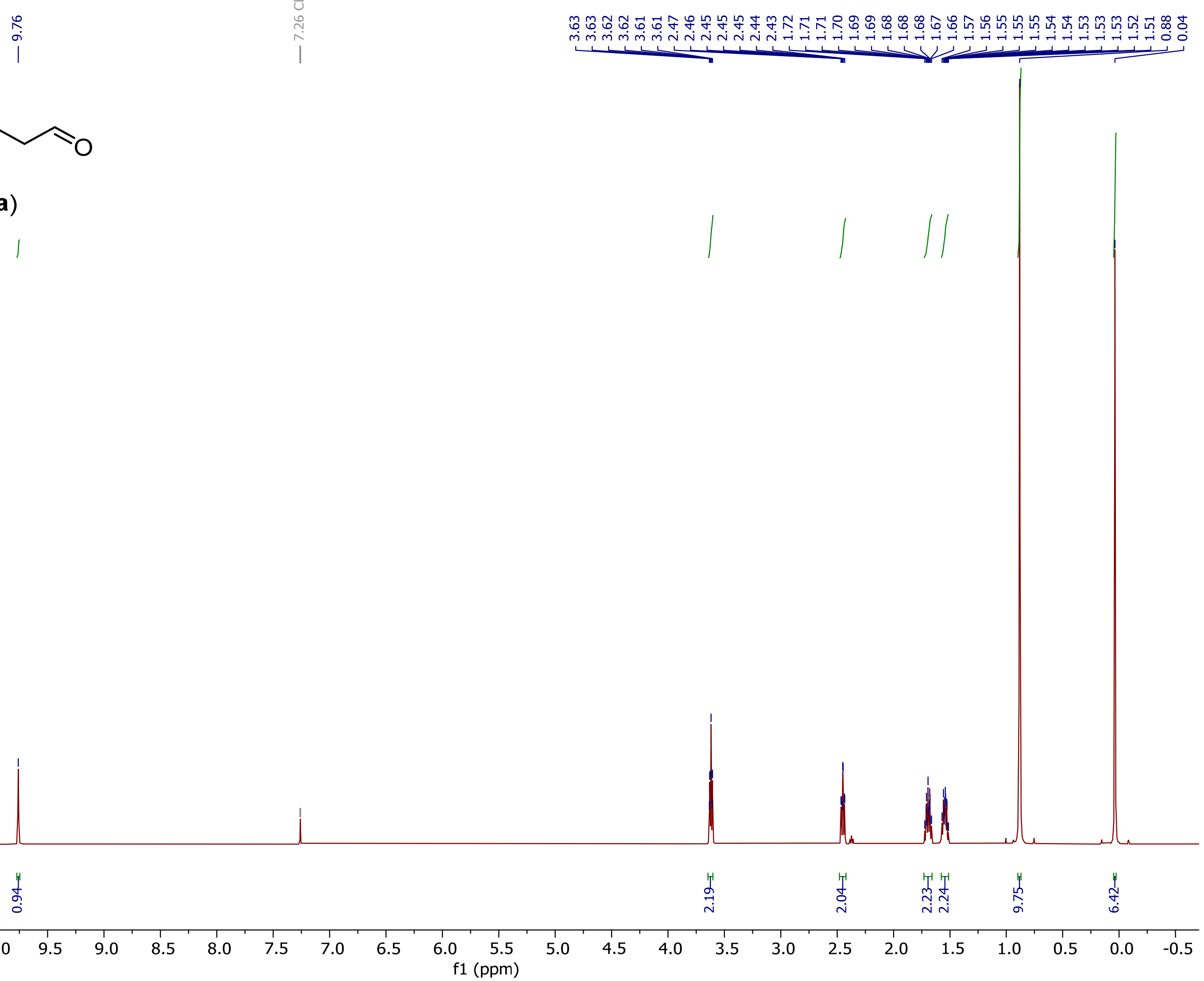

8.126 $^{13}C\{^{1}H\}$ NMR spectrum of S30a

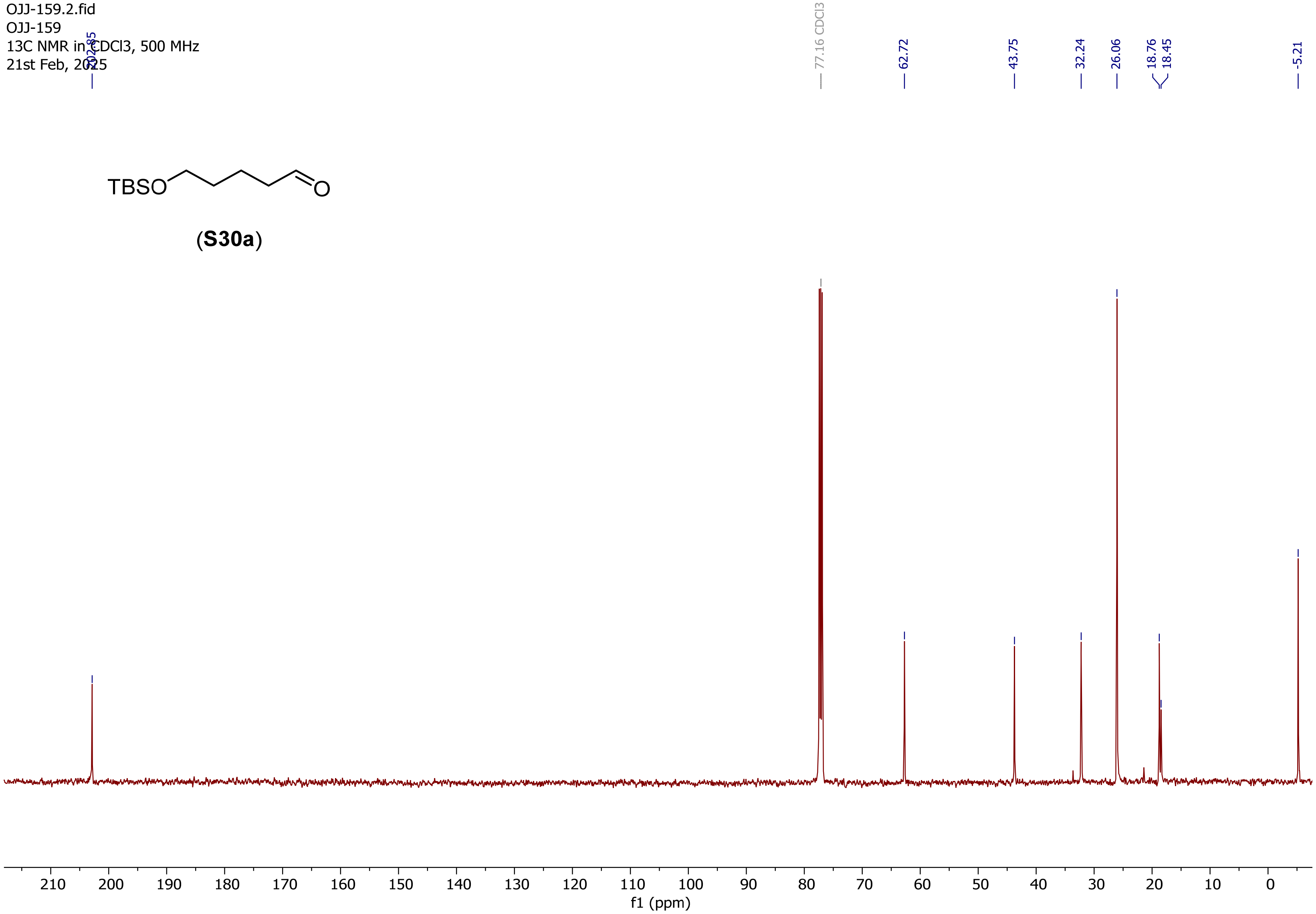

8.127 ^{1}H NMR spectrum of S30b

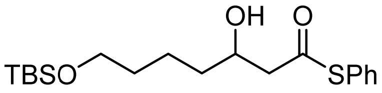

(**S30b**)

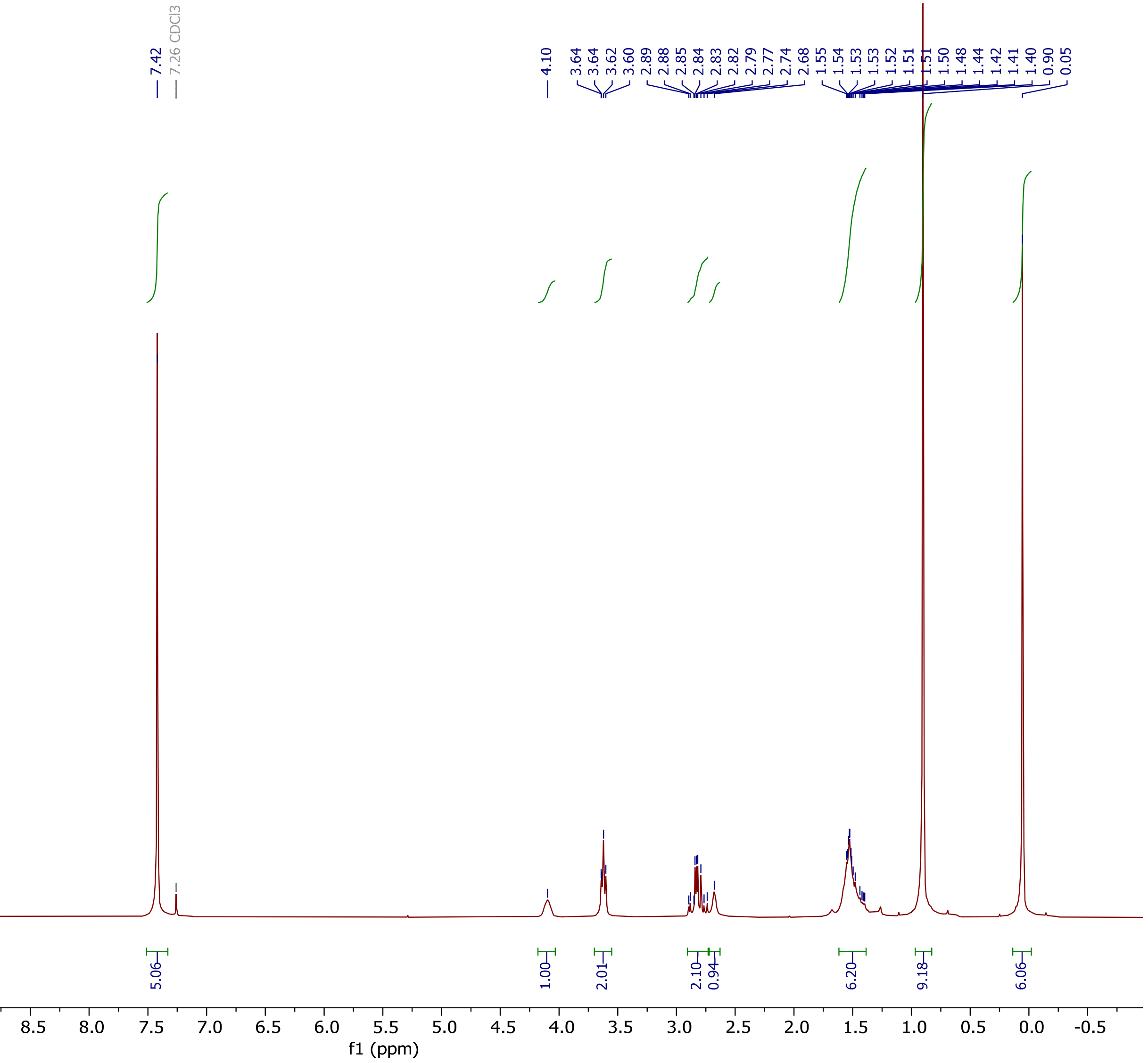

8.128 $^{13}C\{^1H\}$ NMR spectrum of S30b

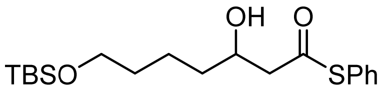

(**S30b**)

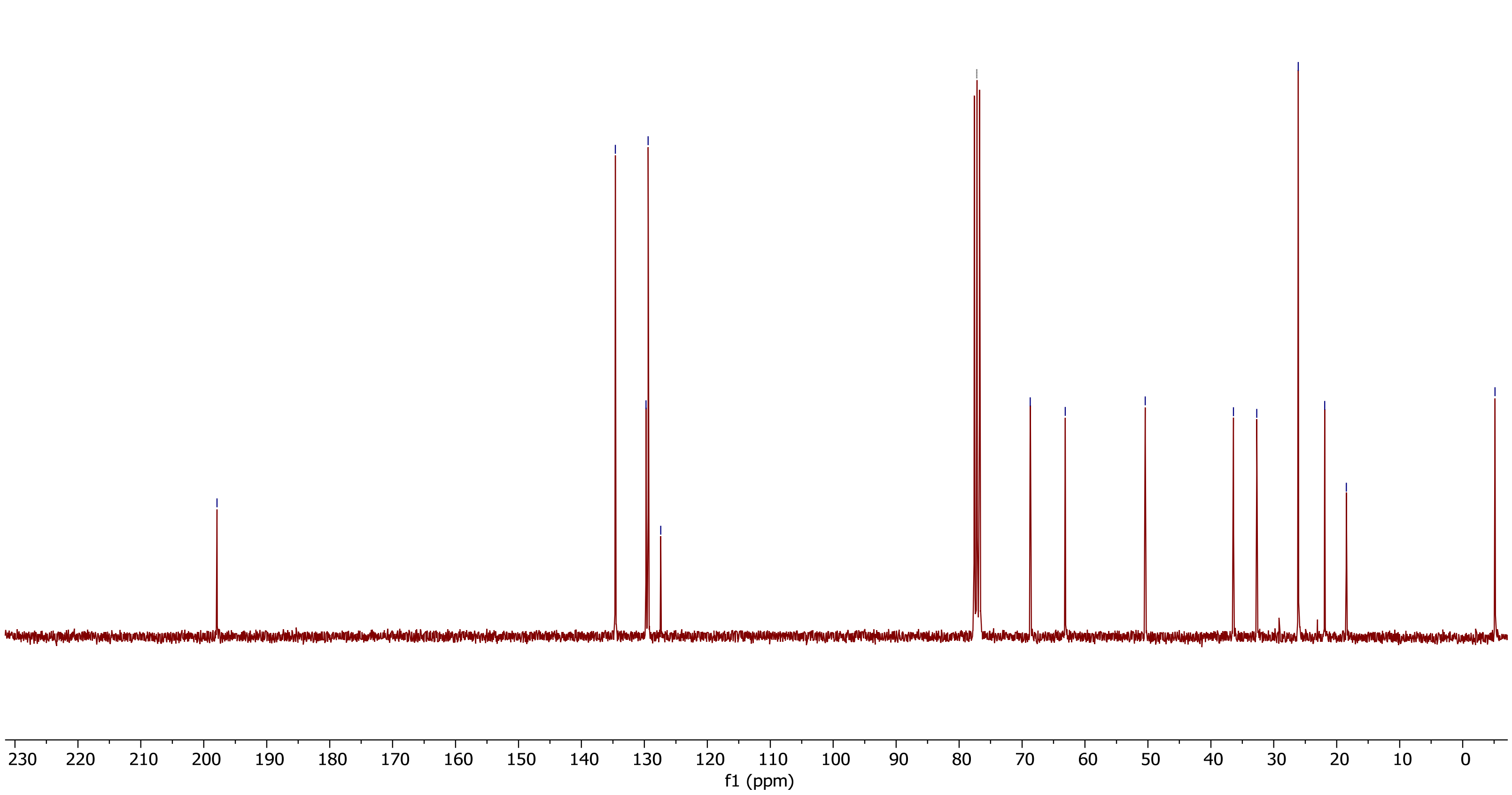

8.129 ^{1}H NMR spectrum of S30c

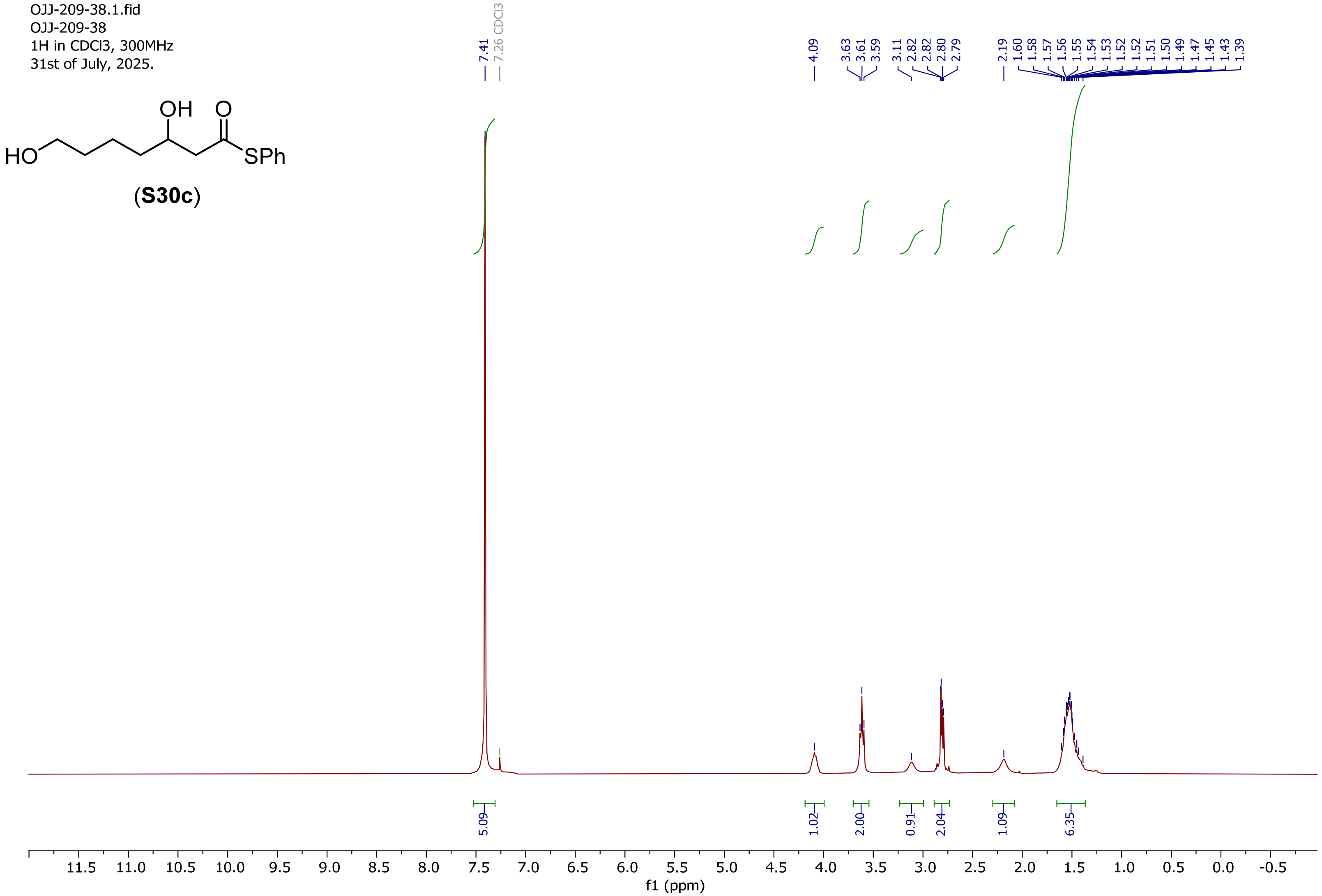

8.130 $^{13}C\{^{1}H\}$ NMR spectrum of S30c

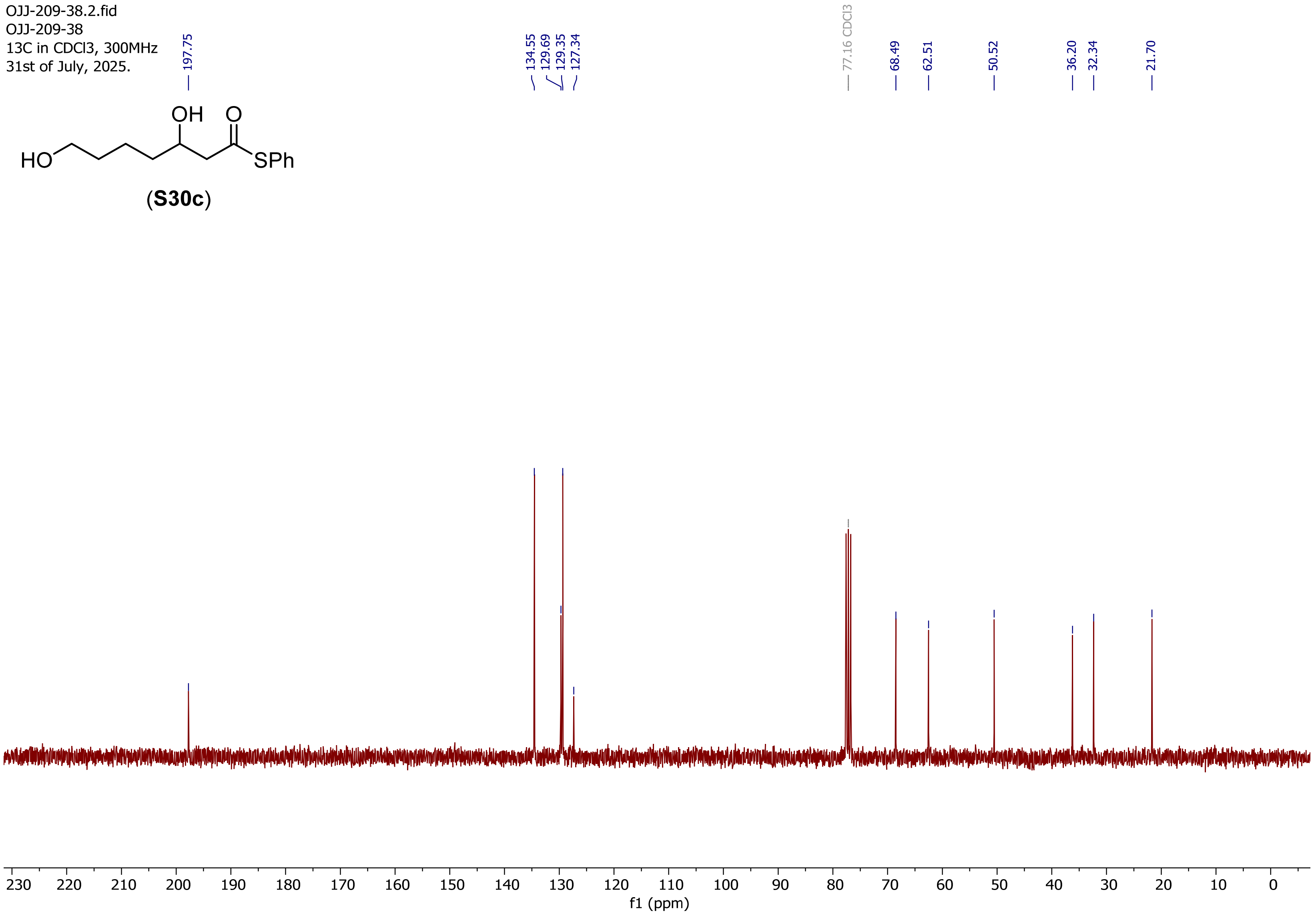

8.131 ^{1}H NMR spectrum of 6’j

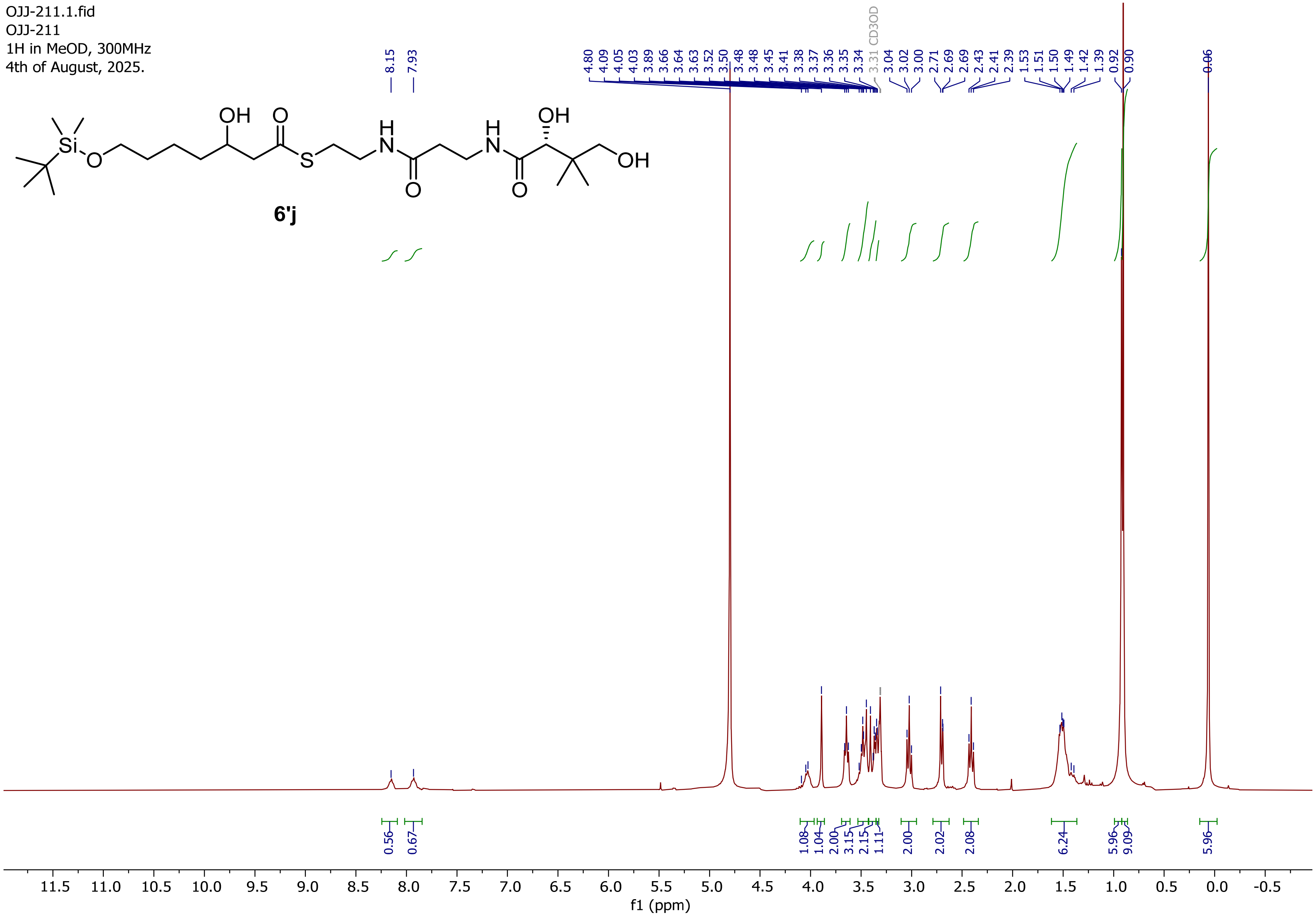

8.132 $^{13}C\{^{1}H\}$ NMR spectrum of 6’j

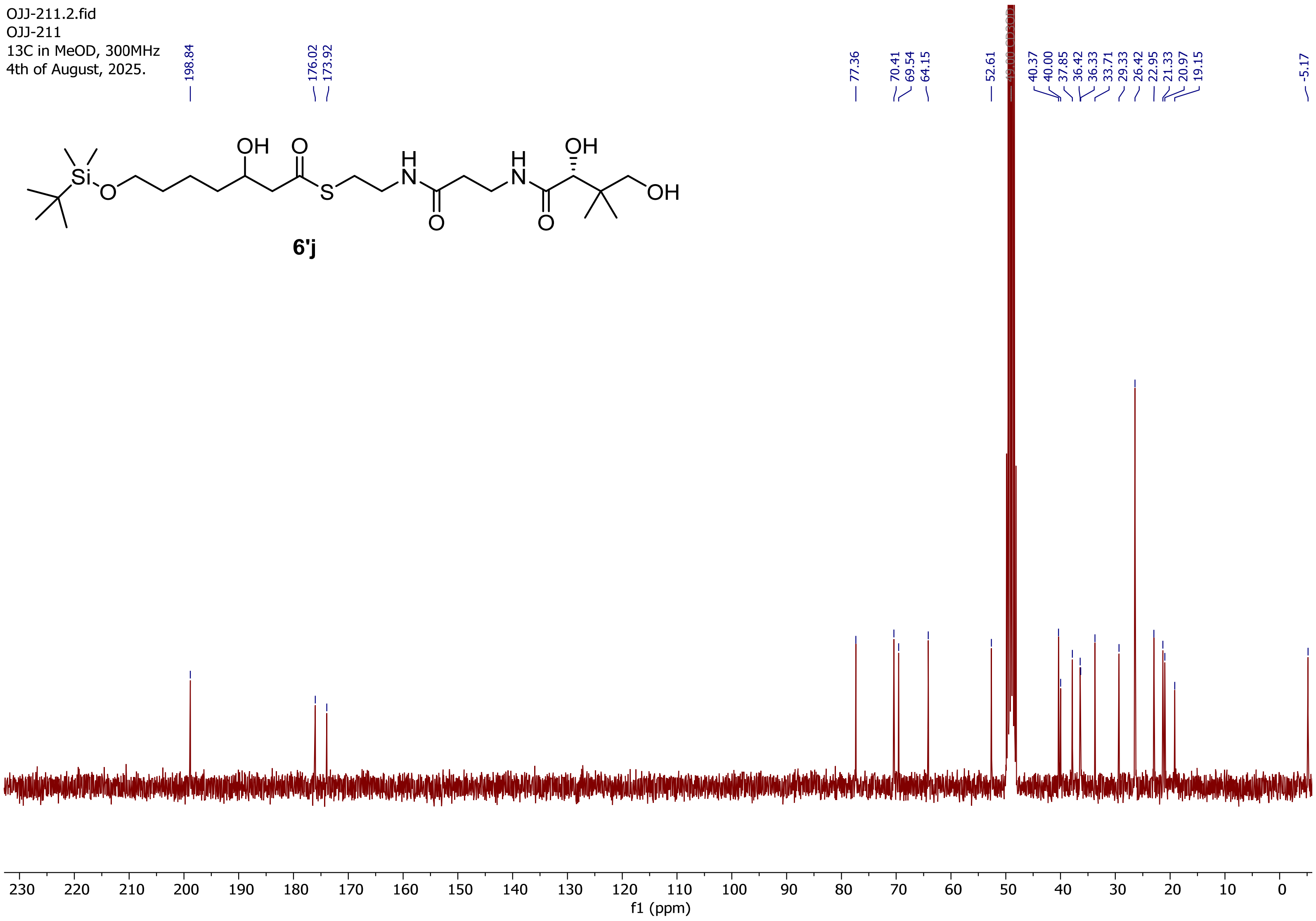

8.133 ^{1}H NMR spectrum of S31b

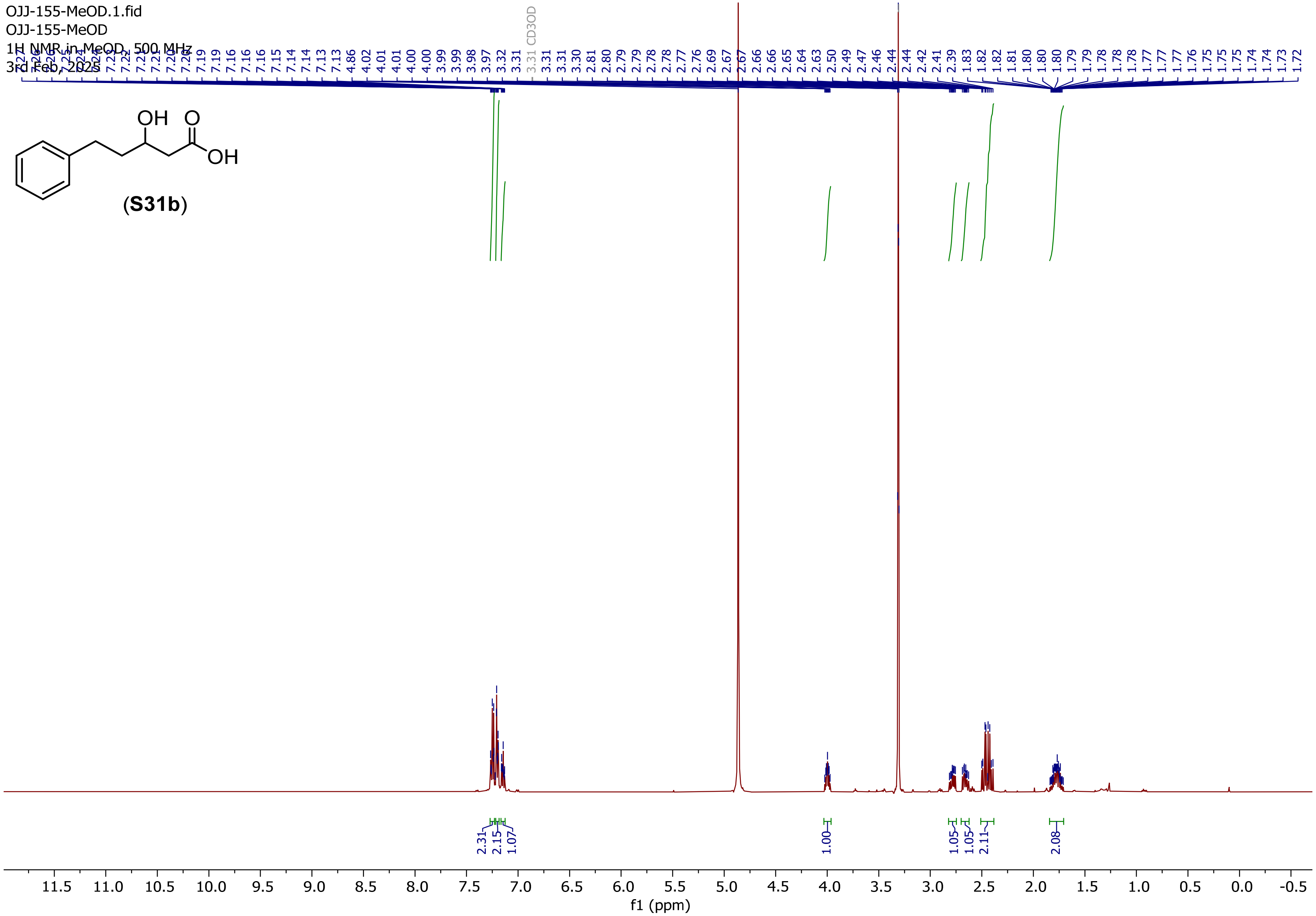

8.134 $^{13}C\{^{1}H\}$ NMR spectrum of S31b

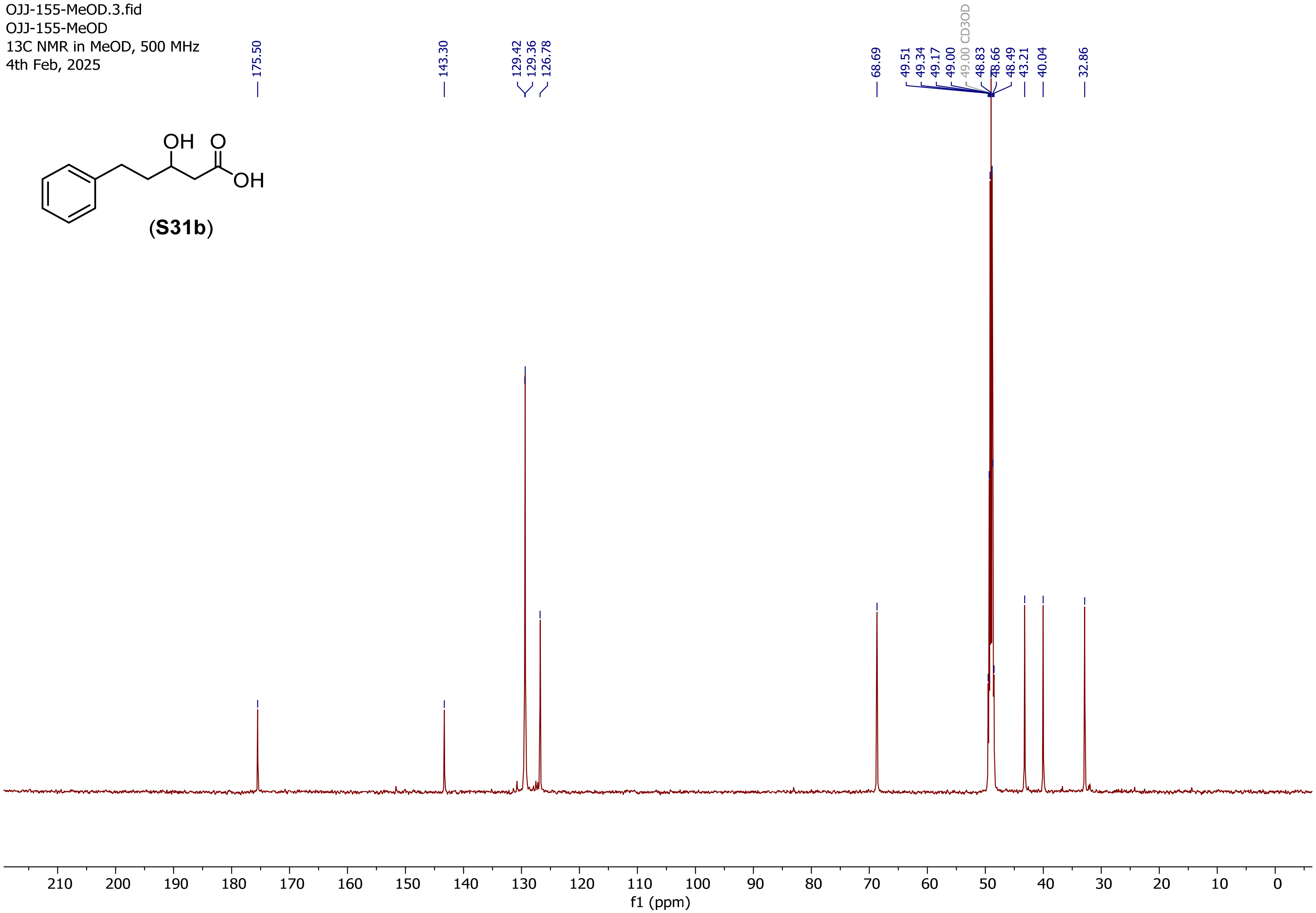

8.135 ¹H NMR spectrum of 6’k

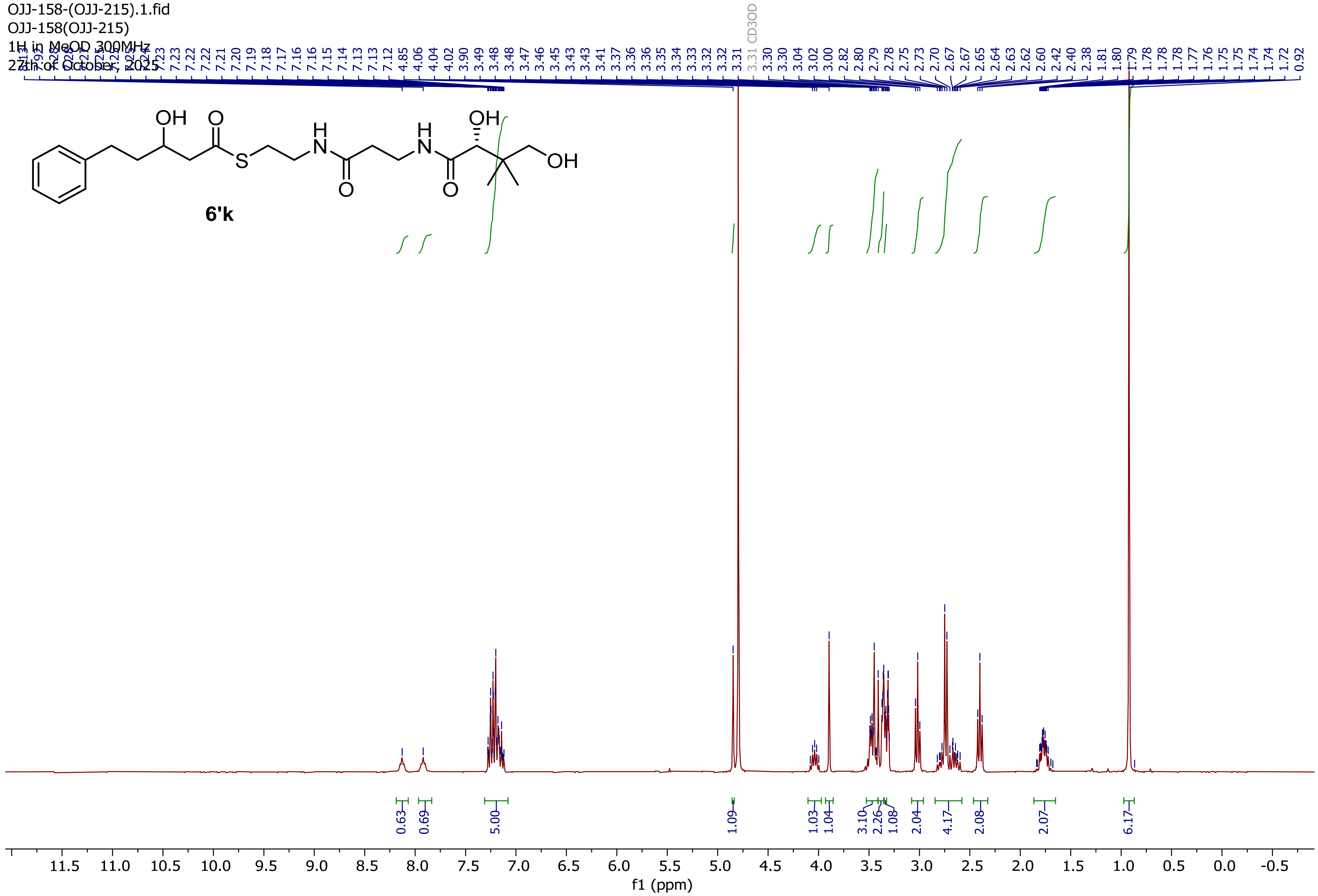

8.136 $^{13}C\{^{1}H\}$ NMR spectrum of 6’k

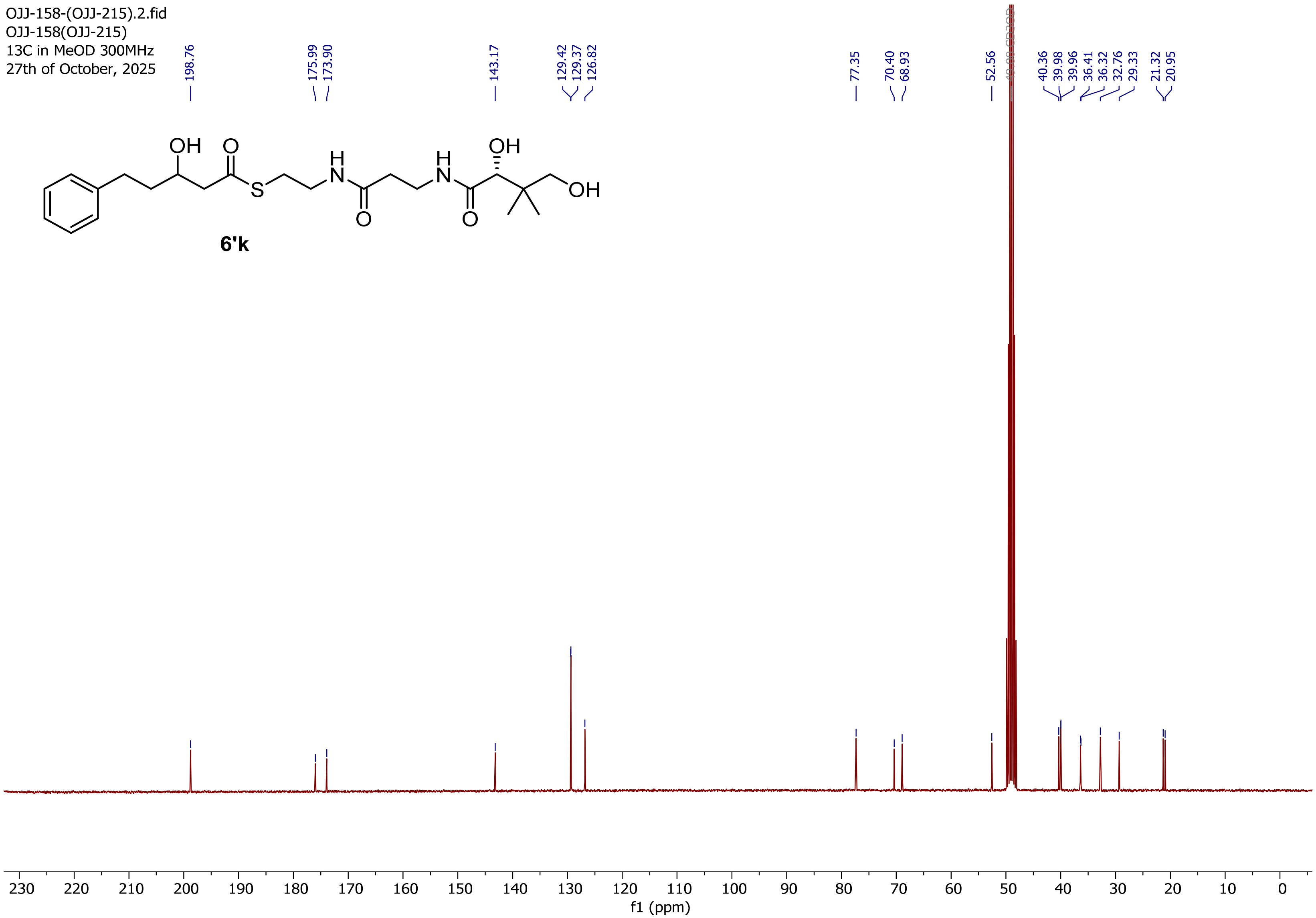

8.137 ^{1}H NMR spectrum of 7'a

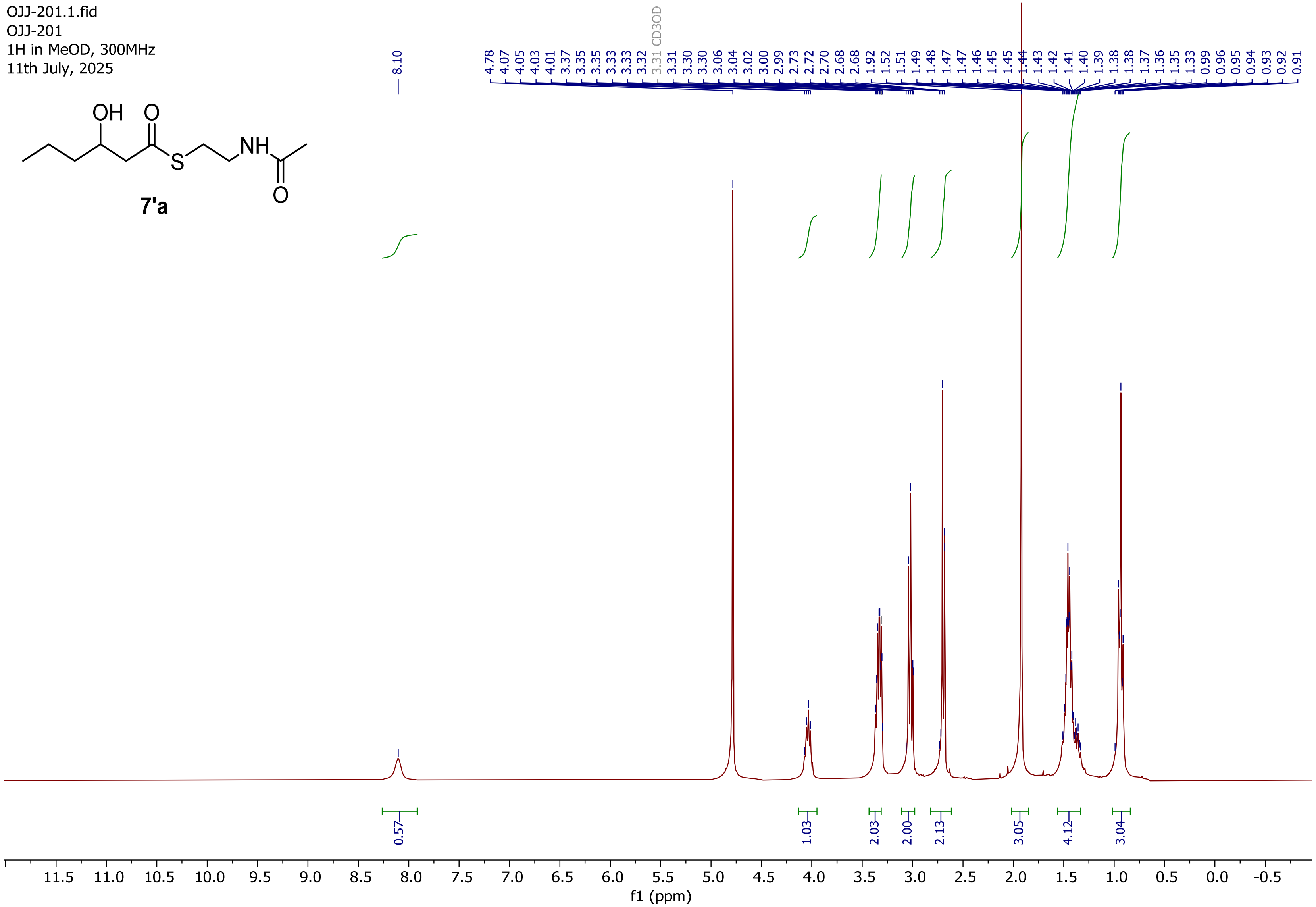

8.138 $^{13}C\{^{1}H\}$ NMR spectrum of 7'a

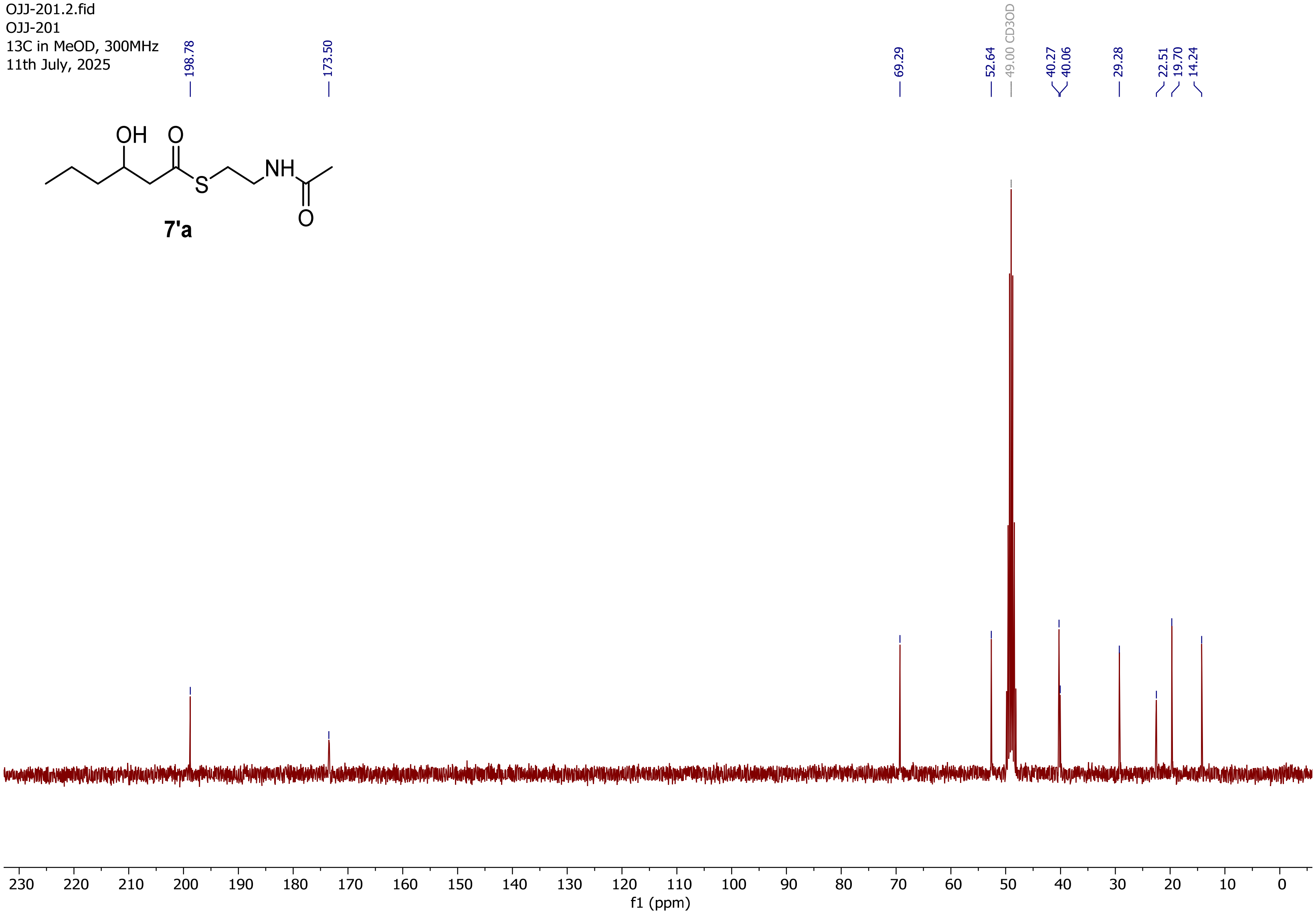

6.1 HPLC chromatograms of a) 6’a, b) hydration reaction mixture (SI, optimization table, entry 4) containing 4a+6a, and c) spike experiment (a+b)

a)

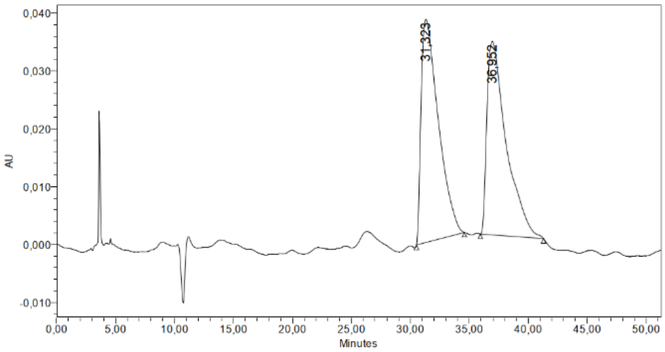

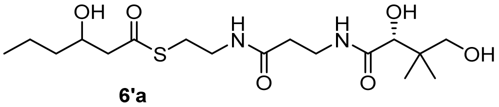

6'a

	RT	Area	% Area	Height
1	31,323	3929129	50,77	38563
2	36,952	3809417	49,23	33433

b)

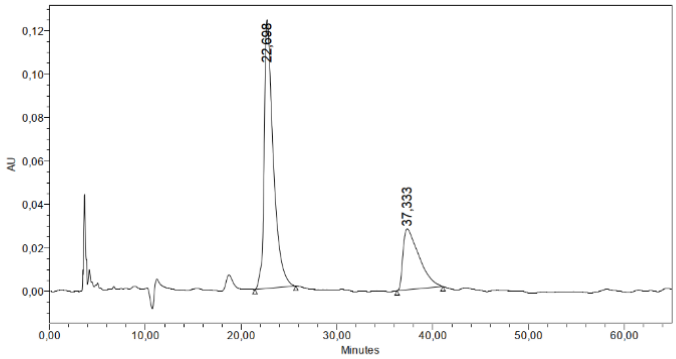

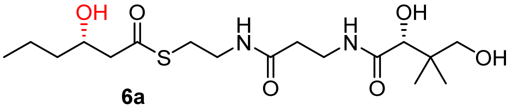

6a

dr >99:<1

	RT	Area	% Area	Height
1	22,698	8153772	71,54	123754
2	37,333	3244168	28,46	28008

c)

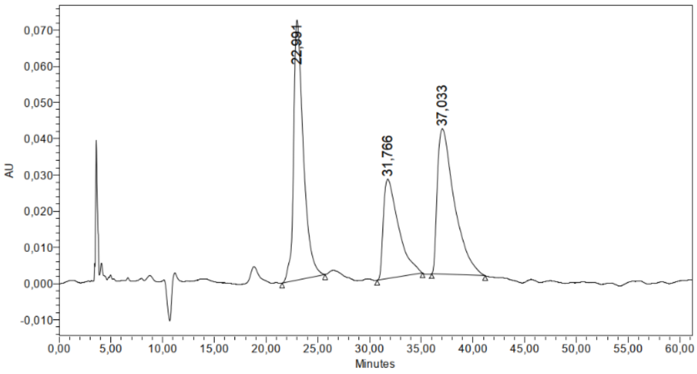

spike experiment (a+b)

	RT	Area	% Area	Height
1	22,991	4715209	38,86	71823
2	31,766	2804670	23,11	27398
3	37,033	4614856	38,03	40080

Column: Chiralpak IB; **Flow rate:** 1.0 mL/min; **Eluent:** Hex: IPA (93:7); **Wavelength:** 230 nm

6.2 HPLC chromatograms of a) 7'a, b) hydration reaction mixture containing 3+7a (SI, optimization table, entry 10)

a)

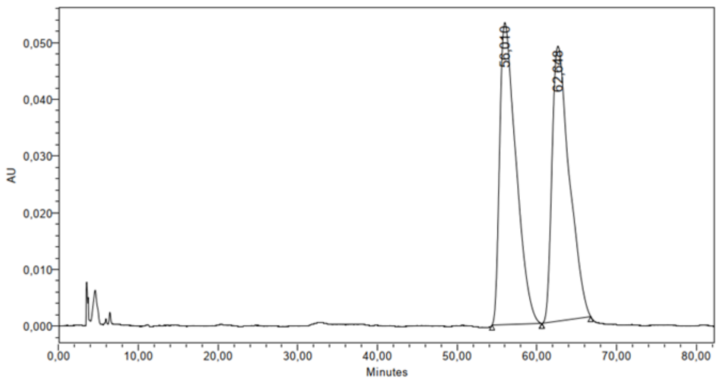

	RT	Area	% Area	Height
1	56,010	7570799	50,89	53203
2	62,648	7306177	49,11	48422

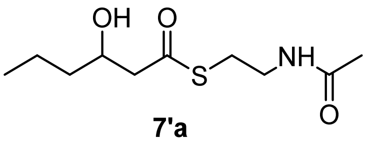

7'a

b)

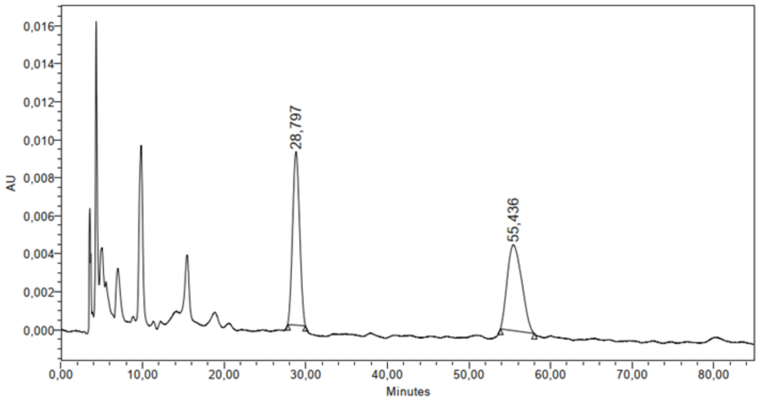

	RT	Area	% Area	Height
1	28,797	542768	49,40	9113
2	55,436	555945	50,60	4515

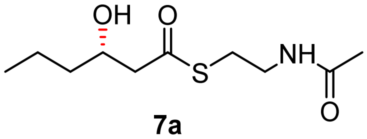

7a

er >99:<1

Column: OZ-H; **Flow rate:** 1.0 mL/min **Solvent system:** Hex:IPA (93:7); **Wavelength:** 230 nm

8.3 HPLC chromatograms of a) 6’b, b) 6b and c) spike experiment (6’b+6b)

a)

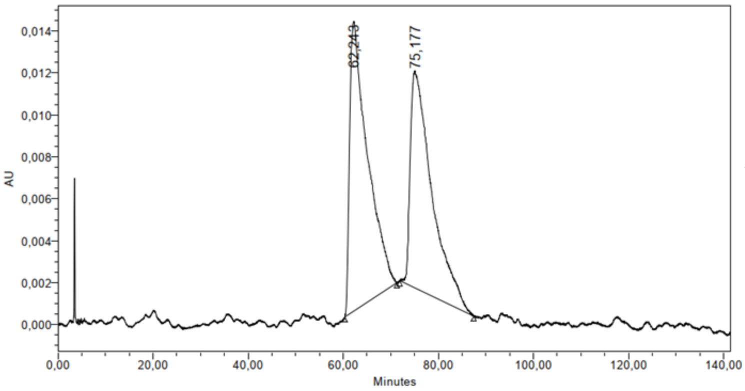

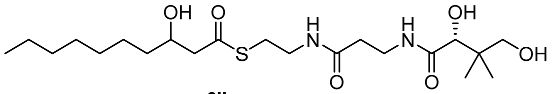

6'b

	RT	Area	% Area	Height
1	62,243	3626584	52,66	13799
2	75,177	3260433	47,34	10333

b)

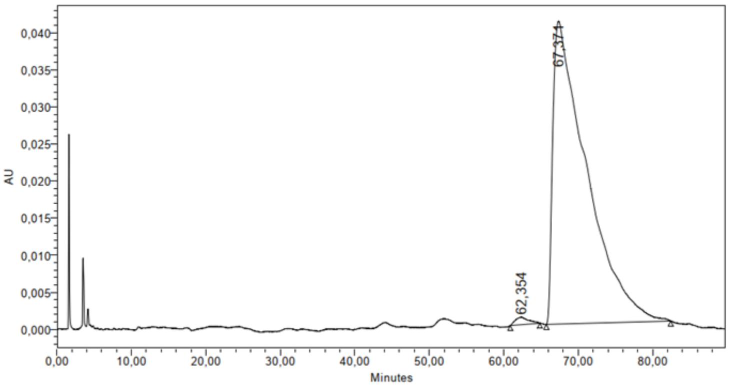

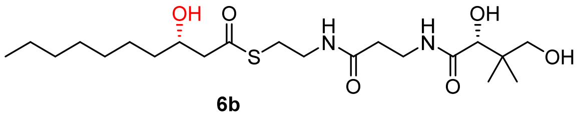

6b

dr >99:<1

	RT	Area	% Area	Height
1	62,354	123987	0,95	1017
2	67,371	12881033	99,05	40854

c)

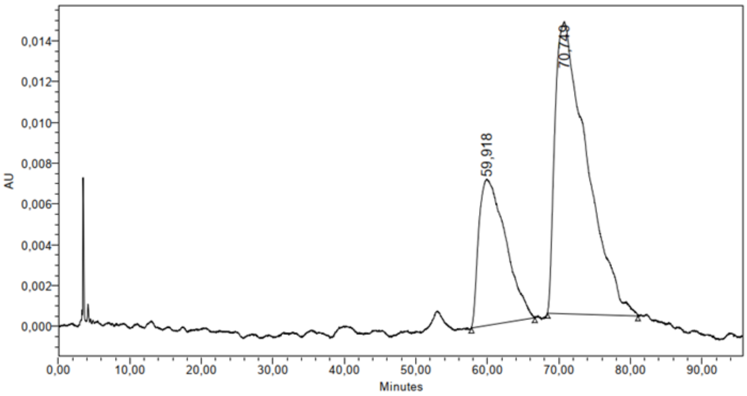

spike experiment (**6’b**+**6b**)

	RT	Area	% Area	Height
1	59,918	1868896	29,63	7172
2	70,749	4438797	70,37	14320

Column: Chiralpak IB; **Flow rate:** 1.0 mL/min; **Eluent:** Hex: IPA (93:7); **Wavelength:** 235 nm

8.4 HPLC chromatograms of a) 6’c, b) 6c and c) spike experiment (6’c+6c)

a)

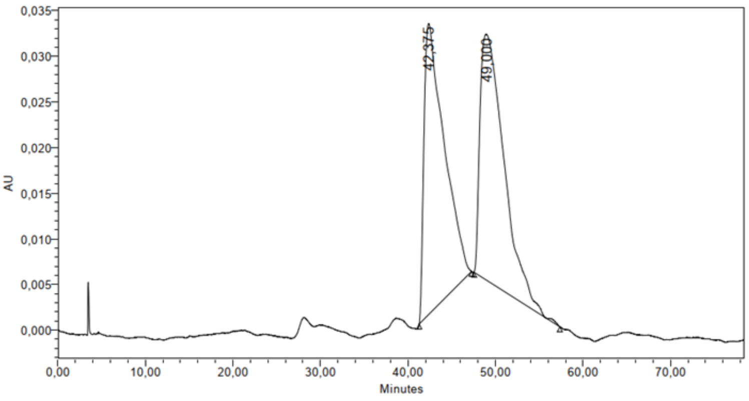

	RT	Area	% Area	Height
1	42,375	5102118	49,78	31874
2	49,000	5147085	50,22	26939

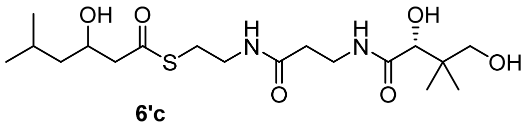

6'c

b)

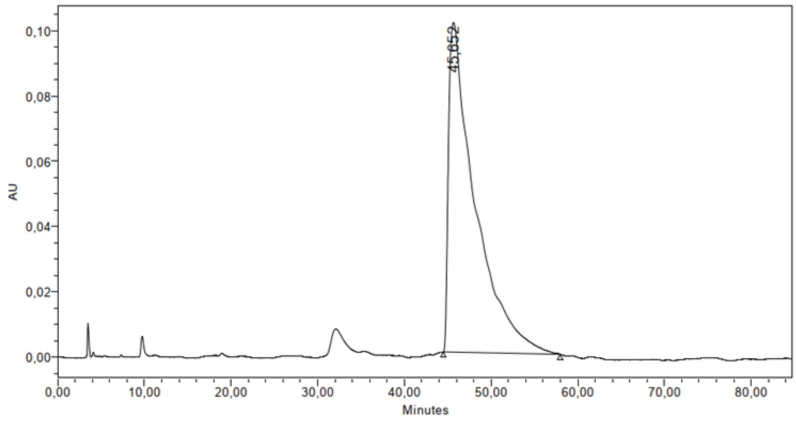

	RT	Area	% Area	Height
1	45,652	21411284	100,00	100904

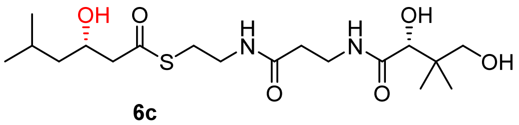

6c

dr >99:<1

c)

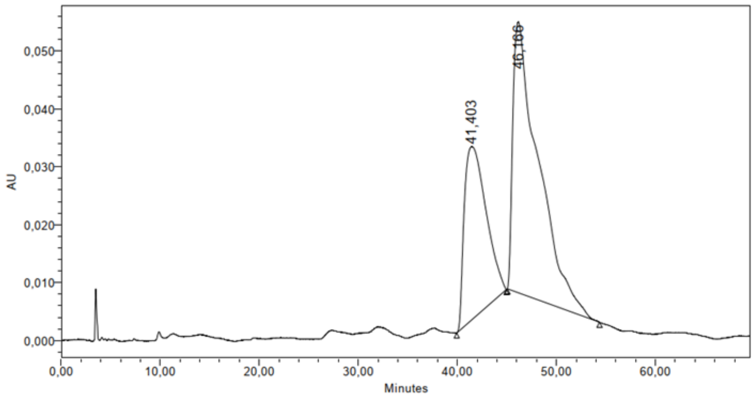

spike experiment (**6’c+6c**)

	RT	Area	% Area	Height
1	41,403	4556320	35,16	29963
2	46,166	8400735	64,84	46754

Column: Chiralpak IB; **Flow rate:** 1.0 mL/min; **Eluent:** Hex:IPA (92:8); **Wavelength:** 235 nm

8.5 HPLC chromatograms of a) 6'd, b) 6d and c) spike experiment (6'd+6d)

a)

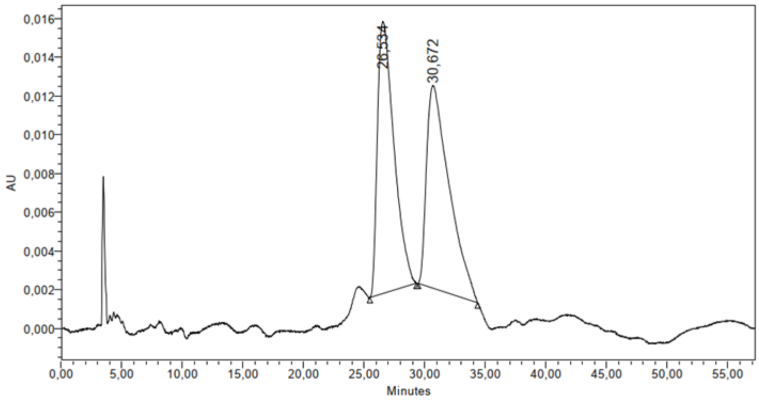

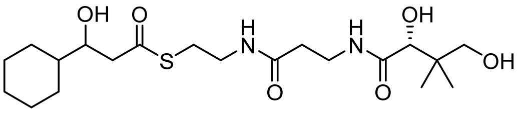

6'd

	RT	Area	% Area	Height
1	26,534	1333372	49,41	14052
2	30,672	1365204	50,59	10460

b)

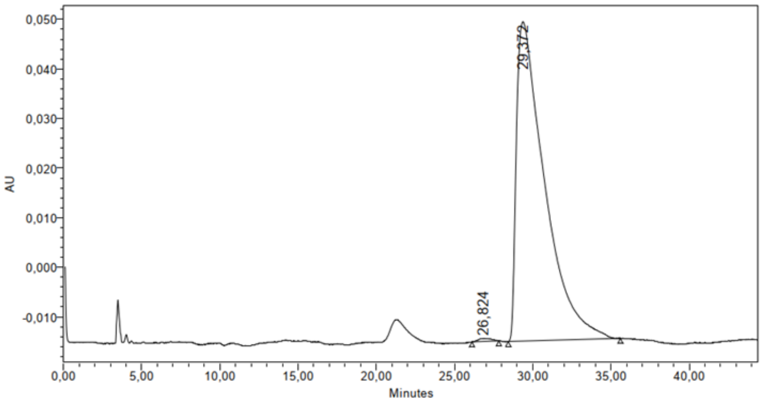

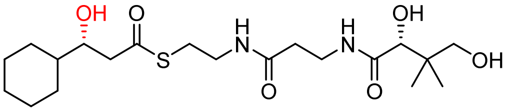

6d

dr >99:<1

	RT	Area	% Area	Height
1	26,824	35721	0,44	619
2	29,372	8056408	99,56	64393

c)

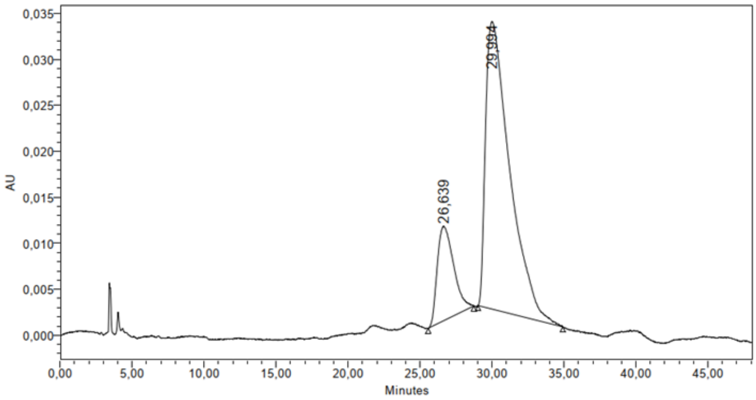

spike experiment (**6'd+6d**)

	RT	Area	% Area	Height
1	26,639	809467	18,22	10292
2	29,994	3632439	81,78	31240

Column: Chiralpak IB; **Flow rate:** 1.0 mL/min; **Eluent:** Hex:IPA (88:12); **Wavelength:** 235 nm

8.6 HPLC chromatograms of a) 6’e, b) 6e

a)

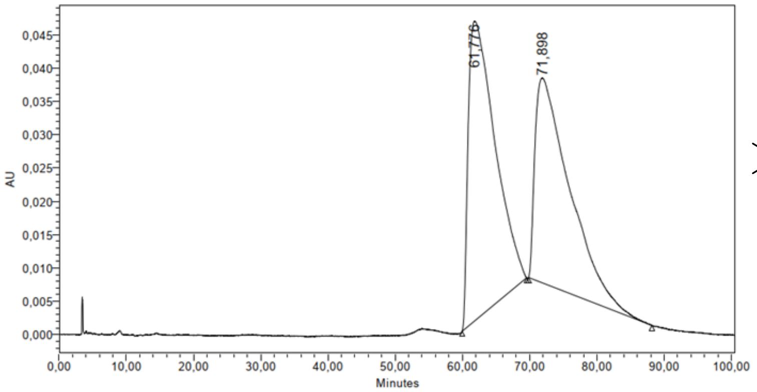

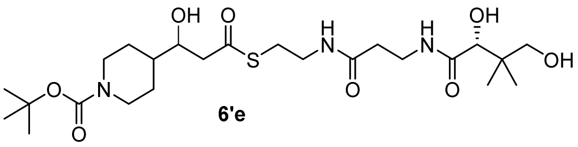

6'e

	RT	Area	% Area	Height
1	61,776	12369411	52,76	45006
2	71,898	11073779	47,24	30753

b)

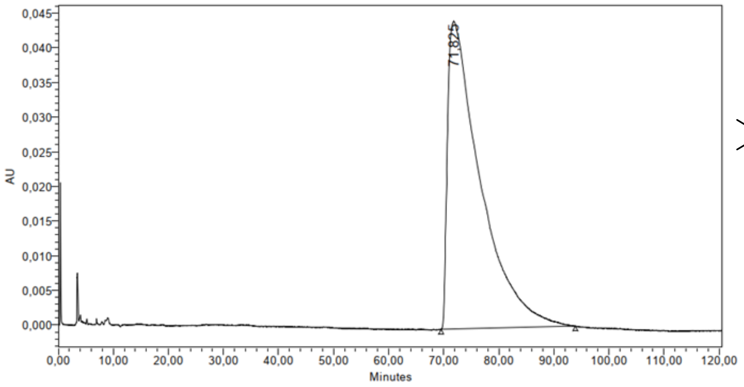

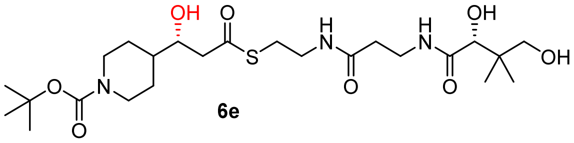

6e

dr >99:<1

	RT	Area	% Area	Height
1	71,825	18367706	100,00	44411

Column: Chiralpak IB; **Flow rate:** 1.0 mL/min; **Eluent:** Hex:IPA (89:11); **Wavelength:** 235 nm

8.7 HPLC chromatograms of a) 6’f, b) 6f and c) spike experiment (6’f+6f)

a)

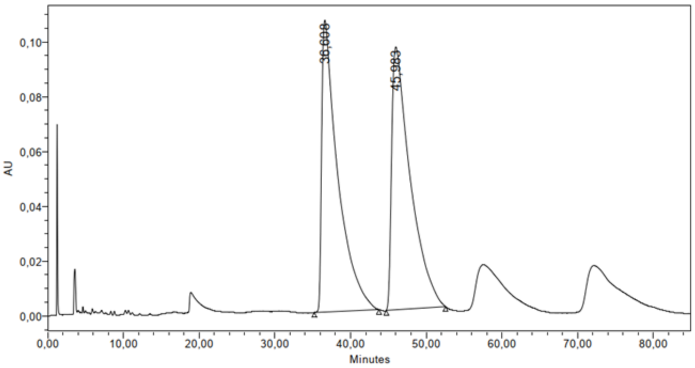

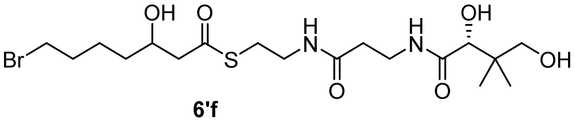

6'f

	RT	Area	% Area	Height
1	36,608	15802522	49,12	106441
2	45,983	16367328	50,88	95919

b)

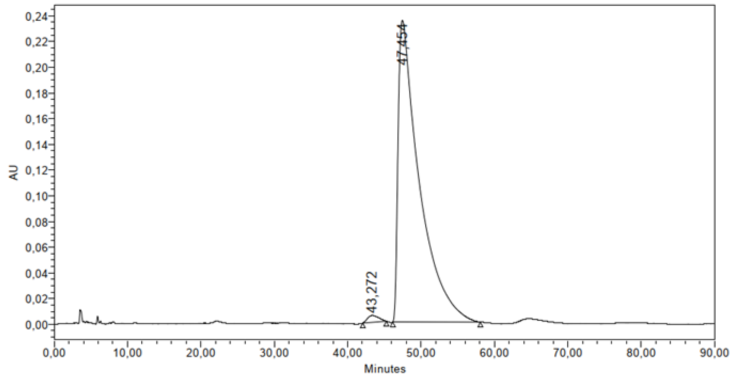

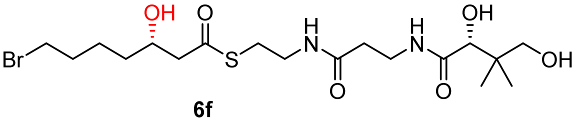

6f

dr >98:<2

	RT	Area	% Area	Height
1	43,272	561033	1,18	5308
2	47,454	46983281	98,82	234594

c)

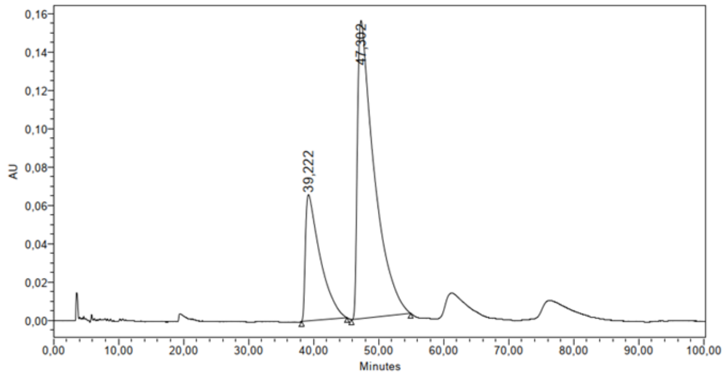

spike experiment (**6’f+6f**)

	RT	Area	% Area	Height
1	39,222	10356842	26,97	65790
2	47,302	28041579	73,03	155381

Column: Chiralpak IC; **Flow rate:** 1.0 mL/min; **Eluent:** Hex:IPA (75:25); **Wavelength:** 230

8.8 HPLC chromatograms of a) 6’g, b) 6g and c) spike experiment (6’g+6g)

a)

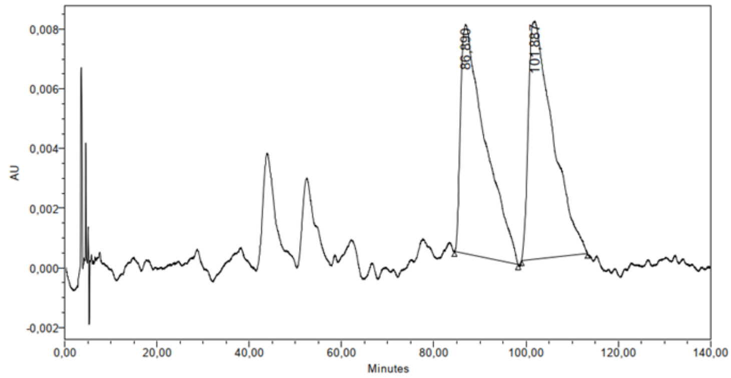

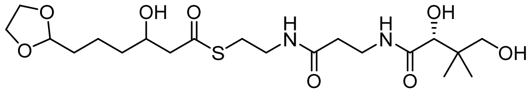

6'g

	RT	Area	% Area	Height
1	86,890	2860731	49,13	7683
2	101,887	2961645	50,87	7983

b)

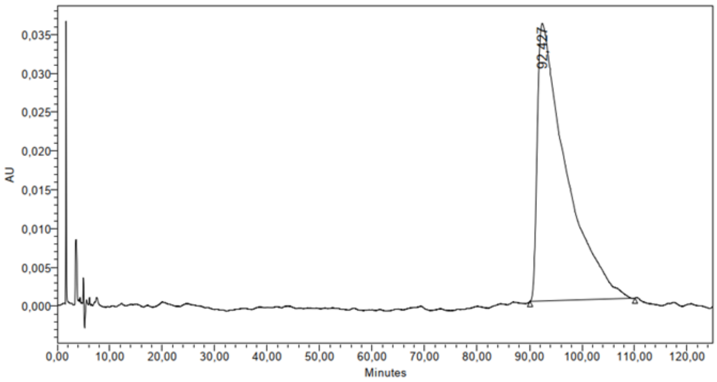

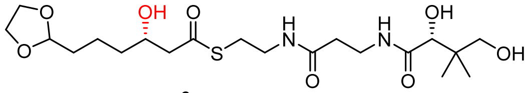

6g

dr >99:<1

	RT	Area	% Area	Height
1	92,427	13540709	100,00	35761

c)

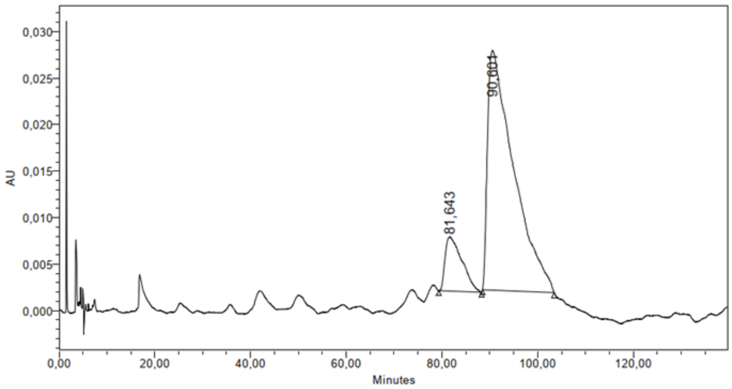

spike experiment (**6’g+6g**)

	RT	Area	% Area	Height
1	81,643	1430002	12,80	5833
2	90,601	9741858	87,20	25763

Column: Chiralpak IC; **Flow rate:** 1.0 mL/min; **Eluent:** Hex:IPA (70:30); **Wavelength:** 230 nm

8.9 HPLC chromatograms of a) 6’h, b) 6h and c) spike experiment (6’h+6h)

a)

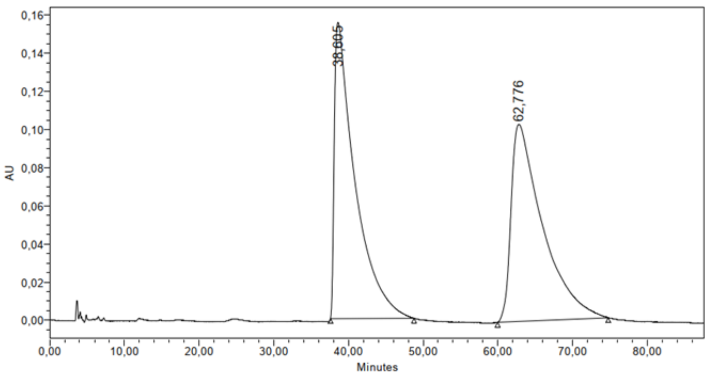

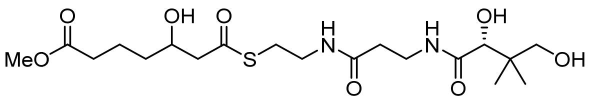

6'h

	RT	Area	% Area	Height
1	38,605	29818187	49,65	155315
2	62,776	30234646	50,35	103378

b)

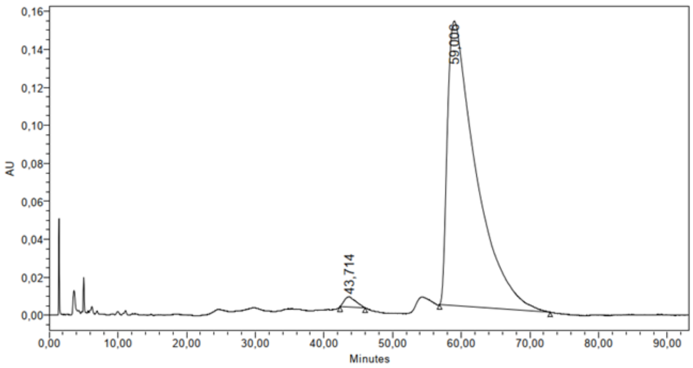

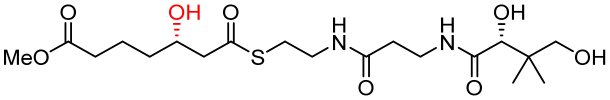

6h

dr >98:<2

	RT	Area	% Area	Height
1	43,714	625375	1,43	5460
2	59,006	43093849	98,57	149717

c)

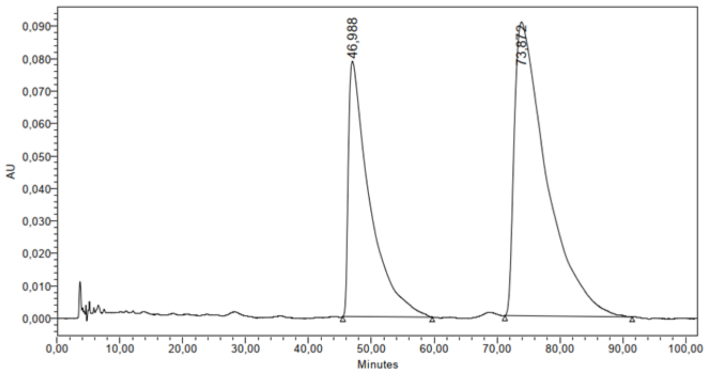

spike experiment (**6’h+6h**)

	RT	Area	% Area	Height
1	46,988	18478010	36,49	78653
2	73,872	32153895	63,51	90519

Column: Chiralpak IC; **Flow rate:** 1.0 mL/min; **Eluent:** Hex:IPA (60:40); **Wavelength:** 230 nm

8.10 HPLC chromatograms of a) 6'i, b) 6i and c) spike experiment (6'i+6i)

a)

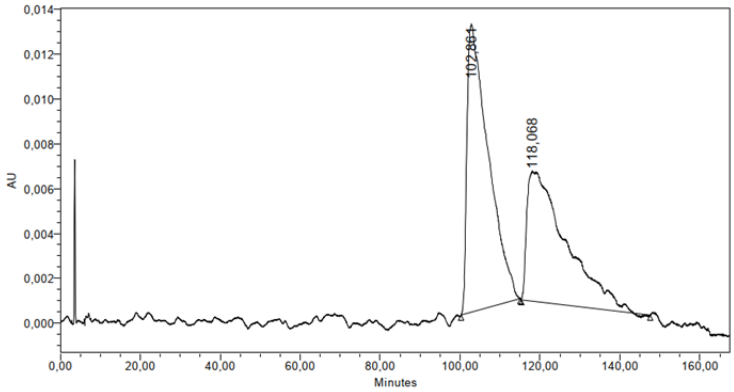

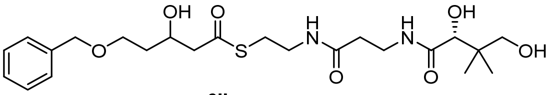

6'i

	RT	Area	% Area	Height
1	102,861	4760214	54,42	12852
2	118,068	3986164	45,58	5797

b)

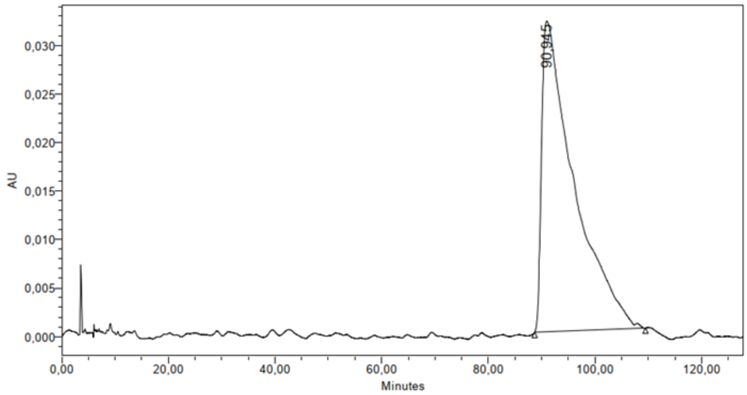

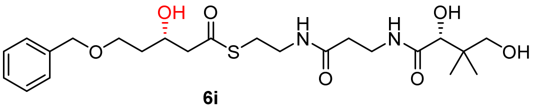

6i

dr >99:<1

	RT	Area	% Area	Height
1	90,945	13455461	100,00	31992

c)

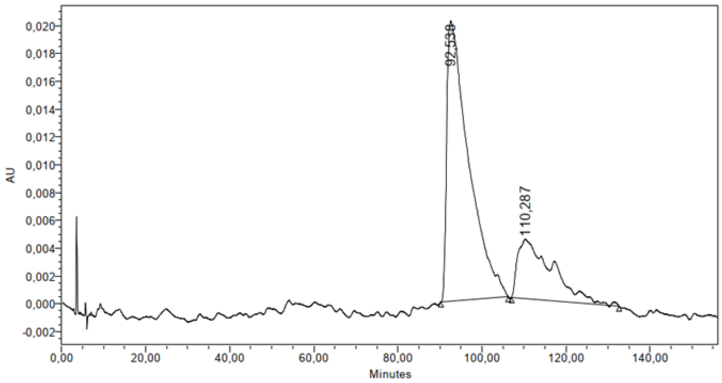

spike experiment (**6'i**+**6i**)

	RT	Area	% Area	Height
1	92,538	7318319	74,82	20163
2	110,287	2462423	25,18	4290

Column: Chiralpak IC; **Flow rate:** 1.0 mL/min; **Eluent:** Hex:IPA (77:23); **Wavelength:** 230 nm

8.11 HPLC chromatograms of a) 6’j, b) 6j and c) spike experiment (6’j+6j)

a)

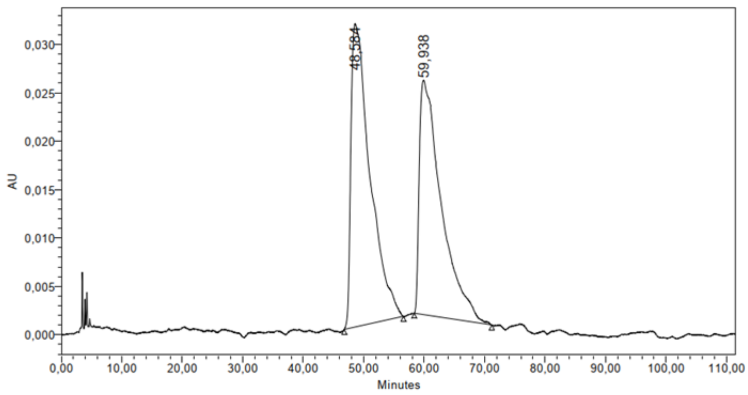

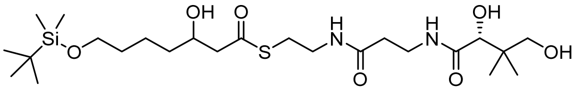

6'j

	RT	Area	% Area	Height
1	48,584	6869371	52,90	31337
2	59,938	6116596	47,10	24217

b)

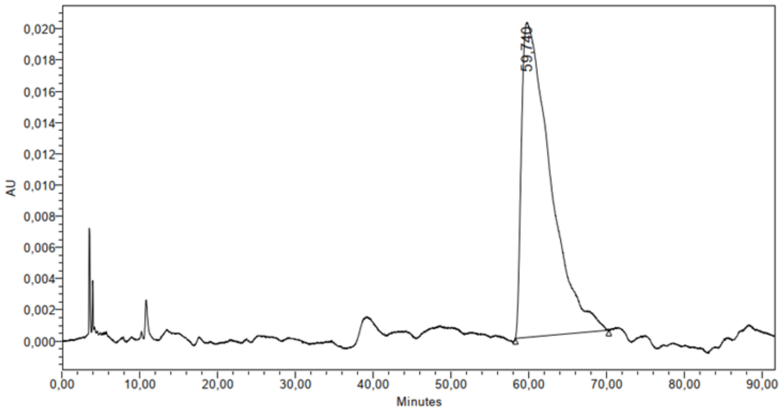

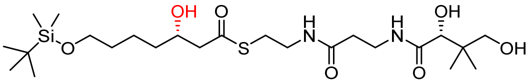

6j

dr >99:<1

	RT	Area	% Area	Height
1	59,740	5184263	100,00	20163

c)

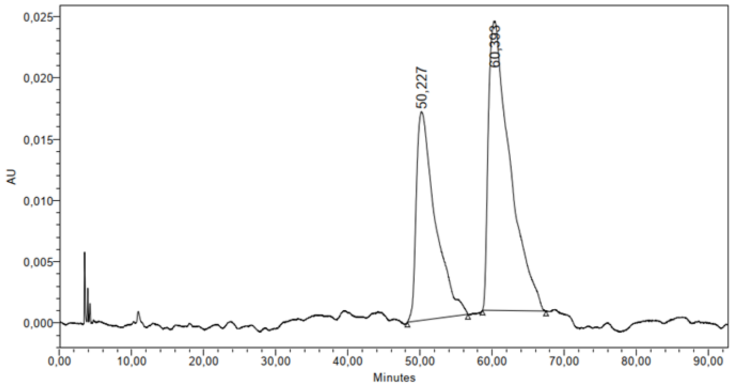

spike experiment (**6’j**+**6j**)

	RT	Area	% Area	Height
1	50,227	3107981	38,79	17031
2	60,393	4904684	61,21	23615

Column: Chiralpak IB; **Flow rate:** 1.0 mL/min; **Eluent:** Hex:IPA (93:7); **Wavelength:** 235 nm

8.12 HPLC chromatograms of a) 6'k, b) 6k and c) spike experiment (6'k+6k)

a)

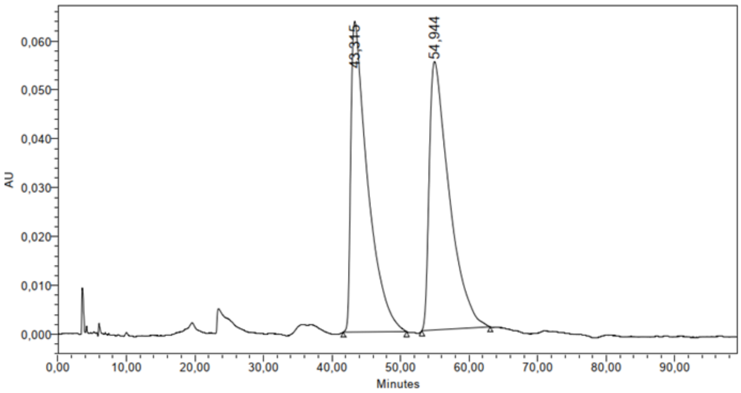

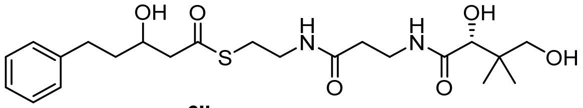

6'k

	RT	Area	% Area	Height
1	43,315	10856974	49,40	63568
2	54,944	11121749	50,60	54933

b)

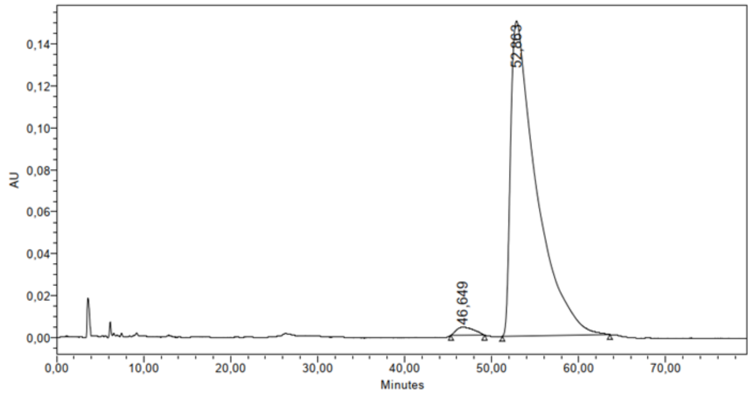

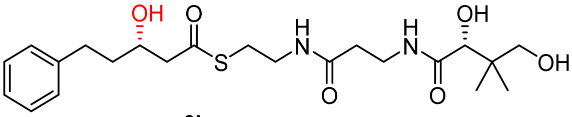

6k

dr >98:<2

	RT	Area	% Area	Height
1	46,649	539626	1,71	3862
2	52,863	30962467	98,29	150113

c)

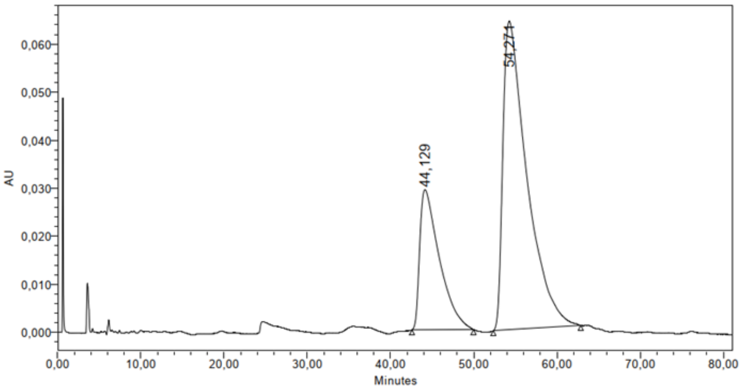

spike experiment (**6'k+6k**)

	RT	Area	% Area	Height
1	44,129	4794105	27,16	29165
2	54,271	12858425	72,84	64225

Column: Chiralpak IC; **Flow rate:** 1.0 mL/min; **Eluent:** Hex:IPA (75:25); **Wavelength:** 230 nm
