# Supplementary material for: Enantioselective Hydration of Non-CoA Enoyl-Thioesters by Enoyl-CoA Hydratase (ECH): Activation of the Active Site Oxyanion Hole with 3′,5′-Adenosine-Diphosphate Enables Competent Catalysis
Source: JACS Au. 2026 Mar 23;6(4):2464–72. doi: 10.1021/jacsau.6c00054 (PMC13126182; doi:10.1021/jacsau.6c00054)
Supplement: Supplementary file 2 [file au6c00054_si_002.zip › NMR fair data_PKM_09012026/Metadata for NMR_PKM_19122025.docx]

**Name of the manufacturer of the spectrometer used to collect the data:**

The ^1^H NMR and ^13^C{^1^H} NMR spectra were recorded in either CDCl_3_, or D_4_-MeOH on Bruker Avance 500 MHz, 400 MHz and 300 MHz NMR spectrometers. The chemical shifts are reported in ppm relative to CHCl_3_ (δ =7.26), D_4_-MeOH (δ =3.31) for ^1^H NMR. For the ^13^C{^1^H} NMR spectra, the residual CDCl_3_ (δ=77.16), D_4_-MeOH (δ =49.0) were used as the internal standards.

**Acquisition software:** Bruker TopSpin 3.5 pl 7 on 500 MHz, Bruker TopSpin 400 MHz and Bruker TopSpin 3.5 pl 6 on NMR 300 MHz.

**Processing programs used to analyse the data:** MestReNova 15.1.0-38027

**The field strength used to measure each nucleus:**

**S1b**: **1H NMR** (300 MHz, MeOD), **^13^C{^1^H} NMR** (75 MHz, MeOD)

**3**: **^1^H NMR** (500 MHz, MeOD), **^13^C{^1^H} NMR** (126 MHz, MeOD)

**4a**: **^1^H NMR** (500 MHz, MeOD), **^13^C{^1^H} NMR** (126 MHz, MeOD)

**4b**: **^1^H NMR** (500 MHz, MeOD), **^13^C{^1^H} NMR** (126 MHz, MeOD)

**4c**: **^1^H NMR** (500 MHz, MeOD), **^13^C{^1^H} NMR** (126 MHz, MeOD)

**S6b**: **^1^H NMR** (300 MHz, CDCl_3_), **^13^C{^1^H} NMR** (75 MHz, CDCl_3_)

**4d**: **^1^H NMR** (500 MHz, MeOD), **^13^C{^1^H} NMR** (126 MHz, MeOD)

**S7b**: **^1^H NMR** (500 MHz, CDCl_3_), **^13^C{^1^H} NMR** (126 MHz, CDCl_3_)

**4e**: **^1^H NMR** (500 MHz, MeOD), **^13^C{^1^H} NMR** (126 MHz, MeOD)

**S8b**: **^1^H NMR** (500 MHz, CDCl_3_), **^13^C{^1^H} NMR** (126 MHz, CDCl_3_),

**S8c**: **^1^H NMR** (500 MHz, CDCl_3_), **^13^C{^1^H} NMR** (126 MHz, CDCl_3_)

**S8d**: **^1^H NMR** (300 MHz, CDCl_3_), **^13^C{^1^H} NMR** (75 MHz, CDCl_3_)

**4f**: **^1^H NMR** (500 MHz, MeOD), **^13^C{^1^H} NMR** (126 MHz, MeOD)

**S9a**: **^1^H NMR** (500 MHz, CDCl_3_), **^13^C{^1^H} NMR** (126 MHz, CDCl_3_)

**S9b**: **^1^H NMR** (500 MHz, CDCl_3_)

**S9c**: **^1^H NMR** (500 MHz, CDCl_3_)

**4g**: **^1^H NMR** (500 MHz, MeOD), **^13^C{^1^H} NMR** (126 MHz, MeOD)

**S10b**: **^1^H NMR** (500 MHz, CDCl_3_), **^13^C{^1^H} NMR** (126 MHz, CDCl_3_)

**4h**: **^1^H NMR** (500 MHz, MeOD), **^13^C{^1^H} NMR** (126 MHz, MeOD)

**S11b**: **^1^H NMR** (300 MHz, CDCl_3_), **^13^C{^1^H} NMR** (75 MHz, CDCl_3_)

**4i:** **^1^H NMR** (500 MHz, MeOD), **^13^C{^1^H} NMR** (126 MHz, MeOD)

**S12b**: **^1^H NMR** (300 MHz, CDCl_3_), **^13^C NMR** (75 MHz, CDCl3)

**4j**: **^1^H NMR** (500 MHz, MeOD), **^13^C{^1^H} NMR** (126 MHz, MeOD)

**S13b**: **^1^H NMR** (300 MHz, CDCl_3_), **^13^C NMR** (75 MHz, CDCl_3_)

**4k**: **^1^H NMR** (500 MHz, MeOD), **^13^C{^1^H} NMR** (126 MHz, MeOD)

**6a**: **^1^H NMR** (500 MHz, MeOD), **^13^C{^1^H} NMR** (126 MHz, MeOD)

**6b**: **^1^H NMR** (500 MHz, MeOD), **^13^C{^1^H} NMR** (126 MHz, MeOD)

**6C**: **^1^H NMR** (300 MHz, MeOD), **^13^C{^1^H} NMR** (75 MHz, MeOD)

**6d**: **^1^H NMR** (300 MHz, MeOD), **^13^C{^1^H} NMR** (75 MHz, MeOD)

**6e**: **^1^H NMR** (500 MHz, MeOD), **^13^C{^1^H} NMR** (126 MHz, MeOD)

**6f**: **^1^H NMR** (500 MHz, MeOD), **^13^C{^1^H} NMR** (126 MHz, MeOD)

**6g**: **^1^H NMR** (500 MHz, MeOD), **^13^C{^1^H} NMR** (126 MHz, MeOD)

**6h**: **^1^H NMR** (500 MHz, MeOD), **^13^C{^1^H} NMR** (126 MHz, MeOD)

**6i**: **^1^H NMR** (500 MHz, MeOD), **^13^C{^1^H} NMR** (126 MHz, MeOD)

**6j**: **^1^H NMR** (500 MHz, MeOD), **^13^C{^1^H} NMR** (126 MHz, MeOD)

**6k**: **^1^H NMR** (300 MHz, MeOD), **^13^C{^1^H} NMR** (75 MHz, MeOD)

**S19b**: **^1^H NMR** (500 MHz, CDCl_3_), **^13^C{^1^H} NMR** (126 MHz, CDCl_3_)

**6’a**: **^1^H NMR** (500 MHz, MeOD), **^13^C{^1^H} NMR** (126 MHz, MeOD)

**S20b**: **^1^H NMR** (500 MHz, CDCl_3_), **^13^C{^1^H} NMR** (126 MHz, CDCl_3_)

**6’b**: **^1^H NMR** (300 MHz, MeOD), **^13^C{^1^H} NMR** (75 MHz, MeOD)

**S21a**: **^1^H NMR** (300 MHz, CDCl_3_), **^13^C{^1^H} NMR** (75 MHz, CDCl_3_)

**6’c**: **^1^H NMR** (500 MHz, MeOD), **^13^C{^1^H} NMR** (126 MHz, MeOD)

**S22b**: **^1^H NMR** (500 MHz, MeOD), **^13^C{^1^H} NMR** (126 MHz, MeOD)

**6’d**: **^1^H NMR** (300 MHz, MeOD), **^13^C{^1^H} NMR** (75 MHz, MeOD)

**S23b**: **^1^H NMR** (500 MHz, MeOD), **^13^C{^1^H} NMR** (126 MHz, MeOD)

**6’e**: **^1^H NMR** (500 MHz, MeOD), **^13^C{^1^H} NMR** (126 MHz, MeOD)

**S25b**: **^1^H NMR** (300 MHz, CDCl_3_), **^13^C{^1^H} NMR** (75 MHz, CDCl_3_)

**S25c**: **^1^H NMR** (500 MHz, CDCl_3_), **^13^C{^1^H} NMR** (126 MHz, CDCl_3_)

**S25d**: **^1^H NMR** (500 MHz, CDCl_3_), **^13^C{^1^H} NMR** (75 MHz, CDCl_3_)

**S24a**: **^1^H NMR** (500 MHz, CDCl_3_), **^13^C{^1^H} NMR** (126 MHz, CDCl_3_)

**S24b**: **^1^H NMR** (300 MHz, CDCl_3_), **^13^C{^1^H} NMR** (75 MHz, CDCl_3_)

**6’f**: **^1^H NMR** (400 MHz, MeOD), **^13^C{^1^H} NMR** (101 MHz, MeOD)

**S27b**: **^1^H NMR** (400 MHz, CDCl_3_), **^13^C{^1^H} NMR** (75 MHz, CDCl_3_)

**S27c: ^1^H NMR** (300 MHz, CDCl_3_), **^13^C{^1^H} NMR** (75 MHz, CDCl_3_)

**S27d: ^1^H NMR** (300 MHz, CDCl_3_), **^13^C{^1^H} NMR** (75 MHz, CDCl_3_)

**S27e: ^1^H NMR** (300 MHz, CDCl_3_), **^13^C{^1^H} NMR** (75 MHz, CDCl_3_)

**S26a: ^1^H NMR** (300 MHz, CDCl_3_), **^13^C{^1^H} NMR** (75 MHz, CDCl_3_)

**S26c: ^1^H NMR** (300 MHz, CDCl_3_), **^13^C{^1^H} NMR** (75 MHz, CDCl_3_)

**6’g:** **^1^H NMR** (300 MHz, MeOD), **^13^C{^1^H} NMR** (75 MHz, MeOD)

**S28a**: **^1^H NMR** (500 MHz, CDCl_3_), **^13^C{^1^H} NMR** (75 MHz, CDCl_3_)

**6’h:** **^1^H NMR** (300 MHz, MeOD), **^13^C{^1^H} NMR** (75 MHz, MeOD)

**S29b**: **^1^H NMR** (500 MHz, MeOD), **^13^C{^1^H} NMR** (126 MHz, MeOD)

**6’i:** **^1^H NMR** (300 MHz, MeOD), **^13^C{^1^H} NMR** (75 MHz, MeOD)

**S30a**: **^1^H NMR** (500 MHz, CDCl_3_), **^13^C{^1^H} NMR** (126 MHz, CDCl_3_)

**S30b: ^1^H NMR** (300 MHz, CDCl_3_), **^13^C{^1^H} NMR** (75 MHz, CDCl_3_)

**S30c: ^1^H NMR** (300 MHz, CDCl_3_), **^13^C{^1^H} NMR** (75 MHz, CDCl_3_)

**6’j:** **^1^H NMR** (300 MHz, MeOD), **^13^C{^1^H} NMR** (75 MHz, MeOD)

**S31b**: **^1^H NMR** (500 MHz, MeOD), **^13^C{^1^H} NMR** (126 MHz, MeOD)

**6’k:** **^1^H NMR** (300 MHz, MeOD), **^13^C{^1^H} NMR** (75 MHz, MeOD)

**7’a:** **^1^H NMR** (300 MHz, MeOD), **^13^C{^1^H} NMR** (75 MHz, MeOD)
